# FLP Mediated Conversion of Difluorobenzylalkynes to Fluoroallenyl Oniums via an S_N_1’ Pathway

**DOI:** 10.1002/chem.71213

**Published:** 2026-06-02

**Authors:** Max Coles, Tianhong Wang, Rubi McLennan, Long Kit Kwan, Bivas Mondal, Chakyu Richard Chan, Rowan D. Young

**Affiliations:** ^1^ School of Chemistry and Molecular Biosciences University of Queensland Brisbane Australia; ^2^ Department of Chemistry National University of Singapore Singapore Singapore

**Keywords:** allene, defluorination, FLP, fluorocarbon, S_N_1’

## Abstract

Fluoroallenes represent an attractive class of synthons and are congeners to naturally occurring haloallenes, yet their preparation remains challenging. We report that frustrated Lewis pair (FLP) mediated selective monodefluorination of difluorobenzylalkynes generates geminal fluorobenzylalkyne onium salts as kinetic products, which subsequently isomerize into thermodynamically preferred fluoroallenyl onium salts through an S_N_1′pathway. The onium‐stabilized fluoroallenes can undergo further derivatization at both allenic and onium positions, providing modular access to new fluorocarbon architectures.

## Graphical abstract

Frustrated Lewis pair (FLP) selective C‒F activation of difluorobenzylalkynes allows the formation of fluoroallenic oniums via an S_N_1’ substitution pathway. The fluoroallenic onium salts are synthetic precursors that allow subsequent functionalization at allenic and onium positions.

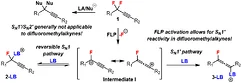

## 1 Introduction

The inclusion of fluorine into organic molecules imparts advantageous characteristics, including increased metabolic stability, improved solubilities, and a useful spectroscopic handle [1, 2, 3]. More recently, the ability of fluorine‐containing motifs to act as bioisosteres has been exploited in pharmaceutical and agrochemical discovery [4, 5]. To this end, ready access to a more expansive variety of fluorinated functional groups is vital to further explore the bioisostericity of fluorine‐containing groups.

Selective defluorination of polyfluorinated molecules has emerged as a powerful technique to access an array of second‐generation fluorocarbons from single polyfluorocarbon precursors [6, 7, 8, 9, 10, 11, 12, 13, 14, 15]. Numerous methods now allow for selective defluorination of CF_3_ and CF_2_R groups, including reduction of trifluoromethyl groups [16, 17, 18, 19, 20, 21, 22, 23, 24, 25, 26, 27], metal‐catalyzed selective defluorination [28, 29, 30, 31, 32, 33], Lewis acid mediated defluorination of aryl polyfluoromethyl groups *ortho* to hydrosilanes [34, 35, 36, 37], and frustrated Lewis pair (FLP) mediated defluorination of polyfluoromethyl groups (where a fluoride is abstracted by the Lewis acid and the concomitantly generated fluorocarbocation is captured by the Lewis base) [38, 39, 40, 41, 42, 43, 44].

However, the earliest approach for selective defluorination involved the monodefluorination of trifluoromethyl alkene via an S_N_2’ attack to generate a difluoroalkene product [45]. This approach was expanded upon [46, 47, 48, 49, 50, 51, 52, 53, 54, 55, 56, 57, 58, 59], and has also been demonstrated for S_N_1’ [60, 61, 62, 63] and metal‐catalyzed [64] pathways with a variety of allyl polyfluorides and in both cases relies upon the conversion of residual C‒F positions from sp^3^ hybridized to more thermodynamically stable sp^2^ hybridized (Figure 1a).

**FIGURE 1:**
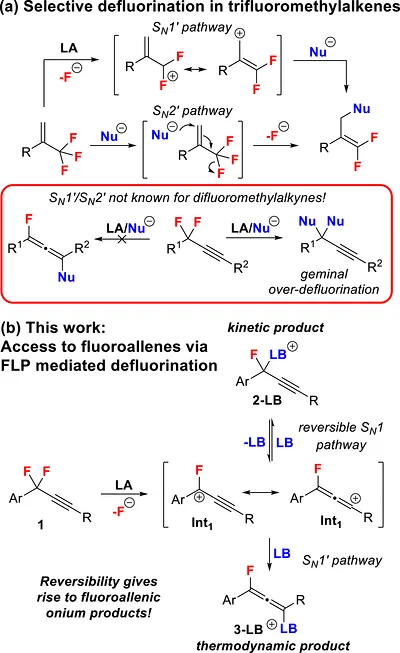
(a) Selective activation of allylic fluorides via S_N_1’ or S_N_2’ pathways is well known but generally not applicable to propargyl fluorides. (b) Reversible access to intermediate **Int_1_** via fluoride abstraction from **1** or Lewis base dissociation from **2‐LB** allows rearrangement to form fluoroallenes **3‐LB**. FLP, frustrated Lewis pair; LA, Lewis acid; LB, Lewis base.

We recently disclosed a mechanism for FLP‐mediated C‐F activation of benzodifluorides [65], which hinged upon reversible Lewis base dissociation from transient fluorobenzylium intermediates. Utilizing this information, we developed methods for difluoromethyl desymmetrization and radiofluorination [66, 67]. We hypothesized that similar access to fluorobenzylium intermediates from difluorobenzylalkynes would allow for 1,3‐migration of Lewis bases to generate fluoroallenes (Figure 1b). Direct generation of fluoroallenes from propargyl difluorides via S_N_1’ or S_N_2’ pathways (analogous to allyl fluorides) is unknown and complicated by both the fact that fluoride acts as a poor leaving group and that *geminal* substitution competes with nucleophilic attack of the alkynyl position [68, 69].

Fluoroallenes are generally difficult to access, requiring mixed fluoro‐halide precursors, ketenes, or metal catalysis [70, 71, 72, 73, 74]. However, numerous reports have highlighted the importance of such motifs as synthons in accessing diverse molecular architecture, in addition to the prevalence of allenes in natural products and associated interest in fluorinated derivatives of such biomolecules [75, 76, 77, 78, 79, 80]. Concurrently, difluoromethylalkynes have become an increasingly utilized starting point in chemical synthesis [81, 82]. Herein, we report on a simple and robust FLP‐mediated approach to generating fluorine‐substituted allenyl onium salts that provide multiple avenues for further functionalization. The resultant products are electrophilic alleniums, whose chemistry is under‐reported and distinct from classical neutral allenes [83].

## 2 Results and Discussion

To test whether FLP‑mediated monodefluorination could furnish fluoroallenic products, model substrate **1a** was treated with BF_3_·OEt_2_ and P(*o*‑Tol)_3_. Initially, the reaction generated a mixture of two monodefluorinated substitution products (**2a‐PTol_3_** and **3a‐PTol_3_**) in a 42:58 ratio (Table 1, entry 1). Compound **2a‐PTol_3_** subsequently isomerized exclusively to **3a‐PTol_3_** quantitatively (adjudged ^19^F NMR) over a matter of hours (Table 1, entry 2).

**TABLE 1:** Activation of **1a** with various Lewis bases (**LB**) to generate **2a‐LB** and **3a‐LB**.

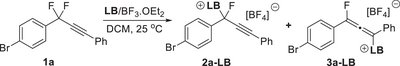				
Entry^a^	Lewis base (LB)	Time	yield	ratio (2a:3a)
1	P(*o*‐Tol)_3_	10 min	70%	42:58
2	P(*o*‐Tol)_3_	2 h	> 95%	0:100
3	PPh_3_	10 min	65%	75:25
4	PPh_3_	2 h	90%	75:25
5	PCy_3_	12 h	80%	67:33
6	PMes_3_	2 h	> 95%	0:100
7	TPPy	2 h	> 95%	0:100
8	THT	2 h	85%	0:100
9^b^	P(*o*‐Tol)_3_	2 h	> 95%	0:100

^a^ General conditions: 1.0 equiv. Lewis base, 1.0 equiv. BF_3_.OEt_2_, ca. 80 mM in CH_2_Cl_2_, 25°C.

^b^ Conditions: 10 mol % B(C_6_F_5_)_3_, 1.0 equiv. Me_3_SiNTf_2_, ca. 60 mM in CH_2_Cl_2_, 25°C. TPPy = 2,4,6‐triphenylpyridine, THT = tetrahydrothiophene. Yields determined through ^19^F NMR analysis.

Compounds **2a‐PTol_3_** and **3a‐PTol_3_** were identified as the α and γ substitution products. These compounds are easily distinguishable through their relative ^31^P─^19^F couplings associated with their well‐resolved ^31^P NMR signals at 31.3 ppm (d, ^2^ *J*_PF_ = 99.3 Hz) for **2a‐PTol_3_** and 20.3 ppm (d, ^4^ *J*_PF_ = 7.0 Hz) for **3a‐PTol_3_**. ^31^P─^19^F couplings in the range of 70–100 Hz are characteristic of α‐fluoroalkylphosphoniums [38]. The ^13^C{^1^H} NMR spectrum of **3a‐PTol_3_** provided conclusive evidence for the formation of an allene with the central allenic C2^13^C NMR resonance identified at 212.5 ppm as a broad doublet. Room temperature ^19^F NMR analysis of **3a‐PTol_3_** revealed two broad signals at −134.0 and −146.0 ppm. These signals begin to coalesce at 313 K and likely represent atropodiastereomers arising from hindered rotation of the phosphino tolyl groups and the axial chirality of the allene. Indeed, at room temperature, a single methyl signal assigned to the phosphino tolyl groups is observed in the ^1^H NMR spectrum of **3a‐PTol_3_** at δ_H_ 2.05, which broadens at 288 K and resolves into two signals (δ_H_ 2.30 and 1.90) below 268 K (Figures S3 and S4).

Repeating the reaction of **1a** with BF_3_·OEt_2_ and PPh_3_ gave a distribution of **2a‐PPh_3_** and **3a‐PPh_3_** in a 75:25 ratio that failed to isomerize under ambient conditions (Table 1, entries 3 and 4). Heating this mixture at 40°C overnight led to decomposition of **2a‐PPh_3_** without any significant increase in the concentration of **3a‐PPh_3_**. Similarly, the use of PCy_3_ gave predominantly the α‐product (**2a‐PCy_3_**: **3a‐PCy_3_** = 67:33) that failed to isomerize and led to decomposition of **2a‐PCy_3_** with overnight heating (Table 1, entry 5). Conversely, the use of PMes_3_ as the Lewis base partner resulted exclusively in the formation of fluoroallene product **3a‐PMes_3_**. At room temperature, the two atropodiastereomers of **3a‐PMes_3_** gave rise to well‐resolved signals in the ^19^F NMR spectrum at −134.1 and −148.3 ppm, presumably due to the higher barrier to rotation of the mesityl groups (cf. tolyl).

These results led us to suspect that isomerization from alkyne to the allene products was only possible with Lewis bases that provided facile access to cationic intermediate **Int_1a_** (Figure 1b). As such, we repeated the experiments with 2,4,6‐triphenylpyridine (TPPy) and tetrahydrothiophene (THT), Lewis bases that we have shown to be better nucleofuges than P(*o*‐Tol)_3_ [65]. In these reactions, we exclusively observed the allenic products **3a‐TPPy** and **3a‐THT** (Table 1, entries 7 and 8). Finally, we demonstrated that this reaction is achievable under catalytic conditions, using B(C_6_F_5_)_3_ catalyst with stoichiometric Me_3_SiNTf_2_ to generate the bistriflimide salt (Table 1, entry 9).

To understand the process of isomerization, we designed a series of control and computational experiments. Initial rate experiments established that the reaction was zero‐order in PTol_3_, supporting the hypothesis that **3a‐PTol_3_** was formed via an S_N_1’ process (Figures S1). Conducting an Eyring analysis over the temperature range of 288–308 K determined a kinetic barrier of Δ*G* ^‡^ = 23 ± 3 kcal mol^−1^ (Figures 2 and S2). This value is comparable to the previously calculated barrier of 23.8 kcal mol^−1^ for dissociation of TPPy from [PhCF_2_]^+^ [65].

**FIGURE 2:**
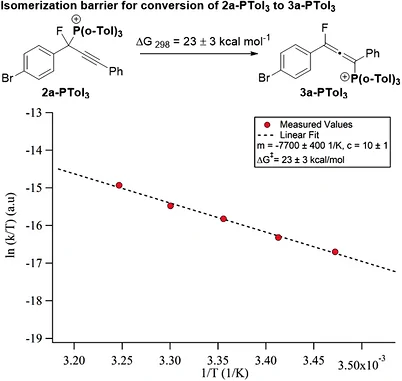
A kinetic barrier of Δ*G* ^‡^ = 23 ± 3 kcal mol^−1^ was experimentally determined via Eyring analysis for the conversion of **2a‐PTol_3_** to **3a‐PTol_3_**. Counter anion (NTf_2_) omitted. See Supporting Information for experimental details.

DFT calculations at the B3LYP/6‐31G(d,p)/SMD(CH_2_Cl_2_) level of theory were undertaken on the proposed intermediate [(4‐BrC_6_H_4_CFCCPh]^+^ (**Int_1a_**), arising from fluoride abstraction from **1a** (Figure 3a). The LUMO of **Int_1a_** is delocalized across the α‐ and γ‐positions (C_A_ and C_B_); however, electrophilic Fukui indices (*ƒ* ^+^) indicate the more electrophilic position to be C_A_, attack of which generates products **2a‐LB** as the kinetically favored products. Free energy calculations indicate that conversion of **2‐PTol_3_** to **3‐PTol_3_** is exergonic by 9.9 kcal mol^−1^, providing thermodynamic incentive for complete conversion of **2a‐PTol_3_** to **3‐PTol_3_** (Figure 3b,d).

**FIGURE 3:**
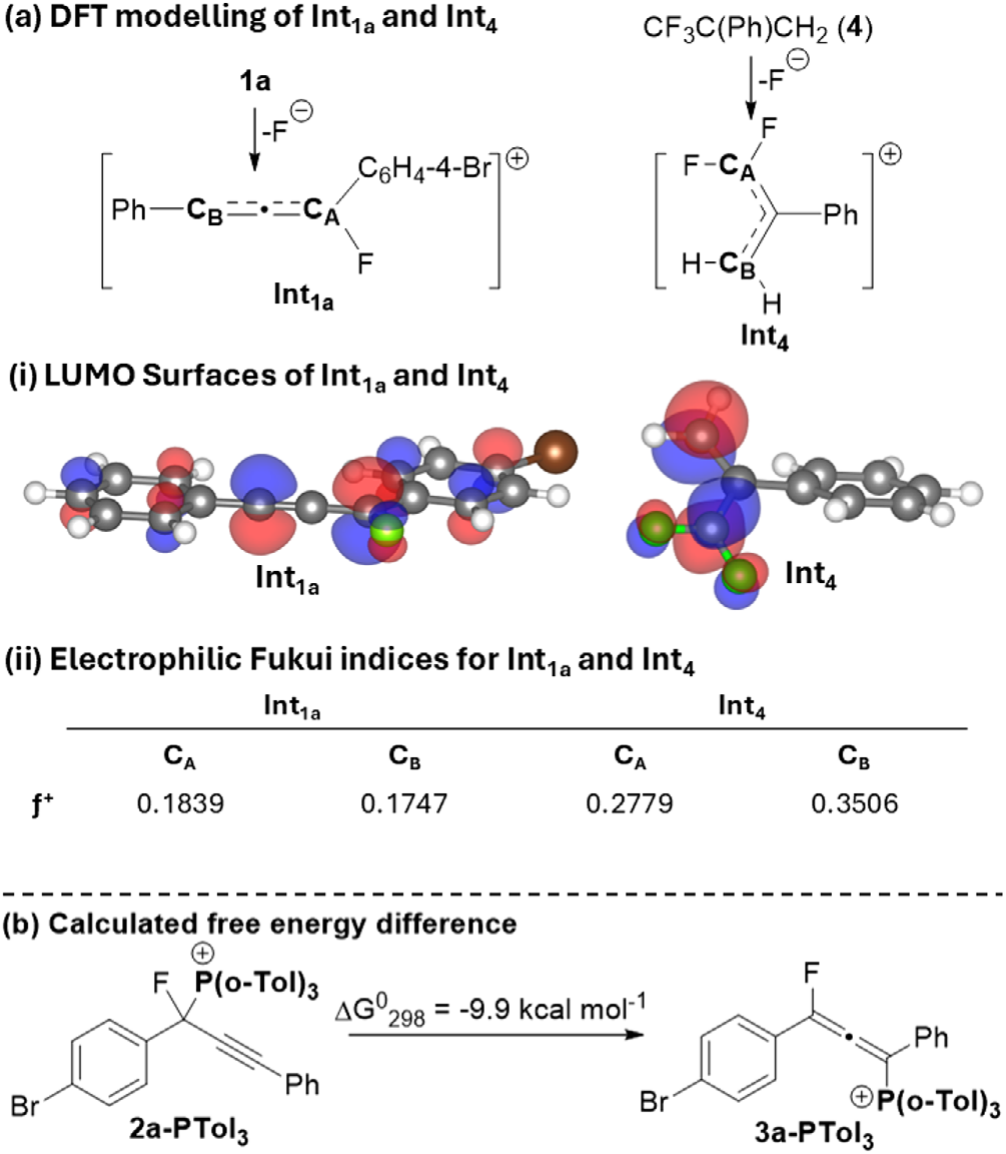
(a) Calculated results for [PhCCC(F)C_6_H_4_‐4‐Br]^+^ (**Int_1a_**) and [CF_2_C(Ph)CH_2_]^+^ (**Int_4_**). (i) LUMO surfaces for **Int_1a_** and **Int_4_** (contour value = 0.05 a.u.). (ii) Electrophilic Fukui indices (*f* ^+^) for positions C_A_ and C_B_ in **Int_1a_** and **Int_4_** that indicate C_A_ is more electrophilic in **Int_1a_**, while C_B_ is more electrophilic in **Int_4_** (b) The Conversion of **2a‐PTol_3_** to **3a‐PTol_3_** was calculated to be exergonic by 9.9 kcal mol^−1^. Level of theory: B3LYP/6‐31G(d,p)/SMD(CH_2_Cl_2_).

In comparison, the well‐reported S_N_1’ substitution of α‐trifluoromethylstyene (**4**) [61, 62, 63], that proceeds via [CF_2_C(Ph)CH_2_]^+^ (**Int_4_**), is both kinetically and thermodynamically favored. The calculated structure of **Int_4_** possesses a γ‐position (C_B_) with a notably larger electrophilic Fukui index (*ƒ* ^+^) than the α‐position (C_A_), and the γ‐product [CF_2_C(Ph)CH_2_P(*o*‐Tol)_3_]^+^ is preferred over the α‐product [CF_2_P(*o*‐Tol)_3_C(Ph)CH_2_]^+^ by 8.3 kcal mol^−1^ (Figure S200). These data suggest that S_N_1’ pathways in propargyl fluorides are undeveloped as compared to allyl fluorides due to a kinetic preference for S_N_1 products in propargyl fluorides versus S_N_1’ products in allyl fluorides. This is congruent with a higher reorganizational energy required for sp to sp^2^ as compared to sp^2^ to sp^3^ rehybridization [84].

Having established a synthetic route to fluoroallenes from propargyl difluorides, we proceeded to explore the scope of the reaction (Figure 4). Reactions were performed under optimized conditions, in CH_2_Cl_2_ at ambient temperature for 2 h with stoichiometric Lewis base and either BF_3_·OEt_2_ (method A, see Supporting Information) or catalytic B(C_6_F_5_)_3_ (10 mol %) and Me_3_SiNTf_2_ (method B, see Supporting Information). Aryl substituents (including bromine, hydrogen, trifluoromethyl, phenyl, and methoxy groups) in the α‐ and γ‐positions allowed formation of phosphonium, pyridinium, and sulfonium fluoroallenic salts in good to excellent isolated yields ranging from 61%–98% (**3a‐PTol_3_**, **3a‐PMes_3_**, **3a‐TPPy**, **3a‐THT**, **3b‐PTol_3_**, **3c‐PTol_3_**, **3d‐PTol_3_**, **3f‐PTol_3_, 3f‐PTPPy**). Heteroaryl groups were also well tolerated with **3e‐PTol_3_**, featuring a 2‐thiophene group isolated in 83% yield.

**FIGURE 4:**
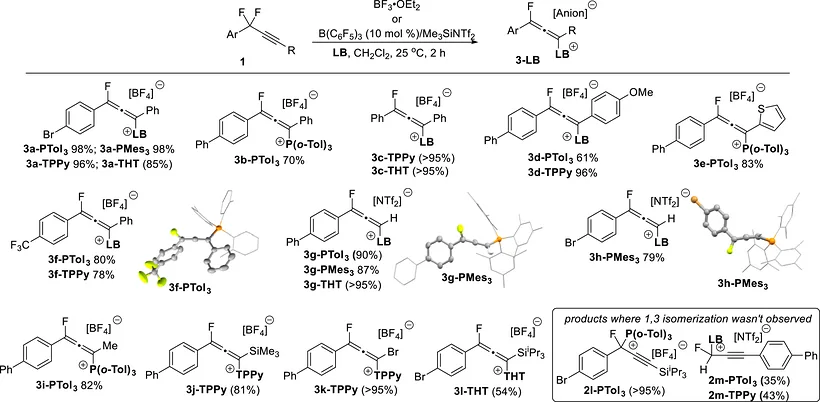
Reaction scope exploration for conversion of propargyl difluorides to fluoroallenes. Parentheses indicate ^19^F NMR yield; otherwise, isolated yields are given. See Supporting Information for reaction conditions. TPPy = 2,4,6‐triphenylpyridine, THT = tetrahydrothiophene.

Propargyl difluorides with terminal alkynyl groups led cleanly to the formation of fluoroallenes **3g‐PTol_3_**, **3g‐PMes_3_**, **3g‐THT**, **3h‐PTol_3_**, **3h‐THT**, and **3h‐PMes_3_**. However, the masked terminal products proved unstable with the exception of the PMes_3_ adducts **3g‐PMes_3_** and **3h‐PMes_3_**, where the allenic hydrogens were well protected, and products **3g‐PTol_3_**, **3g‐THT**, **3h‐PTol_3_**, and **3h‐THT** were thus characterized in situ. Substrates featuring alkyl, silyl, and bromo groups in the alkynyl γ‐position all provided fluoroallene products in good yields, namely, **3i‐PTol_3_** (82%), **3j‐TPPy** (78%), and **3k‐PTol_3_** (87%). Functional groups such as silyl, halo, and hydro groups provide powerful anchors for further orthogonal functionalization in addition to potential derivatization at the sites of the installed Lewis bases and allene backbone.

Activation of **1l**, which contains a sterically large triisopropylsilyl (TIPS) group in the γ‐position, led to Lewis base‐induced divergent reactivity. Utilizing THT gave the allenic product **3l‐THT** exclusively in 54% NMR yield, while use of P(*o*‐Tol)_3_ led to quantitative formation of **2l‐PTol_3_** (as adjudged by ^19^F NMR), which did not transform to **3l‐PTol_3_**. The divergence in products is seemingly a result of the larger steric profiles of P(*o*‐Tol)_3_ compared to THT, which renders **2l‐PTol_3_** as the thermodynamic product. This effect was also observed in the activation of difluoromethylalkynyl‐4‐biphenyl (**1m**) with a hydro‐substituted α‐position. The Lewis bases P(*o*‐Tol)_3_ and TPPy gave α‐substituted products, **2m‐PTol_3_** and **2m‐TPPy**. These compounds were found to be stable and did not undergo isomerization to the fluoroallenic onium salts. DFT calculations suggested that isomerization of **2m‐**

**PTol_3_** to the corresponding flouroallene **3m‐PTol_3_** was endergonic by 3.6 kcal mol^−1^ (Figure S205). As such, the formation of **2l‐PTol_3_**, **2m‐PTol_3_**, and **2m‐TPPy** represents examples where inversion of the thermodynamic product has occurred as opposed to the trapping of kinetic products, as is the case with **2a‐PPh_3_** and **2a‐PCy_3_**.

Single crystal x‐ray diffraction (SCXRD) studies of **3f‐PTol_3_**, **3g‐PMes_3_**, and **3h‐PMes_3_** provided structural confirmation that products **3‐LB** were formulated as fluoroallenic onium salts (Figure 4). However, all compounds **3‐LB** characterized by ^13^C NMR spectroscopy possessed characteristic allenic signals in the range of δ_C_ 190–225.

In contrast to previously synthesized fluoroallenes [70, 71, 72, 73, 74], compounds **3‐LB** are cationic onium salts, which have demonstrated divergent reactivity compared to neutral allenes [81, 82]. Compounds **3‐LB** have multiple positions amenable to further derivatization, including both the Lewis base and allenic positions. In principle, a diverse range of fluorinated architectures is quickly accessible from salts **3‐LB**, and given the novelty of these allenes, we sought to demonstrate some of the possible functionalization reactions (Figure 5).

**FIGURE 5:**
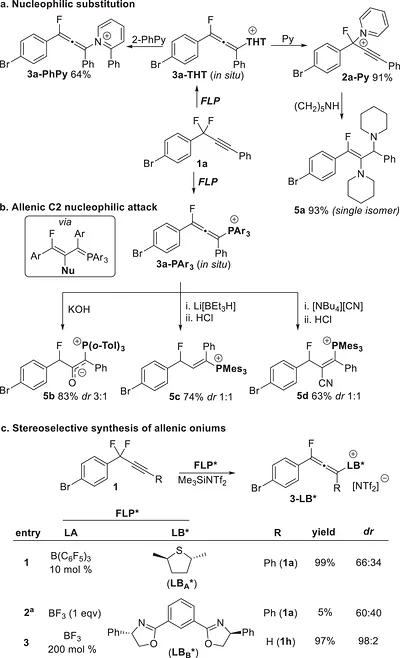
Demonstrated one‐pot functionalization of **1a**. (a) Substitution of the THT ligand in **3a‐THT** results in alkyne or allene products. (b) Attack of the allenic C2 position in **3a‐PAr_3_** (Ar = *p*‐Tol, Mes) gives rise to phosphine ylide products. Isolated yields are given from **1a** over two steps except **5a**, which was from isolated **2a‐Py**. (c) The use of chiral frustrated Lewis pairs (FLP*), consisting of chiral Lewis bases (LB*) and achiral Lewis acids (LA), allows for the stereoselective formation of products 3‐LB*. ^19^F NMR yields are given for products 3‐LB*. ^a^Me_3_SiNTf_2_ not added. See Supporting Information for reaction conditions.

We have previously shown that Lewis bases employed in FLP‐mediated C─F activation are excellent nucleofuges, allowing facile nucleophilic substitution [65, 66, 67]. Indeed, we were able to demonstrate similar ability through the nucleophilic displacement of THT in **3a‐THT** (Figure 5a). Treatment of in situ generated **3a‐THT** with 2‐phenyl pyridine leads to **3a‐PhPy** in 64% yield over two steps. However, use of the smaller nucleophile pyridine leads exclusively to the alkyne **2a‐Py** in 91% yield over two steps. Notably, pyridine could not be used directly in the FLP activation reactions of compounds **1** (as described above) due to Lewis adduct formation with BF_3_. Compound **2a‐Py** is susceptible to S_N_2’ reactivity, as demonstrated in reaction with piperidine that initially forms a neutral piperidinyl substituted allene that then quickly adds another equivalent of piperidine across the allene motif (i.e., HX addition) to give **5a** in 93% yield from isolated **2a‐Py**.

Phosphonium allenes were prone to ylide formation in preference to nucleophilic substitution (Figure 5b). When in situ generated **3a‐PTol_3_** was treated with KOH in THF, hydroxide attack occurred at the allenic C2 position, before tautomerization led to the formation of **5b** in 83% yield. Compound **5b** was observed as a 3:1 mixture of two isomers; however, coupling constants suggested exclusive *E* selectivity [85], and it is probable that the diastereomers arise from the chiral fluoro position and hindered rotation of phosphine aryl groups. Although **5b** can be formulated as a β‐keto ylide, the carbonyl ^13^C NMR resonance was observed at 182.0 ppm, suggesting a significant contribution of the *E‐*enolate canonical form [86]. Reaction of in situ generated **3a‐PMes_3_** with borohydride nucleophiles (viz. [NBu_4_][BH_4_] or Li[BHEt_3_]} led the formation of a fluorovinyl phosphonium ylide that was protonated with HCl to give **5c** in 74% yield (from **1a**) with exclusive *E* selectivity and a *dr* of 1:1. Finally, treatment of in situ generated **3a‐PMes_3_** with [NBu_4_]CN followed by HCl led to formation of **5d** in 63% yield (*dr* 1:1) via a fluoro‐cyano‐vinyl phosphonium ylide intermediate. Similar to **5b**, two isomers were observed for **5c** and **5d** with exclusive *E* selectivity.

Lastly, it was demonstrated that fluoroallenic salts could be generated in high stereoselectivity utilizing chiral frustrated Lewis pairs (FLP*), consisting of chiral Lewis bases (LB*) and achiral Lewis acids (Figure 5c). When an FLP* containing B(C_6_F_5_)_3_ and enantiopure (*R*,*R*)−2,5‐dimethylthiolane (LB_A_*) was applied to substrate **1a** in a similar manner as previously reported by us [66], the observed diastereoselectivity for product 3a‐LB_A_* was only 66:34, albeit with quantitative yield. Attempts to utilize enantiopure (4*S*,4′*S*)‐2,2′‐(1,3‐phenylene)bis[4,5‐dihydro‐4‐phenyloxazole] (LB_B_*) with BF_3_ in the stereoselective formation of 3a‐LB_B_* led to trace yields (<5%). However, applying this FLP* with superstoichiometric BF_3_ to **1h** provided 3h‐LB_B_* in 97% NMR yield with a *dr* of 98:2 (see Table S1 for details). This level of stereoselectivity compares well with reports of metal‐catalyzed desymmetrization of propargyl difluorides [72, 74]. The formulation of epimers 3h‐LB_B_* was confirmed via ^19^F─^13^C correlation spectroscopy, where an HMBC experiment aligned ^19^F NMR signals at δ_F_ −131.6 (major) and −132.0 (minor) with allenic C2^13^C NMR signals at 196.9 ppm (Figure S134).

## 3 Conclusion

In summary, we have developed a simple and practical method for generating fluoroallenyl onium salts from propargyl difluorides. This demonstrates a unique extension of S_N_1’ selective defluorination chemistry to difluorobenzylalkynes and is only possible using FLP systems that promote the formation of thermodynamic products over kinetic products. The title reaction can be made stereoselective through the use of chiral frustrated Lewis pairs, which can achieve diastereomeric excesses (*de*) of up to 96%. The resulting fluoroallenyl salts can serve as synthetic building blocks to access a wide range of fluorinated compounds quickly.

## Funding

This work was supported by the Australian Research Council (DP250102847).

## Conflicts of Interest

The authors declare no conflicts of interest.

## Supporting information



The authors have cited additional references within the Supporting Information [87, 88, 89, 90, 91, 92, 93, 94, 95, 96, 97, 98, 99, 100, 101, 102, 103, 104].

## Data Availability

The data that support the findings of this study are available in the Supporting Information of this article.
