# Supplementary material for: FLP Mediated Conversion of Difluorobenzylalkynes to Fluoroallenyl Oniums via an SN1’ Pathway
Source: Chemistry. 2026 Jun 2;32(41):e71213. doi: 10.1002/chem.71213 (PMC13729919; doi:10.1002/chem.71213)
Supplement: Supplementary file 1 [file CHEM-32-e71213-s001.pdf]

Electronic Supporting Information

FLP mediated conversion of difluorobenzylalkynes to fluoro-allenyl oniums via an SN1' pathway

Max Coles,[a] Tianhong Wang[a], Rubi McLennan,[a] Long Kit Kwan,[a] Bivas Mondal,[b] Richard Chan,[b] Rowan D. Young[a]*

[a] *School of Chemistry and Molecular Biosciences, University of Queensland, QLD, Australia 4069*

[b] *Department of Chemistry, National University of Singapore, 3 Science Drive 3, Singapore 117543*

**Corresponding Author*

Table of Contents

**General Experimental**	**2**
**Synthesis of Substrates 1a-m**	**3**
**Synthesis of Allene Adducts**	**11**
**Synthesis of Phosphonium Salts**	**12**
**Synthesis of Pyridinium Salts**	**22**
**Synthesis of Sulfonium Salts**	**26**
**Nucleophilic Substitution of Sulfonium Salts**	**28**
**Allenic C2 nucleophilic attack of phosphonium salts**	**30**
**Stereoselective synthesis of allenic onium salts**	**34**
**Kinetic Experiments**	**36**
**NMR Spectra**	**40**
**Mass Spectrometry Data**	**122**
**Computational details**	**153**
**References**	**185**

General Experimental

All experiments were performed under inert atmosphere using standard Schlenk techniques or an argon filled glove box (Vigor Technologies USA). All glassware was dried at 125 °C prior to use in reactions.

Dichloromethane, *n*-hexane, and toluene were purified using a LC Technology Solution Inc. SP-1 Solvent Purification System, solvents taken from this system were further deoxygenated and stored over 3 Å molecular sieves. Chloroform-*d*, dichloromethane-d_2, 1,2-dichloroethane, dimethylformamide, pyridine and dimethyl sulfoxide were stirred over calcium hydride overnight under argon prior to reduced pressure distillation, solvents were deoxygenated after distillation and stored over 3 Å molecular sieves. Tetrahydrofuran and diethyl ether were distilled over sodium and benzophenone and then stored over 3 Å molecular sieves. Triethylamine was stirred over a mixture of calcium hydride and Drierite™ overnight prior to distillation and was deoxygenated after. Column chromatography was performed using silica gel (230-400 mesh) with analytical grade solvents.

NMR spectra (^{1}H, ^{13}C, ^{19}F, ^{31}P) were collected on a Bruker Avance III HD 400 MHz spectrometer with a 5 mm BBFO probe at 298 K, Bruker Avance III HD 500 MHz spectrometer with a 5 mm DUL probe at 298 K, Bruker Avance III HD 500 MHz spectrometer with a 5 mm BBFO probe at 298 K, Bruker Avance III HD 500 MHz spectrometer with a 5 mm SEI probe at 298 K, Bruker Avance III HD 600 MHz spectrometer with a 5 mm TCI cryoprobe at 298 K, Bruker Avance III HD 700 MHz spectrometer with a 5 mm TCI cryoprobe at 298 K and a Bruker Avance NEO 900 MHz spectrometer with a 5 mm TCI cryoprobe at 298 K. Data were processed using MestReNova or Bruker Topspin software packages. The chemical shifts (δ) for ^{1}H and ^{13}C NMR spectra are given in ppm relative to solvent signals, and ^{31}P and ^{19}F NMR spectra were referenced to external 85% H_3PO_4 and $CFCl_3$ standards, respectively.

HR-MS(ESI) and HR-MS(APCI) data were acquired using an Orbitrap Elite Mass Spectrometer in positive modes. Elemental analysis was performed on a Thermo Scientific Flash 2000 Organic Elemental Analyser at the Microanalytical unit of the School of Chemistry and Molecular Biosciences at UQ.

X-ray data were collected on a Bruker D8 Venture dual source diffractometer at 150 K at the National University of Singapore on a Rigaku XtaLAB Synergy, Dualflex, Pilatus 300 K

diffractometer at 150 K at the University of Queensland. Structures were solved by intrinsic phasing methods using ShelXT[1] and refined with ShelXL[2] within the Olex2-1.5 software package.[3]

α,α-difluorocarboxylic acid precursors were synthesised following the method of Gouverneur.[4] Using these compounds, substrates were synthesised by the method of Wu[5] using EBX reagents synthesised using the methods of Waser.[6-7] Tris(pentafluorophenyl)borane was prepared using the method of Park and Massey.[8] All other materials were purchased from commercial suppliers and used as delivered.

Synthesis of Substrates 1a-m

1a - 1-bromo-4-(1,1-difluoro-3-phenylprop-2-yn-1-yl)benzene

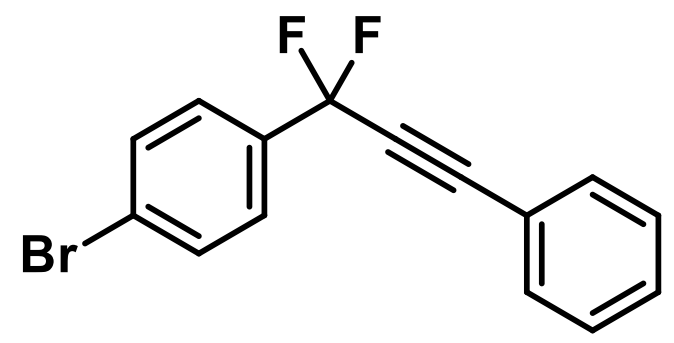

Compound **1a** was synthesised on a 2.98 mmol scale following literature procedures, the product was obtained in 71% yield (653 mg, 2.11 mmol). Characterisation data was in agreement with the literature.[5]

^{1}H NMR (500 MHz, $CDCl_3$) δ 7.61 (s, 4H), 7.56 – 7.51 (m, 2H), 7.46 – 7.41 (m, 1H), 7.40 – 7.35 (m, 2H). ^{13}C{^{1}H} NMR (125 MHz, $CDCl_3$) δ 135.5 (t, J = 28.8 Hz), 132.3 (t, J = 2.1 Hz), 132.0, 130.3, 128.7, 127.3 (t, J = 4.5 Hz), 125.4 (t, J = 2.2 Hz), 120.1 (t, J = 2.8 Hz), 112.4 (t, J = 231.8 Hz), 89.3 (t, J = 6.0 Hz), 81.5 (t, J = 41.7 Hz). ^{19}F NMR (470 MHz, $CDCl_3$) δ -75.2 (s, 2F). GC-MS m/z (ion, % relative intensity): 308 ($[M]^{\cdot+}$, 29), 306 ($[M]^{\cdot+}$, 29), 227 ($[M-Br]^{\cdot+}$, 100).

1b - 4-(1,1-difluoro-3-phenylprop-2-yn-1-yl)-1,1'-biphenyl

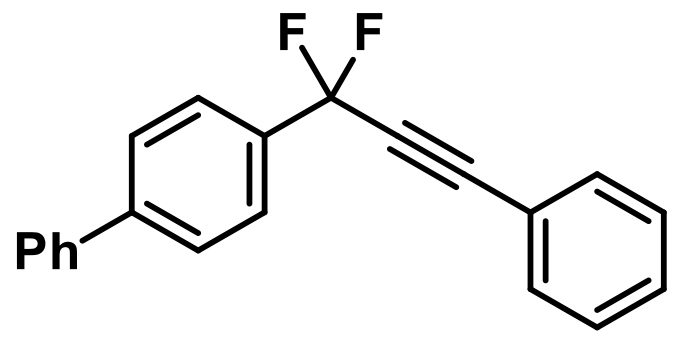

Compound **1b** was synthesised on a 0.534 mmol scale following literature procedures, the product was obtained in 77% yield (127 mg, 0.412 mmol). Characterisation data was in agreement with the literature.[5]

^{1}H NMR (500 MHz, $CDCl_3$) δ 7.98 – 7.86 (m, 2H), 7.79 (d, *J* = 8.5 Hz, 2H), 7.74 – 7.69 (m, 2H), 7.69 – 7.64 (m, 2H), 7.61 – 7.54 (m, 2H), 7.54 – 7.45 (m, 4H). $^{13}C\{^{1}H\}$ NMR (125 MHz, $CDCl_3$) δ 143.6, 140.0, 135.0 (t, *J* = 28.3 Hz), 132.4 – 131.7 (m), 129.9, 128.8, 128.4, 127.8, 127.2, 127.2, 125.8 (t, *J* = 4.5 Hz), 120.1 (t, *J* = 2.6 Hz), 112.7 (t, *J* = 230.8 Hz), 88.7 (t, *J* = 5.9 Hz), 81.8 (t, *J* = 41.9 Hz). ^{19}F NMR (470 MHz, $CDCl_3$) δ -74.6 (s, 2F). GC-MS m/z (ion, % relative intensity): 304 ($[M]^{\cdot+}$, 100), 283 ($[M\text{-}F]^{\cdot+}$, 32), 227 ($[M\text{-}C_6H_5]^{\cdot+}$, 28).

1c - (3,3-difluoroprop-1-yne-1,3-diyl)dibenzene

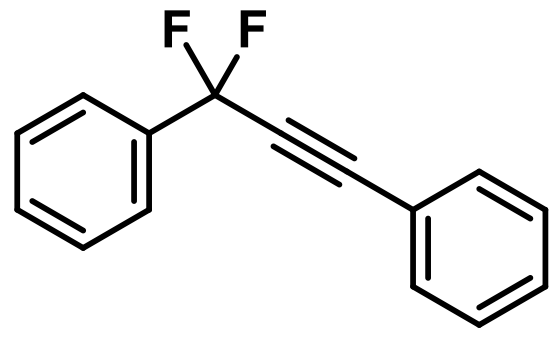

Compound **1c** was synthesised on a 1.45 mmol scale following literature procedures, the product was obtained in 28% yield (94 mg, 0.41 mmol). Characterisation data was in agreement with the literature.[5]

^{1}H NMR (500 MHz, $CDCl_3$) δ 7.81 – 7.70 (m, 2H), 7.60 – 7.53 (m, 2H), 7.51 – 7.44 (m, 3H), 7.45 – 7.40 (m, 1H), 7.40 – 7.33 (m, 3H). ^{13}C NMR (125 MHz, $CDCl_3$) δ 136.5 (t, *J* = 28.1 Hz), 132.3 (t, *J* = 2.4 Hz), 130.9 (d, *J* = 1.9 Hz), 130.2, 128.67 (d, *J* = 8.3 Hz), 125.6 (t, *J* = 4.6 Hz), 120.4 (t, *J* = 2.8 Hz), 112.9 (t, *J* = 231.3 Hz), 88.9 (t, *J* = 6.1 Hz), 82.0 (t, *J* = 41.8 Hz). ^{19}F NMR (470 MHz, CH_2Cl_2) δ -75.5 (s, 2F).

1d - 4-(1,1-difluoro-3-(4-methoxyphenyl)prop-2-yn-1-yl)-1,1'-biphenyl

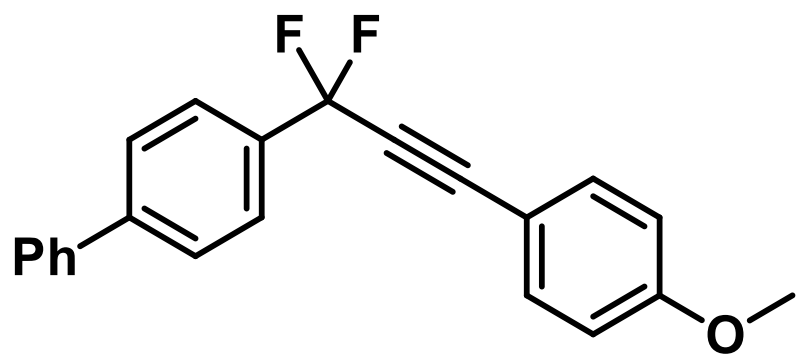

To a solution of 4-(1,1-difluoroprop-2-yn-1-yl)-1,1'-biphenyl (**1g**) (100.0 mg, 0.438 mmol), in triethylamine (2.0 mL) was added copper iodide (8.44 mg, 0.0440 mmol) and 4-iodoanisole (153.7 mg, 0.557 mmol). This solution was subjected to three freeze, pump, thaw cycles and then $Pd(PPh_3)Cl_2$ (15.54 mg, 0.022 mmol) was added. The reaction was stirred for 18 hours at room temperature and then the solvent removed *in vacuo*. The residue was redissolved in DCM (5 mL) and washed with water (3 × 5 mL) and brine (5 mL), the organic layer was then dried over anhydrous magnesium sulphate. The solvent was removed *in vacuo* and the product

purified *via* column chromatography on silica gel (1% ethyl acetate in hexanes). The title compound was isolated as a white solid. Yield 76.5 mg (52 %).

^{1}H NMR (500 MHz, $CDCl_3$) δ 7.87 – 7.79 (m, 2H), 7.74 – 7.67 (m, 2H), 7.65 – 7.60 (m, 2H), 7.55 – 7.45 (m, 4H), 7.43 – 7.37 (m, 1H), 6.96 – 6.84 (m, 2H), 3.84 (s, 3H). ^{13}C{^{1}H} NMR (125 MHz, $CDCl_3$) δ 161.1, 143.7 (d, *J* = 2.0 Hz), 140.3, 135.4 (t, *J* = 28.4 Hz), 134.0 (d, *J* = 2.2 Hz), 129.1, 128.0, 127.4 (d, *J* = 2.6 Hz), 126.1 (t, *J* = 4.4 Hz), 114.3, 113.1 (t, *J* = 230.7 Hz), 112.2 (t, *J* = 2.5 Hz), 89.3 (t, *J* = 6.0 Hz), 81.0 (t, *J* = 41.7 Hz), 55.5. ^{19}F NMR (470 MHz, $CDCl_3$) δ -73.9 (s, 2F). HRMS (APCI+, m/z) calc'd for $C_{22}H_{16}FO$ $[M-F]^+$: 315.1180, found 315.1183.

1e - 2-(3-([1,1'-biphenyl]-4-yl)-3,3-difluoroprop-1-yn-1-yl)thiophene

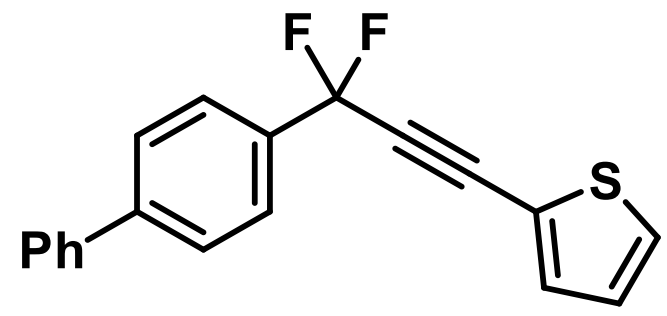

To a solution of 4-(1,1-difluoroprop-2-yn-1-yl)-1,1'-biphenyl (**1g**) (50.0 mg, 0.219 mmol), in triethylamine (2.0 mL) was added copper iodide (4.15 mg, 0.0219 mmol) and 2-iodothiophene (36.2 µL, 0.328 mmol). This solution was subjected to three freeze, pump, thaw cycles and then $Pd(PPh_3)Cl_2$ (7.68 mg, 0.011 mmol) was added. The reaction was stirred for 18 hours at room temperature and then the solvent removed *in vacuo*. The residue was redissolved in DCM (5 mL) and washed with water (3 × 5 mL) and brine (5 mL), the organic layer was then dried over anhydrous magnesium sulphate. The solvent was removed *in vacuo* and the product purified *via* column chromatography on silica gel (1% ethyl acetate in hexanes). The title compound was isolated as a pale yellow solid. Yield 34.7 mg (51 %).

^{1}H NMR (500 MHz, $CDCl_3$) δ 7.84 – 7.76 (m, 2H), 7.73 – 7.67 (m, 3H), 7.64 – 7.58 (m, 2H), 7.51 – 7.44 (m, 3H), 7.43 – 7.38 (m, 2H), 7.04 (dd, *J* = 5.1, 3.7 Hz, 1H). ^{13}C{^{1}H} NMR (125 MHz, $CDCl_3$) δ 143.9, 140.3, 137.0, 134.8 (t, *J* = 2.4 Hz), 131.6, 129.7, 129.1, 128.1, 127.5, 127.4, 126.1 (t, *J* = 4.4 Hz), 119.9, 112.9 (t, *J* = 231.6 Hz), 85.6 (t, *J* = 42.4 Hz), 82.6 (t, *J* = 6.0 Hz). ^{19}F NMR (470 MHz, $CDCl_3$) δ -75.0 (s, 2F). GC-MS m/z (ion, % relative intensity): 310 ($[M]^{\cdot+}$, 100), 291 ($[M-F]^{\cdot+}$, 13), 233 ($[M-C_6H_5]^{\cdot+}$, 32).

1f - 1-(1,1-difluoro-3-phenylprop-2-yn-1-yl)-4-(trifluoromethyl)benzene

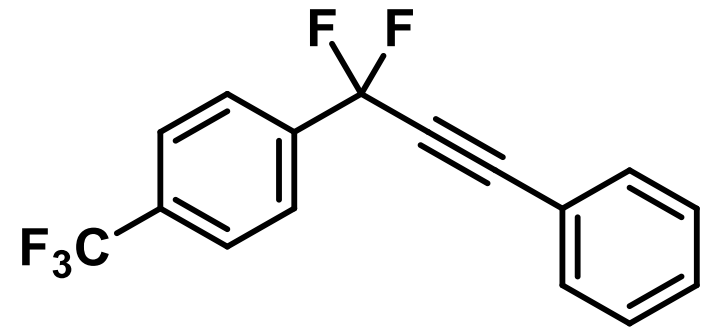

Compound **1f** was synthesised on a 2.10 mmol scale following adapted literature procedures, the product was obtained as a colourless oil in 26% yield (160 mg, 0.540 mmol).

^{1}H NMR (500 MHz, $CDCl_3$) δ 7.92 – 7.83 (m, 2H), 7.75 (d, *J* = 8.2 Hz, 2H), 7.58 – 7.52 (m, 2H), 7.47 – 7.41 (m, 1H), 7.41 – 7.35 (m, 2H). ^{13}C{^{1}H} NMR (125 MHz, $CDCl_3$) δ 133.0 (qt, *J* = 32.8, 1.6 Hz), 132.3 (t, *J* = 2.4 Hz), 130.4, 128.7, 126.1 (t, *J* = 4.5 Hz), 125.9 (q, *J* = 3.8 Hz), 123.8 (q, *J* = 272.5 Hz), 119.9 (t, *J* = 2.8 Hz), 112.0 (t, *J* = 232.5 Hz), 89.8 (t, *J* = 6.1 Hz), 81.3 (t, *J* = 41.5 Hz). ^{19}F NMR (470 MHz, $CDCl_3$) δ -63.0 (s, 3F), -76.3 (s, 2F). HRMS (APCI+, m/z) calc'd for $C_{16}H_9F_4$ [M-F]$^+$: 277.0635, found 277.0640.

1g-TIPS - (3-([1,1'-biphenyl]-4-yl)-3,3-difluoroprop-1-yn-1-yl)triisopropylsilane

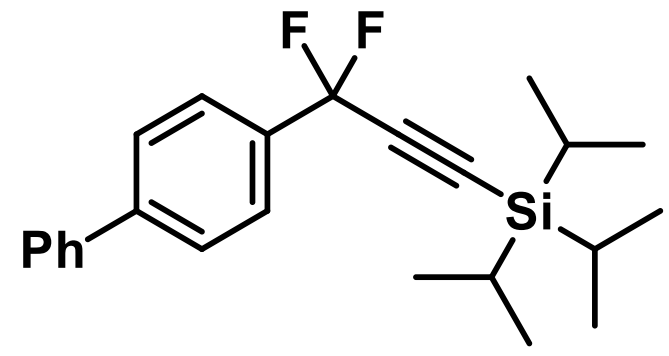

Compound **1e** was synthesised on a 4.028 mmol scale following literature procedures, the product was obtained in 90% yield (1.390 g, 3.128 mmol). Characterisation data was in agreement with the literature.[5]

^{1}H NMR (500 MHz, $CDCl_3$) δ 7.84 – 7.72 (m, 2H), 7.71 – 7.64 (m, 2H), 7.65 – 7.56 (m, 2H), 7.52 – 7.42 (m, 2H), 7.41 – 7.35 (m, 1H), 1.56 (s, 3H), 1.16 – 1.08 (m, 20H). ^{13}C{^{1}H} NMR (125 MHz, $CDCl_3$) δ 143.8, 140.3, 135.2 (t, *J* = 28.0 Hz), 129.0, 128.1, 127.4, 127.3, 126.1 (t, *J* = 4.3 Hz), 111.8 (t, *J* = 230.9 Hz), 99.0 (t, *J* = 40.0 Hz), 92.8 (t, *J* = 5.0 Hz), 18.7, 11.1. ^{19}F NMR (470 MHz, $CDCl_3$) δ -74.1 (s, 2F). GC-MS m/z (ion, % relative intensity): 384 ([M]$^{\cdot+}$, 30), 341 ([M-*i*Pr]$^{\cdot+}$, 88.67).

1g - 4-(1,1-difluoroprop-2-yn-1-yl)-1,1'-biphenyl

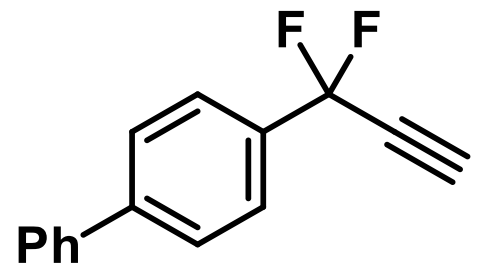

Compound **1g** was synthesised following a modified literature procedure. Characterisation was in agreement with the literature.[5]

A solution of (3-([1,1'-biphenyl]-4-yl)-3,3-difluoroprop-1-yn-1-yl)triisopropylsilane (500 mg, 1.30 mmol) was dissolved in tetrahydrofuran (10.0 ml) and cooled to -78 °C. To this was added tetrabutylammonium fluoride trihydrate (490.2 mg, 1.56 mmol) and the solution stirred for one hour at -78 °C. Upon warming to room temperature sodium bicarbonate solution was added (10% w/w, 10 mL) and the resulting solution extracted with dichloromethane (3 × 10 mL). The organics were combined, dried over anhydrous magnesium sulphate and the solvent removed *in vacuo*. Column chromatography on silica gel (1% ethyl acetate in hexanes) gave the title compound as a white solid. Yield 449 mg (68%).

^{1}H NMR (500 MHz, $CDCl_3$) δ 7.75 (dq, J = 7.7, 1.0 Hz, 2H), 7.67 (m, 2H), 7.61 – 7.57 (m, 2H), 7.49 – 7.44 (m, 2H), 7.42 – 7.36 (m, 1H), 3.01 (t, J = 5.0 Hz, 1H). ^{13}C{^{1}H} NMR (125 MHz, $CDCl_3$) δ 144.1 (d, J = 2.0 Hz), 140.2, 138.2, 136.8, 134.5 (t, J = 27.6 Hz), 129.1, 128.2, 127.5, 127.4, 126.0 (t, J = 4.7 Hz), 111.8 (t, J = 232.1 Hz).

1h - 1-bromo-4-(1,1-difluoroprop-2-yn-1-yl)benzene

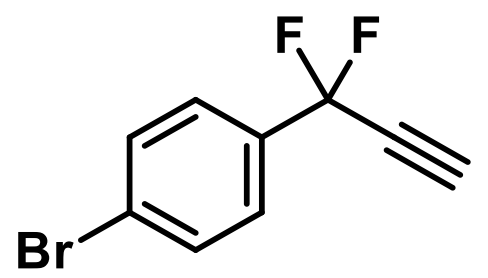

Compound **1h** was synthesised on a 1.29 mmol scale following the procedure of **1g** with compound **1l** as substrate. The product was found to be inseparable from the deprotected silane in our hands, as such only a crude yield of 52 % is reported. Characterisation confirmed the desired product was present despite the presence of this impurity.

^{1}H NMR (500 MHz, $CDCl_3$) δ 7.62 – 7.58 (m, 2H), 7.56 – 7.53 (m, 2H), 2.99 (t, J = 5.0 Hz, 1H). ^{13}C{^{1}H} NMR (125 MHz, $CDCl_3$) δ 134.7 (t, J = 28.1 Hz), 132.0, 127.2, 125.7 (t, J = 2.4 Hz), 111.3 (t, J = 232.5 Hz), 77.8 (t, J = 6.2 Hz), 76.4 (t, J = 41.9 Hz). ^{19}F NMR (470 MHz, $CDCl_3$) δ -77.2 (d, J = 4.8 Hz, 2F). HRMS (APCI+, m/z) calc'd for $^{79}BrC_9H_5F$ $[M-F]^+$: 210.9553, found 210.9548.

1i - 4-(1,1-difluorobut-2-yn-1-yl)-1,1'-biphenyl

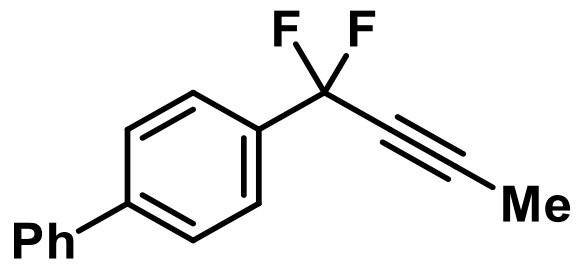

A solution of **1g** (50.0 mg, 0.219 mmol) in THF (5.0 mL) was cooled to -78 °C and *n*-BuLi (2.0 M in cyclohexane, 130 µL, 0.263 mmol) was added dropwise. The resulting solution was allowed to stir for 15 minutes and then methyl triflate (24.7 µL, 0.219 mmol) was added. The reaction was then allowed to stir and warm to room temperature over 18 hours. The reaction was quenched by the addition of saturated ammonium chloride solution (1.0 mL) and the resulting suspension diluted with water (4.0 mL). This was extracted with dichloromethane (3 × 10 mL), the organics were combined, dried over anhydrous magnesium sulphate and the solvent removed *in vacuo*. Column chromatography on silica gel (hexanes) gave the product as a yellow oil. Yield 37.3 mg (71%).

^{1}H NMR (500 MHz, $CDCl_3$) δ 7.76 – 7.70 (m, 2H), 7.69 – 7.63 (m, 2H), 7.62 – 7.58 (m, 2H), 7.49 – 7.44 (m, 2H), 7.41 – 7.36 (m, 1H), 2.01 (t, J = 5.1 Hz, 3H). ^{13}C{^{1}H} NMR (125 MHz, $CDCl_3$) δ 143.7, 140.4, 135.5 (t, J = 28.5 Hz), 129.1, 128.0, 127.4, 127.4, 126.0 (t, J = 4.5 Hz), 112.4 (t, J = 229.9 Hz), 87.1 (t, J = 5.8 Hz), 73.6 (t, J = 41.3 Hz), 3.8 (t, J = 2.4 Hz). ^{19}F NMR (470 MHz, $CDCl_3$) δ -73.9 (q, J = 5.1 Hz, 2F). HRMS (APCI+, m/z) calc'd for $C_{16}H_{12}F$ $[M-F]^+$: 223.0918, found 223.0923.

1j - (3-([1,1'-biphenyl]-4-yl)-3,3-difluoroprop-1-yn-1-yl)trimethylsilane

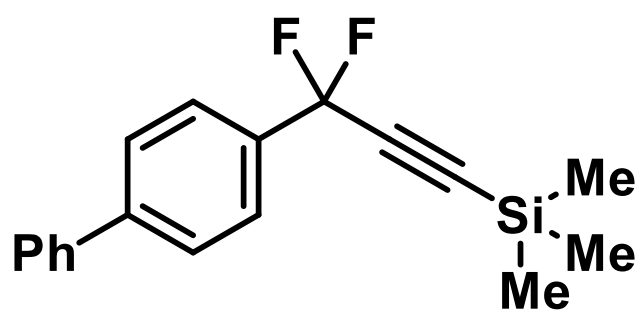

A solution of **1g** (50.0 mg, 0.219 mmol) in THF (5.0 mL) was cooled to -78 °C and *n*-BuLi (2.0 M in cyclohexane, 130 µL, 0.263 mmol) was added dropwise. The resulting solution was allowed to stir for 15 minutes and then a solution of trimethylsilyl chloride (28 mg, 0.262 mmol) in THF (1.0 mL) was added over the course of five minutes. The reaction was then allowed to stir and warm to room temperature over 18 hours. The reaction was quenched by the addition of saturated ammonium chloride solution (1.0 mL) and the resulting suspension diluted with water (4.0 mL). This was extracted with diethyl ether (3 × 10 mL), the organics were combined, dried over anhydrous magnesium sulphate and the solvent removed *in vacuo*.

Column chromatography on silica gel (hexanes) gave the title compound as a white crystalline solid. Yield 58.6 mg (88%).

^{1}H NMR (500 MHz, $CDCl_3$) δ 7.77 – 7.72 (m, 2H), 7.69 – 7.65 (m, 2H), 7.63 – 7.59 (m, 2H), 7.49 – 7.44 (m, 2H), 7.42 – 7.35 (m, 1H), 0.27 (s, 9H). ^{13}C{^{1}H} NMR (125 MHz, $CDCl_3$) δ 143.8, 140.3, 134.9 (t, *J* = 28.1 Hz), 129.1, 128.1, 127.4, 126.1 (t, *J* = 4.5 Hz), 111.7 (t, *J* = 230.8 Hz), 96.8 (t, *J* = 40.1 Hz), 95.6 (t, *J* = 4.9 Hz), -0.5. ^{19}F NMR (470 MHz, $CDCl_3$) δ -74.8 (s, 2F). HRMS (APCI+, m/z) calc'd for $C_{18}H_{18}FSi$ $[M-F]^+$: 281.1156, found 281.1162.

1k - 4-(3-bromo-1,1-difluoroprop-2-yn-1-yl)-1,1'-biphenyl

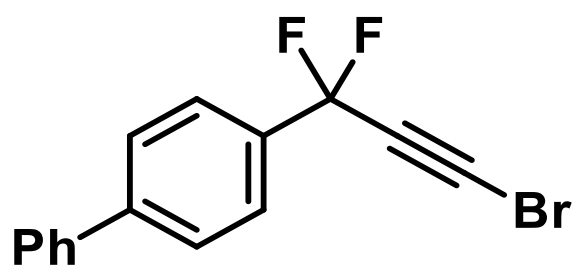

Compound **1k** was synthesised following the adapted method of Cloutier and Paquin.[9]

To a solution of 4-(1,1-difluoroprop-2-yn-1-yl)-1,1'-biphenyl (50.0 mg, 0.219 mmol), in acetone (5.0 mL), was added *N*-bromosuccinimide (46.0 mg, 0.263 mmol) and silver nitrate (1.86 mg, 0.0110 mmol). The resulting suspension was allowed to stir for 18 hours at room temperature. The reaction was quenched by the addition of saturated ammonium chloride solution (1 mL) and was then extracted with diethyl ether (3 × 10 mL). The organics were washed with brine (5 mL) and then dried over anhydrous magnesium sulphate. The solvent was removed *in vacuo* and the residue subjected to column chromatography on silica gel (hexanes) to yield the title compound as a white crystalline solid. Yield 65.2 mg (96 %).

^{1}H NMR (500 MHz, $CDCl_3$) δ 7.75 – 7.70 (m, 2H), 7.69 – 7.66 (m, 2H), 7.62 – 7.58 (m, 2H), 7.50 – 7.44 (m, 2H), 7.43 – 7.38 (m, 1H). ^{13}C{^{1}H} NMR (126 MHz, $CDCl_3$) δ 144.1 (d, *J* = 2.0 Hz), 140.2, 134.5 (t, *J* = 27.9 Hz), 129.1, 128.2, 127.5, 127.4, 125.9 (t, *J* = 4.6 Hz), 112.2 (t, *J* = 233.2 Hz), 74.0 (t, *J* = 43.9 Hz). ^{19}F NMR (470 MHz, $CDCl_3$) δ -75.4 (s, 2F). HRMS (APCI+, m/z) calc'd for $^{79}BrC_{15}H_9F$ $[M-F]^+$: 286.9866, found 286.9874.

1l - (3-(4-bromophenyl)-3,3-difluoroprop-1-yn-1-yl)triisopropylsilane

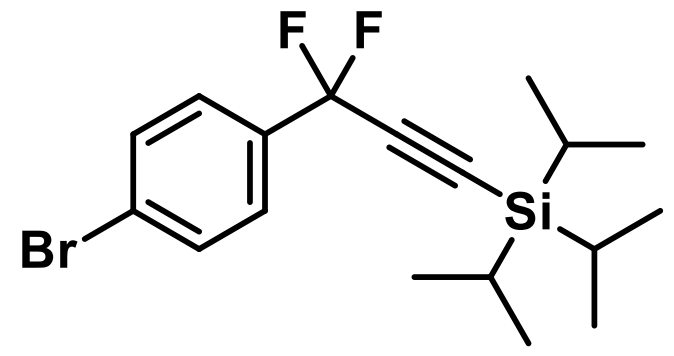

Compound **1l** was synthesised on a 2.98 mmol scale following literature procedures, the product was obtained as a colourless oil in 92% yield (1.07 g, 2.74 mmol). Characterisation data was in agreement with the literature.[5]

^{1}H NMR (400 MHz, $CDCl_3$) δ 7.63 – 7.49 (m, 4H), 1.30 – 1.22 (m, 3H), 1.18 – 1.02 (m, 18H). ^{13}C{^{1}H} NMR (125 MHz, $CDCl_3$) δ 139.6, 135.4 (t, J = 28.4 Hz), 131.9, 127.3 (t, J = 4.5 Hz), 111.2 (t, J = 231.6 Hz), 98.5 (t, J = 40.1 Hz), 93.3 (t, J = 4.9 Hz), 18.6, 11.1. ^{19}F NMR (470 MHz, $CDCl_3$) δ -75.0 (s, 2F). GC-MS m/z (ion, % relative intensity): 388 ($[M]^{\cdot+}$, 19), 386 ($[M]^{\cdot+}$, 18), 345 ($[M\text{-}i\text{Pr}]^{\cdot+}$, 72), 343 ($[M\text{-}i\text{Pr}]^{\cdot+}$, 71).

1m - 4-(3,3-difluoroprop-1-yn-1-yl)-1,1'-biphenyl

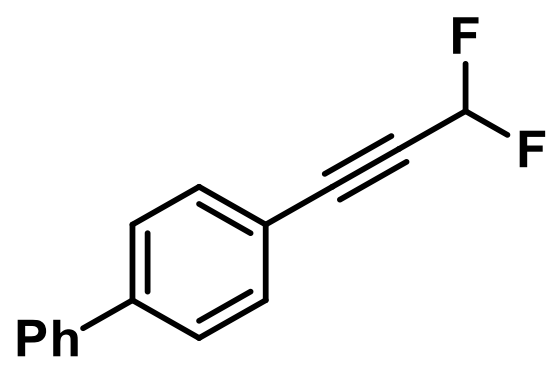

Compound **1m** was synthesised on a 1.00 mmol scale following literature procedures, the product was obtained as a white solid in 64 % yield (147 mg, 0.64 mmol). Characterisation data was in agreement with the literature.[10]

^{1}H NMR (500 MHz, $CDCl_3$) δ 7.67 – 7.54 (m, 7H), 7.50 – 7.43 (m, 2H), 7.42 – 7.35 (m, 1H), 6.44 (t, J = 55.1 Hz, 1H). ^{13}C{^{1}H} NMR (125 MHz, $CDCl_3$) δ 143.0, 139.9, 132.6 (t, J = 2.7 Hz), 129.0, 128.1, 127.2, 127.1, 118.6 (t, J = 3.2 Hz), 104.3 (t, J = 231.9 Hz), 88.4 (t, J = 7.3 Hz), 80.3 (t, J = 33.9 Hz). ^{19}F NMR (470 MHz, $CDCl_3$) δ -105.2 (d, J = 55.0 Hz).

Synthesis of Allene Adducts

General Procedure A – C-F Activation of Internal Alkyne Substrates using Stoichiometric Lewis Acid

Substrate (10.0 mg) was dissolved in dichloromethane (0.2 mL) and to this was added a dichloromethane solution (0.4 mL) containing $BF_3{\cdot}OEt_2$ (1.1 eqv) and the relevant Lewis base (1.1 eqv) dropwise. The resulting solution was stood at room temperature and analysed by ^{1}H NMR, ^{19}F NMR, ^{13}C NMR and ^{31}P NMR spectroscopy (where appropriate). $PhOCF_3$ was used as an internal standard to evaluate the progress of the reaction and calculate yields.

Allenes bearing electron withdrawing substituents or with phosphorous Lewis bases were found to be stable to isolation. In these cases, after completion of the reaction, the crude reaction mixtures were diluted with further dichloromethane (1.0 mL) and washed with sodium bicarbonate solution (1% w/w, 3 × 1.0 mL). The organics were dried over anhydrous sodium sulphate and the solvent removed *in vacuo*. The residue was redissolved in CD_2Cl_2 or $CDCl_3$ and either $PhOCF_3$ or $PPNBArF_{24}$ were added as an internal standard to calculate the yield. These compounds could be further purified by the layering of *n*-hexane onto dichloromethane solutions of the product; in some cases this gave the product as single crystals.

General Procedure B – C-F Activation of Alkyne Substrates Using $TMSNTf_2$ and catalytic Lewis Acid

Substrate (10.0 mg) was dissolved in dichloromethane (0.2 mL) and to this was added a dichloromethane solution (0.3 mL) containing Lewis acid (10 mol %) and the relevant Lewis base (1.1 eqv) dropwise. To this mixture was added a solution of *N*-(trimethylsilyl)bis(trifluoromethanesulfonyl)imide (1.1 eqv) in DCM (0.2 mL) dropwise. The resulting solution was stood at room temperature and analysed by ^{1}H NMR, ^{19}F NMR, ^{13}C NMR and ^{31}P NMR spectroscopy (where appropriate). $PhOCF_3$ was used as an internal standard to evaluate the progress of the reaction and calculate yields.

Allenes bearing electron withdrawing substituents or with phosphorous Lewis bases were found to be stable to isolation. In these cases, after completion of the reaction, the crude reaction mixtures were dried *in vacuo* and the resulting residue triturated from dichloromethane with *n*-hexane in triplicate. The residue was redissolved in CD_2Cl_2 or $CDCl_3$ and either $PhOCF_3$ or $PPNBArF_{24}$ were added as an internal standard to calculate the yield. These compounds

could be further purified by the layering of *n*-hexane onto dichloromethane solutions of the product; in some cases this gave the product as single crystals.

Synthesis of Phosphonium Salts

3a-PTol$_3$

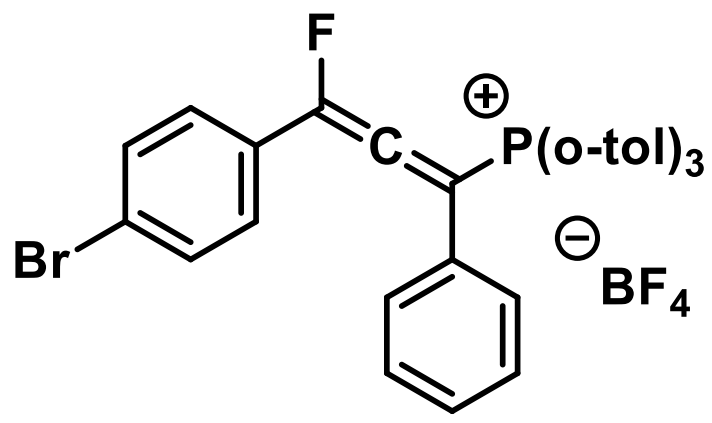

Compound **2a** was prepared from **1a** (10.0 mg, 32.6 μmol) following general procedure A, using P(*o*-tol)$_3$ as the Lewis base. The reaction reached completion after 18 hours and workup gave the isolated product as an off-white solid. This residue was redissolved in $CDCl_3$, PPNBArF$_{24}$ (0.5 eqv, 16.3 μmol, 22.8 mg) was added as an internal standard and gave the yield of the product as 92 % by ^{31}P NMR.

^{1}H NMR (700 MHz, $CDCl_3$) δ 7.85 – 7.69 (m, 3H), 7.62 – 7.52 (m, 5H), 7.50 – 7.44 (m, 2H), 7.43 – 7.38 (m, 1H), 7.32 – 7.27 (m, 2H), 7.25 – 7.22 (m, 2H), 7.09 – 6.95 (m, 2H), 2.05 (s, 9H). ^{13}C{^{1}H} NMR (175 MHz, $CDCl_3$) δ 212.5 (d, *J* = 31.9 Hz), 148.3 (d, *J* = 233.3 Hz), 144.2 (d, *J* = 8.2 Hz), 136.3, 135.2, 134.3 (d, *J* = 11.3 Hz), 132.8, 131.5, 129.9, 128.8 (d, *J* = 13.2 Hz), 128.6 (d, *J* = 4.4 Hz), 126.7, 126.1, 125.9 (dd, *J* = 30.7, 5.8 Hz), 115.9 (d, *J* = 89.6 Hz), 111.2 (d, *J* = 79.6 Hz), 23.1. ^{19}F{^{1}H} NMR (470 MHz, $CDCl_3$) δ -132.7 (bs), -144.6 (bs), -152.1 – -153.5 (m, 4F). ^{31}P{^{1}H} NMR (202 MHz, $CDCl_3$) δ 20.2 (d, *J* = 7.0 Hz, 1P). HRMS (ESI+, m/z) calc'd for $^{79}BrC_{36}H_{30}FP$ [M-BF$_4$]$^+$: 591.1247, found 591.1231. Elemental Analysis for $C_{36}H_{30}F_5BrPB$ Anal. Calc. (%): C 63.65, H 4.45. Found (%): C 63.49, H 4.99, average of two runs. – Co-elution of H and F

2a-PPh3 and 3a-PTol3

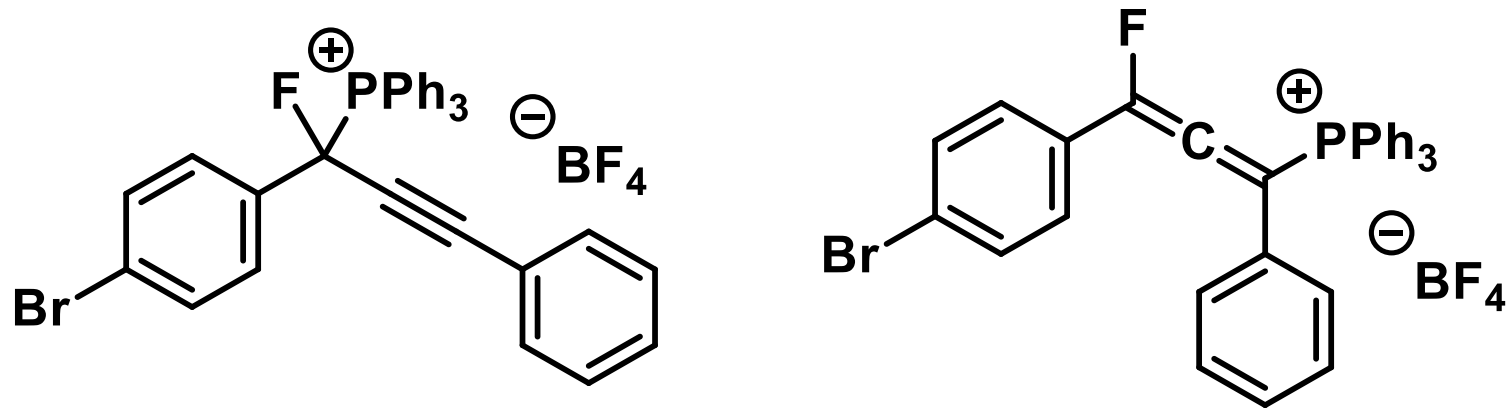

3:1

Compounds **2a-PPh3** and **3a-PPh3** were prepared from **1a** (10.0 mg, 32.6 μmol) following general procedure A, using PPh_3 as the Lewis base. The reaction reached completion after 18 hours, with the yield determined to be 96 % by comparison to the $PhOCF_3$ internal standard (1.0 eqv, 4.30 μL) in ^{19}F NMR. The alkyne and allene products were determined to be in a 3:1 ratio by ^{19}F NMR.

^{1}H NMR (500 MHz, CH_2Cl_2) δ 8.06 – 7.91 (m, 3H), 7.89 – 7.80 (m, 1H), 7.80 – 7.69 (m, 10H), 7.70 – 7.60 (m, 1H), 7.62 – 7.47 (m, 2H), 7.46 – 7.36 (m, 2H)†, 7.36 – 7.26 (m, 6H)†, 7.10 – 7.03 (m, 2H). ^{19}F{^{1}H} NMR (470 MHz, CH_2Cl_2) δ -139.1 (d, *J* = 102 Hz, 1F), -143.7 (s, 1F)*, -153.1 (s, 4F). ^{31}P{^{1}H} NMR (202 MHz, CH_2Cl_2) δ 30.1 (d, *J* = 102 Hz, 1P), 21.2 (s, 1P)*.

Resonance marked with a † are overlapped with (trifluoromethoxy)benzene. Allene resonance are marked with an *.

3a-PMes3

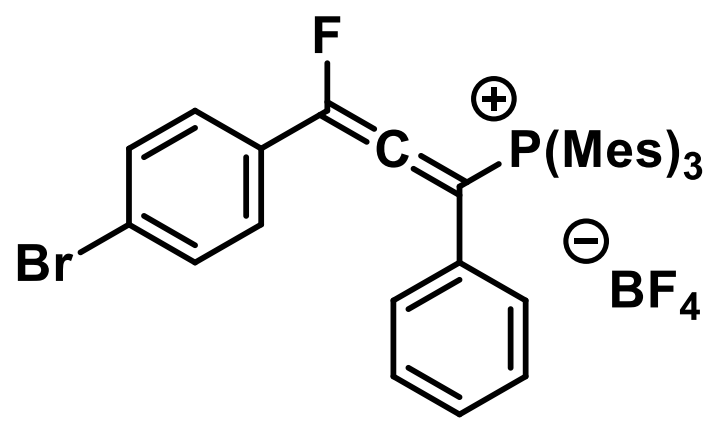

Compound **2ac** was prepared from **1a** (10.0 mg, 32.6 μmol) following general procedure A, using $P(Mes)_3$ as the Lewis base. The reaction reached completion after 4 hours, with the yield determined to be 98 % by mass balance in ^{31}P NMR. The product was isolated as a mixture of diastereomers resulting from the axial chirality of the allene and the orientation of the three aryl rings of the phosphine.

^{1}H NMR (900 MHz, CD_2Cl_2) δ 7.61 (d, *J* = 8.2 Hz, 2H), 7.52 (d, *J* = 8.2 Hz, 2H), 7.37 (t, *J* = 7.5 Hz, 1H), 7.29 (dt, *J* = 15.6, 7.6 Hz, 3H), 7.23 (d, *J* = 7.9 Hz, 2H), 7.20 (t, *J* = 7.8 Hz, 1H),

7.12 (d, J = 8.0 Hz, 2H), 7.04 (d, J = 5.2 Hz, 3H), 6.93 (d, J = 4.9 Hz, 2H), 6.85 (d, J = 4.9 Hz, 3H), 6.83 (d, J = 8.2 Hz, 2H), 2.30 (s, 18H), 2.28 (s, 9H), 2.21 (s, 9H), 2.19 (s, 9H), 2.17 (s, 9H). ^{13}C NMR (225 MHz, CD_2Cl_2) δ 212.0 (d, J = 30.0 Hz), 211.3 (d, J = 30.9 Hz), 148.7 (dd, J = 247.4, 15.7 Hz), 147.4 (dd, J = 247.7, 14.8 Hz), 145.9 – 145.8 (m), 145.7 (d, J = 3.1 Hz), 144.8, 144.6, 144.3 (d, J = 9.7 Hz), 144.2 (d, J = 10.0 Hz), 133.6 (d, J = 12.5 Hz), 133.6 (d, J = 12.3 Hz), 133.2, 133.2, 132.3, 131.0 (d, J = 11.1 Hz), 130.7 (d, J = 11.7 Hz), 130.5, 130.2, 129.1 (d, J = 22.7 Hz), 129.0 (d, J = 13.9 Hz), 128.6, 126.9, 126.8, 125.6, 125.4, 117.7 (d, J = 78.3 Hz), 117.4 (d, J = 77.7 Hz), 116.5 (dd, J = 71.8, 10.9 Hz), 115.5 (dd, J = 72.5, 8.1 Hz), 24.8, 24.8, 23.7, 23.7, 23.6, 23.6, 20.7, 20.7. ^{19}F NMR (565 MHz, CD_2Cl_2) δ -134.1 (d, J = 6.5 Hz, 1F), -148.3 (d, J = 6.5 Hz, 1F), -153.3 (m, 4F). ^{31}P NMR (202 MHz, CD_2Cl_2) δ 12.2 (d, J = 6.0 Hz, 1P), 11.2 (d, J = 6.0 Hz, 1P). HRMS (ESI+, m/z) calc'd for $^{79}BrC_{42}H_{42}FP$ [M-BF_4]$^+$: 675.2186, found 675.2185.

2a-PCy3 and 3a-PCy3

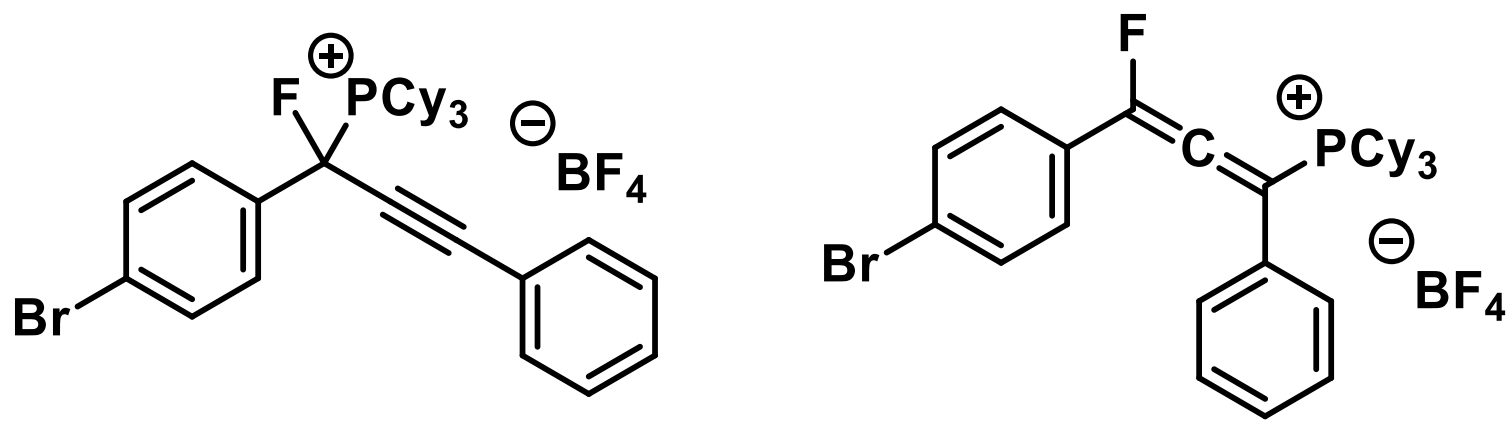

4:1

Compounds **2a-PCy3** and **3a-PCy3** were prepared from **1a** (10.0 mg, 32.6 μmol) following general procedure A, using PCy_3 as the Lewis base. The reaction was monitored after 18 hours, with the conversion determined to be 80 % by mass balance in the ^{31}P NMR. The alkyne and allene products were determined to be in a 4:1 ratio by ^{31}P NMR.

^{19}F NMR (470 MHz, CH_2Cl_2) δ -129.4 (d, J = 84.0 Hz), -145.4 (d, J = 4.7 Hz). ^{31}P NMR (202 MHz, CH_2Cl_2) δ 39.1 (d, J = 84.0 Hz), 32.1 (d, J = 4.7 Hz).

3b-PTol3

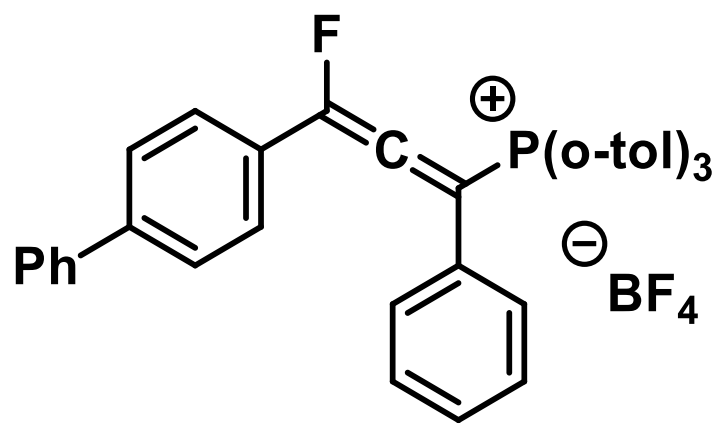

Compound **3b-PTol3** was prepared from **1b** (10.0 mg, 32.9 μmol) following general procedure A, using P(*o*-tol)3 as the Lewis base. The reaction reached completion after 24 hours workup gave the isolated product as an off-white solid. This residue was redissolved in $CDCl_3$, PPNBArF24 (0.25 eqv, 8.23 μmol, 11.5 mg) was added as an internal standard and gave the yield of the product as 70 % by ^{31}P NMR.

^{1}H NMR (500 MHz, $CDCl_3$) δ 7.88 – 7.68 (m, 8H), 7.64 (d, *J* = 8.1 Hz, 1H), 7.62 – 7.56 (m, 5H), 7.51 – 7.43 (m, 3H), 7.42 – 7.37 (m, 2H), 7.35 – 7.24 (m, 4H), 7.24 – 7.13 (m, 2H), 2.08 (s, 9H). ^{13}C{^{1}H} NMR (125 MHz, $CDCl_3$) δ 213.8 (d, *J* = 37.2 Hz), 144.4 (d, *J* = 1.8 Hz), 144.3 (d, *J* = 8.1 Hz), 139.4, 136.3, 135.1 (d, *J* = 12.5 Hz), 134.3 (d, *J* = 11.2 Hz), 131.4, 129.8, 129.2, 128.8 (d, *J* = 13.1 Hz), 128.7 – 128.5 (m), 128.0, 127.2, 125.7, 125.4 (d, *J* = 5.6 Hz), 115.9 (d, *J* = 112.5 Hz), 110.5 (d, *J* = 68.6 Hz), 23.1. ^{19}F{^{1}H} NMR (470 MHz, $CDCl_3$) δ -134.4 (bs), -146.1 (bs), -154.6 (m, 4F). ^{31}P{^{1}H} NMR (202 MHz, $CDCl_3$) δ 20.0 (d, *J* = 7.1 Hz, 1P). HRMS (ESI+, m/z) calc'd for $C_{42}H_{35}FP$ [M-BF4]$^{+}$: 589.2455, found 589.2440.

3d-PTol3

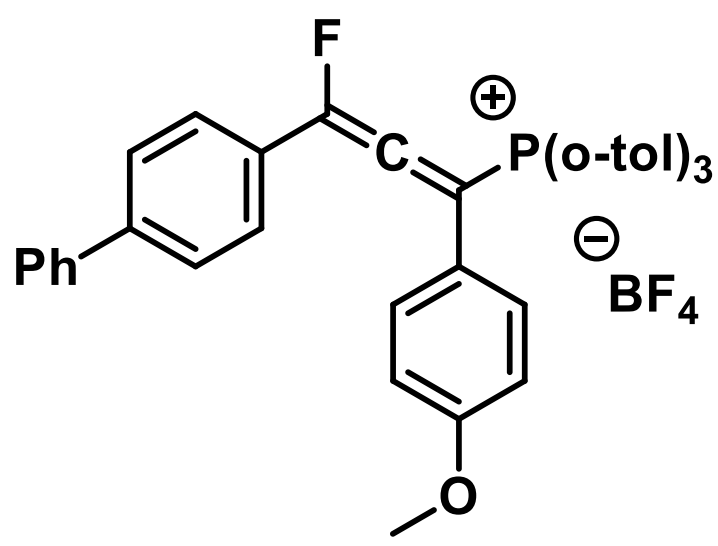

Compound **3d-PTol3** was prepared from **1d** (10.0 mg, 29.9 μmol) following general procedure A, using P(*o*-tol)3 as the Lewis base. The reaction reached completion after 24 hours, workup gave the isolated product as an off-white solid. This residue was redissolved in $CDCl_3$, PPNBArF24 (0.5 eqv, 15.0 μmol, 21.0 mg) was added as an internal standard and gave the yield of the product as 67 % by ^{31}P NMR.

^{1}H NMR (500 MHz, $CDCl_3$) δ 7.75 (s, 5H), 7.66 – 7.54 (m, 8H), 7.51 – 7.43 (m, 4H), 7.41 – 7.35 (m, 1H), 7.20 (d, *J* = 8.5 Hz, 5H), 6.89 – 6.69 (m, 2H), 3.79 (s, 3H), 2.07 (s, 9H). $^{13}C\{^{1}H\}$ NMR (125 MHz, $CDCl_3$) δ 211.2 (d, *J* = 31.7 Hz), 161.9, 144.3 (d, *J* = 8.2 Hz), 144.2 (d, *J* = 1.7 Hz), 139.5, 136.2, 135.1, 134.3 (d, *J* = 11.2 Hz), 130.2 (d, *J* = 4.7 Hz), 128.5, 128.2 (d, *J* = 250.3 Hz), 127.9, 125.7, 121.7 (d, *J* = 10.1 Hz), 115.8 – 114.9 (m), 110.1 (d, *J* = 75.1 Hz), 55.8, 23.0. $^{19}F\{^{1}H\}$ NMR (470 MHz, $CDCl_3$) δ -132.3 (bs), -144.5 (bs), -154.13 (m, 4F). $^{31}P\{^{1}H\}$ NMR (202 MHz, $CDCl_3$) δ 19.9 (d, *J* = 6.8 Hz, 1P). HRMS (ESI+, m/z) calc'd for $C_{43}H_{37}FOP$ $[M\text{-}BF_4]^+$: 619.2561, found 619.2532.

3e-PTol₃

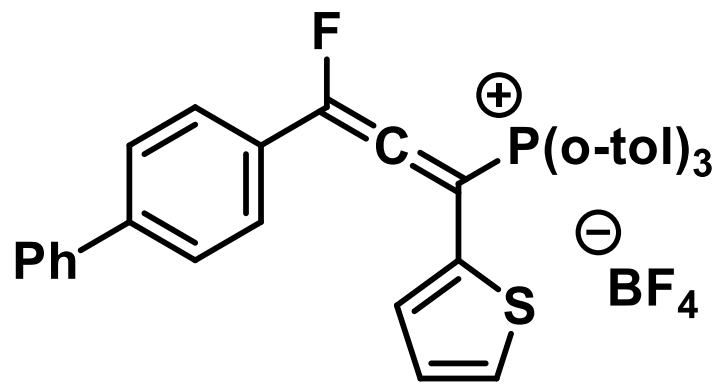

Compound **3e-PTol₃** was prepared from **1e** (10.0 mg, 32.2 μmol) following general procedure A, using P(*o*-tol)₃ as the Lewis base. The reaction reached completion after 24 hours, workup gave the isolated product as an off-white solid. This residue was redissolved in $CDCl_3$, $PPNBArF_{24}$ (0.5 eqv, 16.1 μmol, 22.5 mg) was added as an internal standard and gave the yield of the product as 83 % by ^{31}P NMR.

^{1}H NMR (500 MHz, CH_2Cl_2) δ 7.83 – 7.44 (m, 18H), 7.06 (td, *J* = 7.3, 1.9 Hz, 1H), 6.97 – 6.85 (m, 1H), 6.70 (dd, *J* = 7.8, 4.2 Hz, 1H), 6.62 (d, *J* = 3.9 Hz, 1H), 2.33 – 1.86 (m, 9H). $^{13}C\{^{1}H\}$ NMR (125 MHz, CH_2Cl_2) δ 149.7, 144.9, 144.7, 139.7 (d, *J* = 7.0 Hz), 136.6, 135.5, 134.5, 134.1 – 133.8 (m), 133.4 (d, *J* = 6.5 Hz), 131.4, 130.5 (d, *J* = 5.1 Hz), 129.5 (d, *J* = 106.0 Hz), 129.4 (d, *J* = 6.8 Hz), 128.8 (d, *J* = 7.4 Hz), 127.6 (d, *J* = 221.7 Hz), 115.8, 104.9. $^{19}F\{^{1}H\}$ NMR (470 MHz, CH_2Cl_2) δ -123.0 (bs), -139.8 (bs), -153.3 (m, 4F). $^{31}P\{^{1}H\}$ NMR (202 MHz, CH_2Cl_2) δ 19.5 (d, *J* = 7.1 Hz, 1P). HRMS (ESI+, m/z) calc'd for $C_{40}H_{33}FSP$ $[M\text{-}BF_4]^+$: 595.2019, found 595.1995.

3f-PTol₃

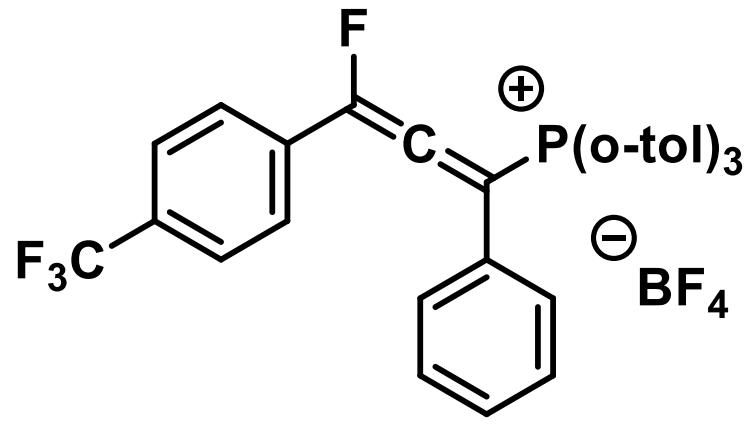

Compound **3f-PTol₃** was prepared from **1f** (10.0 mg, 33.7 µmol) following general procedure A, using P(*o*-tol)$_3$ as the Lewis base. The reaction reached completion after 72 hours, workup gave the isolated product as an off-white solid. This residue was redissolved in $CDCl_3$, PPNBArF$_{24}$ (0.5 eqv, 16.9 µmol, 23.6 mg) was added as an internal standard and gave the yield of the product as 88 % by ^{31}P NMR. Layering *n*-hexane onto a dichloromethane solution of the pure product gave diffraction quality crystals upon standing at 5 °C for 48 hours.

^{1}H NMR (500 MHz, CH_2Cl_2) δ 7.88 – 7.72 (m, 4H), 7.67 (d, J = 8.1 Hz, 1H), 7.61 – 7.51 (m, 3H), 7.52 – 7.40 (m, 8H), 7.36 – 7.22 (m, 5H), 2.08 (s, 9H). ^{13}C{^{1}H} NMR (125 MHz, CH_2Cl_2) δ 212.1 (d, J = 31.9 Hz), 147.9 (d, J = 260.0 Hz), 144.6 (d, J = 8.4 Hz), 136.5 (d, J = 3.2 Hz), 135.4 (d, J = 12.9 Hz), 134.5 (d, J = 11.6 Hz), 131.9, 130.1, 129.8 (d, J = 8.9 Hz), 129.0 (d, J = 4.7 Hz), 126.6 (d, J = 4.2 Hz), 125.9, 123.9 (q, J = 272.8 Hz), 116.0 (d, J = 89.6 Hz), 112.0 (d, J = 69.2 Hz), 23.2. ^{19}F{^{1}H} NMR (470 MHz, CH_2Cl_2) δ -64.2 (s, 3F), -135.7 (bs), -147.2 (bs), -153.9 (m, 4F). ^{31}P{^{1}H} NMR (202 MHz, CH_2Cl_2) δ 20.7 (d, J = 5.0, 1P). HRMS (ESI+, m/z) calc'd for $C_{37}H_{30}F_4P$ [M-BF$_4$]$^+$: 581.2016, found 581.2005. Elemental Analysis for $C_{37}H_{30}F_8PB{\cdot}0.5\ H_2O$ Anal. Calc. (%): C 65.60, H 4.61. Found (%): C 65.70, H 5.19, average of two runs. – Co-elution of H and F. Crystal Data for $C_{38}H_{32}BCl_2F_8P$ (M =753.31 g/mol): orthorhombic, space group Aea2 (no. 41), a = 17.6269(12) Å, b = 21.1112(14) Å, c = 18.8270(13) Å, V = 7006.0(8) Å^3, Z = 8, T = 150.00 K, μ(CuKα) = 2.720 mm^{-1}, $Dcalc$ = 1.428 g/cm^3, 37239 reflections measured (8.046° ≤ 2Θ ≤ 136.822°), 6344 unique (R_{int} = 0.0563, R_{sigma} = 0.0434) which were used in all calculations. The final R_1 was 0.0506 (I > 2σ(I)) and wR_2 was 0.1456 (all data).

3g-PMes3

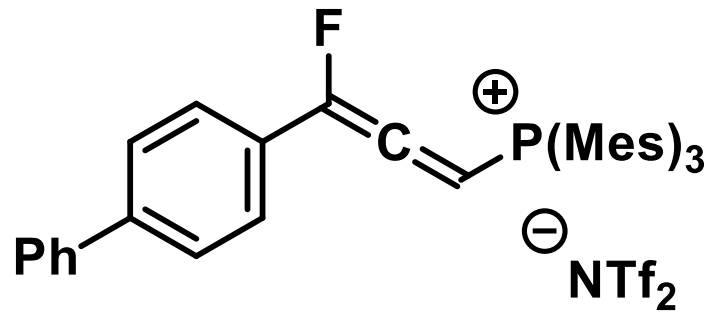

Compound **3g-PMes3** was prepared from **1g** (10.0 mg, 43.8 μmol) following general procedure B, using tris(pentafluorophenyl)borane as the Lewis acid and $P(Mes)_3$ as the Lewis base. The reaction reached completion after 2 hours, workup gave the isolated product as a yellow solid. This residue was redissolved in CD_2Cl_2, $PPNBArF_{24}$ (0.25 eqv) was added as an internal standard and gave the yield of the product as 87 % by ^{31}P NMR. Layering *n*-hexane onto a dichloromethane solution of the pure product gave diffraction quality crystals upon standing at 5 °C for 48 hours.

^{1}H NMR (600 MHz, CD_2Cl_2) δ 7.72 – 7.38 (m, 8H), 7.38 – 7.20 (m, 2H), 7.21 – 7.05 (m, 6H), 7.04 – 6.94 (m, 1H), 2.44 – 2.18 (m, 20H), 2.08 (s, 9H). ^{13}C{^{1}H} NMR (150 MHz, CD_2Cl_2) δ 212.6 – 210.3 (m), 147.9 (dd, *J* = 331.8, 2.3 Hz), 145.9 (d, *J* = 3.1 Hz), 143.8 (dd, *J* = 167.9, 10.0 Hz), 143.4, 139.2, 133.0 (dd, *J* = 46.8, 12.0 Hz), 129.9, 129.6, 128.8, 128.0, 127.3 (d, *J* = 20.3 Hz), 127.0, 126.7, 126.0 (dd, *J* = 32.6, 5.2 Hz), 125.5 (t, *J* = 3.2 Hz), 119.7 (q, *J* = 321.7 Hz), 117.6 – 115.1 (m), 101.1 (t, *J* = 95.0 Hz), 24.8, 23.6 (d, *J* = 6.0 Hz), 20.7. ^{19}F NMR (565 MHz, CD_2Cl_2) δ -79.5 (s, 6F), -140.1 (s), -150.4 (s). ^{31}P{^{1}H} NMR (202 MHz, CD_2Cl_2) δ 3.9 (s), 3.6 (s). HRMS (ESI+, m/z) calc'd for $C_{42}H_{43}FP$ $[M\text{-}NTf_2]^+$: 597.3081, found 597.3053. Crystal Data for $C_{44}H_{43}F_7NO_4PS_2$ (*M* =877.88 g/mol): monoclinic, space group $P2_1/n$ (no. 14), *a* = 11.7568(4) Å, *b* = 23.6567(9) Å, *c* = 14.8933(6) Å, *β* = 99.4790(10)°, *V* = 4085.7(3) Å^3, *Z* = 4, *T* = 100.00 K, μ(MoKα) = 0.246 mm^{-1}, *Dcalc* = 1.427 g/cm^3, 65539 reflections measured (4.448° ≤ 2Θ ≤ 50.236°), 6893 unique (R_{int} = 0.0522, R_{sigma} = 0.0291) which were used in all calculations. The final R_1 was 0.1225 (I > 2σ(I)) and wR_2 was 0.2996 (all data).

3g-PTol₃

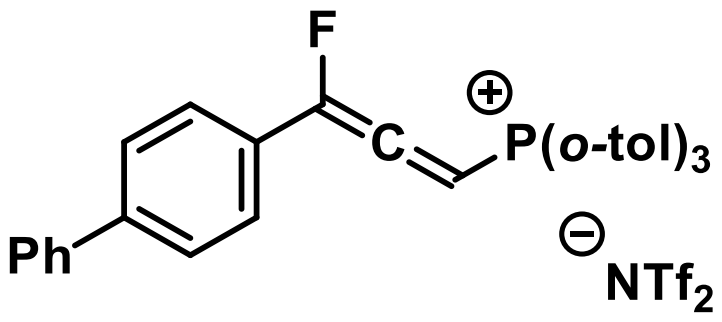

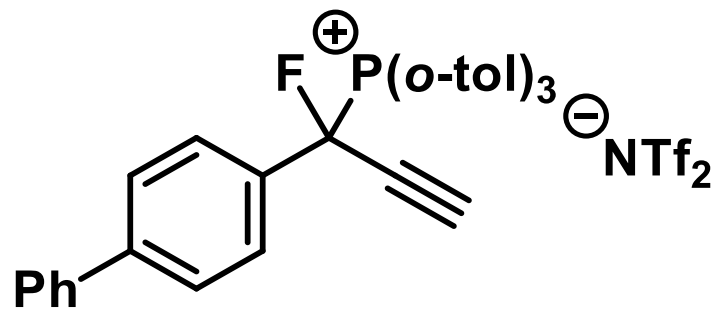

Compound **3g-PTol₃** was prepared from **1g** (10.0 mg, 43.8 μmol) following general procedure B, using tris(pentafluorophenyl)borane as the Lewis acid and P(*o*-tol)$_3$ as the Lewis base. The reaction reached completion after 30 minutes and comparison to the $PhOCF_3$ internal standard (1 eqv, 43.8 μmol) showed quantitative conversion to a 1:9 mixture of alkyne to allene product in ^{19}F NMR. This mixture of products was found to be unstable, decomposing when workup was attempted.

^{1}H NMR (600 MHz, CD_2Cl_2) δ 7.79 (tt, *J* = 7.5, 1.8 Hz, 4H), 7.69 – 7.65 (m, 2H), 7.65 – 7.59 (m, 3H), 7.59 – 7.51 (m, 10H), 7.50 – 7.45 (m, 3H), 7.42 – 7.39 (m, 1H), 7.28 – 7.25 (m, 3H), 2.26 (d, *J* = 1.4 Hz, 9H). ^{13}C{^{1}H} NMR (150 MHz, CD_2Cl_2) δ 217.9 (dd, *J* = 36.9, 4.5 Hz), 149.4 (dd, *J* = 246.7, 17.2 Hz), 145.6 (d, *J* = 9.3 Hz), 144.4, 144.4 (d, *J* = 8.6 Hz), 143.3 (d, *J* = 26.3 Hz), 139.9, 137.2 (dd, *J* = 9.8, 2.7 Hz), 136.6 (d, *J* = 3.0 Hz), 135.2 (dd, *J* = 51.5, 11.5 Hz), 135.1 (dd, *J* = 152.5, 12.0 Hz), 133.5, 130.6 (d, *J* = 4.8 Hz), 129.6, 129.3, 128.9, 128.7 (d, *J* = 13.5 Hz), 128.2, 127.5, 126.7, 126.1 (t, *J* = 3.1 Hz), 125.8 (dd, *J* = 29.0, 6.3 Hz), 120.5 (q, *J* = 321.7 Hz), 115.7 (d, *J* = 85.7 Hz), 95.9 (dd, *J* = 84.4, 8.3 Hz), 23.5 (d, *J* = 4.4 Hz). ^{19}F NMR (565 MHz, CD_2Cl_2) δ -79.0 (s, 6F), -125.2 (d, *J* = 97.6 Hz), -147.1 (s). ^{31}P{^{1}H} NMR (202 MHz, CD_2Cl_2) δ 30.7 (d, *J* = 96.9 Hz), 15.3 (s).

3h-PMes₃

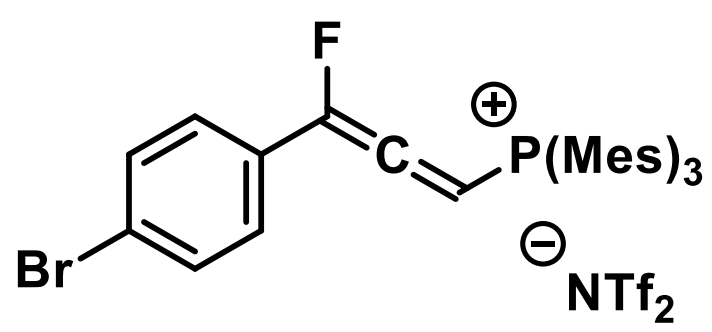

Compound **3h-PMes₃** was prepared from **1h** (10.0 mg, 42.4 μmol) following general procedure B, using tris(pentafluorophenyl)borane as the Lewis acid and P(Mes)$_3$ as the Lewis base. The reaction reached completion after 30 minutes, and comparison to the $PhOCF_3$ internal standard (1 eqv, 43.8 μmol) gave the yield as 79 %. Layering of *n*-hexane on to a dichloromethane solution of the product at -20 °C gave diffraction quality crystals after 48 hours.

^{1}H NMR (600 MHz, CD_2Cl_2) δ 7.73 – 7.37 (m, 2H), 7.39 – 6.97 (m, 8H), 6.79 (s, 1H), 2.38 (d, *J* = 18.5 Hz, 9H), 2.23 (d, *J* = 40.7 Hz, 9H), 2.06 (s, 9H). $^{13}C\{^{1}H\}$ NMR (150 MHz, CD_2Cl_2) δ 211.2 (d, *J* = 151.0 Hz), 147.4 (d, *J* = 3.0 Hz), 146.6 (d, *J* = 3.0 Hz), 145.0 (d, *J* = 9.3 Hz), 143.8 (d, *J* = 9.5 Hz), 133.8 (d, *J* = 11.9 Hz), 133.5 (d, *J* = 11.8 Hz), 132.7, 127.2 (t, *J* = 3.2 Hz), 126.9 (d, *J* = 34.8 Hz), 125.6, 120.3 (q, *J* = 321.7 Hz), 117.1 (d, *J* = 78.6 Hz), 111.9 (d, *J* = 82.9 Hz), 103.4 – 101.0 (m), 25.4, 24.2 (d, *J* = 5.3 Hz), 21.3. ^{19}F NMR (565 MHz, CD_2Cl_2) δ -79.5 (s, 6F) -140.1 (s), -150.5 (s). $^{31}P\{^{1}H\}$ NMR (202 MHz, CD_2Cl_2) δ 4.55 (s), 4.25 (s). HRMS (ESI+, m/z) calc'd for $^{79}BrC_{36}H_{38}FP$ $[M-NTf_2]^+$: 599.1873, found 599.1843. Crystal Data for $C_{38}H_{38}BrF_7NO_4PS_2$ (*M* =880.69 g/mol): monoclinic, space group Cc (no. 9), a = 8.3076(2) Å, b = 33.5003(8) Å, c = 14.1051(4) Å, β = 95.699(2)°, V = 3906.15(17) Å^3, Z = 4, T = 149.9(2) K, μ(Mo Kα) = 1.274 mm^{-1}, *Dcalc* = 1.498 g/cm^3, 41467 reflections measured (3.786° ≤ 2Θ ≤ 56.56°), 9586 unique (R_{int} = 0.0511, R_{sigma} = 0.0458) which were used in all calculations. The final R_1 was 0.0480 (I > 2σ(I)) and wR_2 was 0.1266 (all data).

3i-PTol₃

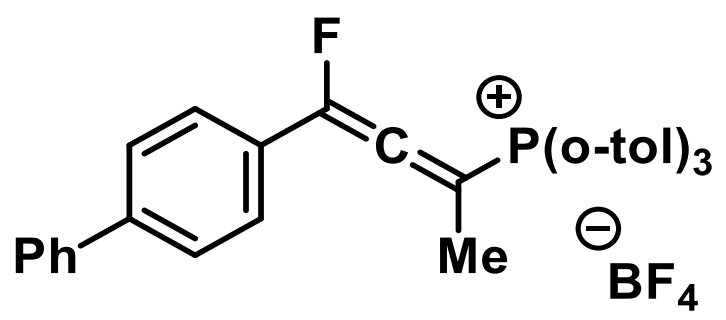

Compound **3i-PTol₃** was prepared from **1i** (10.0 mg, 41.3 μmol) following general procedure A, using P(*o*-tol)$_3$ as the Lewis base. The reaction reached completion after 18 hours, workup gave the isolated product as an off-white solid. This residue was redissolved in $CDCl_3$, $PPNBArF_{24}$ (0.5 eqv, 20.6 μmol, 28.9 mg) was added as an internal standard and gave the yield of the product as 83 % by ^{31}P NMR.

^{1}H NMR (500 MHz, CH_2Cl_2) δ 7.82 – 7.74 (m, 3H), 7.71 – 7.59 (m, 5H), 7.59 – 7.50 (m, 5H), 2.36 (dd, *J* = 13.0, 6.5 Hz, 3H), 2.13 (s, 9H). $^{13}C\{^{1}H\}$ NMR (125 MHz, CH_2Cl_2) δ 212.6 (d, *J* = 34.5 Hz), 149.6, 147.7 (d, *J* = 258.3 Hz), 144.5 – 144.2 (m), 144.2 – 144.0 (m), 139.8 (d, *J* = 6.5 Hz), 136.3 (d, *J* = 6.2 Hz), 135.2 (d, *J* = 10.9 Hz), 134.5 (t, *J* = 8.9 Hz), 129.4 (d, *J* = 6.6 Hz), 128.7 (t, *J* = 10.1 Hz), 128.0 (d, *J* = 6.4 Hz), 126.0, 115.6 (d, *J* = 83.8 Hz), 104.2 (d, *J* = 77.1 Hz), 23.0, 20.4. $^{19}F\{^{1}H\}$ NMR (470 MHz, CH_2Cl_2) δ -139.9 (bs), -148.1 (bs), -153.3 (m, 4F). $^{31}P\{^{1}H\}$ NMR (202 MHz, CH_2Cl_2) δ 21.8 (d, *J* = 6.6 Hz, 1P). HRMS (ESI+, m/z) calc'd for $C_{37}H_{33}FP$ $[M-BF_4]^+$: 527.2298, found 527.2274.

2l-PTol3

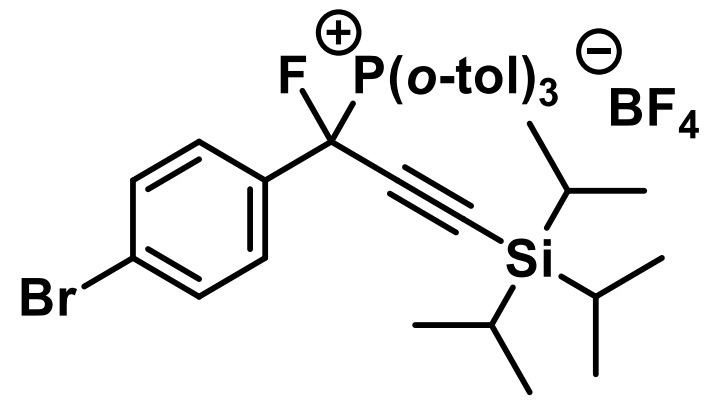

Compound **2l-PTol3** was prepared from **1l** (10.0 mg, 25.8 μmol) following general procedure A, using P(*o*-tol)3 as the Lewis base. The reaction reached completion after 18 hours, with the yield determined to be 97 % by comparison to the PhOCF3 internal standard (1.0 eqv, 3.5 μL) in ^{19}F NMR.

^{1}H NMR (500 MHz, CH_2Cl_2) δ 7.94 – 7.84 (m, 3H), 7.77 (tt, *J* = 7.7, 1.7 Hz, 3H), 7.52 – 7.44 (m, 8H), 7.12 (dt, *J* = 8.7, 1.7 Hz, 2H), 2.00 (s, 9H), 1.08 – 1.01 (m, 3H), 1.00 – 0.93 (m, 18H). ^{19}F{^{1}H} NMR (470 MHz, CH_2Cl_2) δ -121.4 (d, *J* = 98 Hz, 1F), -153.8 (s, 4F). ^{31}P{^{1}H} NMR (202 MHz, CH_2Cl_2) δ 29.9 (d, *J* = 98 Hz, 1P).

2m-PTol3

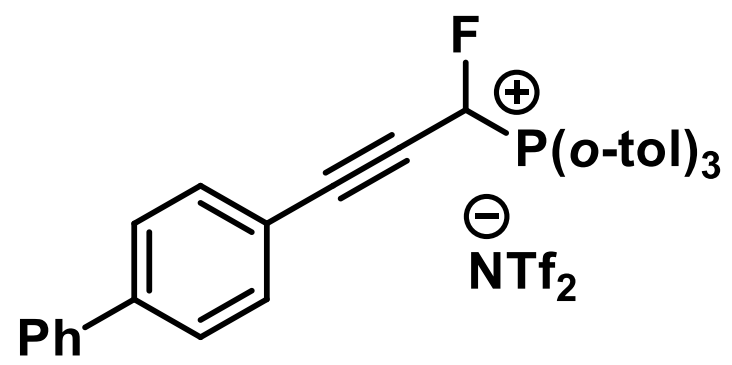

Compound **2m-PTol3** was prepared from **1m** (10.0 mg, 43.8 μmol) following general procedure B, using tris(pentafluorophenyl)borane as the Lewis acid and P(*o*-tol)3 as the Lewis base. The reaction reached completion after one hour forming a complex mixture of products with **2m-PTol3** formed in 35% as adjudged by ^{19}F NMR analysis.

^{19}F NMR (470 MHz, CH_2Cl_2) δ -174.75 (dd, *J* = 84.9, 47.3 Hz). ^{31}P{^{1}H} NMR (202 MHz, CH_2Cl_2) δ 34.51 (d, *J* = 80.7 Hz). HRMS (ESI+, m/z) calc'd for $C_{36}H_{31}FP$ [M-NTf2]$^{+}$: 513.2142, found 513.2132.

Synthesis of Pyridinium Salts

3a-TPPy

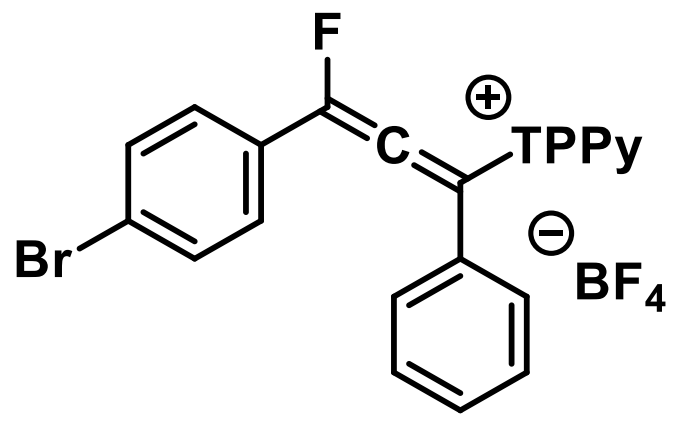

Compound **3a-TPPy** was prepared from **1a** (10.0 mg, 32.6 μmol) following general procedure A, using 2,4,6-triphenylpyridine as the Lewis base. The reaction reached completion after 18 hours, with the yield determined to be 98 % by comparison to the $PhOCF_3$ internal standard (1.0 eqv, 4.30 μL) in ^{19}F NMR. Standard work-up gave the product as an off-white solid.

^{1}H NMR (700 MHz, CD_2Cl_2) δ 8.25 (dd, J = 22.5, 2.2 Hz, 2H), 8.02 – 7.96 (m, 2H), 7.73 – 7.69 (m, 1H), 7.68 – 7.64 (m, 2H), 7.60 (tt, J = 7.3, 1.5 Hz, 1H), 7.55 – 7.52 (m, 2H), 7.52 – 7.48 (m, 1H), 7.47 – 7.34 (m, 12H), 7.07 (dd, J = 7.5, 1.7 Hz, 2H), 6.69 – 6.65 (m, 2H). ^{13}C{^{1}H} NMR (175 MHz, CD_2Cl_2) δ 194.1 (d, J = 27.5 Hz), 158.8, 156.8 (d, J = 27.6 Hz), 146.5 (d, J = 253.9 Hz), 138.6, 133.9, 132.8, 132.6, 132.1, 131.8 (d, J = 8.1 Hz), 131.6, 131.3, 130.5, 130.1 (d, J = 6.4 Hz), 129.8, 129.4, 129.0 (d, J = 14.1 Hz), 127.6 (d, J = 3.8 Hz), 127.3 (d, J = 65.4 Hz), 126.7, 126.1, 123.2 (d, J = 10.0 Hz). ^{19}F NMR (659 MHz, CD_2Cl_2) δ -127.3 (s, 1F), -152.2 – -153.6 (m, 4F). HRMS (ESI+, m/z) calc'd for $^{79}BrC_{38}H_{26}NF$ $[M-BF_4]^+$: 594.1227, found 594.1245. Elemental Analysis for $C_{38}H_{26}F_5NBrB\cdot H_2O$ Anal. Calc. (%): C 65.17, H 4.03, N 2.00. Found (%): C 64.88, H 3.80, N 2.31, average of two runs.

3c-TPPy

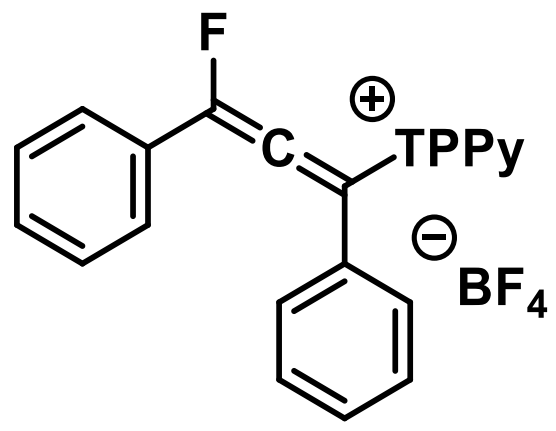

Compound **3c-TPPy** was prepared from **1c** (10.0 mg, 43.8 μmol) following general procedure A, using 2,4,6-triphenylpyridine as the Lewis base. The reaction reached completion after 2 hours, with the yield determined to be 99 % by comparison to the $PhOCF_3$ internal standard (1.0 eqv, 5.75 μL) in ^{19}F NMR.

^{1}H NMR (500 MHz, CH_2Cl_2) δ 8.28 – 8.20 (m, 2H), 8.01 – 7.96 (m, 2H), 7.71 – 7.61 (m, 3H), 7.61 – 7.48 (m, 5H), 7.47 – 7.28 (m, 15H)†, 7.08 (dd, *J* = 7.5, 1.9 Hz, 2H), 6.88 – 6.82 (m, 2H). ^{13}C{^{1}H} NMR (125 MHz, CH_2Cl_2) δ 194.5 (d, *J* = 28.2 Hz), 158.7, 156.9 (d, *J* = 2.5 Hz), 156.8, 149.6, 147.4 (d, *J* = 254.0 Hz), 133.9, 133.6, 132.7, 132.2, 131.8, 131.8, 131.6, 131.6, 131.6, 130.5, 130.1, 130.0, 129.7, 129.4, 129.0, 128.9, 127.3 (d, *J* = 42.8 Hz), 126.7 (d, *J* = 2.3 Hz), 126.1 (d, *J* = 4.0 Hz), 122.7 (d, *J* = 10.1 Hz). ^{19}F{^{1}H} NMR (470 MHz, CH_2Cl_2) δ -127.1 (s, 1F), -152.9 (m, 4F). HRMS (ESI+, m/z) calc'd for $C_{38}H_{27}NF$ [M-BF_4]$^+$: 516.2122, found 516.2103.

Resonance marked with a † are overlapped with (trifluoromethoxy)benzene.

3d-TPPy

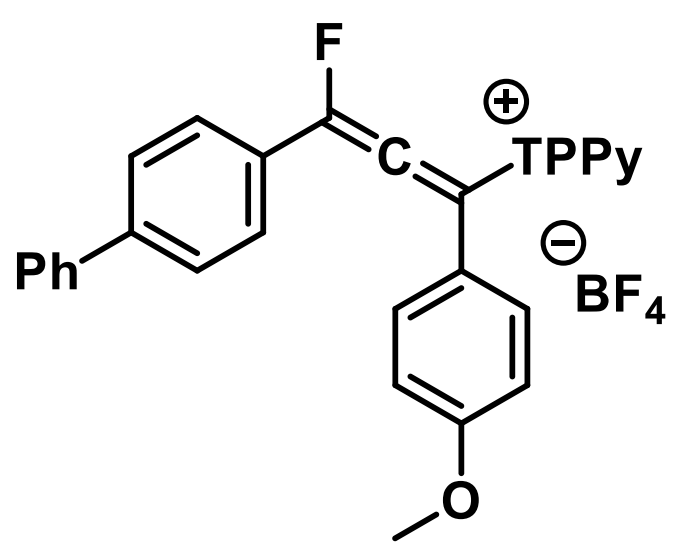

Compound **3d-TPPy** was prepared from **1d** (10.0 mg, 29.9 μmol) following general procedure A, using 2,4,6-triphenylpyridine as the Lewis base. The reaction reached completion after 10 minutes, with the yield determined to be 96 % by comparison to the $PhOCF_3$ internal standard (1.0 eqv, 4.00 μL) in ^{19}F NMR. Standard work-up gave the product an off-white solid. This residue was redissolved in $CDCl_3$ and characterised.

^{1}H NMR (500 MHz, $CDCl_3$) δ 8.13 (dd, *J* = 21.6, 2.2 Hz, 2H), 7.99 – 7.93 (m, 2H), 7.70 – 7.62 (m, 2H), 7.62 – 7.31 (m, 19H), 7.23 – 7.18 (m, 2H), 6.94 – 6.81 (m, 4H), 3.82 (s, 3H). ^{13}C{^{1}H} NMR (125 MHz, $CDCl_3$) δ 191.7 (d, *J* = 25.8 Hz), 162.0, 158.9, 156.2 (d, *J* = 2.7 Hz), 155.9 (d, *J* = 2.5 Hz), 145.6 (d, *J* = 252.5 Hz), 143.9, 139.9, 138.0, 134.4, 133.1, 132.6, 132.3, 131.0 (d, *J* = 11.8 Hz), 129.9, 129.9, 129.3, 129.3, 129.2 (d, *J* = 1.6 Hz), 128.9, 128.7 (d, *J* = 2.2 Hz), 128.6, 128.4, 128.4, 127.7, 127.6, 127.5, 127.2, 126.9, 126.7, 126.3 (d, *J* = 3.6 Hz), 125.4, 123.2 (t, *J* = 5.3 Hz), 55.8. ^{19}F{^{1}H} NMR (470 MHz, $CDCl_3$) δ -125.7 (s, 1F), -151.9 – -154.7 (m, 4F). HRMS (ESI+, m/z) calc'd for $C_{45}H_{33}NOF$ [M-BF_4]$^+$: 622.2541, found 622.2531.

3f-TPPy

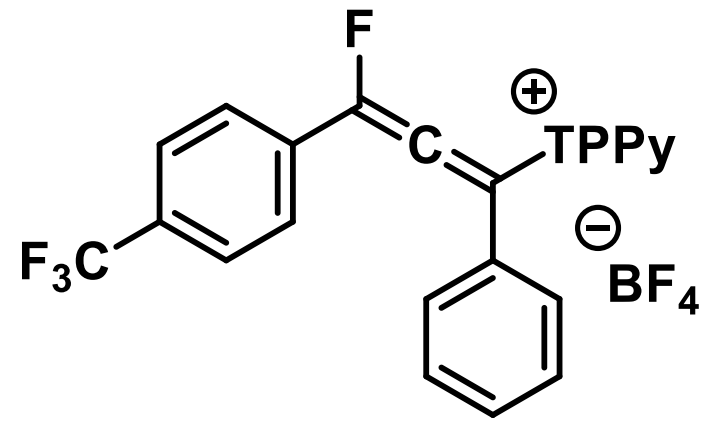

Compound **3f-TPPy** was prepared from **1f** (10.0 mg, 33.7 μmol) following general procedure A, using 2,4,6-triphenylpyridine as the Lewis base. The reaction reached completion after 72 hours, with the yield determined to be 78 % by comparison to the $PhOCF_3$ internal standard (1.0 eqv, 4.45 μL) in ^{19}F NMR. Standard work-up gave the product as an off-white solid.

^1H NMR (500 MHz, $CDCl_3$) δ 8.18 – 8.04 (m, 2H), 8.02 – 7.84 (m, 3H), 7.68 – 7.57 (m, 3H), 7.59 – 7.49 (m, 3H), 7.51 – 7.45 (m, 4H), 7.45 – 7.38 (m, 1H), 7.40 – 7.32 (m, 4H), 7.33 – 7.27 (m, 2H), 7.22 – 7.16 (m, 2H), 6.89 (d, *J* = 8.1 Hz, 2H). $^{13}C\{^1H\}$ NMR (126 MHz, $CDCl_3$) δ 193.8 (d, *J* = 27.1 Hz), 159.1, 156.1 (d, *J* = 25.3 Hz), 145.1 (d, *J* = 254.1 Hz), 134.3, 133.0, 132.7, 132.1, 131.5, 131.1, 131.0, 130.9, 130.2, 130.1, 130.0, 129.9, 129.9, 129.3, 129.0, 128.6, 127.8, 127.4, 126.8 (d, *J* = 2.3 Hz), 126.3 (d, *J* = 3.7 Hz), 125.9, 123.7, 123.7 (q, *J* = 272.1 Hz). $^{19}F\{^1H\}$ NMR (470 MHz, CH_2Cl_2) δ -63.7 (3F, s), -127.4 (s, 1F), -152.8 (m, 4F). HRMS (ESI+, m/z) calc'd for $C_{39}H_{26}NF_4$ $[M-BF_4]^+$: 584.1996, found 584.2008. $C_{39}H_{26}F_8NB{\cdot}2.5\ H_2O$ Anal. Calc. (%): C 65.38, H 4.36, N 2.06. Found (%): C 65.80, H 4.01, N 2.06, average of two runs.

3j-TPPy

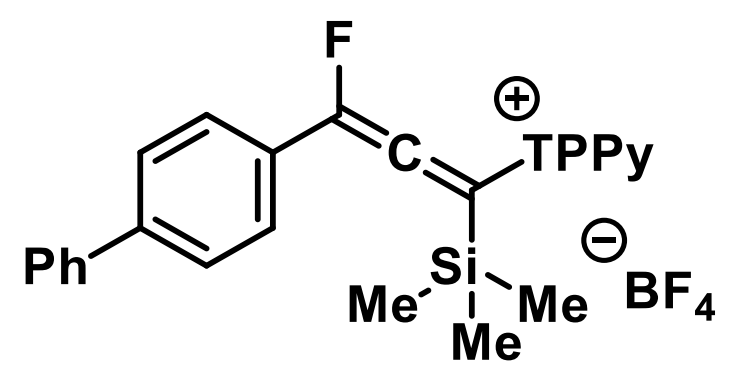

Compound **3j-TPPy** was prepared from **1j** (10.0 mg, 33.2 μmol) following general procedure A, using 2,4,6-triphenylpyridine as the Lewis base. The reaction reached completion after 18 hours, with the yield determined to be 81 % by comparison to the $PhOCF_3$ internal standard (1.0 eqv, 4.50 μL) in ^{19}F NMR.

^1H NMR (500 MHz, CH_2Cl_2) δ 8.21 (d, *J* = 2.3 Hz, 1H), 8.14 (d, *J* = 2.3 Hz, 1H), 7.97 (s, 2H), 7.94 (dd, *J* = 7.0, 1.9 Hz, 2H), 7.81 (dd, *J* = 7.1, 1.9 Hz, 2H), 7.70 (s, 4H), 7.69 – 7.59 (m, 7H), 7.60 – 7.47 (m, 11H), 7.09 – 7.01 (m, 2H), -0.20 (s, 9H). $^{13}C\{^1H\}$ NMR (125 MHz, CH_2Cl_2) δ 204.1 (d, *J* = 31.1 Hz), 156.9, 155.4, 155.3, 147.1 (d, *J* = 247.9 Hz), 144.1, 138.7, 138.6, 133.6,

133.5, 133.3, 132.5, 132.2, 132.0, 130.5, 130.3, 130.0, 129.9, 129.8, 129.7, 129.5, 129.3, 128.7 (d, J = 2.0 Hz), 127.9, 127.7, 126.9 (d, J = 9.2 Hz), 126.5 (d, J = 4.3 Hz), 118.2, -1.50. $^{19}F\{^1H\}$ NMR (470 MHz, CH_2Cl_2) δ -141.5 (s, 1F), -152.6 (m, 4F). HRMS (ESI+, m/z) calc'd for $C_{38}H_{27}NF$ [M+H -(TMS, BF_4)]$^+$: 516.2122, found 516.2112. In all cases, sample was found to undergo protodesilylation under ESI conditions.

N.B. This reaction was run with 1.5 equivalents of 2,4,6-triphenylpyridine as the product exists in an apparent equilibrium with the starting materials.

3k-TPPy

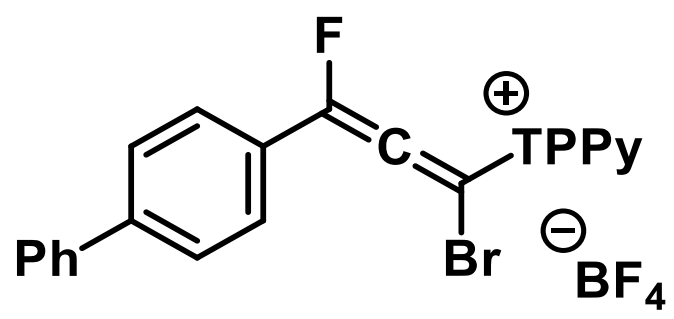

Compound **3k-TPPy** was prepared from **1k** (10.0 mg, 32.6 µmol) following general procedure A, using 2,4,6-triphenylpyridine as the Lewis base. The reaction reached completion after 18 hours, with the yield determined to be 96 % by comparison to the $PhOCF_3$ internal standard (1.0 eqv, 4.25 µL) in ^{19}F NMR.

^1H NMR (500 MHz, CH_2Cl_2) δ 8.20 (d, J = 2.3 Hz, 1H), 8.15 – 8.11 (m, 3H), 8.03 (s, 1H), 7.94 (dd, J = 7.2, 1.8 Hz, 2H), 7.87 – 7.80 (m, 3H), 7.80 – 7.74 (m, 2H), 7.74 – 7.66 (m, 3H), 7.64 (dd, J = 7.7, 1.6 Hz, 4H), 7.58 (td, J = 7.5, 7.0, 2.4 Hz, 3H), 7.54 – 7.47 (m, 2H), 6.93 – 6.87 (m, 2H). $^{13}C\{^1H\}$ NMR (125 MHz, CH_2Cl_2) δ 159.3, 156.5, 156.2, 156.1, 149.1 (d, J = 138.7 Hz), 146.4, 145.5, 139.8, 133.9, 133.7, 132.6, 132.2, 131.9, 131.0, 130.8, 130.5, 130.4, 130.0, 129.9 (d, J = 2.7 Hz), 129.6, 129.5, 129.0, 129.0, 128.1, 128.0, 127.9, 127.6, 127.5 (d, J = 4.5 Hz), 127.2, 127.0. $^{19}F\{^1H\}$ NMR (470 MHz, CH_2Cl_2) δ -121.8 (s, 1F), -152.5 (m, 4F). HRMS (ESI+, m/z) calc'd for $^{79}BrC_{38}H_{26}NF$ [M-BF_4]$^+$: 594.1227, found 594.1229.

2m-TPPy

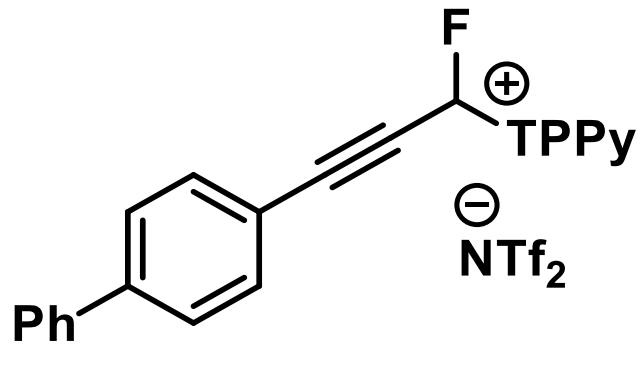

Compound **2m-TPPy** was prepared from **1m** (10.0 mg, 43.8 µmol) following general procedure B, using tris(pentafluorophenyl)borane as the Lewis acid and TPPy as the Lewis

base. The reaction reached 60% completion after one hour forming **2m-TPPy** formed in 43% as adjudged by ^{19}F NMR analysis. Longer reaction times led to decomposition of **2m-TPPy**.

^{19}F NMR (470 MHz, CH_2Cl_2) δ -79.8 (s, 6F), -111.0 (d, 1F, *J* = 45.8 Hz).

Synthesis of Sulfonium Salts

3a-THT

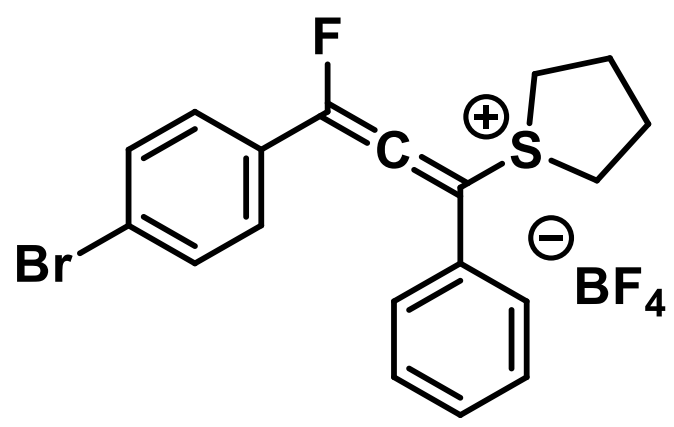

Compound **3a-THT** was prepared from **1a** (10.0 mg, 32.6 μmol) following general procedure A, using tetrahydrothiophene as the Lewis base. The reaction reached completion after 18 hours, with the yield determined to be 85% by comparison to the $PhOCF_3$ internal standard (1.0 eqv, 4.30 μL) in ^{19}F NMR.

^{1}H NMR (600 MHz, CD_2Cl_2) δ 7.74 – 7.69 (m, 2H), 7.69 (s, 0H), 7.68 – 7.65 (m, 2H), 7.62 – 7.53 (m, 3H), 7.48 – 7.38 (m, 3H), 4.06 (dt, *J* = 14.0, 7.2 Hz, 1H), 3.97 (dt, *J* = 13.3, 7.4 Hz, 1H), 3.50 – 3.39 (m, 7H)†, 2.55 – 2.41 (m, 3H), 2.32 (dt, *J* = 13.2, 6.6 Hz, 1H), 2.28 – 2.19 (m, 1H). ^{13}C{^{1}H} NMR (150 MHz, CD_2Cl_2) δ 193.5 (d, *J* = 31.0 Hz), 150.5 (d, *J* = 254.3 Hz), 133.2, 132.7, 130.5, 128.7, 128.6 (d, *J* = 2.2 Hz), 127.4 (d, *J* = 4.1 Hz), 126.9, 126.0 (d, *J* = 30.4 Hz), 115.7 (d, *J* = 9.3 Hz), 47.4, 45.6, 29.1, 29.1. ^{19}F{^{1}H} NMR (470 MHz, DCM-*H_2)* δ -130.1 (s, 1F), -151.3 (m, 4F). HRMS (ESI+, m/z) calc'd for $^{79}BrC_{15}H_9F$ [M-(THT, BF_4)]$^+$: 286.9866, found 298.9880, overlapped with $^{79}BrC_{15}H_{10}O$ [M+OH -(F, THT, BF_4)]$^+$: 284.9910, found 284.9917.

Resonance marked with a † are overlapped with residual diethyl ether.

3c-THT

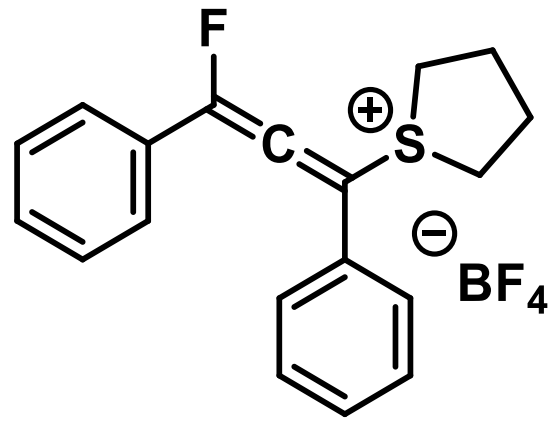

Compound **3c-THT** was prepared from **1c** (10.0 mg, 43.8 μmol) following general procedure A, using tetrahydrothiophene as the Lewis base. The reaction reached completion after 24 hours, with the yield determined to be 96% by comparison to the $PhOCF_3$ internal standard (1.0 eqv, 5.75 μL) in ^{19}F NMR.

^{1}H NMR (500 MHz, CH_2Cl_2) δ 7.70 (dd, *J* = 7.7, 2.0 Hz, 2H), 7.61 – 7.48 (m, 8H), 4.02 (ddt, *J* = 27.6, 13.8, 7.2 Hz, 2H), 3.45 (dt, *J* = 13.2, 6.6 Hz, 2H), 2.55 – 2.40 (m, 2H), 2.31 (dt, *J* = 12.8, 6.6 Hz, 1H), 2.23 (ddd, *J* = 14.6, 13.0, 7.3 Hz, 1H). $^{13}C\{^{1}H\}$ NMR (125 MHz, CH_2Cl_2) δ 193.7 (d, *J* = 31.6 Hz), 151.9, 150.9 (d, *J* = 256.7 Hz), 132.1 (d, *J* = 20.2 Hz), 130.0 (d, *J* = 29.4 Hz), 128.3 – 127.9 (m), 126.5 (d, *J* = 29.7 Hz), 125.7 – 125.2 (m), 114.7 (d, *J* = 9.4 Hz), 46.9, 45.2, 28.8, 28.7.$^{19}F\{^{1}H\}$ NMR (470 MHz, CH_2Cl_2) δ -129.9 (s, 1F), -151.8 (m, 4F). HRMS (ESI+, m/z) calc'd for $C_{15}H_{10}F$ [M -(THT, BF_4)]$^{+}$: 209.0761, found 209.0755, overlapped with $C_{15}H_{11}O$ [M+OH -(F, THT, BF_4)]$^{+}$: 207.0804, found 207.0802.

3g-THT

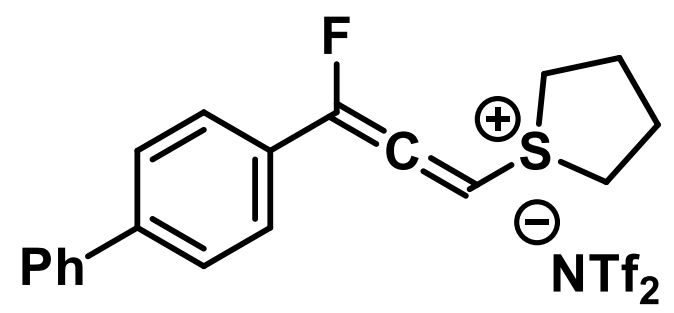

Compound **3g-THT** was prepared from **1g** (10.0 mg, 43.8 μmol) following general procedure B, using tetrahydrothiophene as the Lewis base. The reaction reached completion after 15 minutes, with the yield determined to be 98% by comparison to the $PhOCF_3$ internal standard (1.0 eqv, 5.75 μL) in ^{19}F NMR. Full characterisation could not be acquired for this compound as it rapidly decomposes. Partial characterisation is reported.

^{1}H NMR (500 MHz, $CDCl_3$) δ 7.79 – 7.73 (m, 2H), 7.67 – 7.62 (m, 2H), 7.59 – 7.53 (m, 2H), 7.51 – 7.45 (m, 3H), 7.45 – 7.38 (m, 3H)†, 3.81 (tt, *J* = 13.2, 6.4 Hz, 2H), 3.74 – 3.54 (m, 2H), 2.46 – 2.27 (m, 6H). ^{19}F NMR (470 MHz, $CDCl_3$) δ -78.1 (s, 6F), -132.37 (s, 1F).

Resonance marked with a † are overlapped with (trifluoromethoxy)benzene.

3l-THT

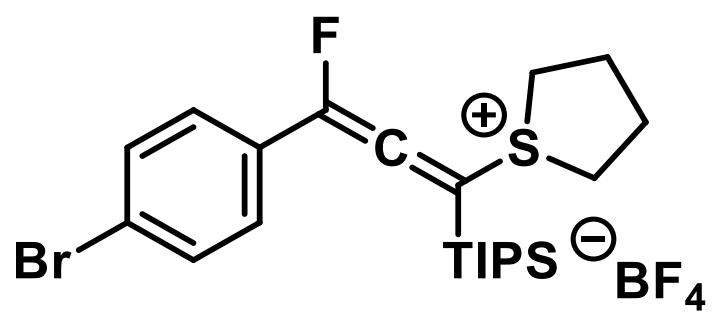

Compound **3l-THT** was prepared from **1l** (10.0 mg, 25.8 μmol) following general procedure A, using tetrahydrothiophene as the Lewis base. After 30 minutes the yield was determined to be 54 % by comparison to the $PhOCF_3$ internal standard (1.0 eqv, 5.75 μL) in ^{19}F NMR. Further reaction time led to decomposition of the compound, similarly, attempts to isolate the compound were unsuccessful. Partial characterisation is reported.

^{1}H NMR (500 MHz, CH_2Cl_2) δ 7.29 – 7.23 (m, 2H), 7.08 – 7.03 (m, 3H)*, 3.59 – 3.42 (m, 1H), 3.23 (ddt, J = 17.2, 13.4, 6.8 Hz, 9H)†, 2.91 (ddt, J = 19.7, 13.4, 6.2 Hz, 2H), 2.07 – 1.84 (m, 4H). ^{19}F NMR (470 MHz, CH_2Cl_2) δ -127.52 (s, 1F), -152.71 (s, 4F).

Resonance marked with a * are overlapped with starting material, resonance marked with a † are overlapped with diethyl ether. TIPS resonances for the product are indistinguishable from those of the starting material.

Nucleophilic Substitution of Sulfonium Salts

2a-Py

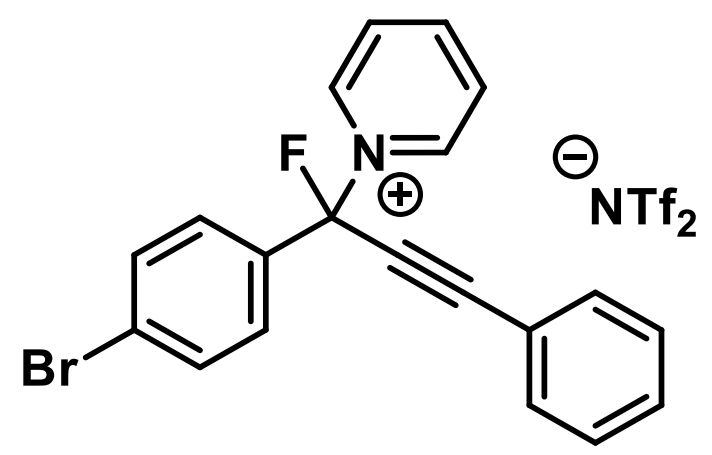

Compound **2a-Py** was prepared in two steps starting with the C-F bond activation of **1a** (10.0 mg, 32.6 μmol) following General Method B, where $BF_3{\cdot}OEt_2$ was used as the Lewis acid, and tetrahydrothiophene the Lewis base. After the C-F bond activation had reached completion, pyridine (5.14 mg, 65.1 μmol) was added to the reaction solution. Across the two steps, the yield was determined to be 91 % by comparison to a $PhOCF_3$ internal standard (1.0 eqv, 4.30 μL) in ^{19}F NMR.

^{1}H NMR (600 MHz, CD_2Cl_2) δ 9.18 – 9.11 (m, 2H), 8.73 (tt, *J* = 7.7, 1.3 Hz, 1H), 8.32 – 8.26 (m, 2H), 7.77 (dd, *J* = 8.9, 0.8 Hz, 1H), 7.70 – 7.64 (m, 3H), 7.59 – 7.53 (m, 1H), 7.49 – 7.44 (m, 2H). ^{19}F NMR (565 MHz, CD_2Cl_2) δ -79.4 (s, 6F), -100.6 (s, 1F). $^{13}C\{^{1}H\}$ NMR (151 MHz, CD_2Cl_2) δ 149.6, 140.9 (d, *J* = 3.0 Hz), 133.8, 133.0 (d, *J* = 2.8 Hz), 132.5 (d, *J* = 26.3 Hz), 132.2, 129.8, 129.3, 128.4 (d, *J* = 5.0 Hz), 128.1 (d, *J* = 2.1 Hz), 126.7 (d, *J* = 2.4 Hz), 120.2 (q, *J* = 321.4 Hz), 118.3 (d, *J* = 3.5 Hz), 104.3 (d, *J* = 220.2 Hz), 99.2 (d, *J* = 7.0 Hz), 78.5 (d, *J* = 30.9 Hz). ^{19}F NMR (565 MHz, Methylene Chloride-*d*$_2$) δ -79.4 (s, 6F), -100.6 (s, 1F). HRMS (ESI+, m/z) calc'd for $^{79}BrC_{20}H_{14}FN$ [M-NTf$_2$]$^+$: 366.0288, found 366.0283

3a-PhPy

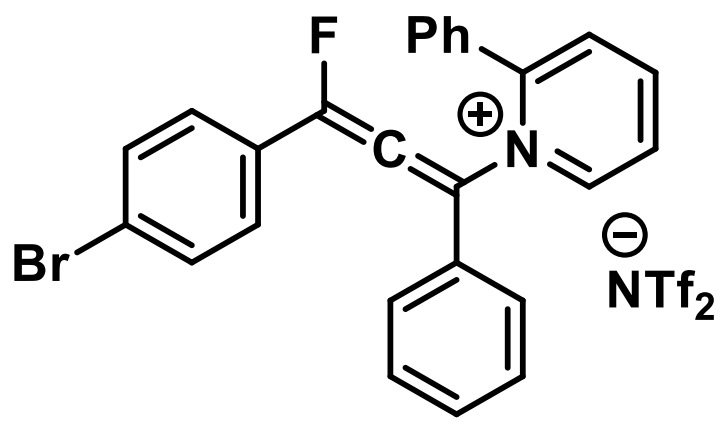

Compound **3a-PhPy** was prepared in two steps starting with the C-F bond activation of **1a** (10.0 mg, 32.6 µmol) following General Method B, where $BF_3{\cdot}OEt_2$ was used as the Lewis acid, and tetrahydrothiophene the Lewis base. After the C-F bond activation had reached completion, 2-phenylpyridine (5.1 mg, 35.8 µmol) was added to the reaction and the resulting solution heated at 45 °C for 5 hours. Across the two steps, the yield was determined to be 64 % by comparison to a $PhOCF_3$ internal standard (1.0 eqv, 4.30 µL) in ^{19}F NMR.

^{1}H NMR (500 MHz,CH_2Cl_2) δ 9.11 (d, *J* = 6.1 Hz, 1H), 9.02 – 8.94 (m, 1H), 8.51 – 8.45 (m, 1H), 8.35 (d, *J* = 8.0 Hz, 1H), 7.80 – 7.66 (m, 2H), 7.62 (t, *J* = 7.4 Hz, 4H), 7.58 – 7.54 (m, 2H), 7.30 (d, *J* = 7.9 Hz, 2H), 7.27 (dd, *J* = 8.6, 1.8 Hz, 3H). $^{13}C\{^{1}H\}$ NMR (151 MHz, CD_2Cl_2) δ 192.2 (d, *J* = 29.7 Hz), 148.4, 145.7, 132.5, 132.1, 132.0, 131.5, 130.8, 129.9, 129.8, 129.5, 128.9, 127.7, 127.1 (d, *J* = 4.1 Hz), 126.5 (d, *J* = 2.3 Hz), 126.1, 119.8 (q, *J* = 321.4 Hz). ^{19}F NMR (470 MHz, CH_2Cl_2) δ -79.18 (s, 6F), -124.29 (s, 1F). HRMS (APCI+, m/z) calc'd for $^{79}BrC_{15}H_{10}O$ [M+OH -(F, THT, BF_4)]$^+$: 284.9910, found 284.9904.

5a

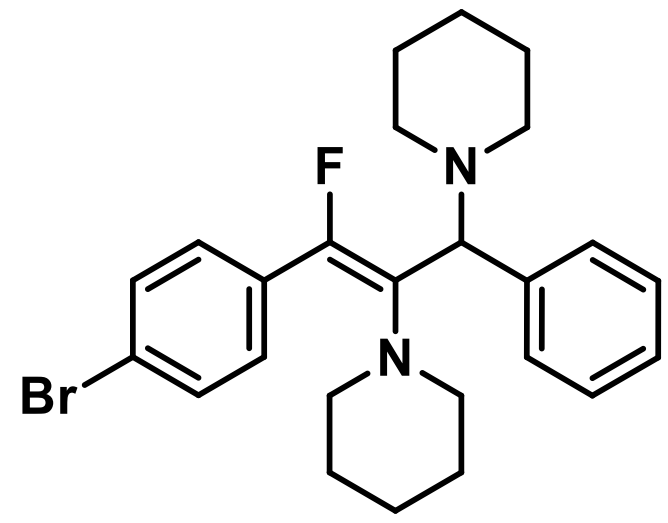

Compound **5a** was prepared by dissolving compound **2a-Py** in piperidine (0.6 mL). After 30 minutes of stirring the solvent was removed *in vacuo*. The compound was redissolved in dichloromethane (1.0 mL) and stirred for five minutes before the solvent was removed again. This process was repeated three times, giving the final product as a viscous red oil. A yield of 93% was determined by ^{19}F NMR, compared to a $PhOCF_3$ internal standard (1.0 eqv, 4.30 µL).

^{1}H NMR (600 MHz, C_6D_6) δ 6.97 (dd, J = 8.4, 1.5 Hz, 2H), 6.83 (tq, J = 3.7, 2.1, 1.4 Hz, 3H), 6.69 – 6.58 (m, 2H), 6.54 – 6.40 (m, 2H), 5.85 (d, J = 1.4 Hz, 1H), 3.79 (q, J = 5.4 Hz, 4H), 2.73 (td, J = 8.5, 5.3 Hz, 4H), 1.78 – 1.64 (m, 4H), 1.41 (p, J = 6.0 Hz, 5H), 1.31 (p, J = 6.1 Hz, 4H), 1.03 (q, J = 5.5 Hz, 5H), 0.98 (p, J = 6.1 Hz, 2H). ^{13}C{^{1}H} NMR (151 MHz, C_6D_6) δ 171.5 (d, J = 199.4 Hz), 135.0, 133.8, 131.3, 130.9, 129.7, 129.4, 128.1, 128.0, 124.4, 120.9 (q, J = 321.9 Hz), 97.6, 51.8, 51.8, 51.3, 51.2, 26.3, 26.3, 26.1, 25.6, 23.2, 23.2. ^{19}F NMR (470 MHz, CH_3CN) δ -79.3 (s, 6F), -129.8 (bs, 1F). HRMS (ESI+, m/z) calc'd for $^{79}BrC_{25}H_{30}N_2$ $[M-F]^+$: 437.1587, found 437.1588.

Allenic C2 nucleophilic attack of phosphonium salts

5b

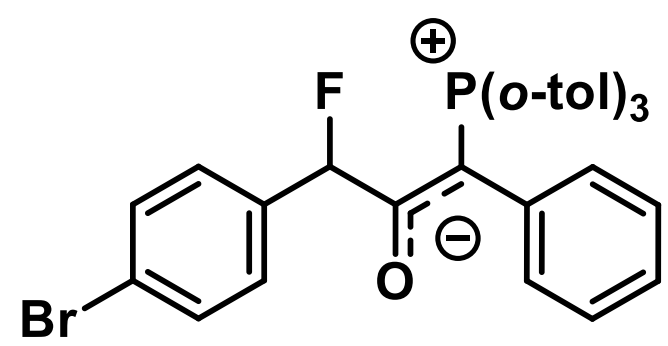

Compound **5b** was prepared in two steps starting with the C-F bond activation of **1a** (10.0 mg, 32.6 µmol) following General Method A, with tri(*o*-tolyl)phosphine the Lewis base. After the C-F bond activation had reached completion, the solution was evaporated to dryness and the residue redissolved in tetrahydrofuran (1.0 mL). To this was added aqueous potassium hydroxide solution (0.4 M, 40.0 µmol, 100 µL) and the reaction allowed to stir for on hour. The

solution was then diluted with dichloromethane (2.0 mL), and the organics washed with aqueous hydrochloric acid (1 M, 3 × 1 mL), dried over sodium sulphate and then concentrated *in vacuo*. This residue was triturated from dichloromethane/hexane three times and the resulting off-white solid characterised. Yield 83 % by ^{19}F NMR compared to a $PhOCF_3$ internal standard (1.0 eqv, 4.30 μL), mixture of diastereomers, approximately 3:1.

^{1}H NMR (900 MHz, CD_2Cl_2) δ 8.45 (dd, J = 16.5 ($^3J_{PH}$)*, 7.8 ($^3J_{HH}$) Hz, 1H), 8.07 (d, J = 20.4 ($^3J_{PH}$) Hz)†, 7.67 – 7.32 (m, 11H), 7.26 (dd, J = 23.5, 8.3 Hz, 3H), 7.23 – 7.15 (m, 4H), 7.10 (d, J = 7.3 Hz, 1H), 6.87 – 6.80 (m, 3H), 6.78 (d, J = 7.9 Hz, 2H), 6.16 (d, J = 48.3 Hz ($^2J_{FH}$), 1H)*, 5.75 (d, J = 48.6 ($^2J_{FH}$) Hz)†, 2.94 (s)†, 2.16 (s, 3H)*, 1.60 (s)†, 1.55 (s, 3H)*, 0.97 (s, 3H)*, 0.94 (s)†. ^{13}C{^{1}H} NMR (225 MHz, CD_2Cl_2) δ 182.0 (d, J = 25.8 ($^2J_{PC}$) Hz), 181.7, 144.6, 142.7, 142.1, 138.1, 137.6 (d, J = 14.7 ($^2J_{FC}$) Hz), 137.2, 137.1, 136.5 (d, J = 13.7 ($^2J_{FC}$) Hz), 135.9, 134.4, 134.2, 133.1 – 132.2 (m), 131.9, 131.6, 131.2, 130.9, 128.9, 128.1 (d, J = 87.9 ($^1J_{PC}$) Hz), 127.2 (d, J = 13.4 ($^3J_{PC}$) Hz), 126.4 (d, J = 13.2 ($^3J_{PC}$) Hz), 126.2, 125.8, 124.7 (d, J = 83.6 ($^1J_{PC}$) Hz), 122.9 (d, J = 81.7 ($^1J_{PC}$) Hz), 122.4 (d, J = 96.1 ($^1J_{PC}$) Hz), 122.3, 90.7 (dd, J = 175.8 ($^1J_{FC}$), 13.2 ($^3J_{PC}$) Hz), 89.5, 70.4, 69.9. ^{19}F NMR (565 MHz, CD_2Cl_2) δ -164.3 (d, J = 48.3 Hz)†, -177.4 (d, J = 48.2 Hz)*. ^{31}P{^{1}H} NMR (202 MHz, CD_2Cl_2) δ 17.5*, 16.1†. HRMS (ESI+, m/z) calc'd for $^{79}BrC_{36}H_{32}FOP$ [M-BF_4]$^+$: 609.1353, found 609.1333.

Peaks assigned to the major diastereomer are marked with a *, peaks assigned to the minor diastereomer are marked with a †.

5c

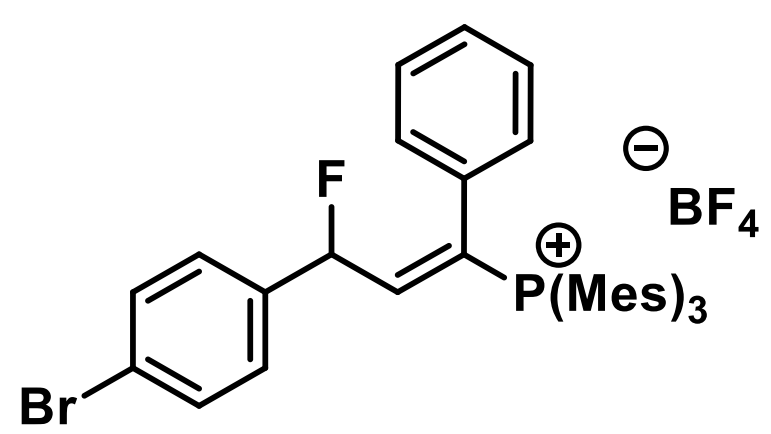

Compound **5c** was prepared in two steps starting with the C-F bond activation of **1a** (10.0 mg, 32.6 μmol) following General Method A, with tri(mesityl)phosphine the Lewis base. After the C-F bond activation had reached completion, the solution was evaporated to dryness and the residue redissolved in tetrahydrofuran (1.0 mL). This solution was cooled to -78 °C and $LiHBEt_3$ (1.0 M in tetrahydrofuran, 35 μmol, 35 μL) was added dropwise. The resulting bright-red solution was allowed to stir at -78 °C for one hour. Subsequently, the solvent was removed *in vacuo* at -78 °C and the residue thoroughly dried. This was once again redissolved

in THF (1.0 mL) and cooled to -78 °C, and hydrochloric acid in diethyl ether (2.0 M, 35 μmol, 35 μL) was added dropwise and the reaction allowed to come to room temperature over one hour. The solvent was removed *in vacuo* and the residue dissolved in dichloromethane (2.0 mL), the organics were washed with aqueous hydrochloric acid (1 M, 3 × 1 mL), dried over sodium sulphate and the solvent removed *in vacuo*. This residue was triturated from dichloromethane/hexane three times and the resulting yellow oil characterised. Yield 74 % by ^{19}F NMR compared to a $PhOCF_3$ internal standard (1.0 eqv, 4.30 μL), mixture of diastereomers, approximately 1:1.

^{1}H NMR (600 MHz, $CDCl_3$) δ 7.55 (d, J = 8.1 Hz, 2H), 7.55 – 7.47 (m, 2H), 7.46 – 7.38 (m, 3H), 7.36 (t, J = 7.7 Hz, 3H), 7.31 (t, J = 7.7 Hz, 1H), 7.06 (dd, J = 13.0, 4.9 Hz, 7H), 7.00 – 6.93 (m, 5H), 6.92 – 6.79 (m, 6H), 6.72 – 6.66 (m, 2H), 6.68 – 6.59 (m, 1H)†, 6.59 (d, J = 8.0 Hz, 2H), 6.43 (ddd, J = 21.9 ($^3J_{PH}$), 10.4 ($^3J_{FH}$), 8.5 ($^3J_{HH}$) Hz, 1H)*, 5.65 (ddd, J = 46.5 ($^2J_{FH}$), 8.6 ($^3J_{HH}$), 2.5 Hz ($^4J_{PH}$), 1H)*, 5.40 (ddd, J = 47.0 ($^2J_{FH}$), 8.4 ($^3J_{HH}$), 2.4 Hz ($^4J_{PH}$), 1H)†, 2.36 (s, 9H), 2.36 (s, 9H), 2.06 (s, 9H), 2.05 (s, 9H), 1.88 – 1.69 (m, 18H). ^{13}C{^{1}H} NMR (150 MHz, $CDCl_3$) δ 153.5 (dd, J = 32.7 ($^2J_{PC}$), 11.8 ($^2J_{FC}$) Hz)†, 152.9 (dd, J = 31.0 ($^2J_{PC}$), 11.4 ($^2J_{FC}$) Hz)*, 146.0 (d, J = 7.4 Hz), 145.5 (d, J = 11.3 Hz), 145.1 – 144.7 (m), 134.2 (d, J = 11.8 Hz ($^2J_{FC}$))†, 134.0 (d, J = 12.0 ($^2J_{FC}$) Hz)*, 133.5 – 133.2 (m), 132.7, 132.4, 130.7, 130.6, 130.2 (d, J = 3.6 Hz), 129.3, 129.0 (d, J = 4.8 Hz), 128.8, 127.8 (d, J = 5.3 Hz), 124.6 (d, J = 33.6 Hz), 119.0 – 116.1 (m), 90.6 (dd, J = 168.5 ($^1J_{FC}$), 15.9 ($^3J_{PC}$) Hz)†, 89.9 (dd, J = 170.6 ($^1J_{FC}$), 16.3 ($^3J_{PC}$) Hz)*, 25.9, 25.4, 24.5 (m), 21.2. ^{19}F NMR (565 MHz, $CDCl_3$) δ -154.1 (s, 4F), -161.0 (dd, J = 47.1 ($^2J_{HF}$), 11.9 ($^3J_{HF}$) Hz)†, -170.4 (dd, J = 46.6 ($^2J_{HF}$), 10.4 ($^3J_{HF}$) Hz)*. ^{31}P{^{1}H} NMR (202 MHz, Chloroform-*d*) δ 17.2*, 17.1†. HRMS (ESI+, m/z) calc'd for $^{79}BrC_{42}H_{44}FP$ $[M-BF_4]^+$: 677.2343, found 677.2326.

Peaks assigned to each diastereomer are marked with a * or a †.

5d

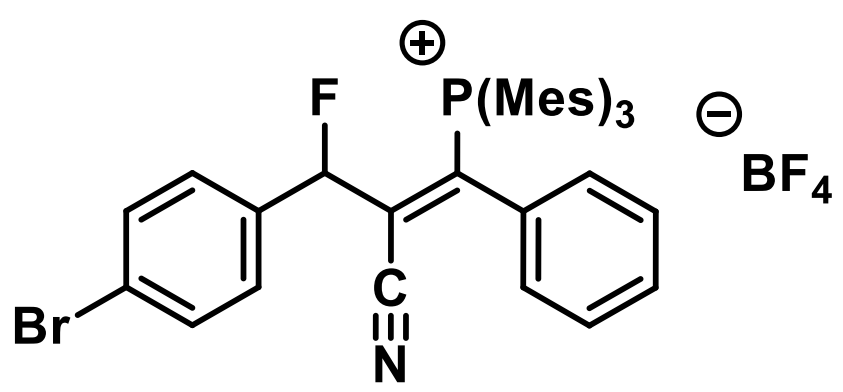

Compound **5d** was prepared in two steps starting with the C-F bond activation of **1a** (10.0 mg, 32.6 μmol) following General Method A, with tri(mesityl)phosphine the Lewis base. After the C-F bond activation had reached completion, tetrabutylammonium cyanide (38.8 μmol, 9.61

mg) dissolved in dichloromethane (0.4 mL) was added dropwise to the reaction mixture and the reaction left to stir for 30 minutes. The resulting dark red solution was cooled to 0 °C and hydrochloric acid in diethyl ether (2.0 M, 200 µmol, 100 µL) was added dropwise. The solution was then warmed to room temperature and left stirring for one hour. The solvent was then removed *in vacuo* and the resulting residue triturated from toluene/*n*-hexanes three times and the resulting sticky oil characterised. Yield 63% by ^{19}F NMR compared to a $PhOCF_3$ internal standard (1.0 eqv, 4.30 µL), mixture of diastereomers, approximately 1:1.

^{1}H NMR (600 MHz, C_6D_6) δ 7.57 (d, J = 7.5 Hz, 1H), 7.43 (dd, J = 15.8, 7.9 Hz, 3H), 7.37 – 7.31 (m, 4H), 7.29 (d, J = 8.3 Hz, 4H), 7.23 – 7.19 (m, 2H), 7.10 (ddt, J = 7.8, 5.8, 2.0 Hz, 4H), 7.04 – 6.99 (m, 5H), 6.88 (dt, J = 8.6, 4.1 Hz, 4H), 6.84 – 6.77 (m, 5H), 6.77 – 6.71 (m, 2H), 6.64 (dt, J = 5.2, 2.3 Hz, 4H), 6.45 (d, J = 8.0 Hz, 1H), 6.40 (d, J = 8.0 Hz, 1H), 6.15 (ddd, J = 7.3, 4.7, 1.8 Hz, 2H), 5.92 (dd, J = 43.9 ($^{2}J_{FH}$), 2.2 Hz ($^{4}J_{PH}$), 1H), 5.60 (dd, J = 45.0 ($^{2}J_{FH}$), 2.2 Hz ($^{4}J_{PH}$), 1H), 2.93 (s, 3H), 2.66 (s, 3H), 2.62 (s, 3H), 2.19 (s, 3H), 2.14 (s, 3H), 2.13 (s, 9H), 2.12 (s, 3H), 2.07 (s, 3H), 2.01 (s, 3H), 1.95 (s, 3H), 1.92 (s, 6H), 1.92 (s, 9H), 1.90 (s, 3H), 1.71 (s, 3H), 1.70 (s, 6H). ^{13}C{^{1}H} NMR (150 MHz, C_6D_6) δ 149.6, 148.8 (d, J = 11.9 Hz), 148.2 (d, J = 3.2 Hz), 148.1 (d, J = 3.1 Hz), 146.7 (d, J = 9.6 Hz), 146.2 (d, J = 9.6 Hz), 146.1 (dd, J = 9.3, 6.5 Hz), 146.0 – 145.7 (m), 145.1 (d, J = 13.2 Hz), 144.8 (d, J = 13.1 Hz), 142.9 (t, J = 8.8 Hz), 135.0 (d, J = 12.5 Hz), 134.6 (d, J = 12.7 Hz), 134.4 – 134.2 (m), 134.2 – 134.0 (m), 133.9 – 133.7 (m), 132.7 (d, J = 36.2 Hz), 132.5 – 132.1 (m), 131.9 (d, J = 5.9 Hz), 131.5 (d, J = 5.3 Hz), 122.8 (d, J = 74.9 Hz), 122.7 (d, J = 74.7 Hz), 116.2 (d, J = 72.9 Hz), 116.1 (d, J = 72.7 Hz), 113.1 (d, J = 13.6 Hz), 112.6 (d, J = 10.2 Hz), 112.5 (d, J = 14.4 Hz), 91.4 (dd, J = 179.8 ($^{1}J_{FC}$), 9.3 ($^{3}J_{PC}$) Hz), 90.6 (dd, J = 183.5 ($^{1}J_{FC}$), 10.2 ($^{3}J_{PC}$) Hz), 27.6 (d, J = 6.1 Hz), 26.8 (d, J = 6.4 Hz), 25.2 (d, J = 7.7 Hz), 25.1 (d, J = 7.3 Hz), 24.5 (d, J = 4.4 Hz), 24.4 (d, J = 4.2 Hz), 24.3 (d, J = 3.6 Hz), 24.0 (d, J = 3.7 Hz), 23.9 (d, J = 4.8 Hz), 23.8 (d, J = 5.0 Hz), 22.9 (d, J = 3.5 Hz), 22.8 (d, J = 3.3 Hz), 21.2 (d, J = 1.3 Hz), 21.0 (d, J = 1.3 Hz), 20.9 (d, J = 1.5 Hz), 20.9 (d, J = 1.4 Hz), 20.9 (d, J = 1.6 Hz), 20.9 (d, J = 1.5 Hz). ^{19}F NMR (565 MHz, C_6D_6) δ -153.7 (4F, m), -158.9 (d, J = 45.0 ($^{2}J_{HF}$) Hz), -170.7 (dd, J = 44.2 ($^{2}J_{HF}$), 2.9 ($^{4}J_{PF}$) Hz). ^{31}P{^{1}H} NMR (202 MHz, C_6D_6) δ 17.8 (d, J = 2.7 ($^{4}J_{PF}$) Hz), 17.1 (d, J = 1.5 ($^{4}J_{PF}$) Hz). HRMS (ESI+, m/z) calc’d for $^{79}BrC_{43}H_{43}FPN$ [M-BF_4]$^{+}$: 702.2295, found 702.2291.

Stereoselective synthesis of allenic onium salts

Stereoselective synthesis of allenic onium salts was tested using both Methods A and B with the enantiopure chiral Lewis bases (LB*) (*R*,*R*)−2,5-dimethylthiolane (**LB$_A$***) and (4*S*,4'*S*)-2,2'-(1,3-phenylene)bis[4,5-dihydro-4-phenyloxazole] (**LB$_B$***) as according to Table SX below

Table S1: Testing chiral Lewis bases for the stereoselective synthesis of compounds **3-LB***. [a] Me_3SiNTf_2 not added.

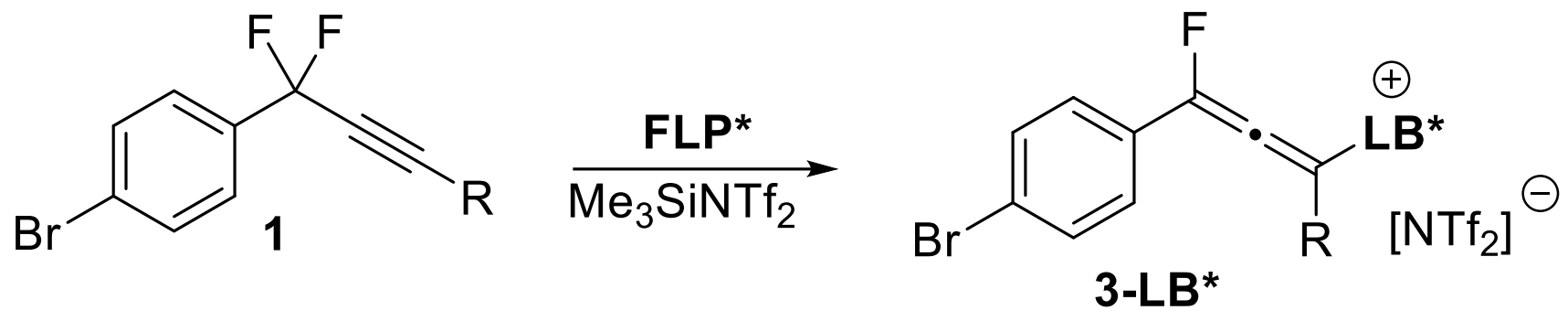

	FLP*						
**entry**	**LA**	**LB* (1 eqv)**	**R**	**temp**	**time**	**temp**	***dr***
**1**	$B(C_6F_5)_3$ 10 mol %	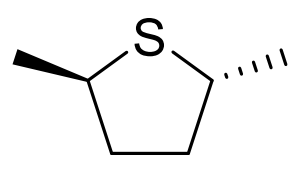 (**LB$_A$***)	Ph (**1a**)	40 °C	48 h	99%	66:34
**2[a]**	BF_3 (1 eqv)	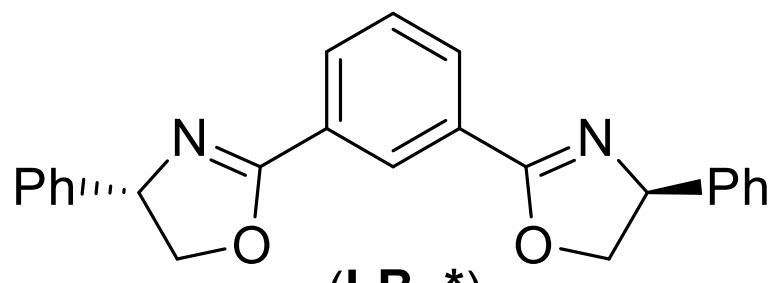 (**LB$_B$***)	Ph (**1a**)	40 °C	5 h	5%	60:40
**3**	BF_3 10 mol %		H (**1h**)	r.t.	24 h	35%	>9:1
**4**	$B(C_6F_5)_3$ 10 mol %		H (**1h**)	r.t.	5 h	0%	-
**5**	BF_3 200 mol %		H (**1h**)	r.t.	24 h	82%	98:2
**6**	BF_3 200 mol %		H (**1h**)	r.t.	48 h	97%	98:2

3h-LB$_B$*

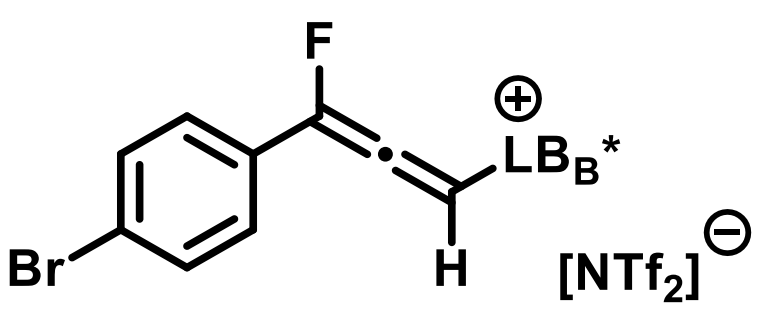

Compound **3h-LB$_B$*** was prepared from **1h** (10.0 mg, 32.6 μmol) following general procedure B but with 200 mol % of BF_3•OEt_2 as Lewis acid, and (4*S*,4'*S*)-2,2'-(1,3-phenylene)bis[4,5-dihydro-4-phenyloxazole] (**LB$_B$***) as the Lewis base (1 eqv, 13.7 mg). The reaction reached completion after 48 hours, with the yield determined to be 97% by comparison to the $PhOCF_3$

internal standard (1.0 eqv, 4.30 μL) in ^{19}F NMR. Two fluoroallenic signals were observed at δ_F -131.6 (major) and -132.0 (minor) in a ratio of 98:2.

Selected data for major isomer

^{1}H NMR (500 MHz,CH_2Cl_2) δ 8.90 (s, 1H), 8.61 (d, *J* = 7.9 Hz, 1H), 8.52-8.45 (m, 5H), 8.33 (d, *J* = 7.9 Hz, 1H), 7.90 (t, *J* = 7.9 Hz, 1H), 7.85 (t, *J* = 7.9 Hz, 1H), 7.74 (t, *J* = 7.9 Hz, 27.48-7.32 (m, 2H), 6.95 (d, *J* = 2.1 Hz, 1H), 5.80 (dd, *J* = 10.6, 8.0 Hz, 1H), 5.76 (dd, *J* = 10.6, 6.4 Hz, 1H), 5.02 (dd, *J* = 9.8, 4.8 Hz, 1H), 4.86 (dd, *J* = 9.5, 6.3 Hz, 1H), 4.82 (dd, *J* = 9.5, 5.8 Hz, 1H), 4.65 5.80 (dd, *J* = 10.0, 4.8 Hz, 1H). ^{13}C{^{1}H} NMR (151 MHz, CD_2Cl_2) (selected data from correlation spectroscopy) δ 196.9 (d, *J* = 32.0 Hz), 173, 170, 145.2 (d, *J* = 253.1 Hz), 138, 135, 132.0, 129, 126.4 (d, *J* = 32.1 Hz), 122, 102.7 (d, *J* = 2.3 Hz), 119.8 (q, *J* = 321.4 Hz), 76.4, 66. ^{19}F NMR (470 MHz, CH_2Cl_2) δ -79.18 (s, 6F), -131.6 (s, 1F). HRMS (ESI+, m/z) calc'd for $^{79}BrC_{33}H_{25}FN_2O_2$ [M-(NTf_2)]$^+$: 579.1083, found 579.1078.

Selected data for minor isomer

^{13}C{^{1}H} NMR (151 MHz, CD_2Cl_2) (selected data from correlation spectroscopy) δ 196.9 (d, *J* = 32.0 Hz), 145.2 (d, *J* = 254.0 Hz), 126.6 (d, *J* = 27.0 Hz), 119.8 (q, *J* = 321.4 Hz). ^{19}F NMR (470 MHz, CH_2Cl_2) δ -79.18 (s, 6F), -132.0 (s, 1F). HRMS (ESI+, m/z) calc'd for $^{79}BrC_{33}H_{25}FN_2O_2$ [M-(NTf_2)]$^+$: 579.1083, found 579.1078.

Kinetic Experiments

Reaction Order

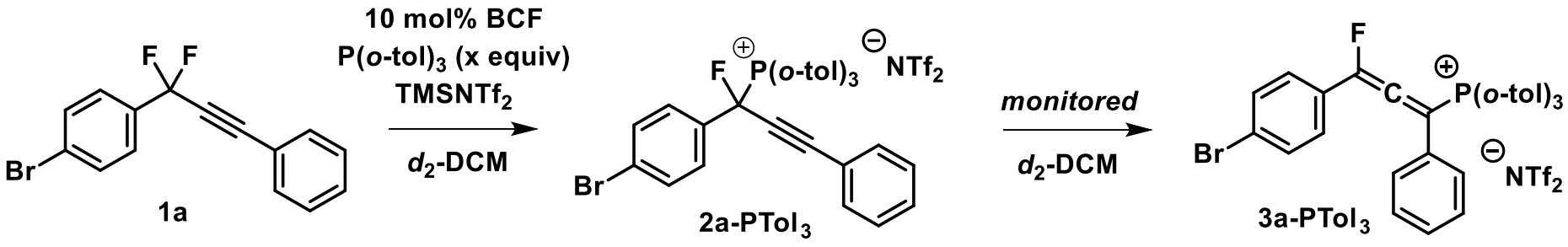

To a d_2-DCM solution (0.2 mL) of **1a** (10.0 mg, 32.6 µmol) was added a d_2-DCM solution (0.4 mL) of tris(pentafluorophenyl)borane (1.66 mg, 3.26 µmol) and tri(*o*-tolyl)phosphine (1, 2, 3, 4, 5 eqv) followed by a d_2-DCM solution (0.2 mL) of *N*-(trimethylsilyl)bis(trifluoromethanesulfonyl)imide (12.6 mg, 35.8 µmol). The resulting solution was used to dissolve bis(triphenylphosphine)iminium tetrakis(3,5-bis(trifluoromethyl)phenyl)borate (8.14 µmol, 11.4 mg) to serve as a ^{31}P NMR internal standard. The resulting sample was measured by ^{31}P NMR at 298 K every five minutes to determine the concentration of alkyne (**2a-PTol₃**) and allene (**3a-PTol₃**) species, allowing the reaction rate to be calculated.

	1 eqv P(*o*-tol)$_3$	2 eqv P(*o*-tol)$_3$	3 eqv P(*o*-tol)$_3$	4 eqv P(*o*-tol)$_3$	5 eqv P(*o*-tol)$_3$
Consumption of **2a-PTol$_3$**	-1.48 E-06 (M/s)	-1.63 E-06 (M/s)	-1.64 E-06 (M/s)	-1.51 E-06 (M/s)	-1.57 E-06 (M/s)
Formation of **3a-PTol$_3$**	1.44 E-06 (M/s)	1.66 E-06 (M/s)	1.46 E-06 (M/s)	1.43 E-06 (M/s)	1.46 E-06 (M/s)

Table S2: Observed rates of isomerisation at various equivalents of tri(*o*-tolyl)phosphine.

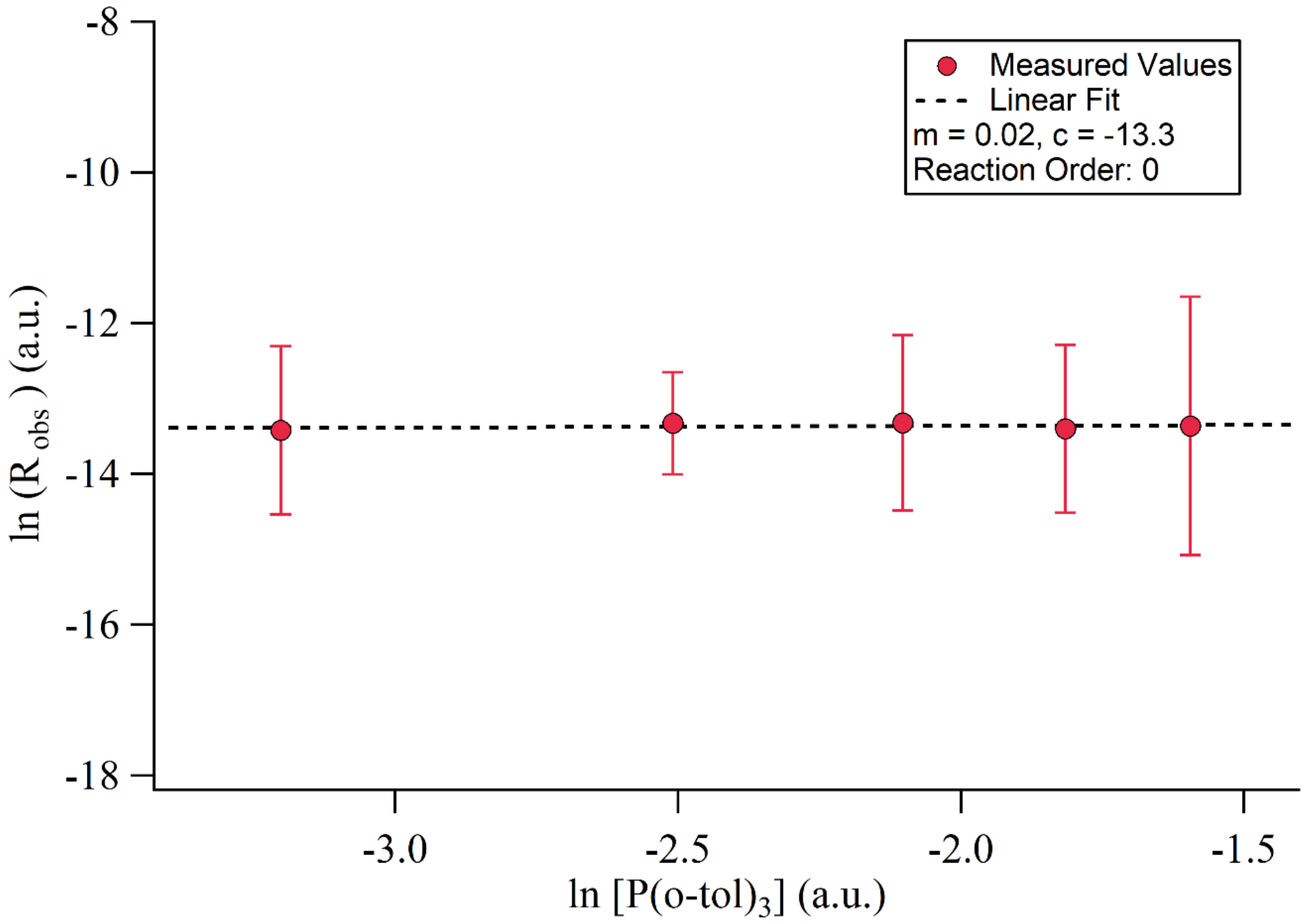

Figure S1: Reaction order plot for isomerisation using formation rate data showing no correlation between reaction rate and equivalents of tri(*o*-tolyl)phosphine.

Activation Barrier to Isomerisation

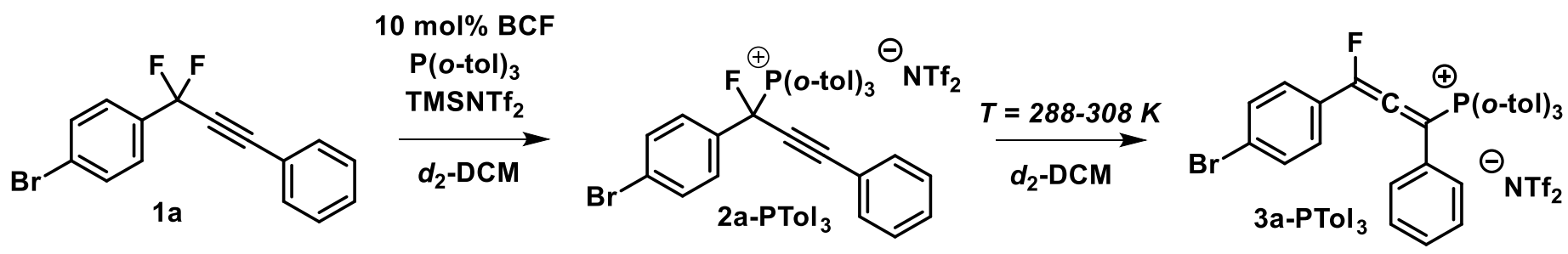

To a d_2-DCM solution (0.2 mL) of **1a** (10.0 mg, 32.6 μmol) was added a d_2-DCM solution (0.4 mL) of tris(pentafluorophenyl)borane (1.66 mg, 3.26 μmol) and tri(*o*-tolyl)phosphine (65.10 μmol, 19.7 mg) followed by a d_2-DCM solution (0.2 mL) of *N*-(trimethylsilyl)bis(trifluoromethanesulfonyl)imide (12.6 mg, 35.8 μmol). The resulting solution was used to dissolve bis(triphenylphosphine)iminium tetrakis(3,5-bis(trifluoromethyl)phenyl)borate (8.14 μmol, 11.4 mg) to serve as a ^{31}P NMR internal standard. The resulting sample was measured by ^{31}P NMR at 298 K every five minutes to determine the concentration of (**2a-PTol3**) and allene (**3a-PTol3**) species, allowing the reaction rate to be calculated. This was repeated at 288 K, 293 K, 303 K and 308 K.

Rate	288 K	293 K	298 K	303 K	308 K
Consumption of **2a-PTol3**	-6.56 E-07 (M/s)	-9.77 E-07 (M/s)	-1.63 E-06 (M/s)	-2.34 E-06 (M/s)	-4.11 E-06 (M/s)
Formation of **3a-PTol3**	3.27 E-07 (M/s)	6.97 E-07 (M/s)	1.66 E-06 (M/s)	2.28 E-06 (M/s)	3.75 E-06 (M/s)

Table S3: Observed rates of isomerisation at various temperatures.

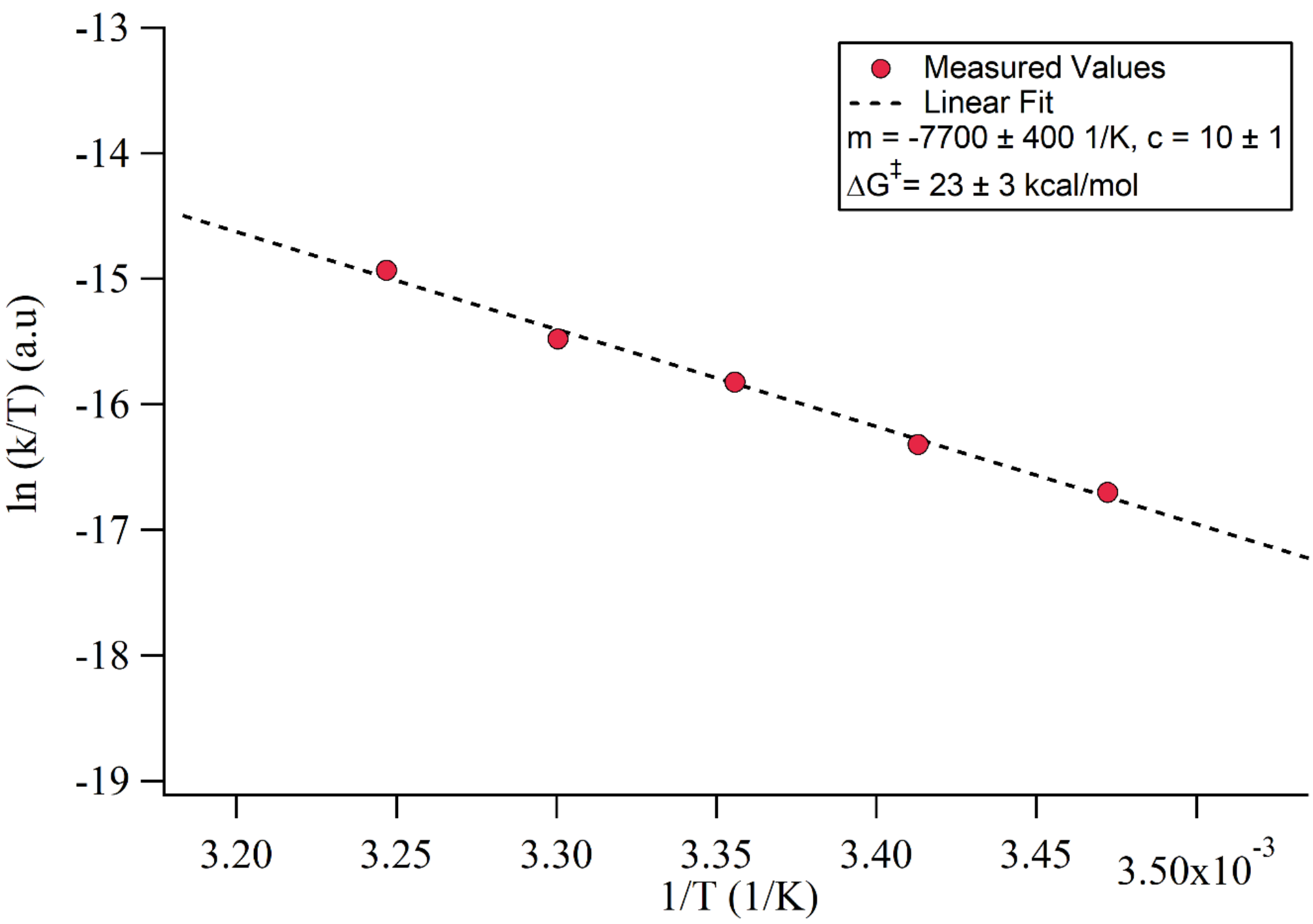

Figure S2: Eyring plot for isomerisation using consumption rate data. The energy barrier to isomerisation is estimated to be 23 ± 3 kcal/mol.

Variable Temperature ^{1}H and ^{31}P NMR Experiments

Variable temperature experiments were conducted using a 54 mM solution of **3a-PTol3** in 1,2-DCE. The temperature of the sample was raised or lowered 5 K at a time and allowed to stabilise, once the temperature had stabilised, ^{1}H and ^{19}F NMR spectra were collected. After the sample was returned to room temperature ^{1}H and ^{19}F NMR spectra were collected again to ascertain if any decomposition of the sample had occurred; across heating and cooling decomposition was not observed.

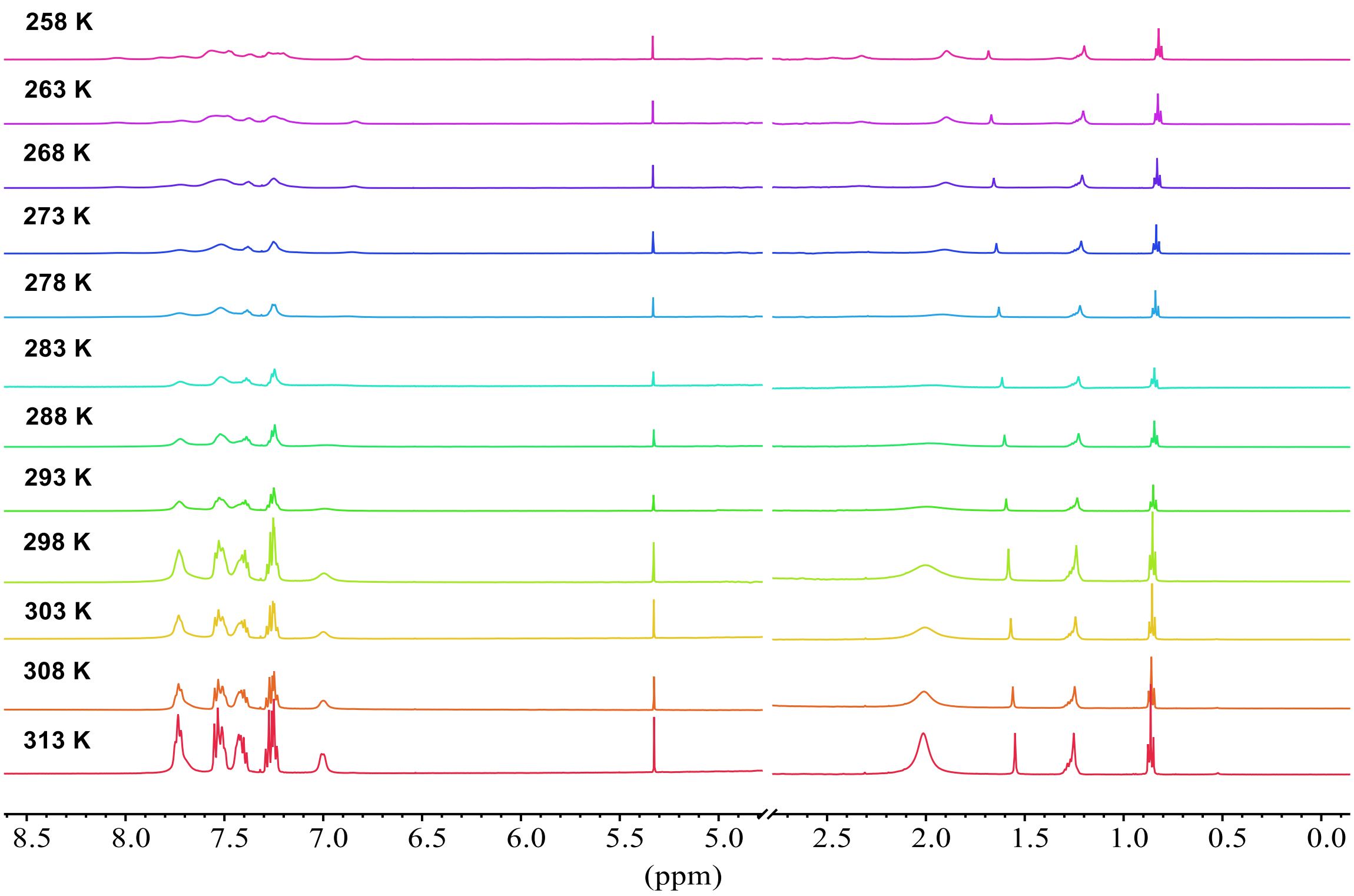

Figure S3: ^{1}H NMR of **3a-PTol₃** at varying temperatures. 1,2-DCE solvent residual peak omitted for clarity.

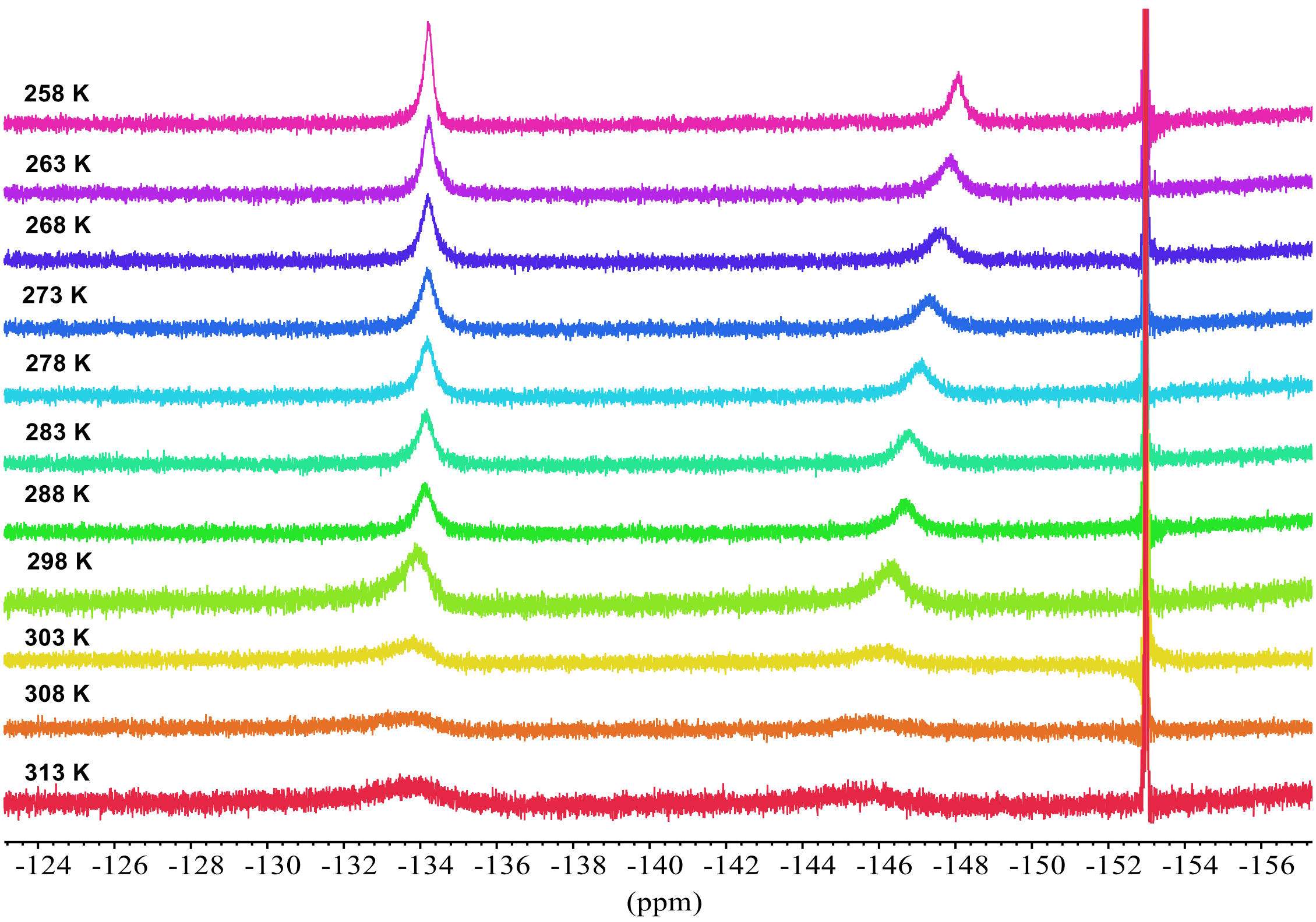

Figure S4: ^{1}H NMR of **3a-PTol$_3$** at varying temperatures. 1,2-DCE solvent residual peak omitted for clarity

NMR Spectra

1a

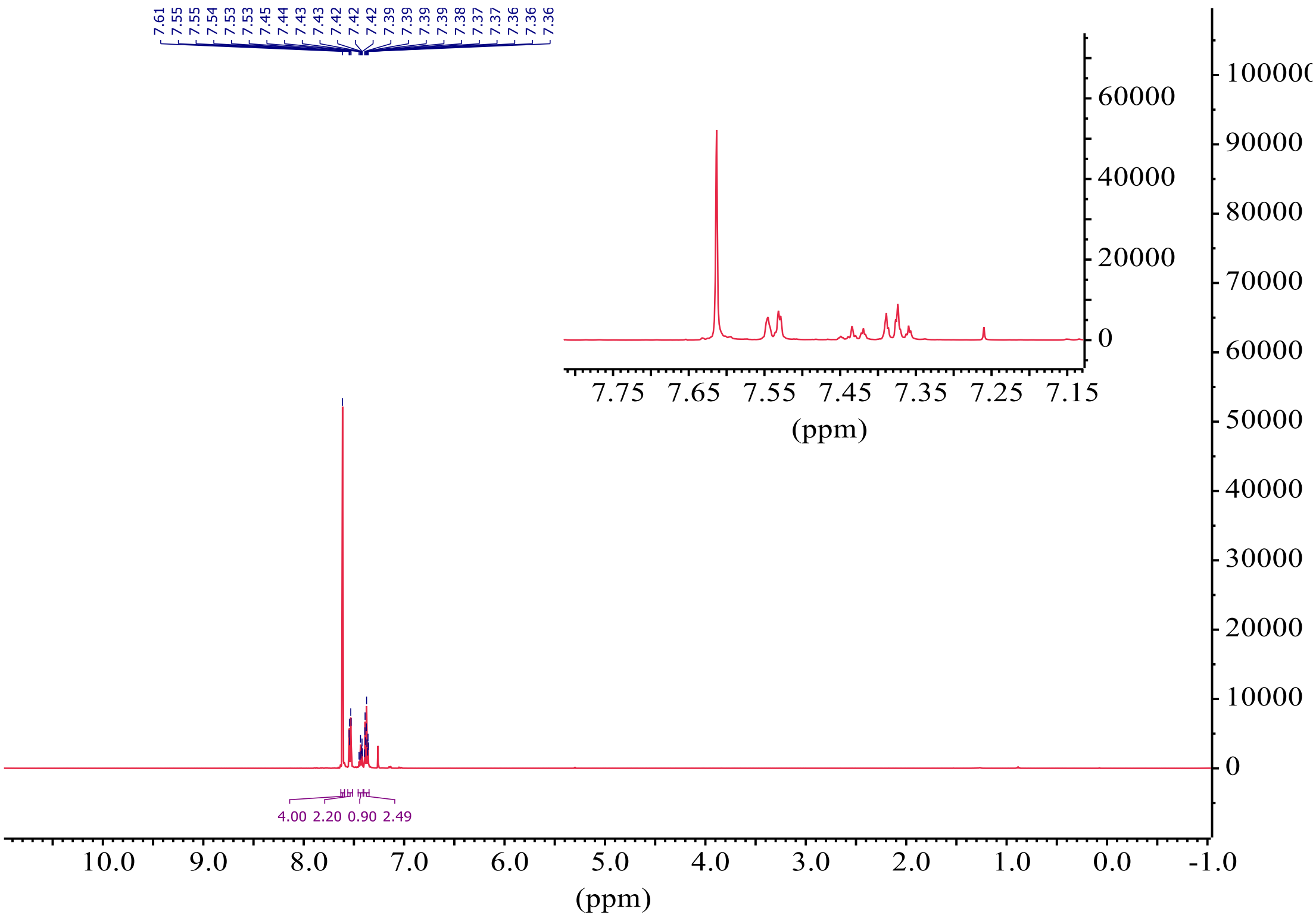

Figure S5: ^{1}H NMR (500 MHz) spectrum of **1a** in $CDCl_3$.

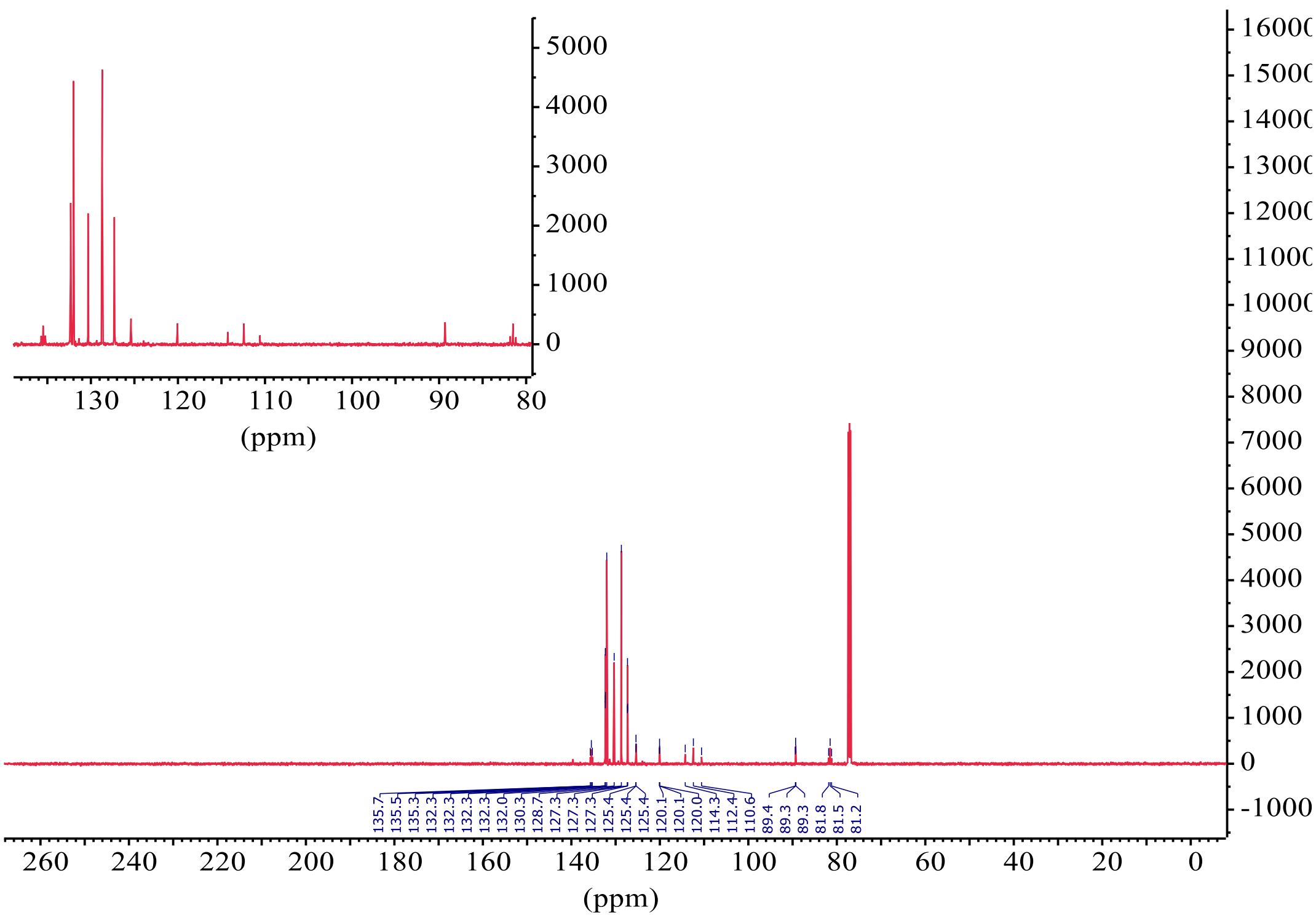

Figure S6: $^{13}C\{^{1}H\}$ NMR (125 MHz) spectrum of **1a** in $CDCl_3$.

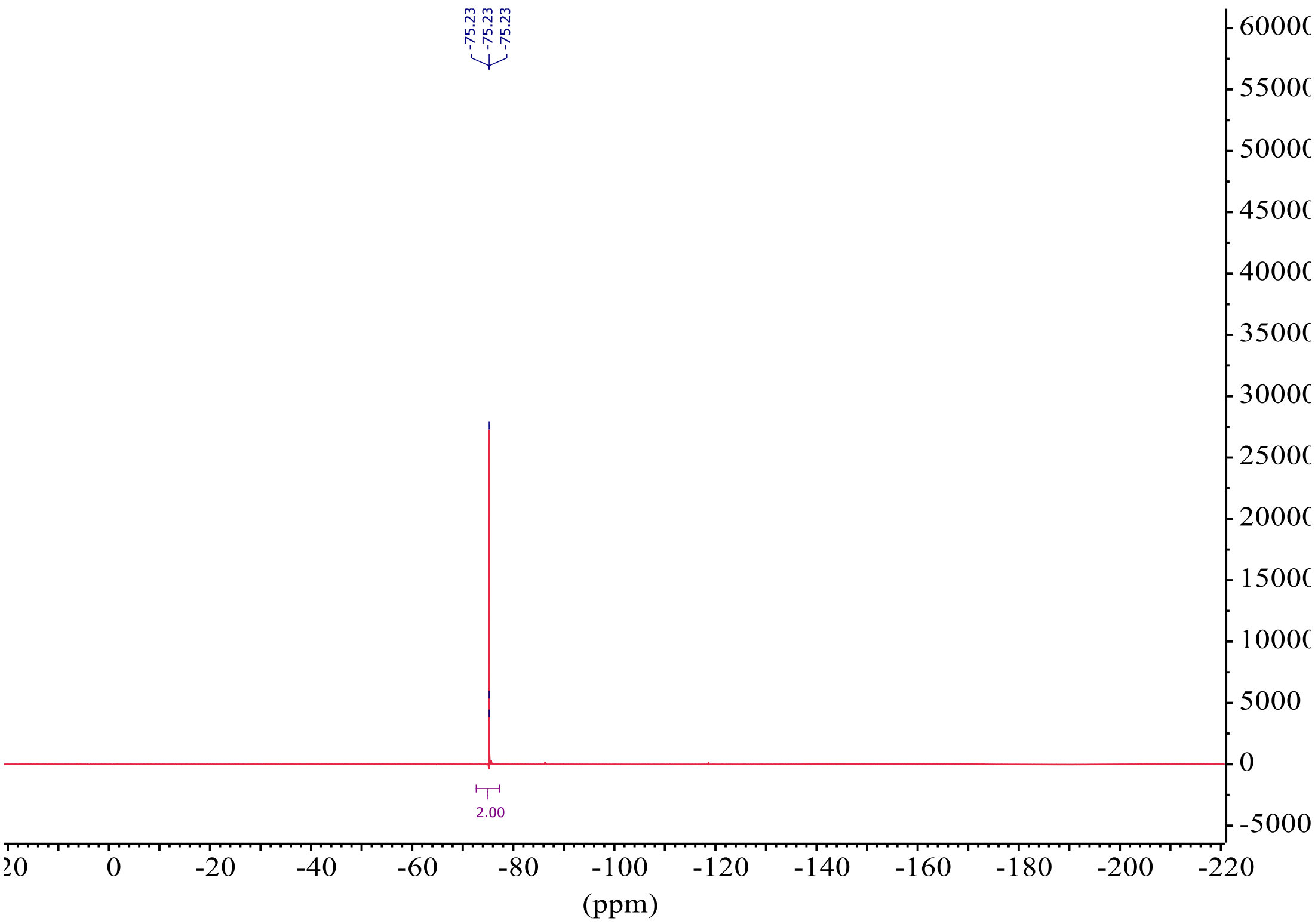

Figure S7: ^{19}F NMR (470 MHz) spectrum of **1a** in $CDCl_3$.

1b

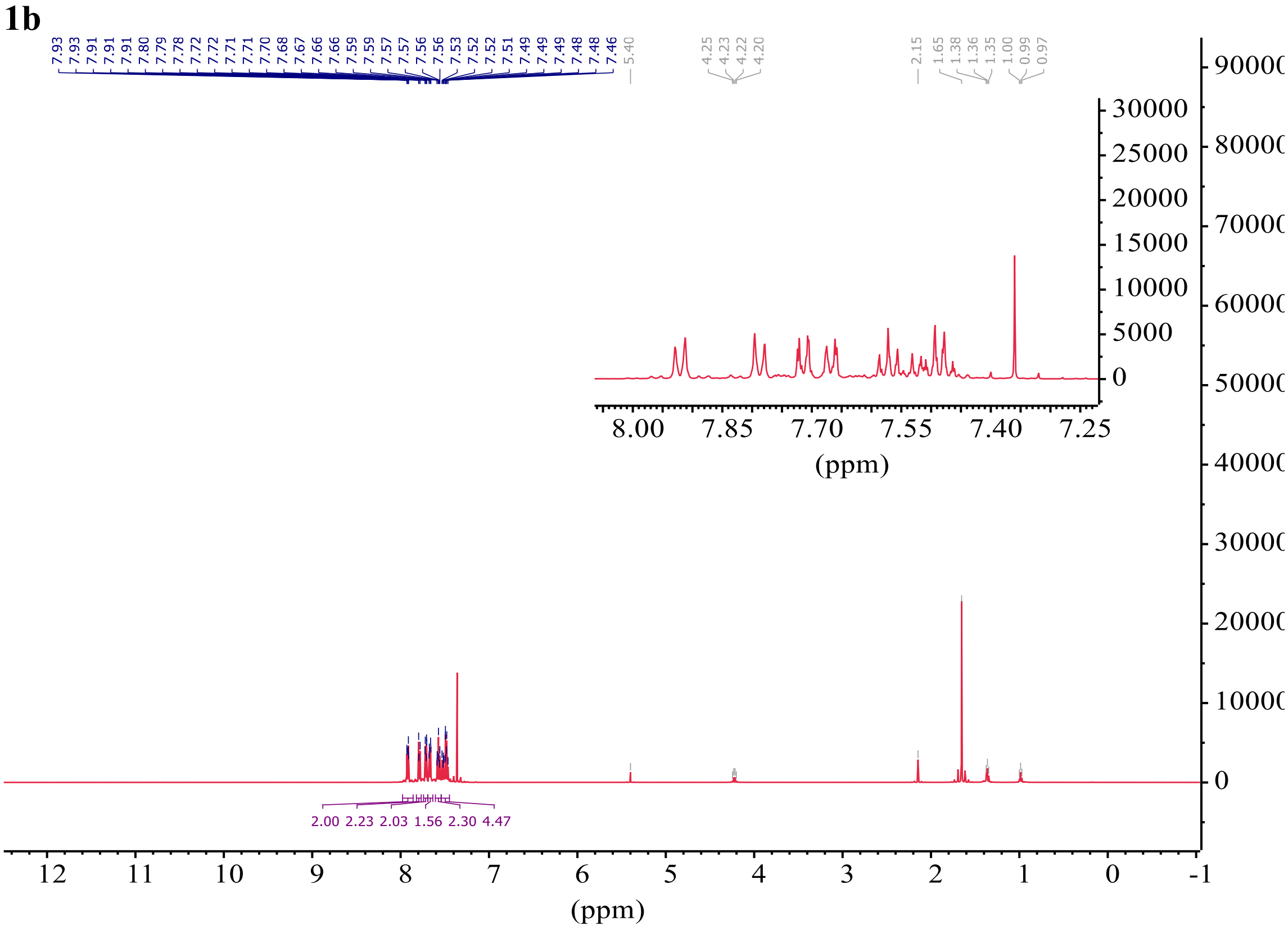

Figure S8: ^{1}H NMR (500 MHz) spectrum of **1b** in $CDCl_3$. Residual solvent impurities are marked with grey ticks.

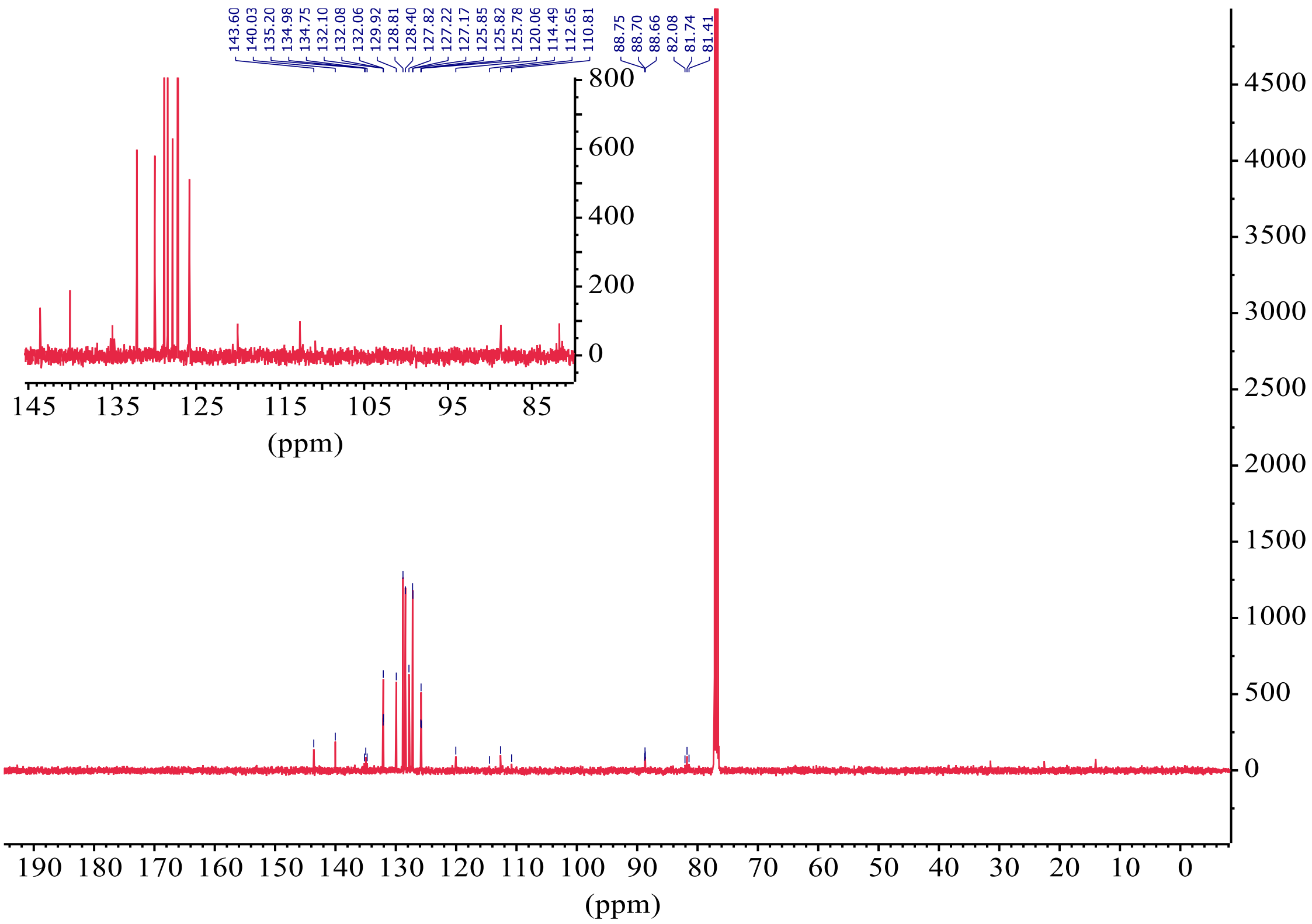

Figure S9: $^{13}C\{^{1}H\}$ NMR (125 MHz) spectrum of **1b** in $CDCl_3$.

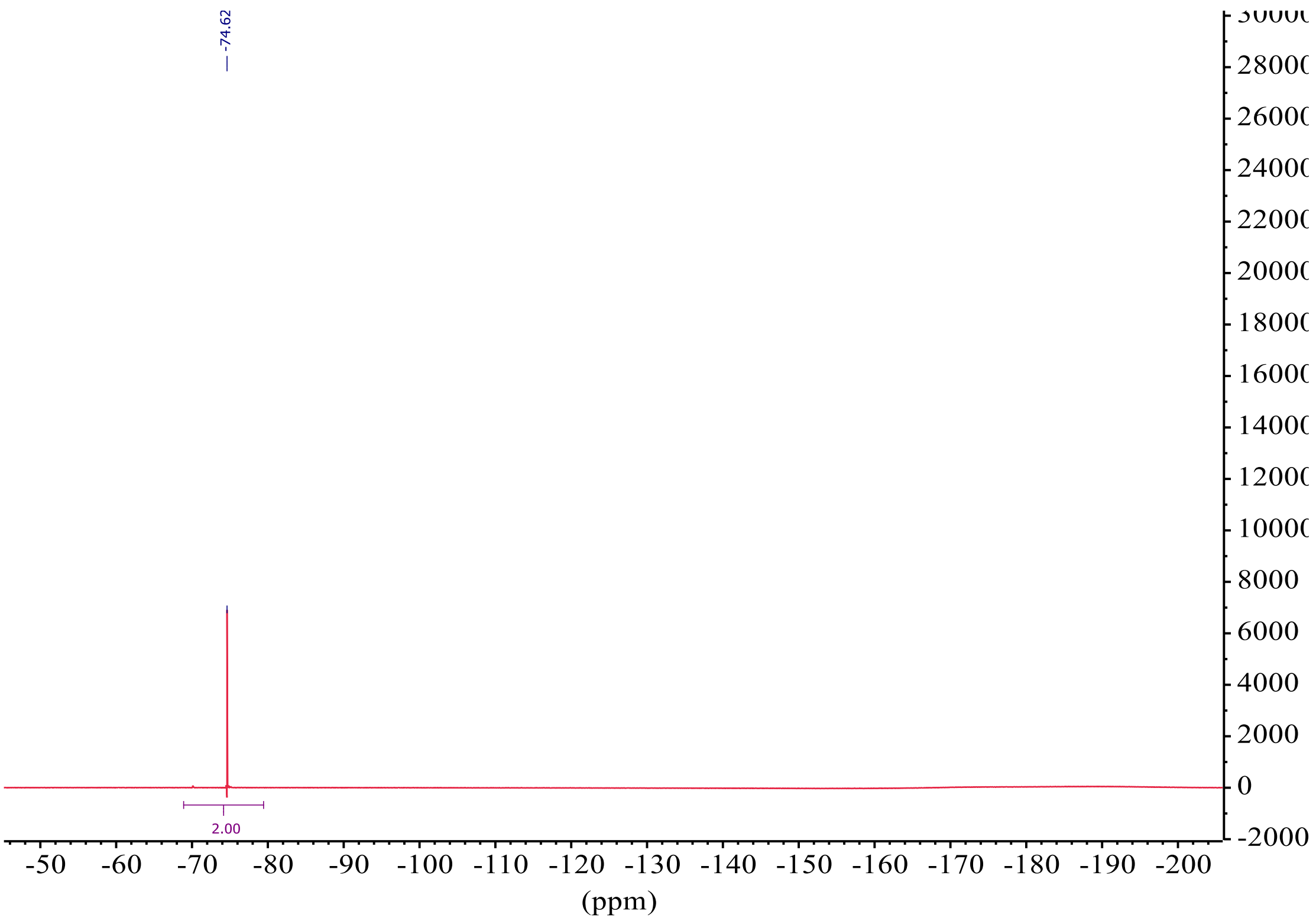

Figure S10: ^{19}F NMR (470 MHz) spectrum of **1b** in $CDCl_3$.

1c

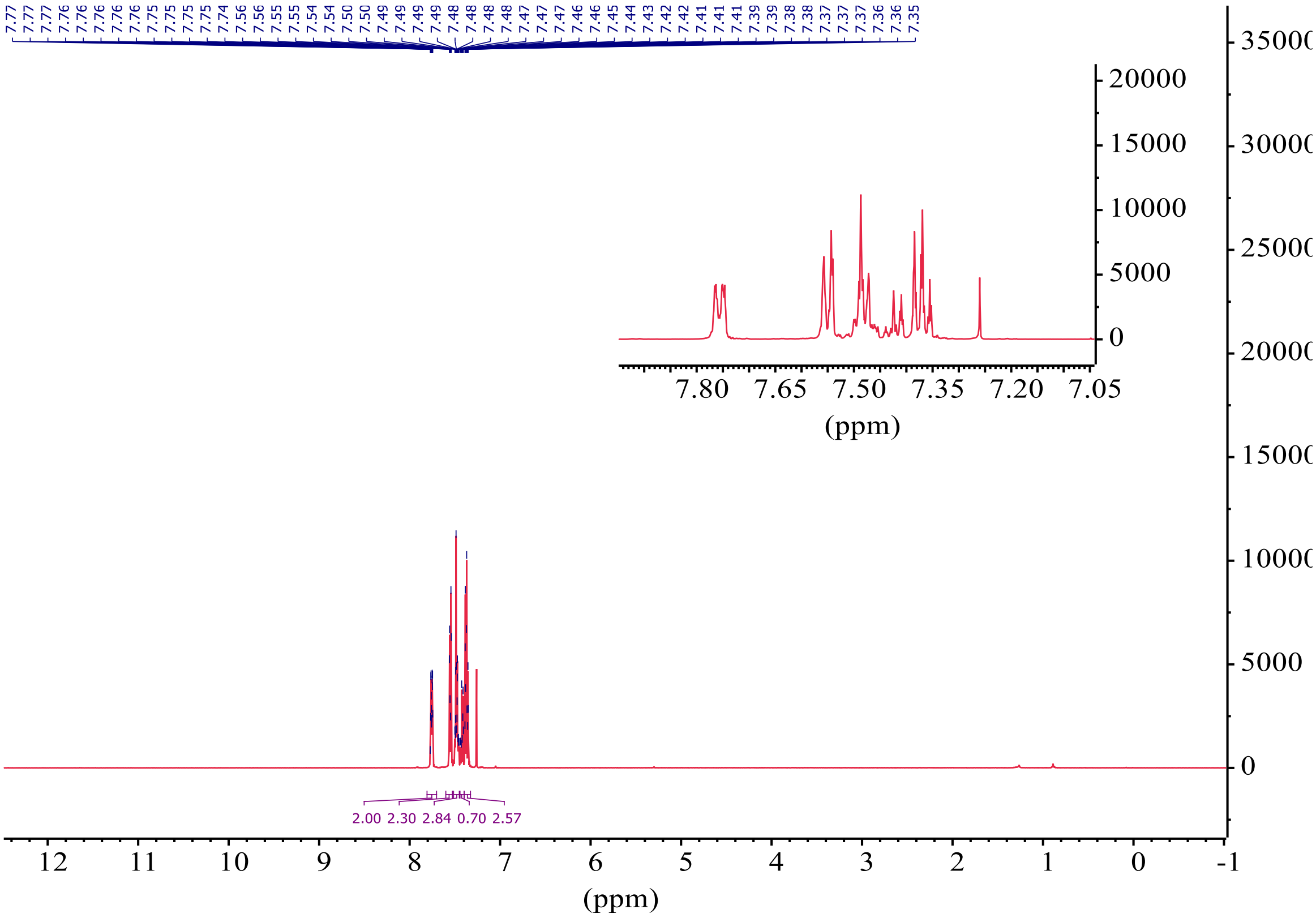

Figure S11: ^{1}H NMR (500 MHz) spectrum of **1c** in $CDCl_3$.

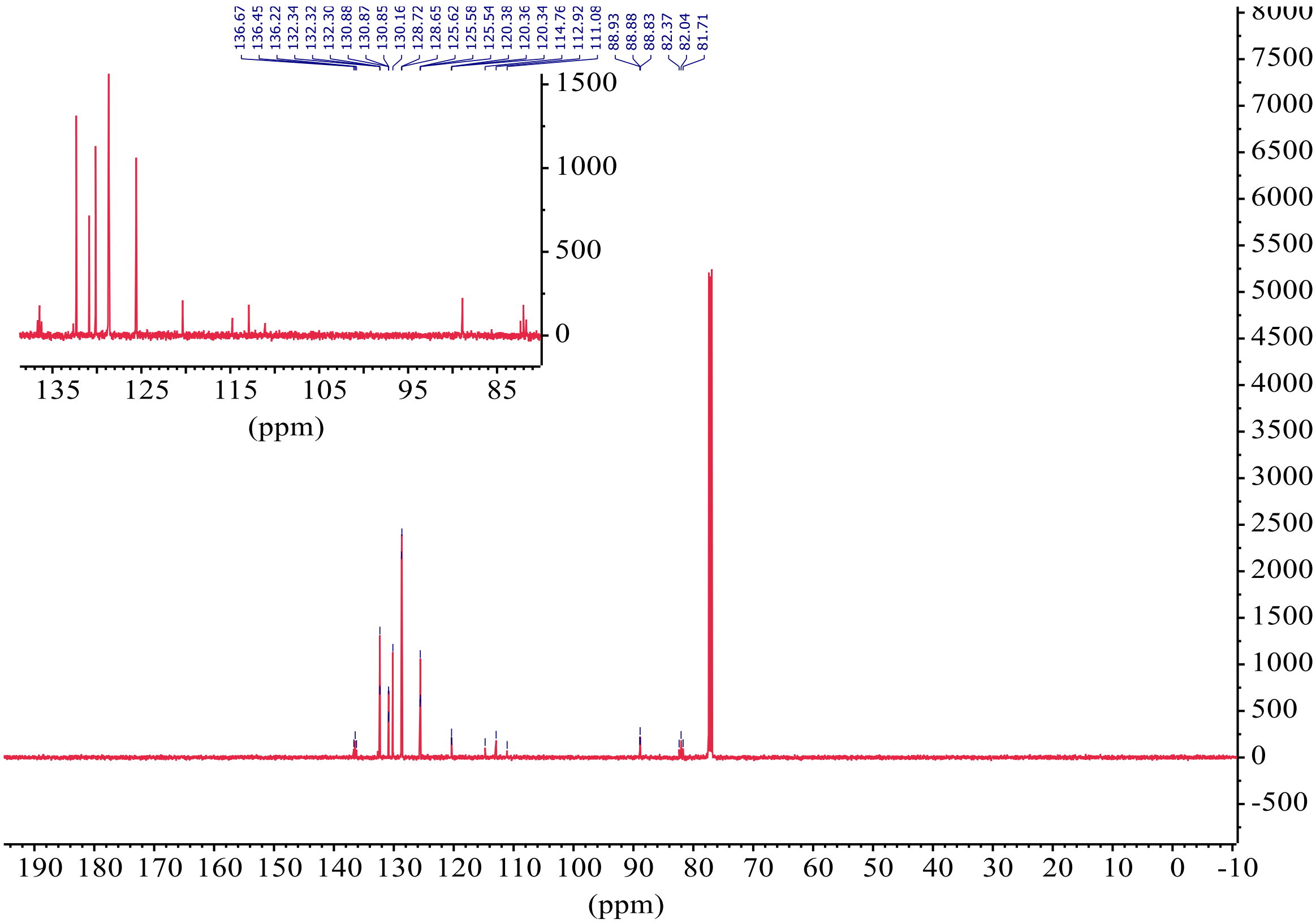

Figure S12: $^{13}C\{^{1}H\}$ NMR (125 MHz) spectrum of **1c** in $CDCl_3$.

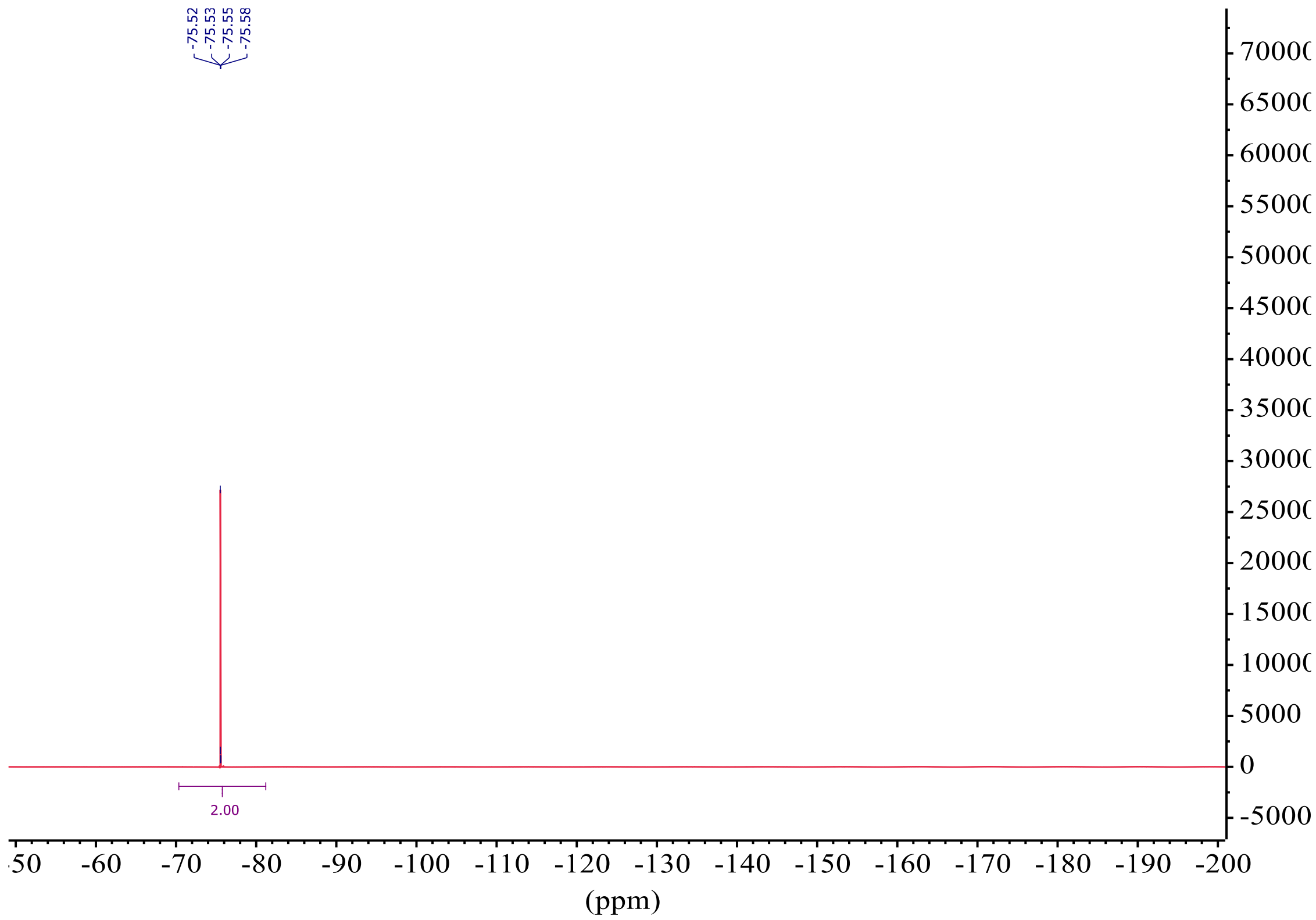

Figure S13: ^{19}F NMR (470 MHz) spectrum of **1c** in $CDCl_3$.

1d

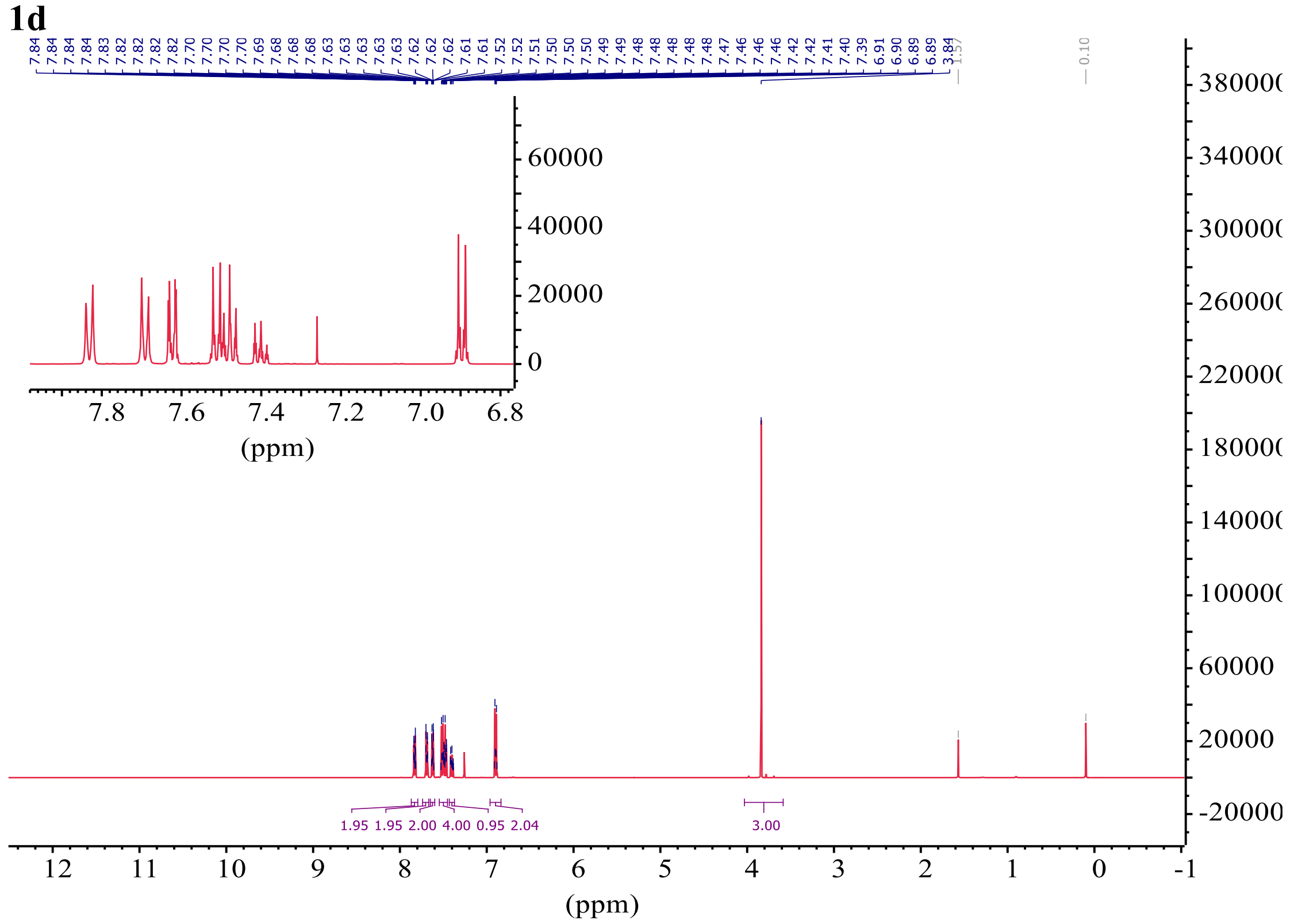

Figure S14: ^{1}H NMR (500 MHz) spectrum of **1d** in $CDCl_3$. Residual solvent impurities are marked with grey ticks.

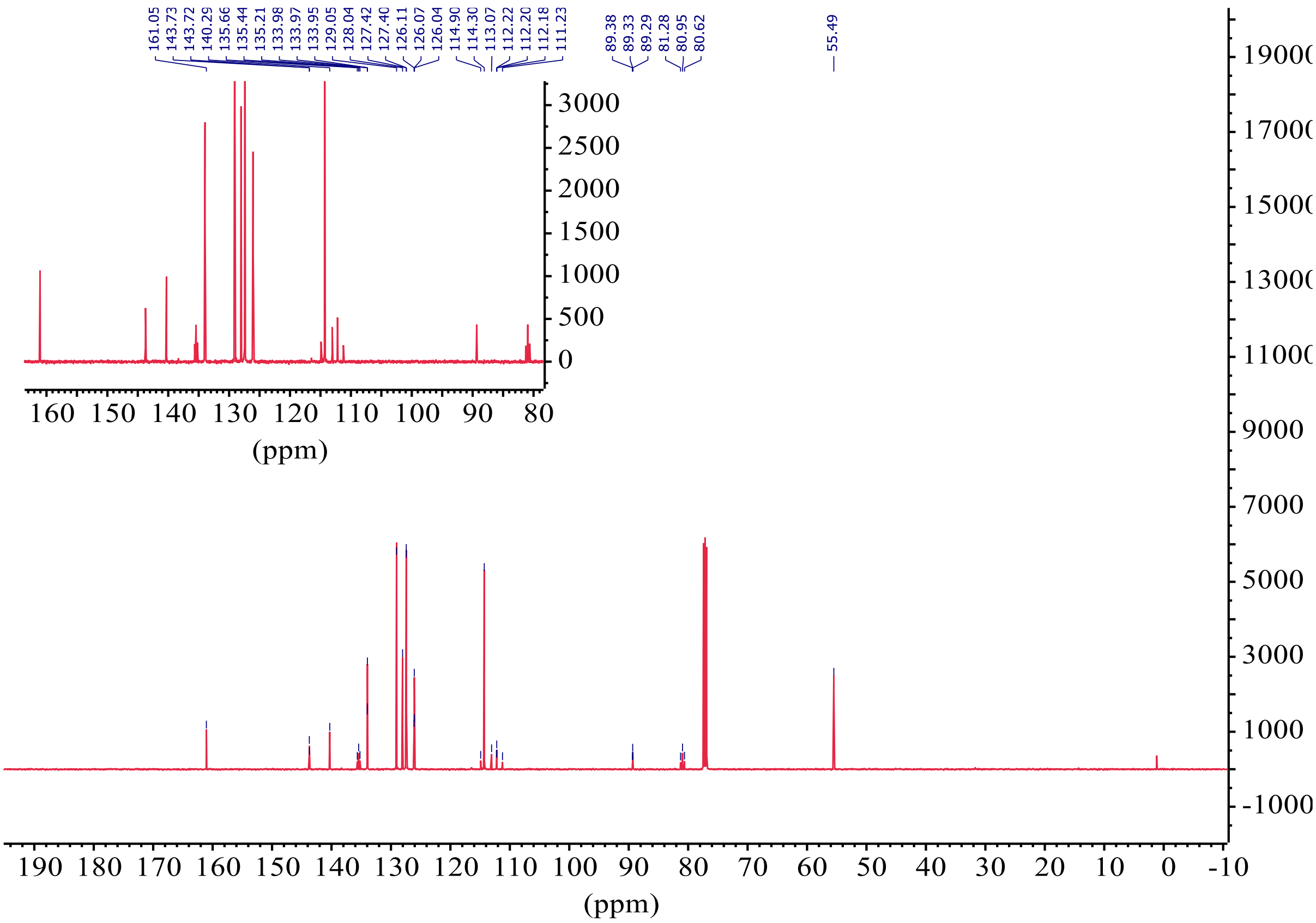

Figure S15: $^{13}C\{^{1}H\}$ NMR (125 MHz) spectrum of **1d** in $CDCl_3$.

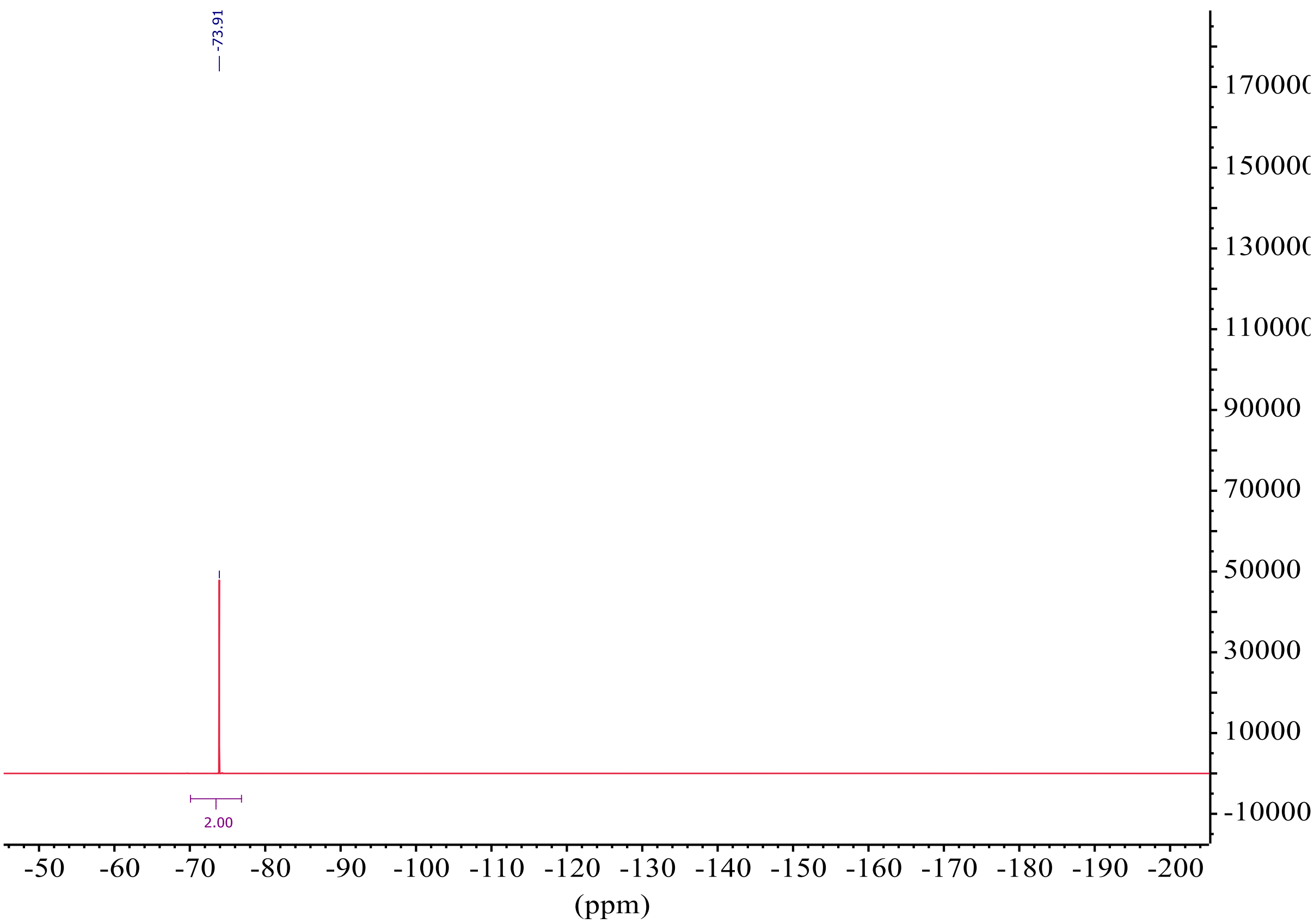

Figure S16: ^{19}F NMR (470 MHz) spectrum of **1d** in $CDCl_3$

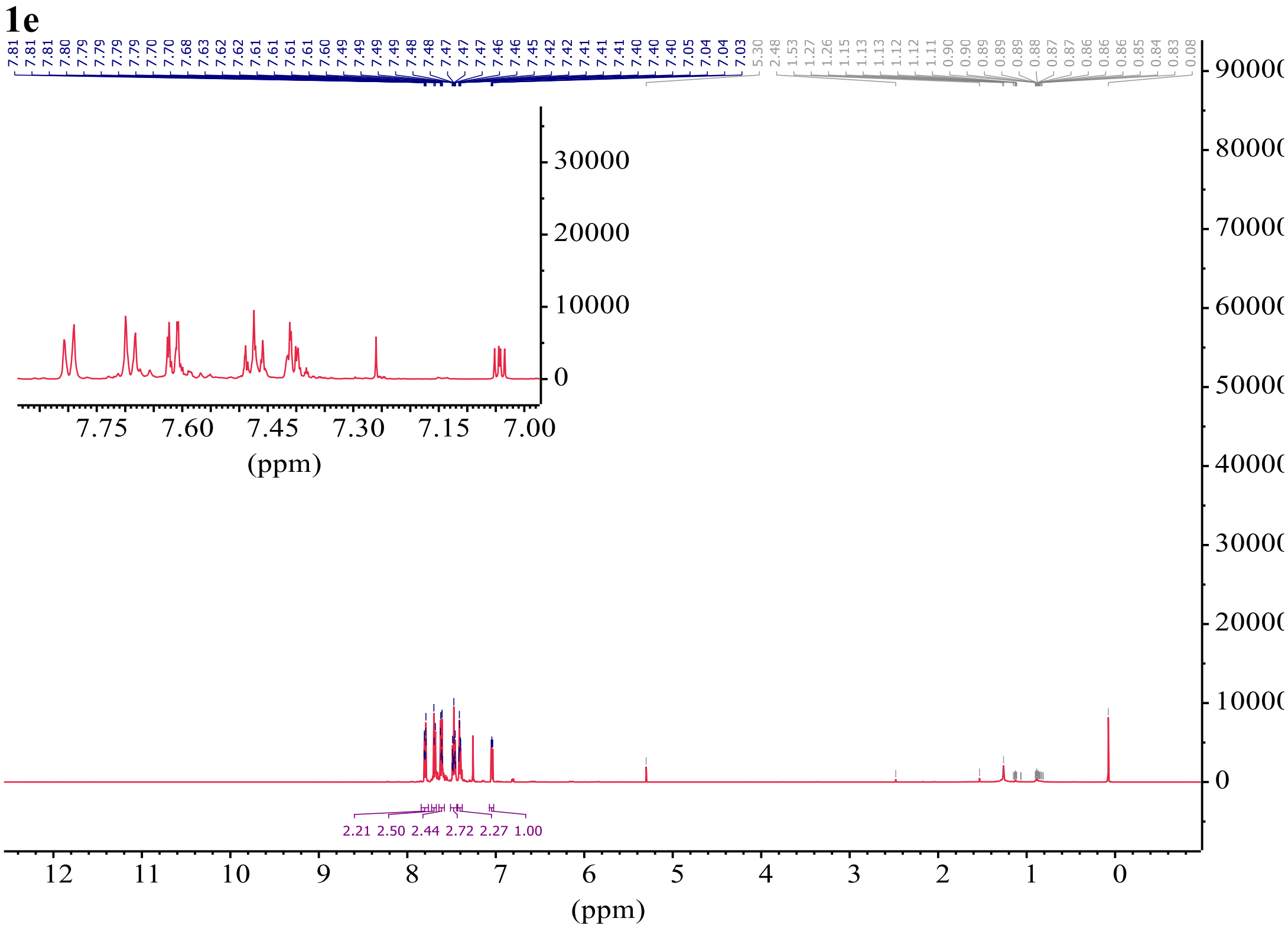

Figure S17: ^{1}H NMR (500 MHz) spectrum of **1e** in $CDCl_3$. Residual solvent impurities are marked with grey ticks.

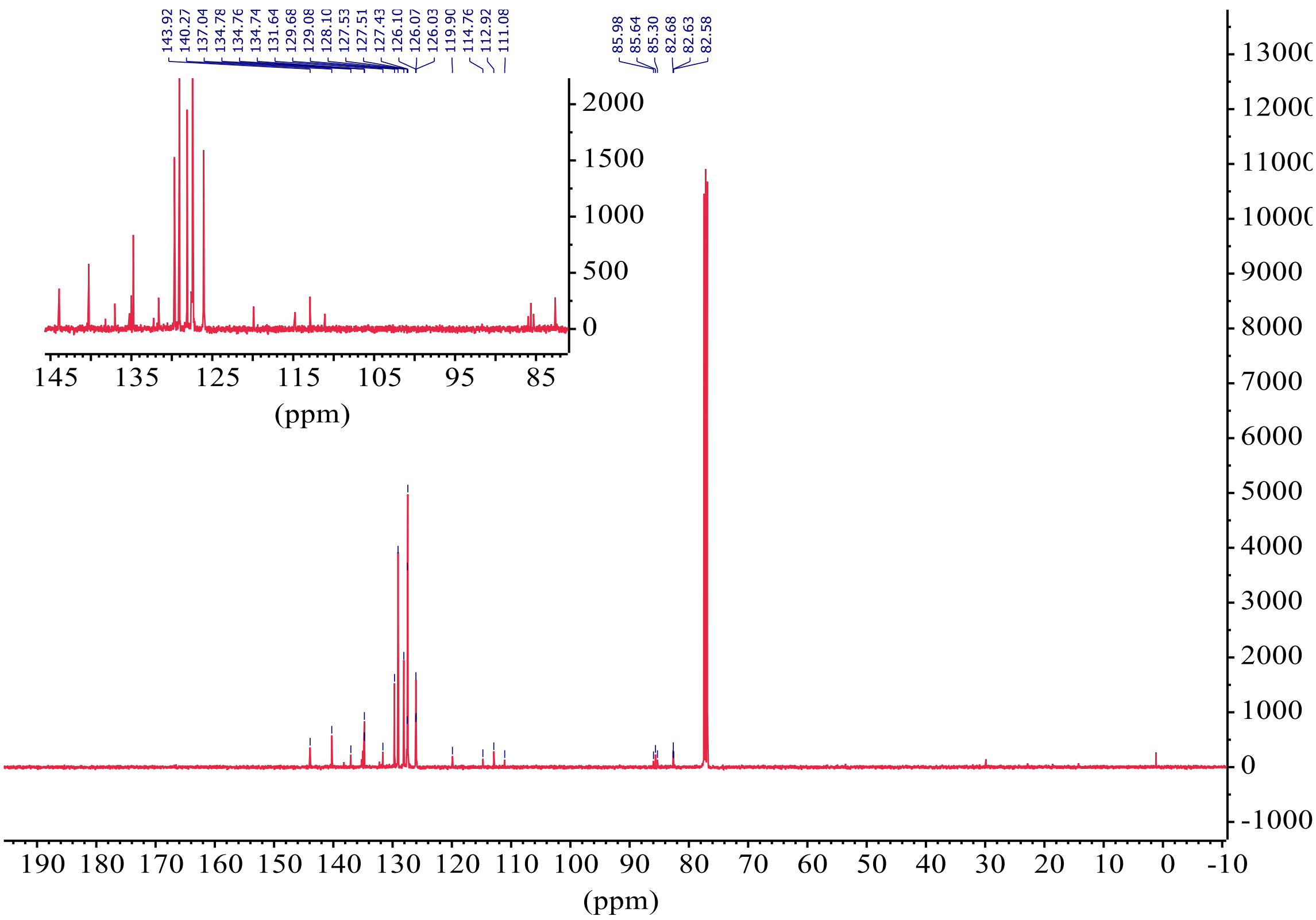

Figure S18: $^{13}C\{^{1}H\}$ NMR (125 MHz) spectrum of **1e** in $CDCl_3$.

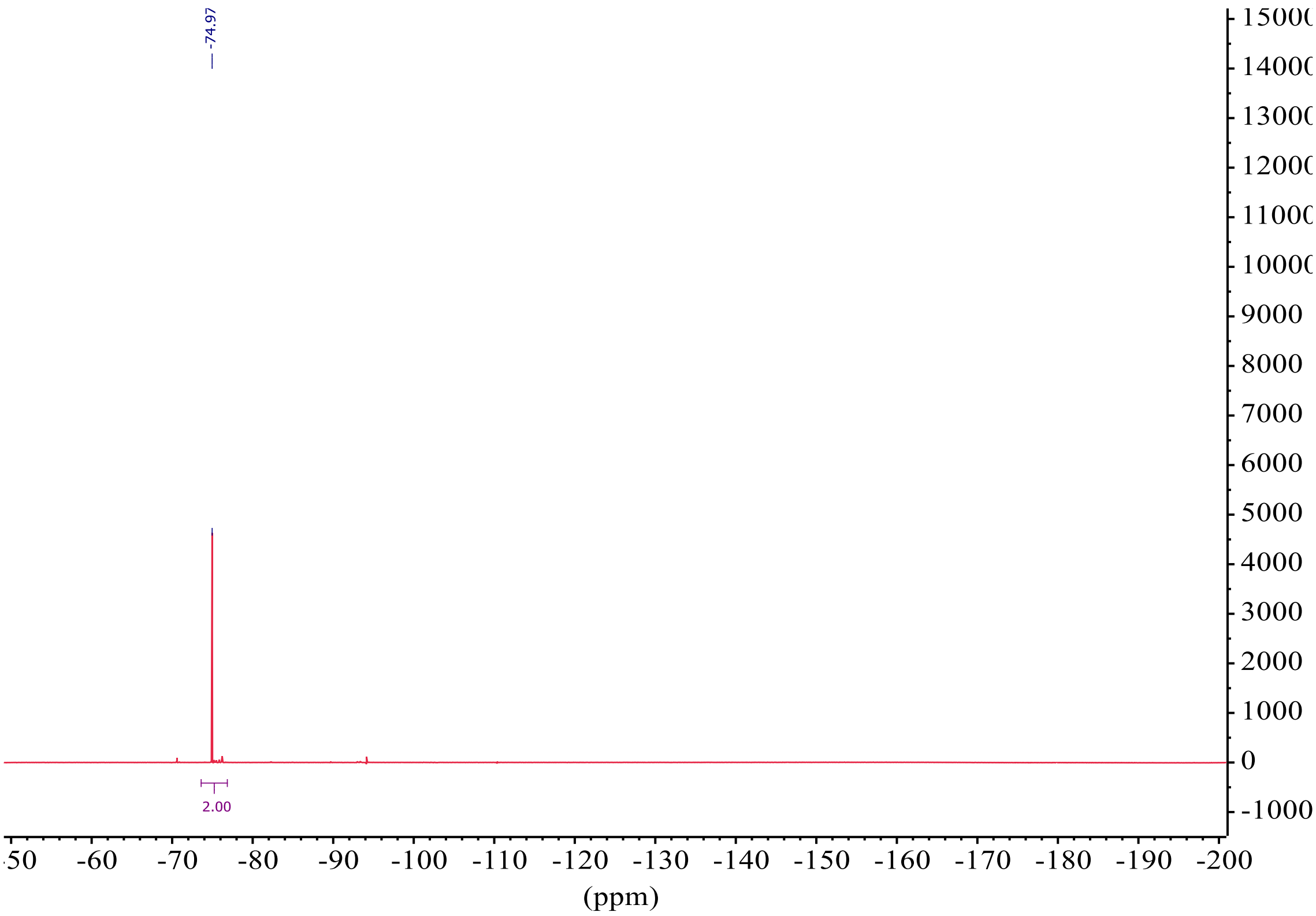
Figure S19: ^{19}F NMR (470 MHz) spectrum of **1e** in $CDCl_3$

1f

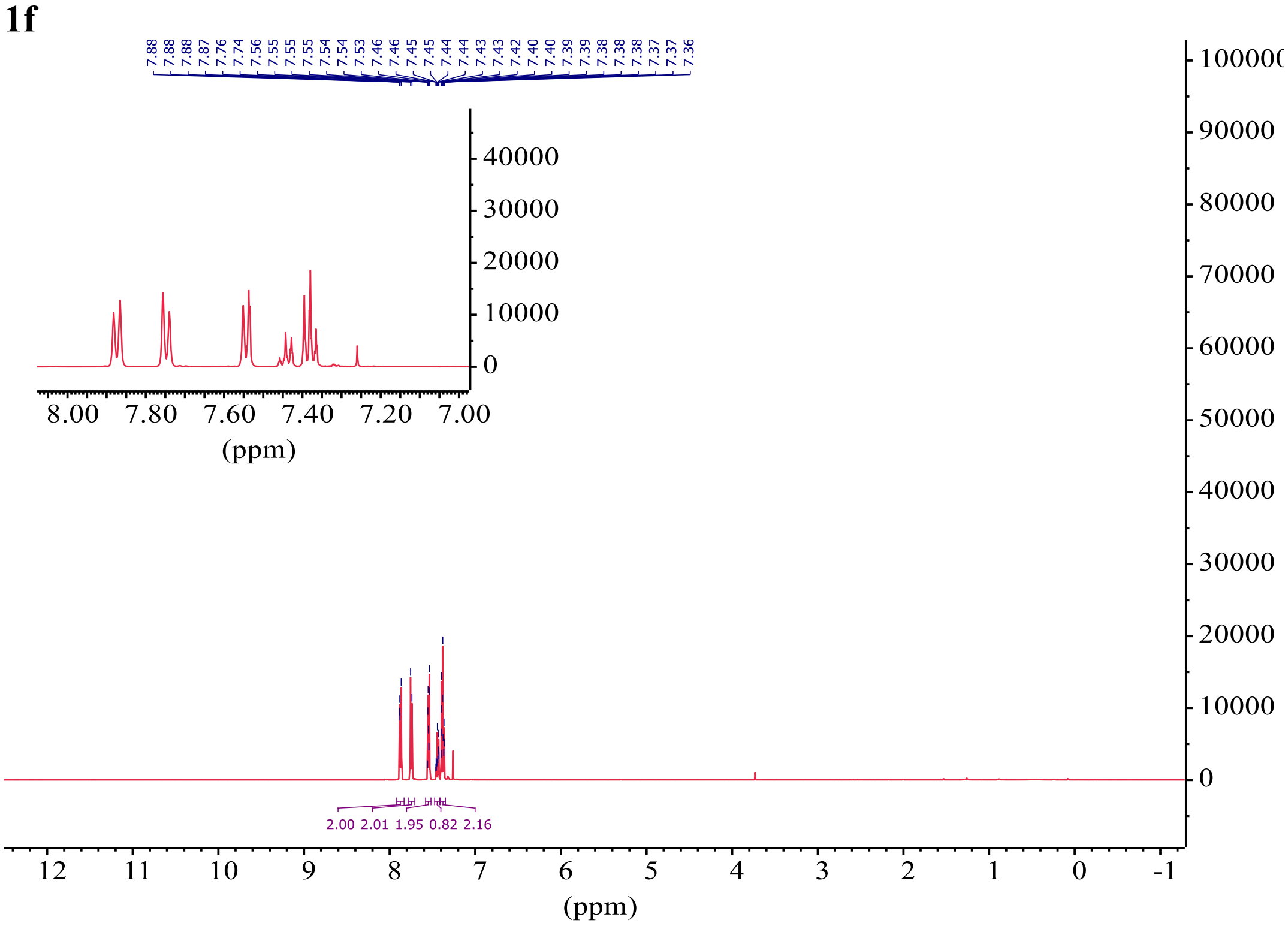
Figure S20: ^{1}H NMR (500 MHz) spectrum of **1f** in $CDCl_3$.

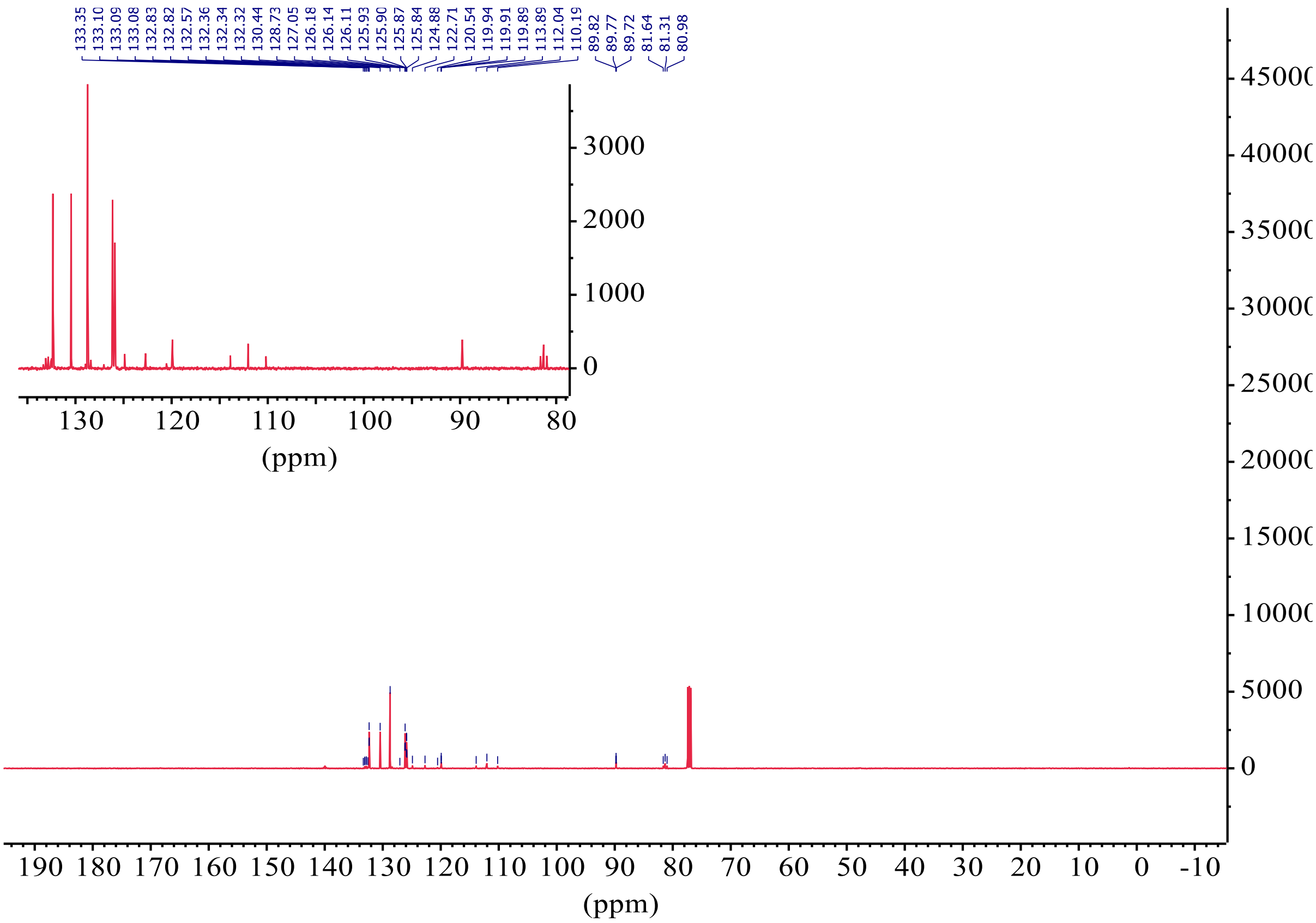

Figure S21: $^{13}C\{^{1}H\}$ NMR (125 MHz) spectrum of **1f** in $CDCl_3$.

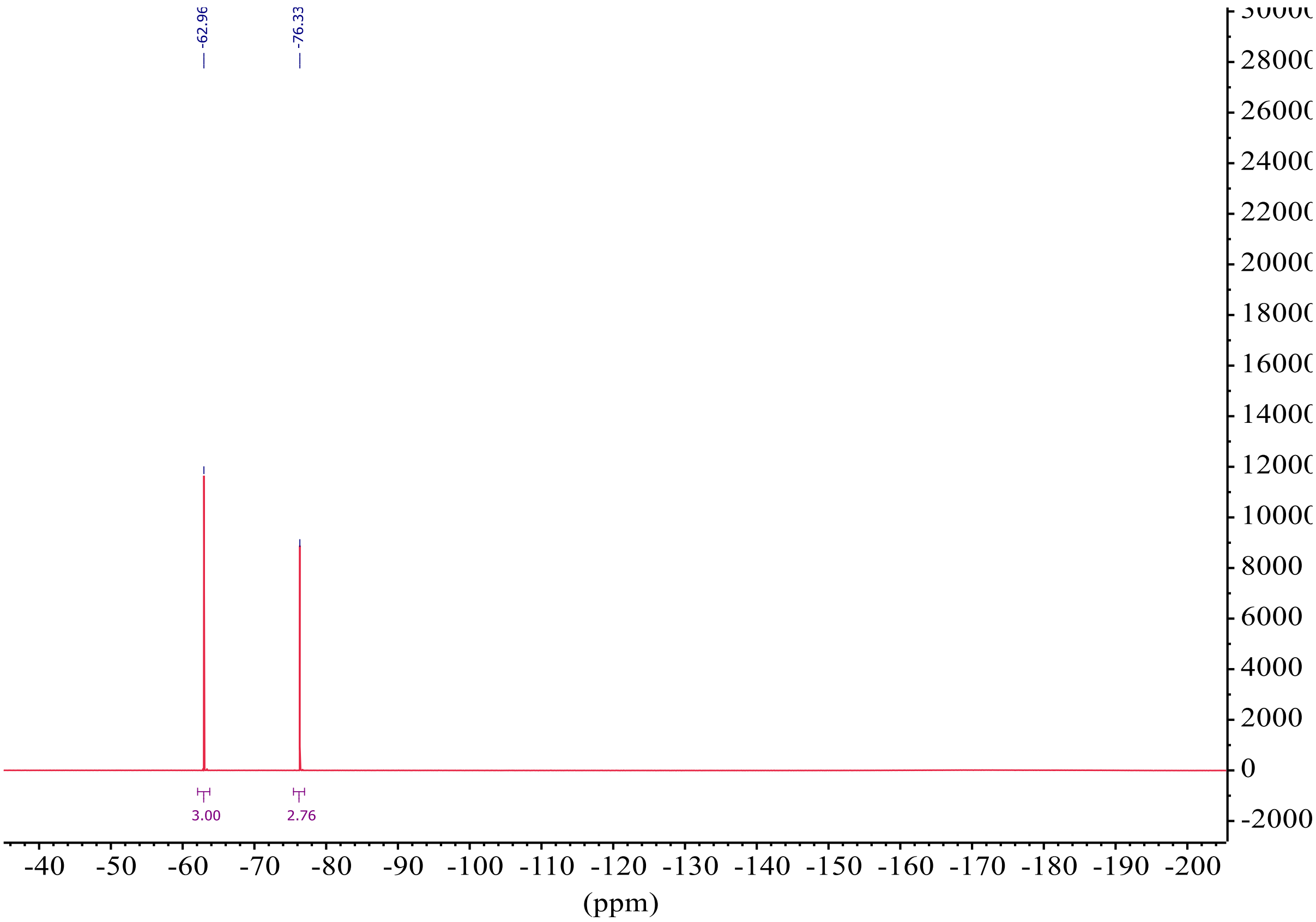

Figure S22: ^{19}F NMR (470 MHz) spectrum of **1f** in $CDCl_3$

1g-TIPS

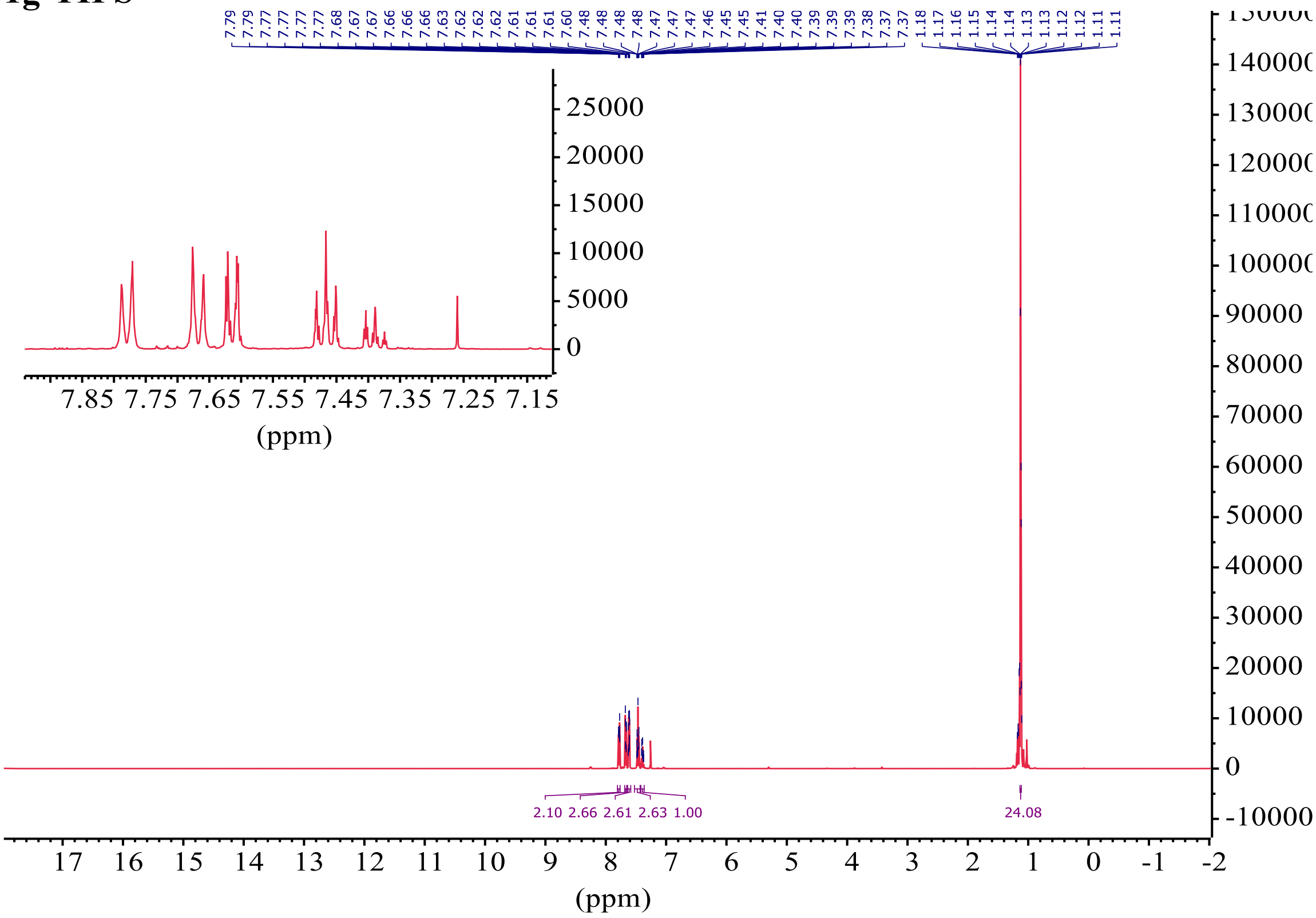

Figure S23: ^{1}H NMR (500 MHz) spectrum of **1g-TIPS** in $CDCl_3$.

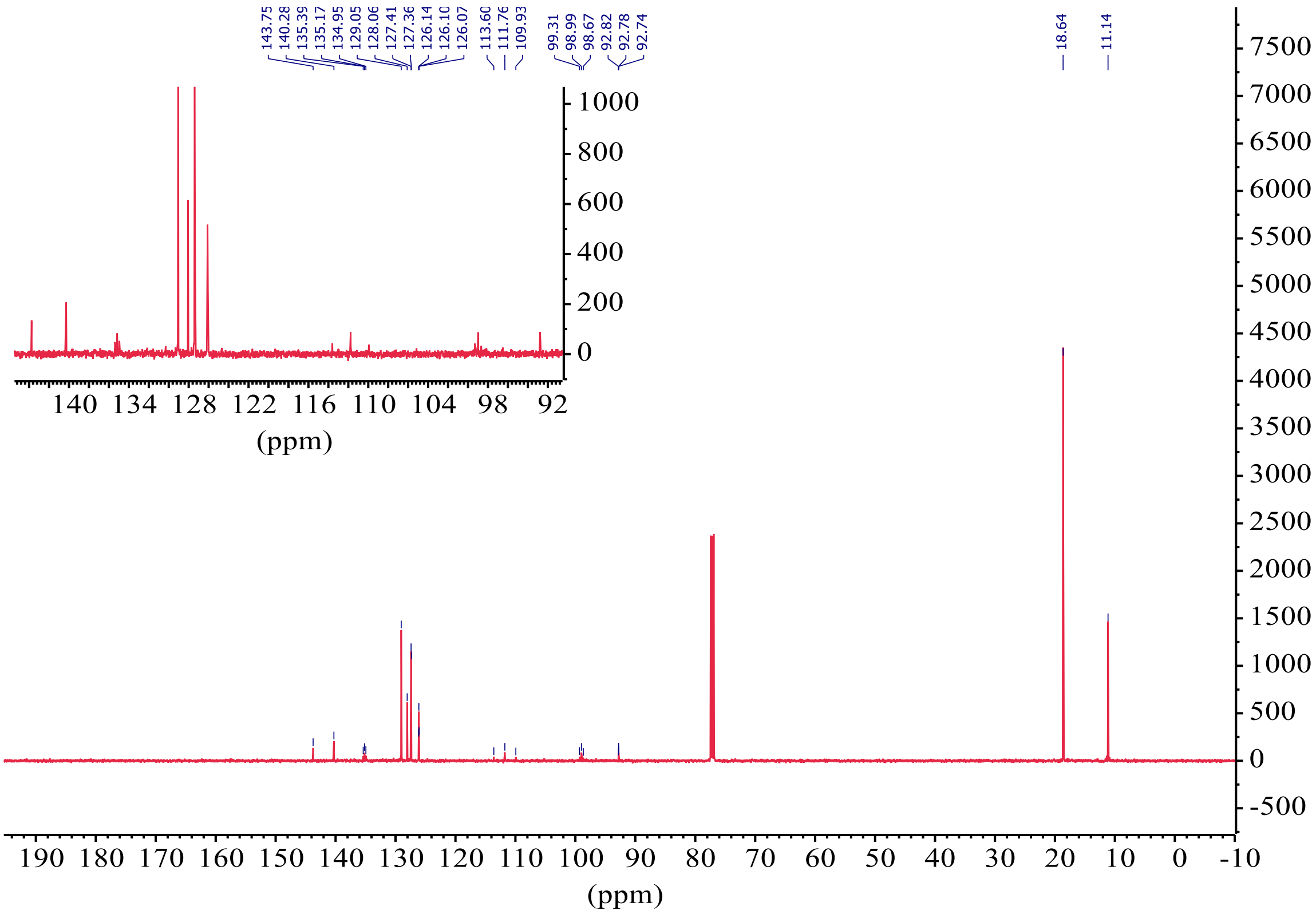

Figure S24: $^{13}C\{^{1}H\}$ NMR (125 MHz) spectrum of **1g-TIPS** in $CDCl_3$.

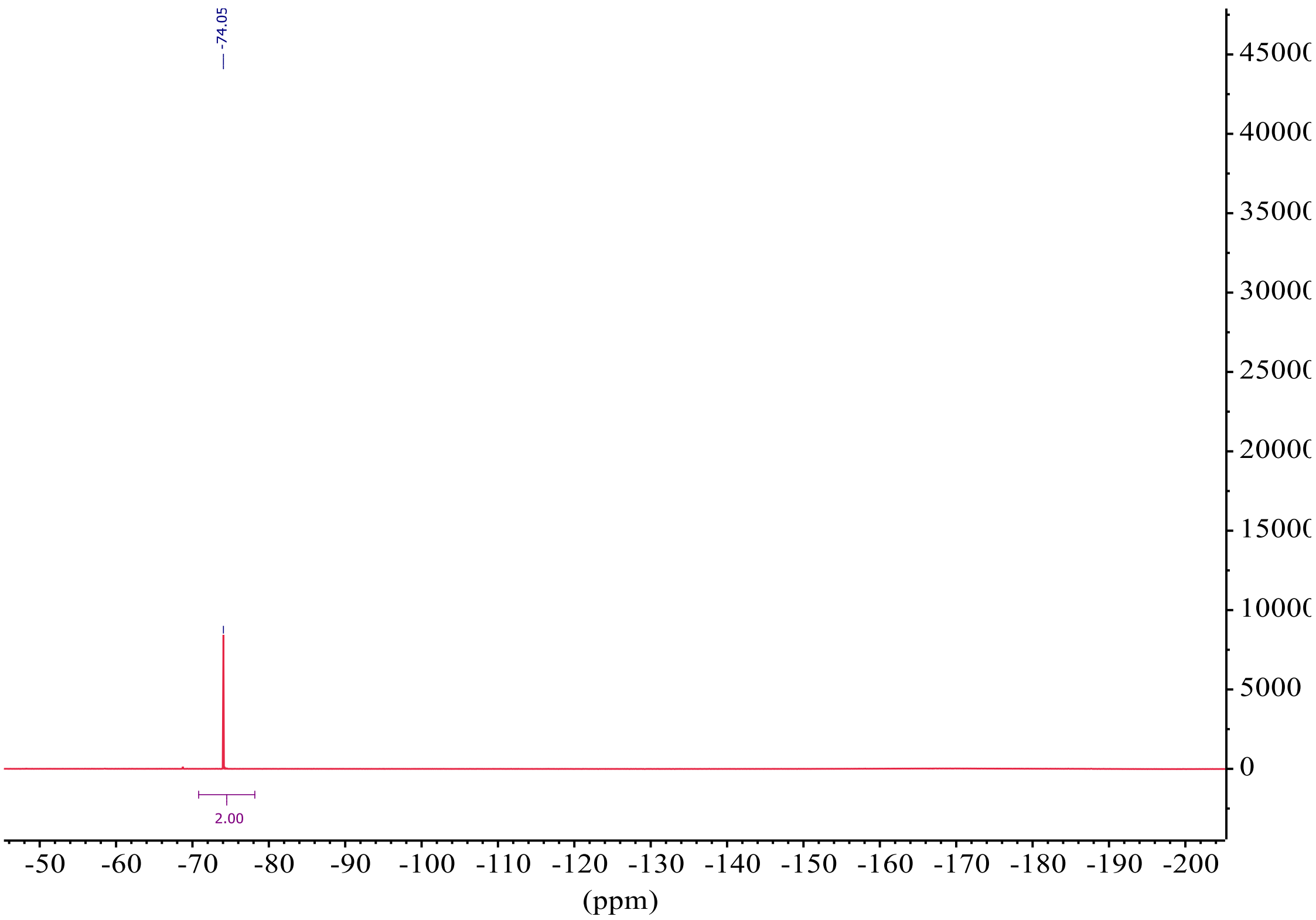

Figure S25: ^{19}F NMR (470 MHz) spectrum of **1g-TIPS** in $CDCl_3$

1g

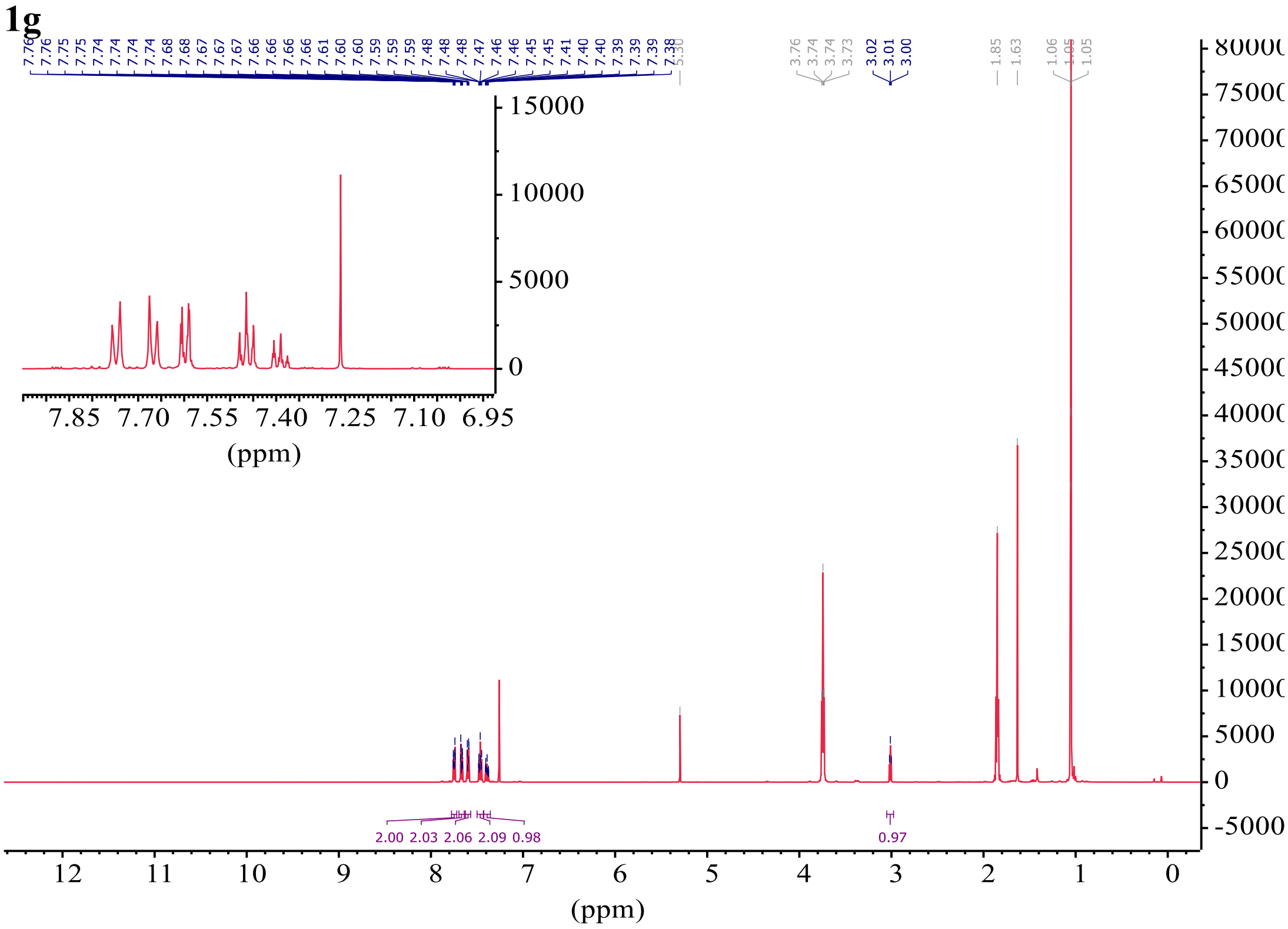

Figure S26: ^{1}H NMR (500 MHz) spectrum of **1g** in $CDCl_3$. Residual solvent impurities are marked with grey ticks.

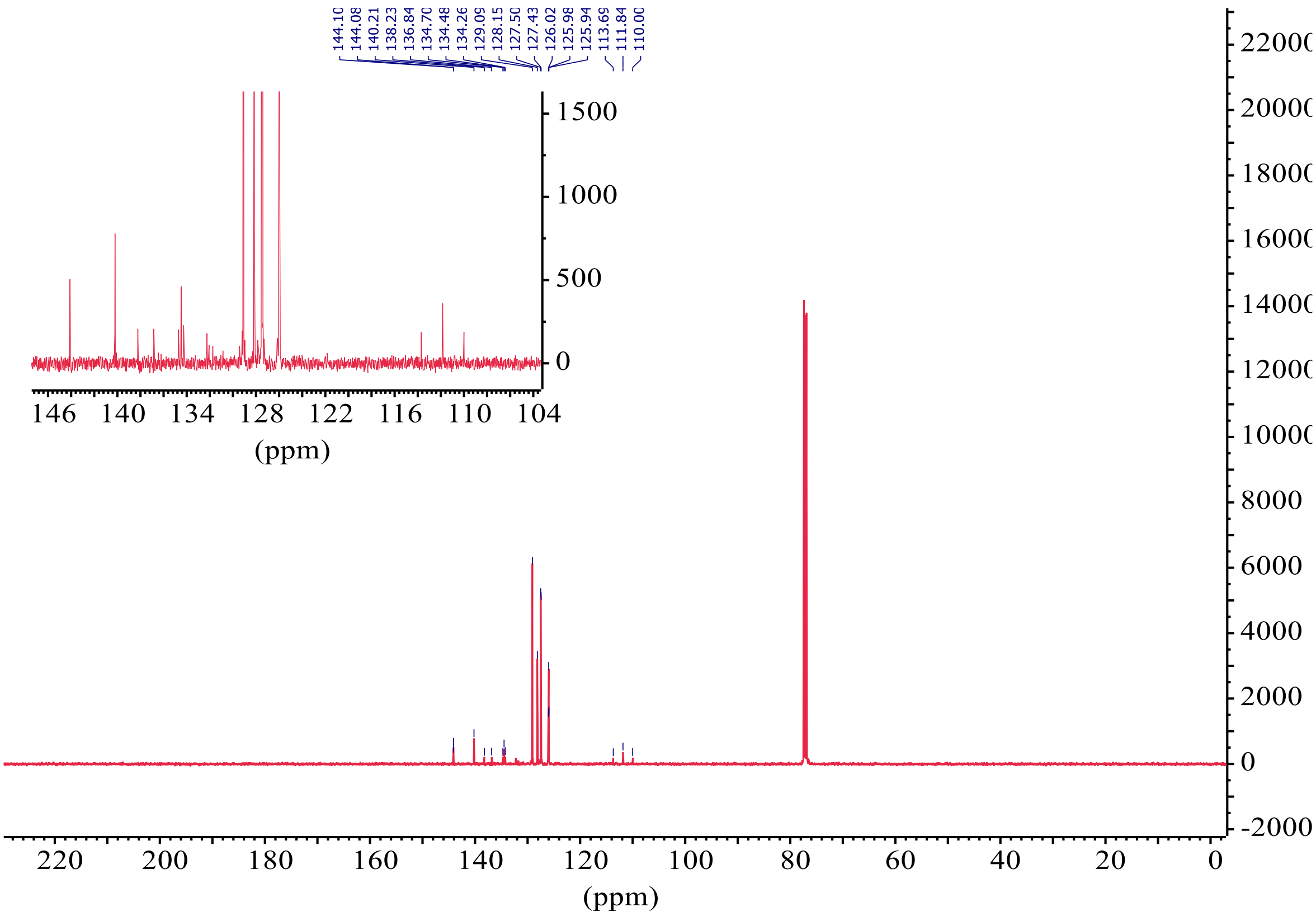

Figure S27: $^{13}C\{^1H\}$ NMR (125 MHz) spectrum of **1g** in $CDCl_3$

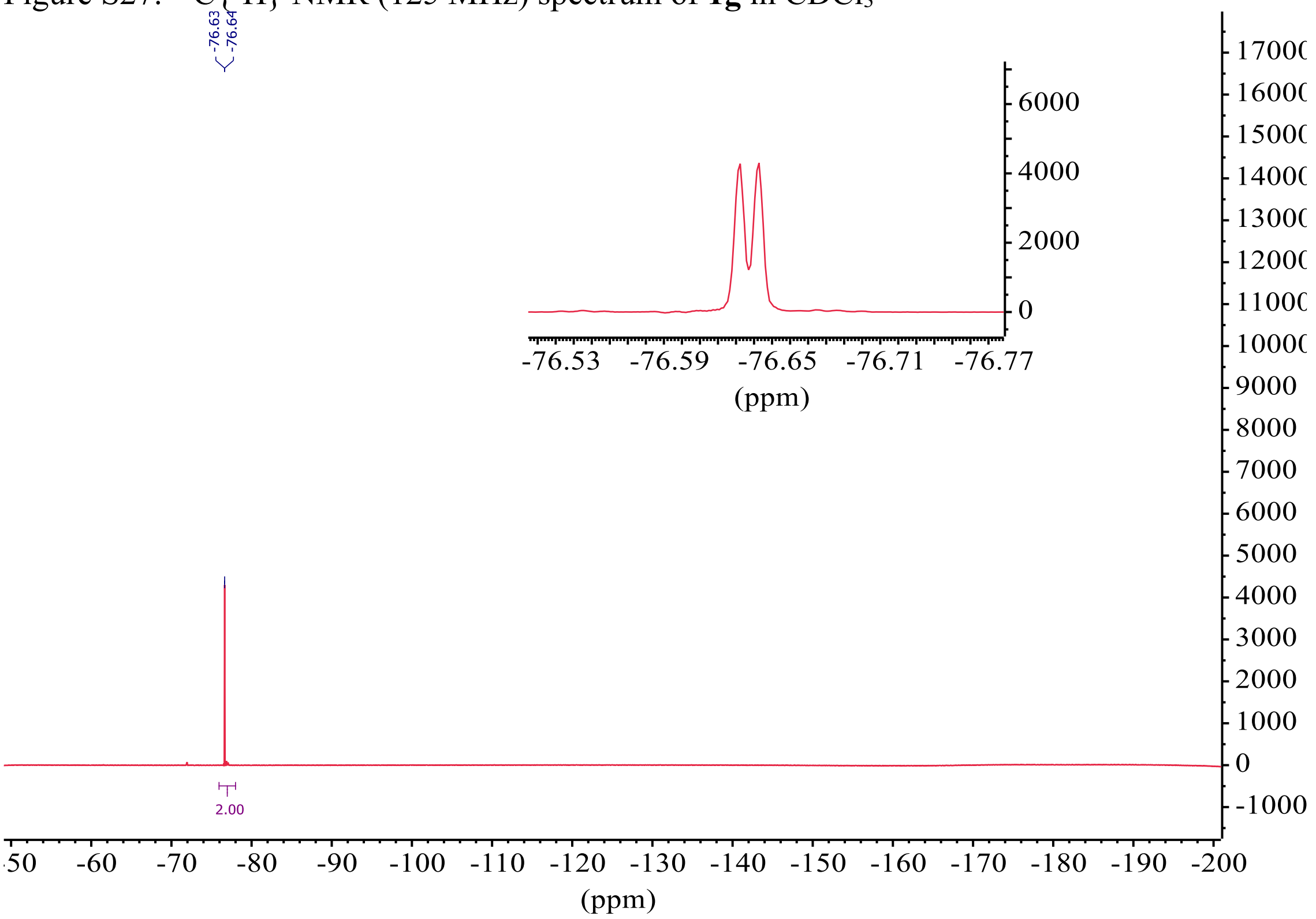

Figure S28: ^{19}F NMR (470 MHz) spectrum of **1g** in $CDCl_3$.

1h

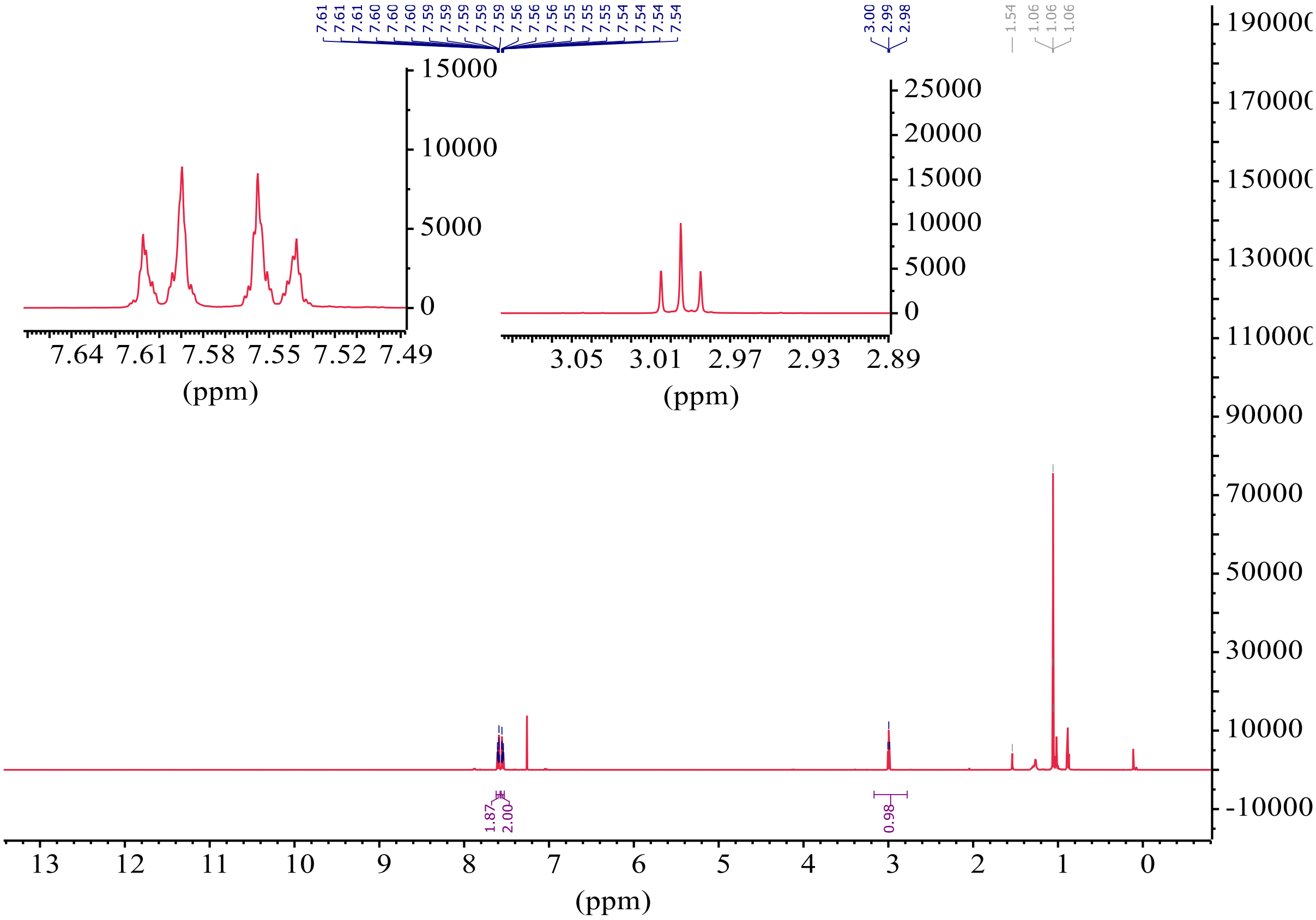

Figure S29: ^{1}H NMR (500 MHz) spectrum of **1h** in $CDCl_3$.

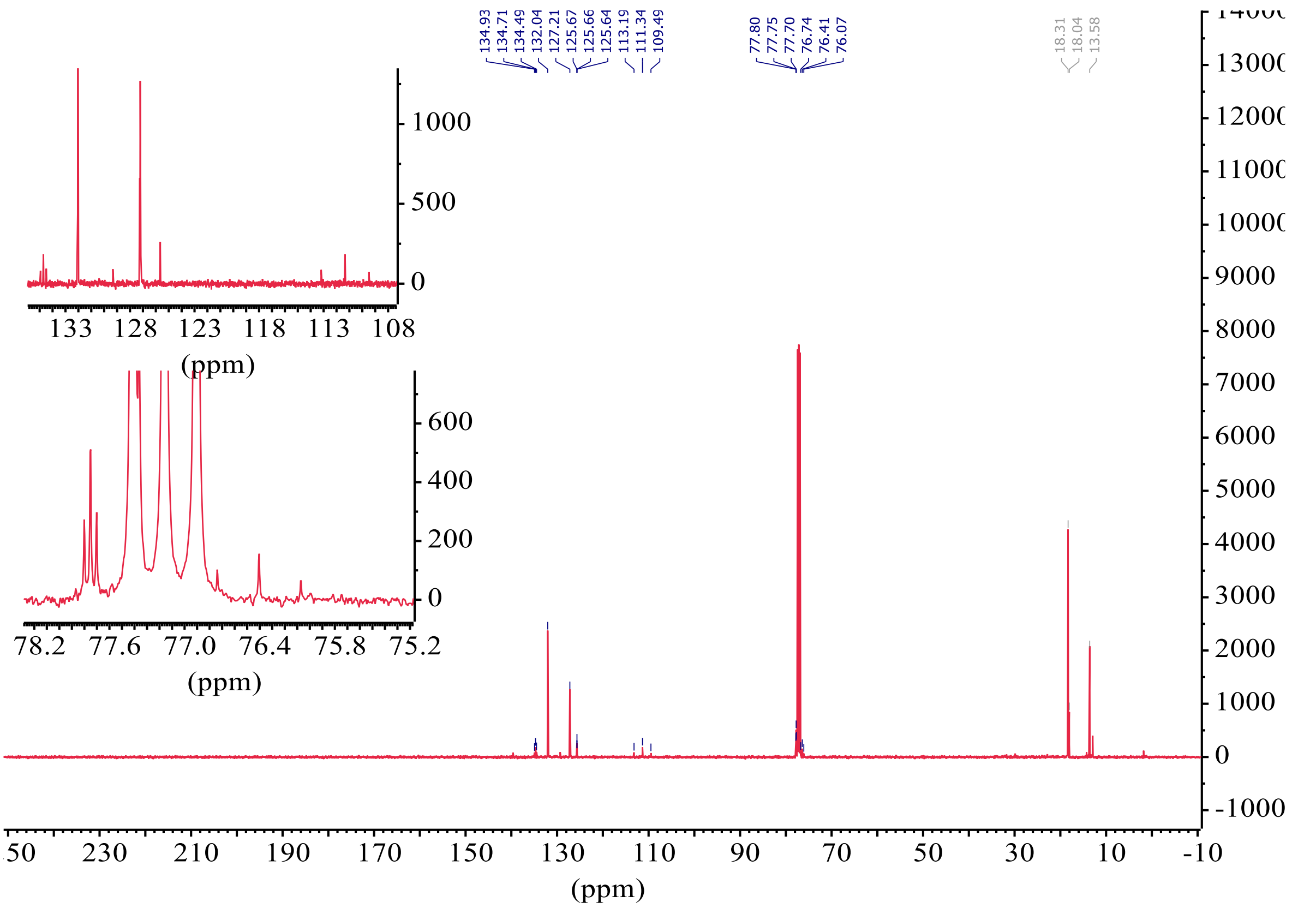

Figure S30: $^{13}C\{^{1}H\}$ NMR (125 MHz) spectrum of **1h** in $CDCl_3$. Residual impurities are marked with grey ticks.

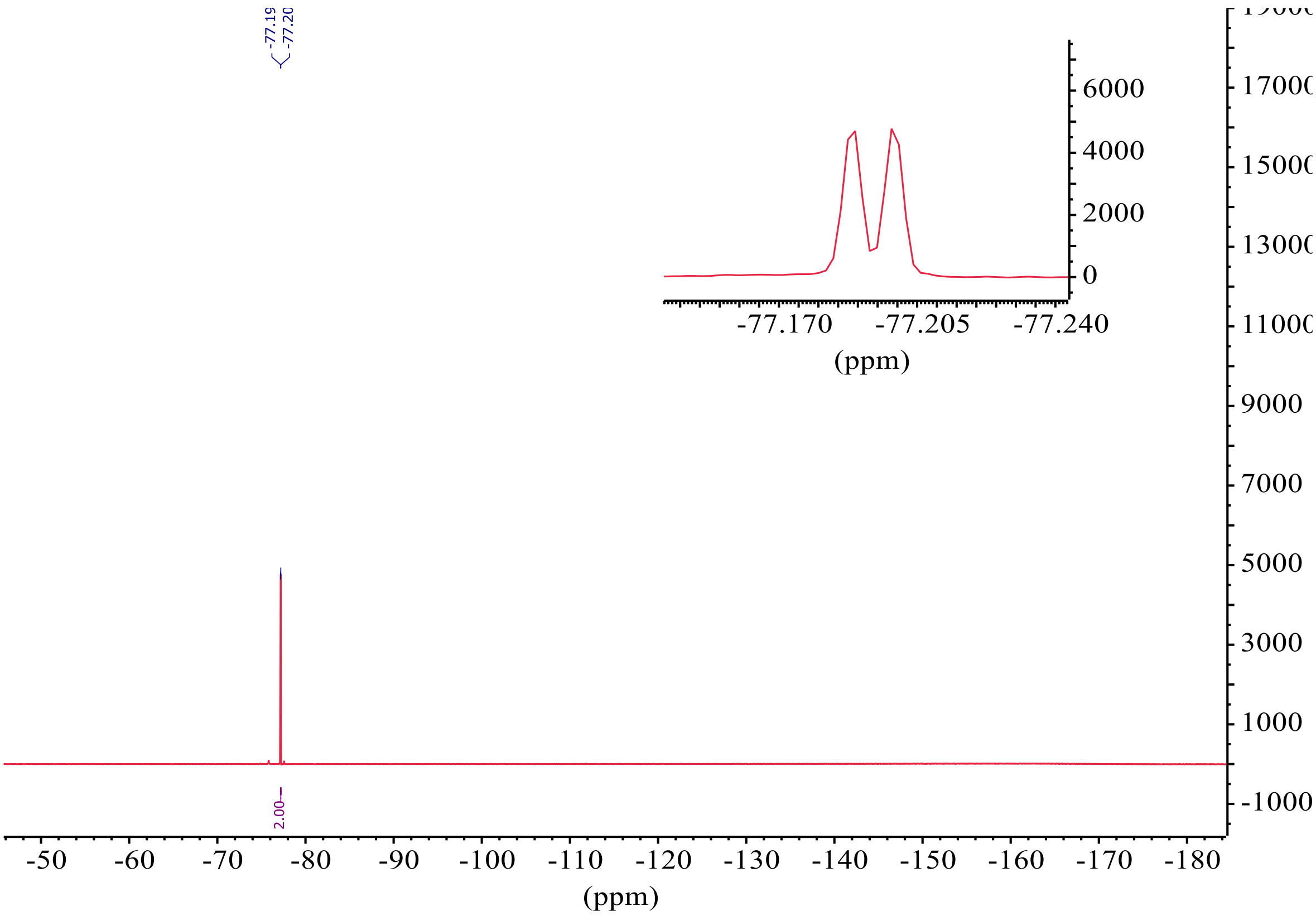

Figure S31: ^{19}F NMR (470 MHz) spectrum of **1h** in $CDCl_3$.

1i

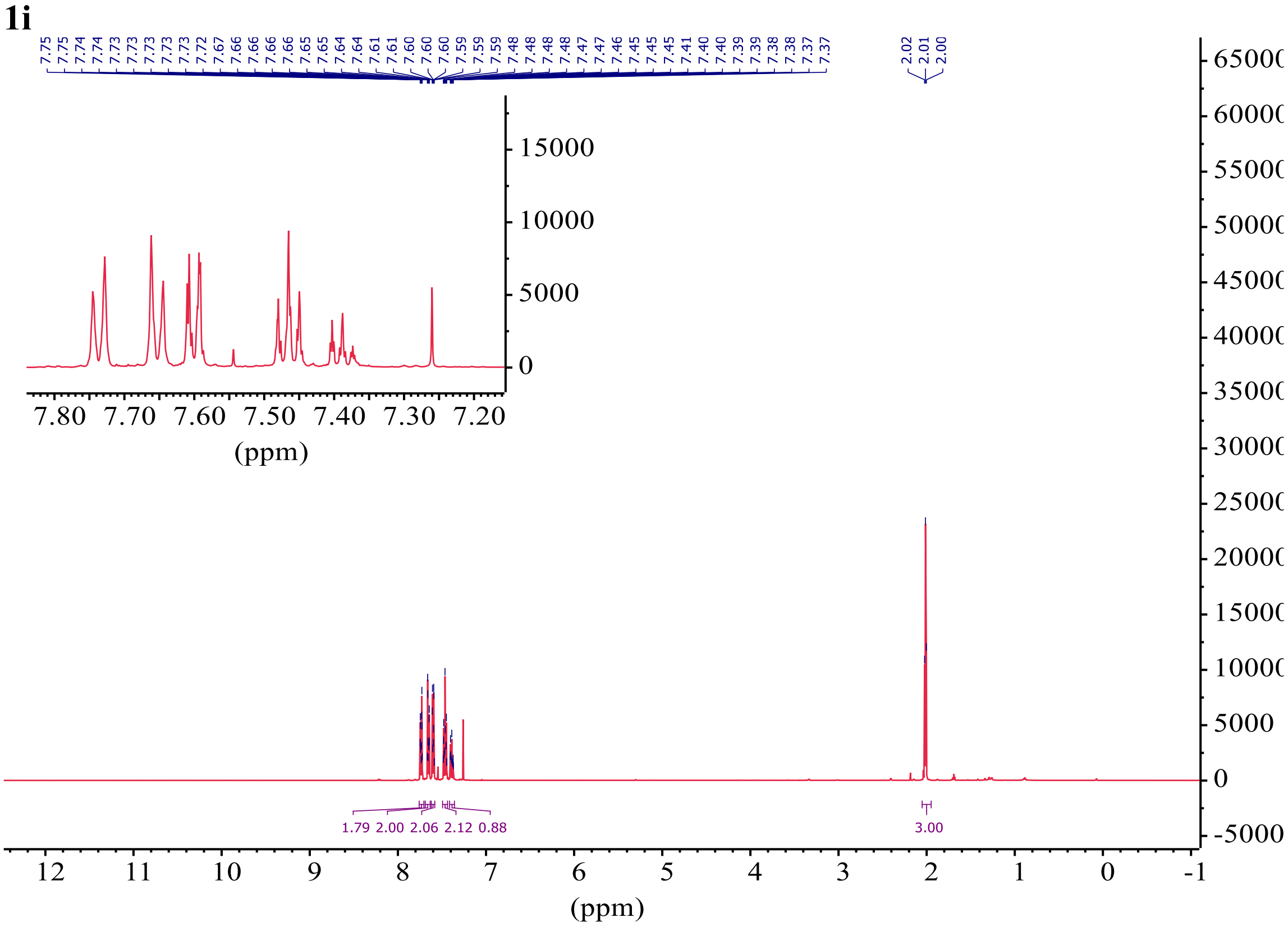

Figure S32: ^{1}H NMR (500 MHz) spectrum of **1i** in $CDCl_3$.

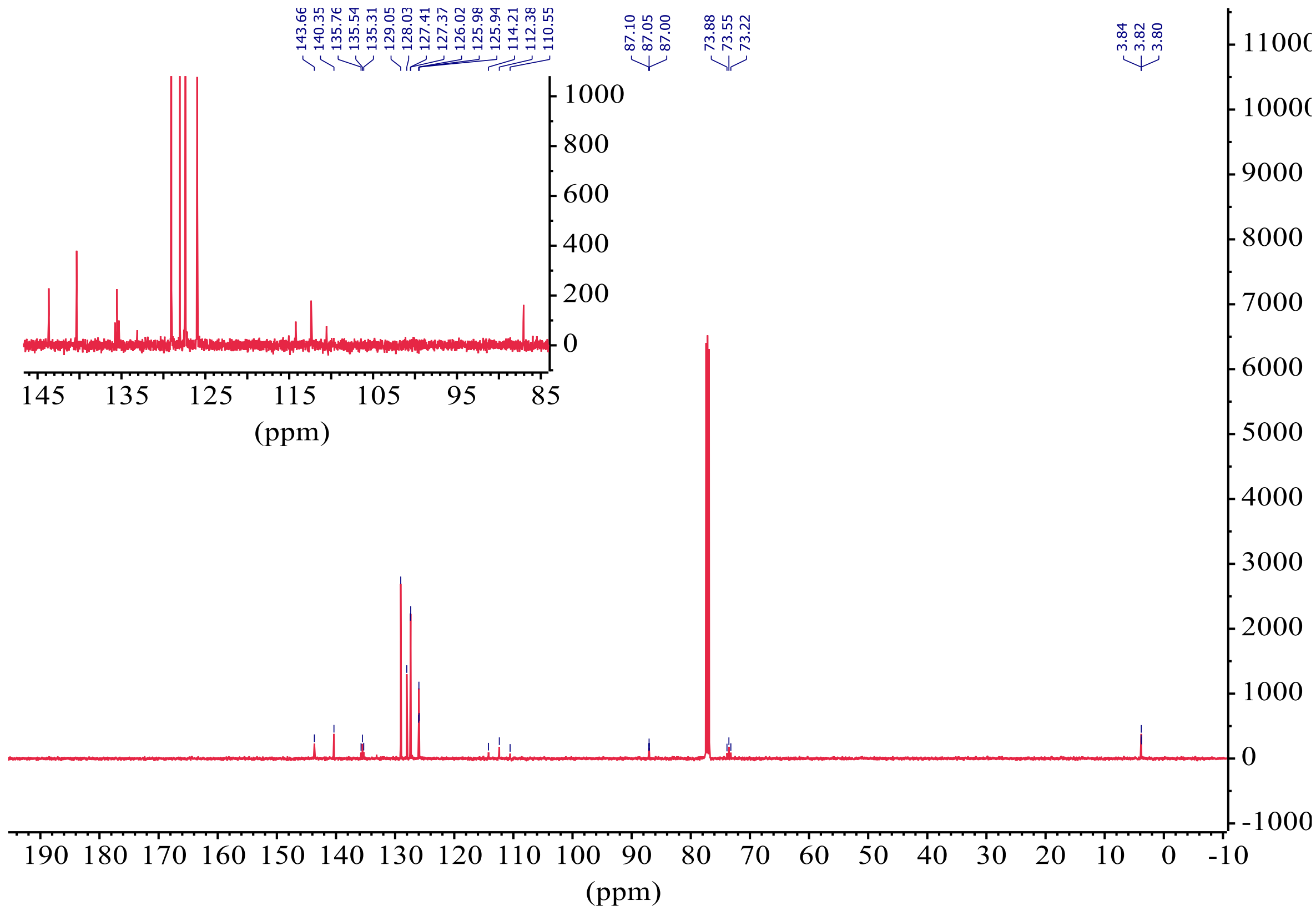

Figure S33: $^{13}C\{^{1}H\}$ NMR (125 MHz) spectrum of **1i** in $CDCl_3$.

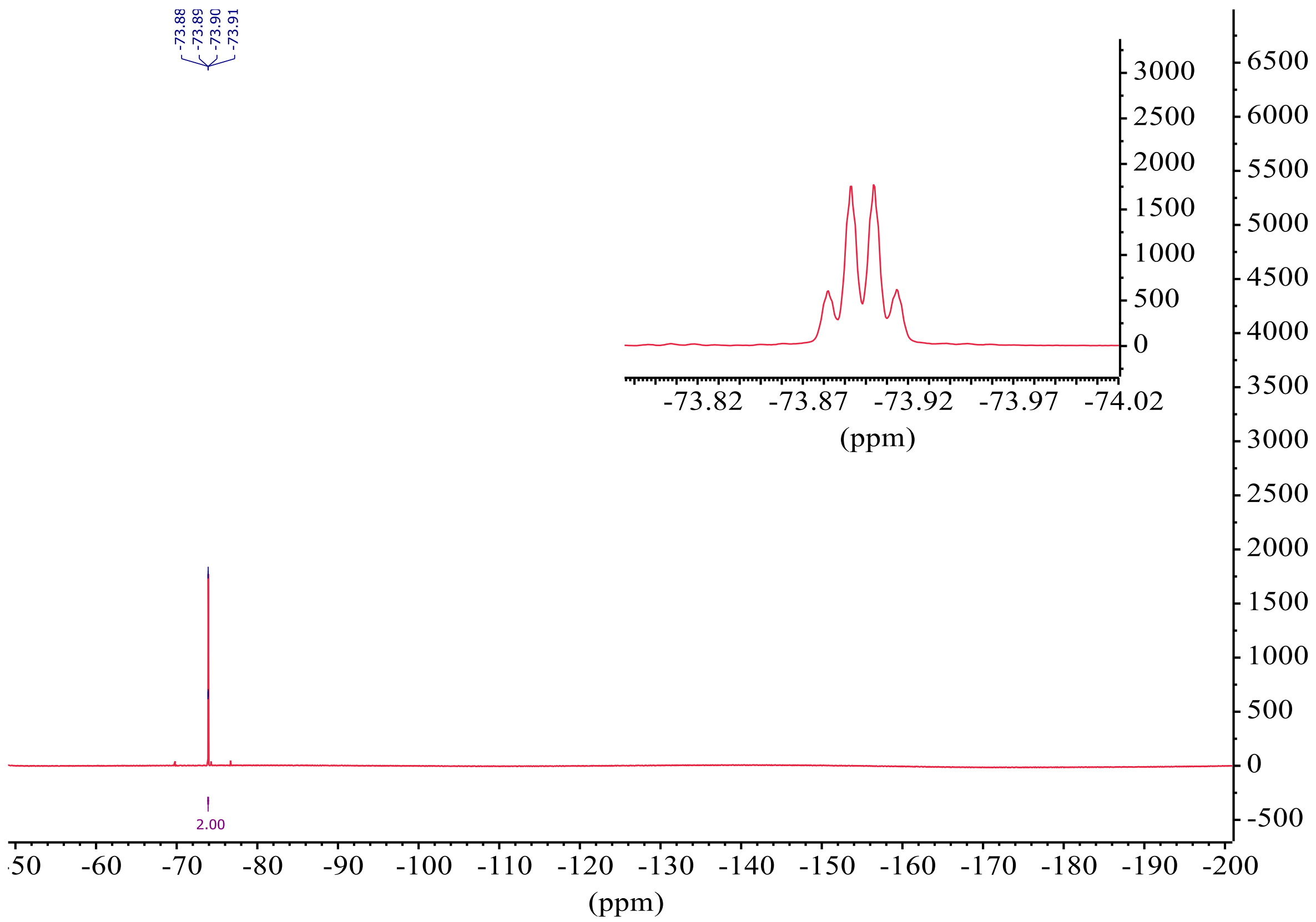

Figure S34: ^{19}F NMR (470 MHz) spectrum of **1i** in $CDCl_3$

1j

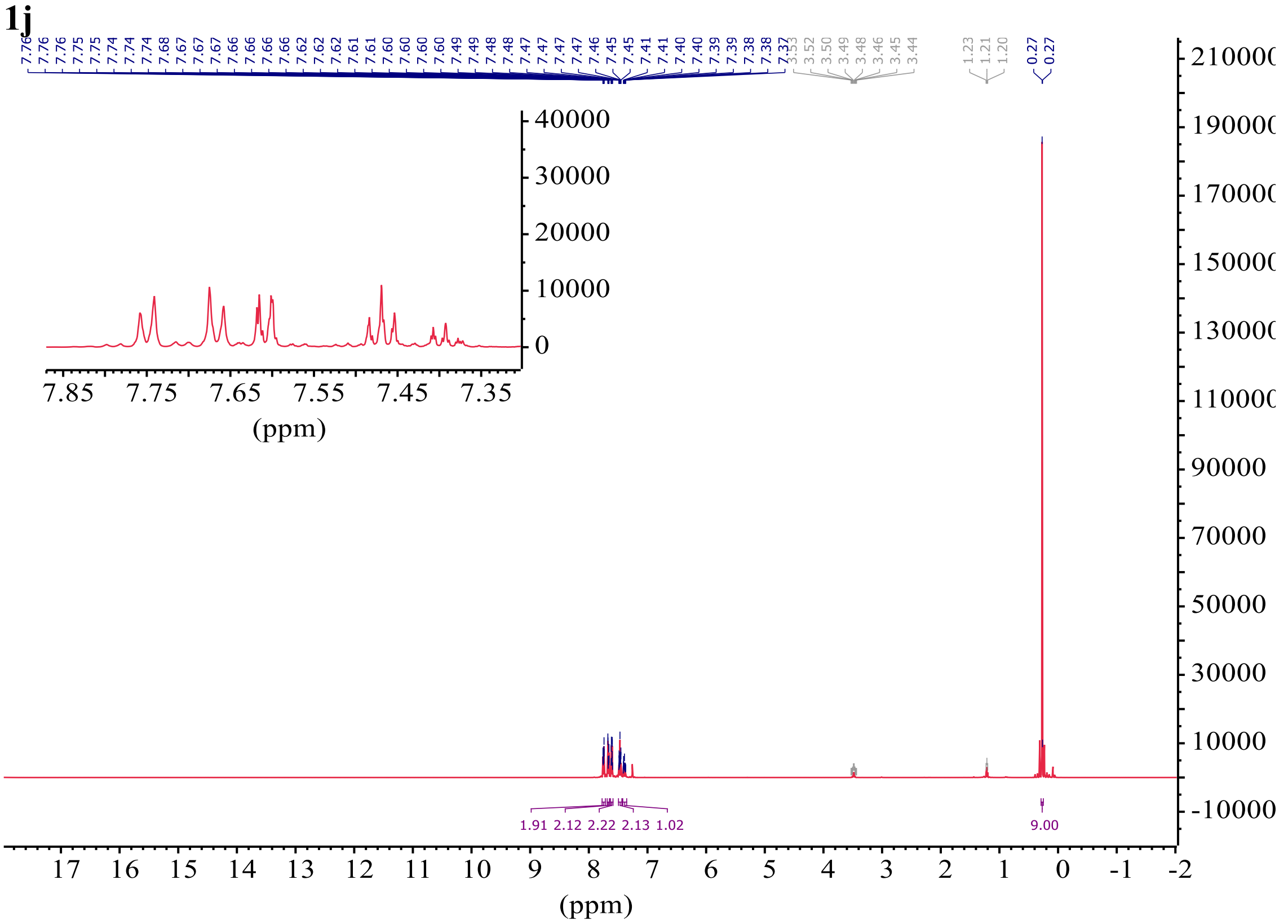

Figure S35: ^{1}H NMR (500 MHz) spectrum of **1j** in $CDCl_3$. Residual solvent impurities are marked with grey ticks.

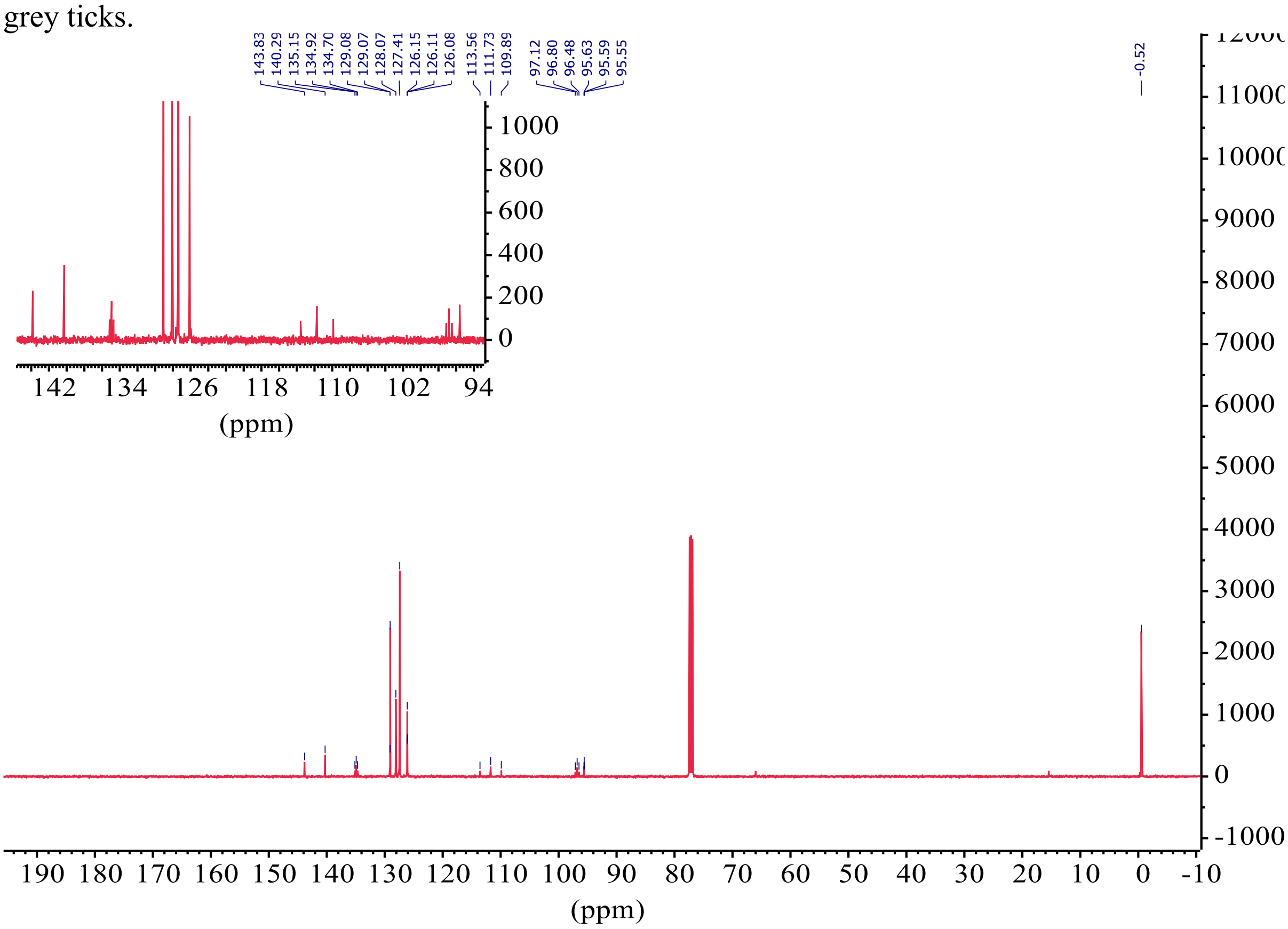

Figure S36: ^{13}C{^{1}H} NMR (125 MHz) spectrum of **1j** in $CDCl_3$.

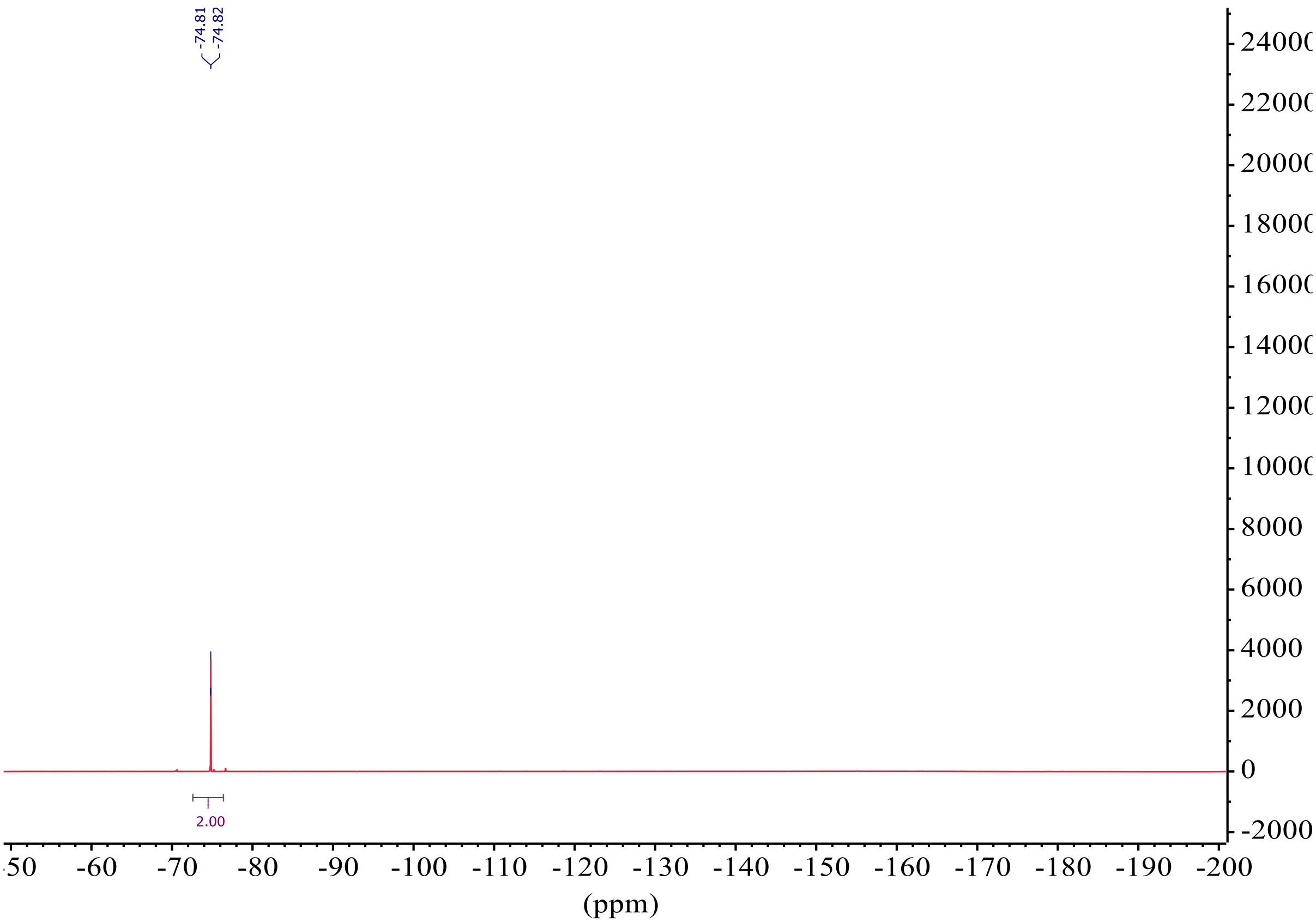

Figure S37: ^{19}F NMR (470 MHz) spectrum of **1j** in $CDCl_3$

1k

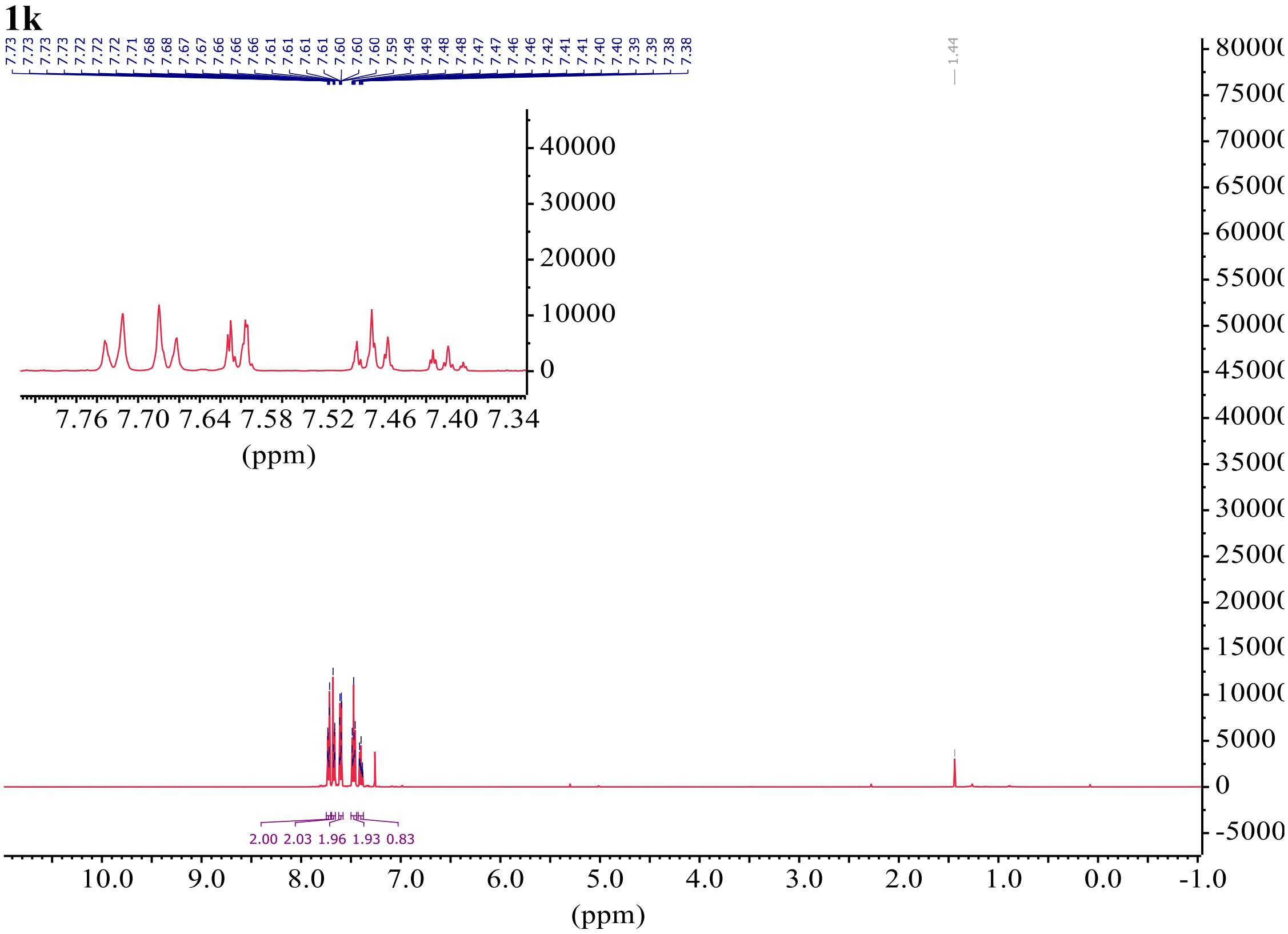

Figure S38: ^{1}H NMR (500 MHz) spectrum of **1k** in $CDCl_3$. Residual solvent impurities are marked with grey ticks.

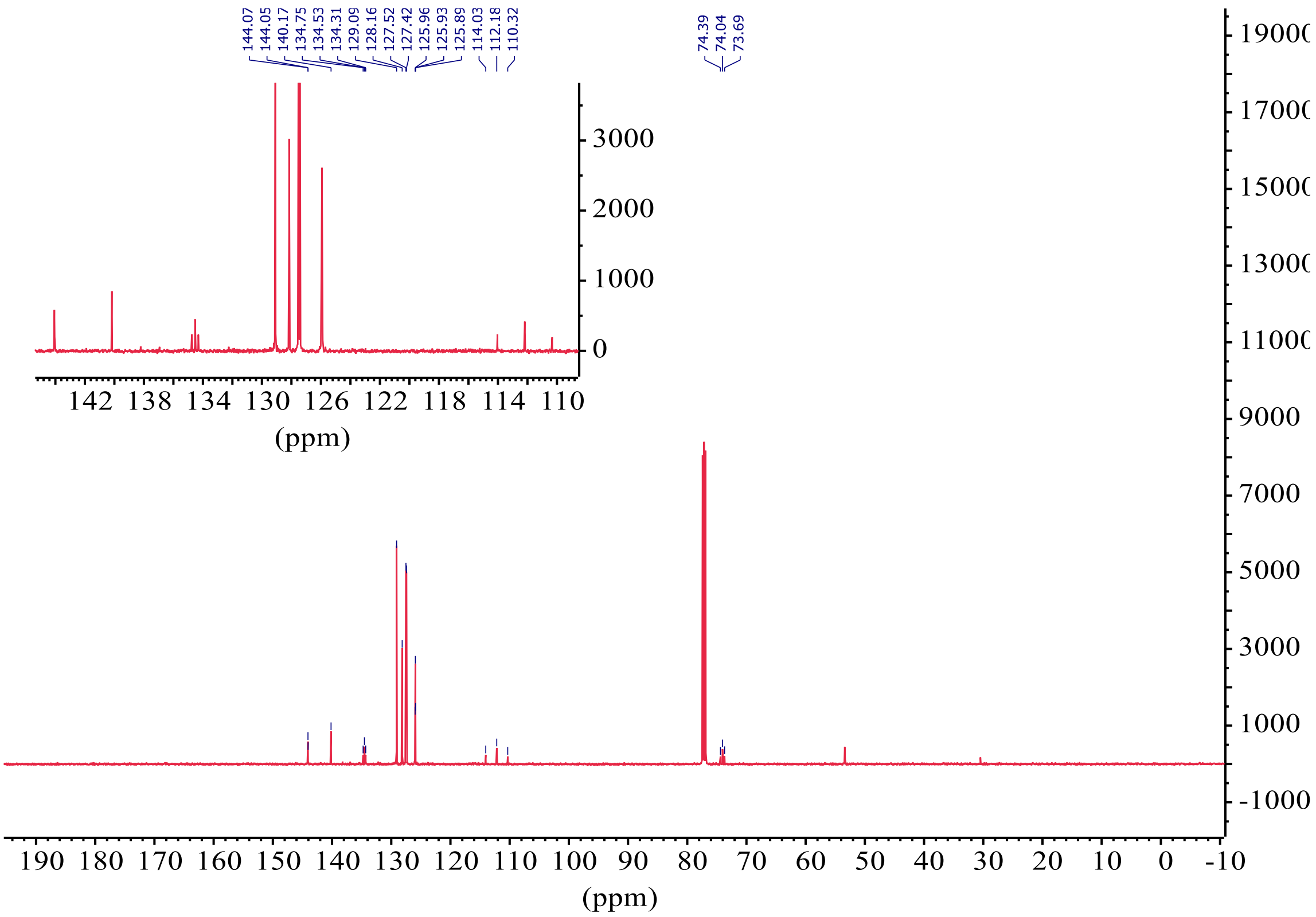

Figure S39: $^{13}C\{^{1}H\}$ NMR (125 MHz) spectrum of **1k** in $CDCl_3$.

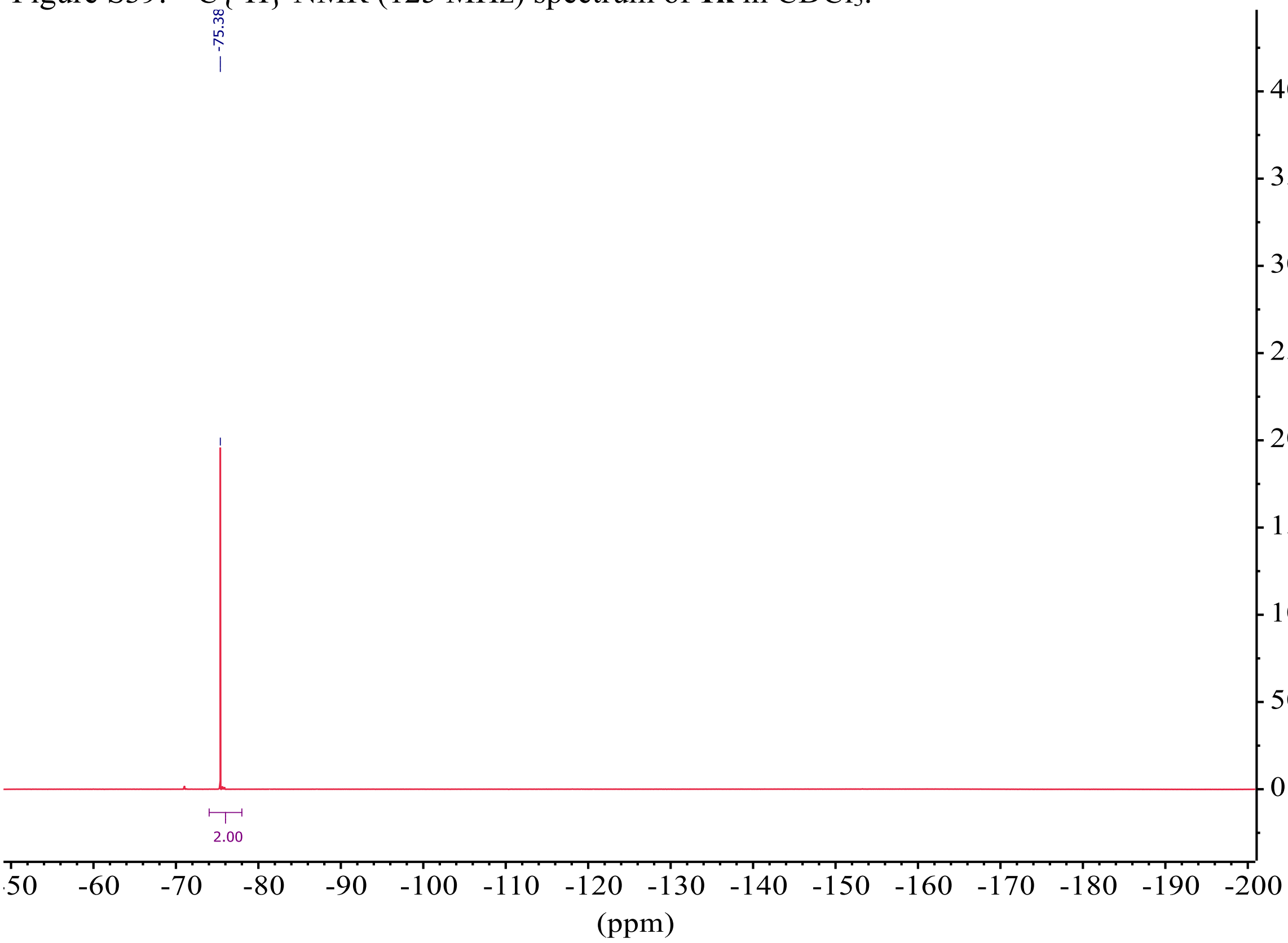

Figure S40: ^{19}F NMR (470 MHz) spectrum of **1k** in $CDCl_3$

1l

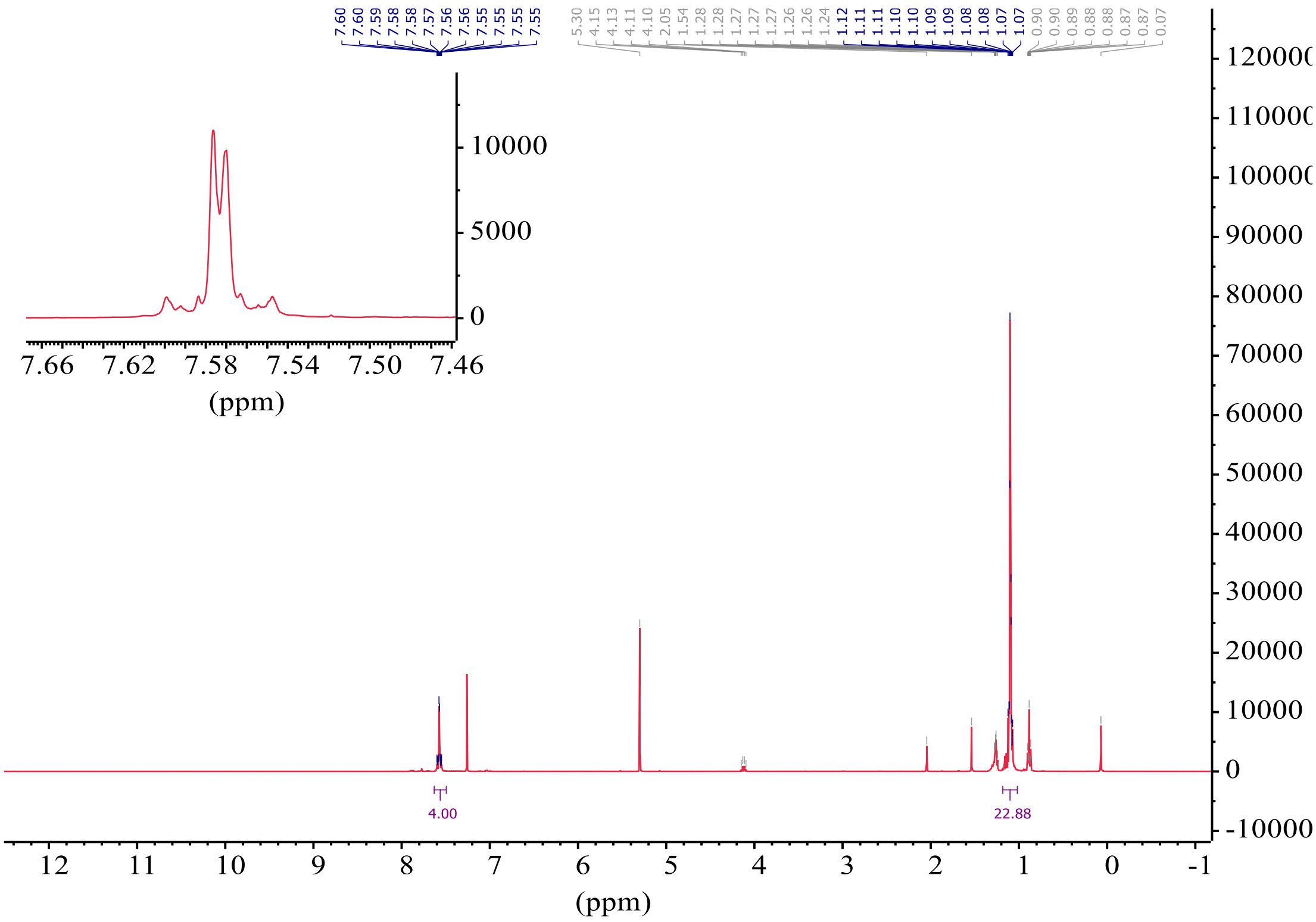

Figure S41: ^{1}H NMR (400 MHz) spectrum of **1l** in $CDCl_3$. Residual solvent impurities are marked with grey ticks.

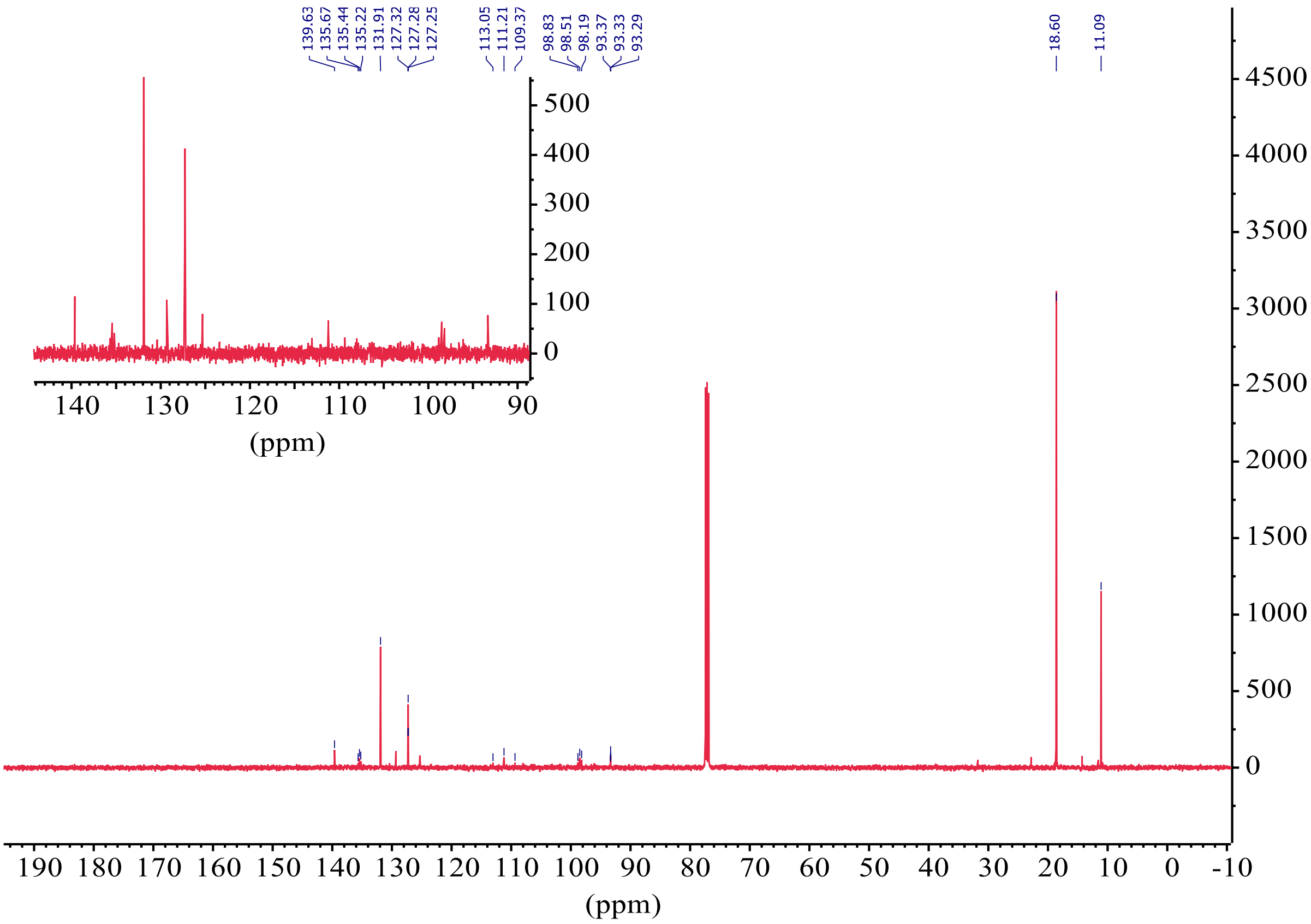

Figure S42: $^{13}C\{^{1}H\}$ NMR (125 MHz) spectrum of **1l** in $CDCl_3$.

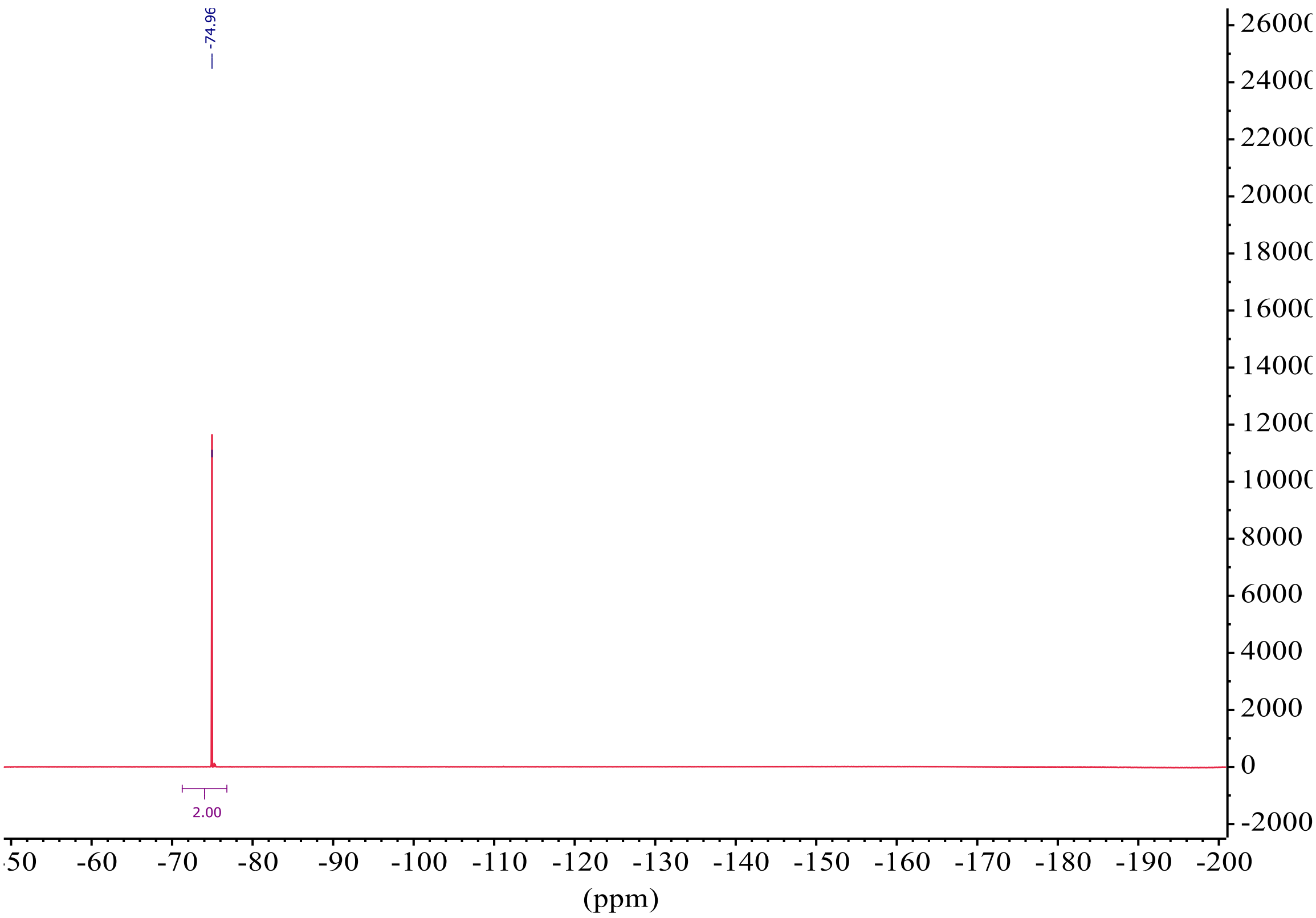

Figure S43: ^{19}F NMR (470 MHz) spectrum of **1l** in $CDCl_3$

1m

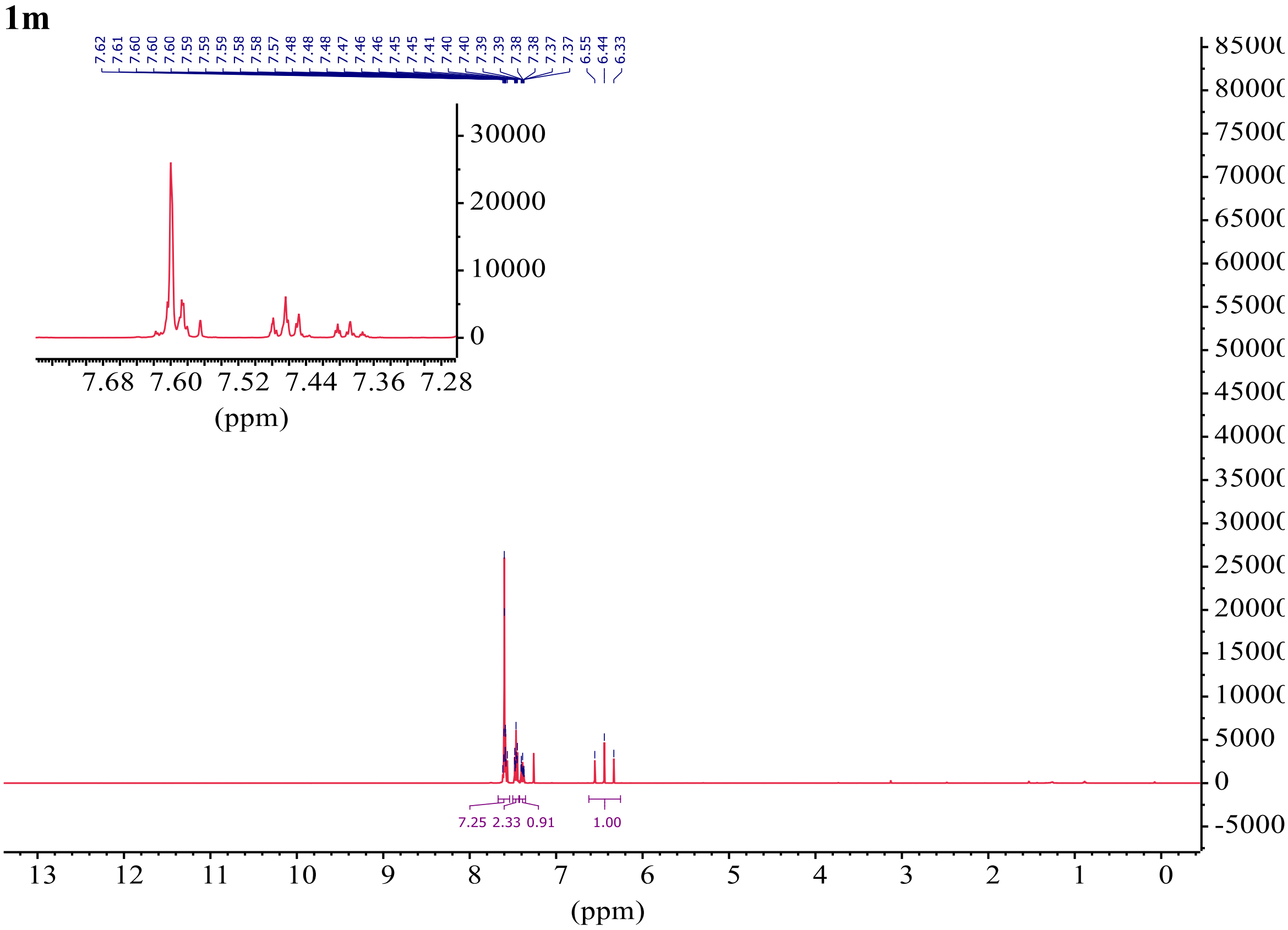

S44: ^{1}H NMR (500 MHz) spectrum of **1m** in $CDCl_3$.

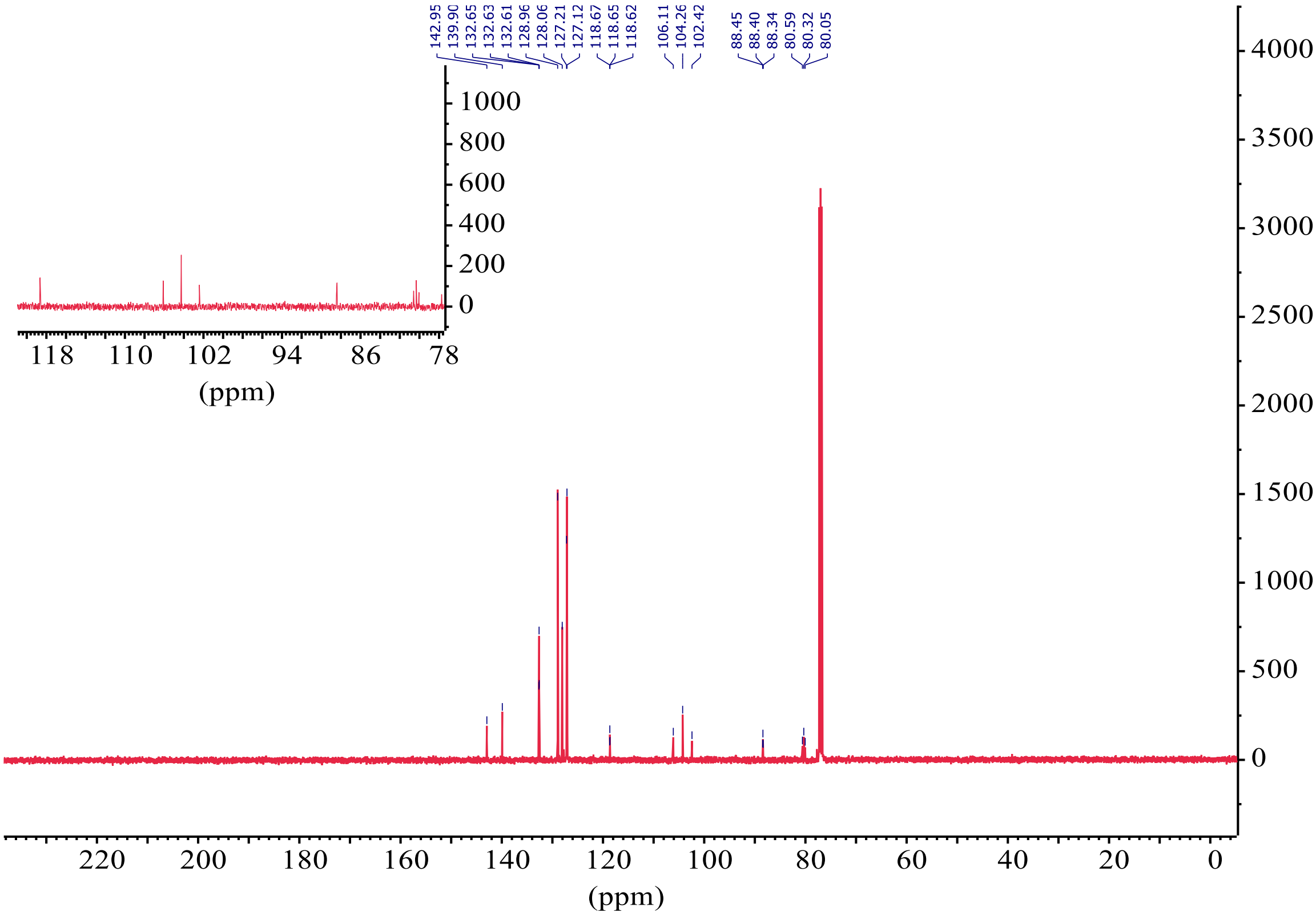

Figure S45: $^{13}C\{^{1}H\}$ NMR (125 MHz) spectrum of **1m** in $CDCl_3$.

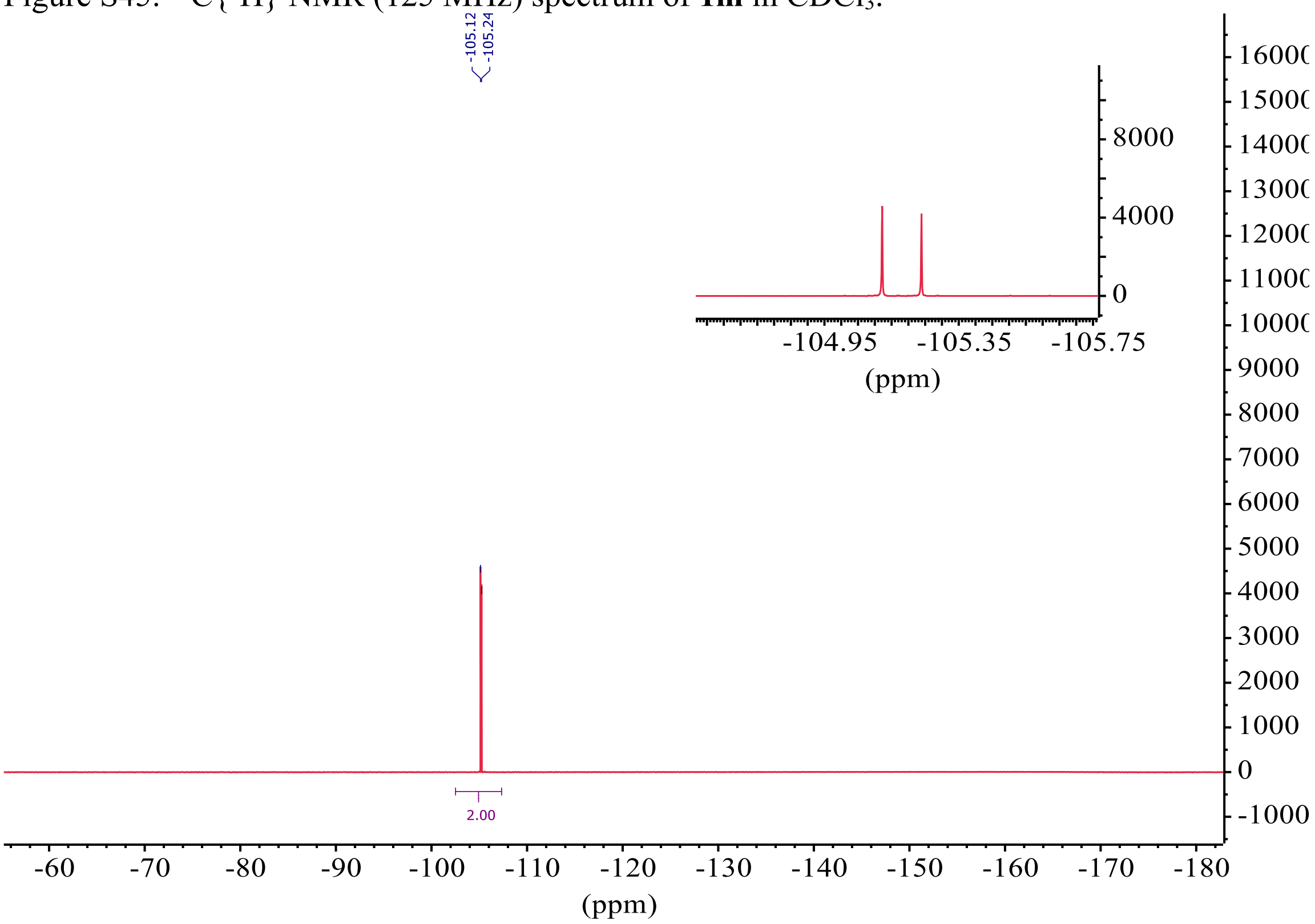

Figure S46: ^{19}F NMR (470 MHz) spectrum of **1m** in $CDCl_3$.

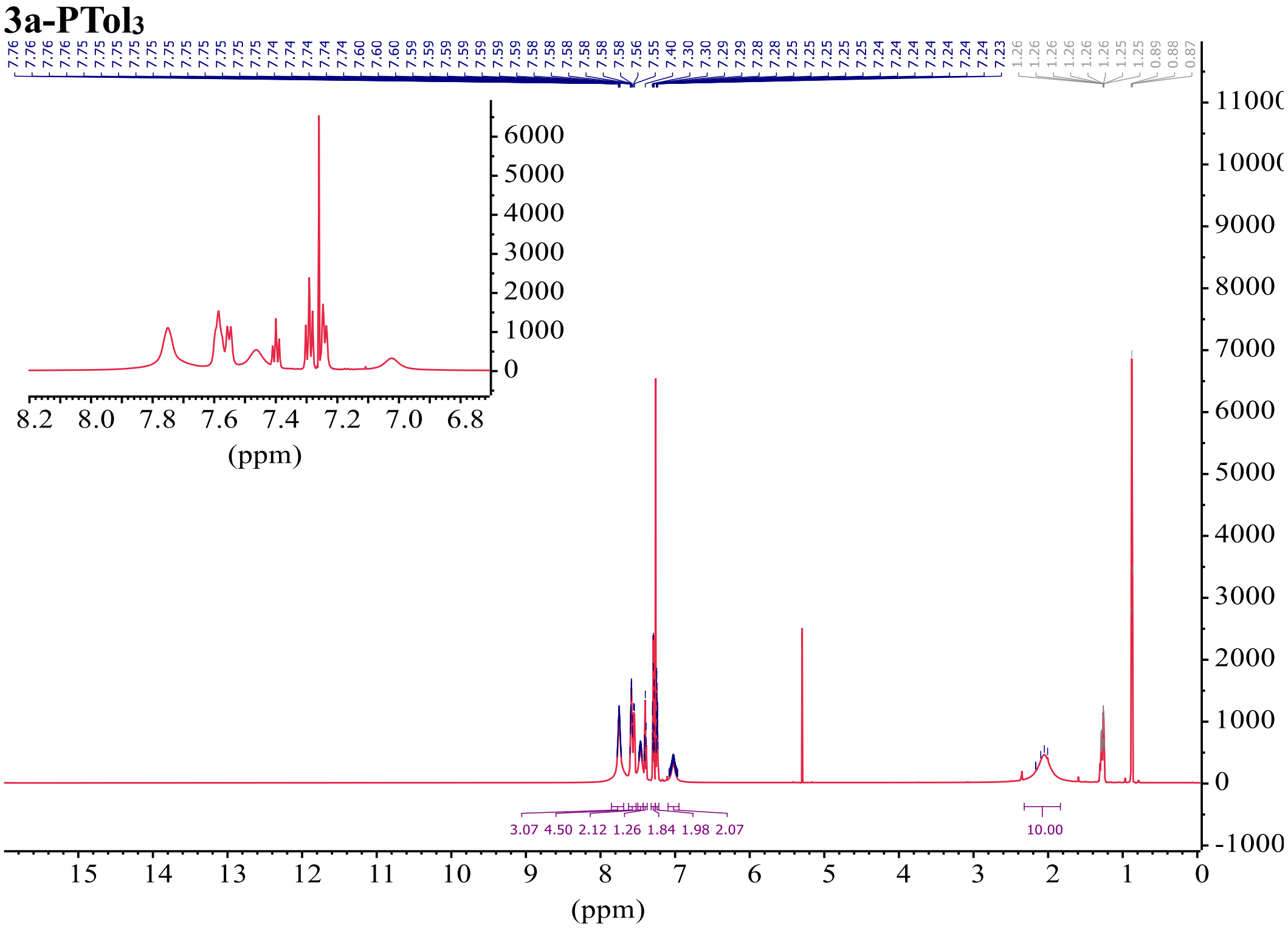

Figure S47: ^{1}H NMR (700 MHz) spectrum of **3a-PTol$_3$** in $CDCl_3$. Residual solvent impurities are marked with grey ticks.

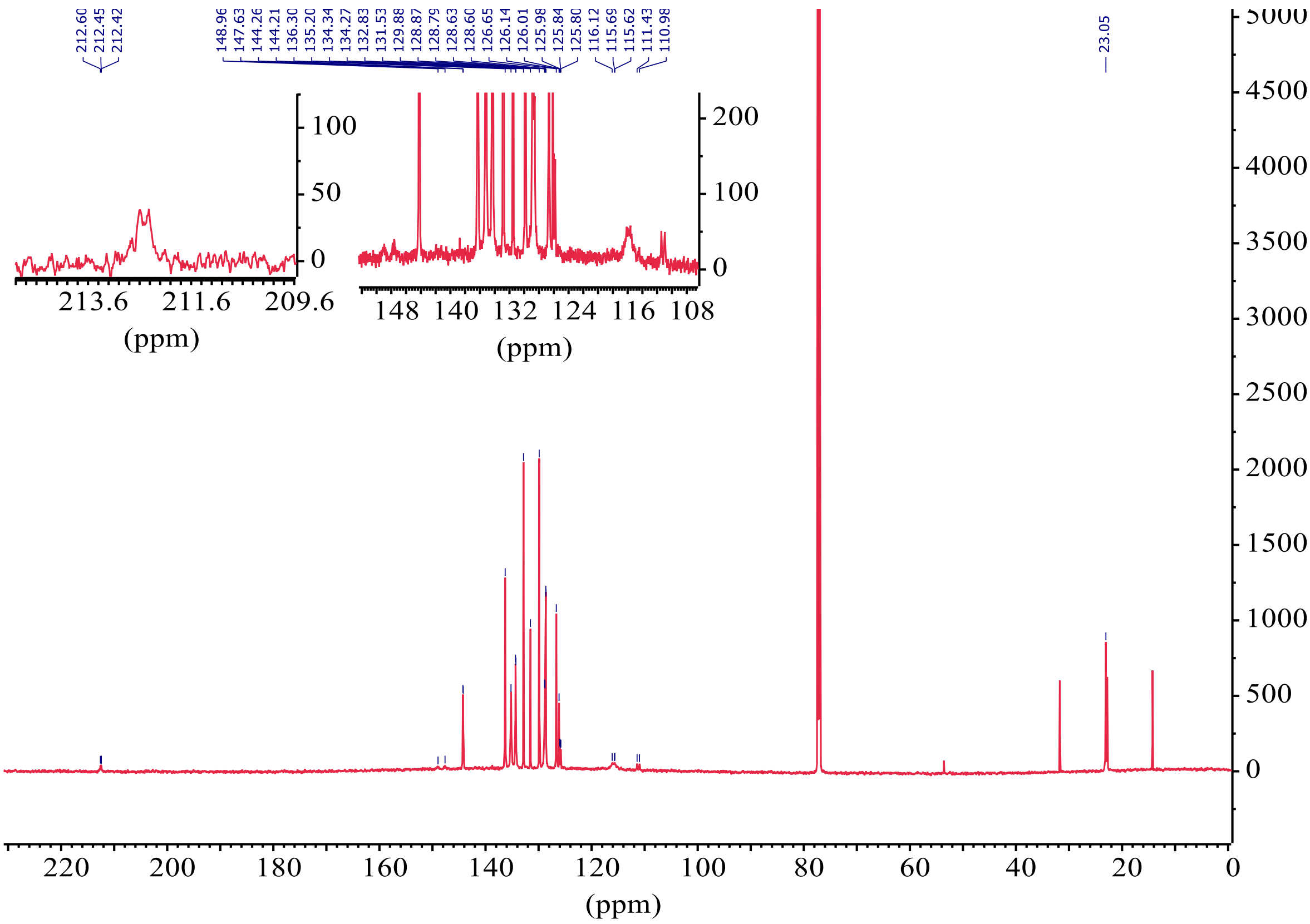

Figure S48: $^{13}C\{^{1}H\}$ NMR (175 MHz) spectrum of **3a-PTol$_3$** in $CDCl_3$.

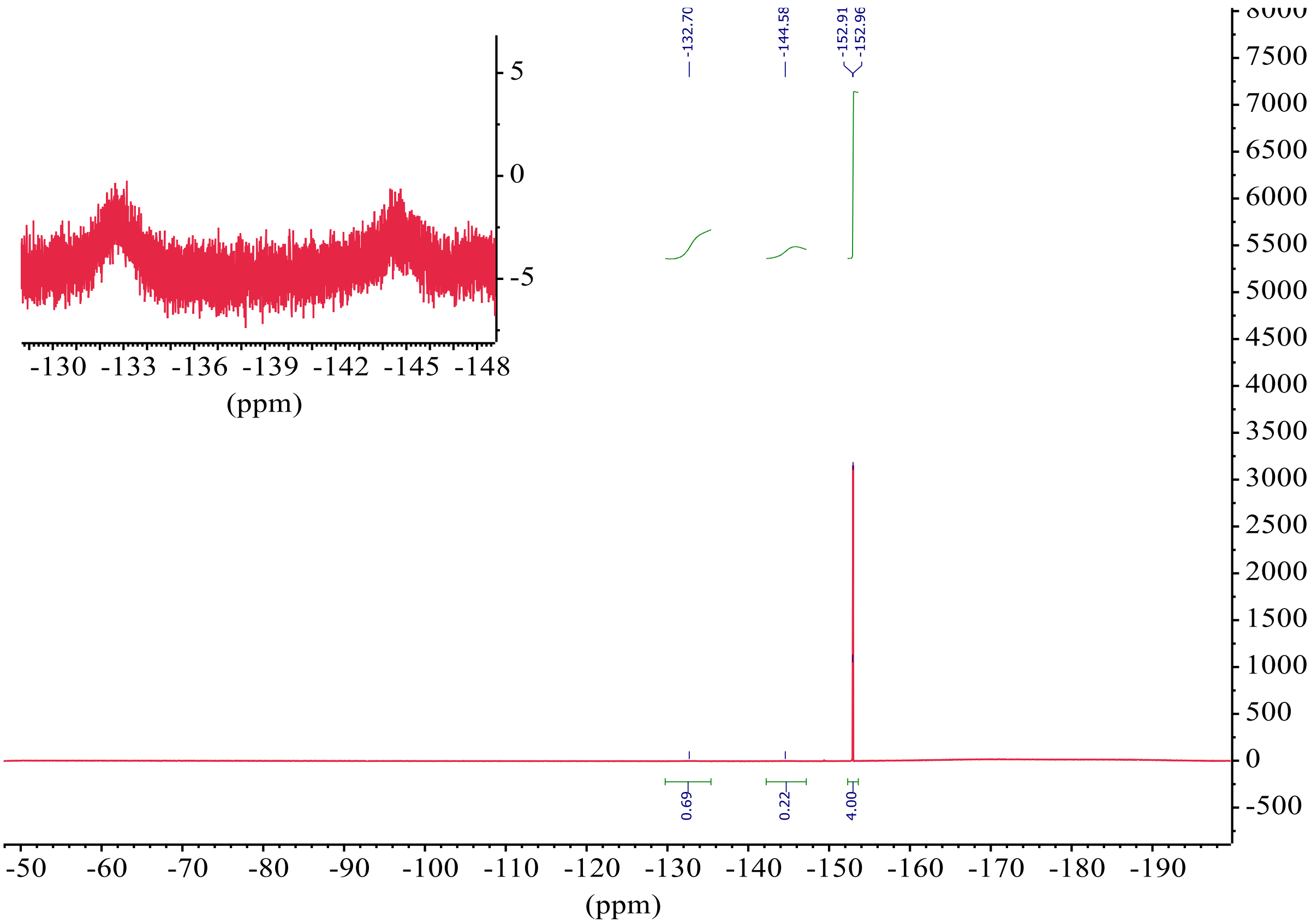

Figure S49: $^{19}F\{^1H\}$ NMR (470 MHz) spectrum of **3a-PTol₃** in $CDCl_3$.

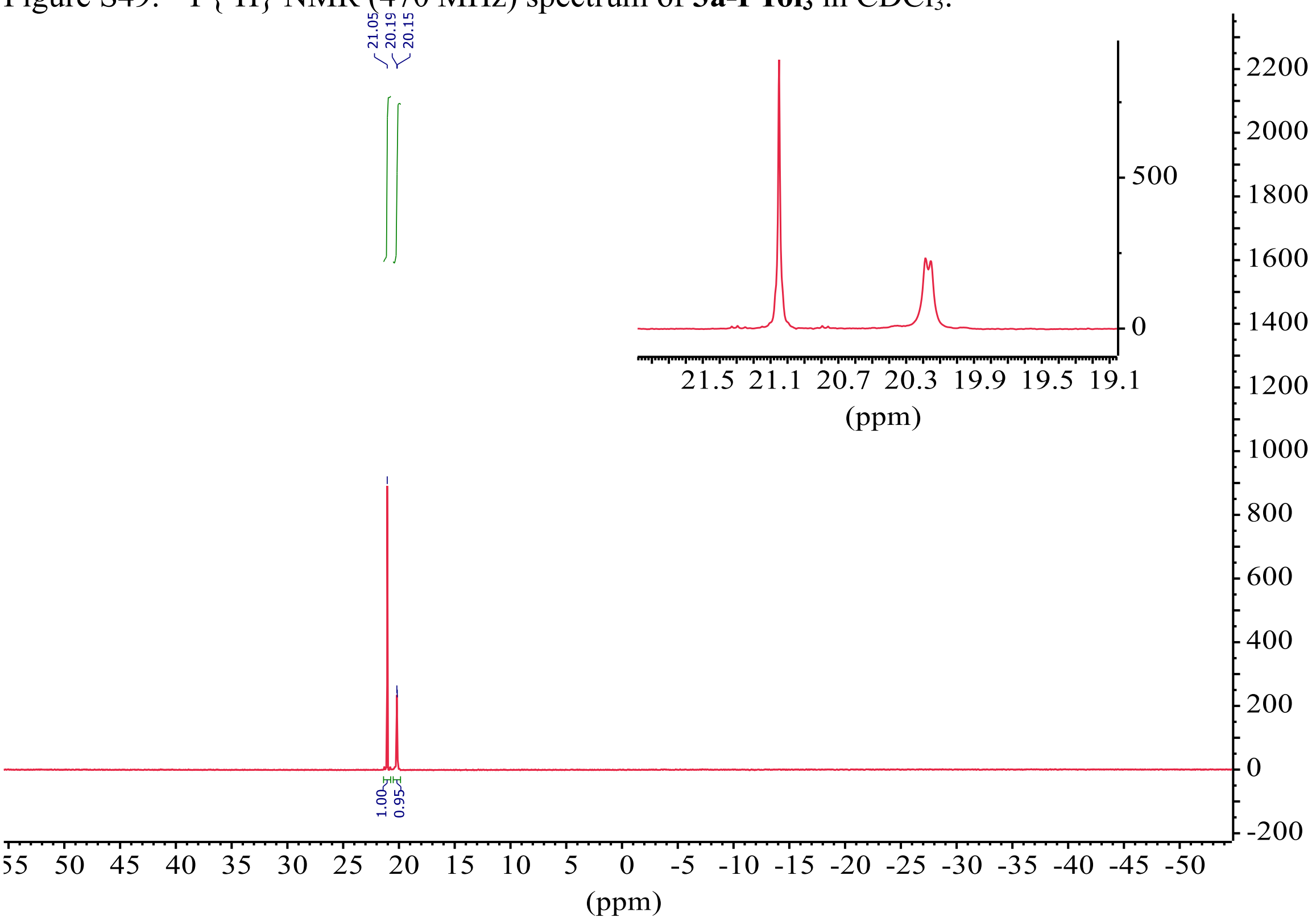

Figure S50: $^{31}P\{^1H\}$ NMR (202 MHz) spectrum of **3a-PTol₃** in $CDCl_3$.

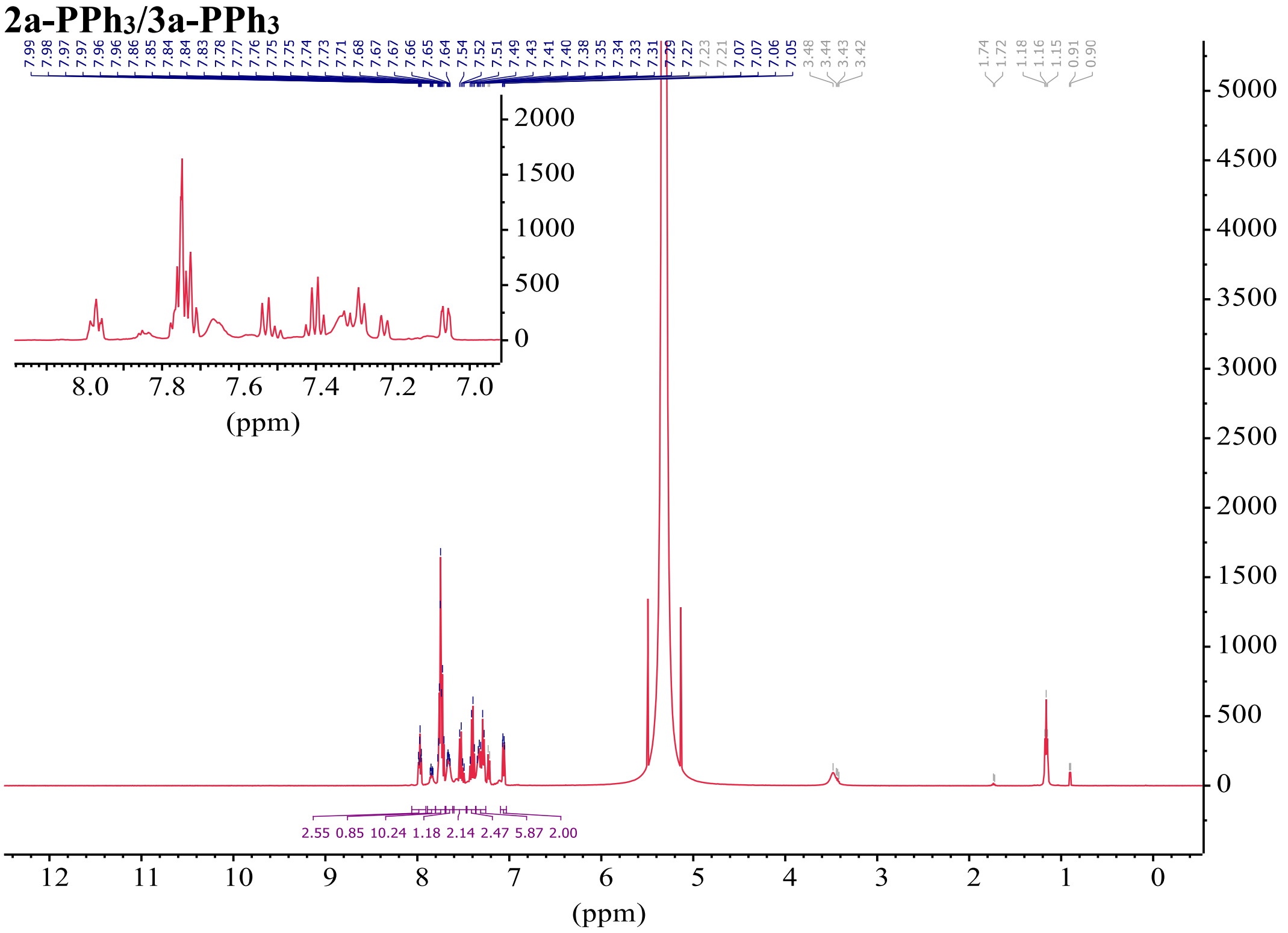

Figure S51: ^{1}H NMR (500 MHz) spectrum of **2a-PPh$_3$/3a-PPh$_3$** in CH_2Cl_2. Residual solvent impurities are marked with grey ticks.

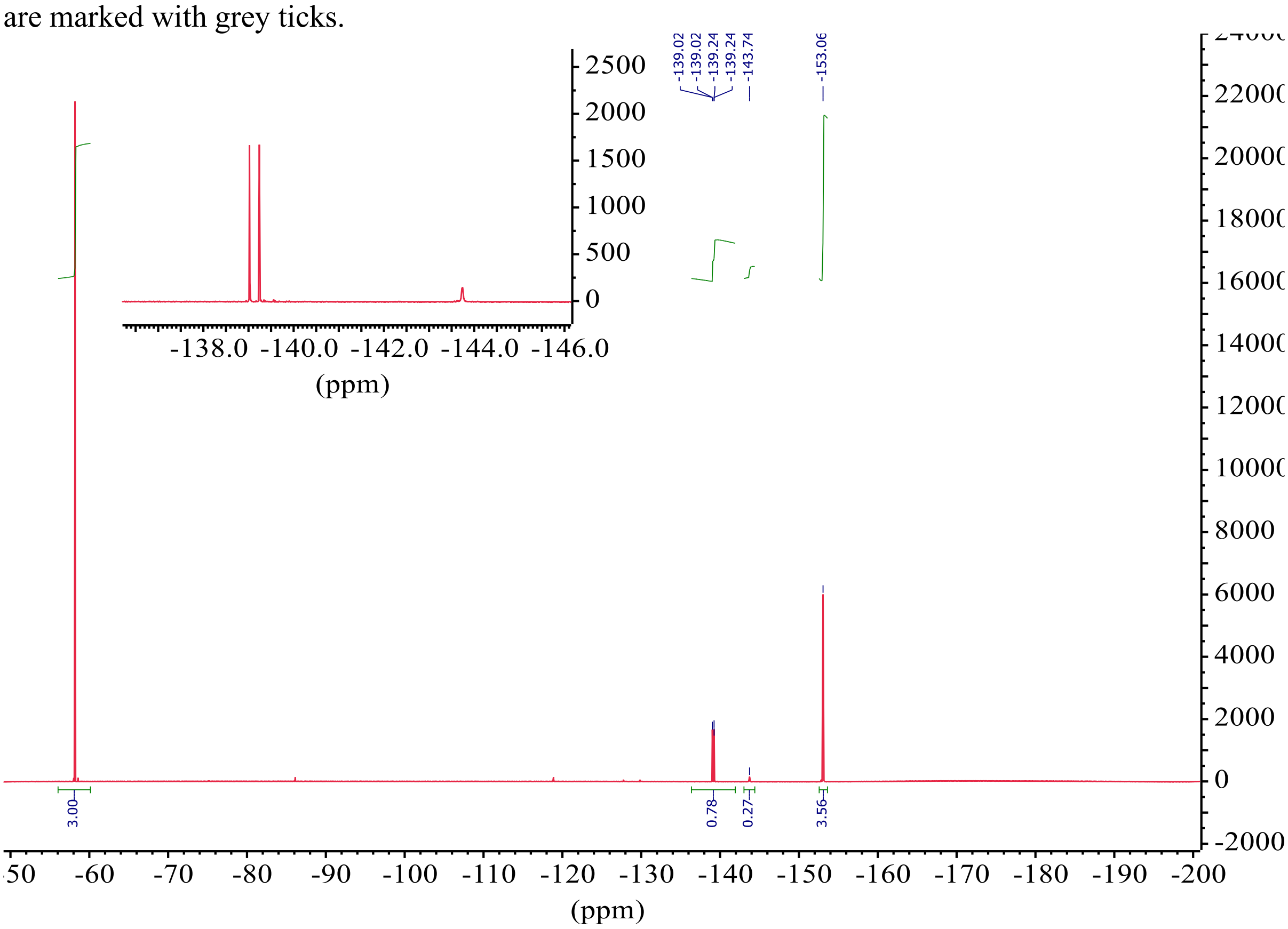

Figure S52: $^{19}F\{^{1}H\}$ NMR (470 MHz) spectrum of **2a-PPh$_3$/3a-PPh$_3$** in CH_2Cl_2.

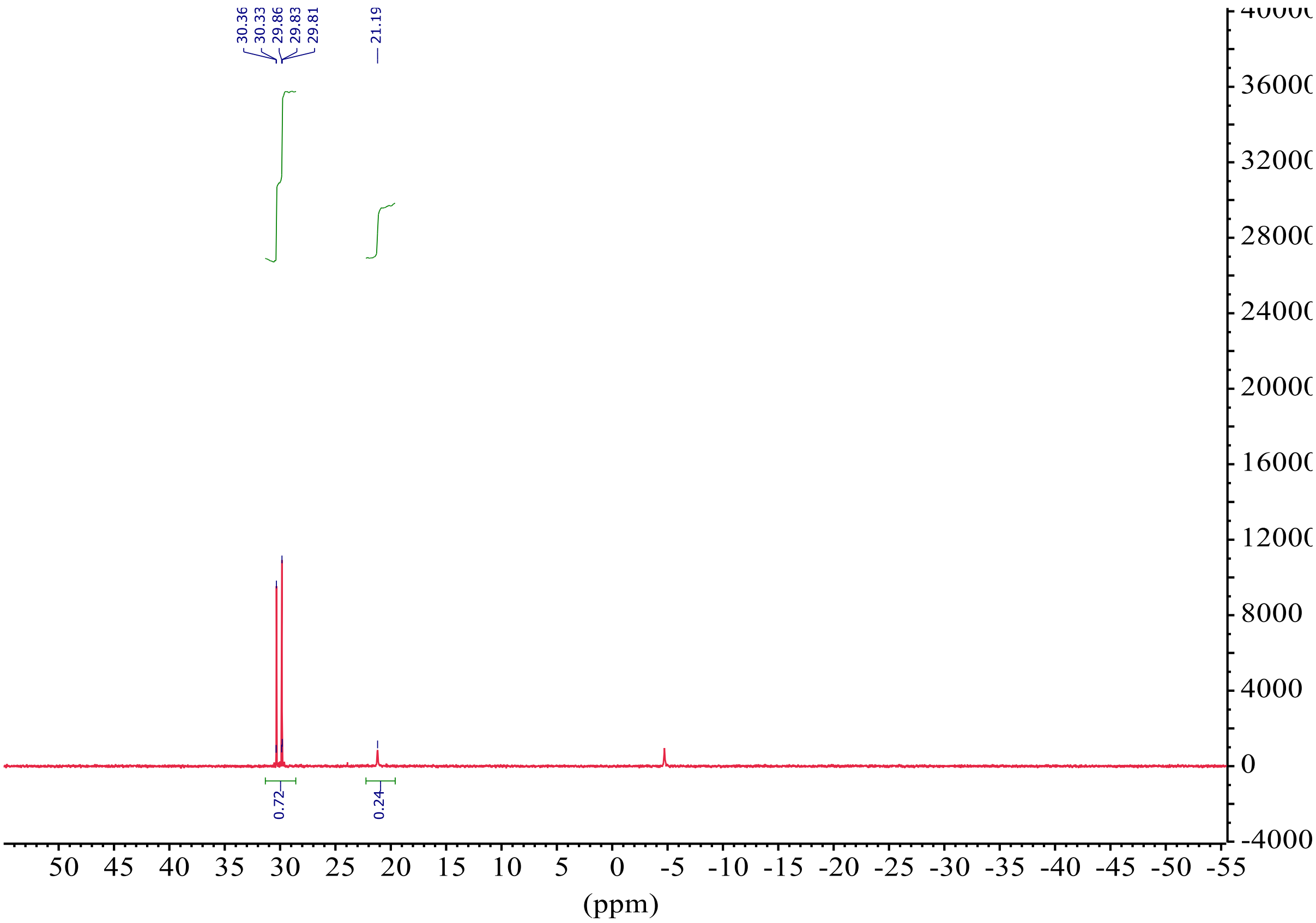

Figure S53: $^{31}P\{^{1}H\}$ NMR (202 MHz) spectrum of **2a-PPh$_3$/3a-PPh$_3$** in CH_2Cl_2.

3a-PMes$_3$

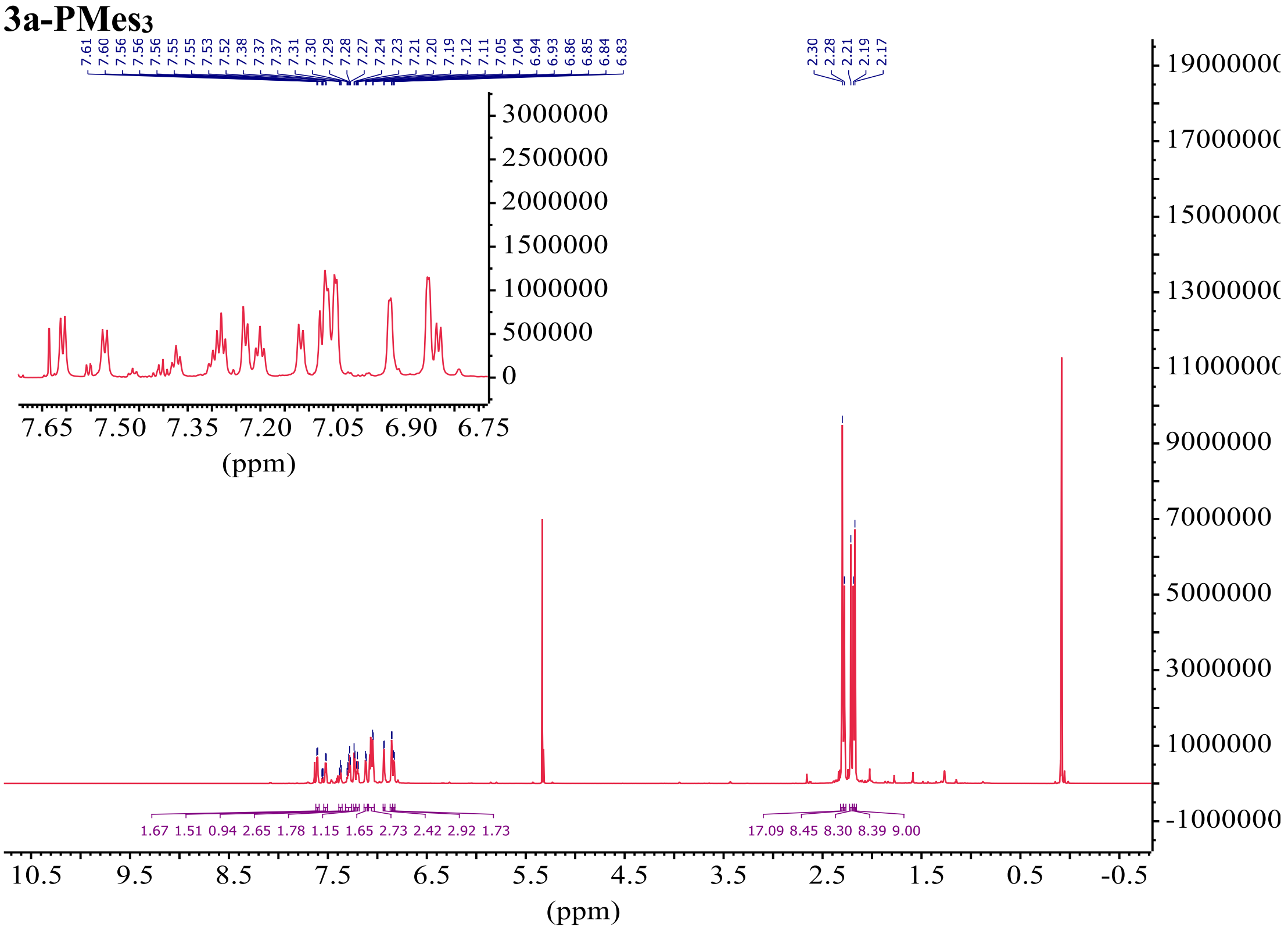

Figure S54: ^{1}H NMR (900 MHz) spectrum of **3a-PMes$_3$** in CD_2Cl_2. Residual solvent impurities are marked with grey ticks.

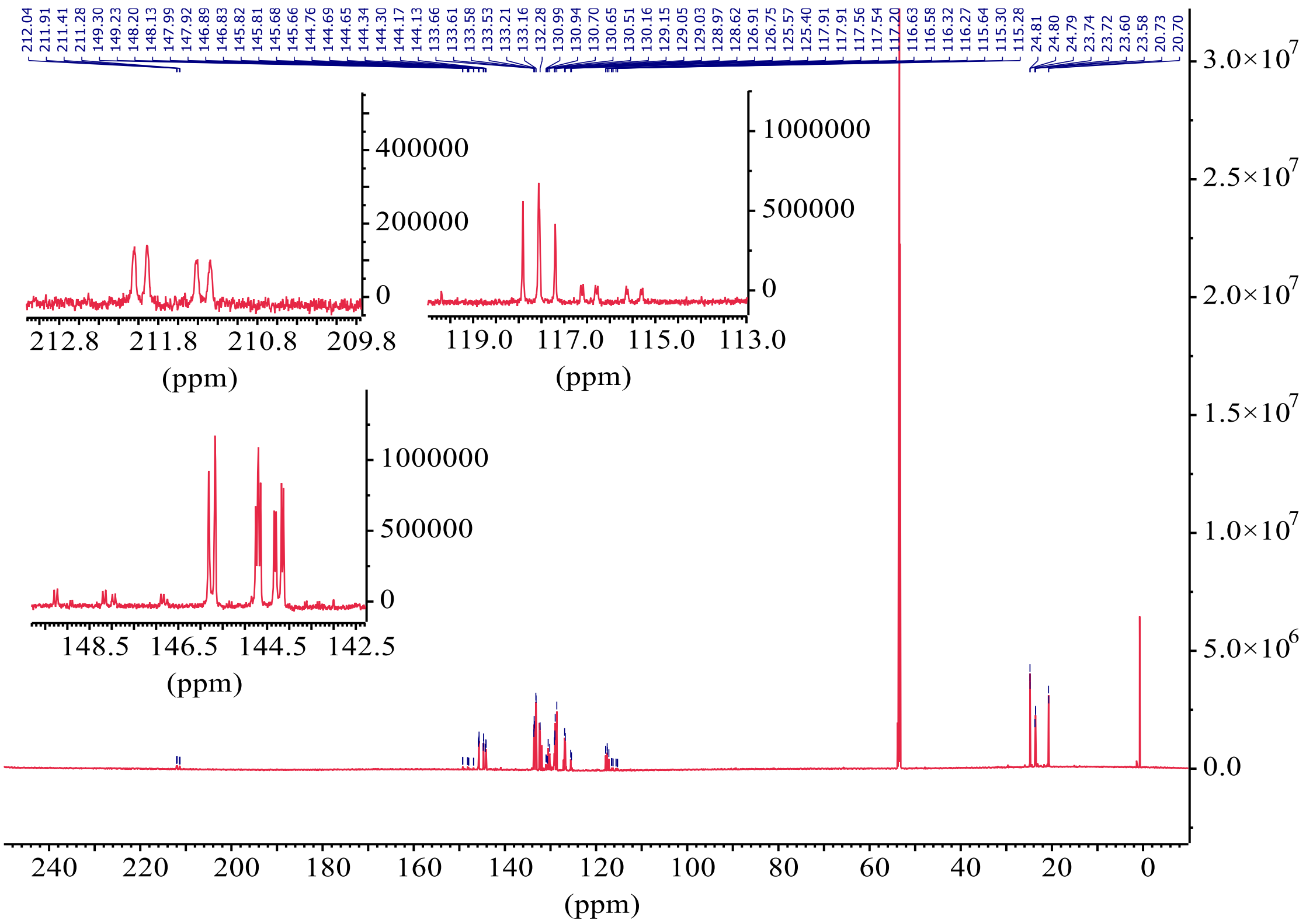

Figure S55: $^{13}C\{^{1}H\}$ NMR (225 MHz) spectrum of **3a-PMes₃** in CD_2Cl_2.

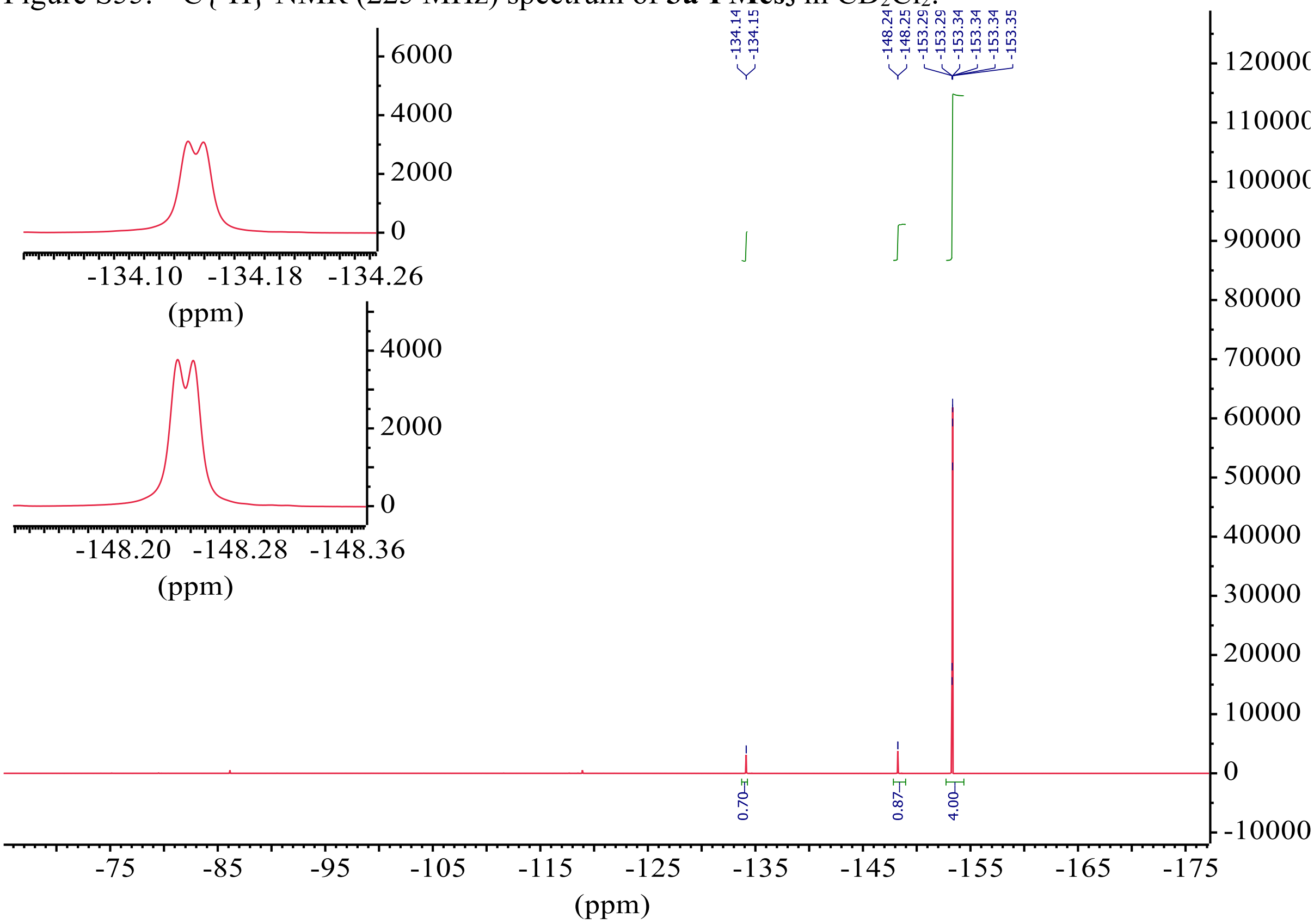

Figure S56: ^{19}F NMR (565 MHz) spectrum of **3a-PMes₃** in CD_2Cl_2.

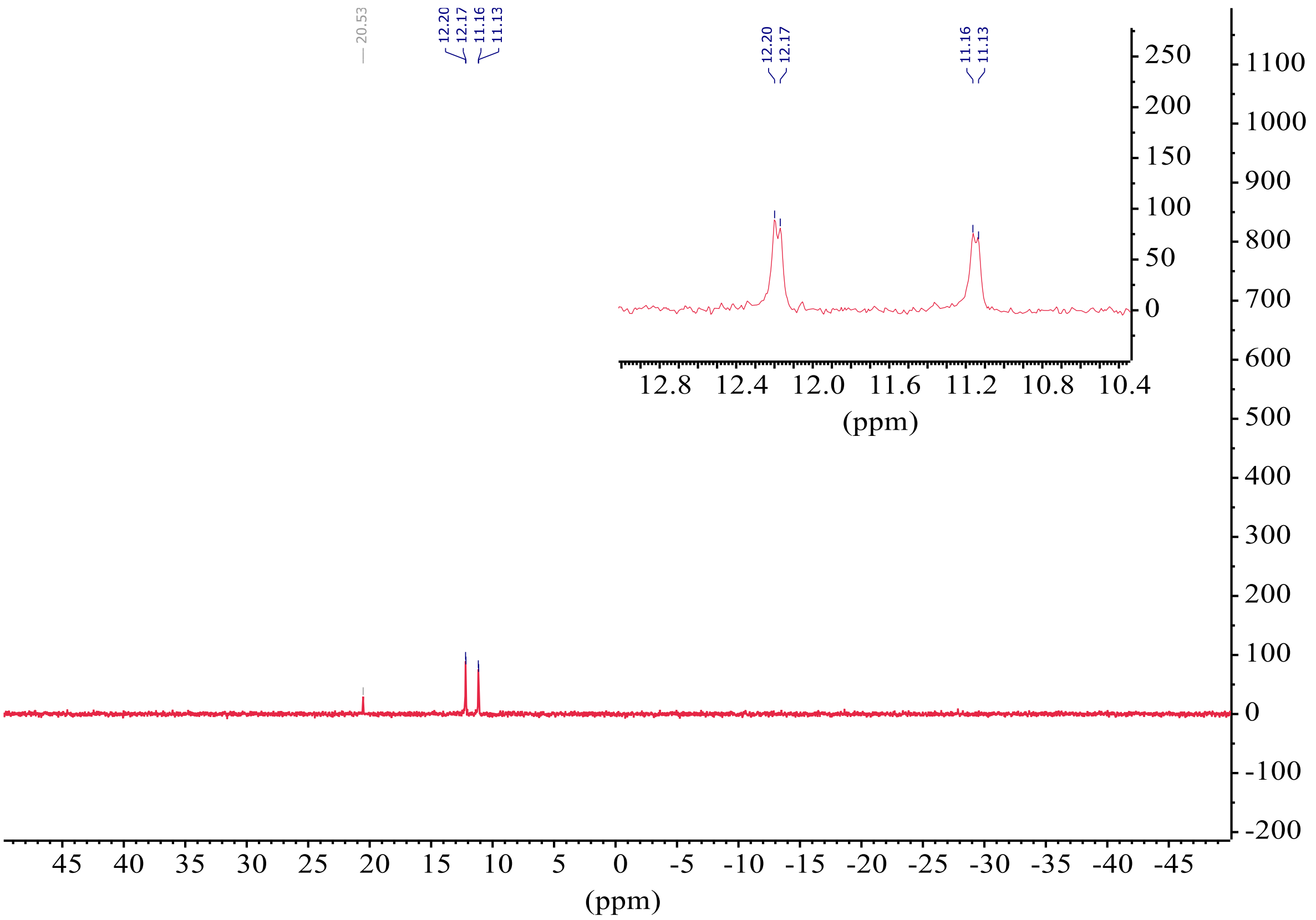

Figure S57: $^{31}P\{^{1}H\}$ NMR (202 MHz) spectrum of **3a-PMes₃** in CD_2Cl_2.

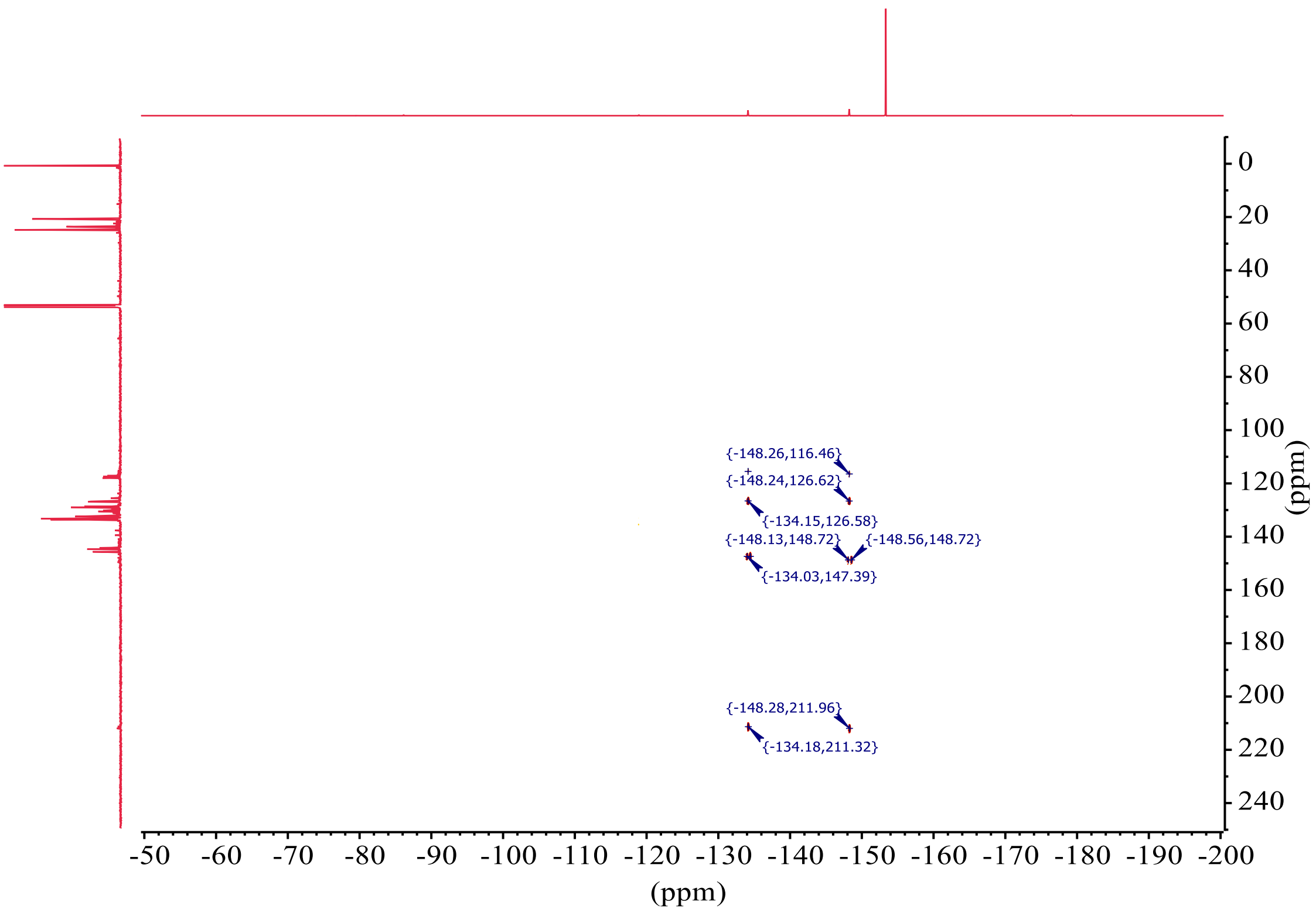

Figure S58: ^{19}F-^{13}C HMBC NMR (565 MHz-150 MHz) spectrum of **3a-PMes₃** in CD_2Cl_2.

2a-PCy3/3a-PCy3

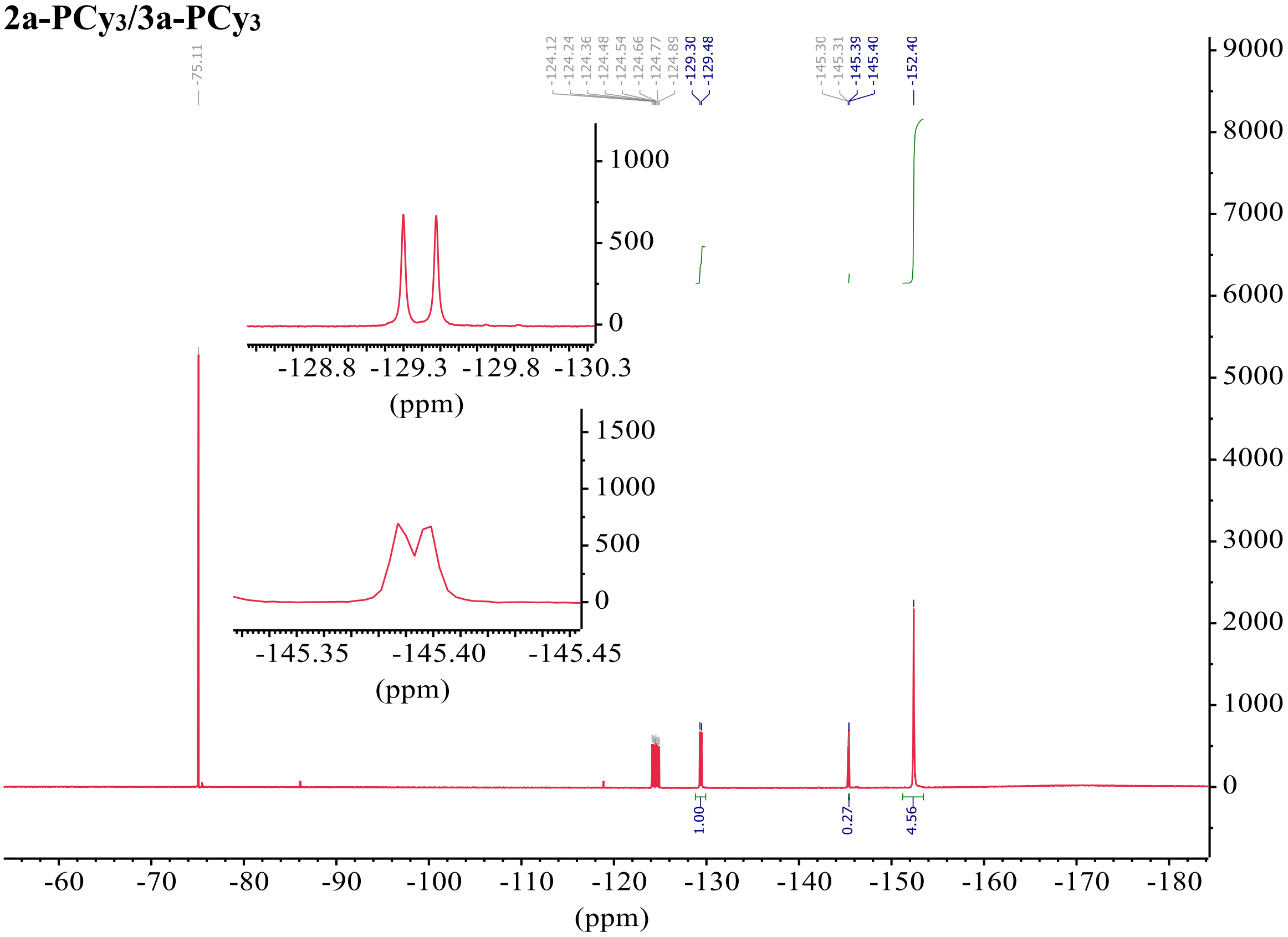

Figure S59: $^{19}F\{^{1}H\}$ NMR (470 MHz) spectrum of **2a-PCy3/3a-PCy3** in CH_2Cl_2. Impurities are marked with grey.

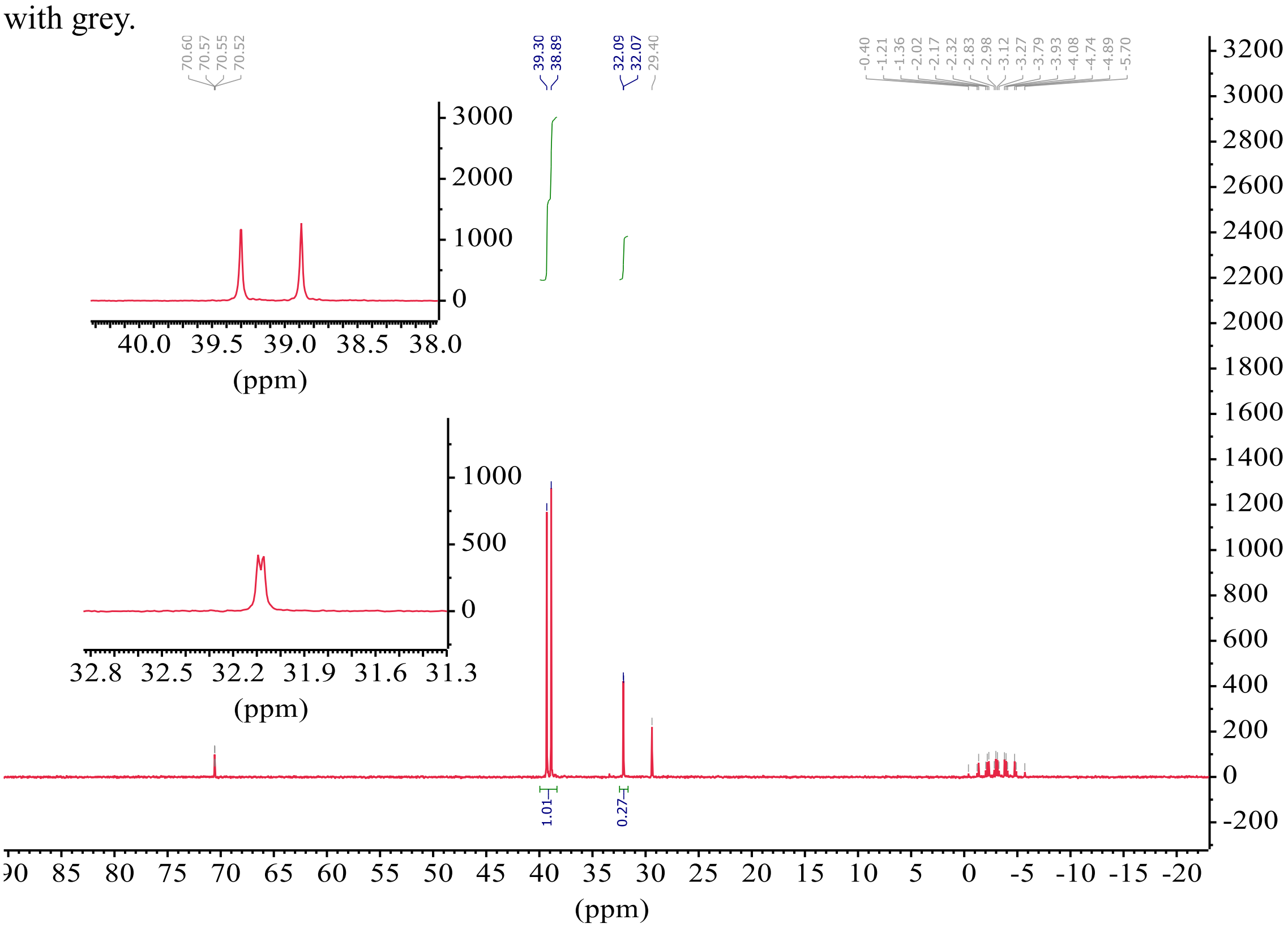

Figure S60: $^{31}P\{^{1}H\}$ NMR (202 MHz) spectrum of **2a-PCy3/3a-PCy3** in CH_2Cl_2. Impurities are marked with grey.

3b-PTol₃

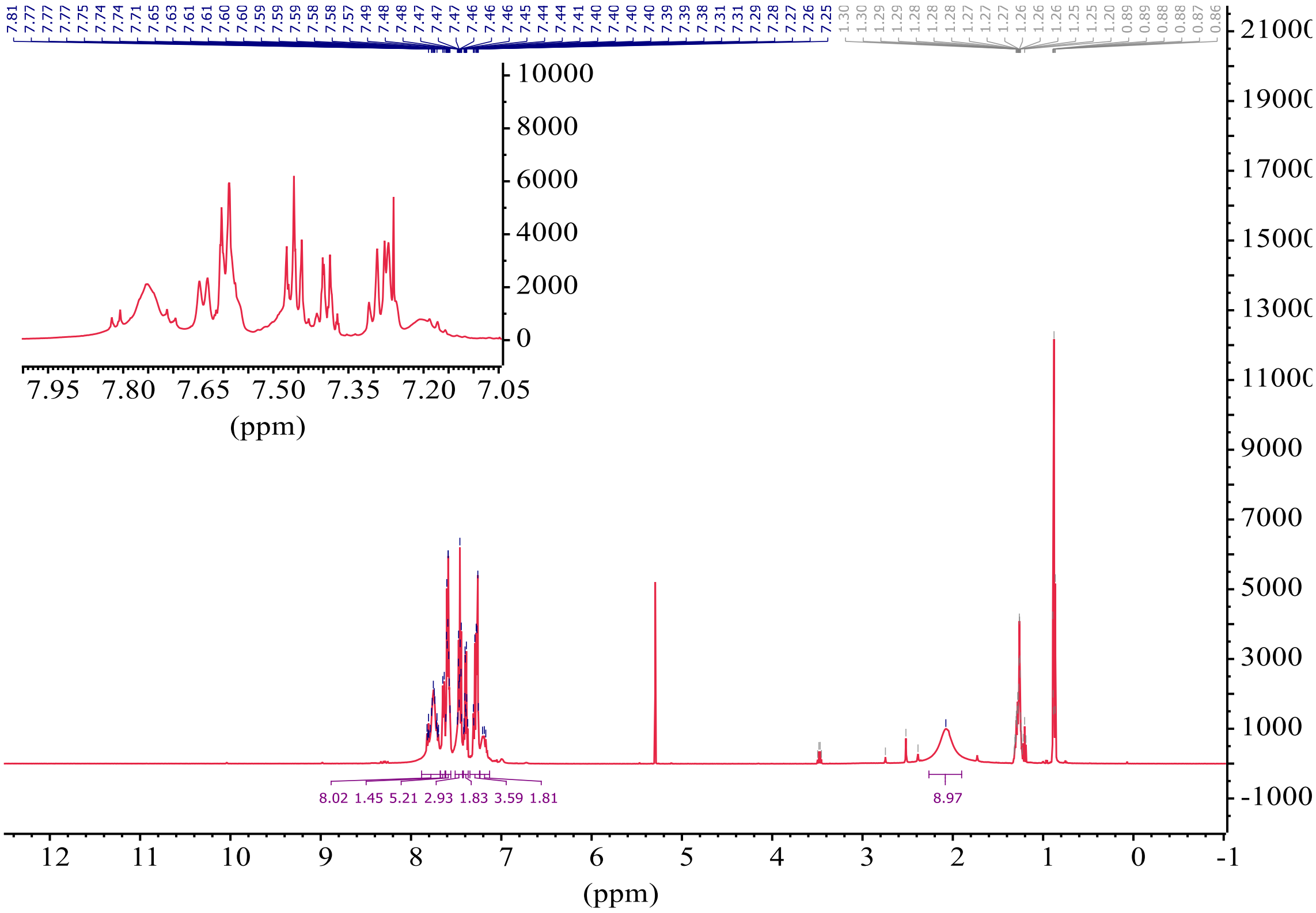

Figure S61: ^{1}H NMR (500 MHz) spectrum of **3b-PTol₃** in $CDCl_3$. Residual solvent impurities are marked with grey ticks.

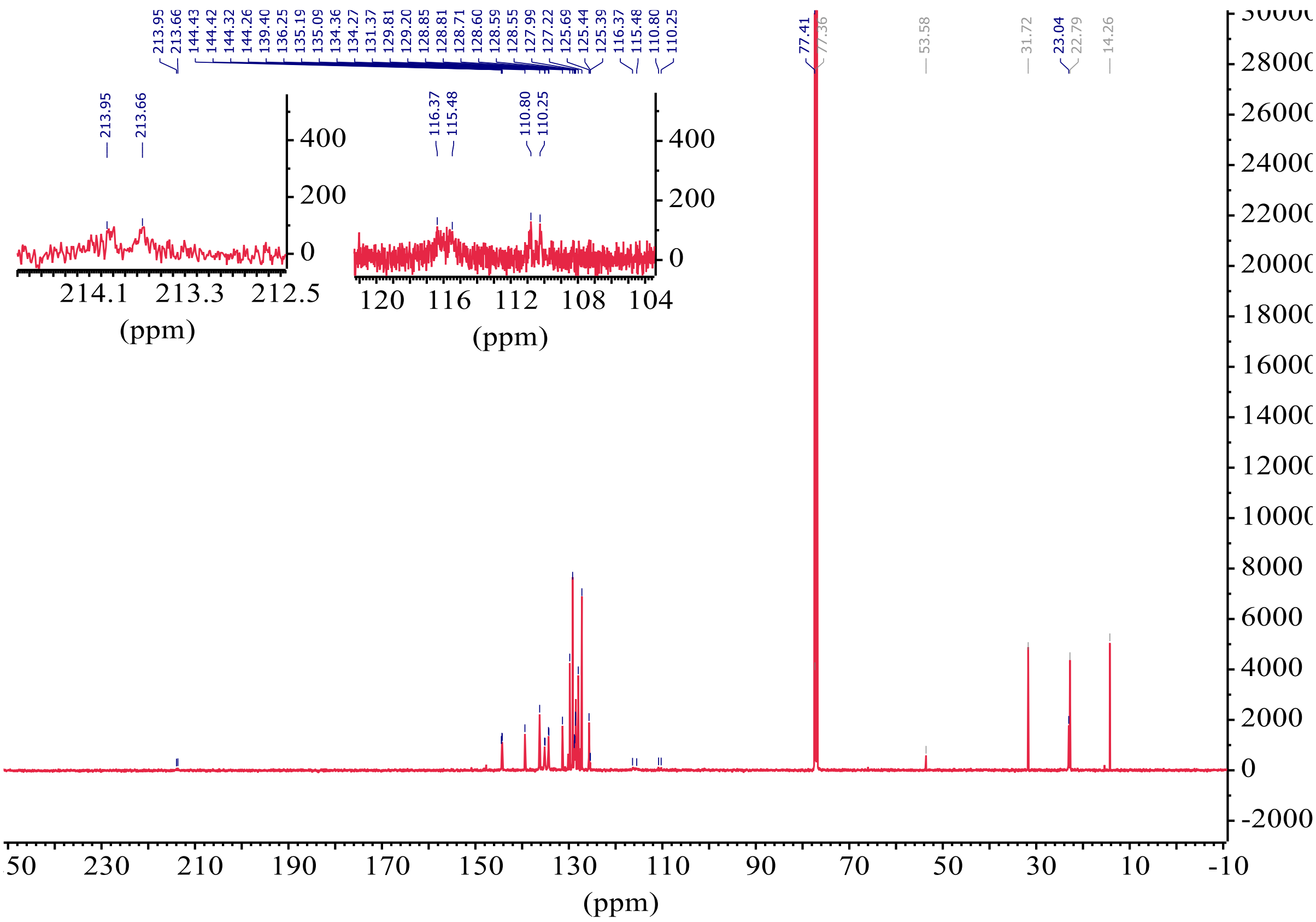

Figure S62: $^{13}C\{^{1}H\}$ NMR (125 MHz) spectrum of **3b-PTol₃** in $CDCl_3$.

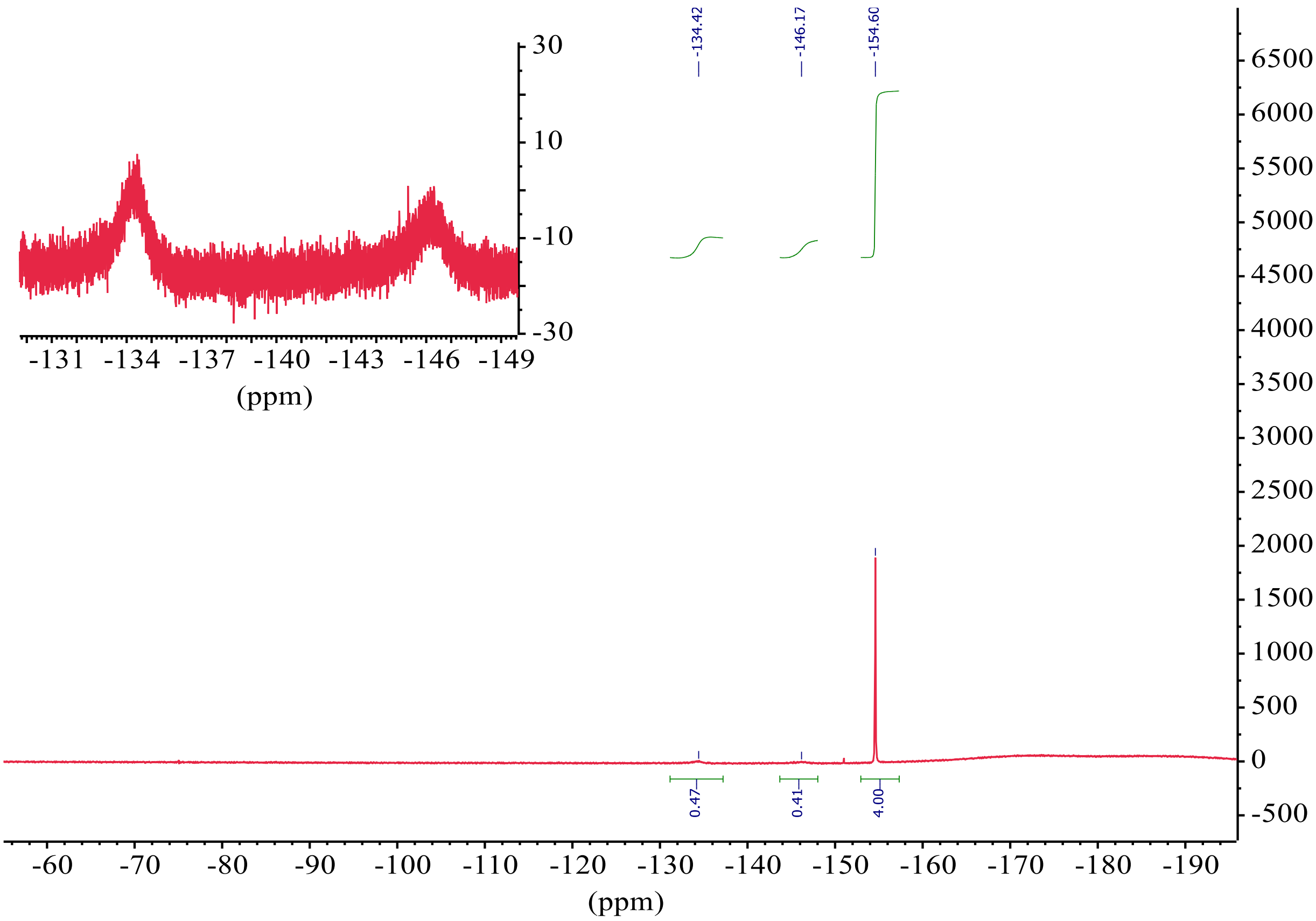

Figure S63: $^{19}F\{^{1}H\}$ NMR (470 MHz) spectrum of **3b-PTol₃** in $CDCl_3$.

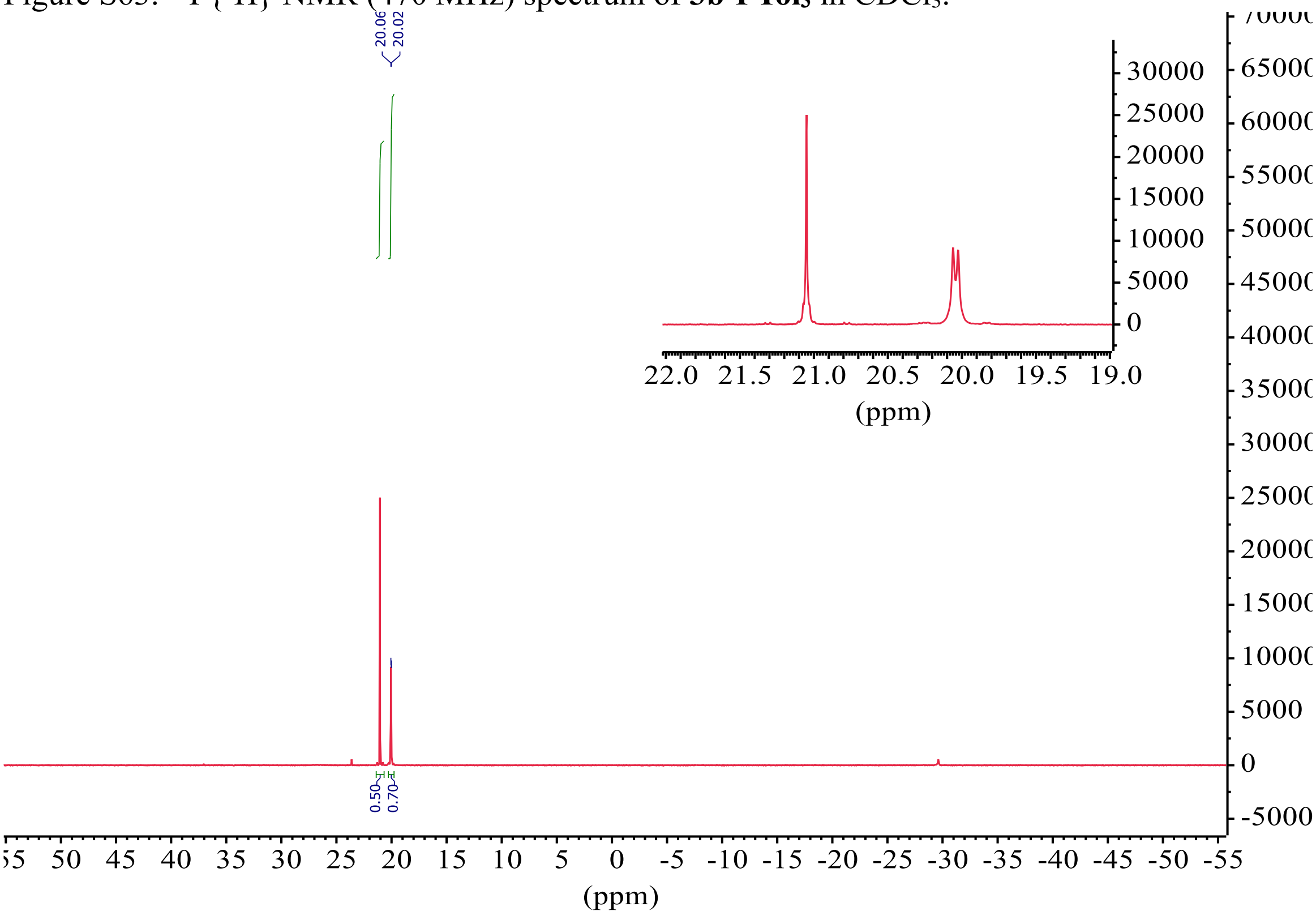

Figure S64: $^{31}P\{^{1}H\}$ NMR (202 MHz) spectrum of **3b-PTol₃** in $CDCl_3$.

3d-PTol₃

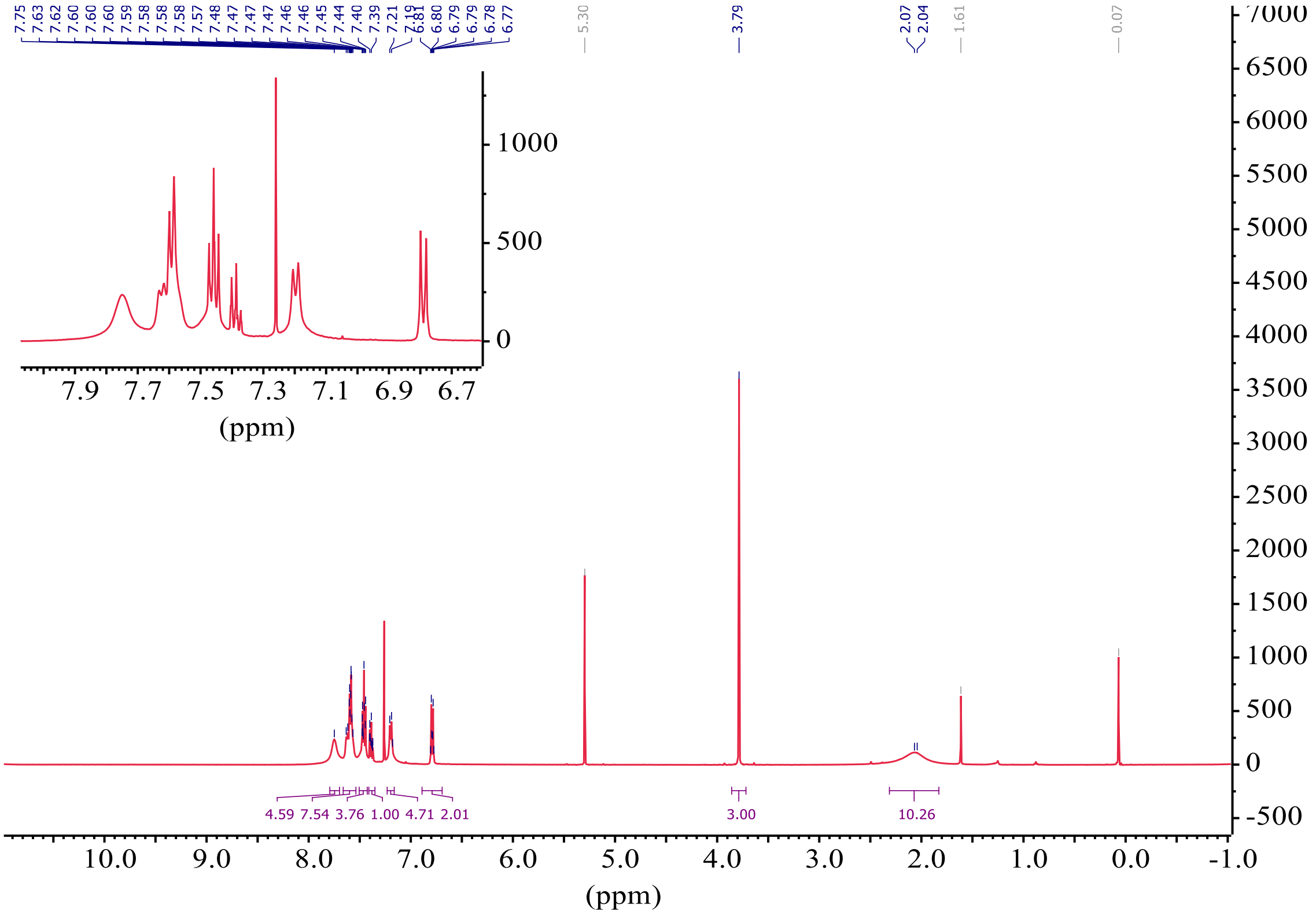

Figure S65: ^{1}H NMR (500 MHz) spectrum of **3d-PTol$_3$** in $CDCl_3$. Residual solvent impurities are marked with grey ticks.

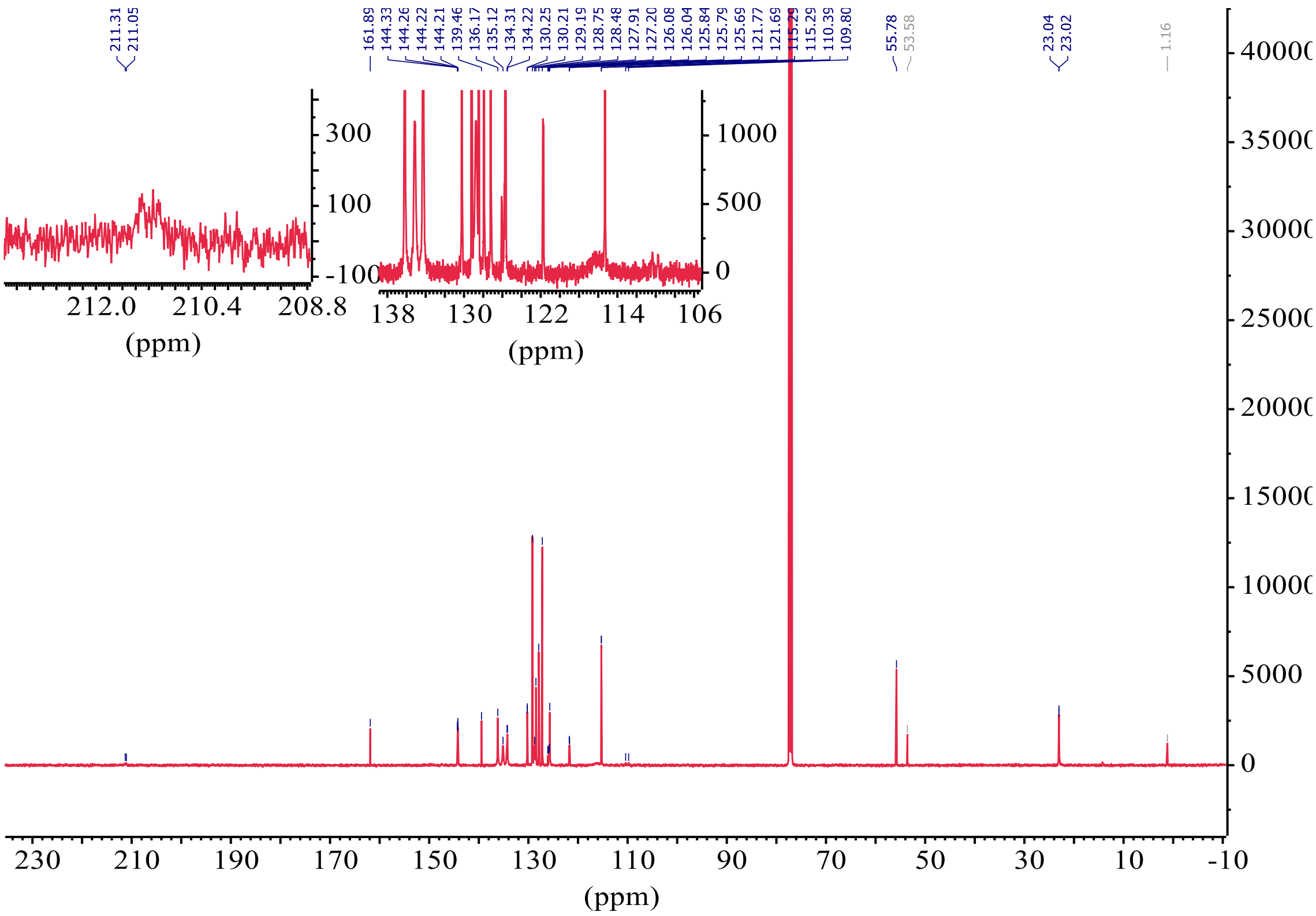

Figure S66: ^{13}C{^{1}H} NMR (125 MHz) spectrum of **3d-PTol$_3$** in $CDCl_3$.

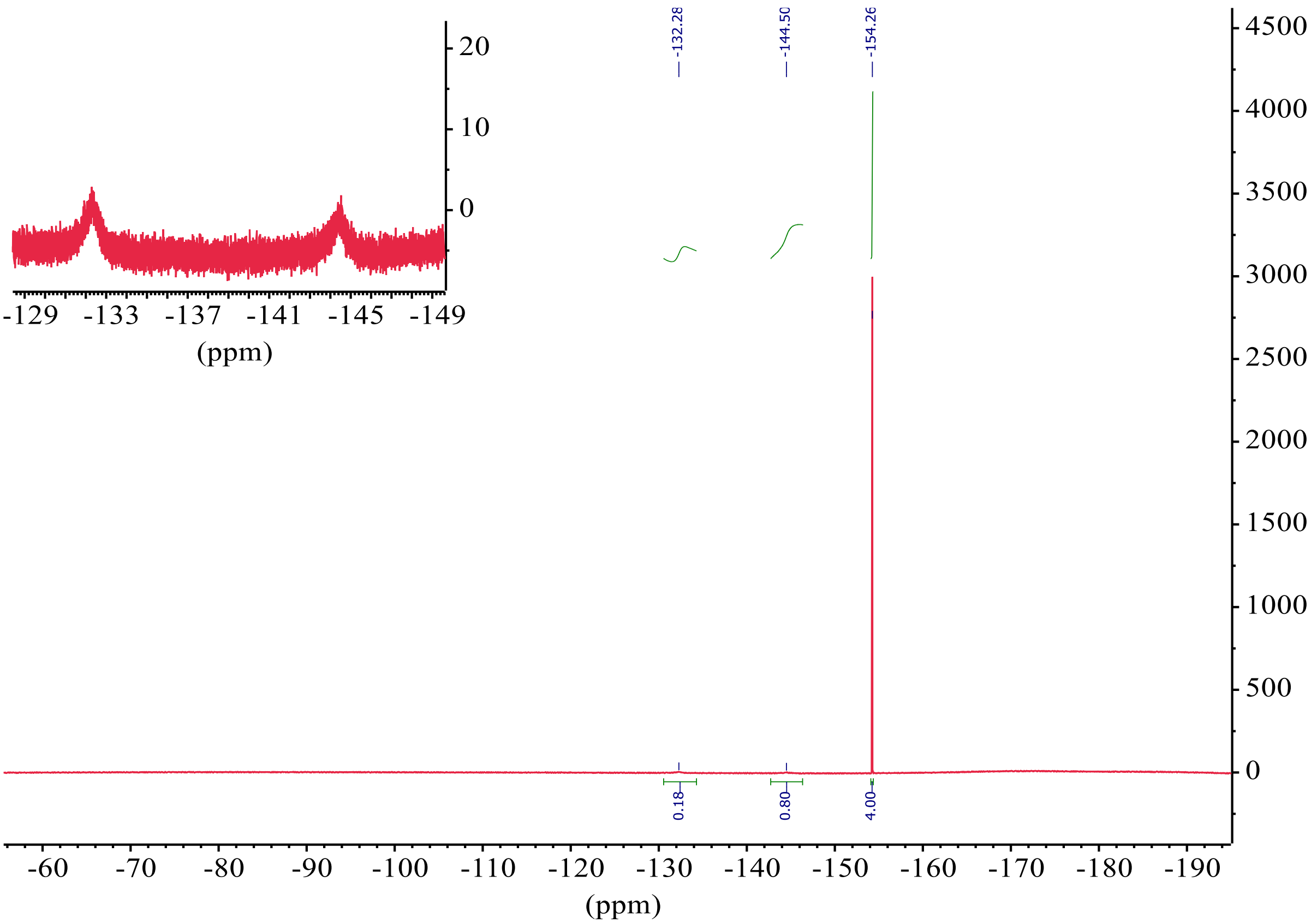

Figure S67: $^{19}F\{^1H\}$ NMR (470 MHz) spectrum of **3d-PTol₃** in $CDCl_3$.

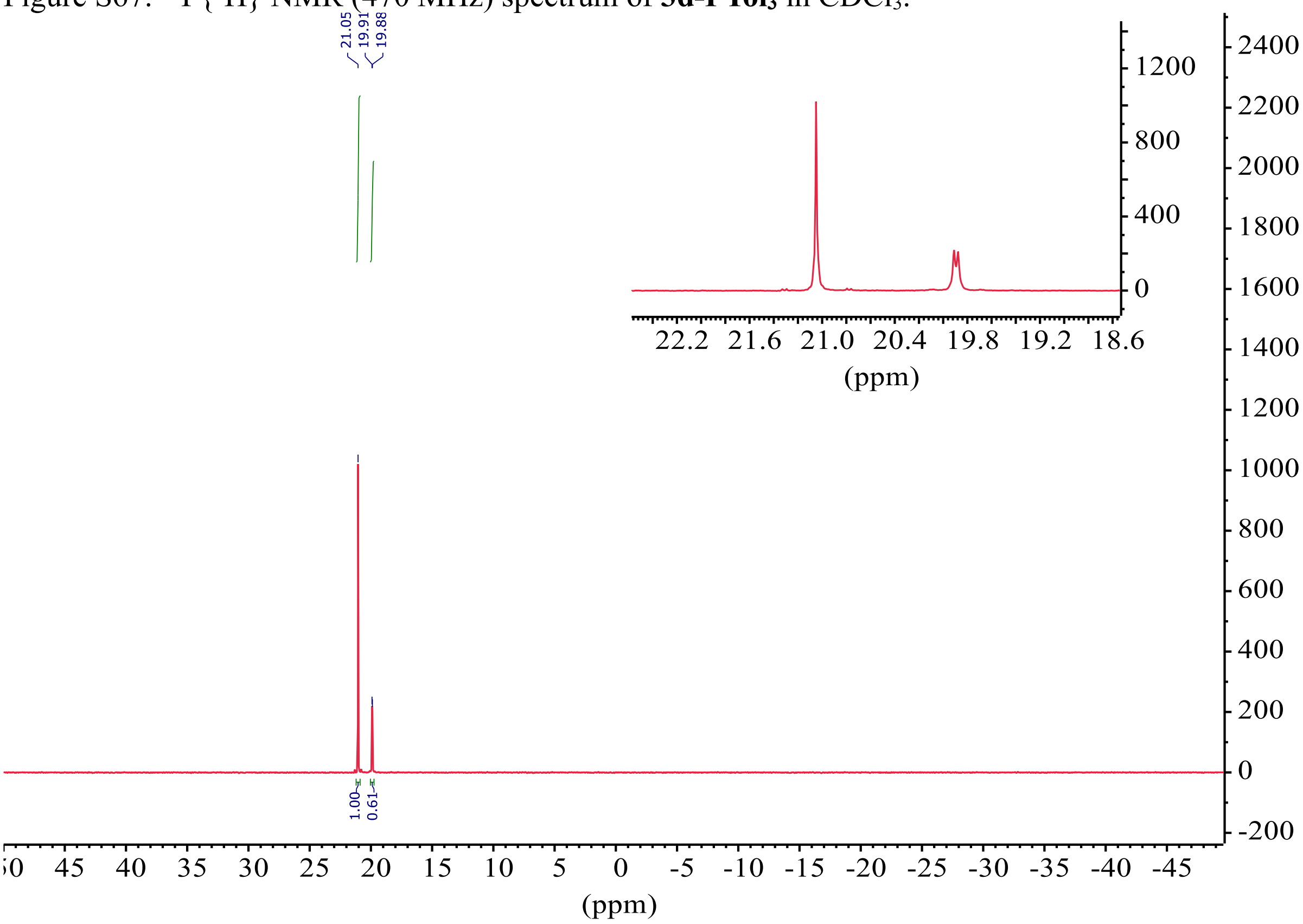

Figure S68: $^{31}P\{^1H\}$ NMR (202 MHz) spectrum of **3d-PTol₃** in $CDCl_3$.

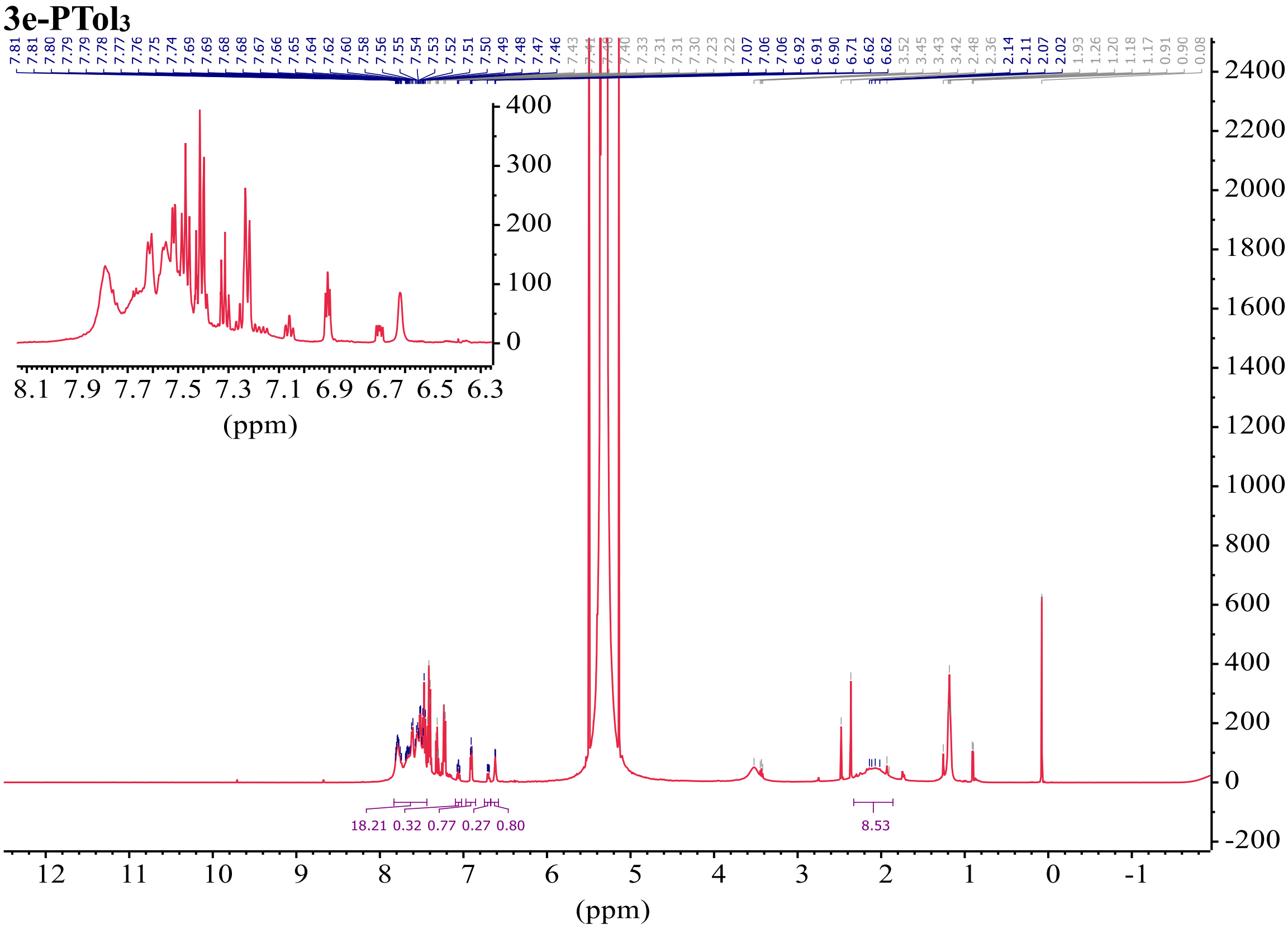

Figure S69: ^{1}H NMR (500 MHz) spectrum of **3e-PTol$_3$** in CH_2Cl_2. Residual solvent impurities and internal standard are marked with grey ticks.

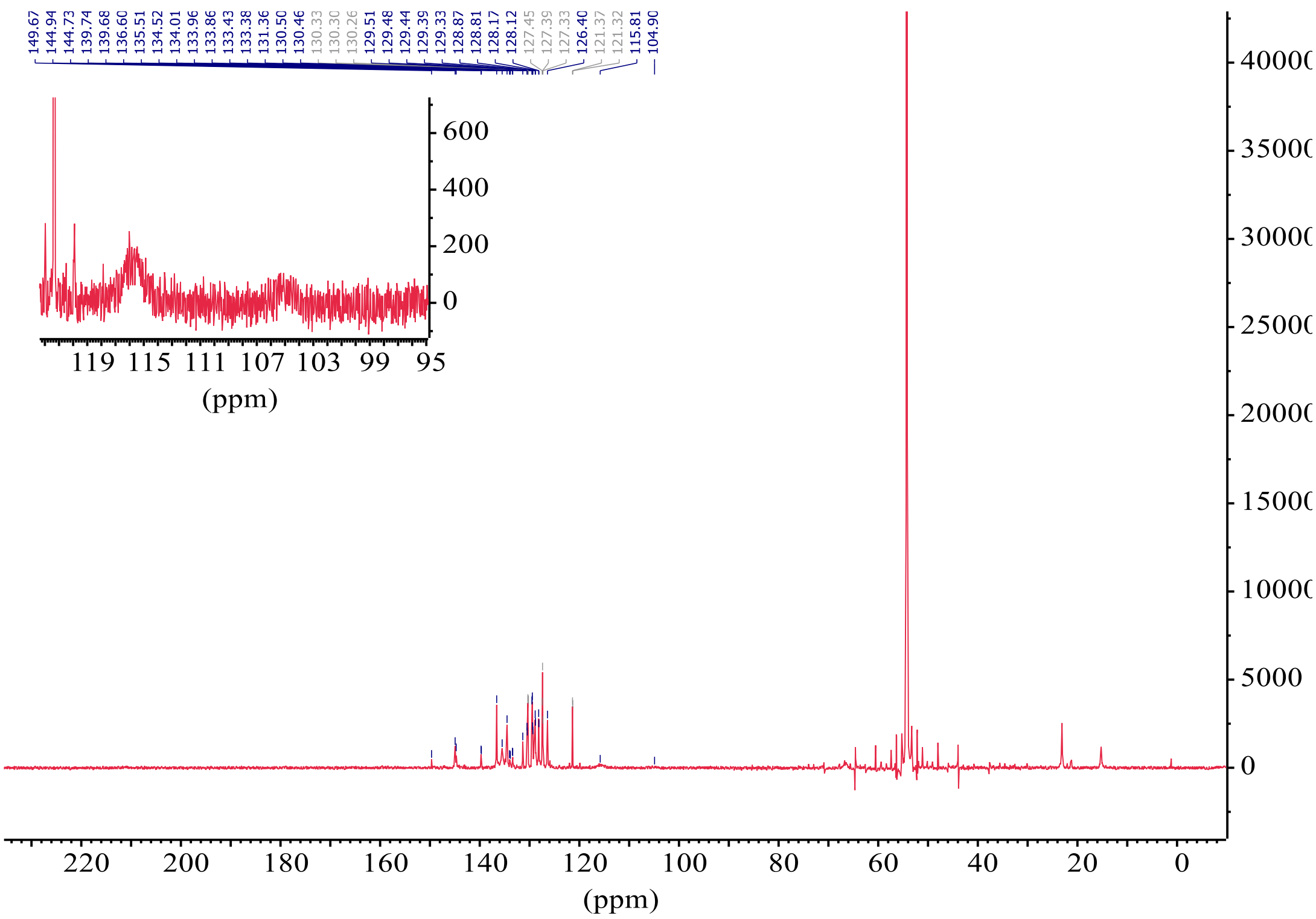

Figure S70: ^{13}C{^{1}H} NMR (125 MHz) spectrum of **3e-PTol$_3$** in CH_2Cl_2.

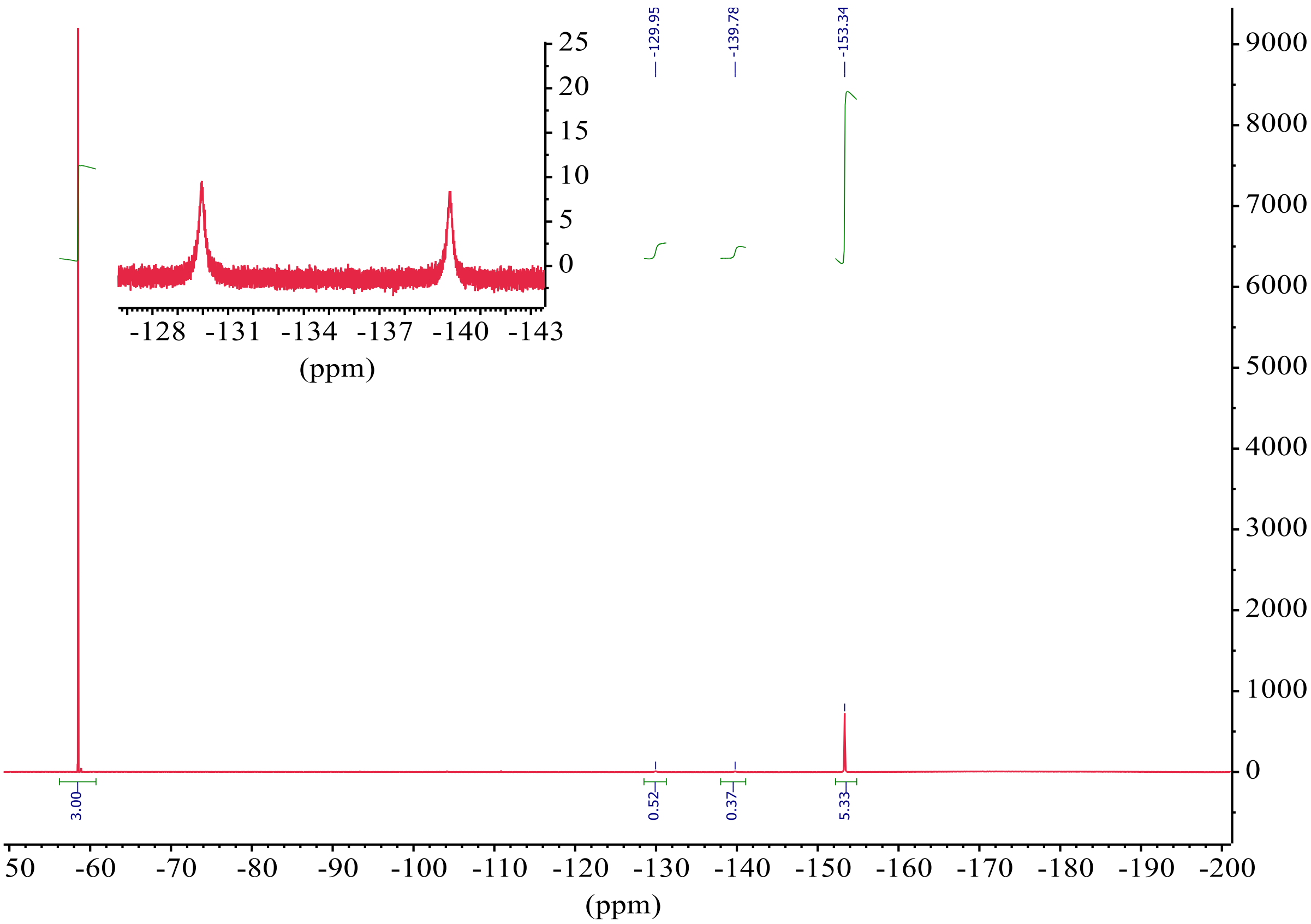

Figure S71: $^{19}F\{^{1}H\}$ NMR (470 MHz) spectrum of **3e-PTol₃** in CH_2Cl_2.

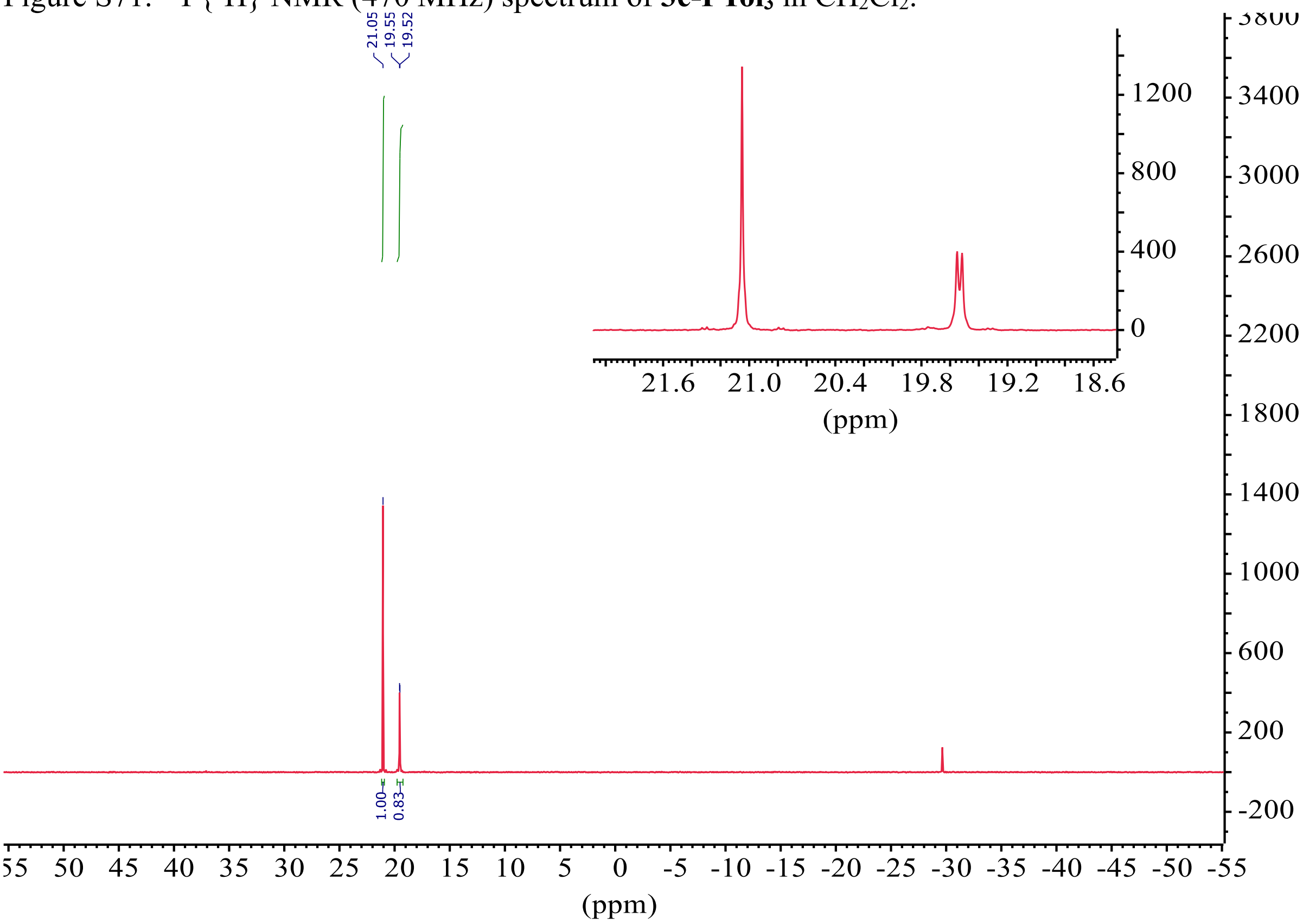

Figure S72: $^{31}P\{^{1}H\}$ NMR (202 MHz) spectrum of **3e-PTol₃** in CH_2Cl_2.

3f-PTol$_3$

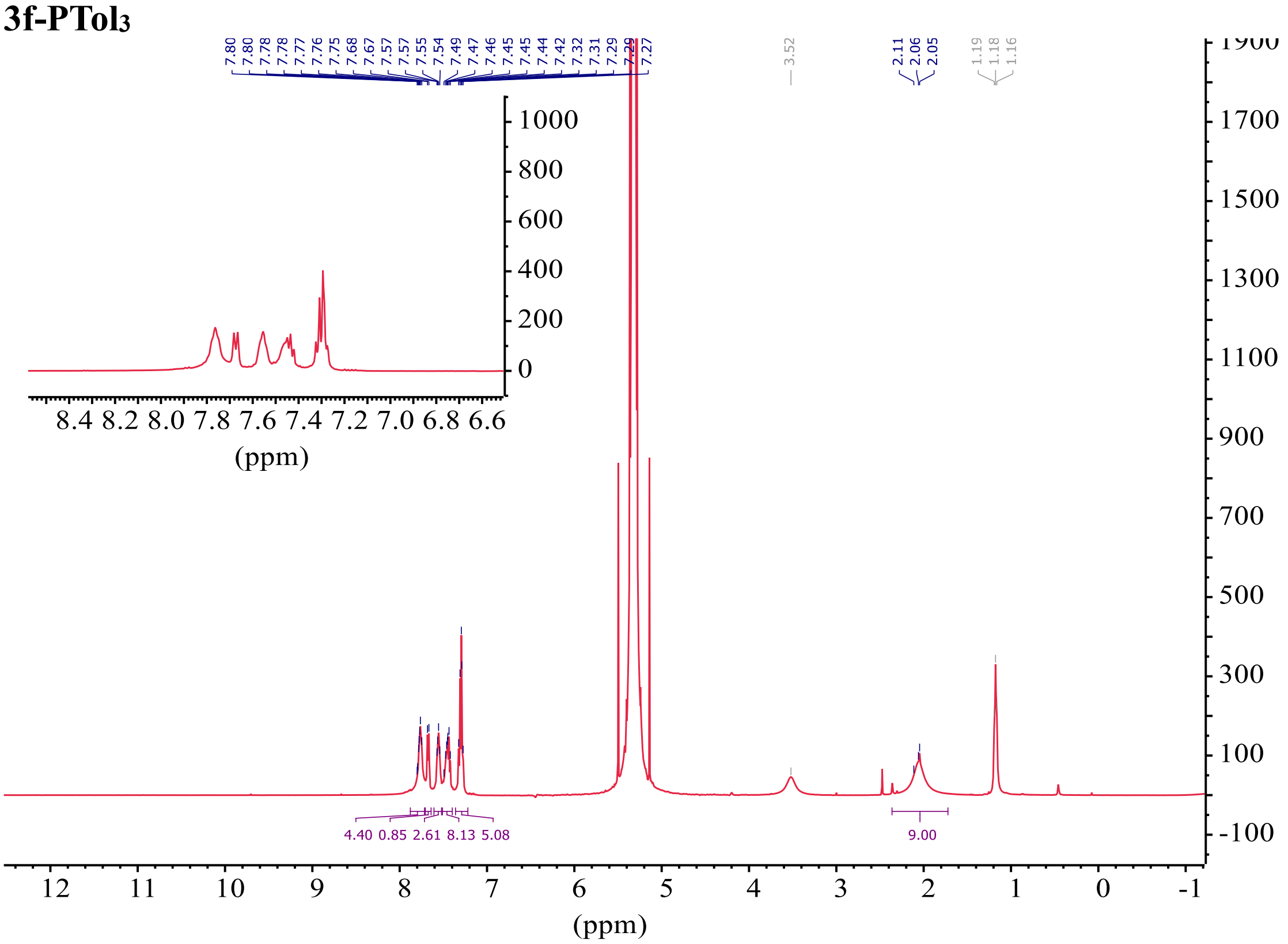

Figure S73: ^{1}H NMR (500 MHz) spectrum of **3f-PTol$_3$** in CH_2Cl_2. Residual solvent impurities are marked with grey ticks.

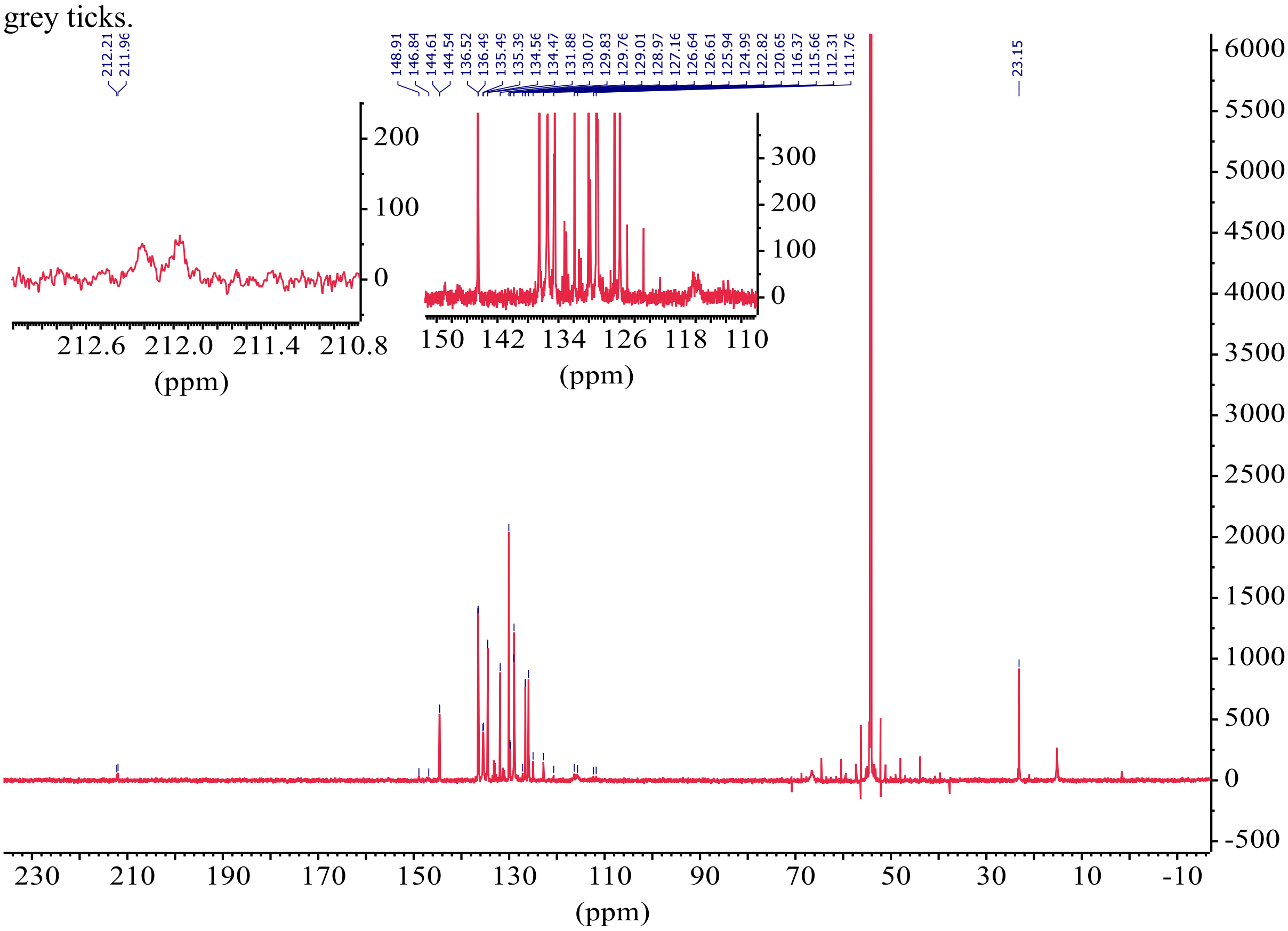

Figure S74: $^{13}C\{^1H\}$ NMR (125 MHz) spectrum of **3f-PTol$_3$** in CH_2Cl_2.

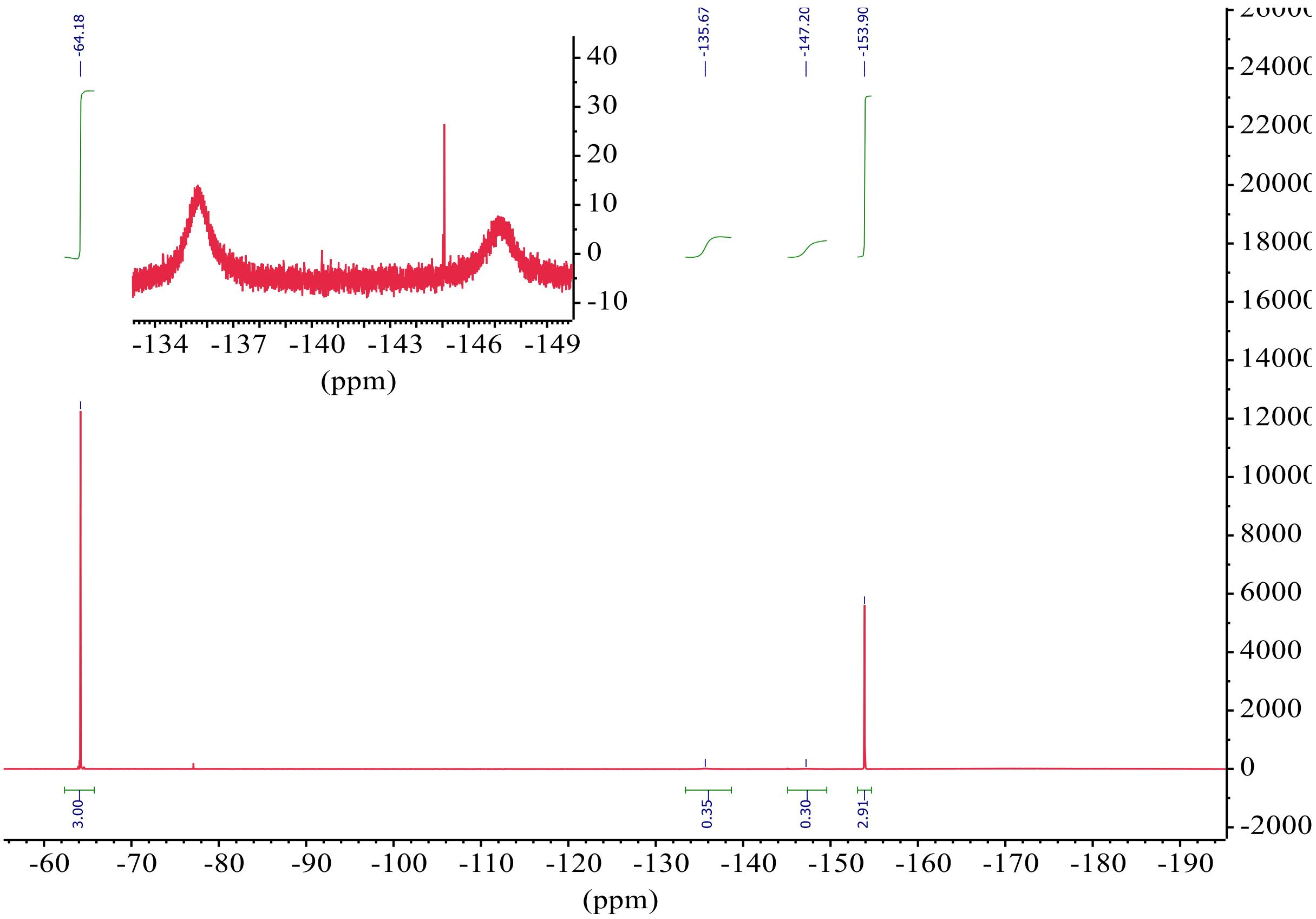

Figure S75: $^{19}F\{^{1}H\}$ NMR (470 MHz) spectrum of **3f-PTol₃** in CH_2Cl_2.

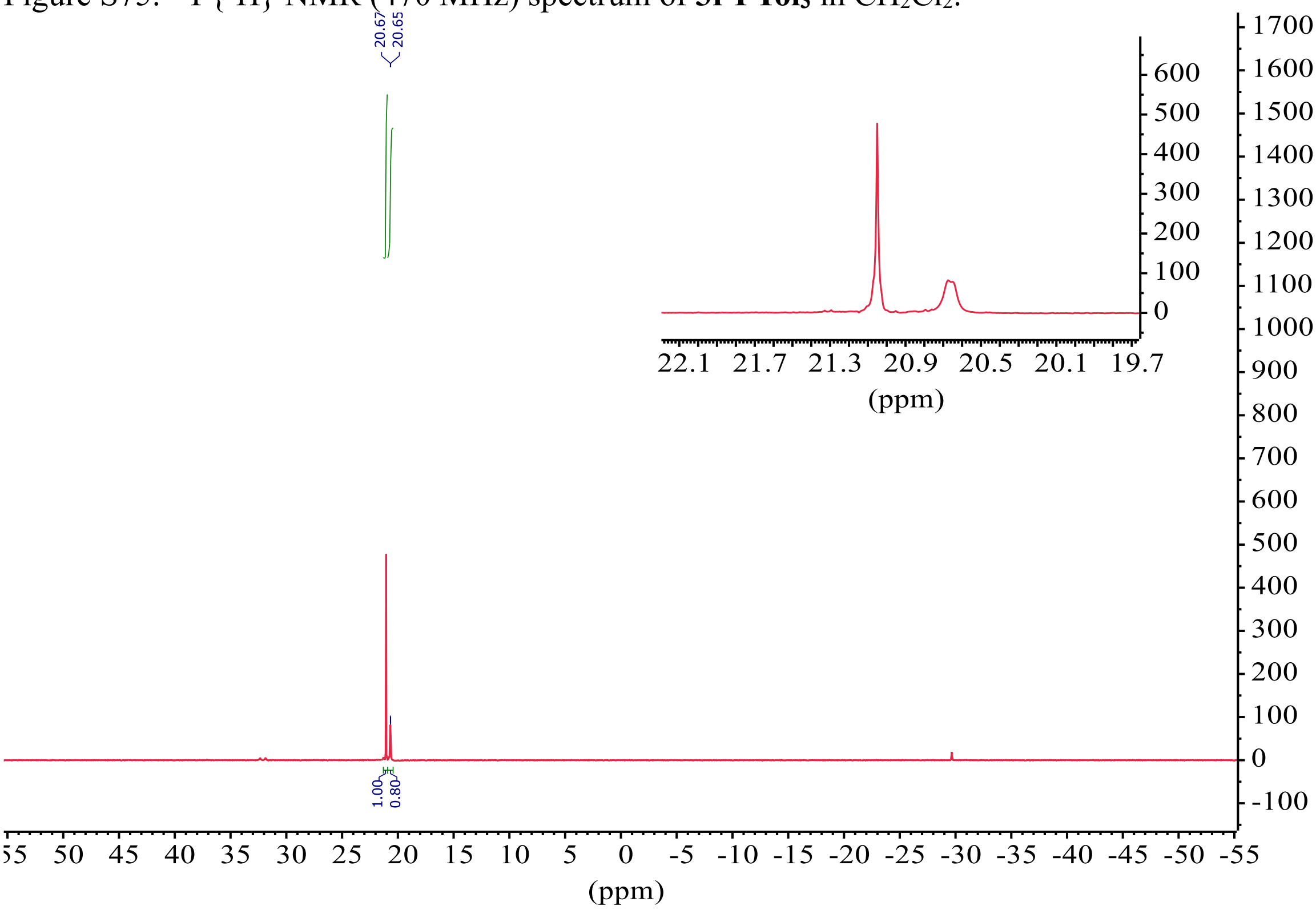

Figure S76: $^{31}P\{^{1}H\}$ NMR (202 MHz) spectrum of **3f-PTol₃** in CH_2Cl_3.

3g-PMes$_3$

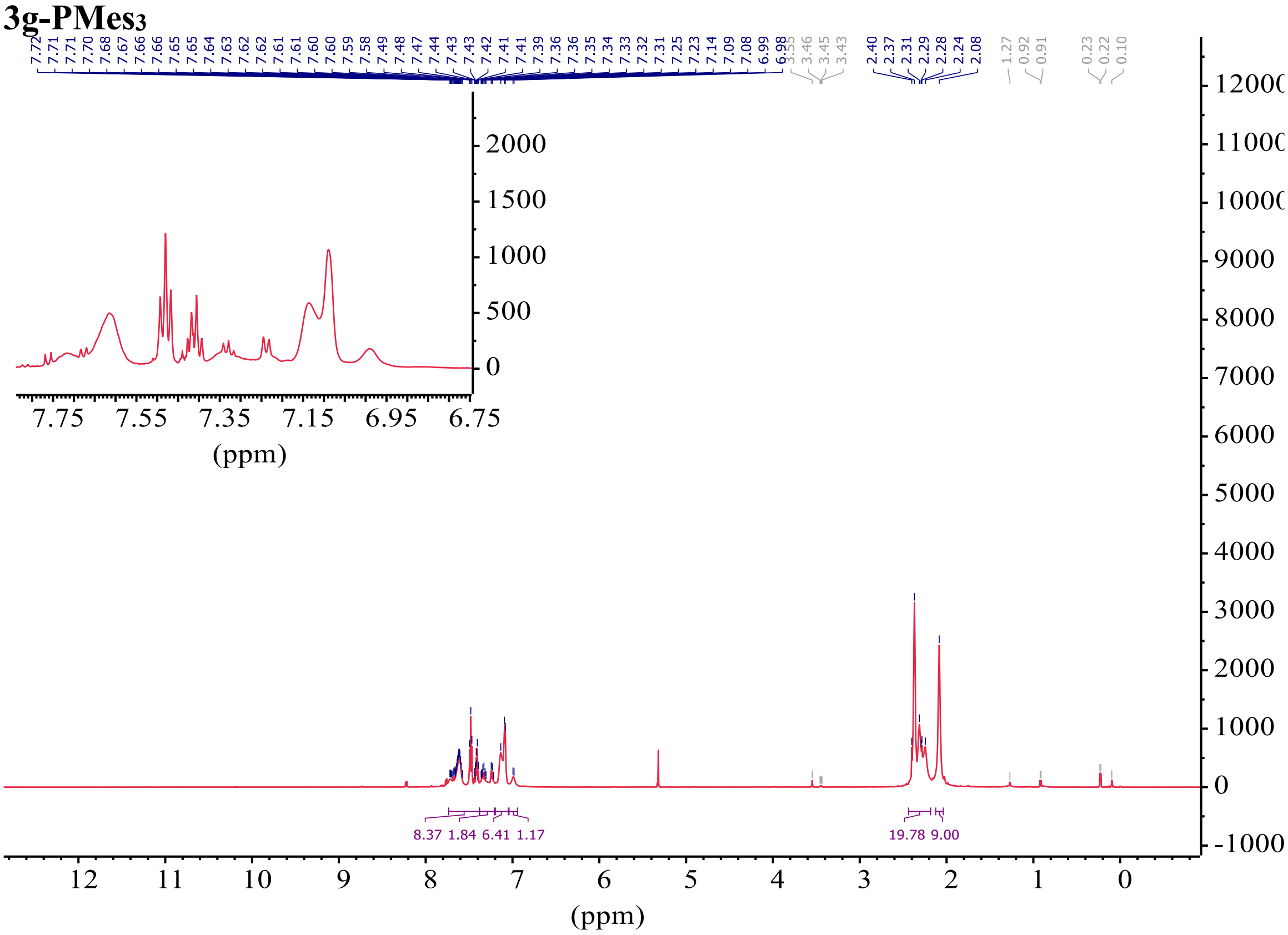

Figure S77: ^{1}H NMR (600 MHz) spectrum of **3g-PMes$_3$** in CD_2Cl_2. Residual solvent impurities are marked with grey ticks.

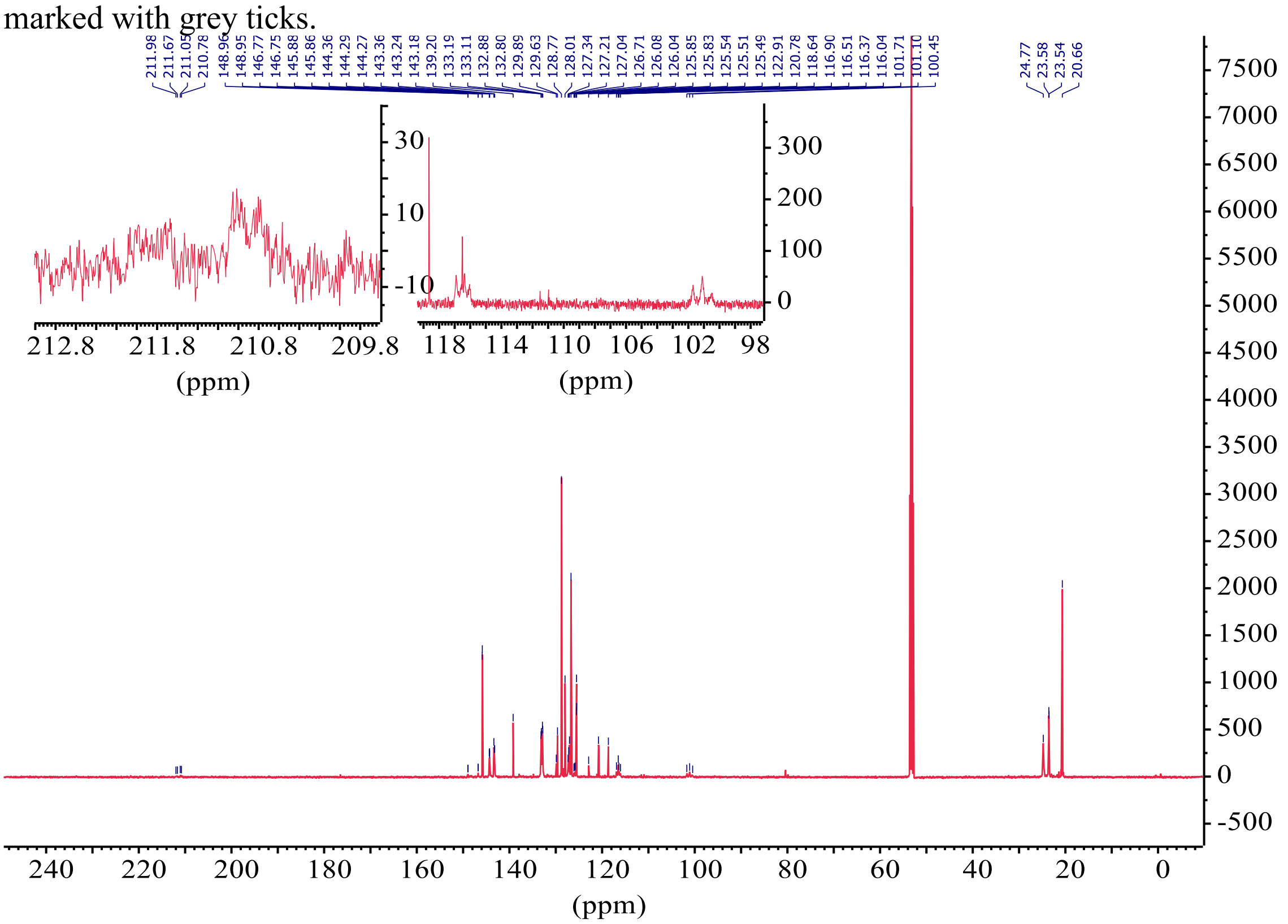

Figure S78: ^{13}C{^{1}H} NMR (150 MHz) spectrum of **3g-PMes$_3$** in CD_2Cl_2.

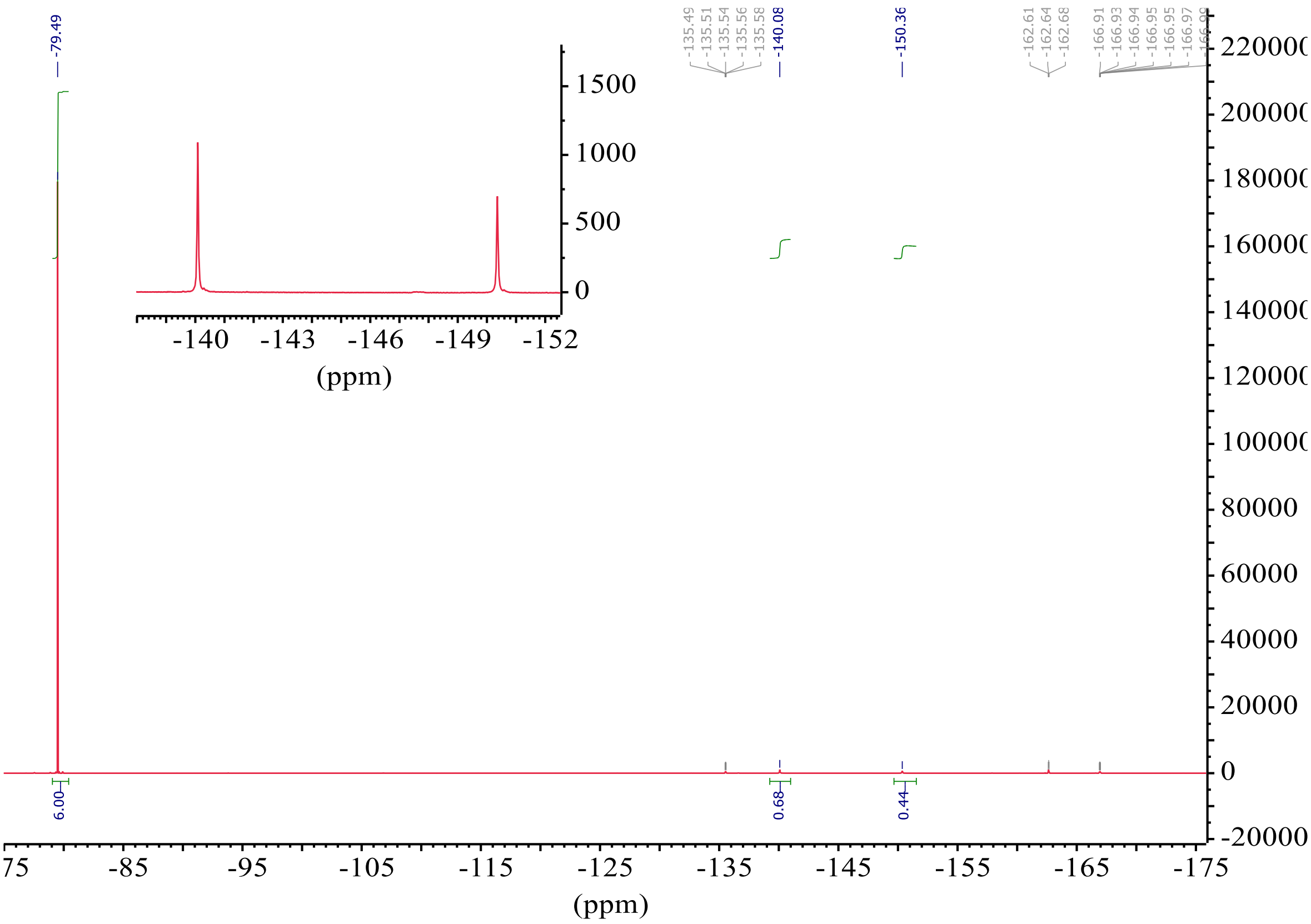

Figure S79: ^{19}F NMR (565 MHz) spectrum of **3g-PMes₃** in CD_2Cl_2. Residual impurities are marked with grey ticks.

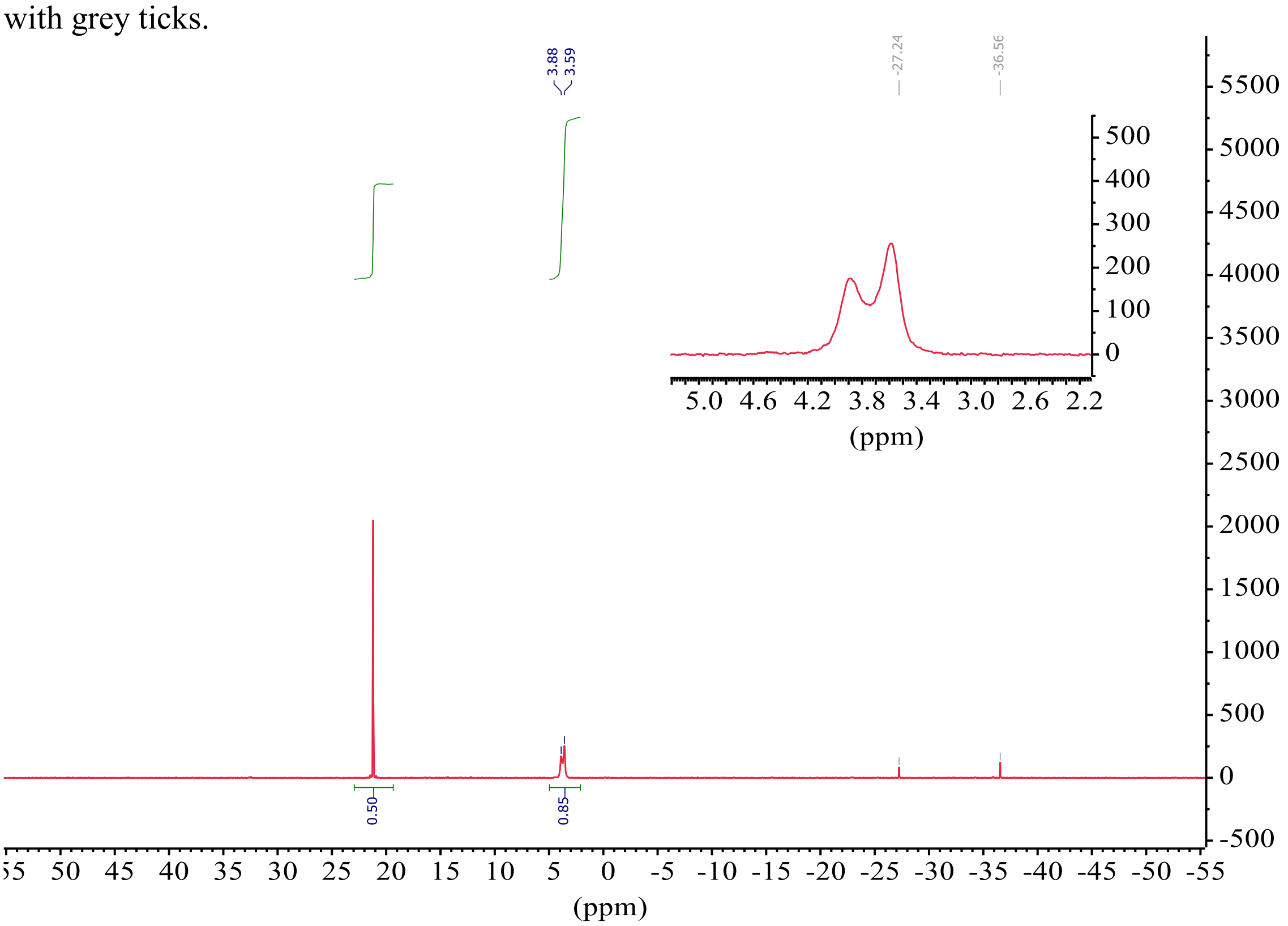

Figure S80: $^{31}P\{^1H\}$ NMR (202 MHz) spectrum of **3g-PMes$_3$** in CD_2Cl_2. Residual impurities are marked with grey ticks.

3g-PTol₃

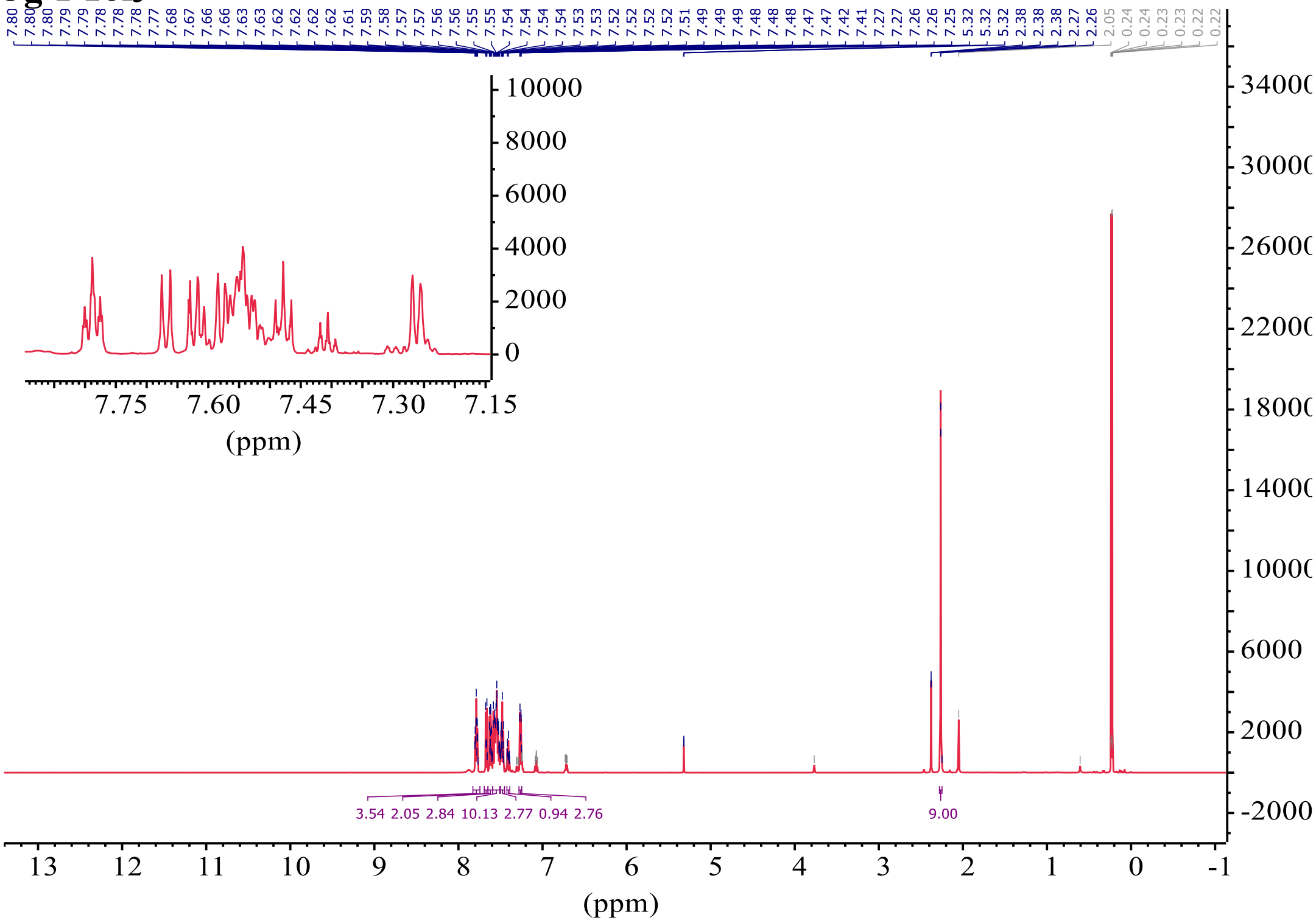

Figure S81: ^{1}H NMR (600 MHz) spectrum of **3g-PTol₃** (10% **2g-PTol₃**) in CD_2Cl_2. Residual impurities are marked with grey ticks.

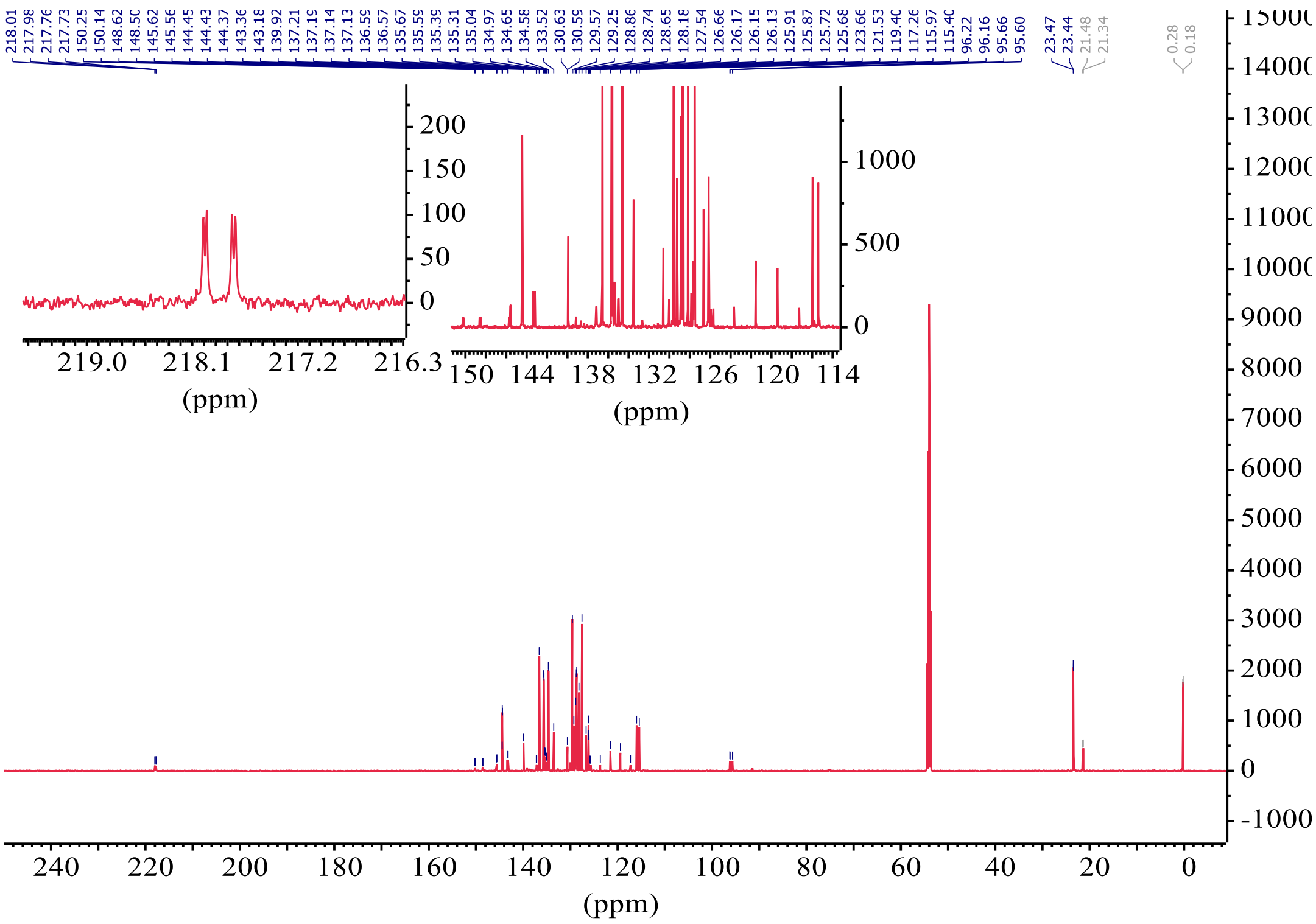

Figure S82: $^{13}C\{^{1}H\}$ NMR (150 MHz) spectrum of **3g-PTol₃** (10% **2g-PTol₃**) in CD_2Cl_2. Residual impurities are marked with grey ticks.

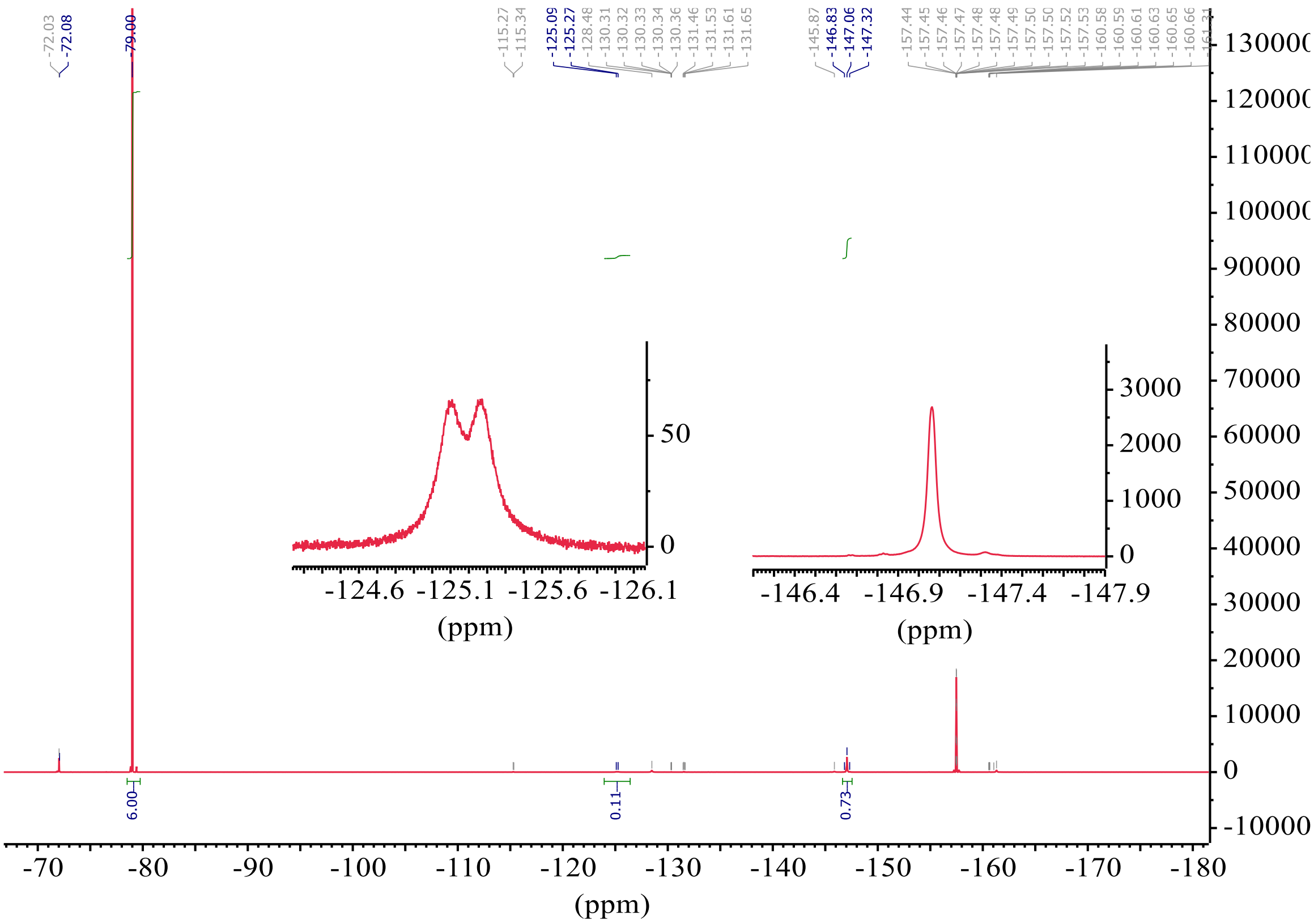

Figure S83: ^{19}F NMR (565 MHz) spectrum of **3g-PTol$_3$** (10% **2g-PTol$_3$**) in CD_2Cl_2. Residual impurities are marked with grey ticks.

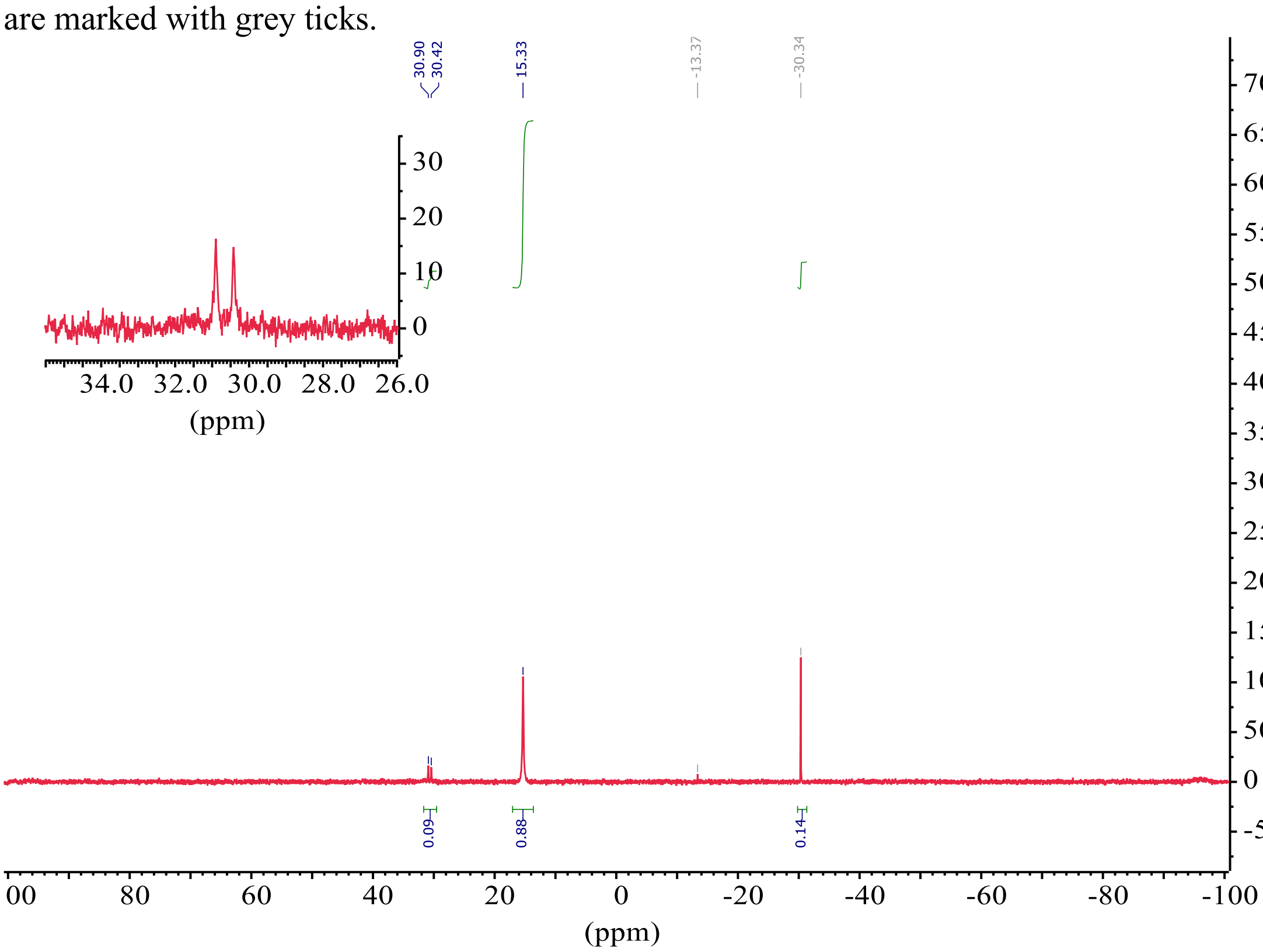

Figure S84: ^{31}P{^{1}H} NMR (202 MHz) spectrum of **3g-PTol$_3$** (10% **2g-PTol$_3$**) in CD_2Cl_2. Residual impurities are marked with grey ticks.

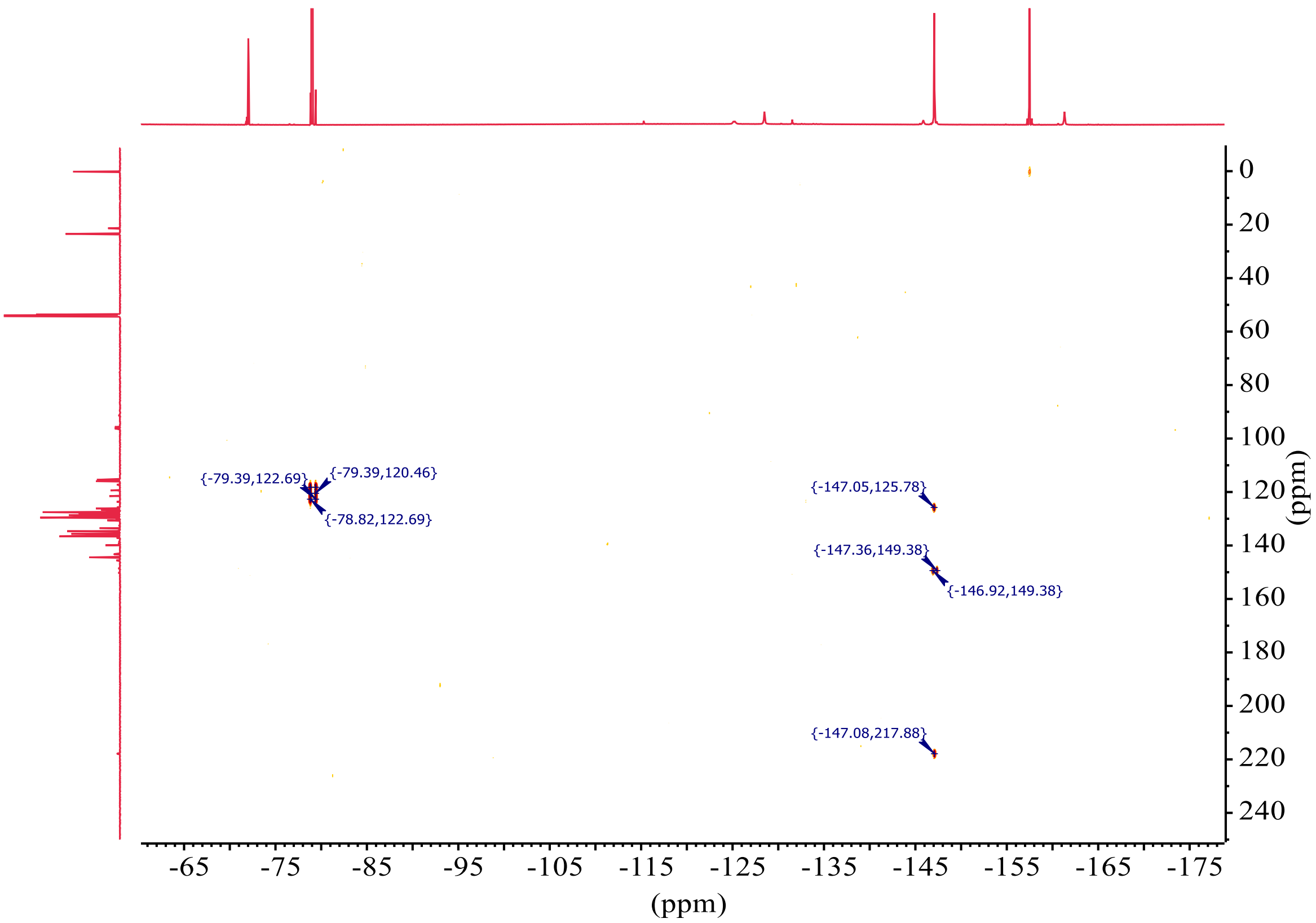

Figure S85: ^{19}F-^{13}C HMBC NMR (565 MHz-150 MHz) spectrum of **3g-PTol₃** (10% **2g-PTol₃**) in CD_2Cl_2.

3h-PMes₃

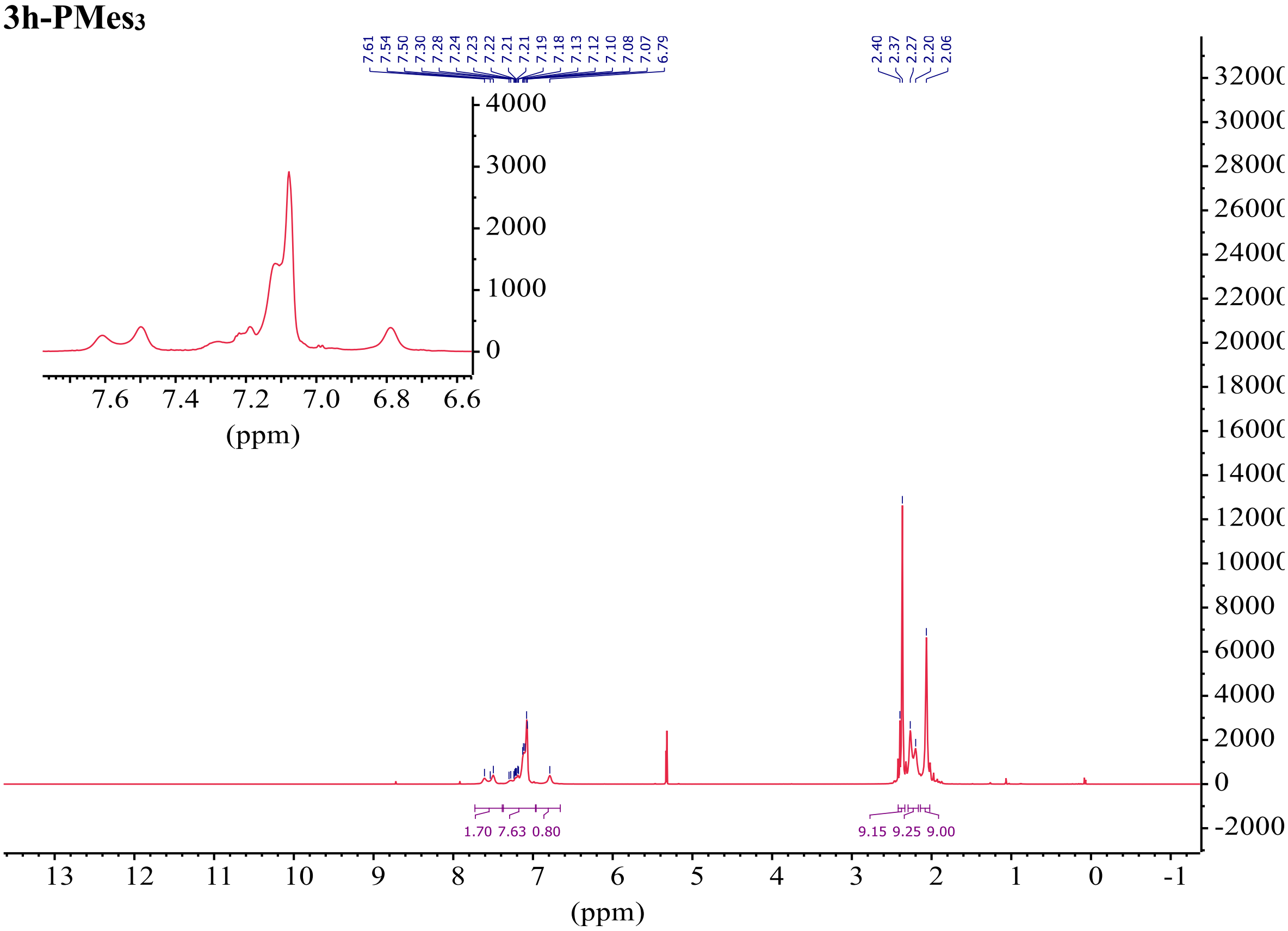

Figure S86: ^{1}H NMR (600 MHz) spectrum of **3h-PMes₃** in CD_2Cl_2.

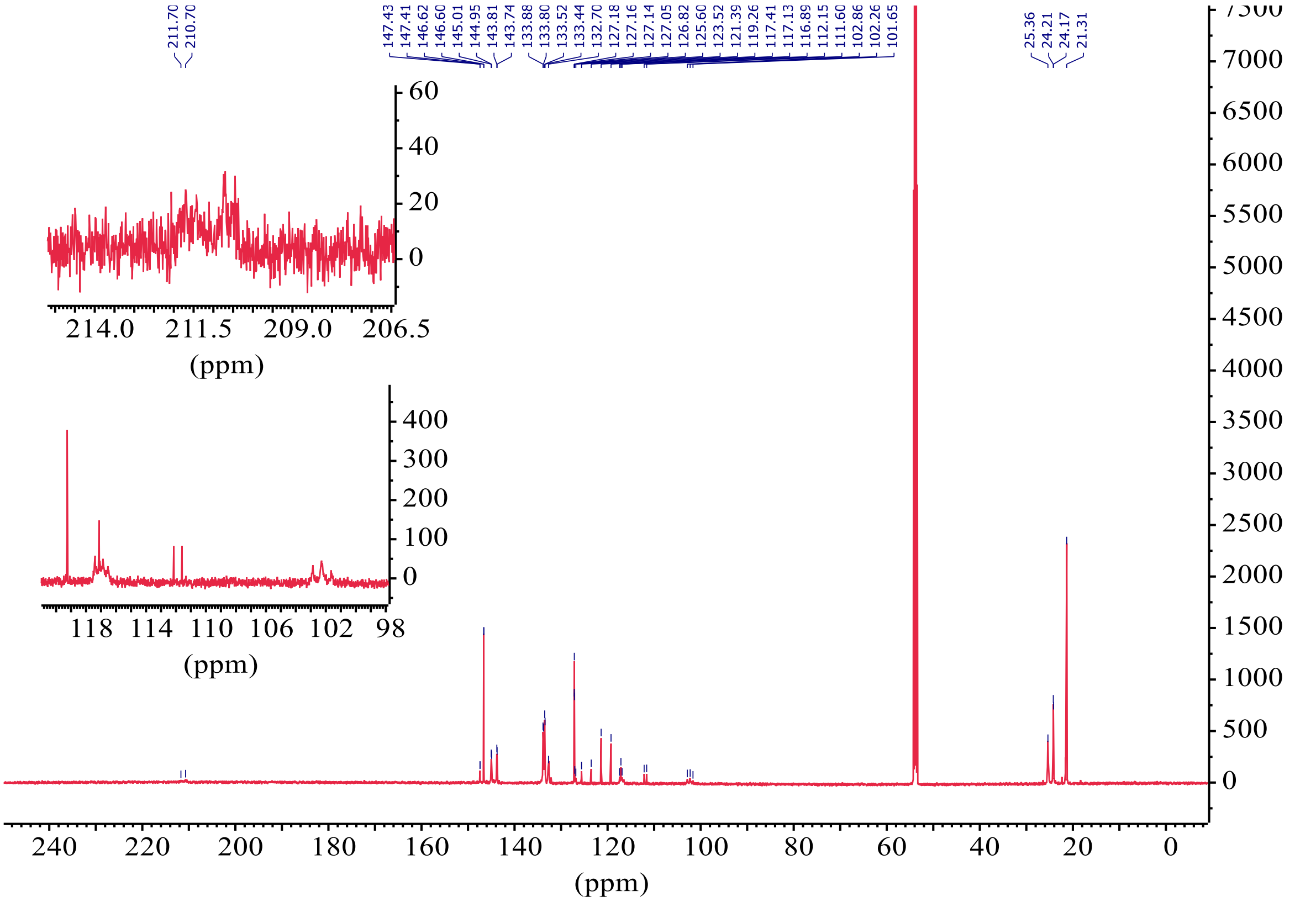

Figure S87: $^{13}C\{^{1}H\}$ NMR (150 MHz) spectrum of **3h-PMes₃** in CD_2Cl_2.

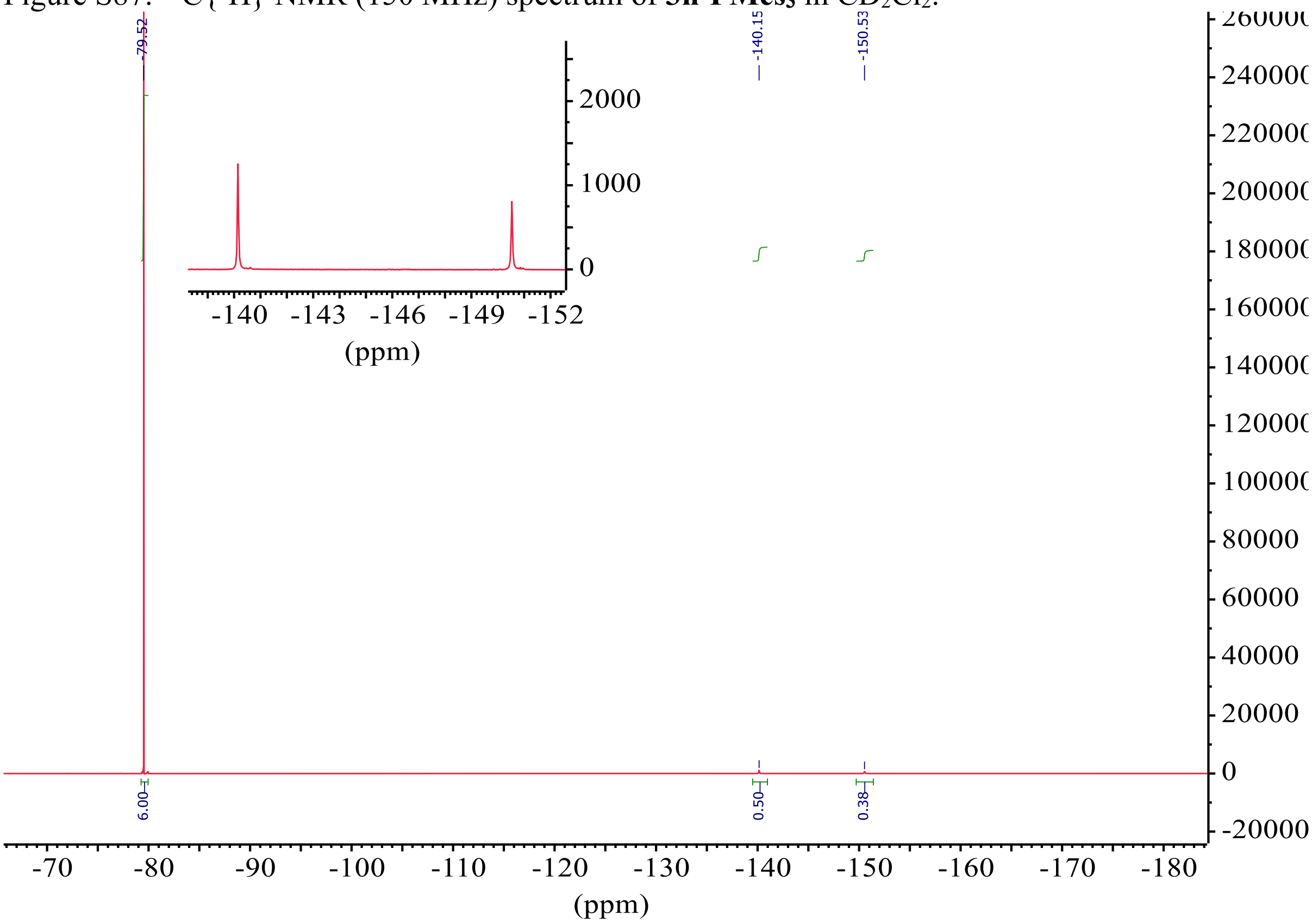

Figure S88: ^{19}F NMR (565 MHz) spectrum of **3h-PMes₃** in CD_2Cl_2.

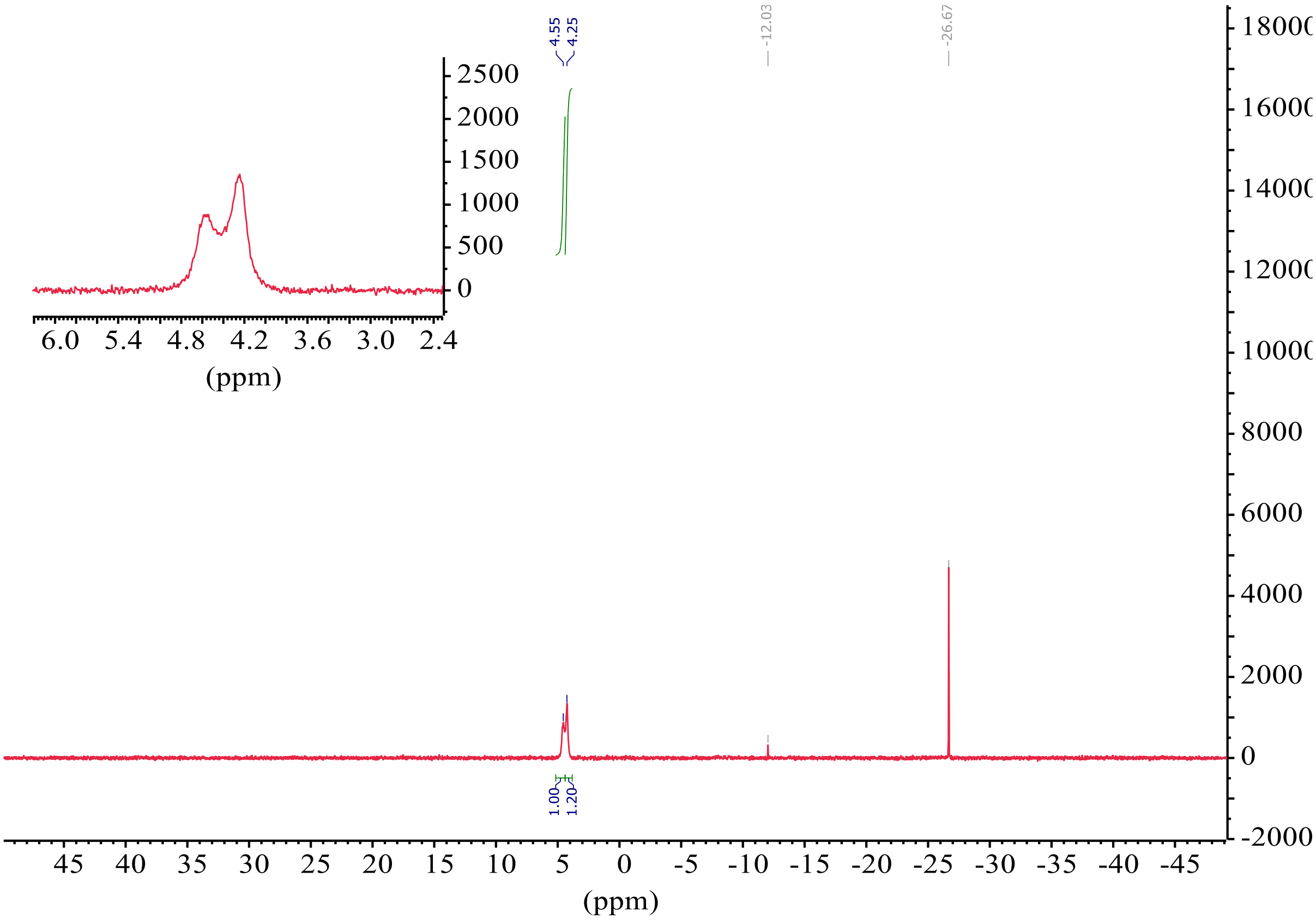

Figure S89: $^{31}P\{^{1}H\}$ NMR (202 MHz) spectrum of **3h-PMes₃** in CD_2Cl_2. Residual impurities marked in grey.

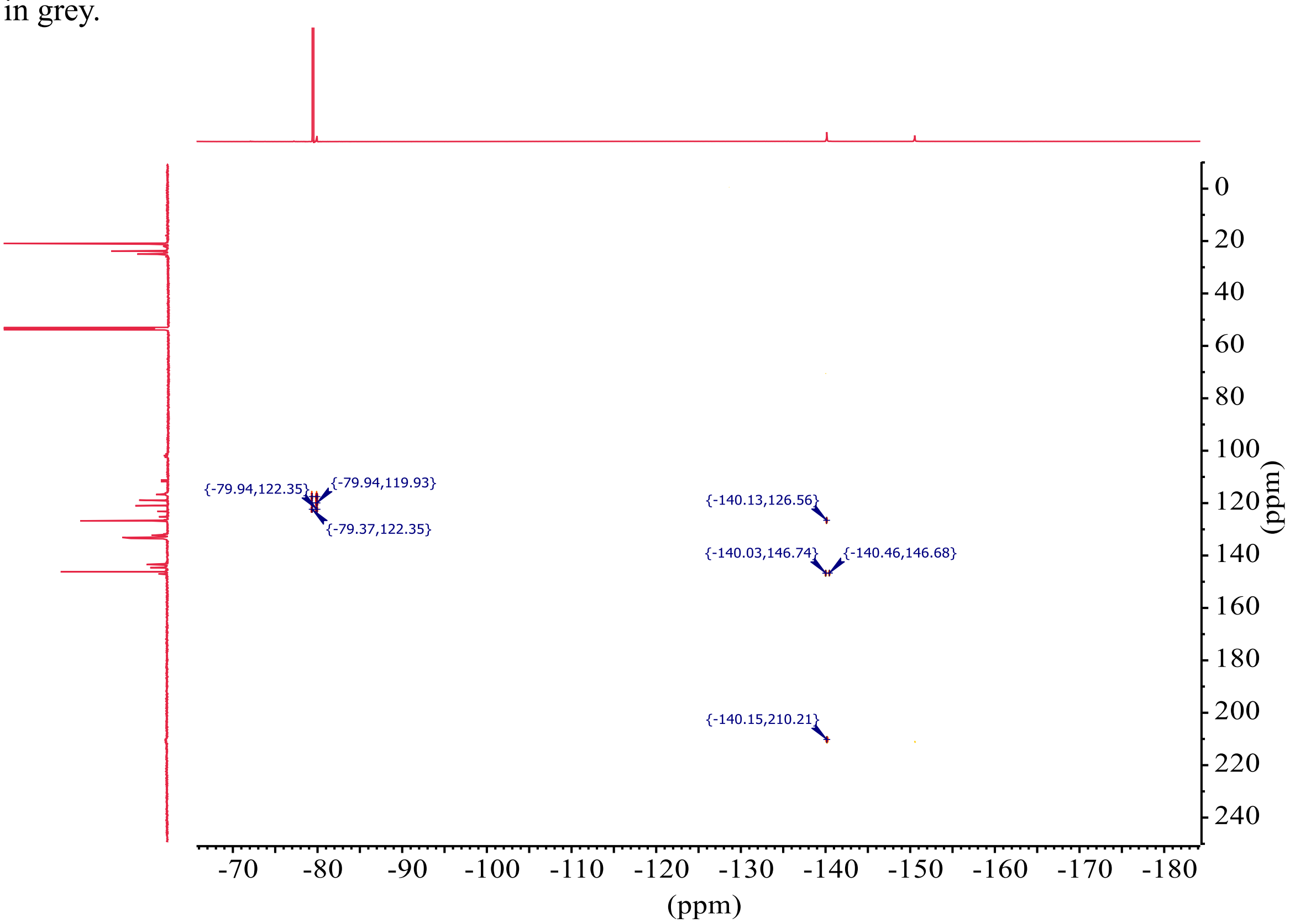

Figure S90: ^{19}F-^{13}C HMBC NMR (565 MHz-150 MHz) spectrum of **3h-PMes₃** in CD_2Cl_2.

3i-PTol3

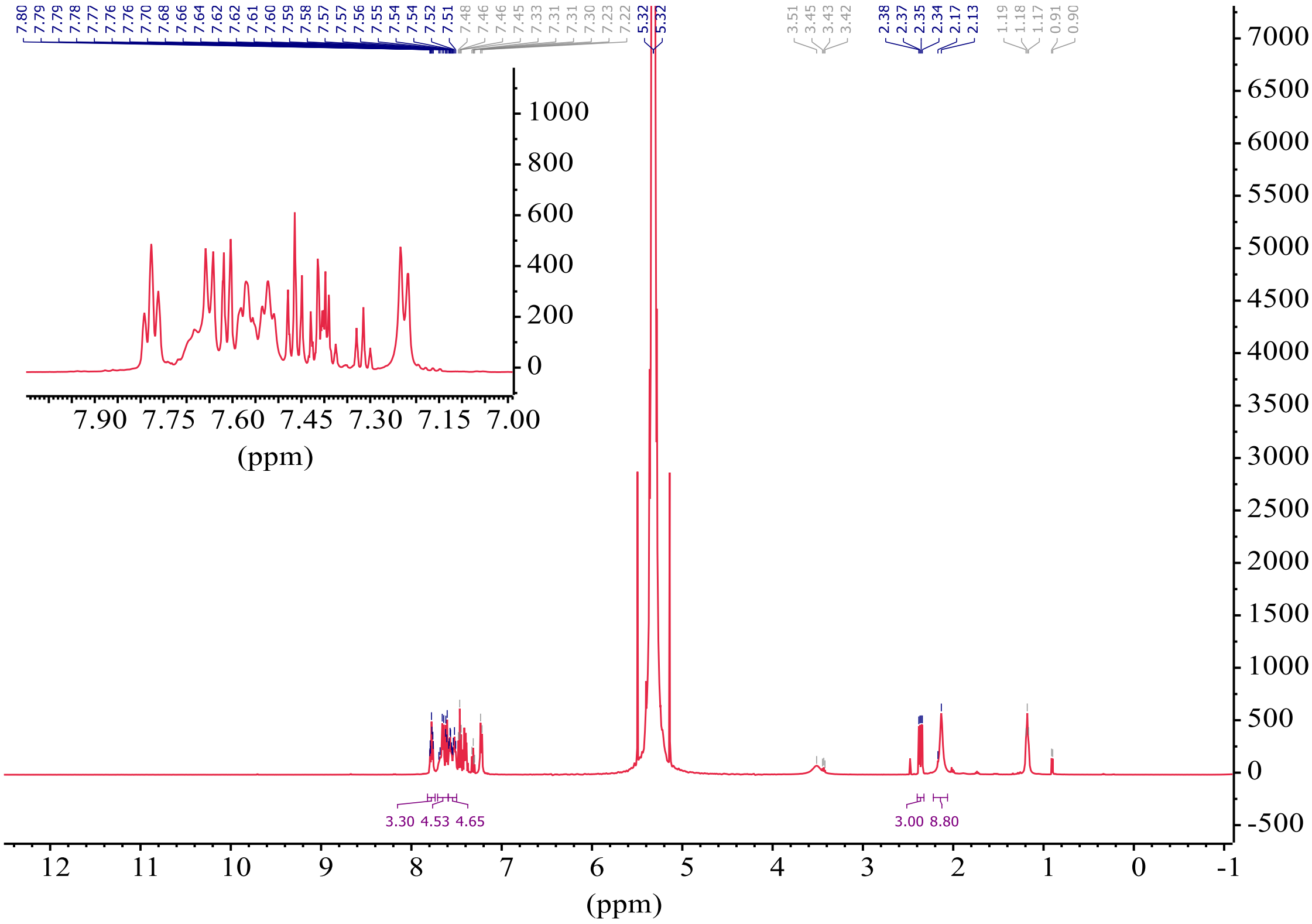

Figure S91: ^{1}H NMR (500 MHz) spectrum of **3i-PTol3** in CH_2Cl_2. Residual solvent impurities are marked with grey ticks.

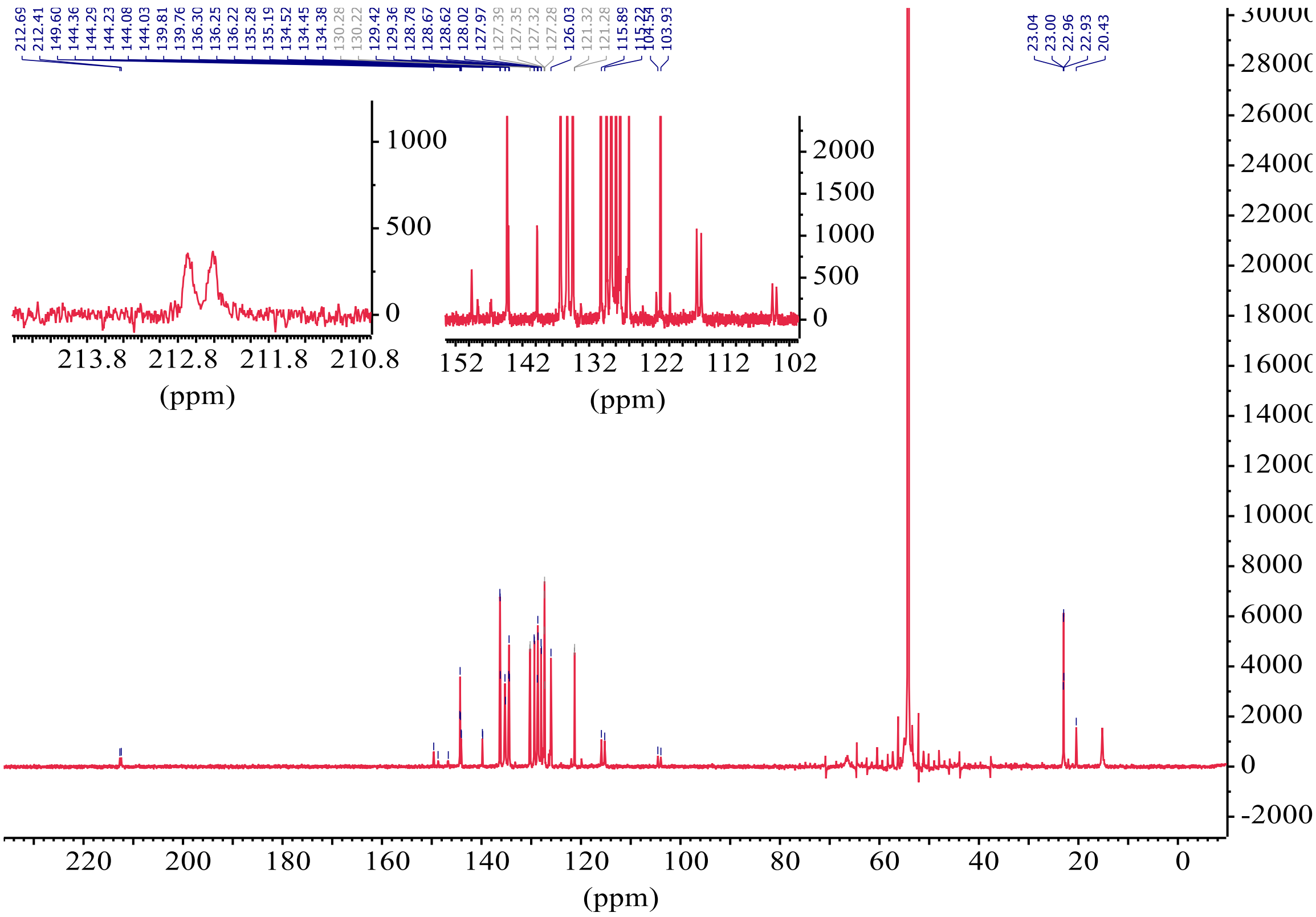

Figure S92: $^{13}C\{^1H\}$ NMR (125 MHz) spectrum of **3i-PTol3** in CH_2Cl_2.

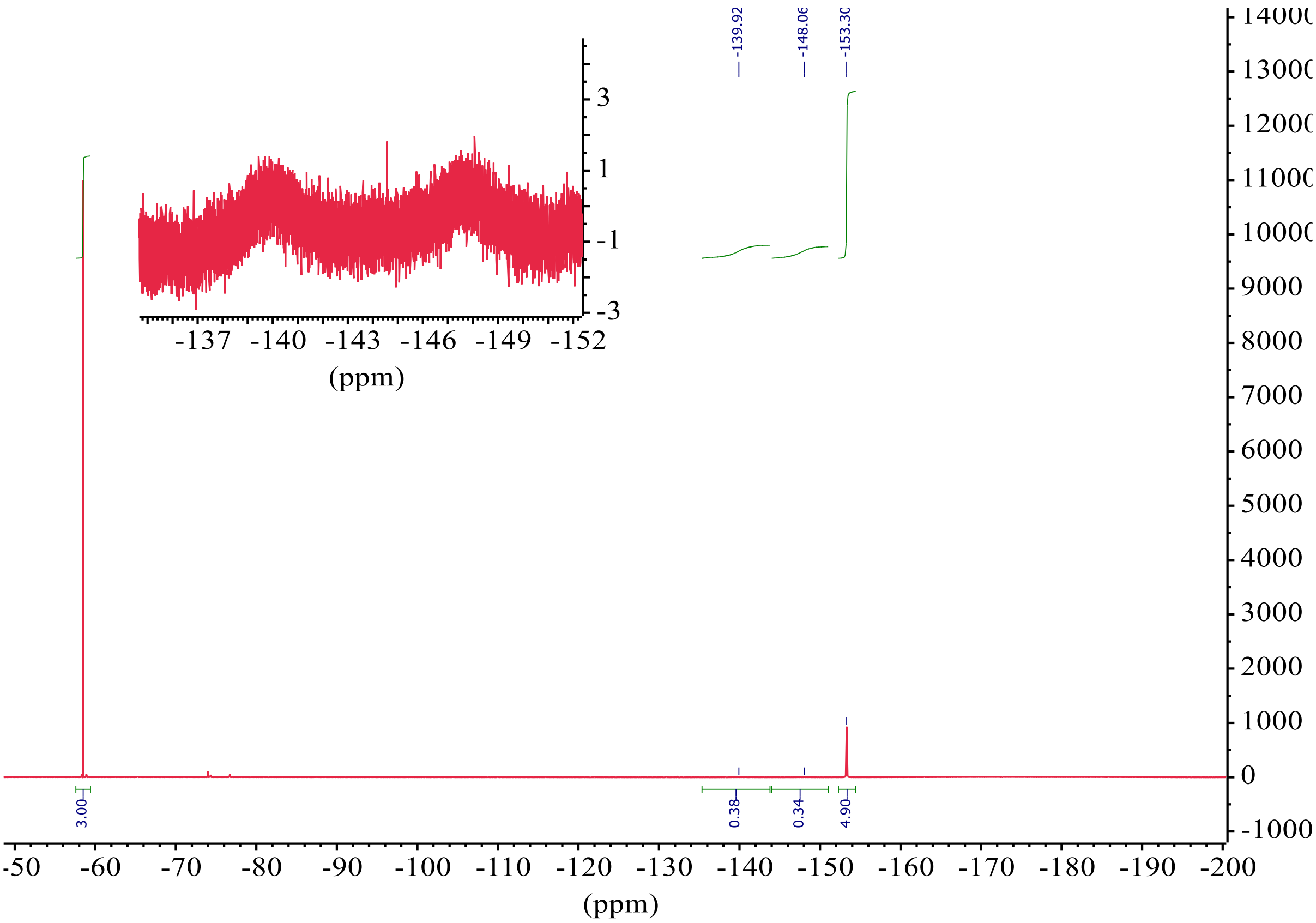

Figure S93: $^{19}F\{^{1}H\}$ NMR (470 MHz) spectrum of **3i-PTol₃** in CH_2Cl_2.

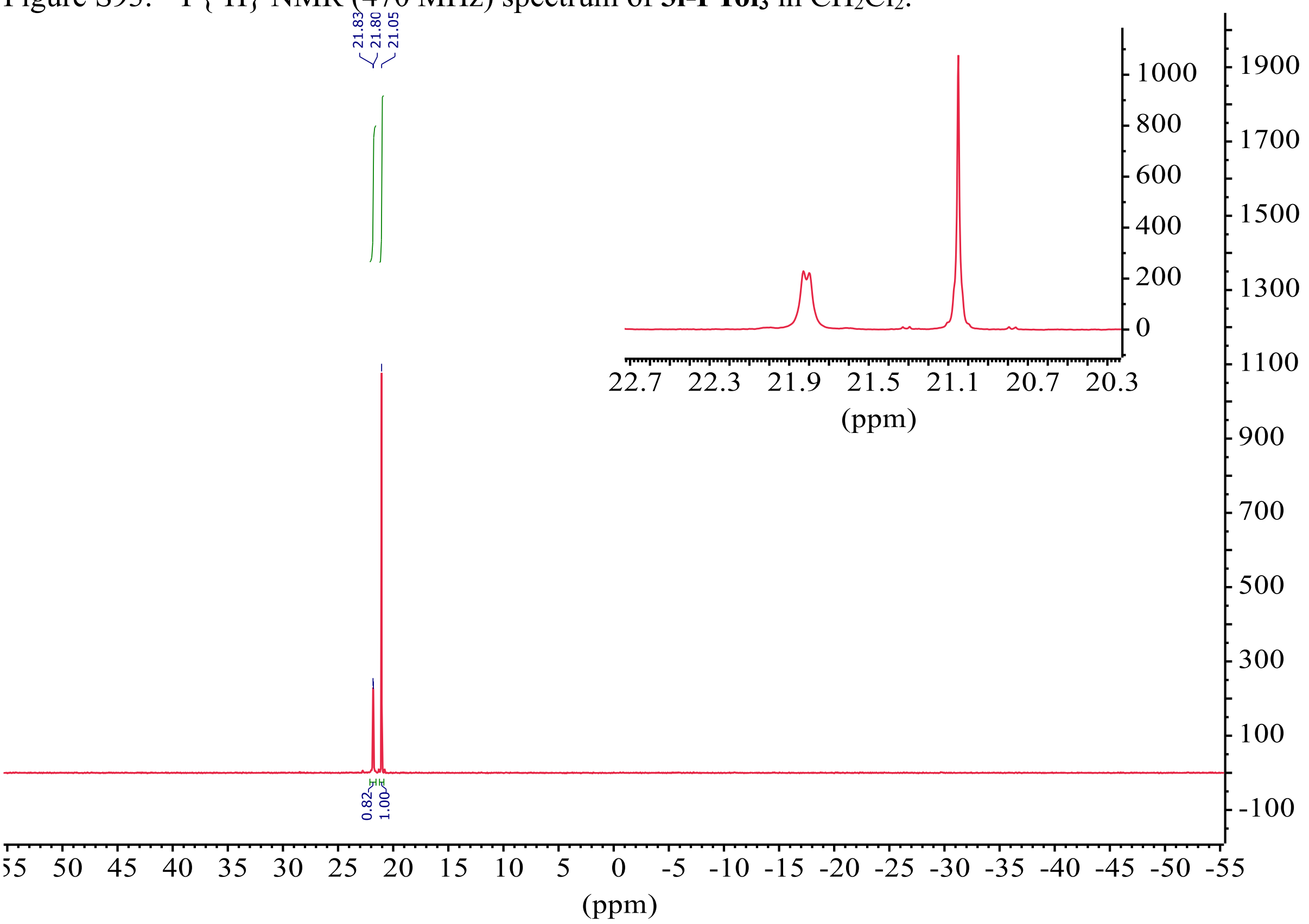

Figure S94: $^{31}P\{^{1}H\}$ NMR (202 MHz) spectrum of **3i-PTol₃** in CH_2Cl_2.

2l-PTol

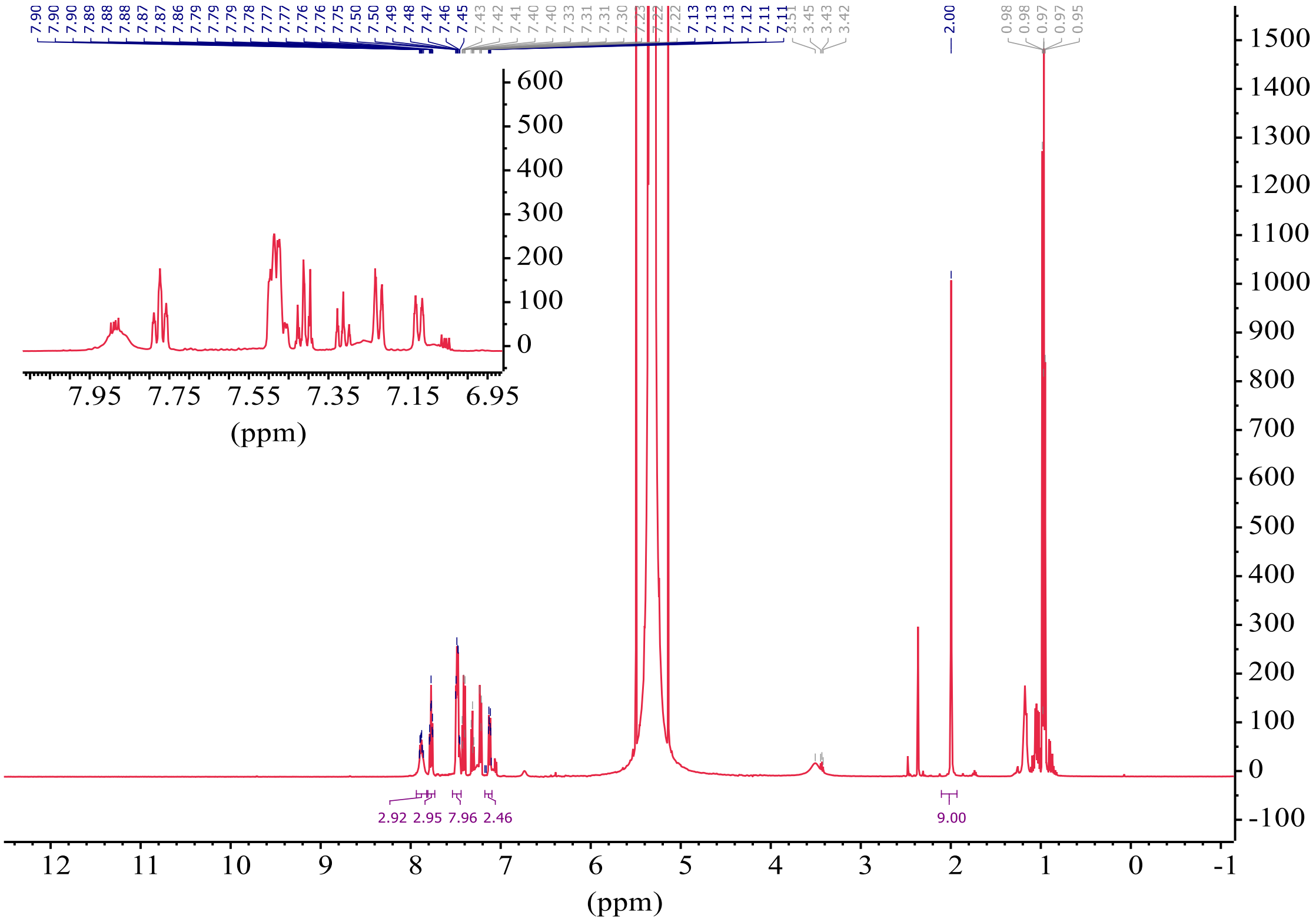

Figure S95: ^{1}H NMR (500 MHz) spectrum of **2l-PTol$_3$** in CH_2Cl_2. Residual solvent impurities are marked with grey ticks.

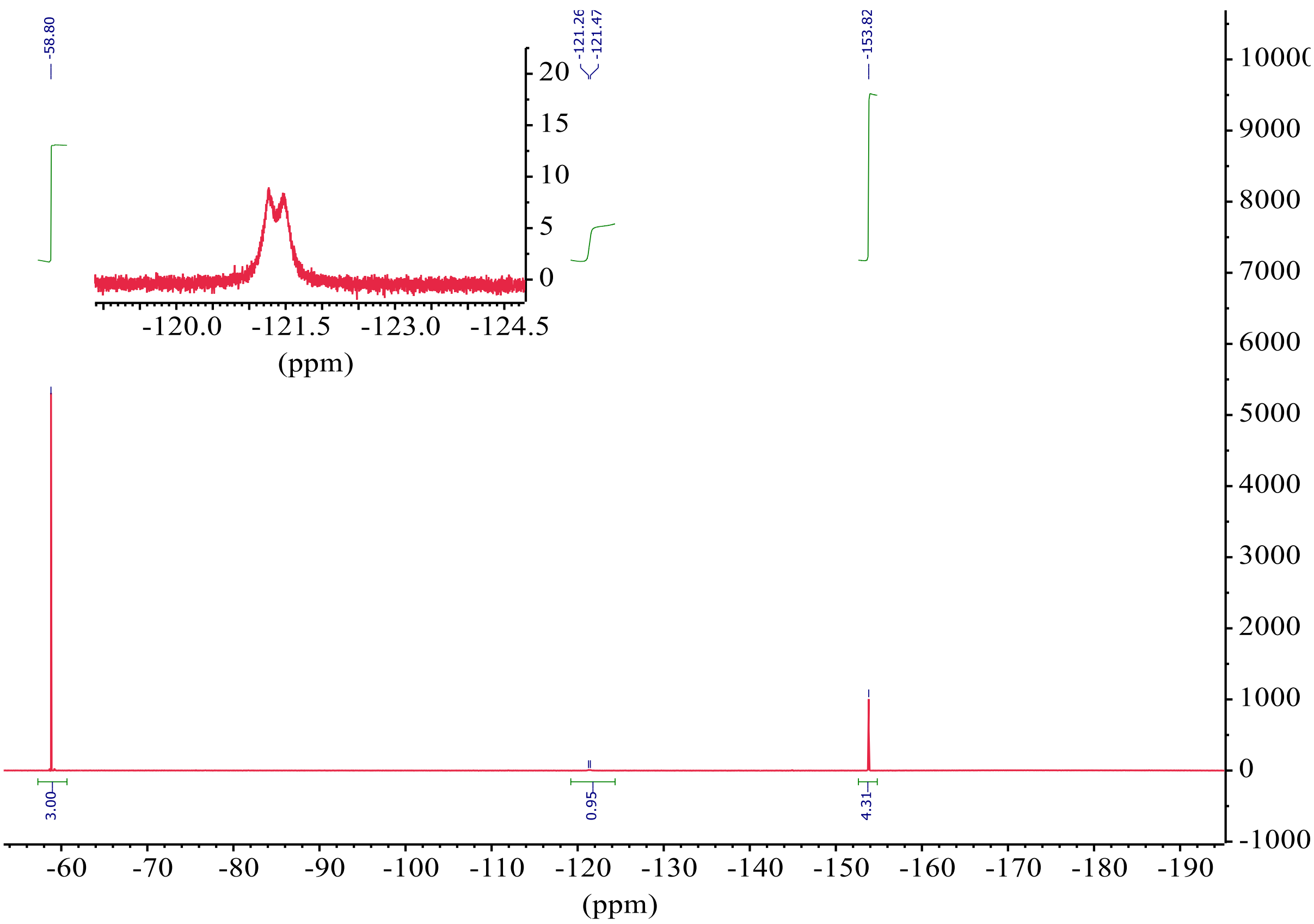

Figure S96: ^{19}F{^{1}H} NMR (470 MHz) spectrum of **2l-PTol$_3$** in CH_2Cl_2.

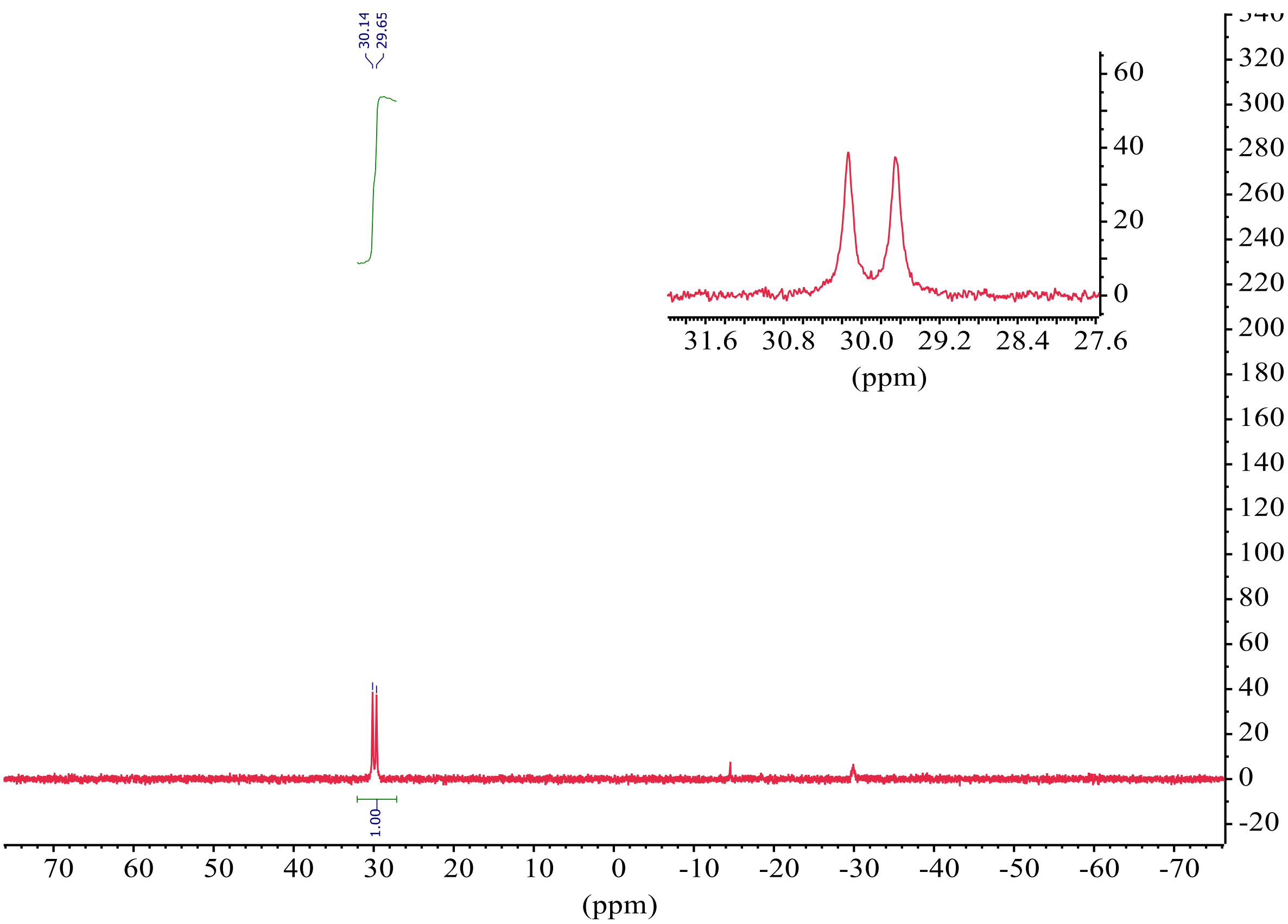

Figure S97: $^{31}P\{^{1}H\}$ NMR (202 MHz) spectrum of **2l-PTol₃** in CH_2Cl_3.

2m-PTol₃

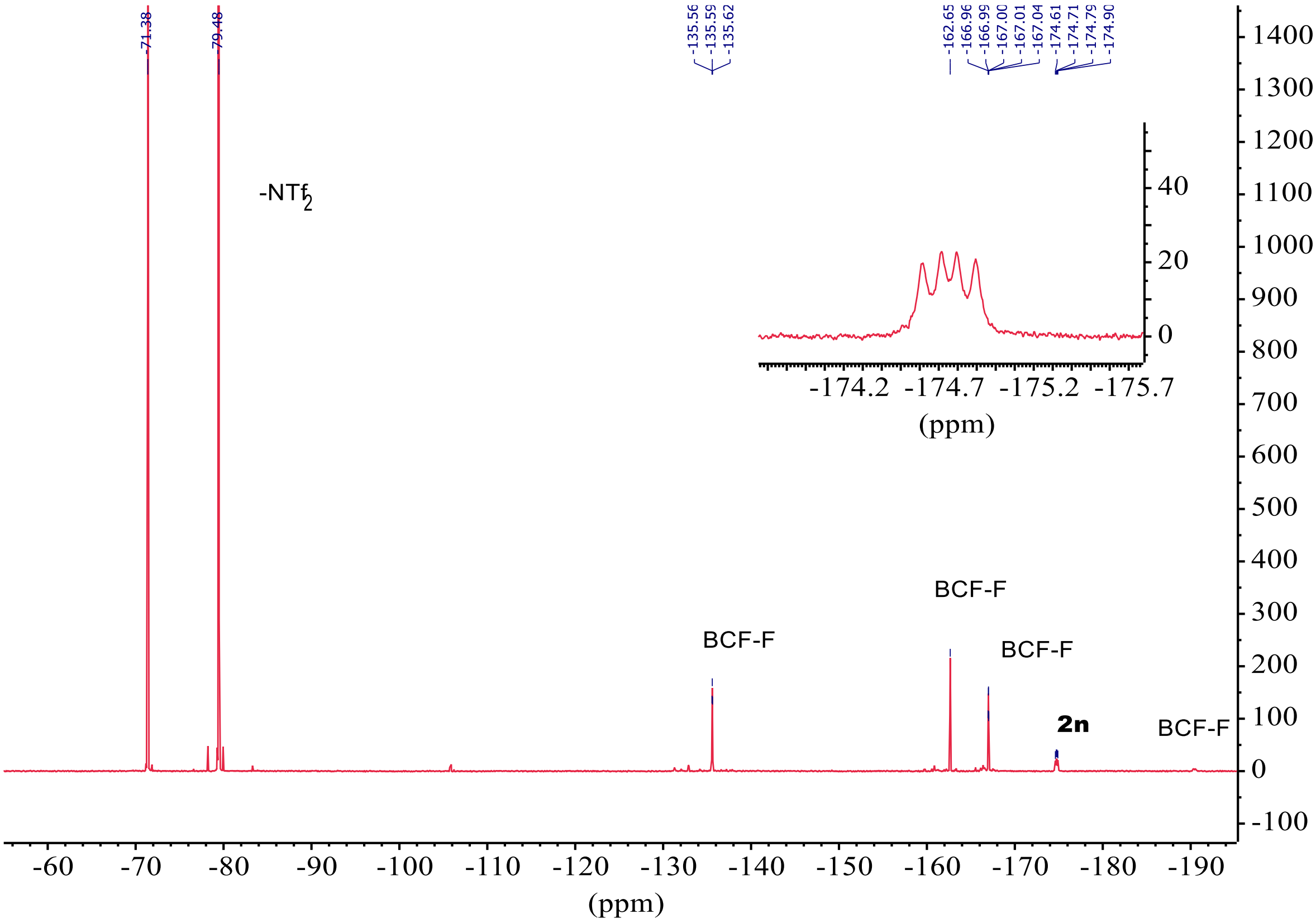

Figure S98: ^{19}F NMR (470 MHz) spectrum of **2m-PTol₃** in CH_2Cl_2.

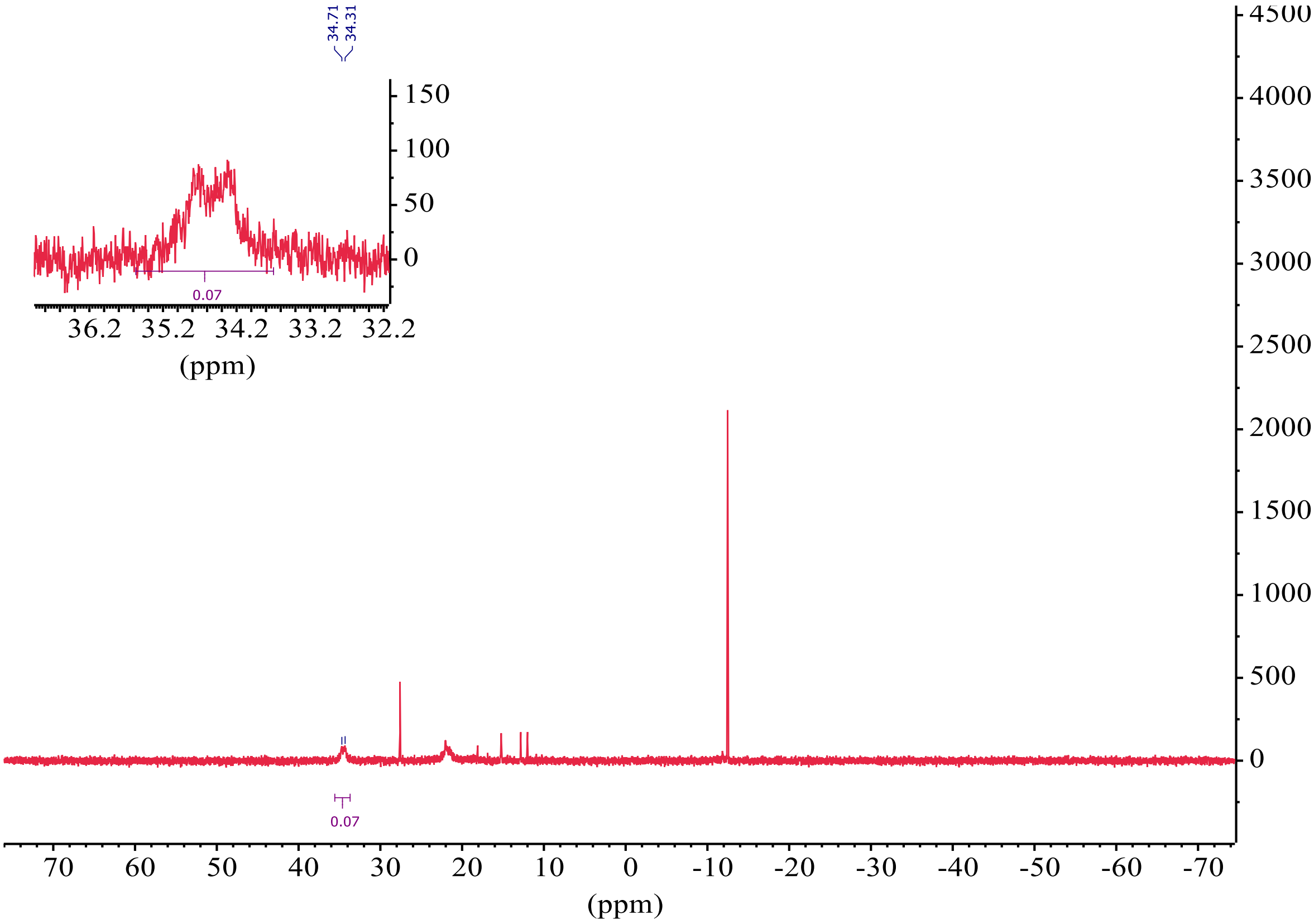

Figure S99: $^{31}P\{^1H\}$ NMR (202 MHz) spectrum of **2m-PTol₃** in CH_2Cl_2.

3a-TPPy

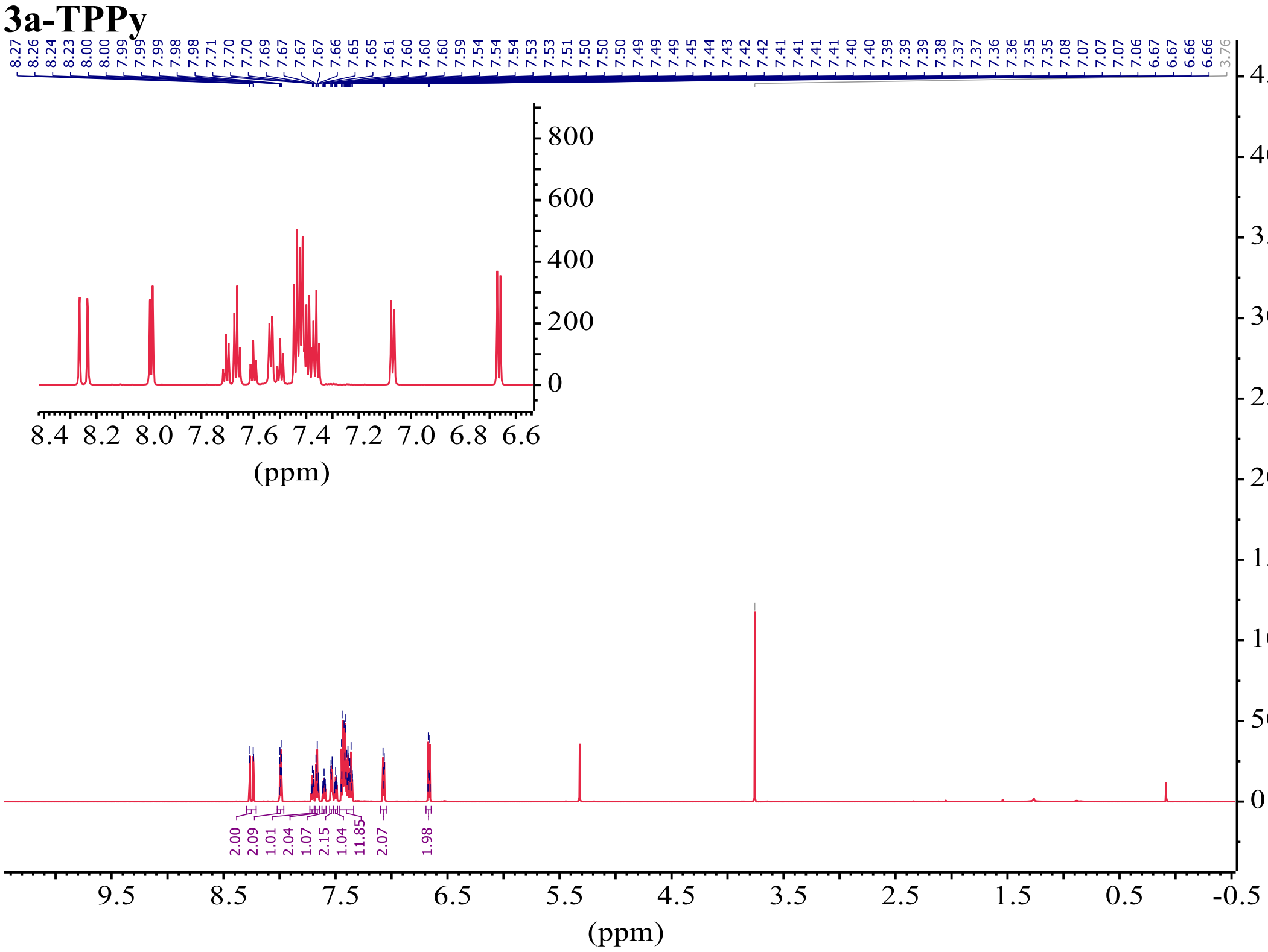

Figure S100: ^{1}H NMR (700 MHz) spectrum of **3a-TPPy** in CD_2Cl_2. Residual solvent impurities are marked with grey ticks.

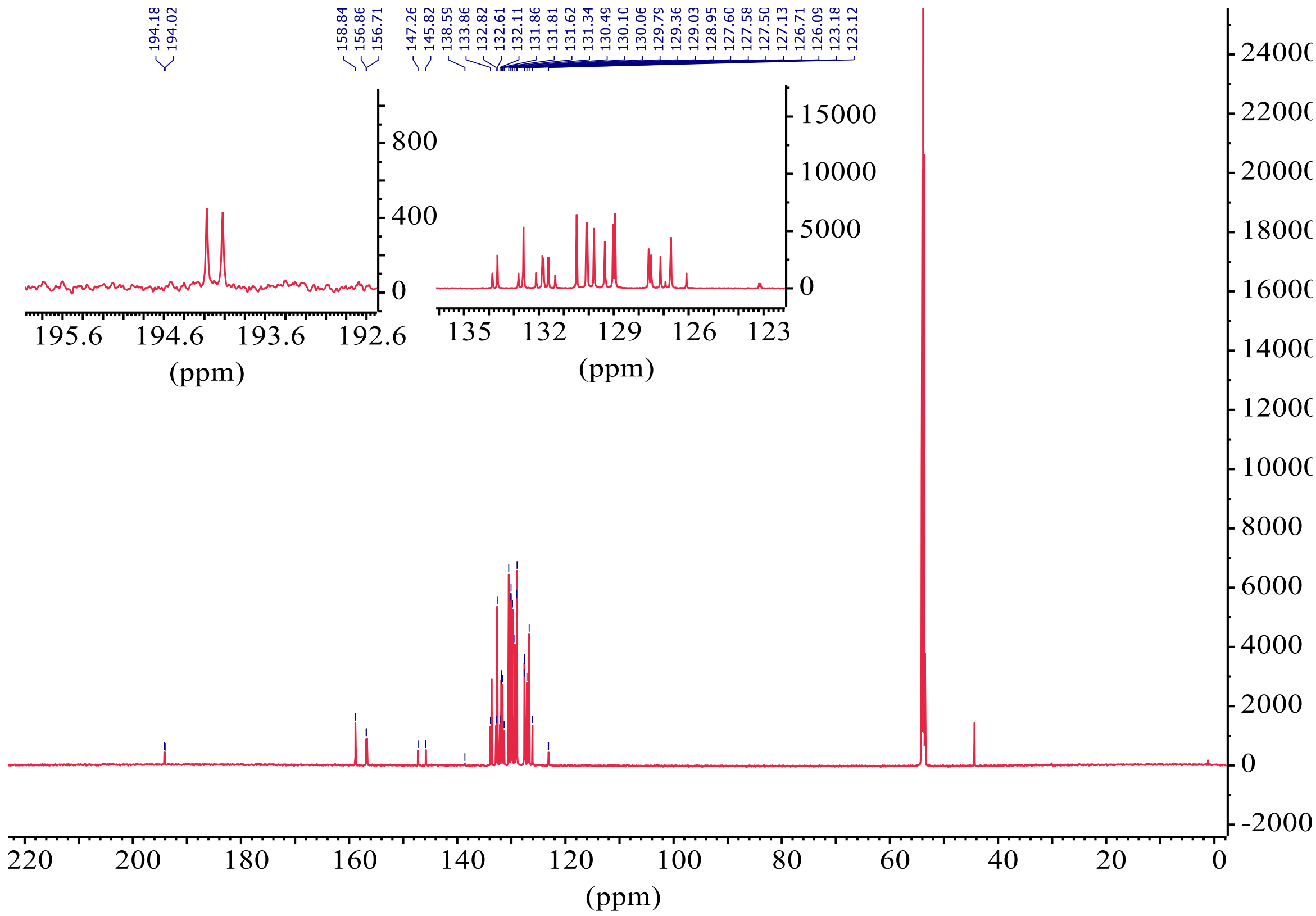

Figure S101: $^{13}C\{^{1}H\}$ NMR (175 MHz) spectrum of **3a-TPPy** in CD_2Cl_2.

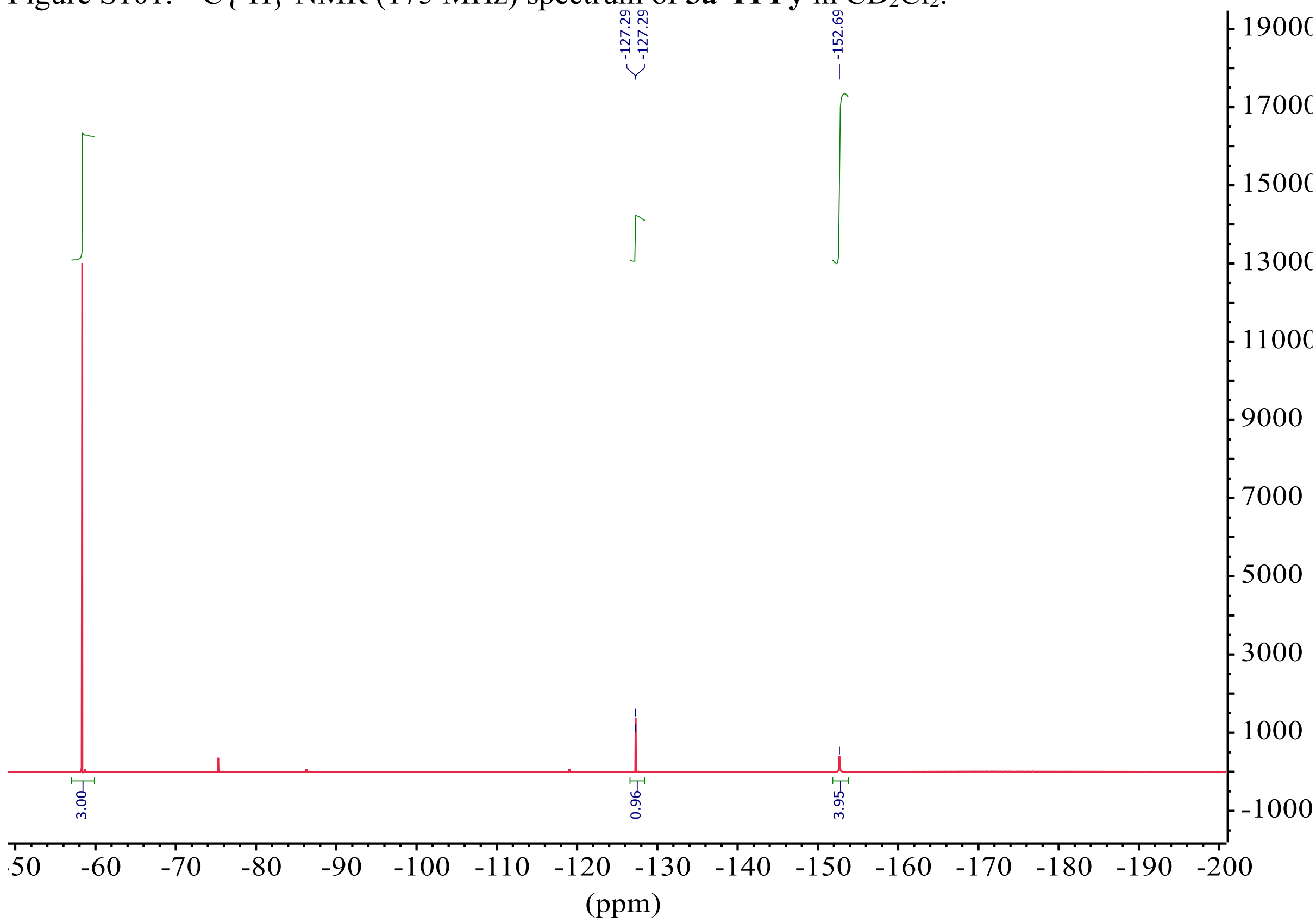

Figure S102: $^{19}F\{^{1}H\}$ NMR (659 MHz) spectrum of **3a-TPPy** in CD_2Cl_2.

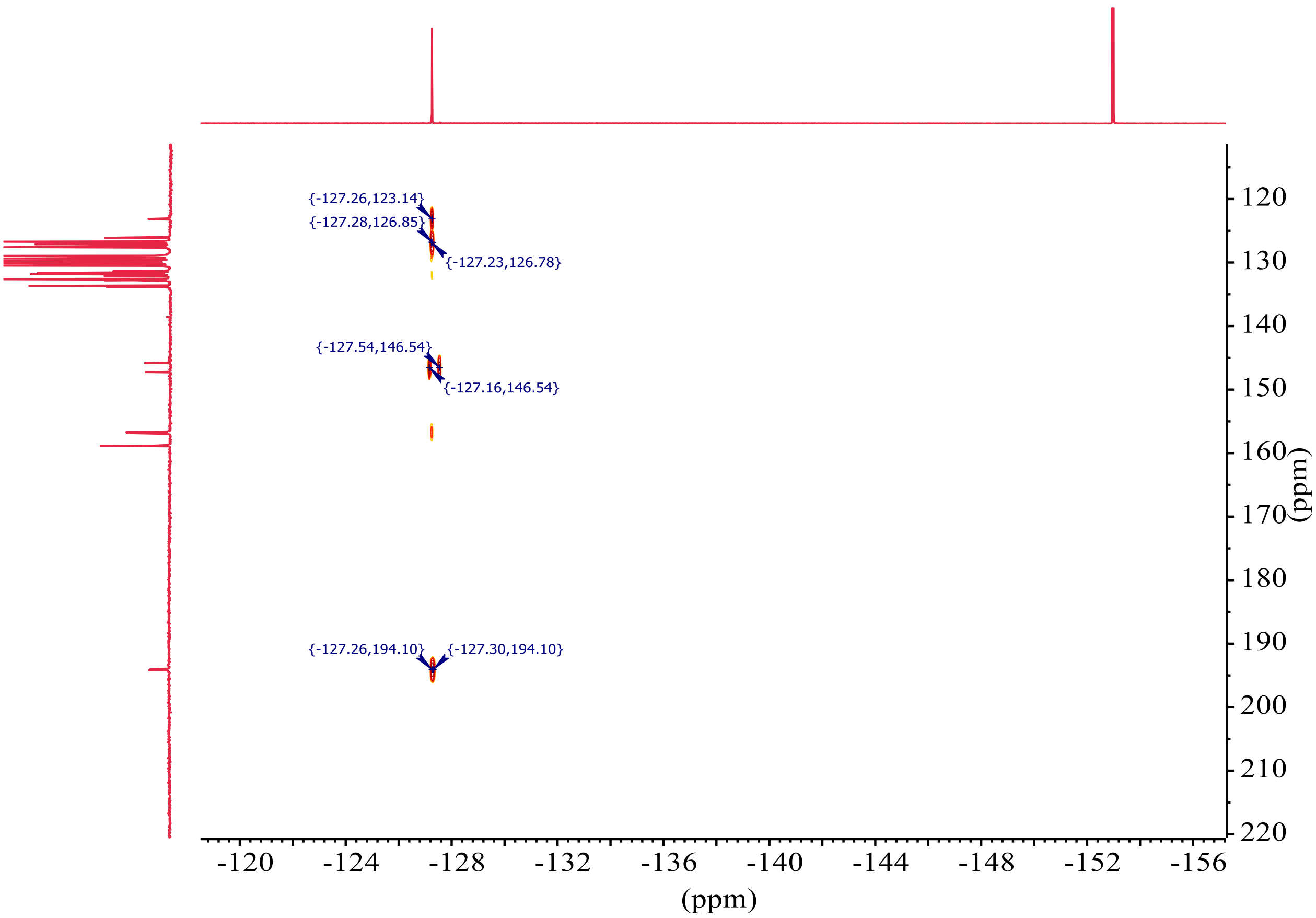

Figure S103: ^{19}F-^{13}C HMBC NMR (659 MHz-175 MHz) spectrum of **3a-TPPy** in CD_2Cl_2.

3c-TPPy

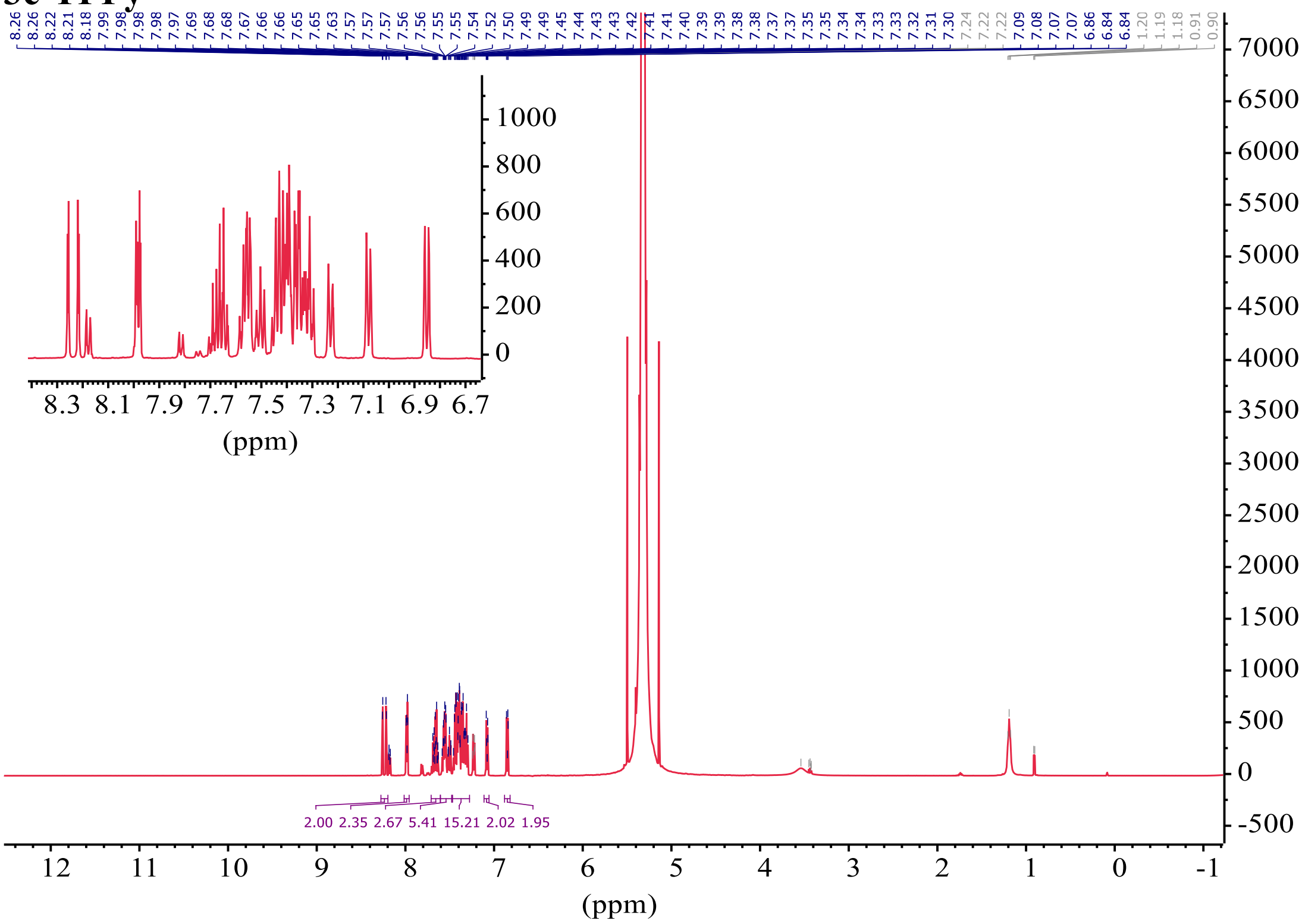

Figure S104: ^{1}H NMR (500 MHz) spectrum of **3c-TPPy** in CH_2Cl_2. Residual solvent impurities are marked with grey ticks.

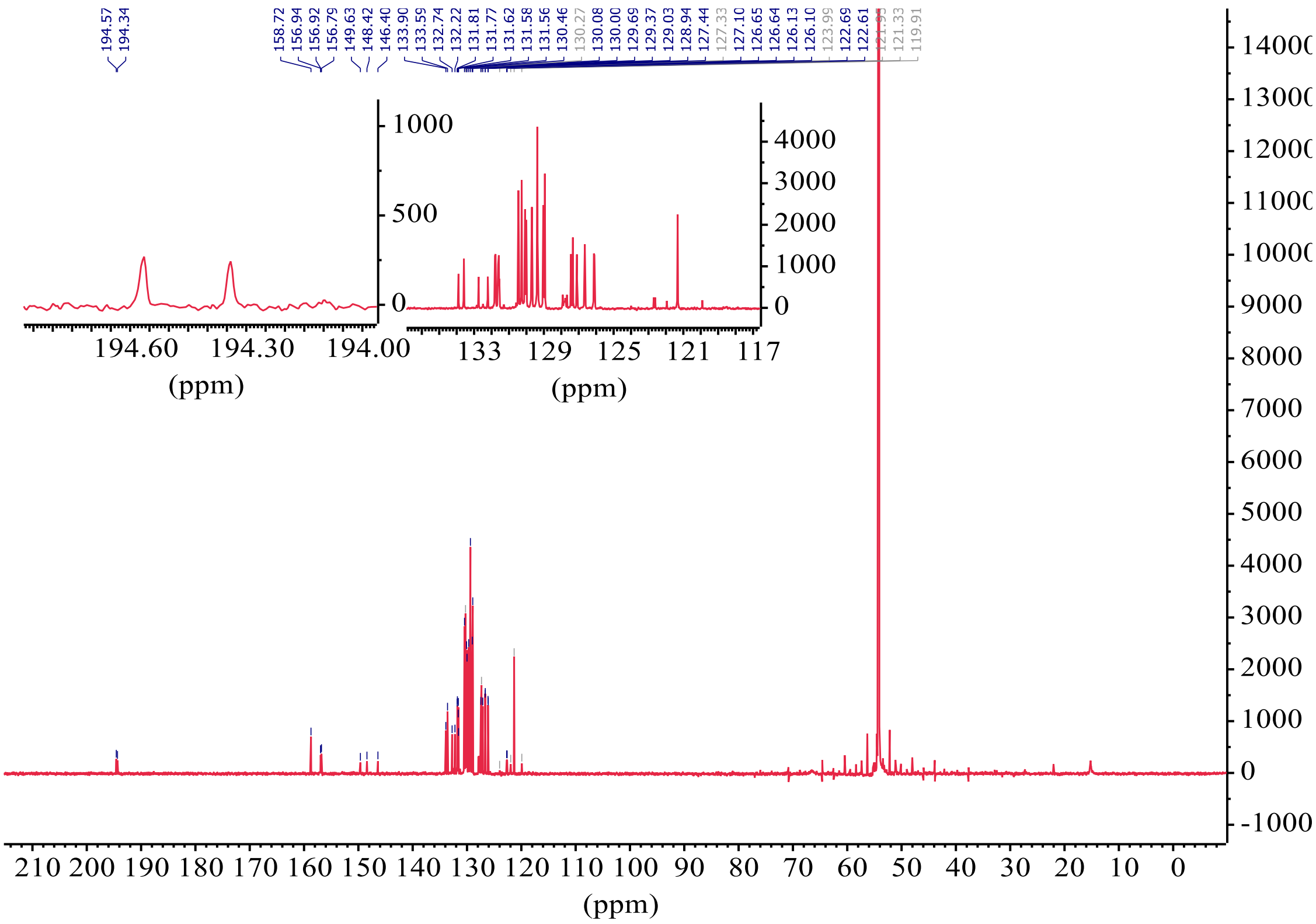

Figure S105: $^{13}C\{^{1}H\}$ NMR (125 MHz) spectrum of **3c-TPPy** in CH_2Cl_2.

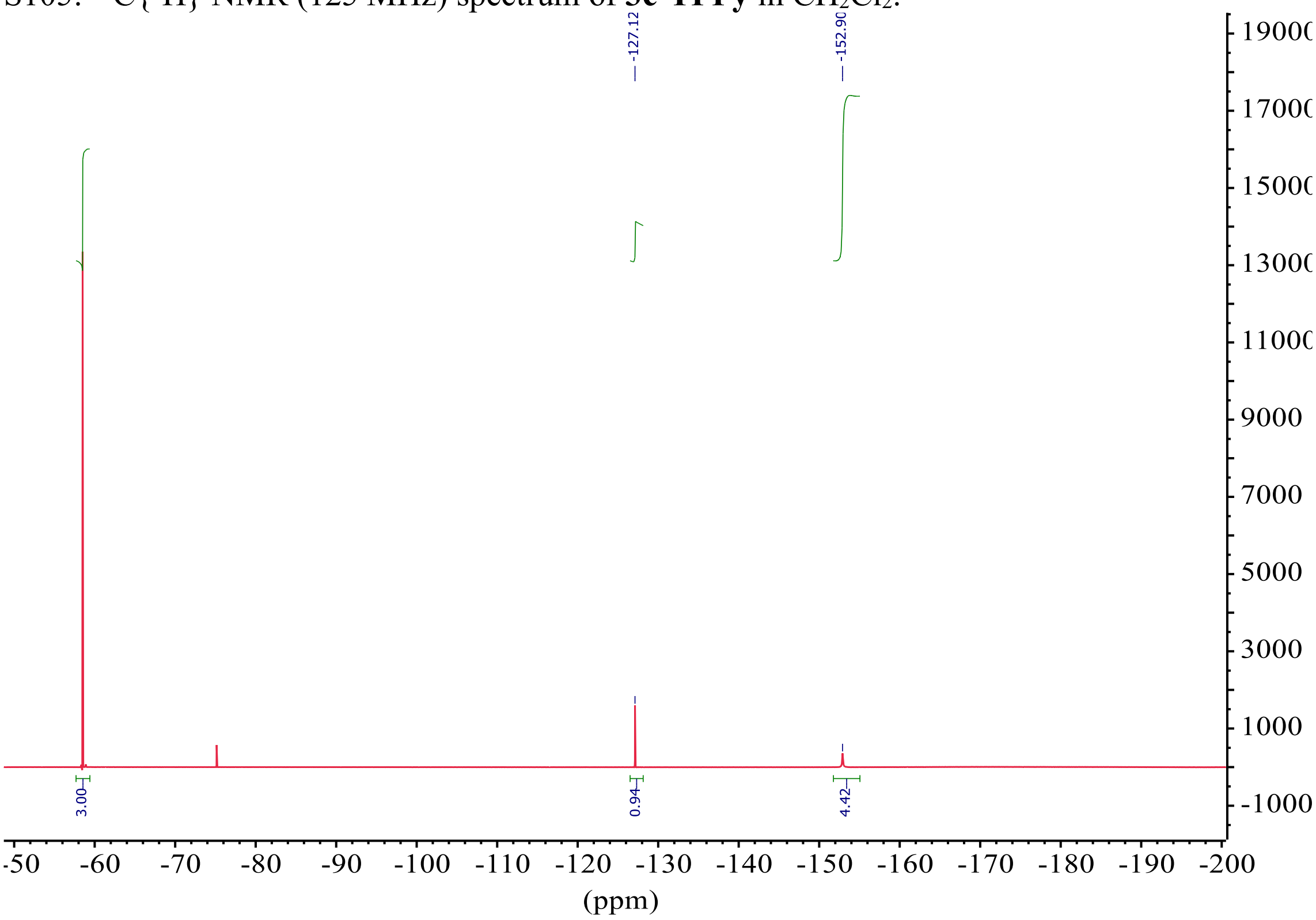

Figure S106: $^{19}F\{^{1}H\}$ NMR (470 MHz) spectrum of **3c-TPPy** in CH_2Cl_2.

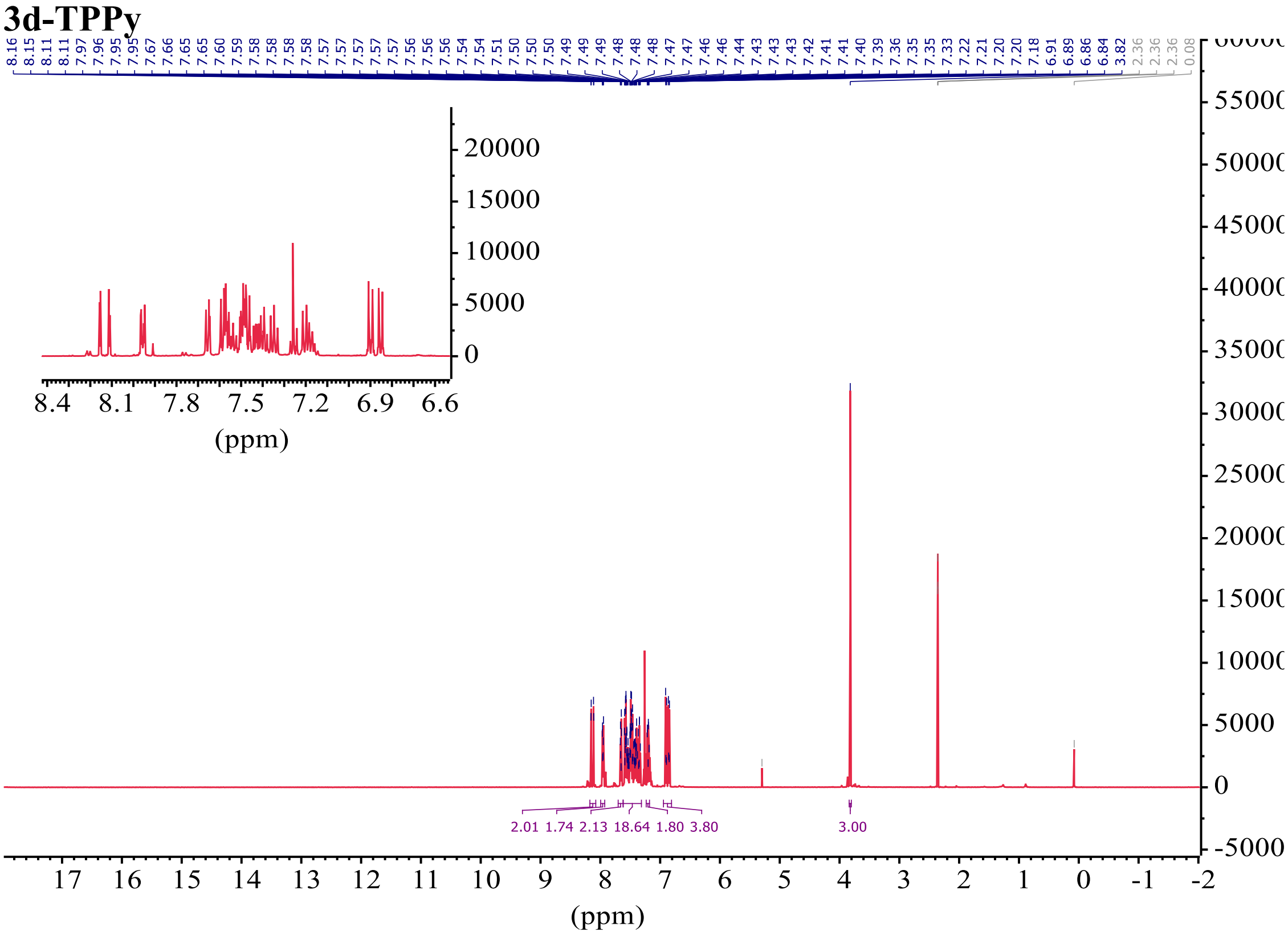

Figure S107: ^{1}H NMR (500 MHz) spectrum of **3d-TPPy** in $CDCl_3$. Residual solvent impurities are marked with grey ticks.

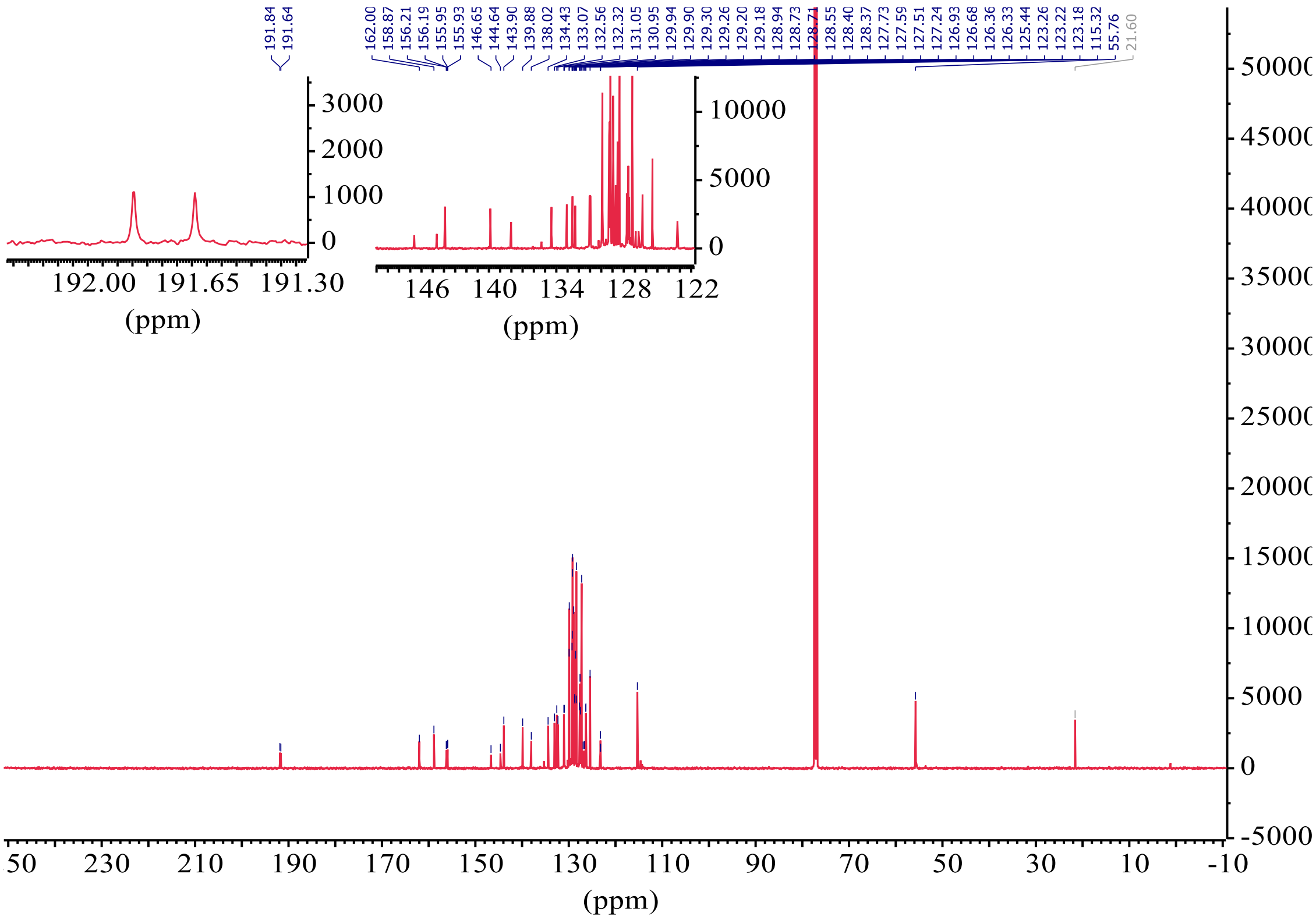

Figure S108: $^{13}C\{^{1}H\}$ NMR (125 MHz) spectrum of **3d-TPPy** in $CDCl_3$.

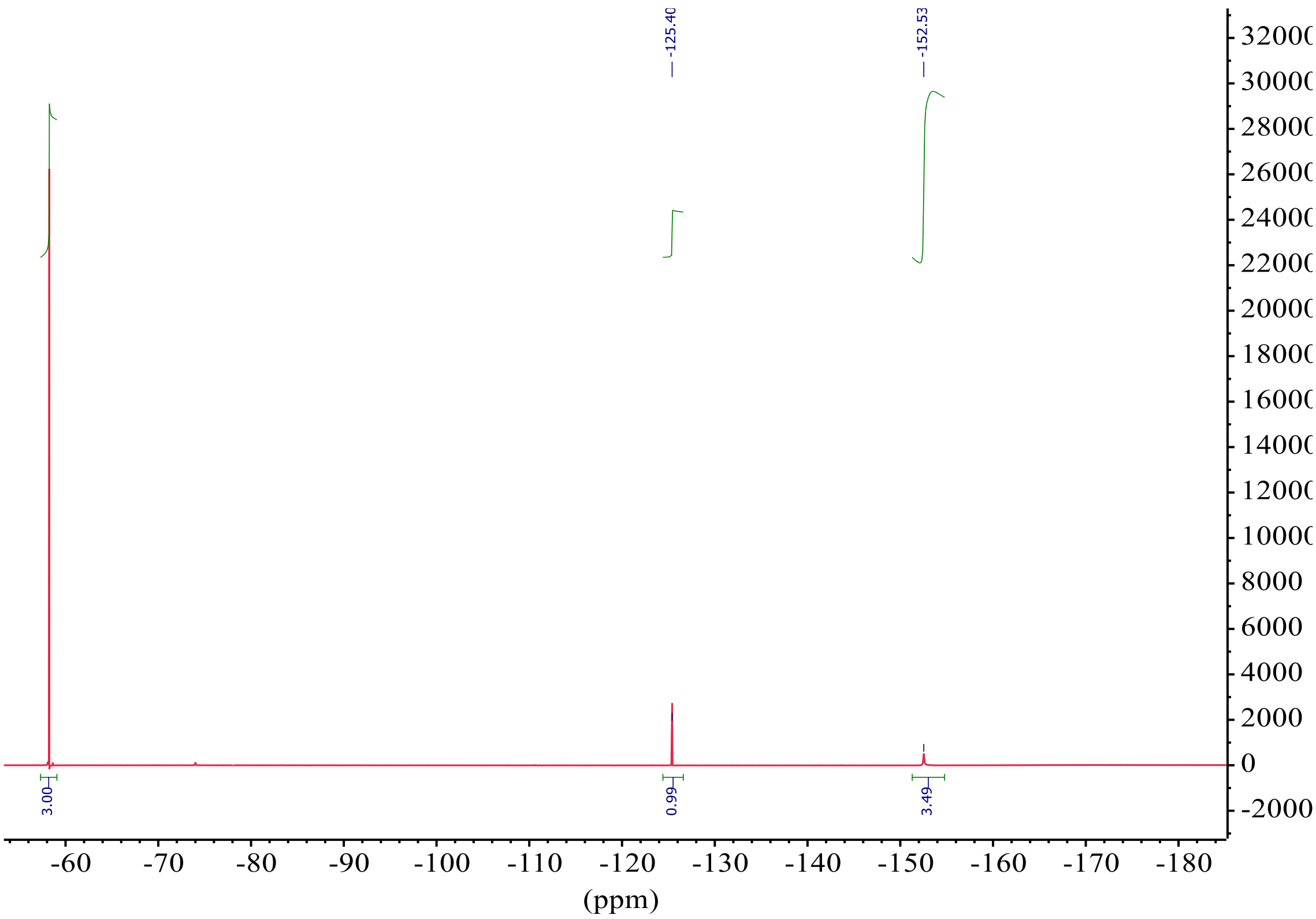

Figure S109: $^{19}F\{^{1}H\}$ NMR (470 MHz) spectrum of **3d-TPPy** in $CDCl_3$.

3f-TPPy

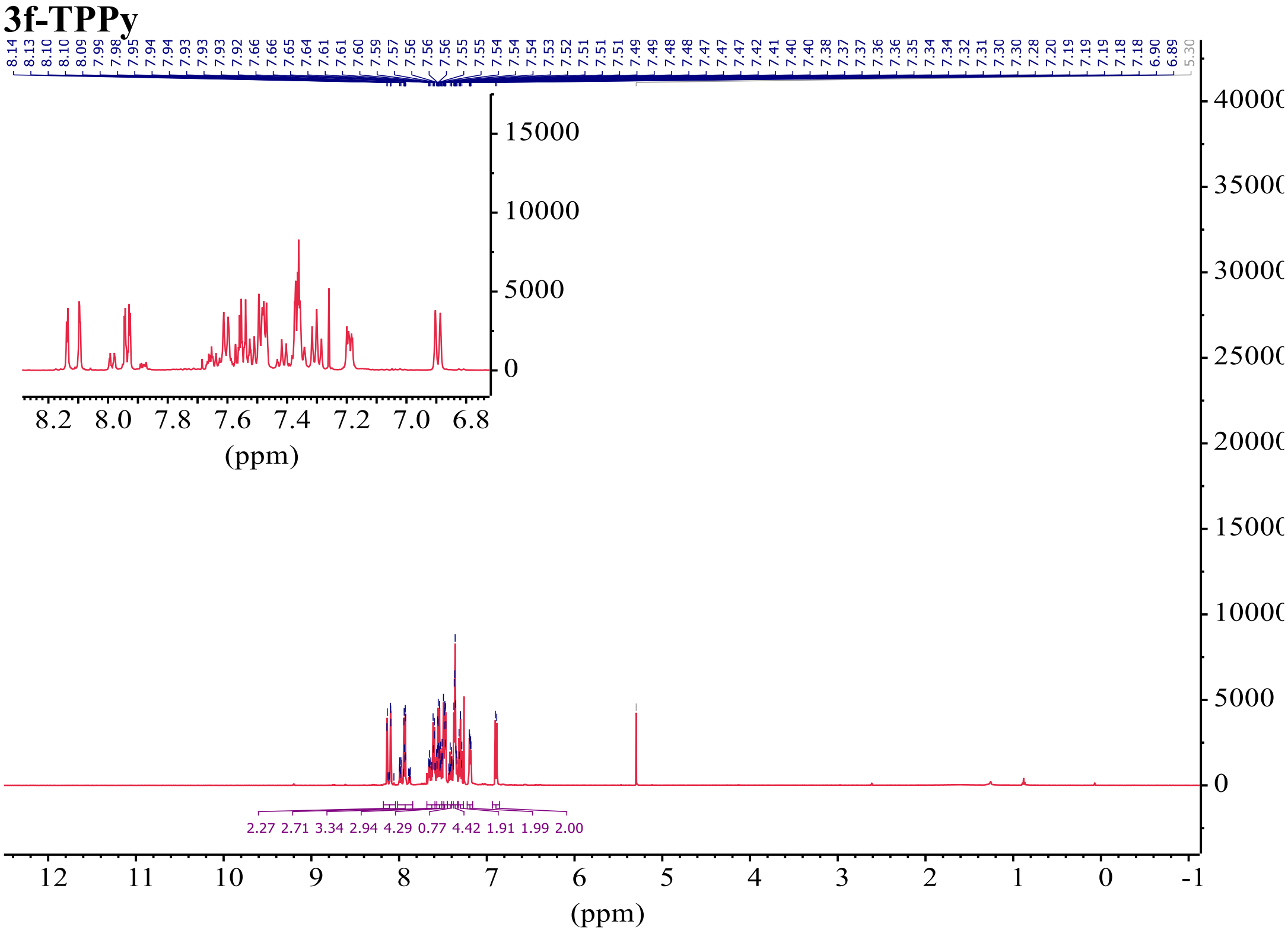

Figure S110: ^{1}H NMR (500 MHz) spectrum of **3f-TPPy** in $CDCl_3$. Residual solvent impurities are marked with grey ticks.

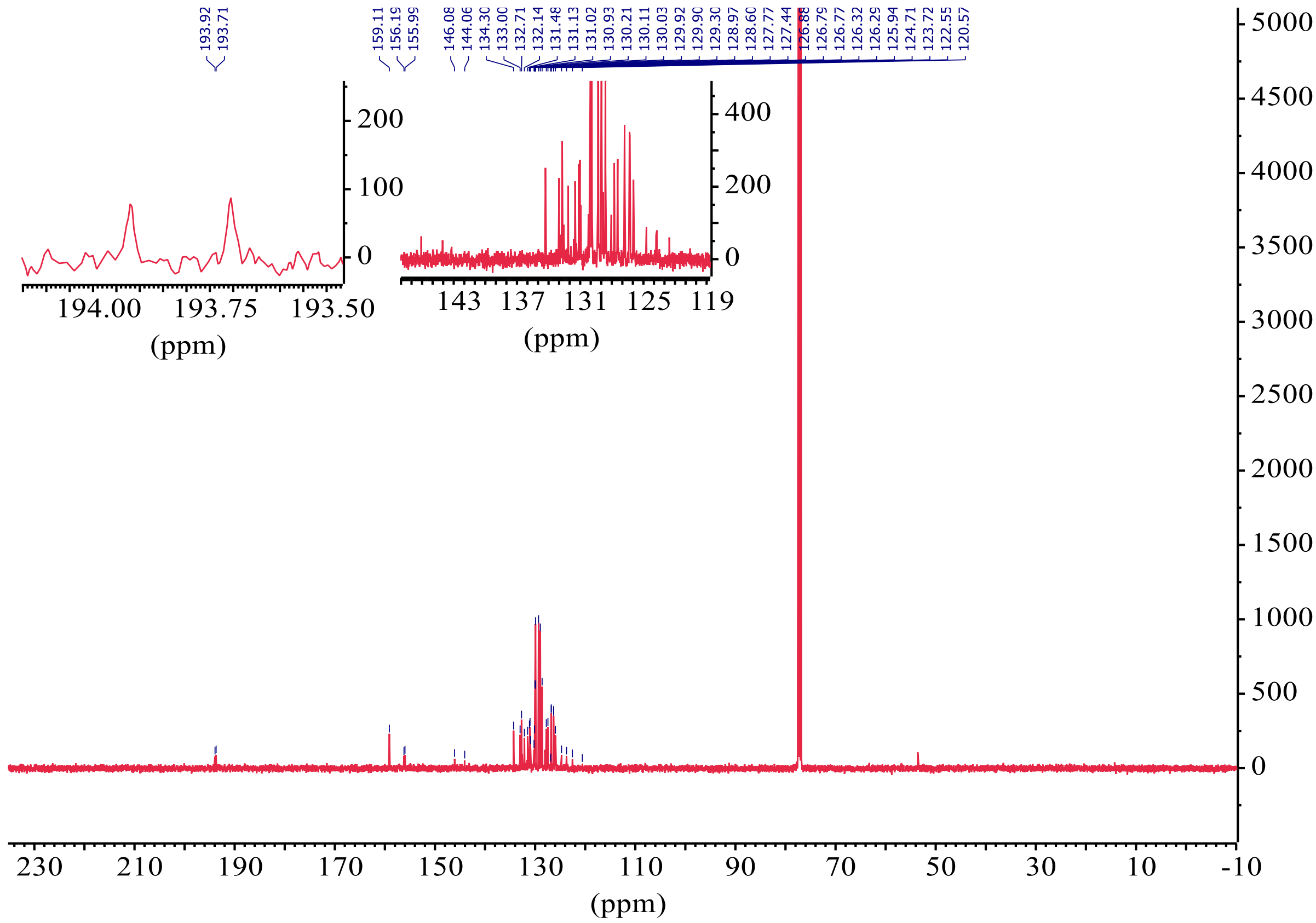

Figure S111: $^{13}C\{^{1}H\}$ NMR (125 MHz) spectrum of **3f-TPPy** in $CDCl_3$.

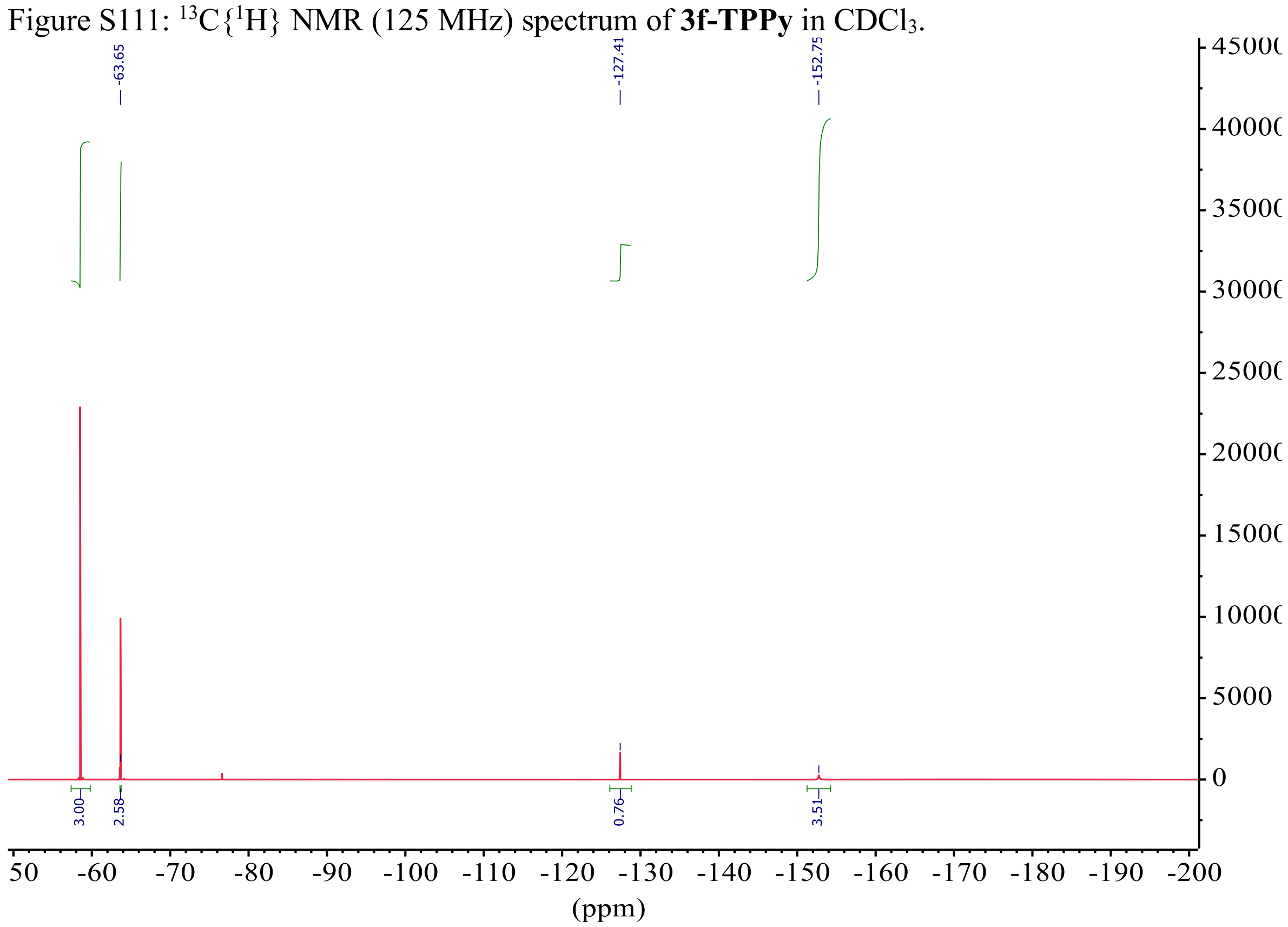

Figure S112: $^{19}F\{^{1}H\}$ NMR (470 MHz) spectrum of **3f-TPPy** in $CDCl_3$.

3j-TPPy

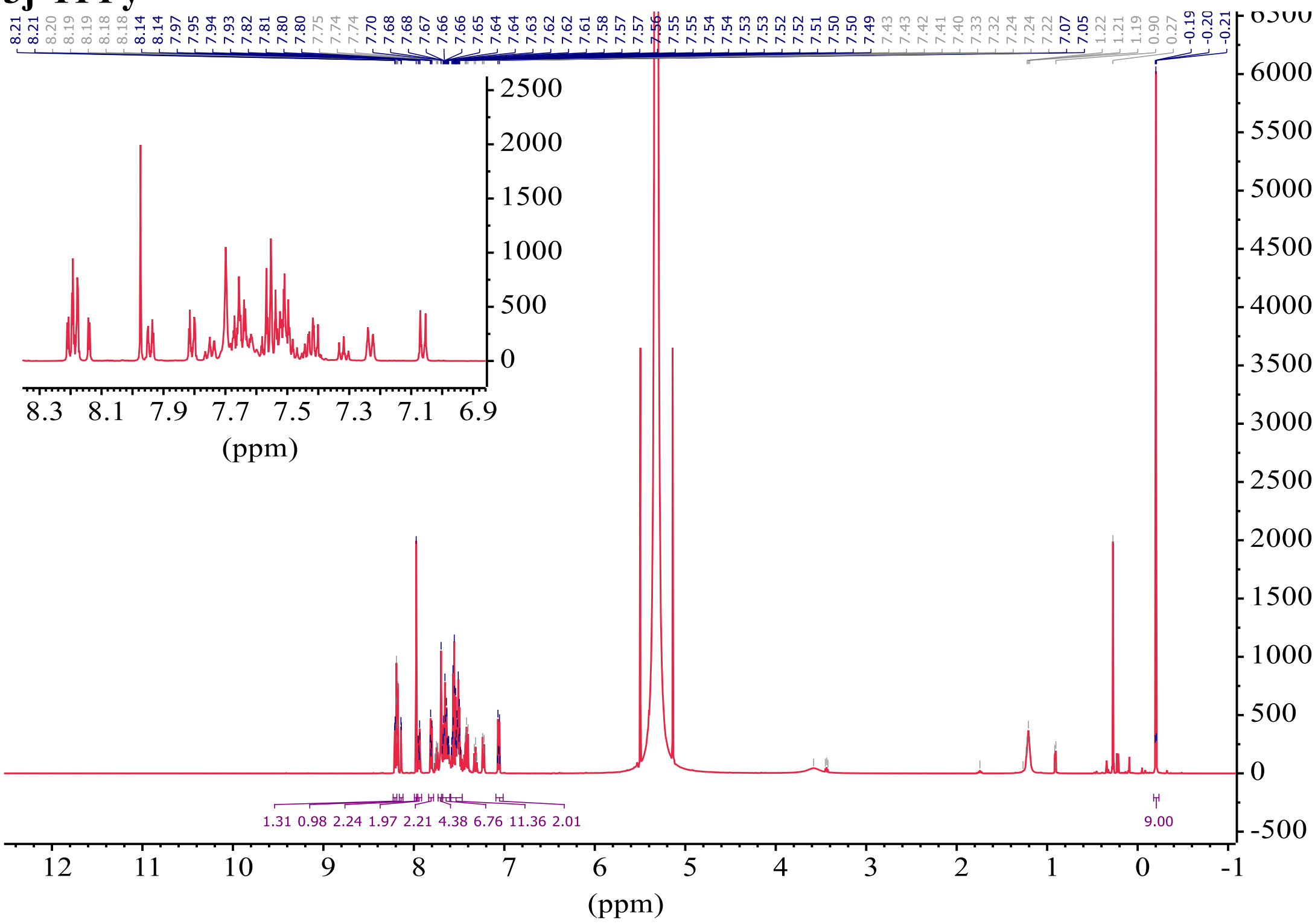

Figure S113: ^1H NMR (500 MHz) spectrum of **3j-TPPy** in CH_2Cl_2. Residual solvent impurities are marked with grey ticks.

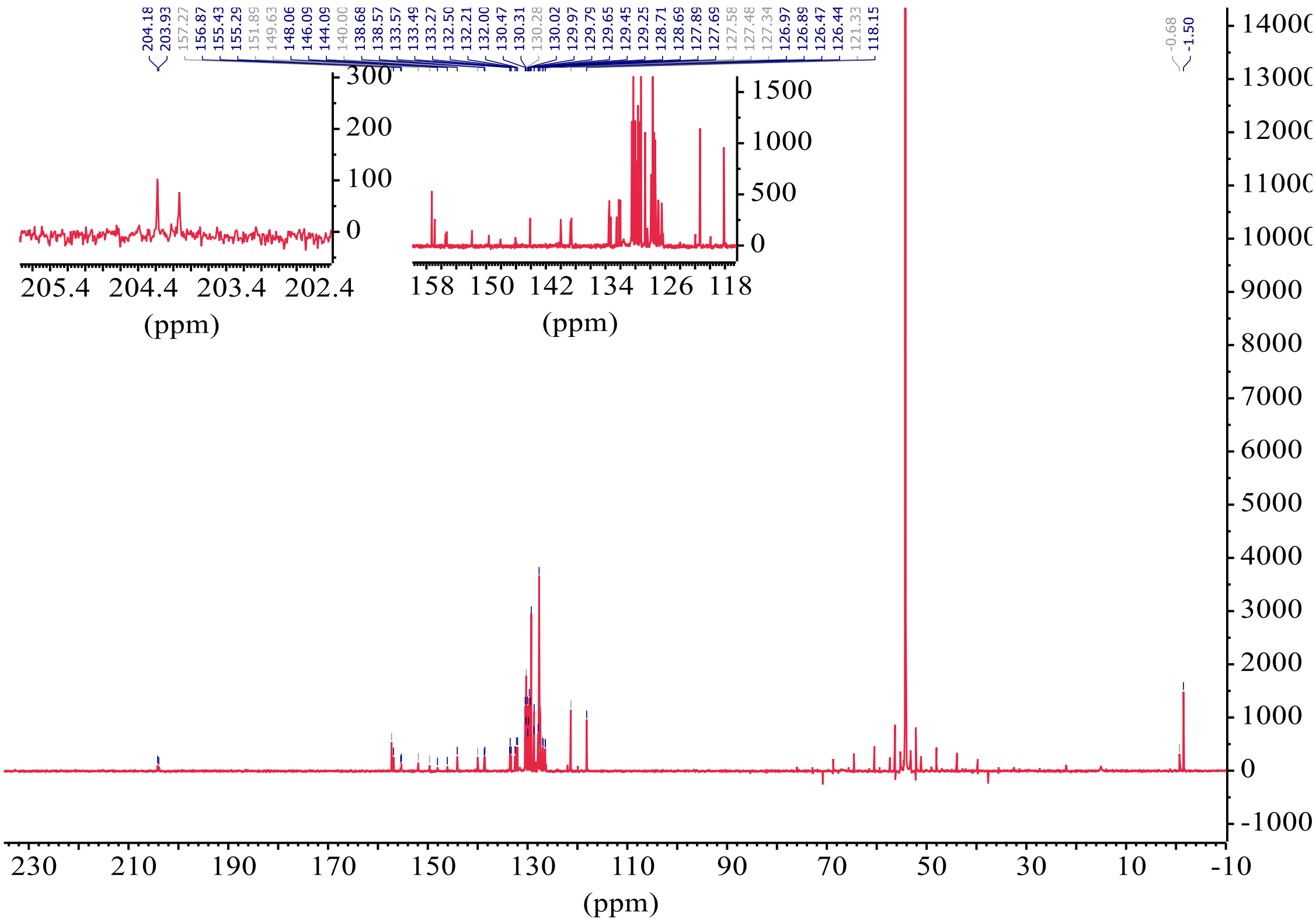

Figure S114: $^{13}C\{^1H\}$ NMR (125 MHz) spectrum of **3j-TPPy** in CH_2Cl_2.

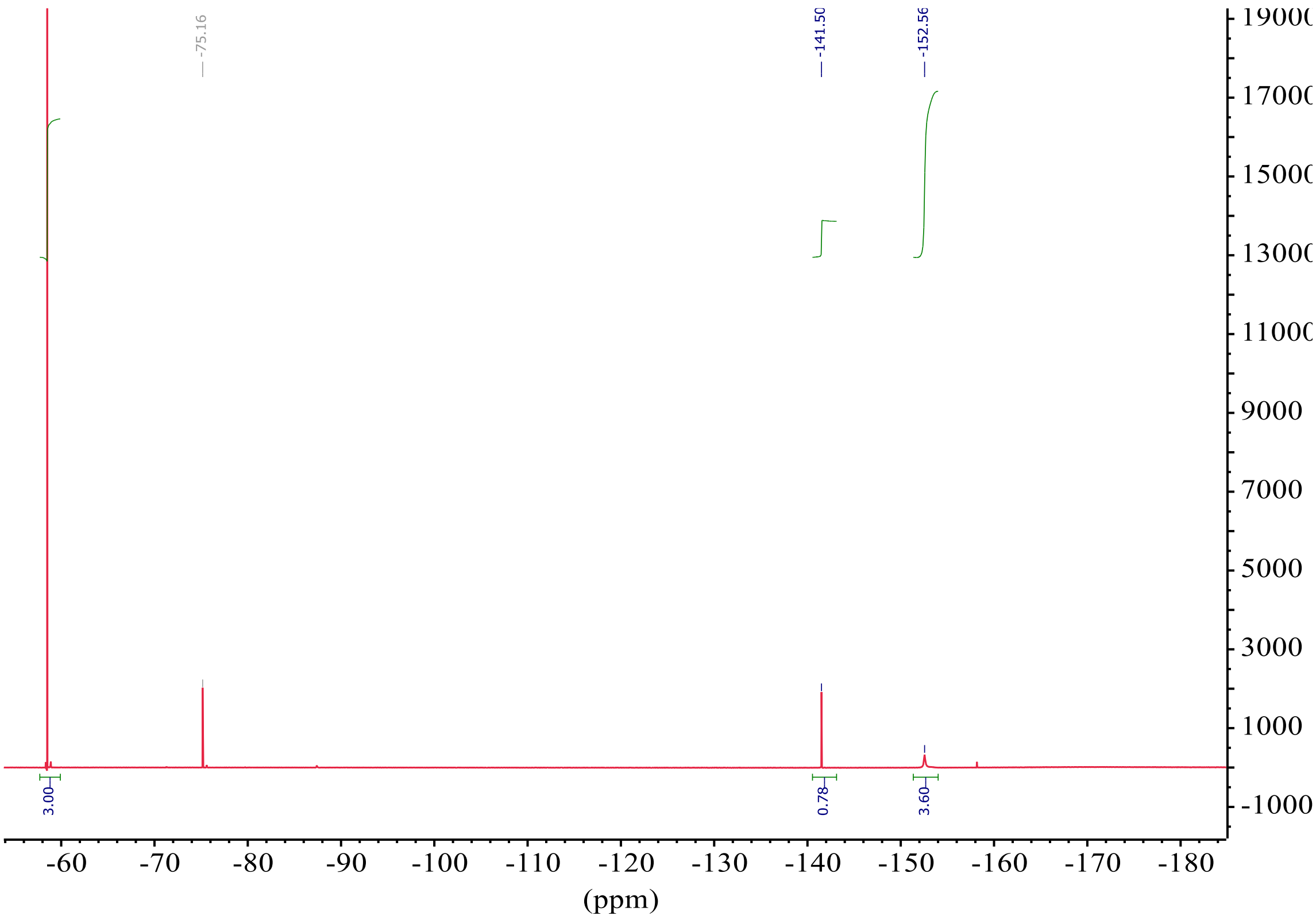

Figure S115: ${}^{19}F\{{}^{1}H\}$ NMR (470 MHz) spectrum of **3j-TPPy** in CH_2Cl_2.

3k-TPPy

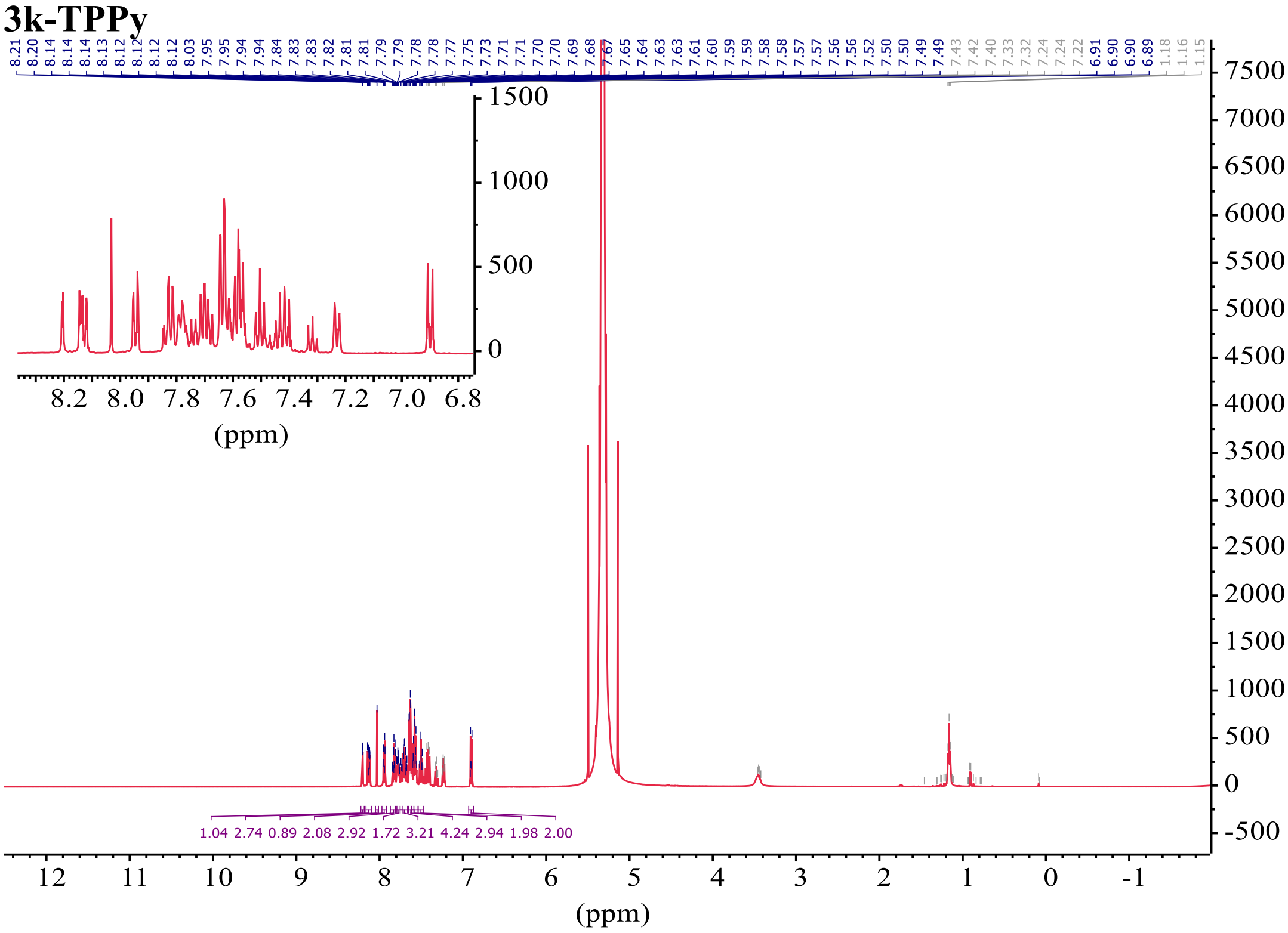

Figure S116: ${}^{1}H$ NMR (500 MHz) spectrum of **3k-TPPy** in CH_2Cl_2. Residual solvent impurities are marked with grey ticks.

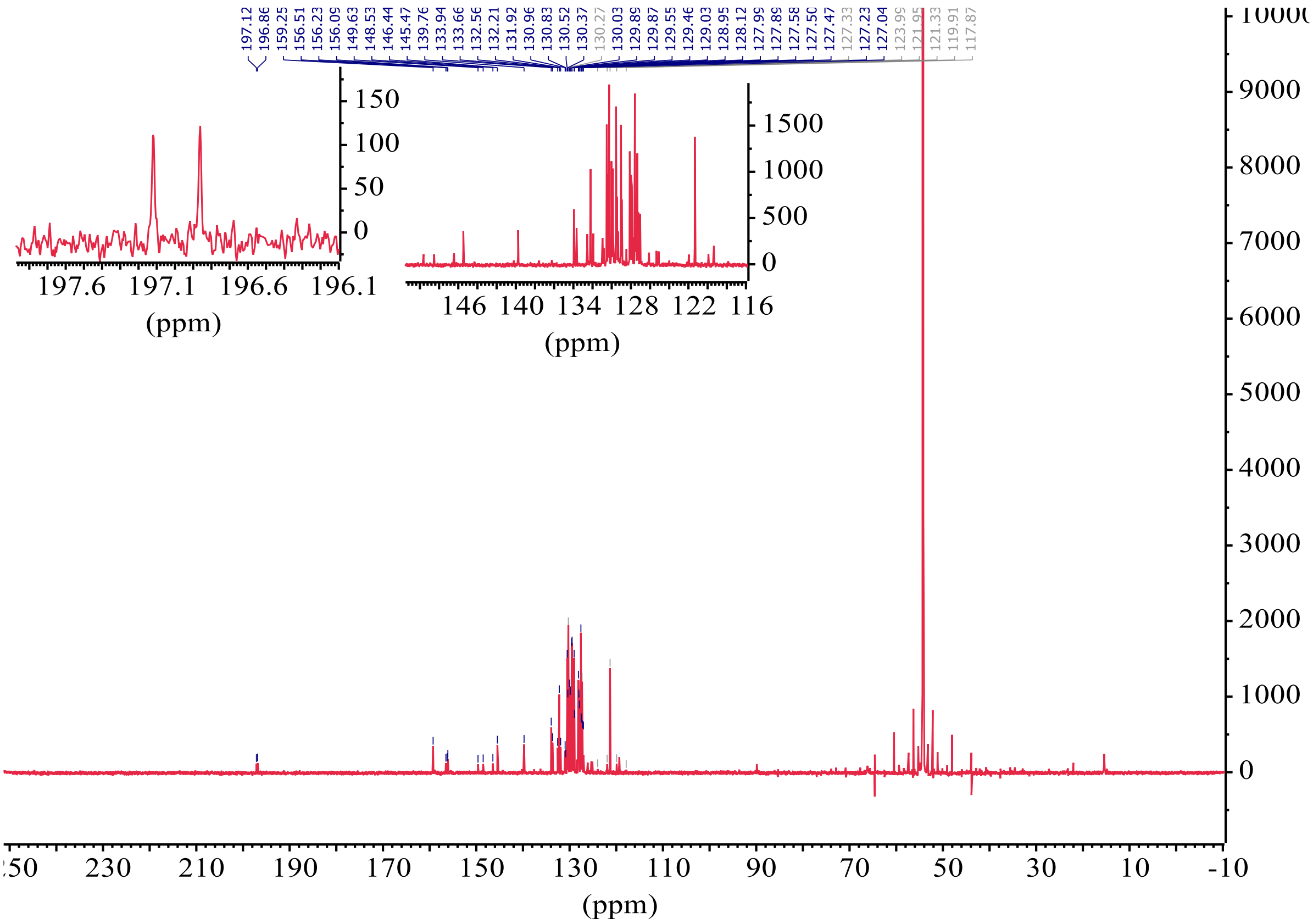

Figure S117: $^{13}C\{^{1}H\}$ NMR (125 MHz) spectrum of **3k-TPPy** in CH_2Cl_2.

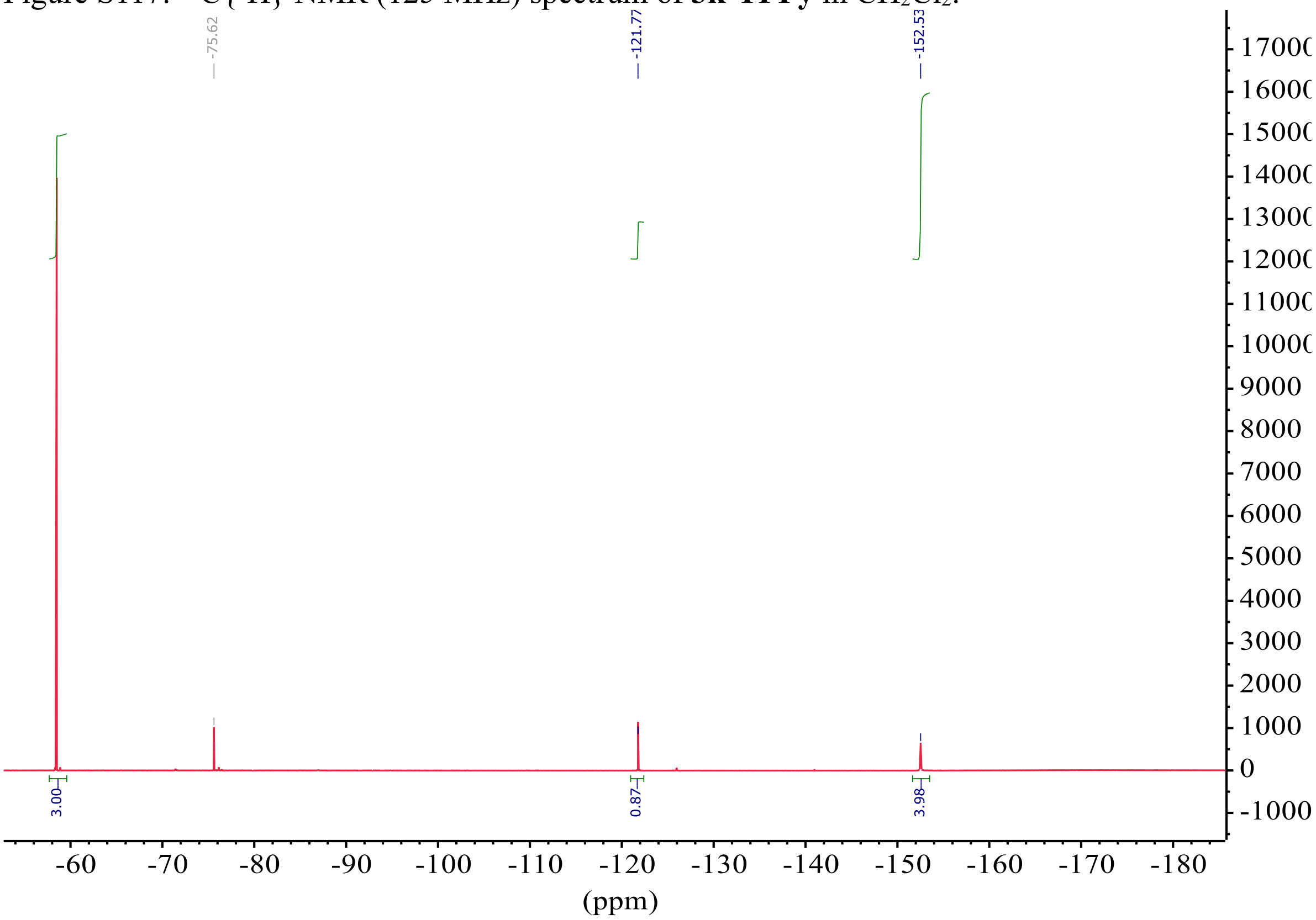

Figure S118: $^{19}F\{^{1}H\}$ NMR (470 MHz) spectrum of **3k-TPPy** in CH_2Cl_2.

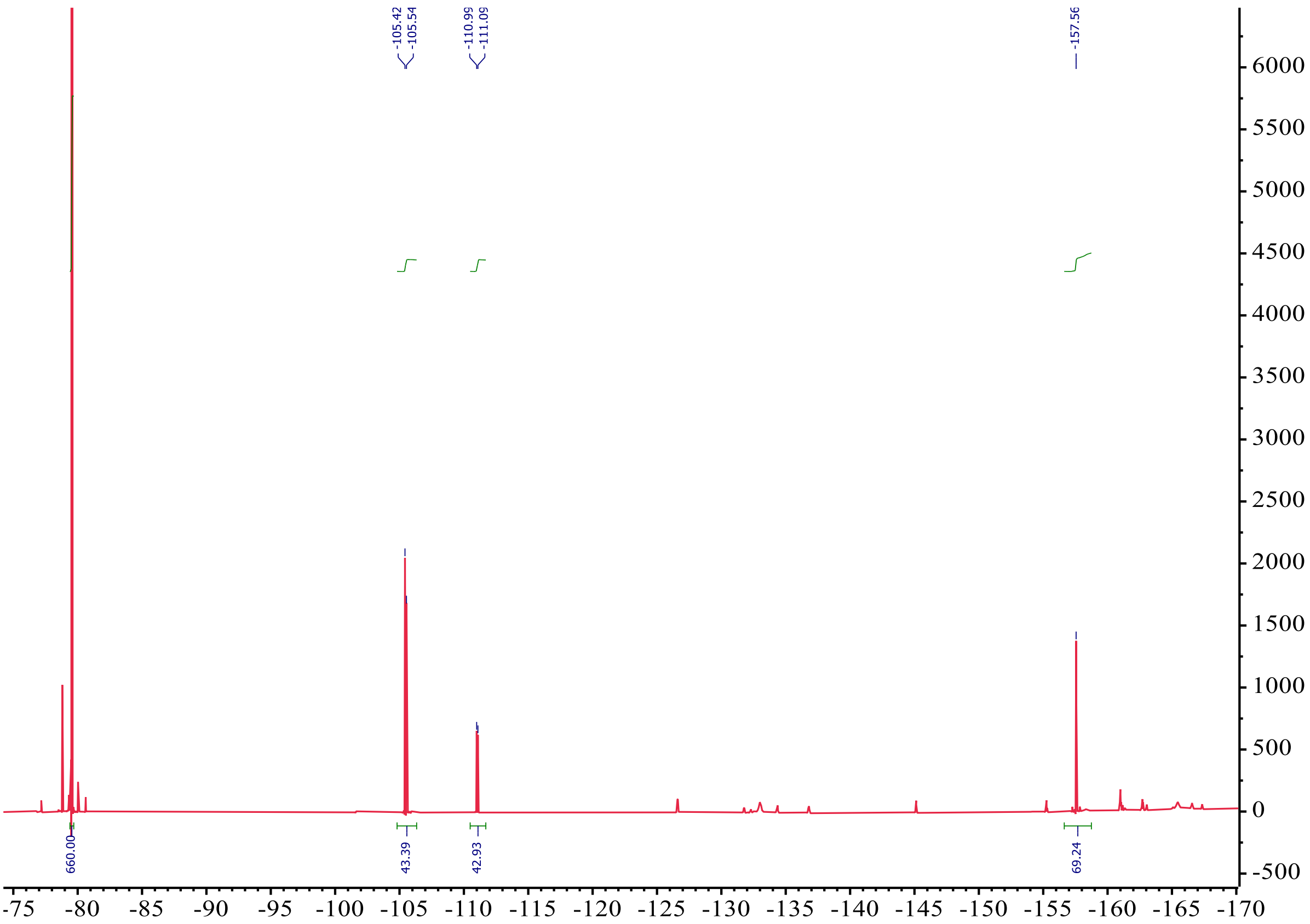

Figure S119: ^{19}F NMR (470 MHz) spectrum of crude reaction of **2m-TPPy** in CH_2Cl_2. Signal at -105.5 corresponds to **1m** and signal at -111.0 corresponds to **2m-TPPy**.

3a-THT

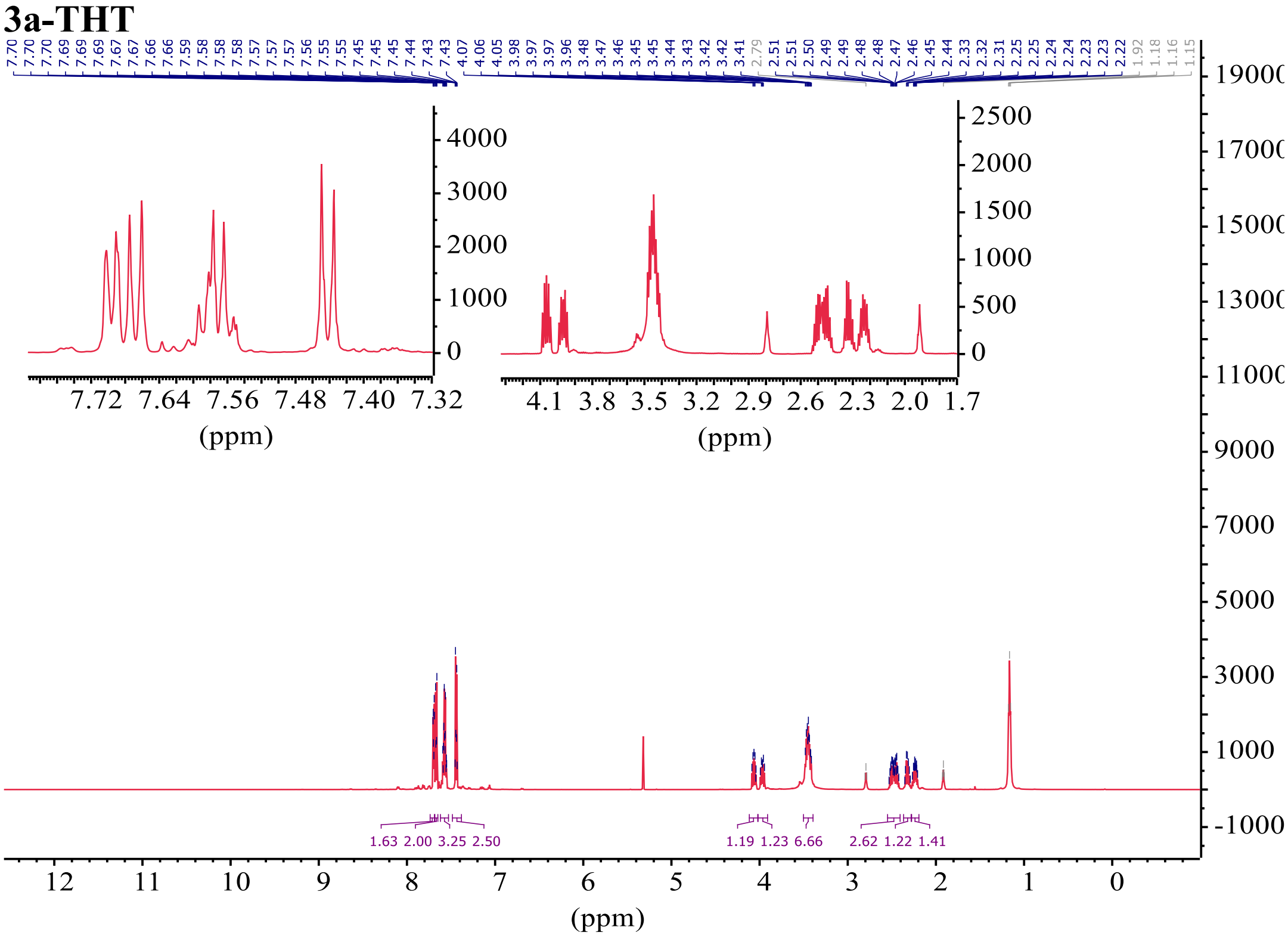

Figure S120: ^{1}H NMR (600 MHz) spectrum of **3a-THT** in CD_2Cl_2. Residual solvent impurities are marked with grey ticks.

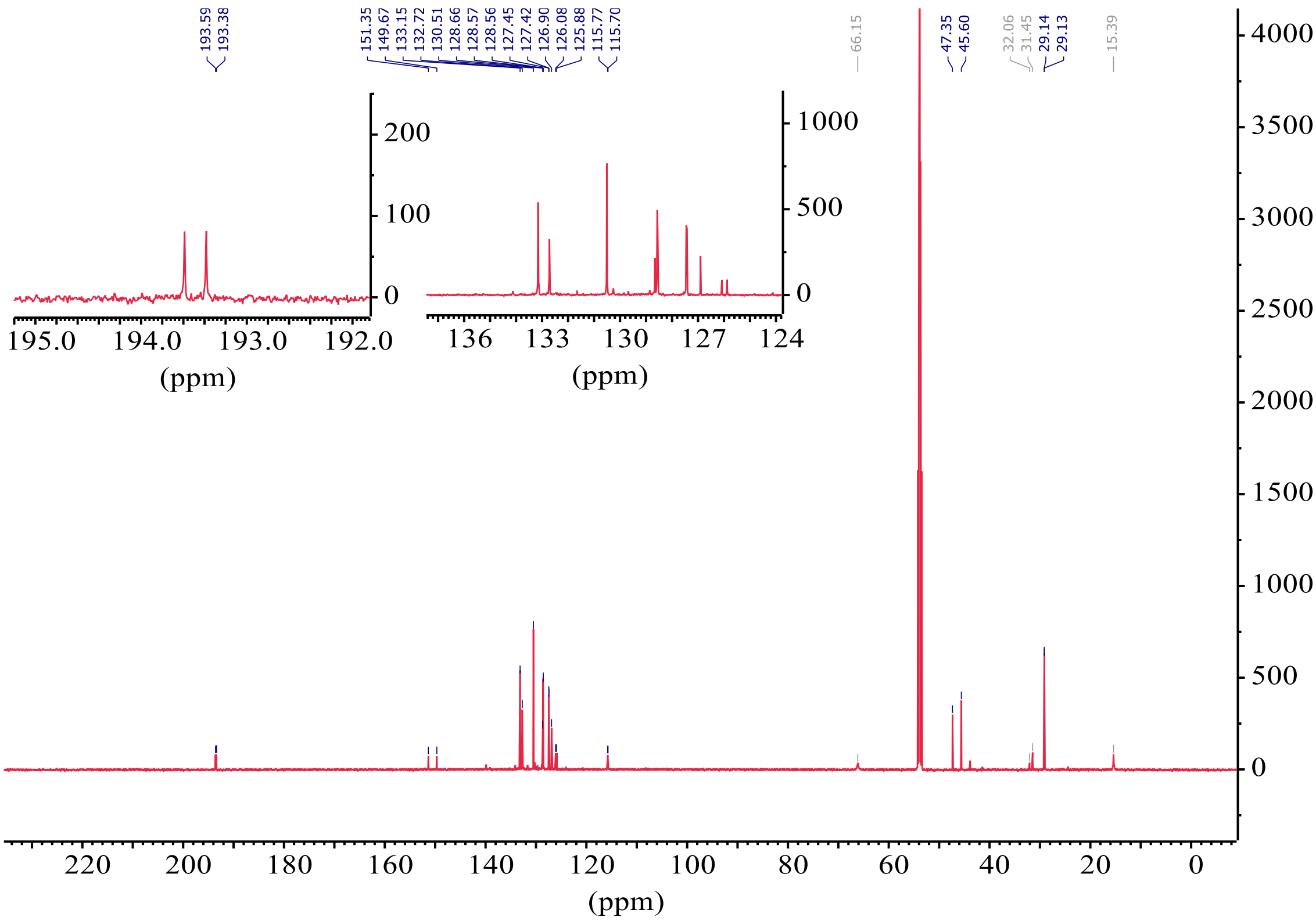

Figure S121: $^{13}C\{^{1}H\}$ NMR (150 MHz) spectrum of **3a-THT** in CD_2Cl_2.

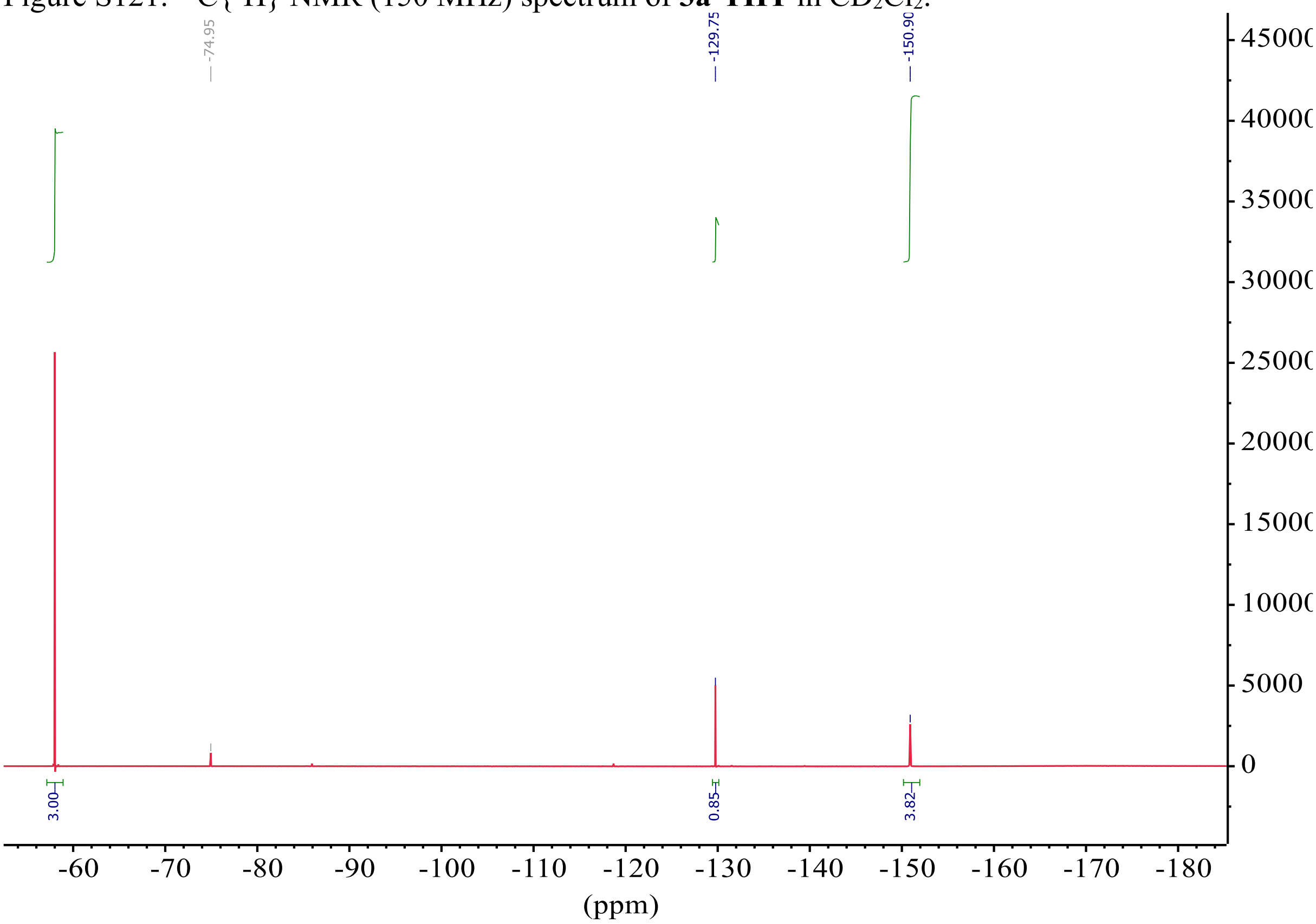

Figure S122: $^{19}F\{^{1}H\}$ NMR (565 MHz) spectrum of **3a-THT** in CD_2Cl_2.

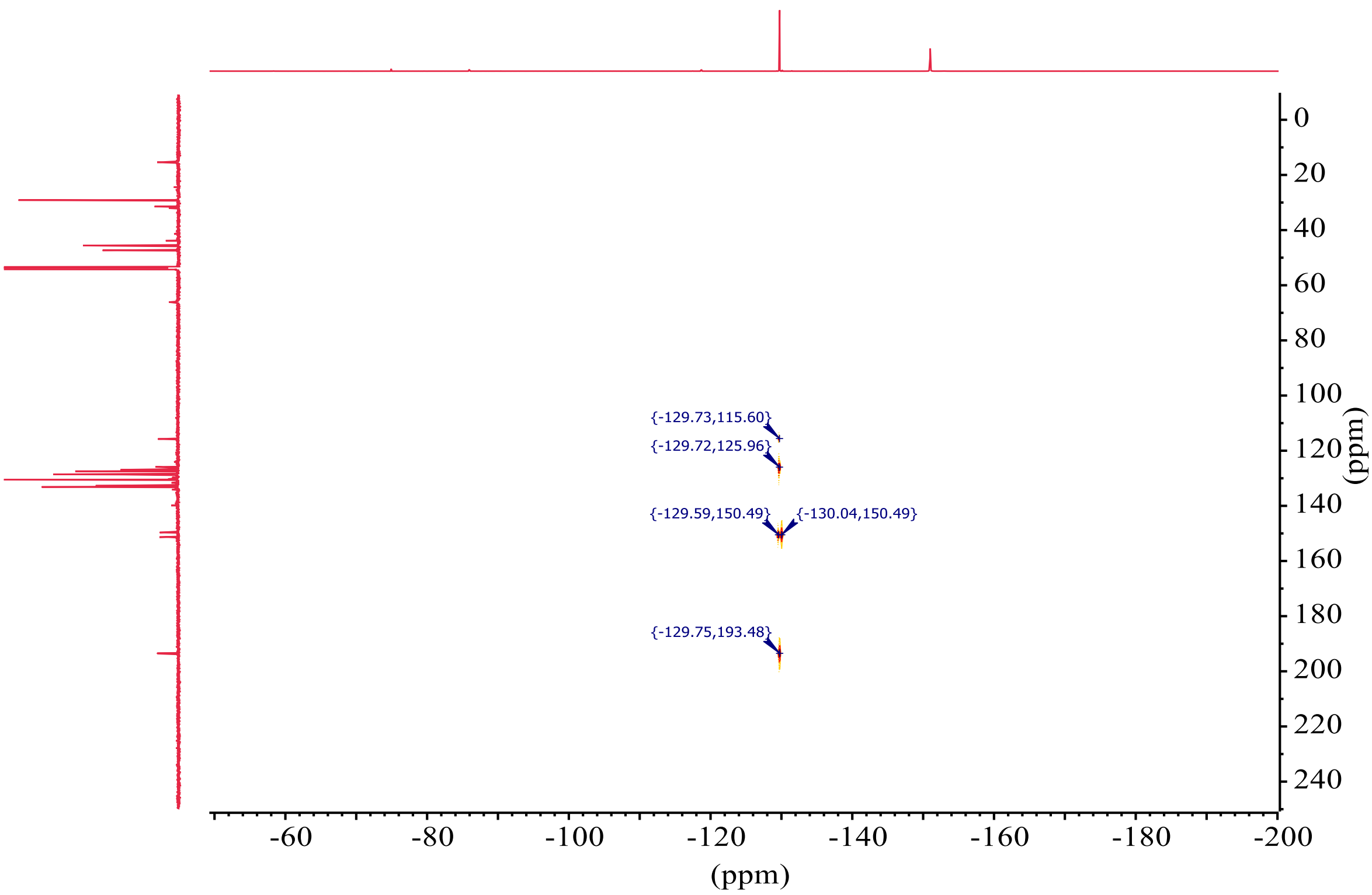

Figure S123: ^{19}F-^{13}C HMBC NMR (565 MHz-150 MHz) spectrum of **3a-THT** in CD_2Cl_2.

3c-THT

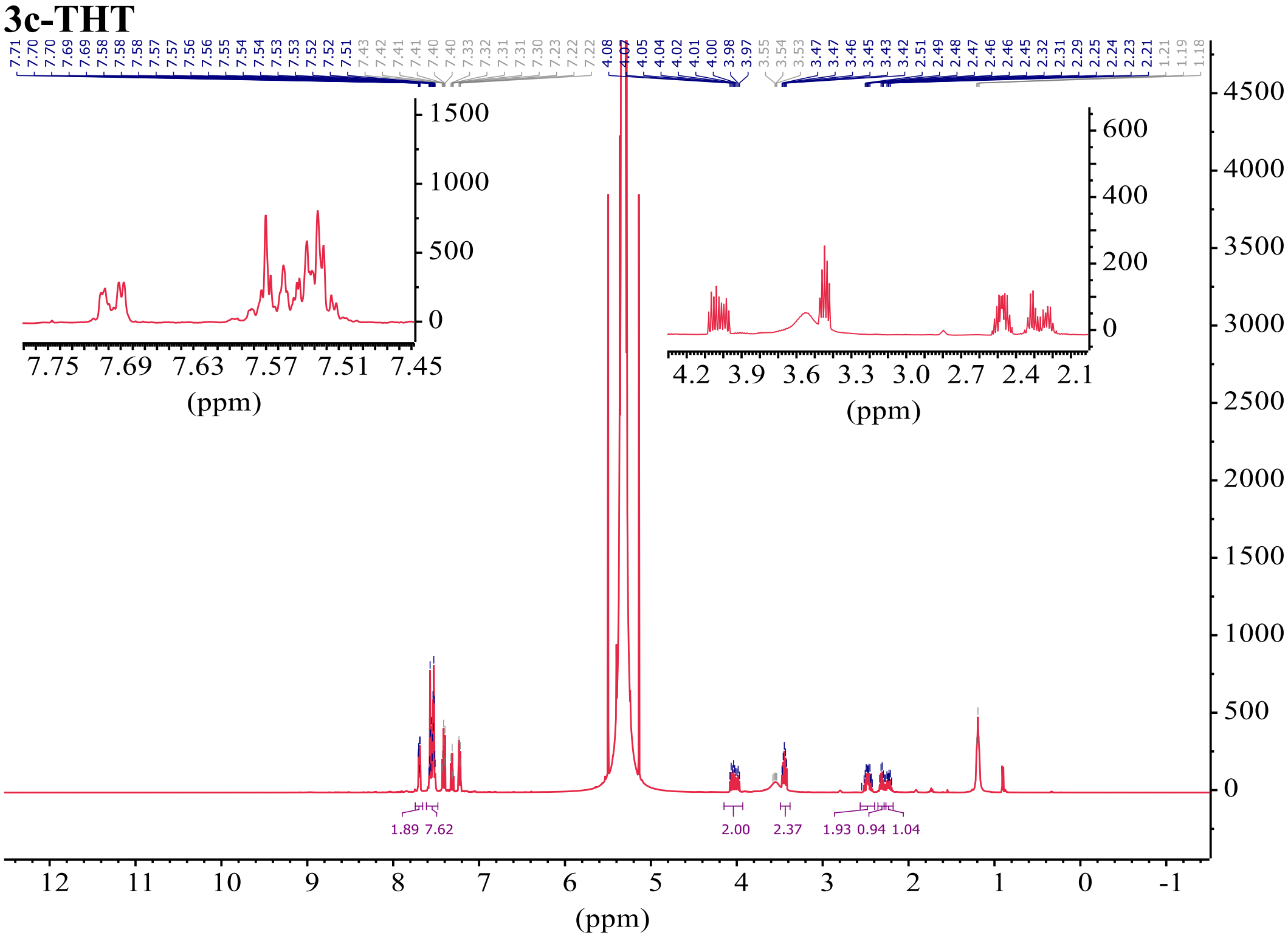

Figure S124: ^{1}H NMR (500 MHz) spectrum of **3c-THT** in CH_2Cl_2. Residual solvent impurities are marked with grey ticks.

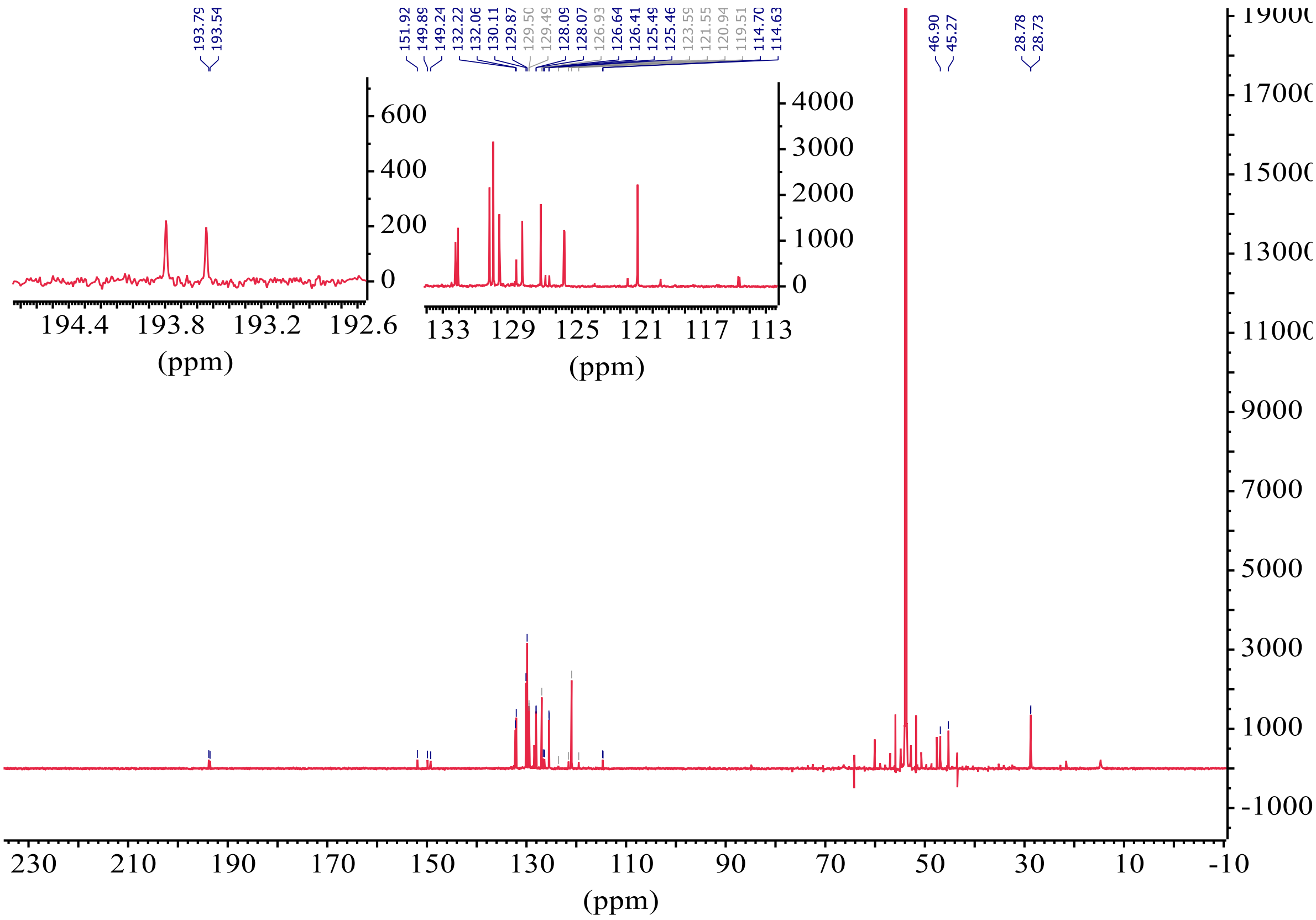

Figure S125: $^{13}C\{^{1}H\}$ NMR (125 MHz) spectrum of **3c-THT** in CH_2Cl_2.

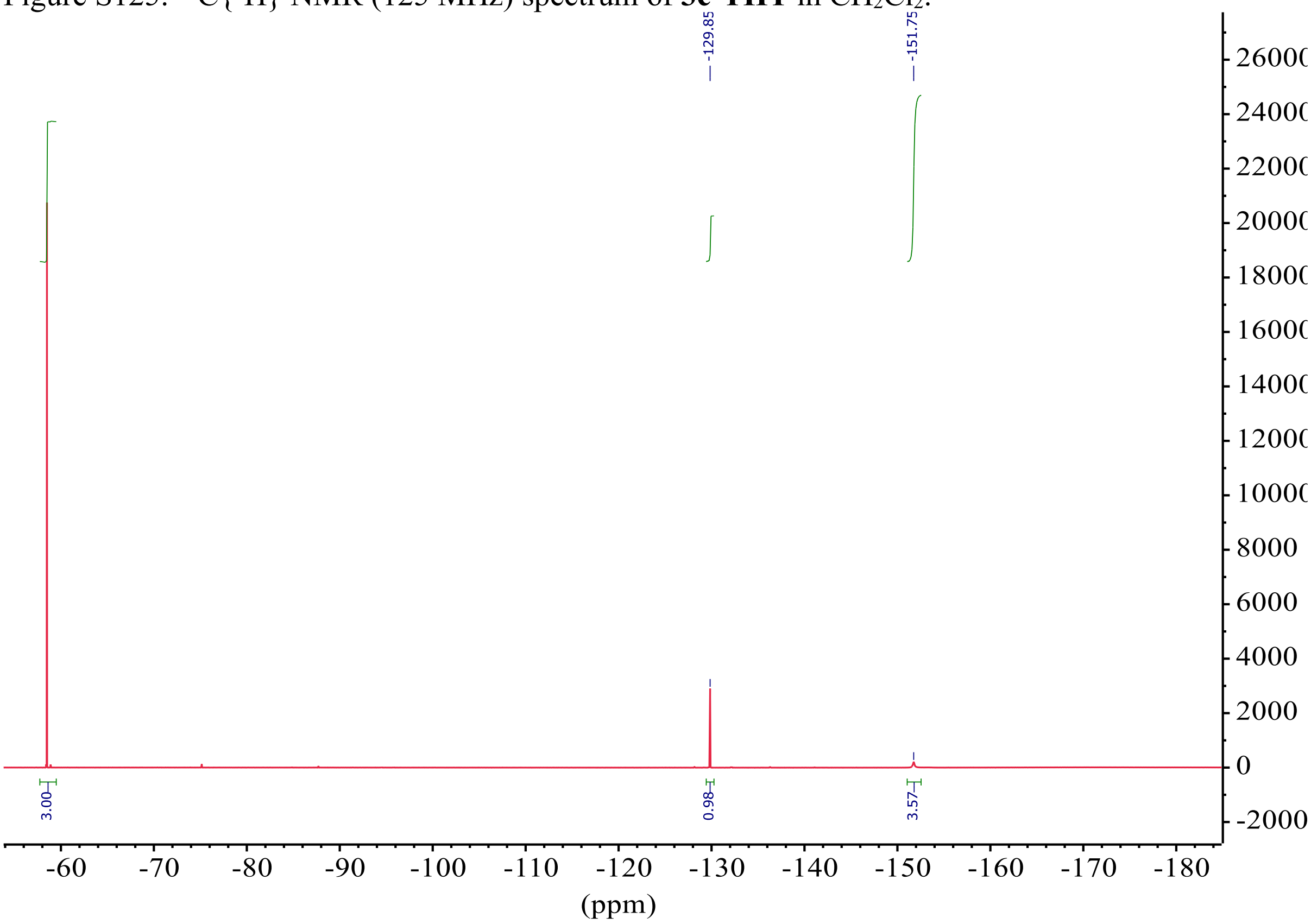

Figure S126: $^{19}F\{^{1}H\}$ NMR (470 MHz) spectrum of **3c-THT** in CH_2Cl_2.

3g-THT

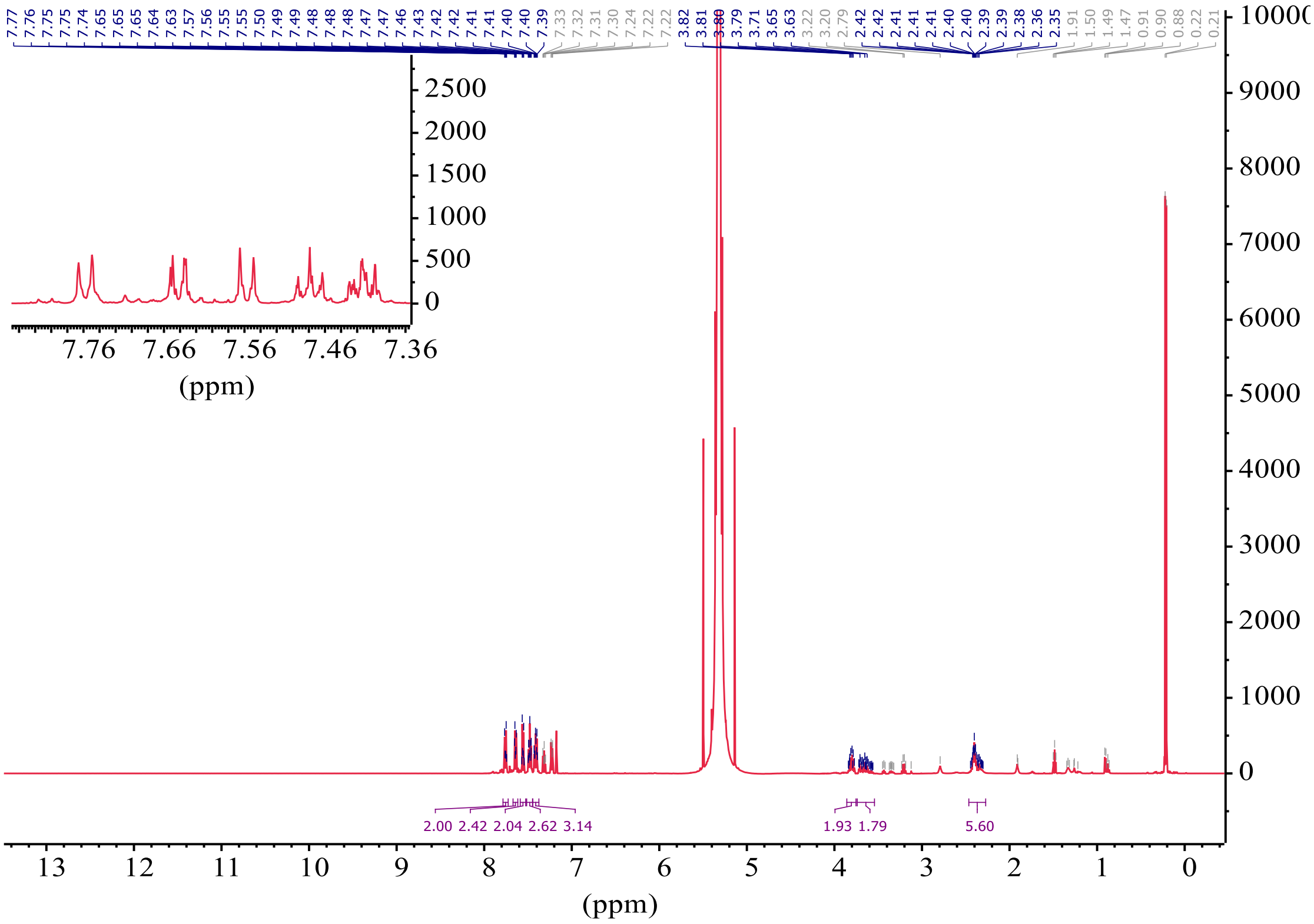

Figure S127: ^{1}H NMR (500 MHz) spectrum of **3g-THT** in CH_2Cl_2. Residual impurities and internal standard are marked with grey ticks.

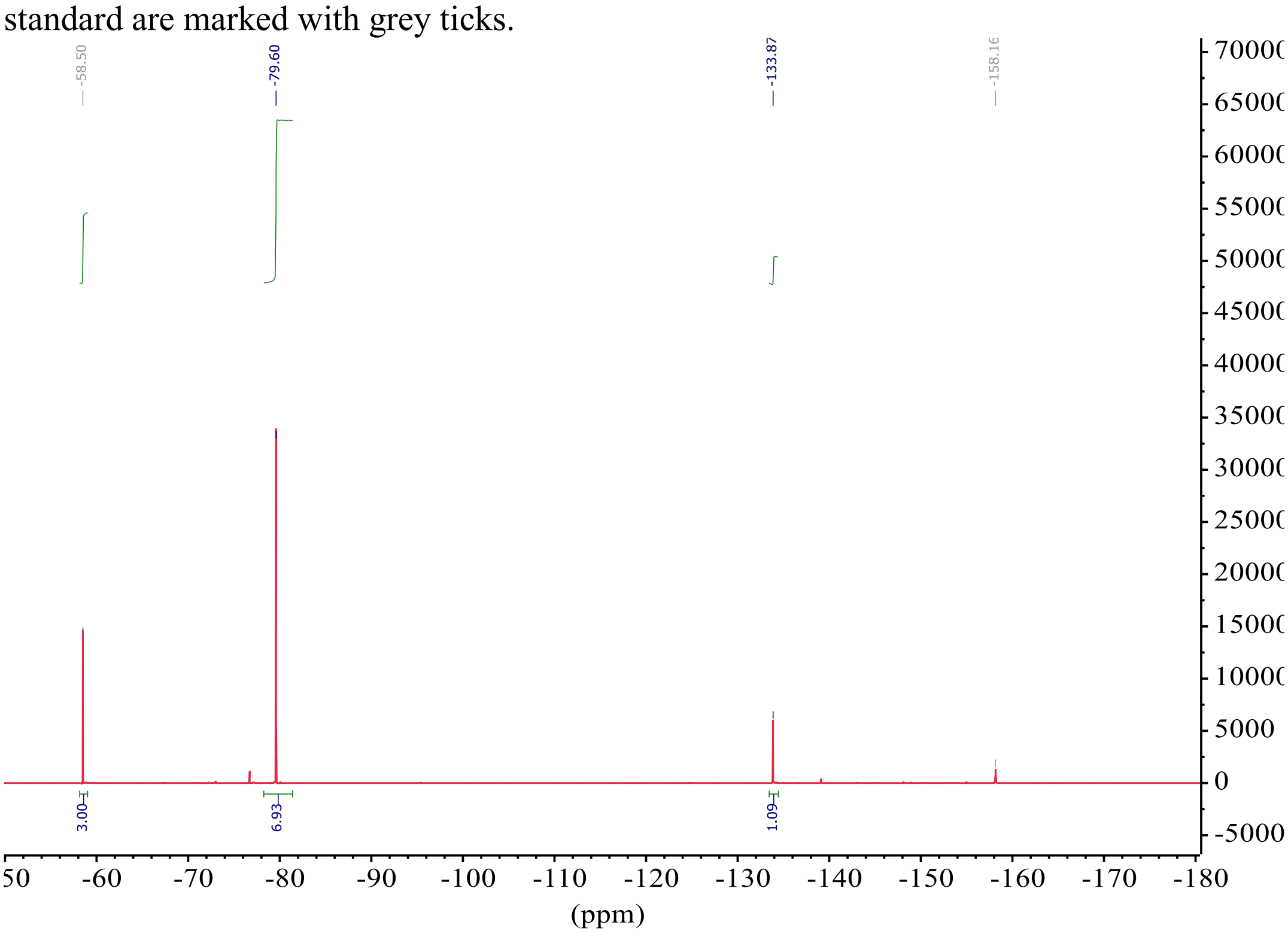

Figure S128: $^{19}F\{^{1}H\}$ NMR (470 MHz) spectrum of **3g-THT** in CH_2Cl_2.

3l-THT

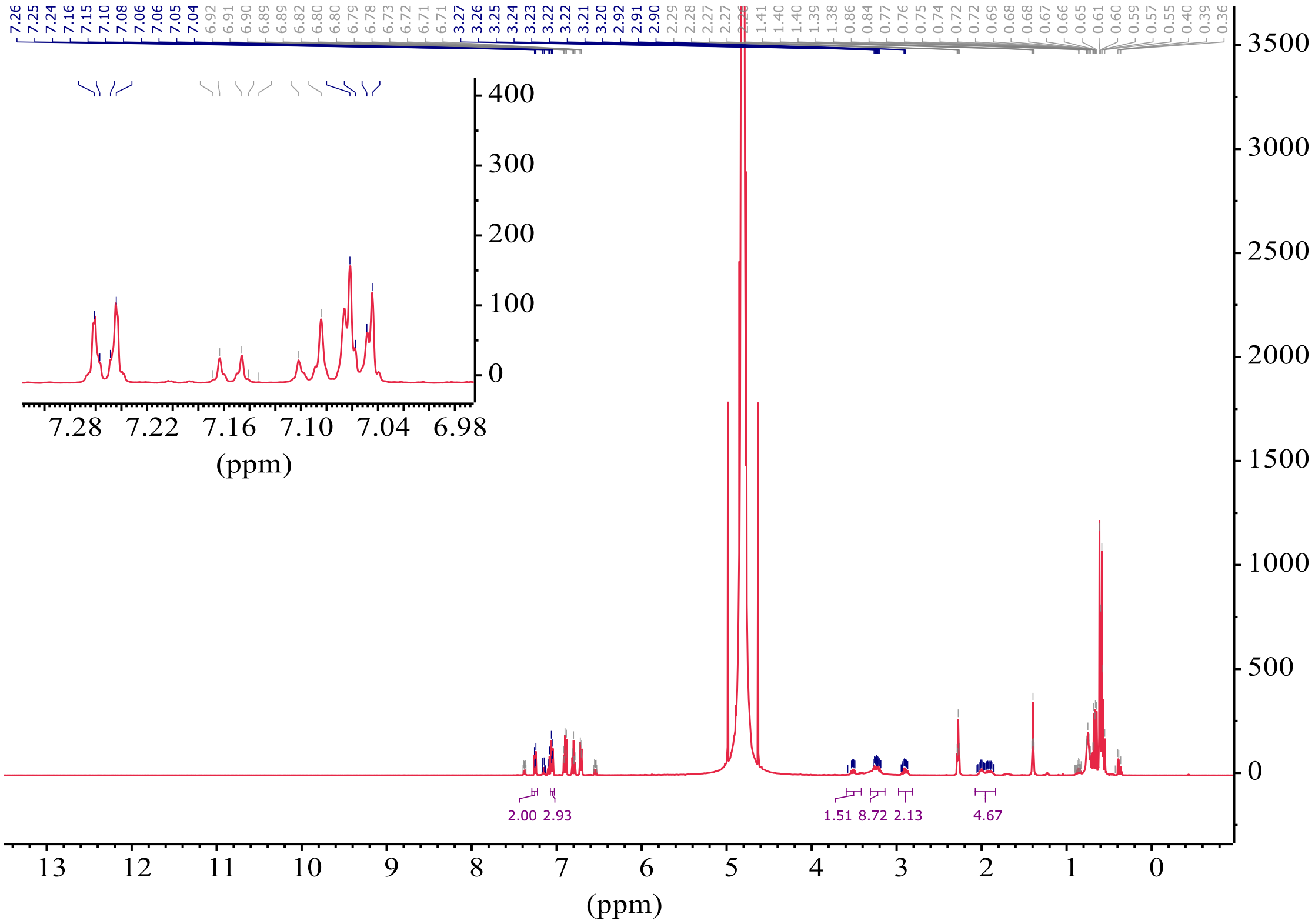

Figure S129: ^{1}H NMR (500 MHz) spectrum of **3l-THT** in CH_2Cl_2. Residual impurities and internal standard are marked with grey ticks.

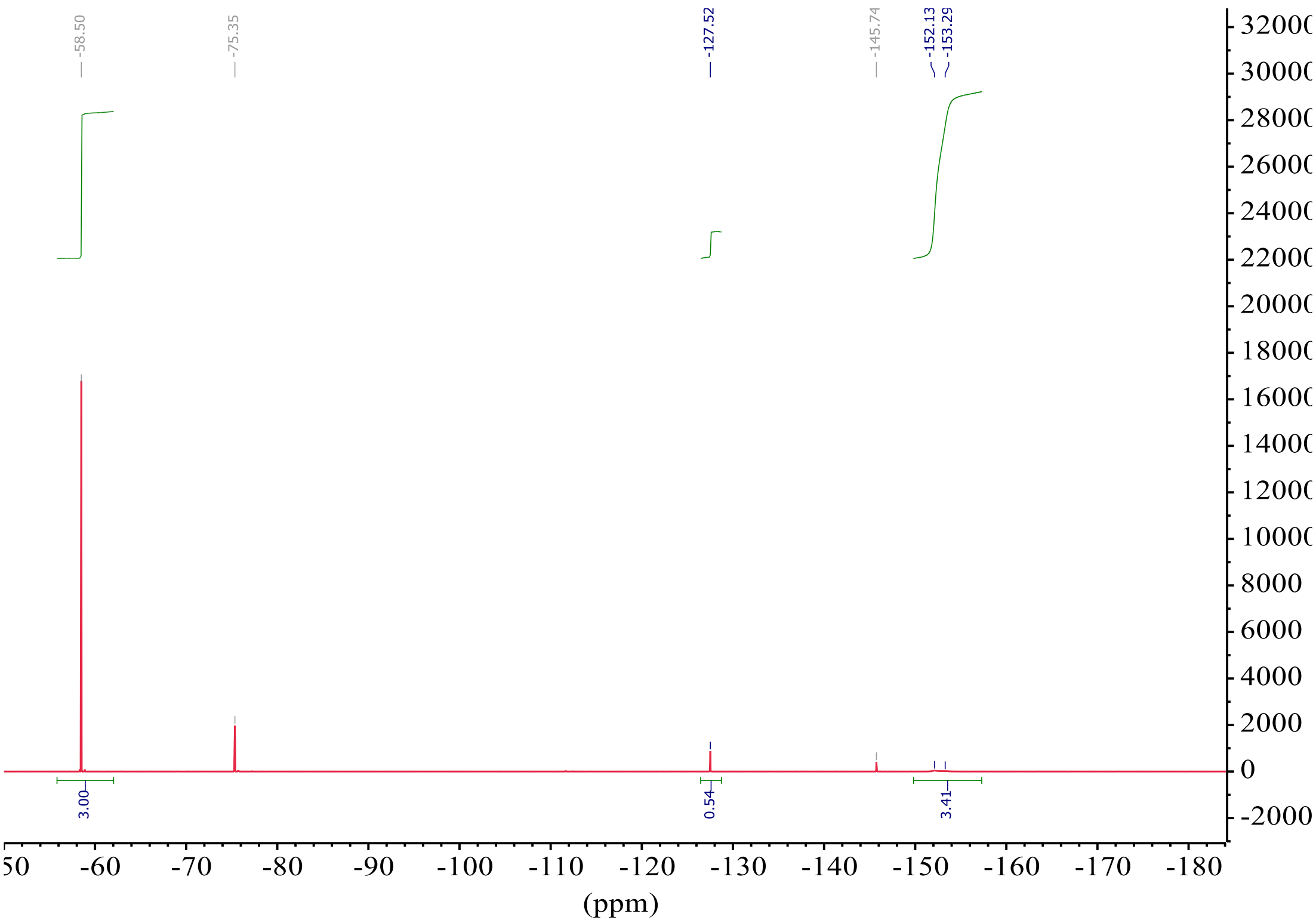

Figure S130: $^{19}F\{^{1}H\}$ NMR (470 MHz) spectrum of **3l-THT** in CH_2Cl_2.

3h-LB$_B$*

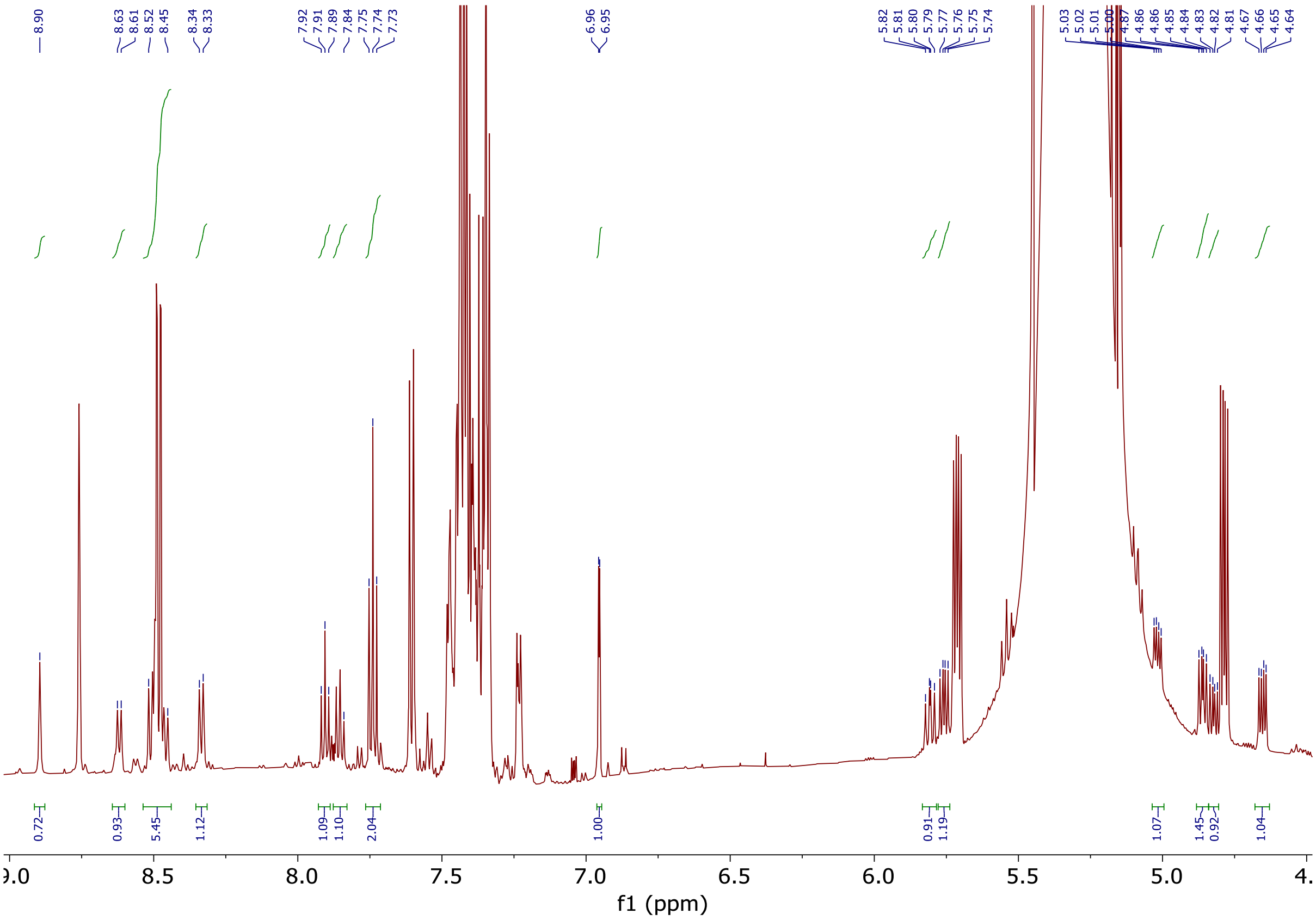

Figure S131: ^{1}H NMR (500 MHz) crude spectrum of **3h-LB$_B$*** in CH_2Cl_2. Signals assigned through correlation spectroscopy.

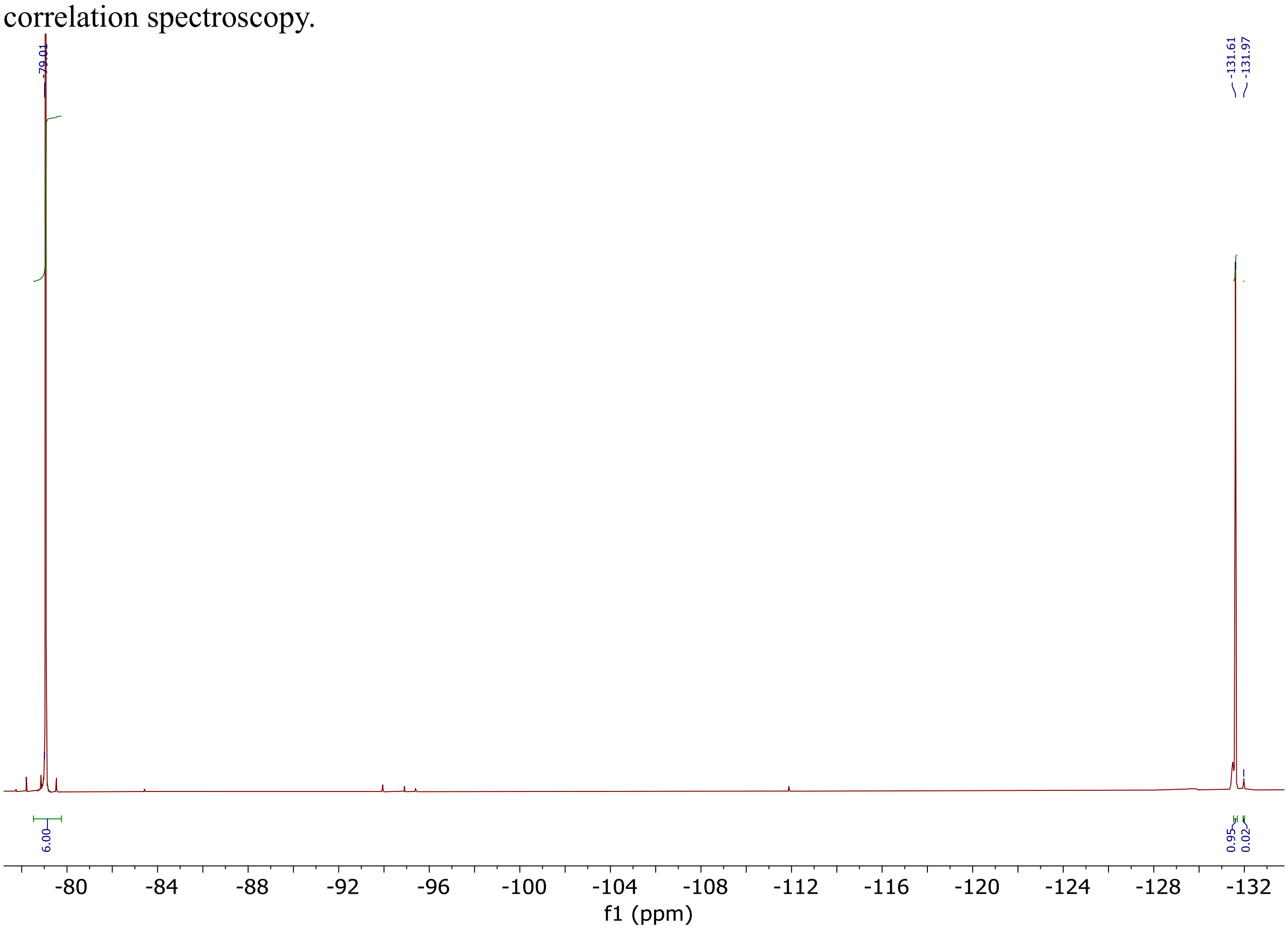

Figure S132: ^{19}F NMR (565 MHz) crude spectrum of **3h-LB$_B$*** in CH_2Cl_2.

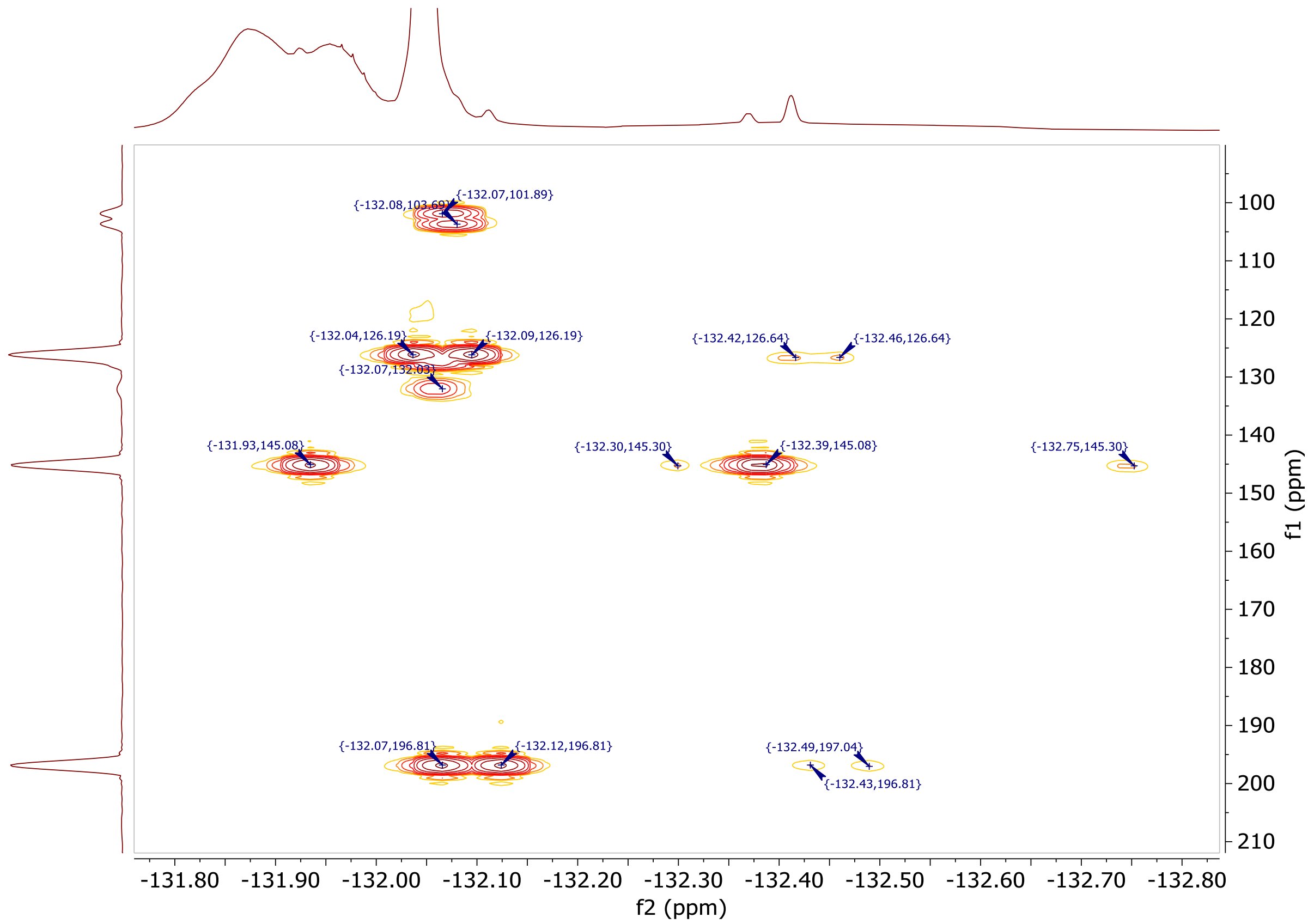

Figure S133: ^{19}F-^{13}C HMBC NMR (565 MHz-150 MHz) crude spectrum of **3h-LB$_B$*** in CH_2Cl_2.

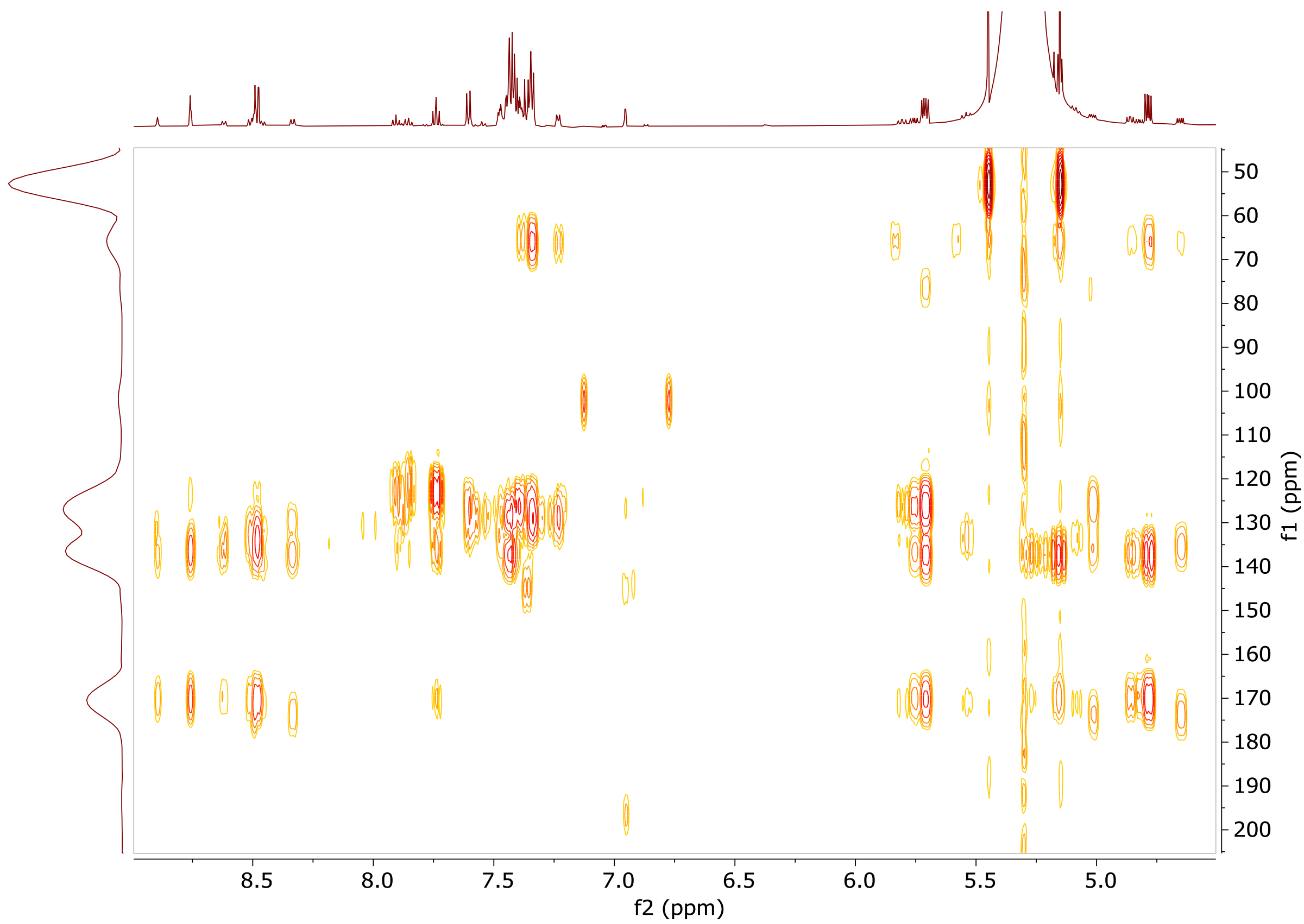

Figure S134: ^{1}H-^{13}C HMBC NMR (500 MHz-150 MHz) crude spectrum of **3h-LB$_B$*** in CH_2Cl_2.

2a-Py

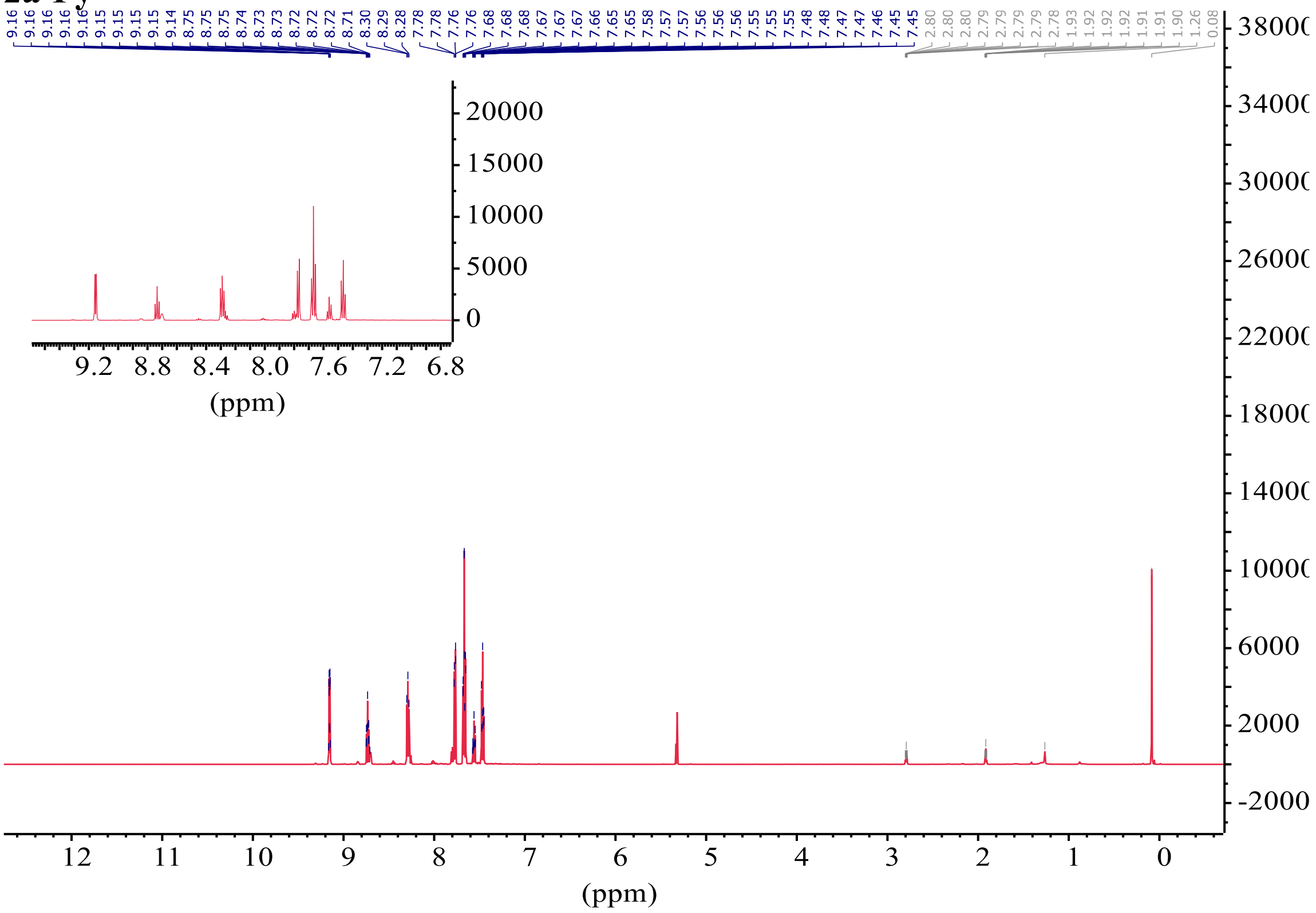

Figure S135: ^{1}H NMR (600 MHz) spectrum of **2a-Py** in CD_2Cl_2. Residual solvent impurities are marked with grey ticks.

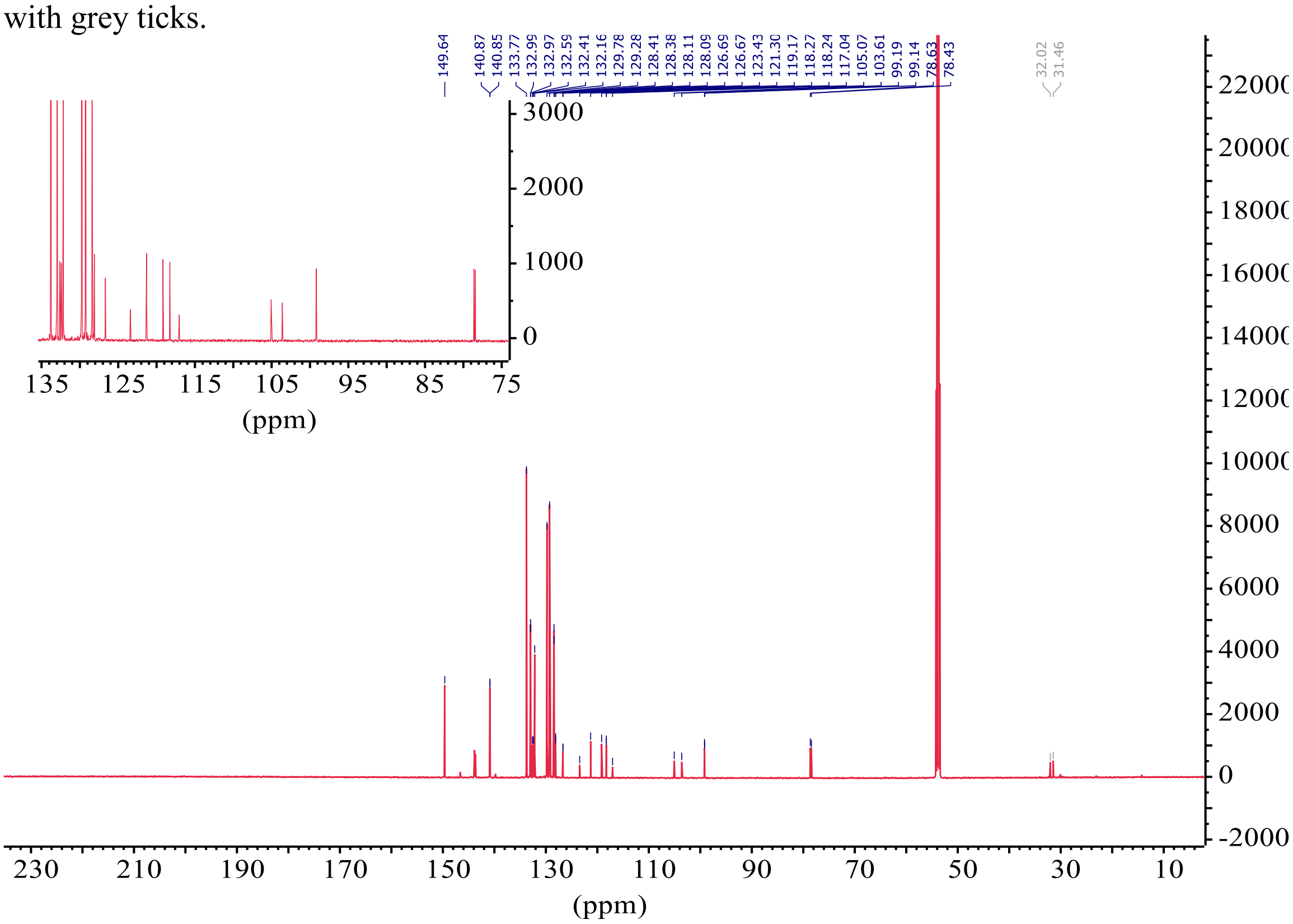

Figure S136: ^{13}C{^{1}H} NMR (150 MHz) spectrum of **2a-Py** in CD_2Cl_2.

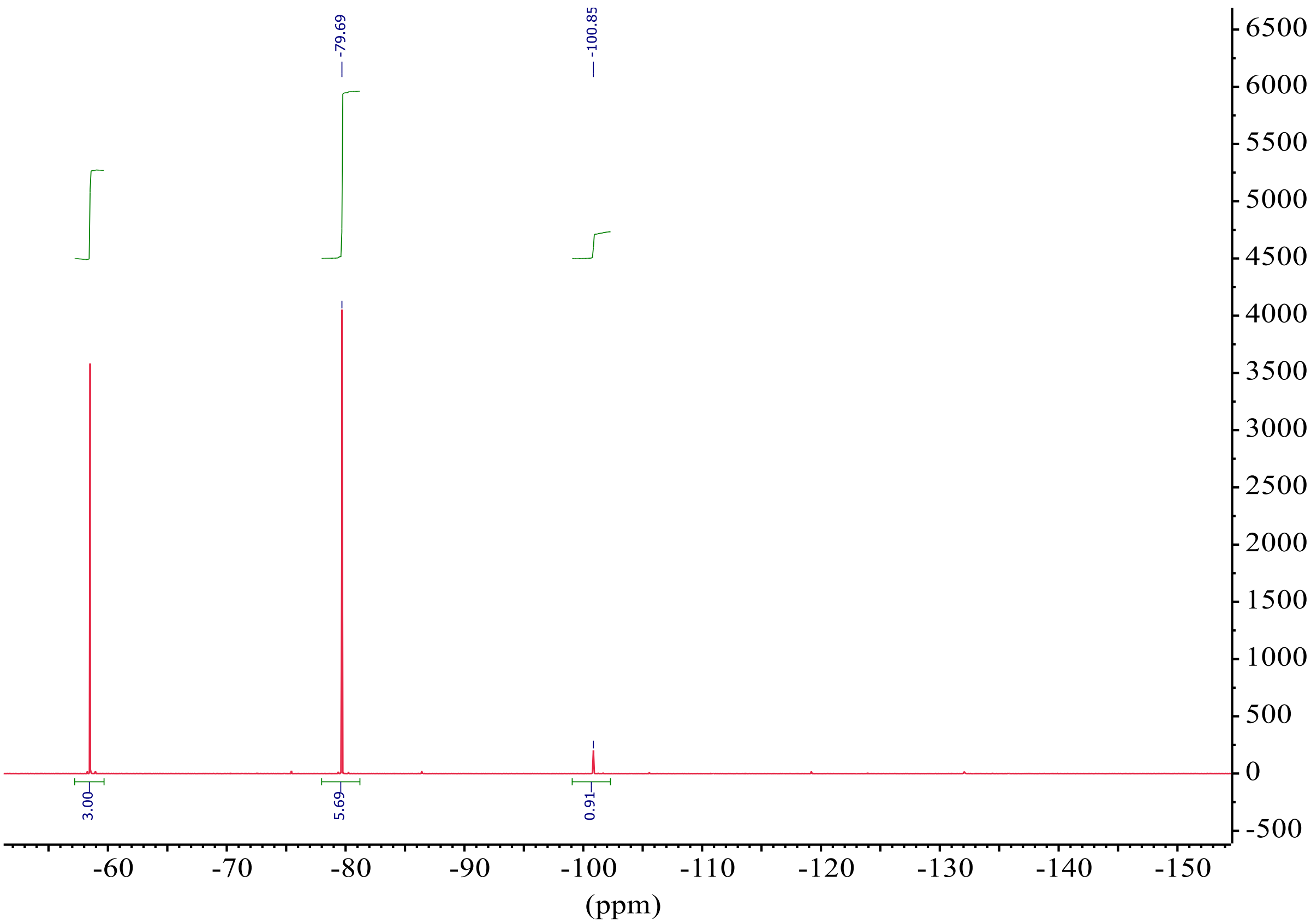

Figure S137: ^{19}F NMR (565 MHz) spectrum of **2a-Py** in CD_2Cl_2.

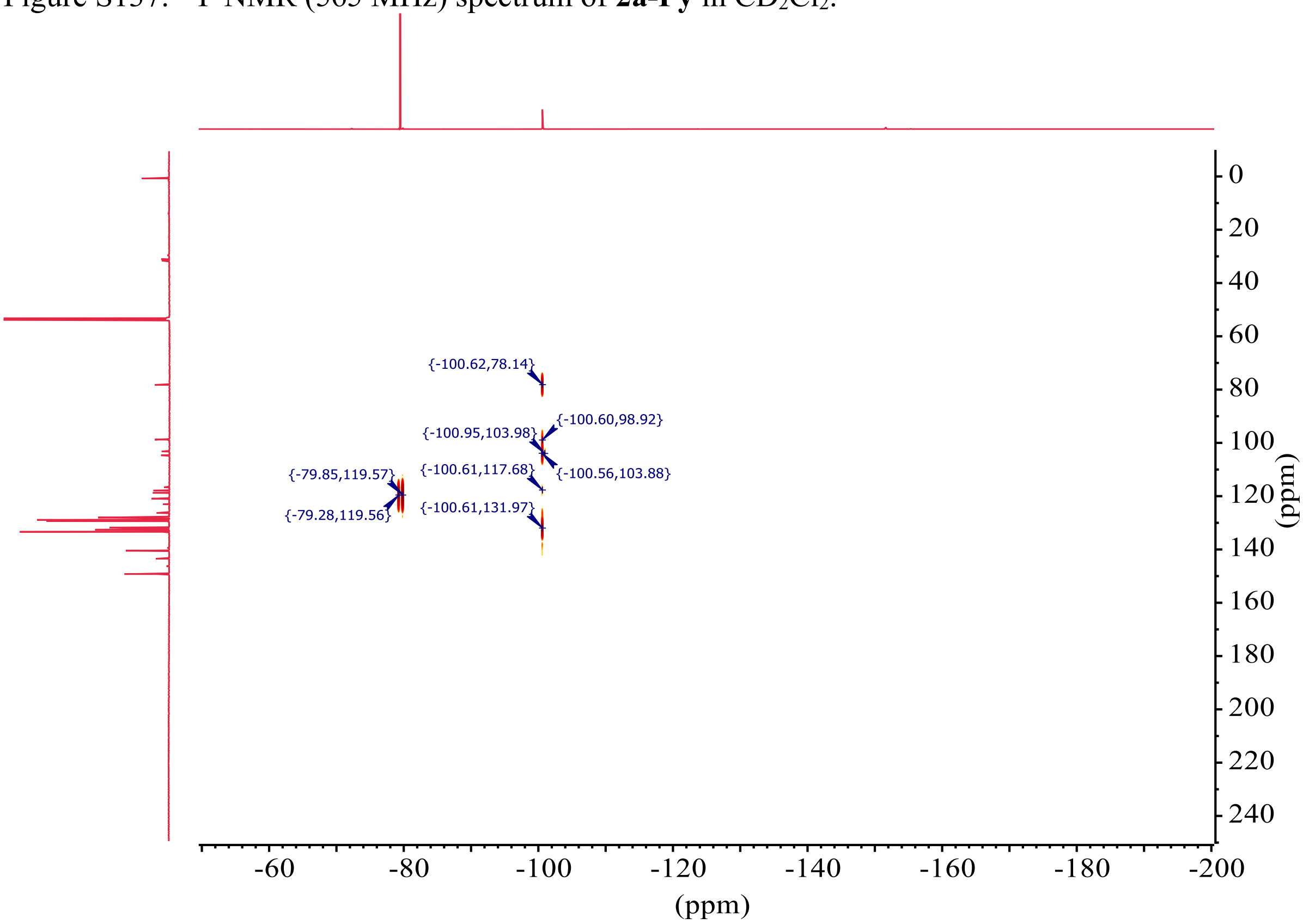

Figure S138: ^{19}F-^{13}C HMBC NMR (565 MHz-150 MHz) spectrum of **2a-Py** in CD_2Cl_2.

3a-PhPy

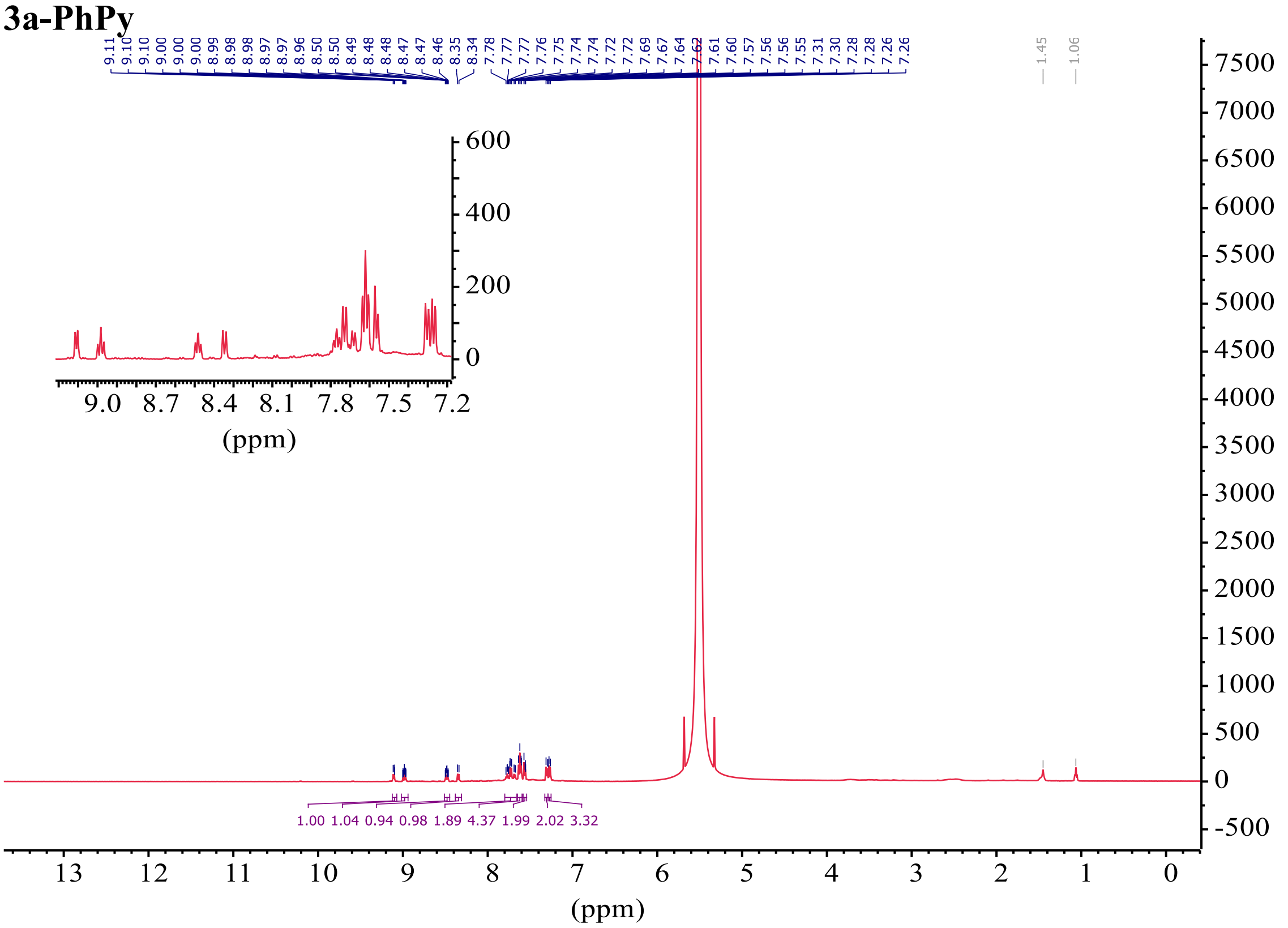

Figure S139: ^{1}H NMR (500 MHz) spectrum of **3a-PhPy** in CH_2Cl_2. Residual impurities are marked with grey ticks.

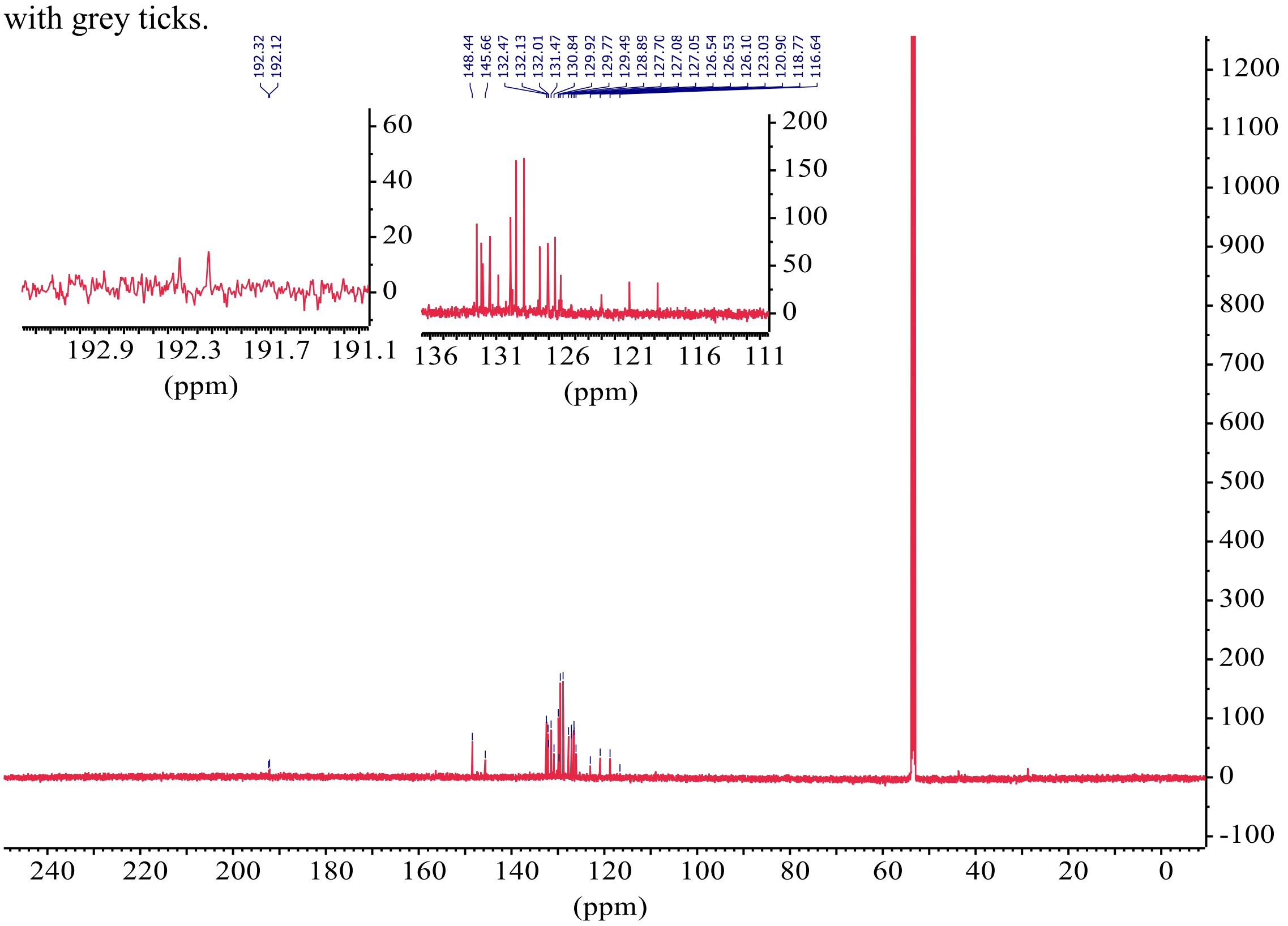

Figure S140: $^{13}C\{^{1}H\}$ NMR (150 MHz) spectrum of **3a-PhPy** in CD_2Cl_2.

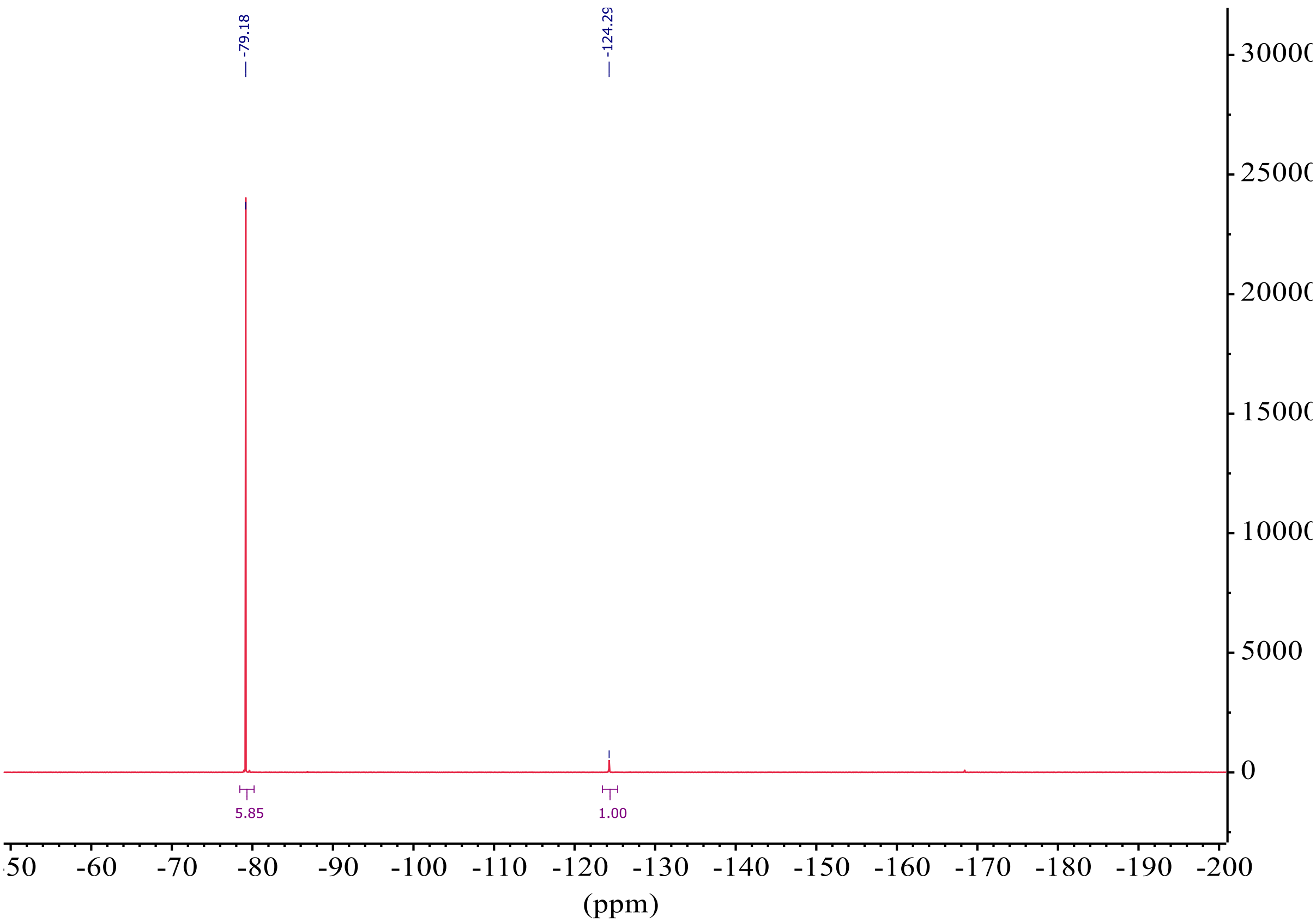

Figure S141: ^{19}F NMR (565 MHz) spectrum of **3a-PhPy** in CD_2Cl_2.

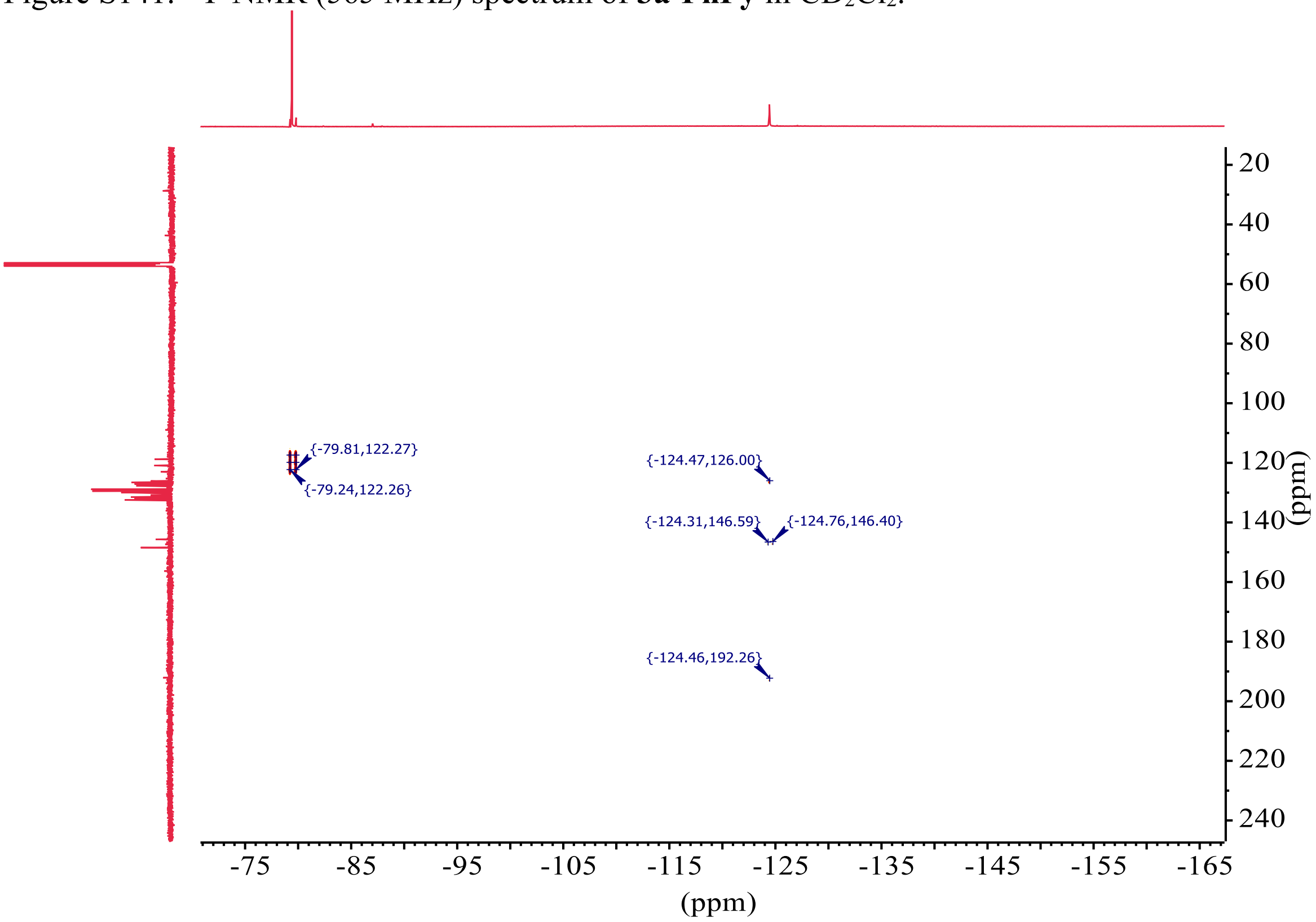

Figure S142: ^{19}F-^{13}C HMBC NMR (565 MHz-150 MHz) spectrum of **3a-PhPy** in CD_2Cl_2.

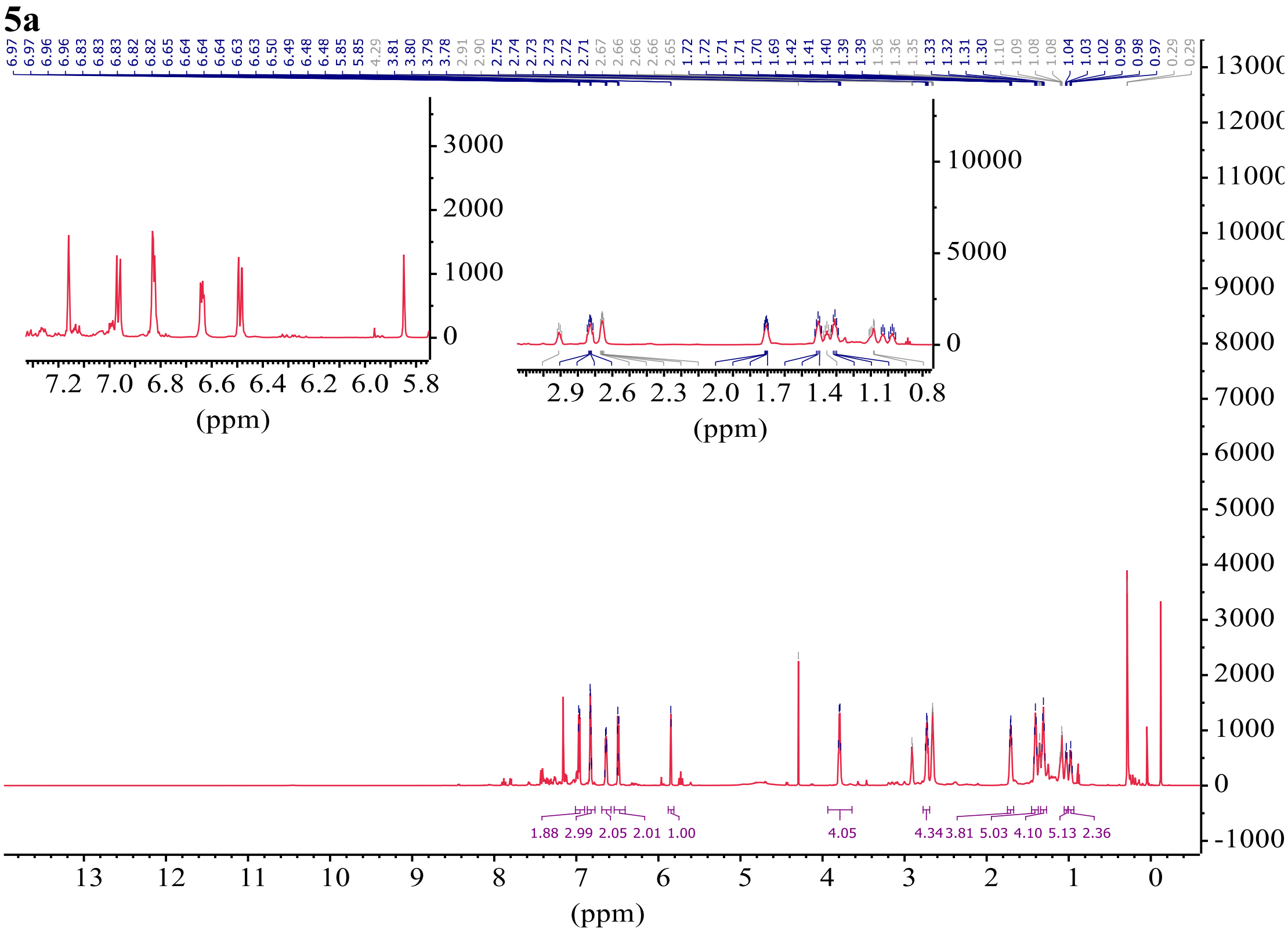

Figure S143: ^{1}H NMR (600 MHz) spectrum of **5a** in C_6D_6. Impurities are marked with grey ticks.

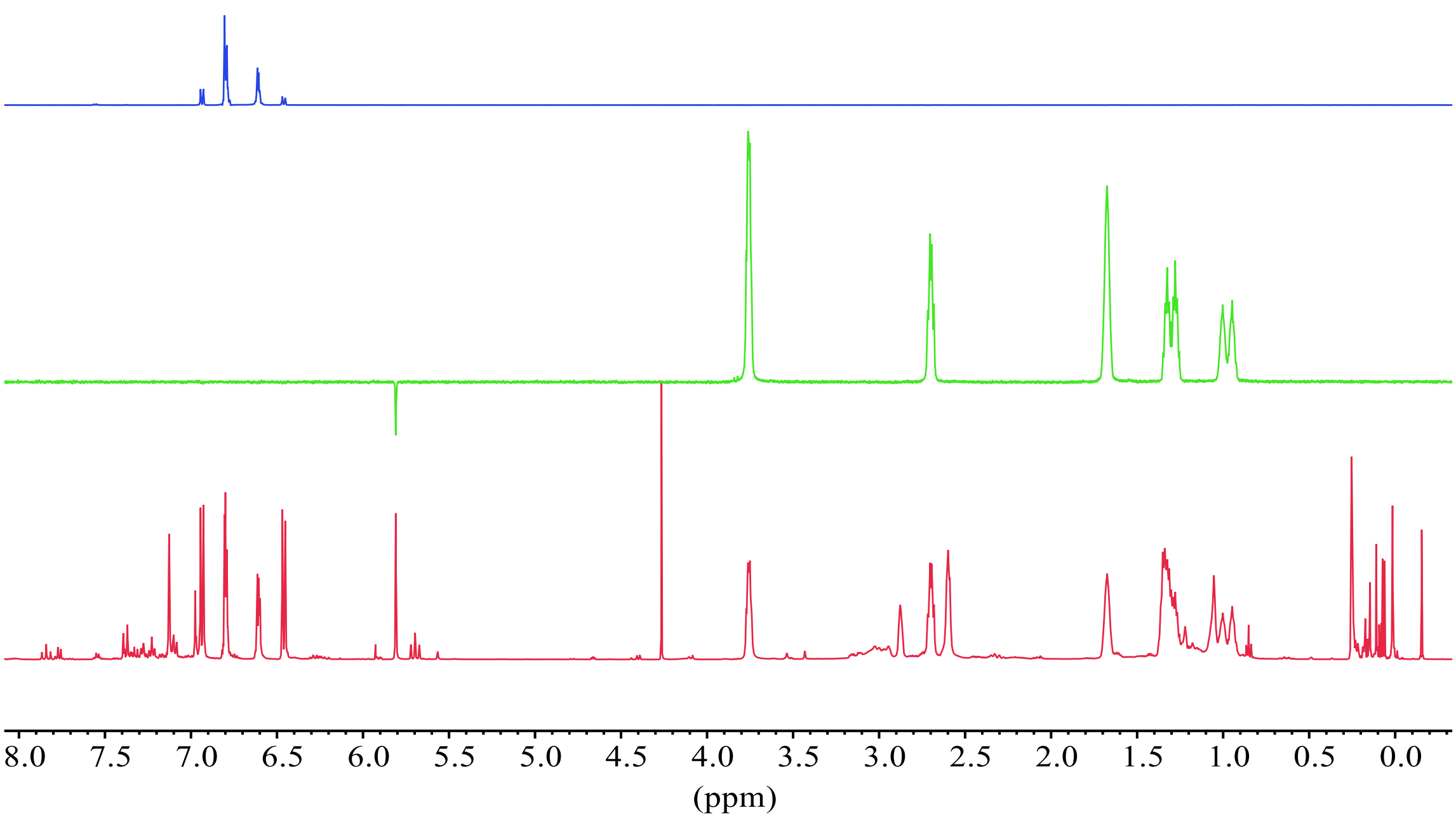

Figure S144: ^{1}H NMR (500 MHz) Selective 1D TOCSY spectrum of **5a** in C_6D_6.

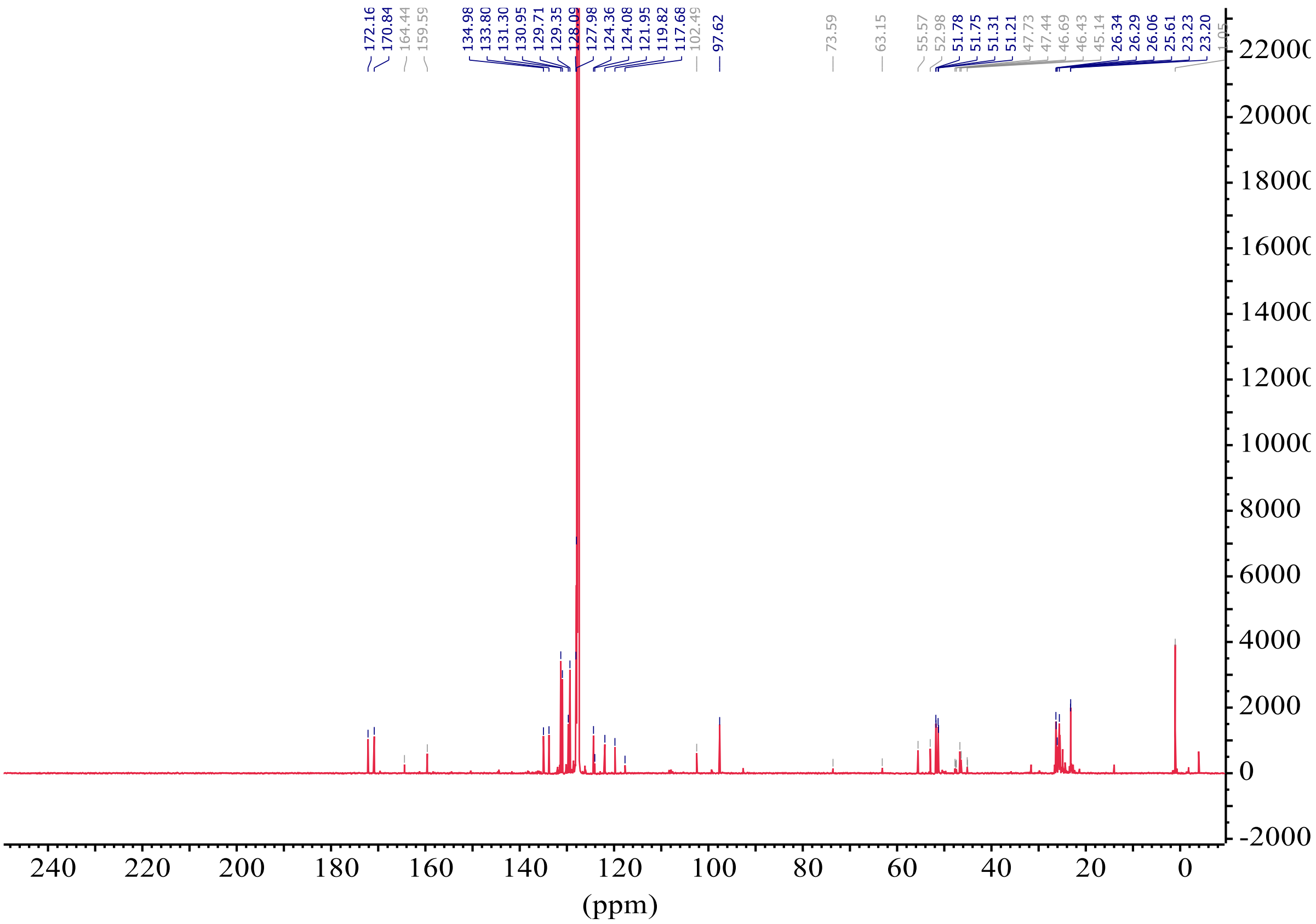

Figure S145: $^{13}C\{^{1}H\}$ NMR (150 MHz) spectrum of **5a** in C_6D_6.

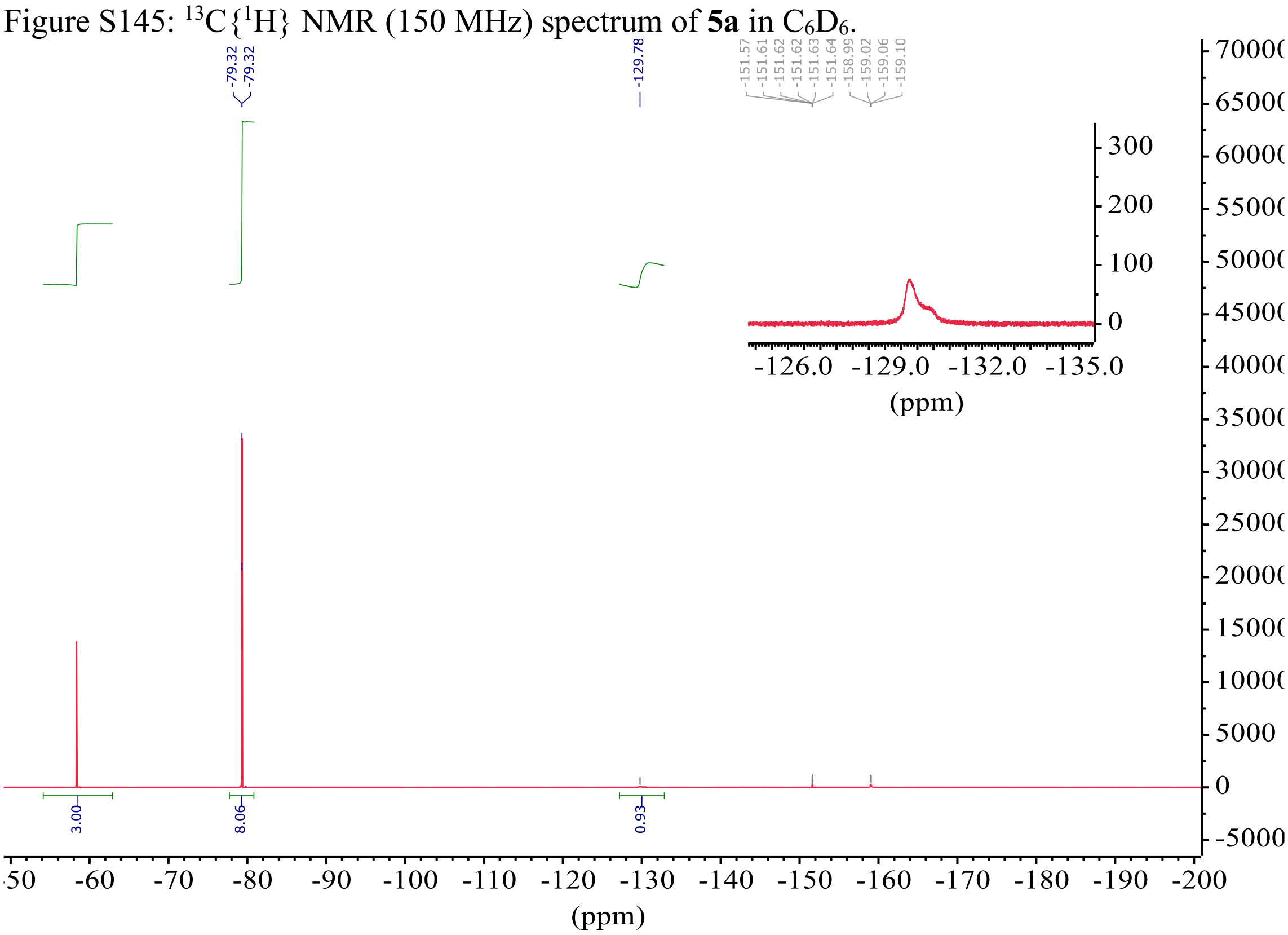

Figure S146: ^{19}F NMR (470 MHz) spectrum of **5a** in CH_3CN.

5b

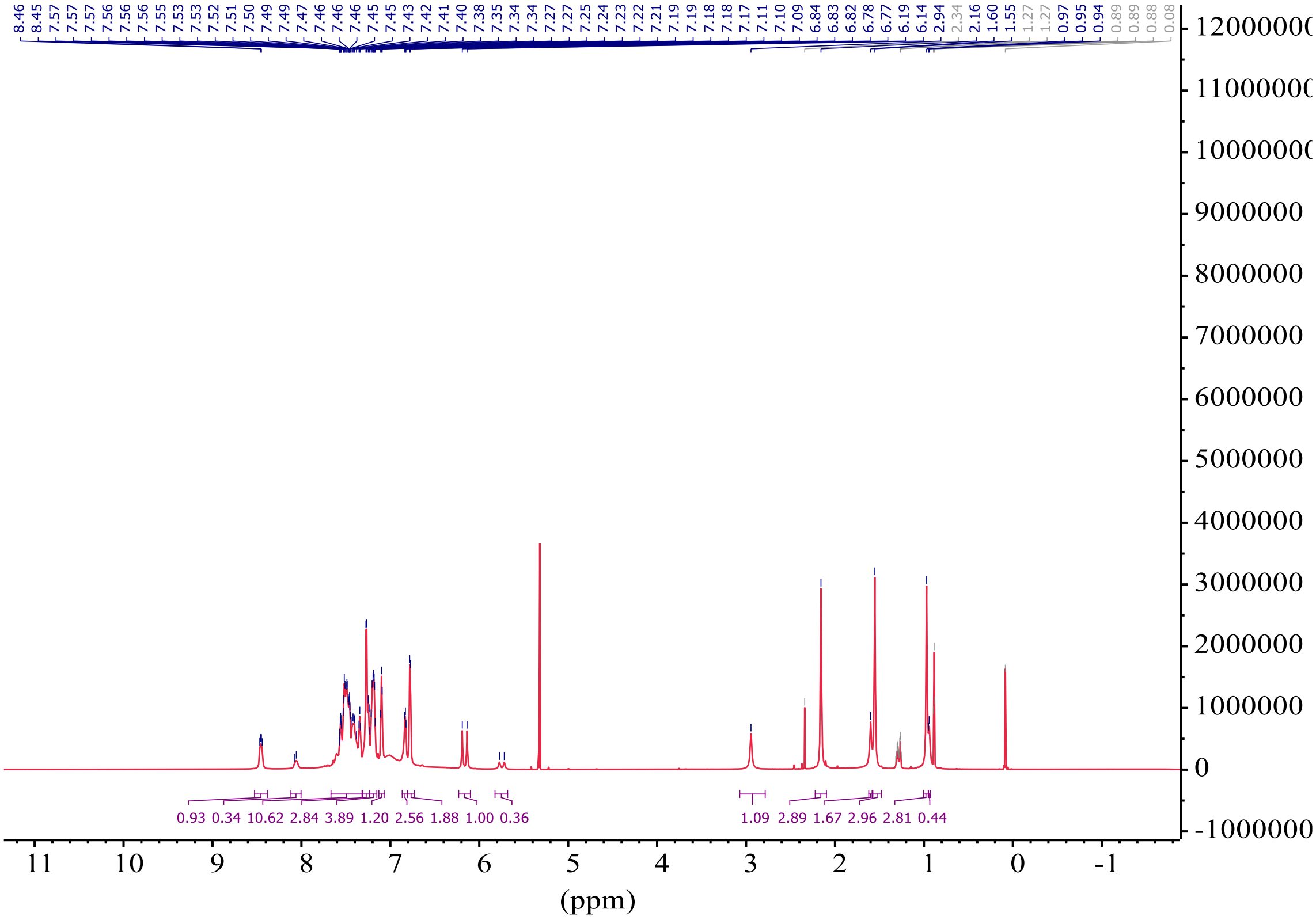

Figure S147: ^{1}H NMR (900 MHz) spectrum of **5b** in CD_2Cl_2. Impurities are marked with grey ticks.

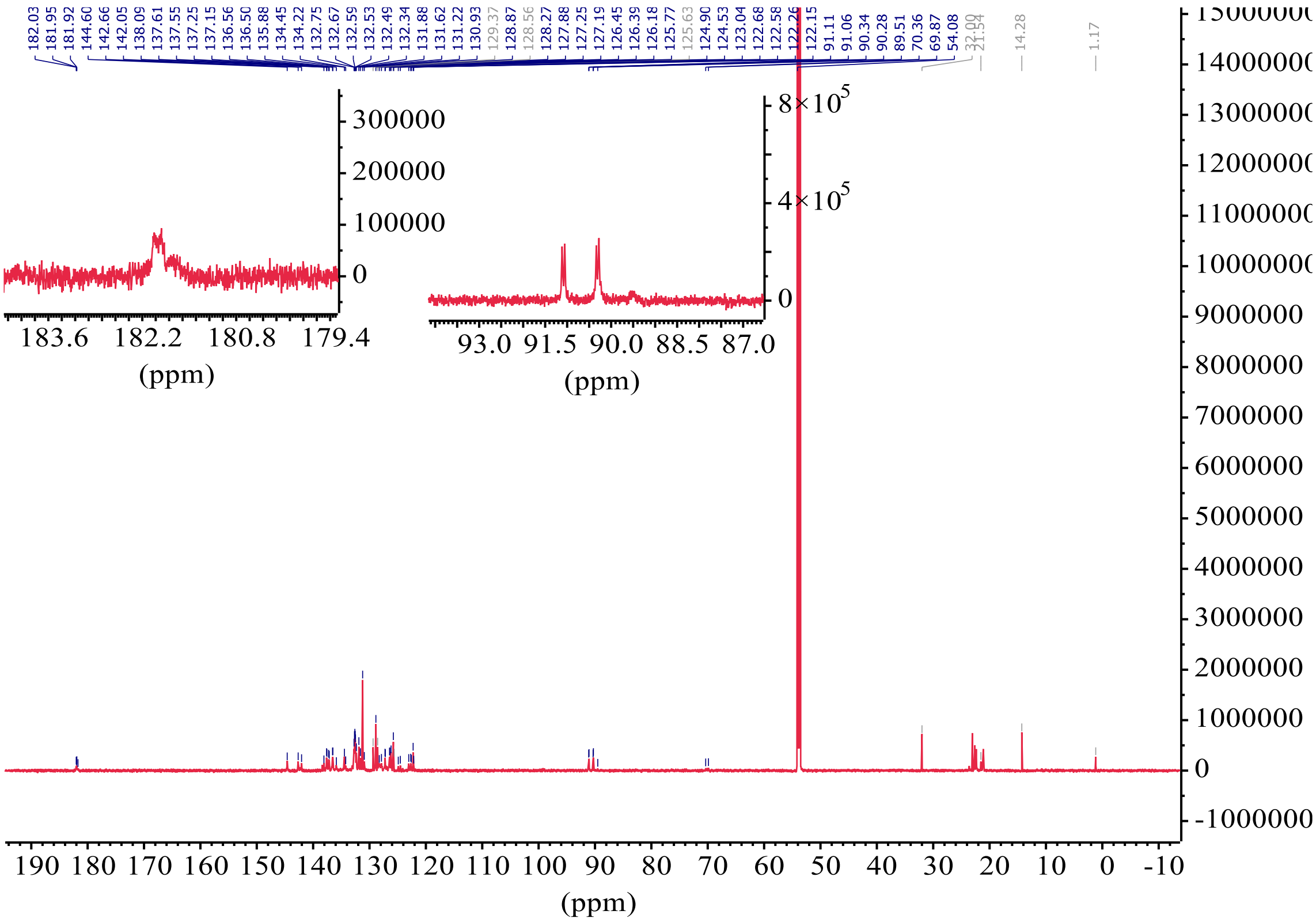

Figure S148: $^{13}C\{^{1}H\}$ NMR (225 MHz) spectrum of **5b** in CD_2Cl_2. Impurities are marked with grey ticks.

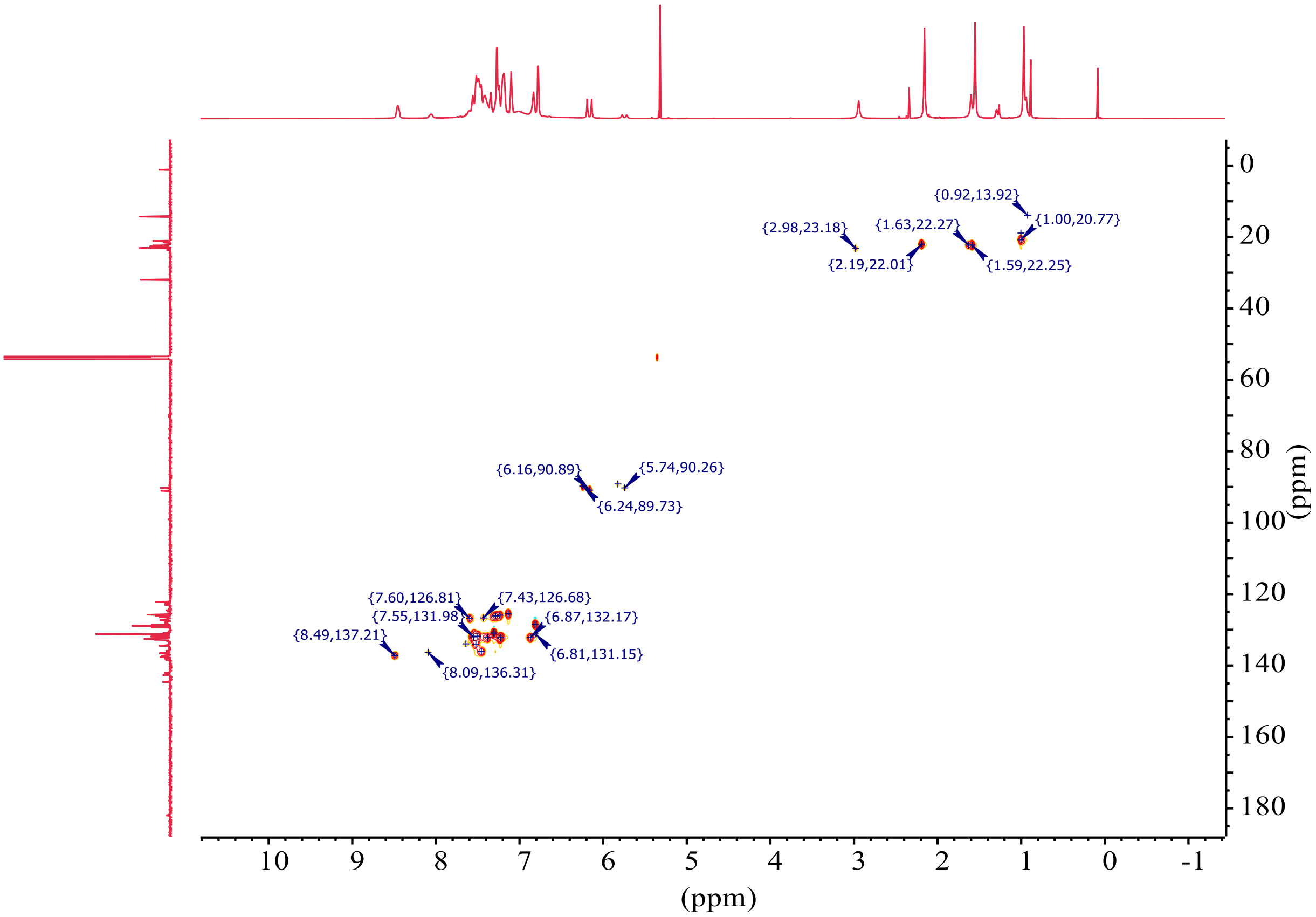

Figure S149: ^{1}H-^{13}C HSQC NMR (600 MHz-150 MHz) spectrum of **5b** in CD_2Cl_2.

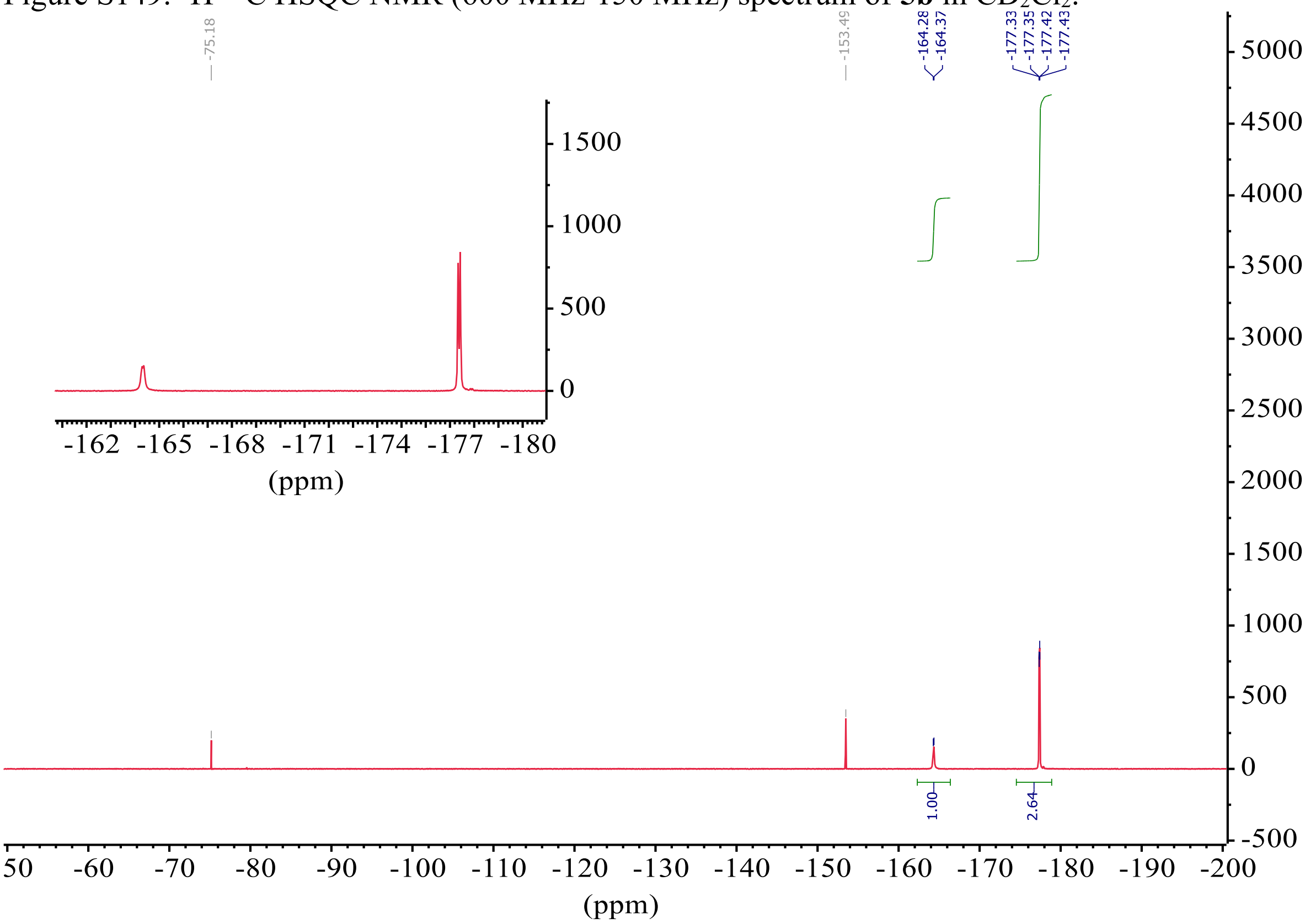

Figure S150: ^{19}F NMR (565 MHz) spectrum of **5b** in CD_2Cl_2.

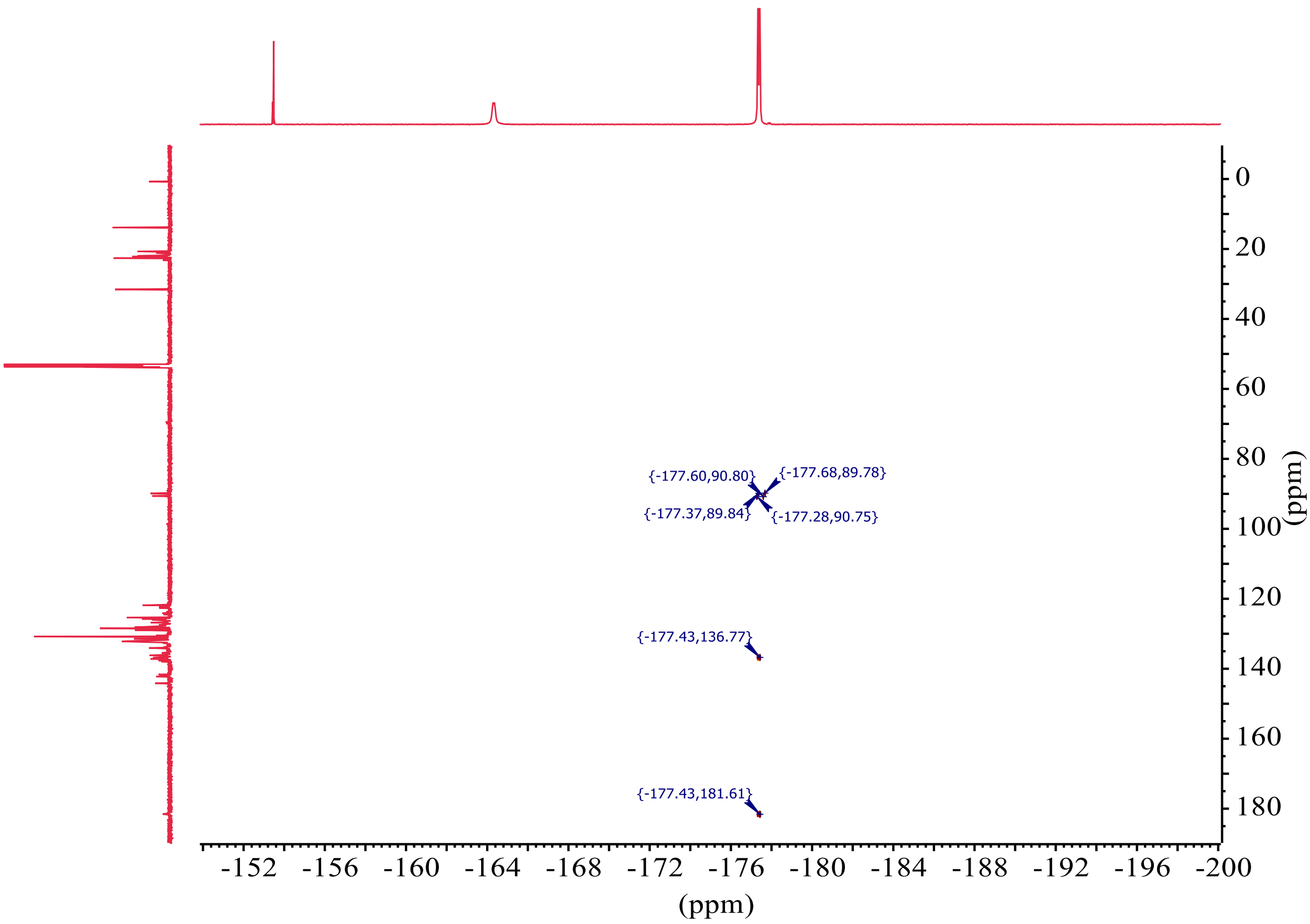

Figure S151: ^{19}F-^{13}C HMBC NMR (565 MHz-150 MHz) spectrum of **5b** in CD_2Cl_2.

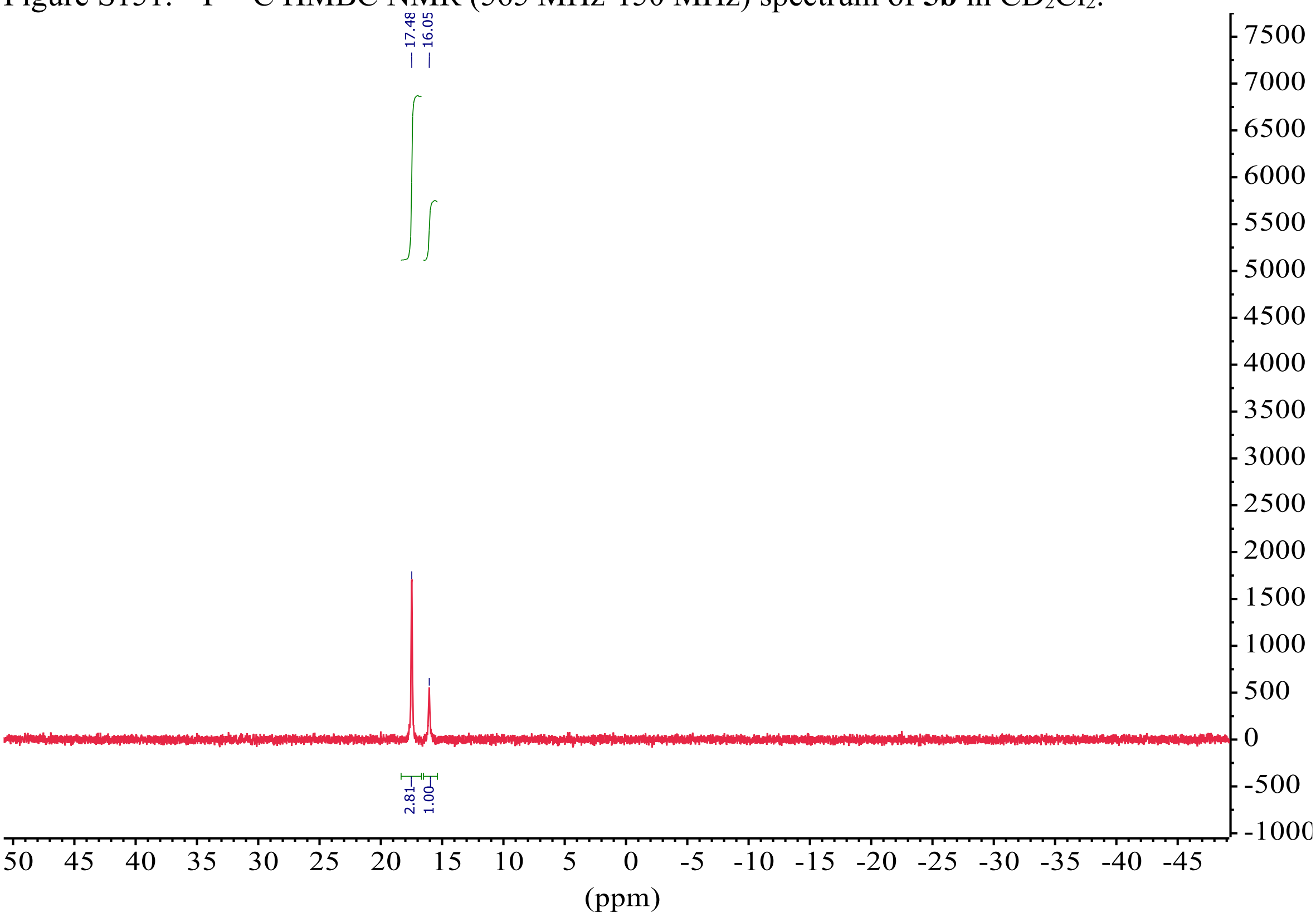

Figure S152: ^{31}P NMR (202 MHz) spectrum of **5b** in CD_2Cl_2.

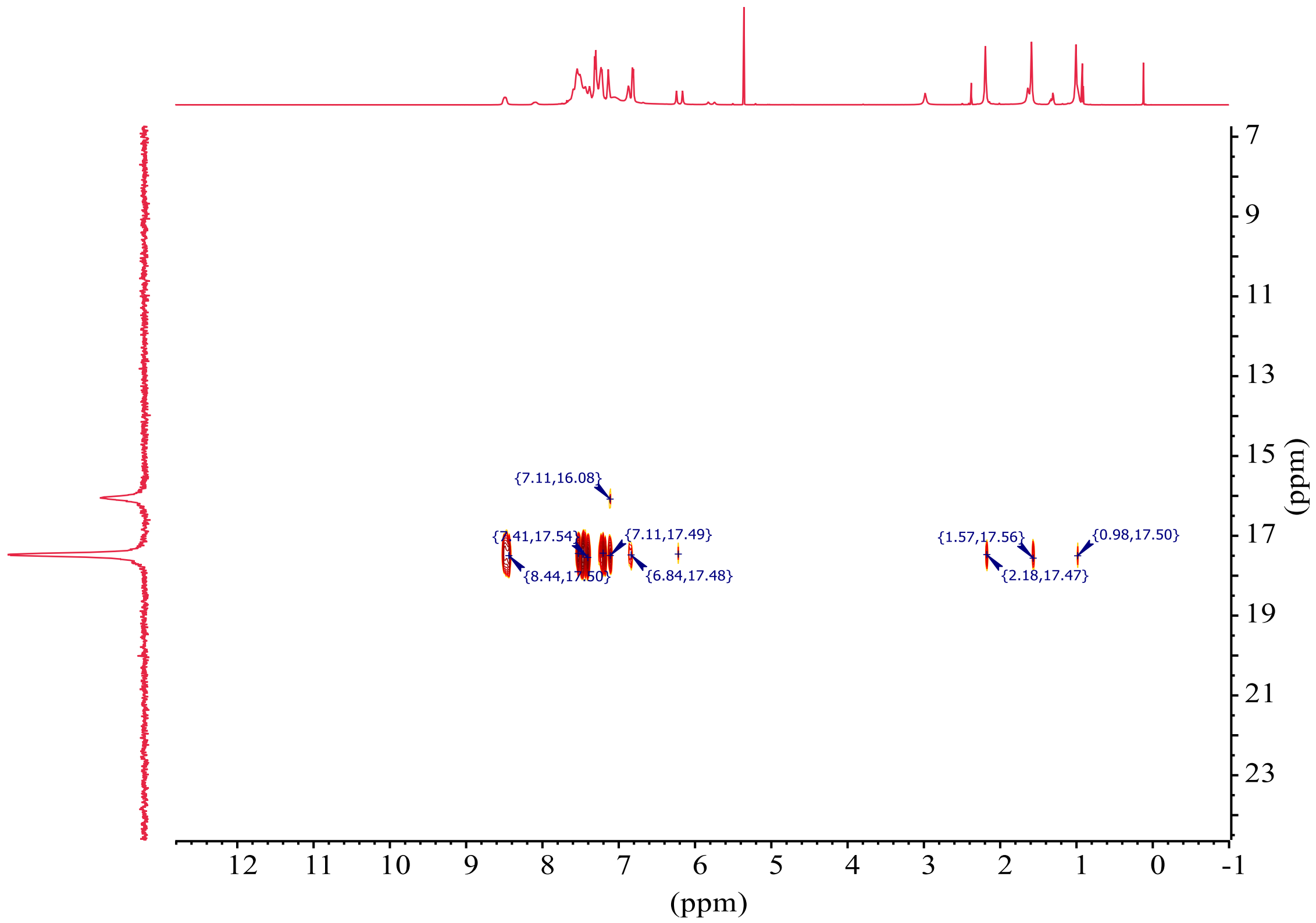

Figure S153: ^{1}H-^{31}P HMBC NMR (500 MHz-202 MHz) spectrum of **5b** in CD_2Cl_2.

5c

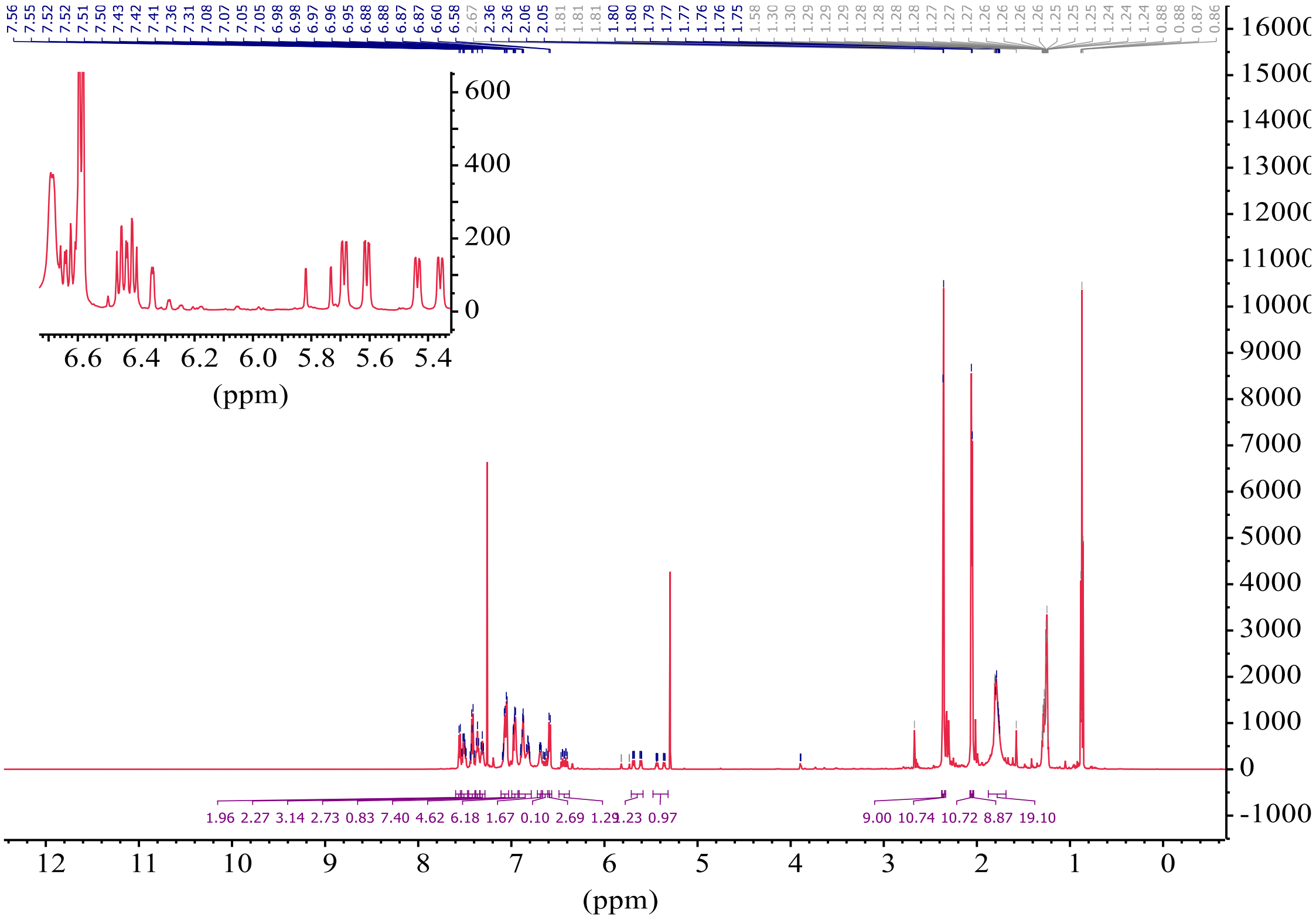

Figure S154: ^{1}H NMR (600 MHz) spectrum of **5c** in $CDCl_3$. Impurities are marked with grey ticks.

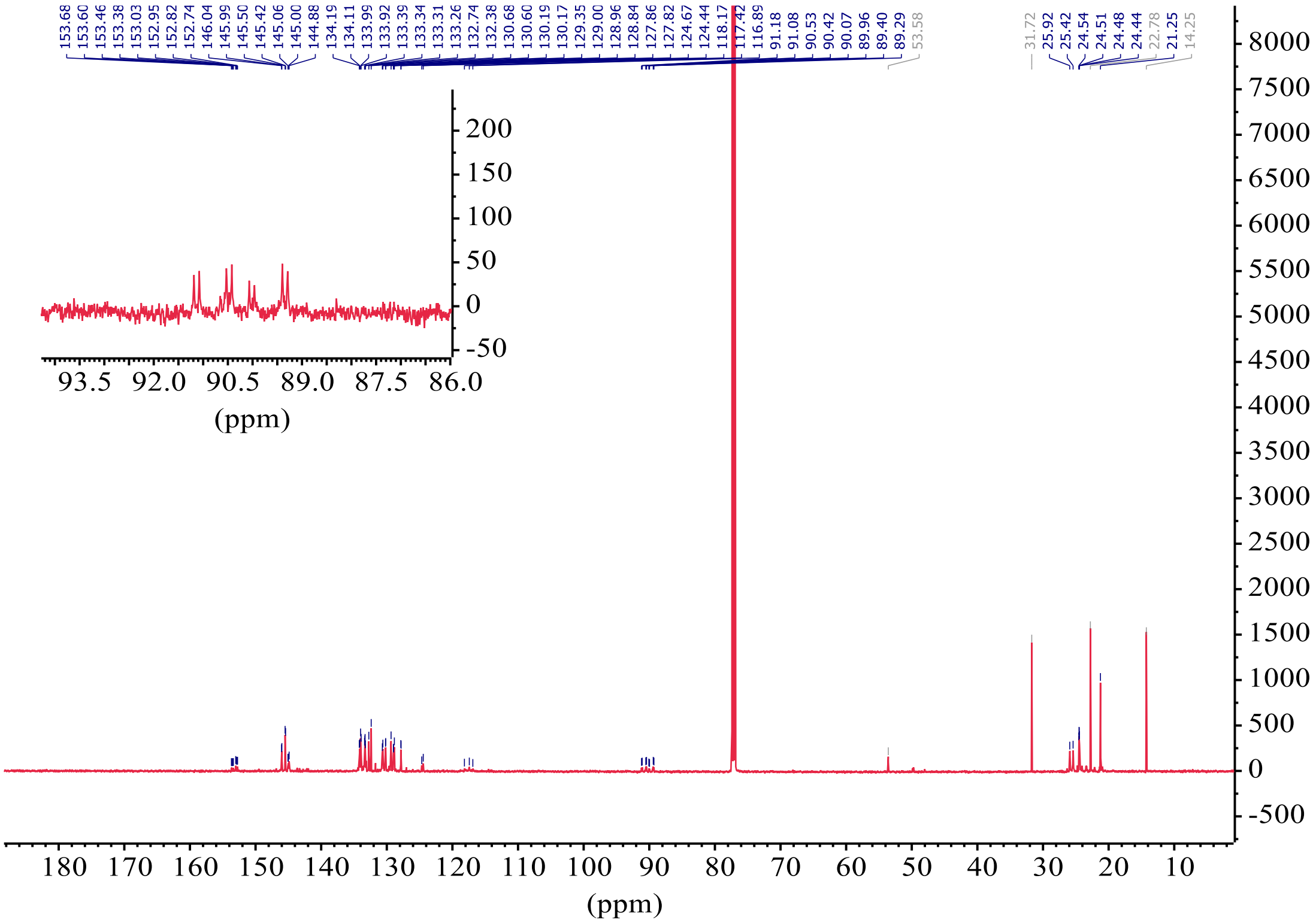

Figure S155: $^{13}C\{^{1}H\}$ NMR (150 MHz) spectrum of **5c** in $CDCl_3$. Impurities are marked with grey ticks.

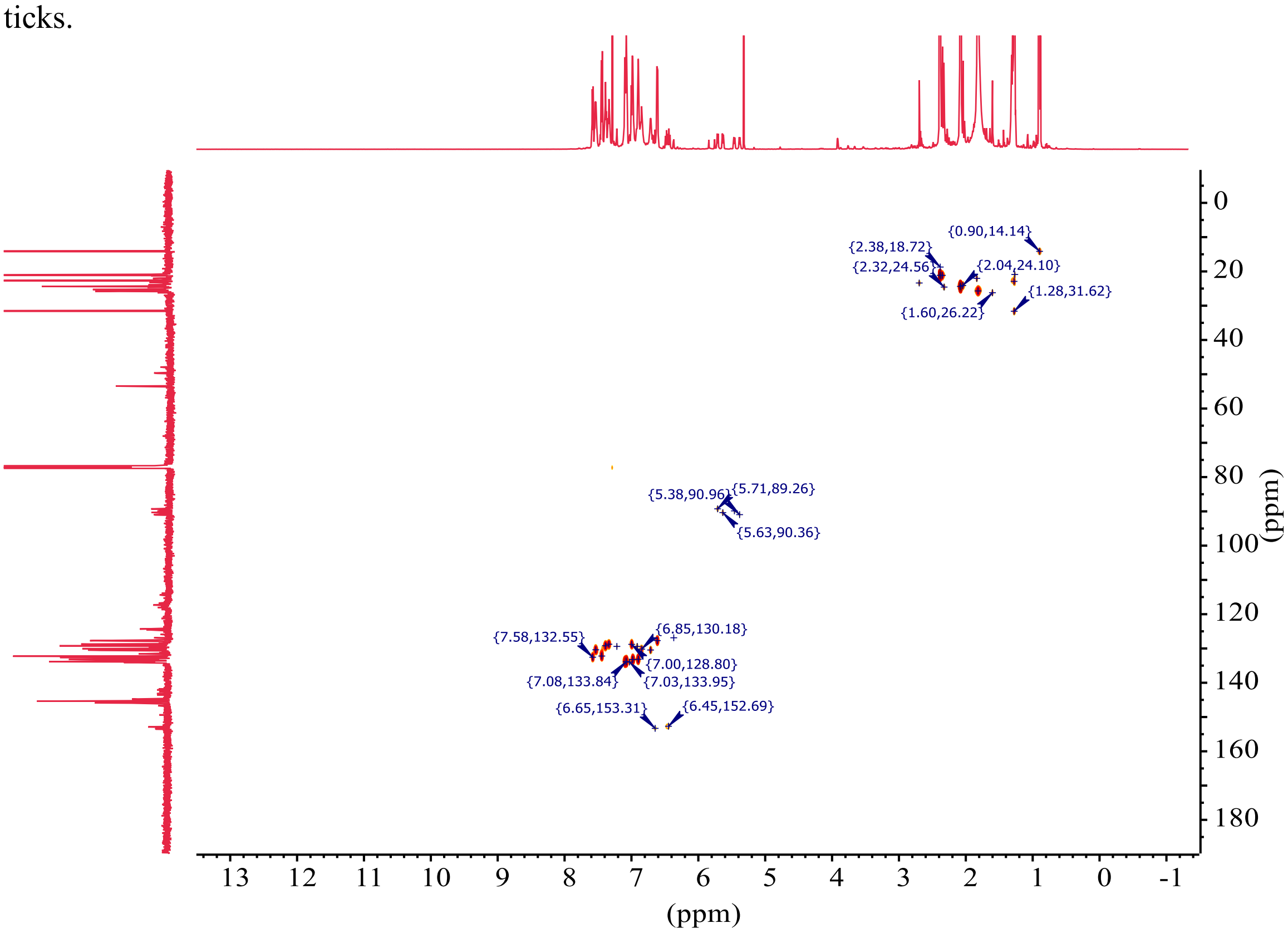

Figure S156: ^{1}H-^{13}C HSQC NMR (600 MHz-150 MHz) spectrum of **5c** in $CDCl_3$.

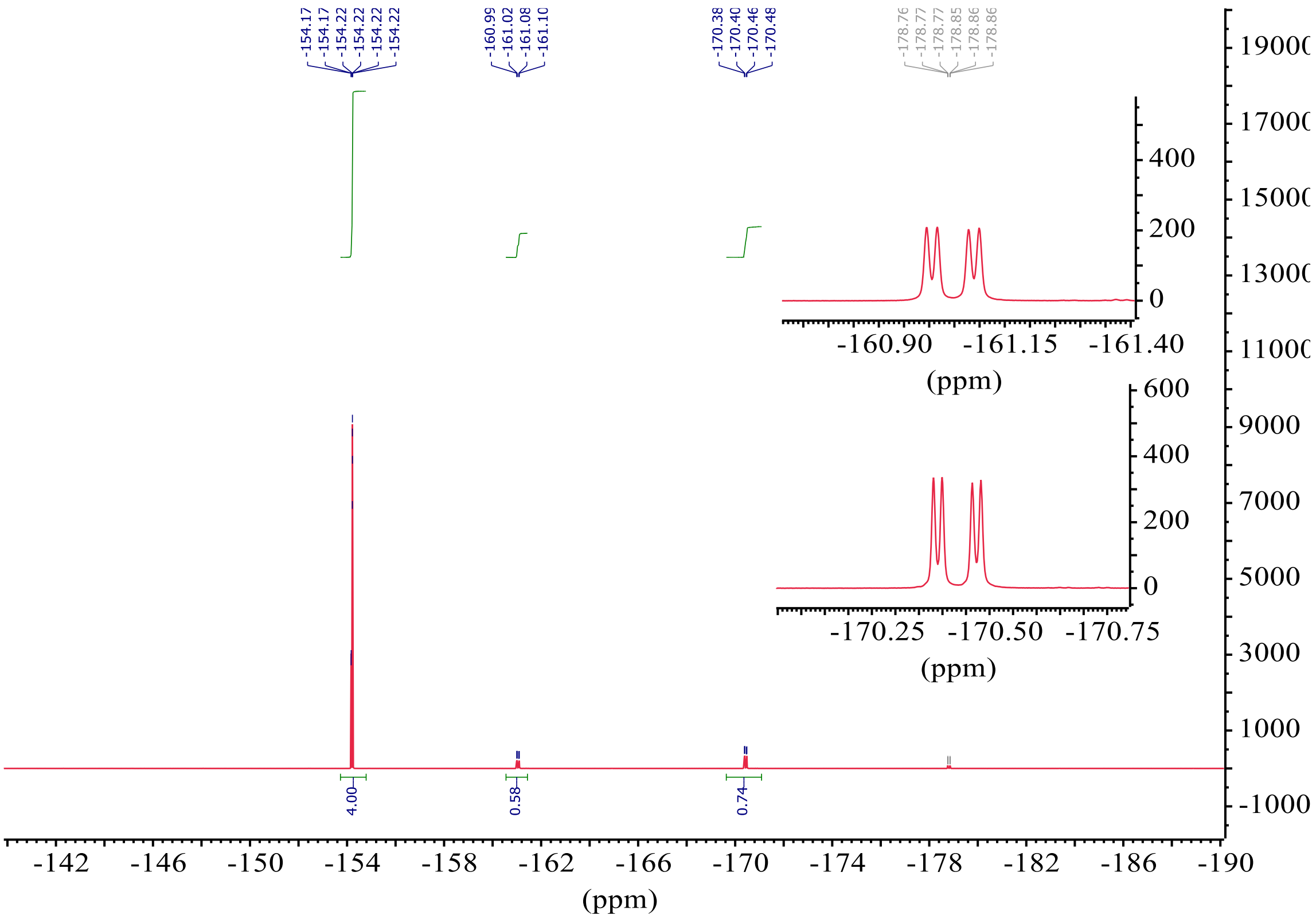

Figure S157: ^{19}F NMR (565 MHz) spectrum of **5c** in $CDCl_3$.

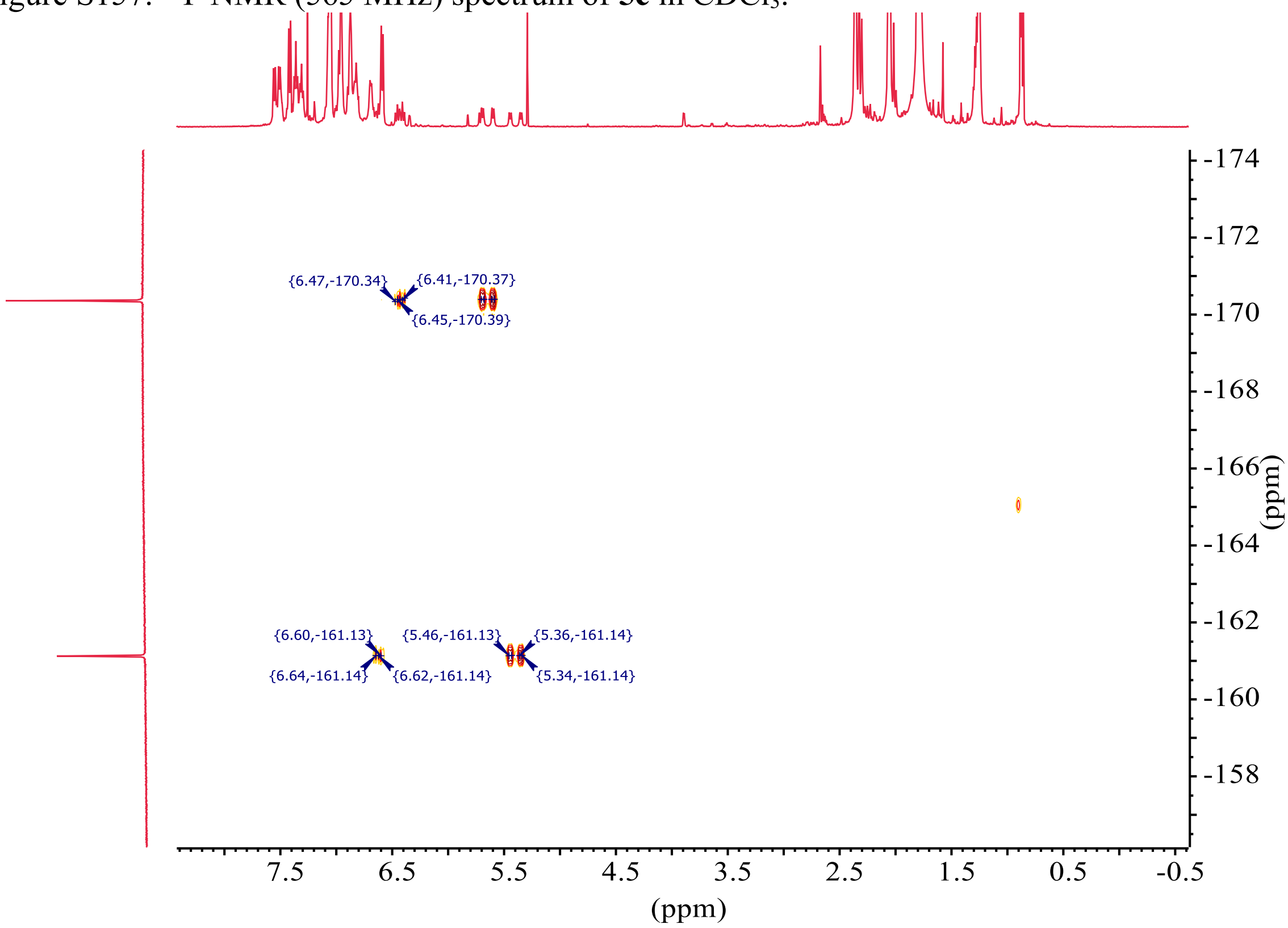

Figure S158: ^{1}H-^{19}F COSY NMR (500 MHz-470 MHz) spectrum of **5c** in $CDCl_3$.

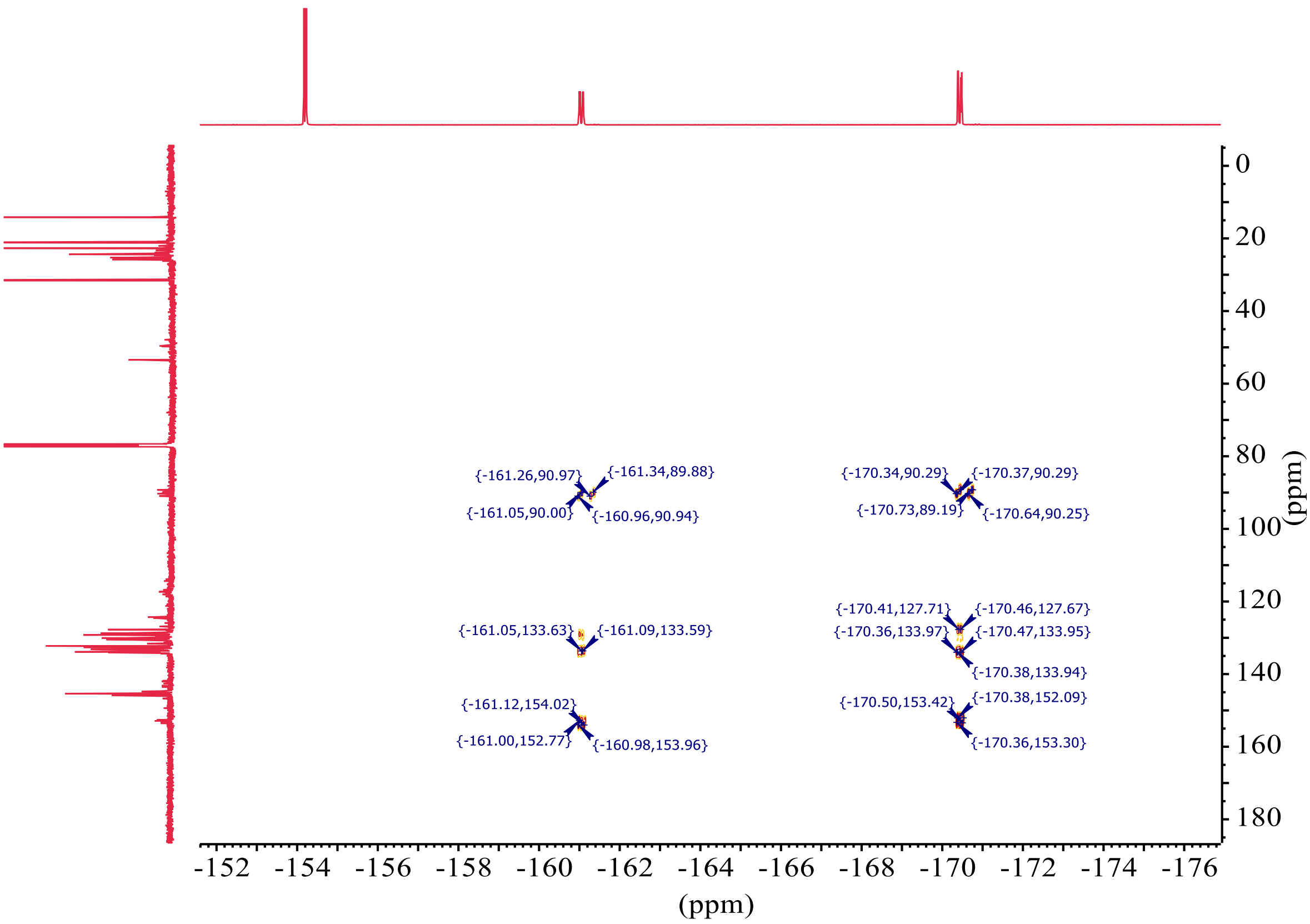

Figure S159: ^{19}F-^{13}C HMBC NMR (565 MHz-150 MHz) spectrum of **5c** in $CDCl_3$.

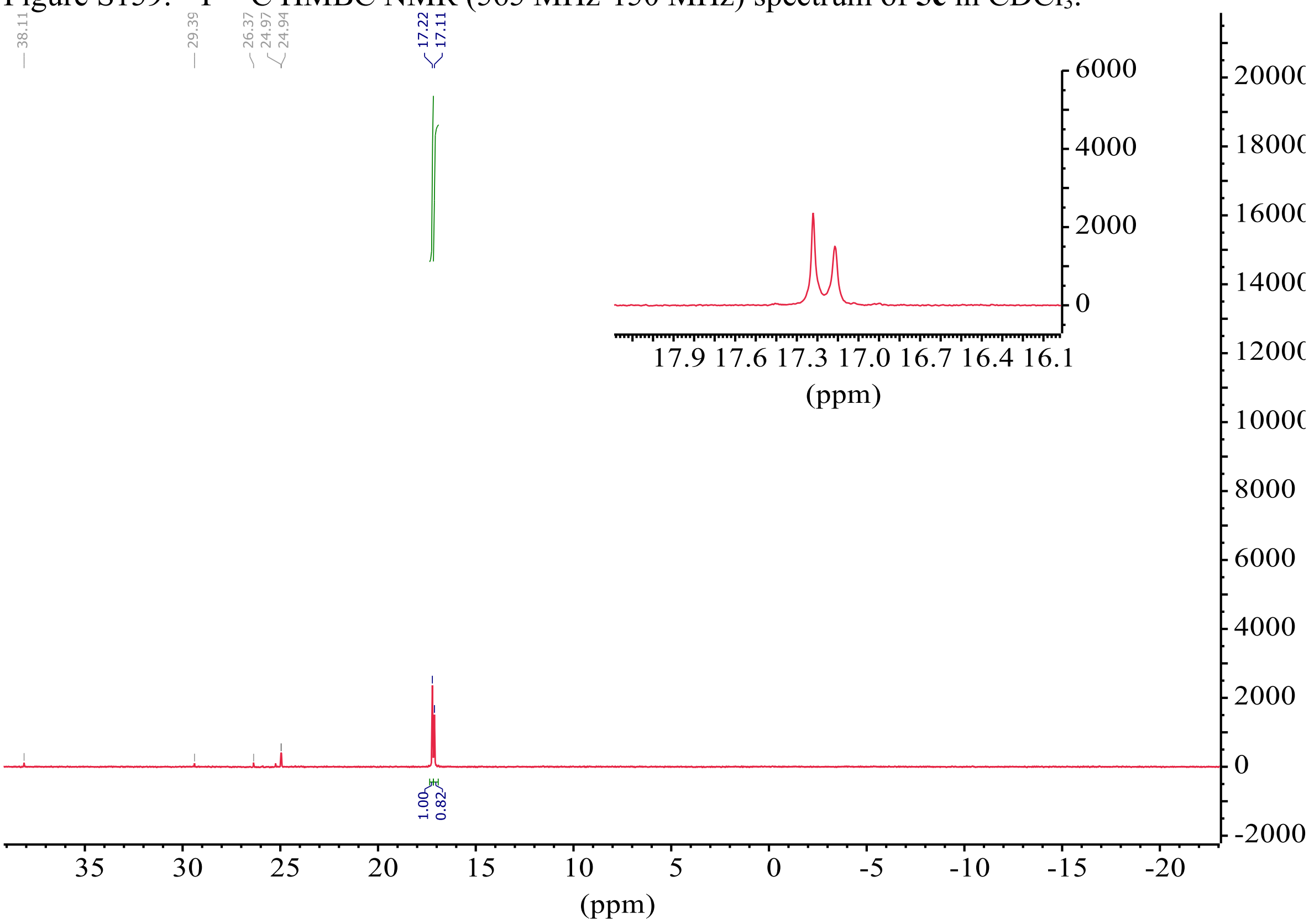

Figure S160: ^{31}P NMR (202 MHz) spectrum of **5c** in $CDCl_3$.

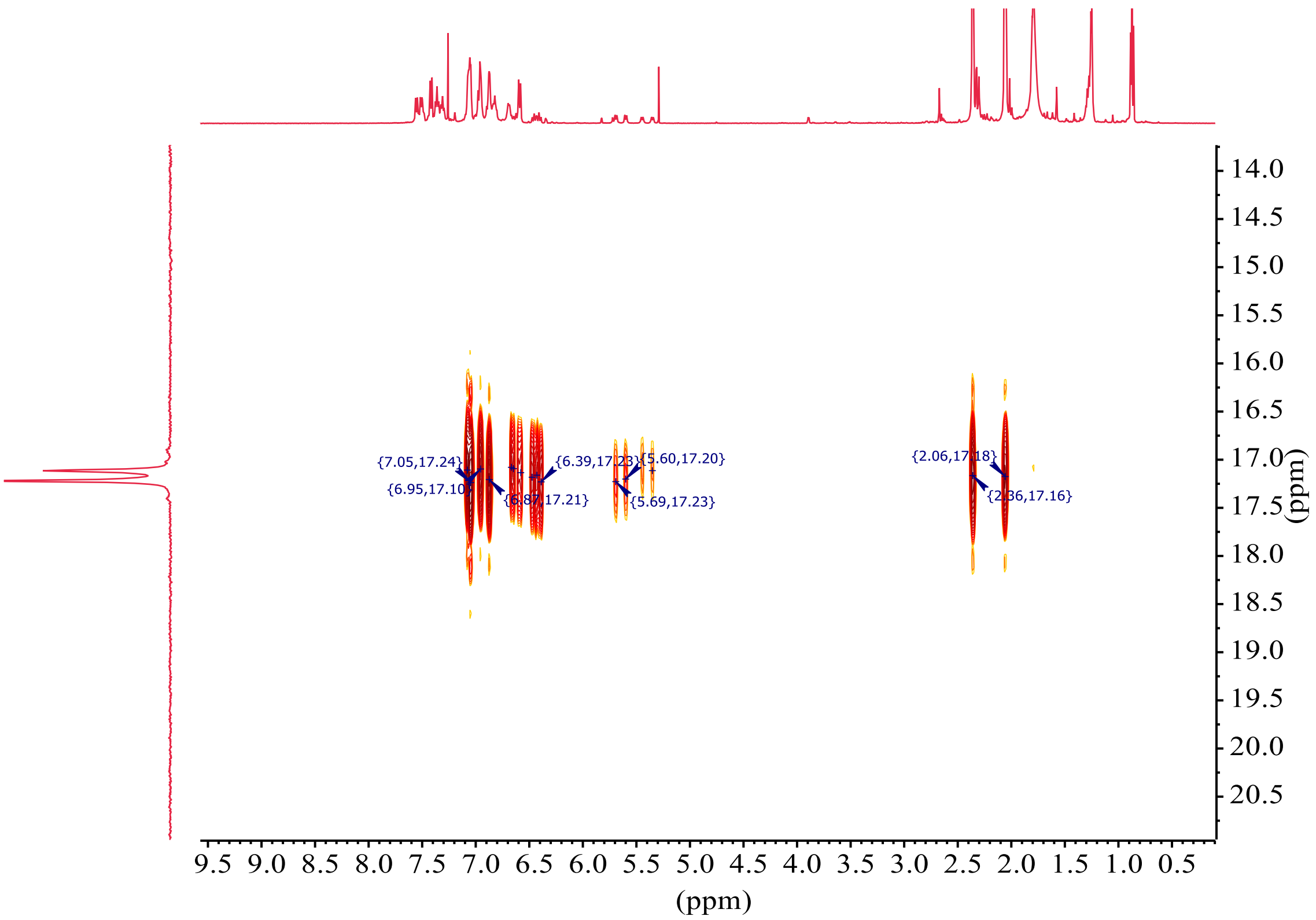

Figure S161: ^{1}H-^{31}P HMBC NMR (500 MHz-202 MHz) spectrum of **5c** in $CDCl_3$.

5d

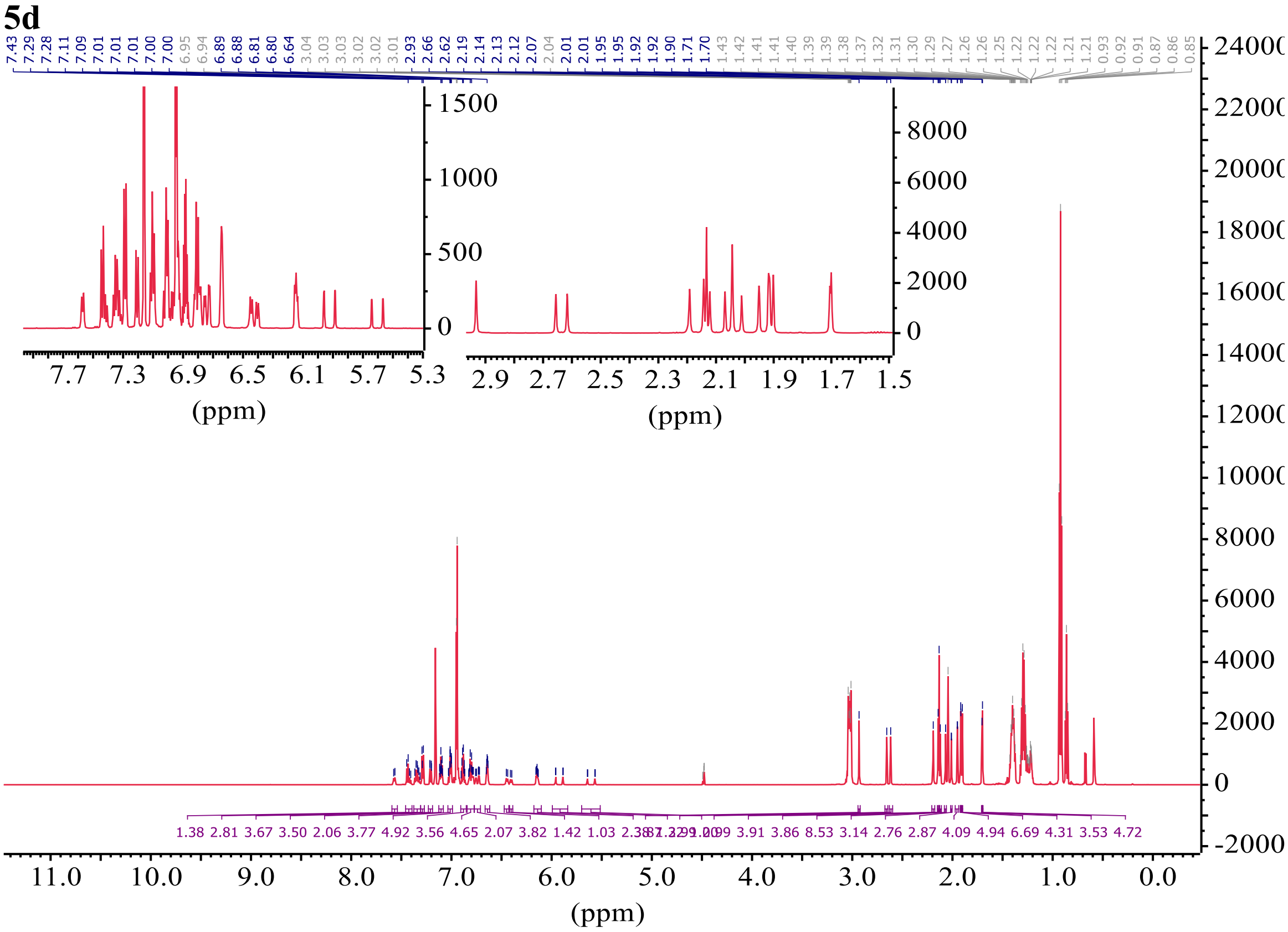

Figure S162: ^{1}H NMR (600 MHz) spectrum of **5d** in C_6D_6. Impurities are marked with grey ticks.

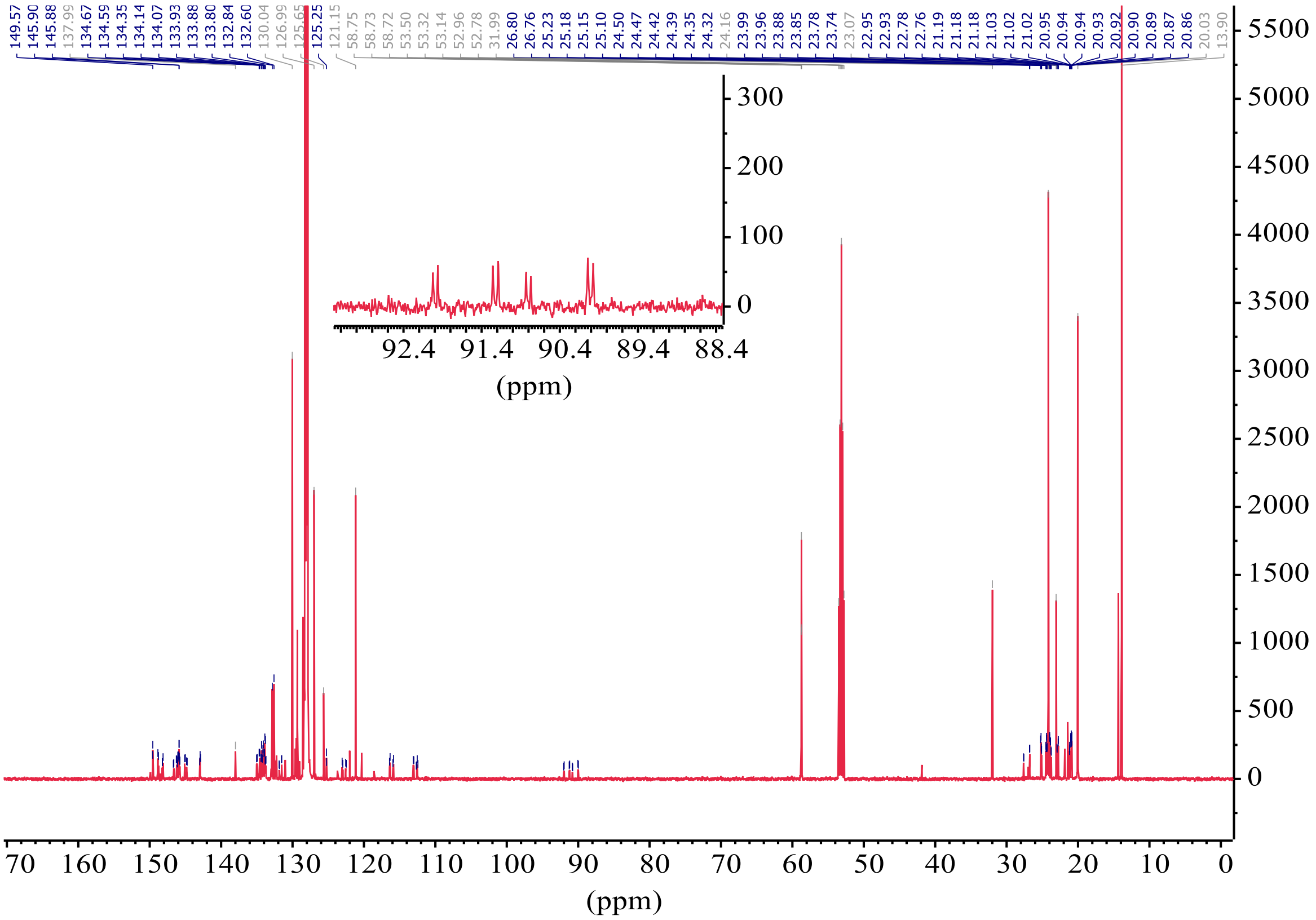

Figure S163: $^{13}C\{^1H\}$ NMR (150 MHz) spectrum of **5d** in C_6D_6. Impurities are marked with grey ticks.

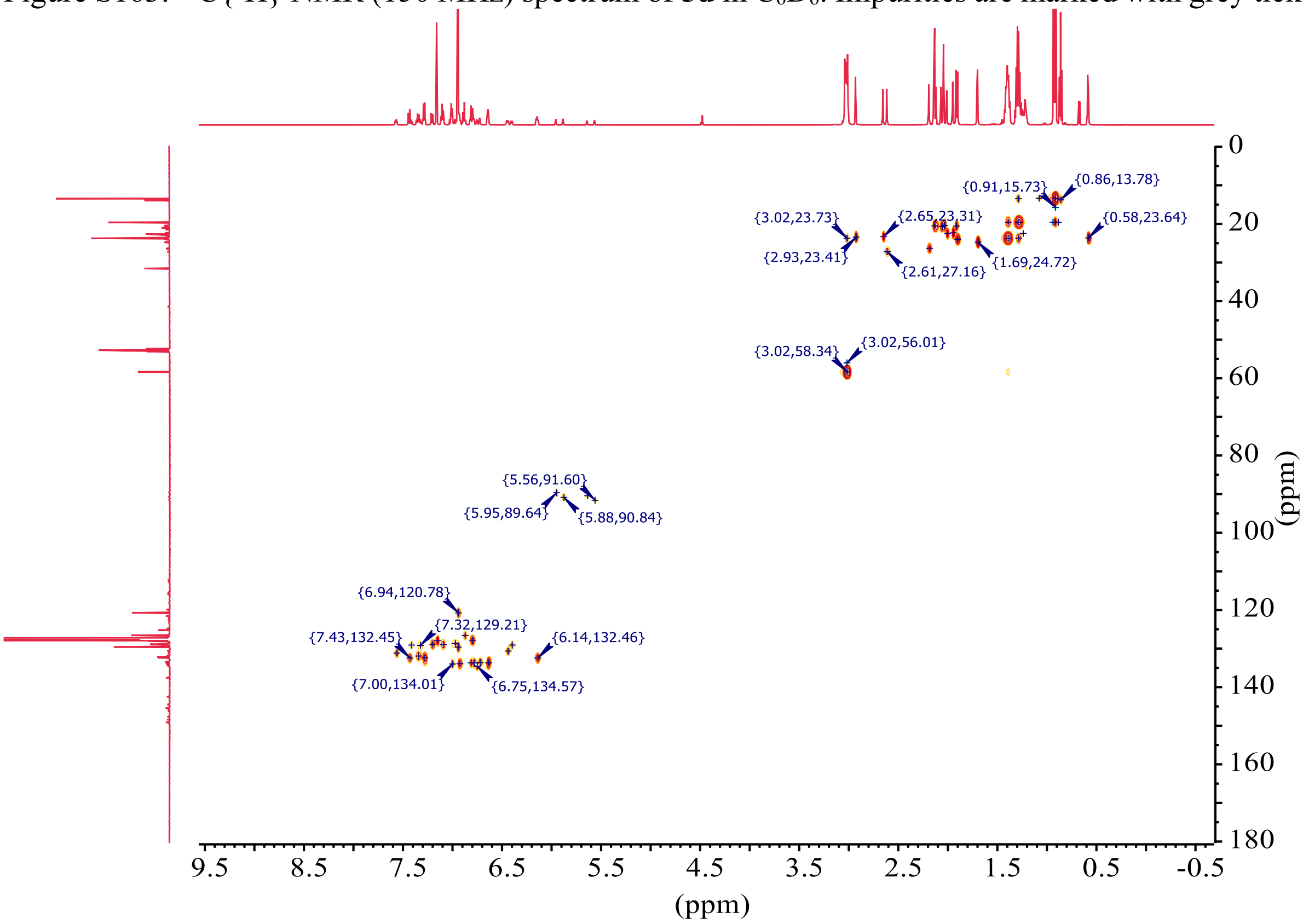

Figure S164: ^1H-^{13}C HSQC NMR (600 MHz-150 MHz) spectrum of **5d** in C_6D_6.

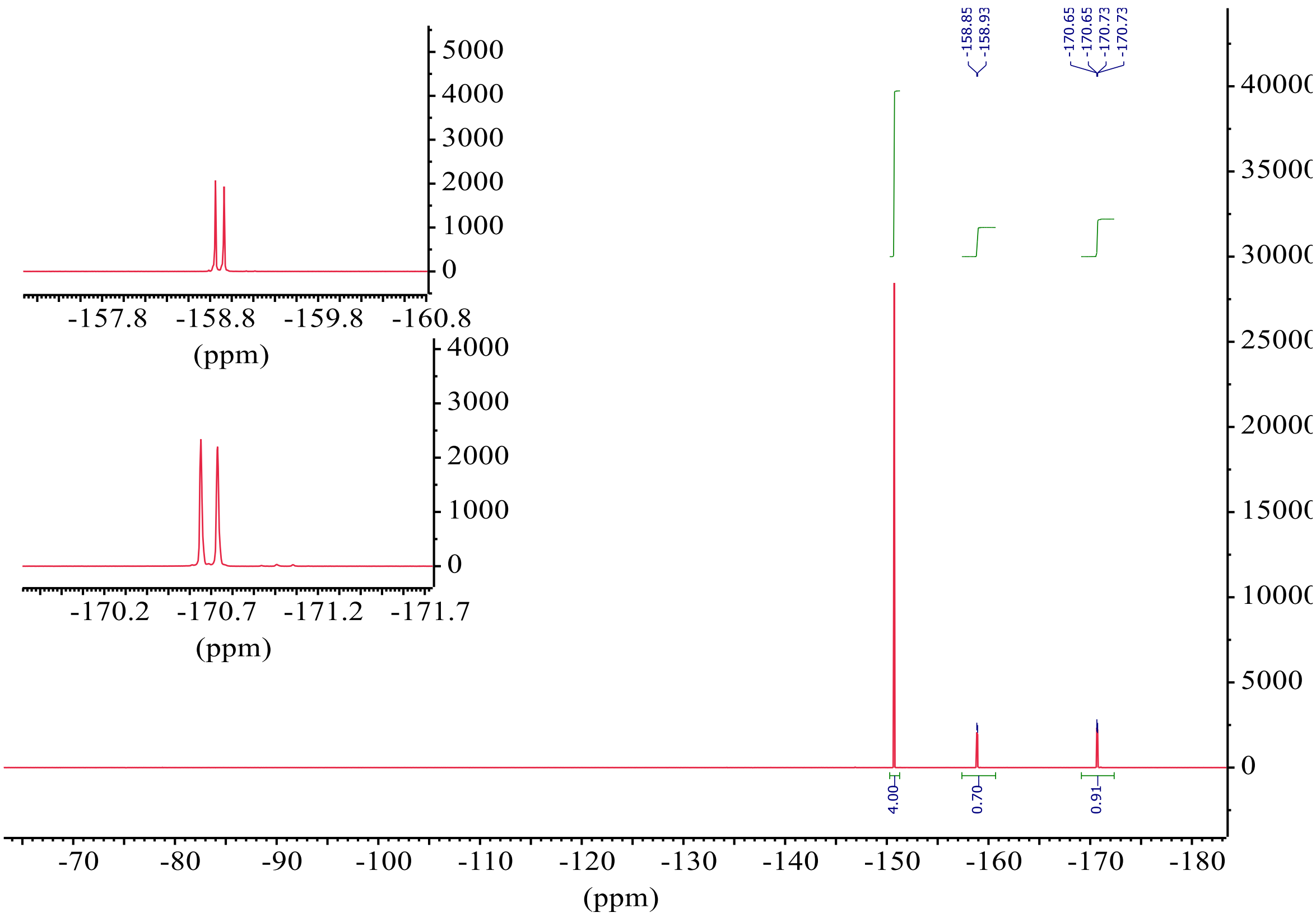

Figure S165: ^{19}F NMR (565 MHz) spectrum of **5d** in C_6D_2.

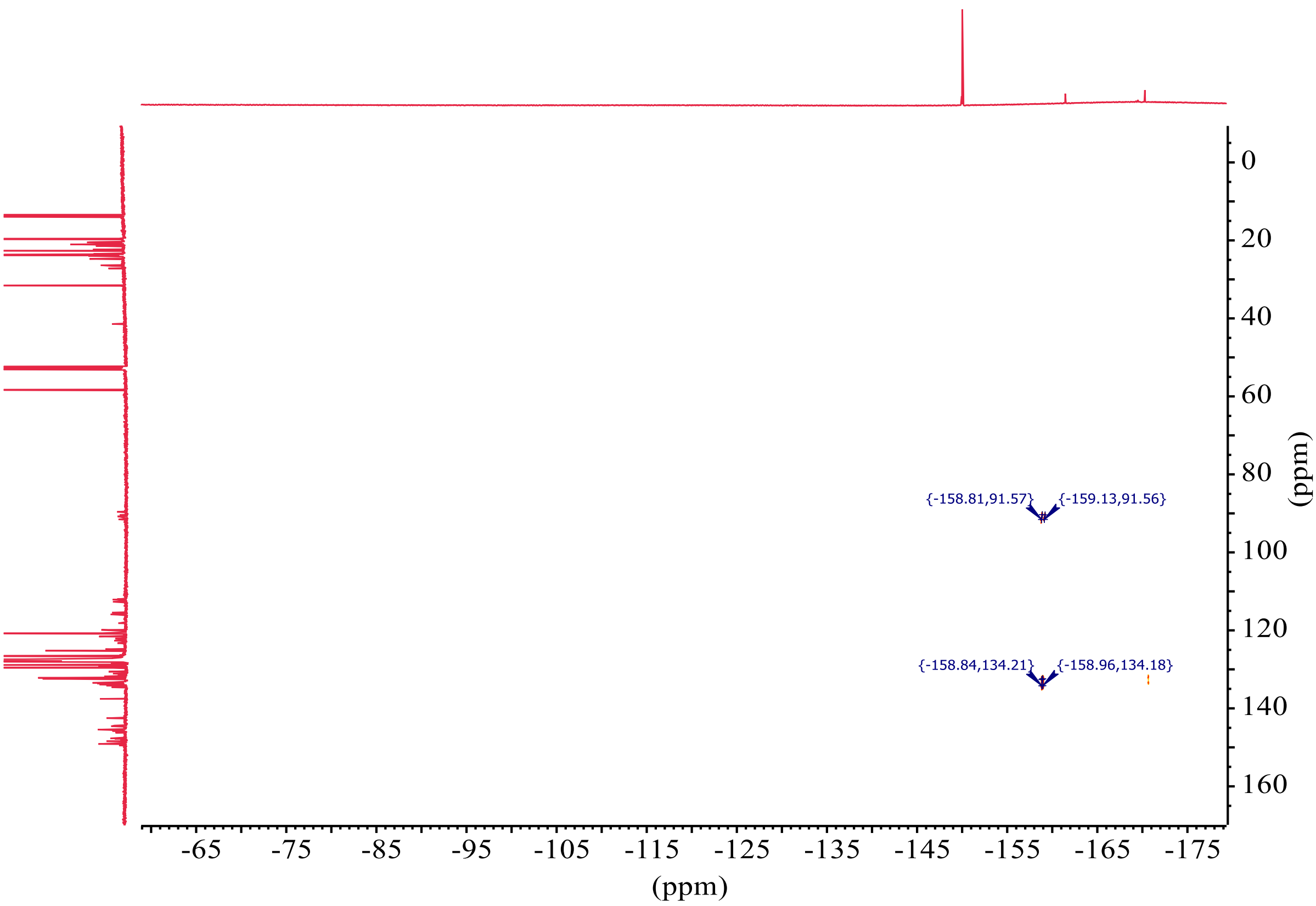

Figure S166: ^{19}F-^{13}C HMBC NMR (565 MHz-150 MHz) spectrum of **5d** in C_6D_2.

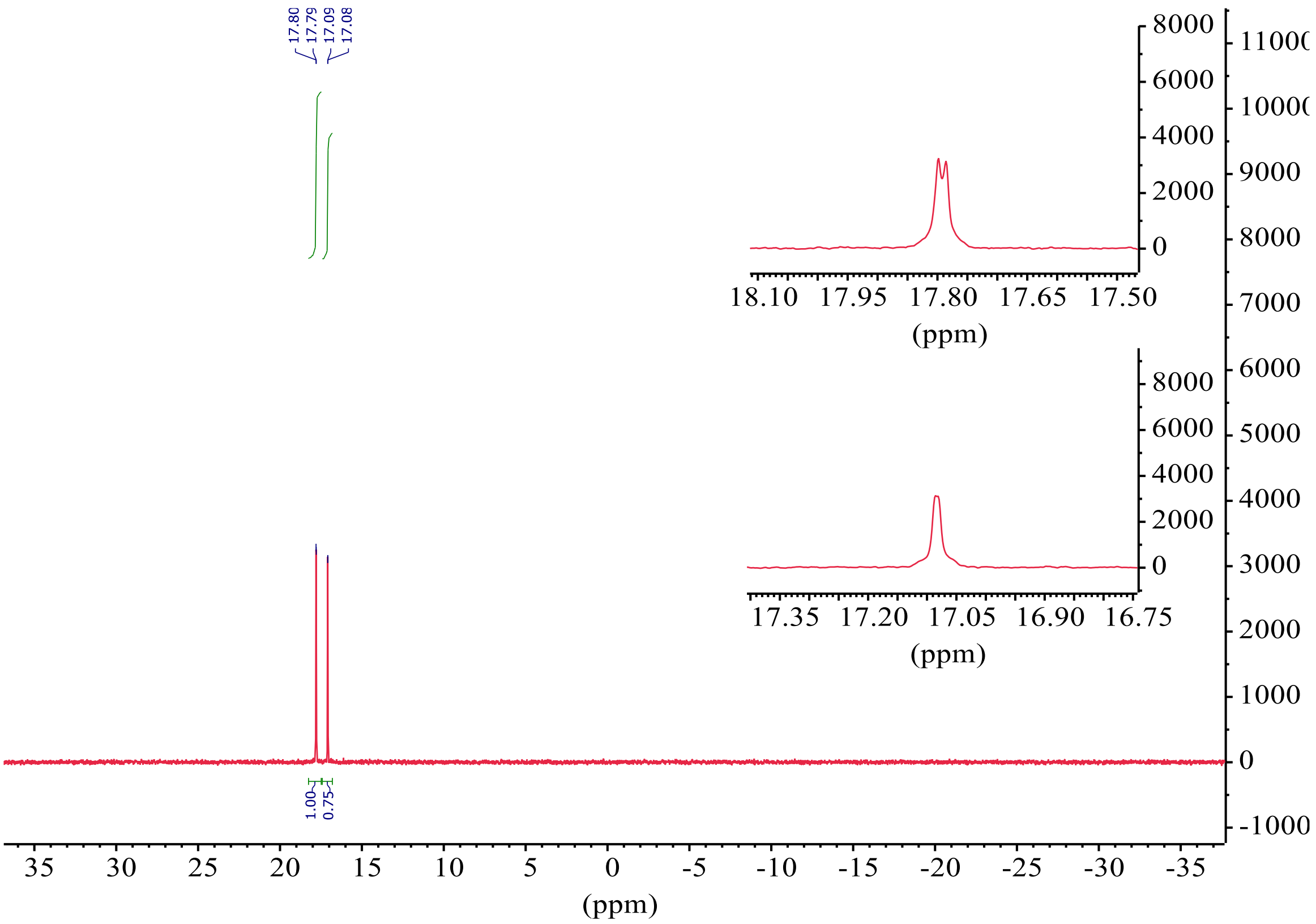

Figure S167: ^{31}P NMR (202 MHz) spectrum of **5d** in C_6D_6.

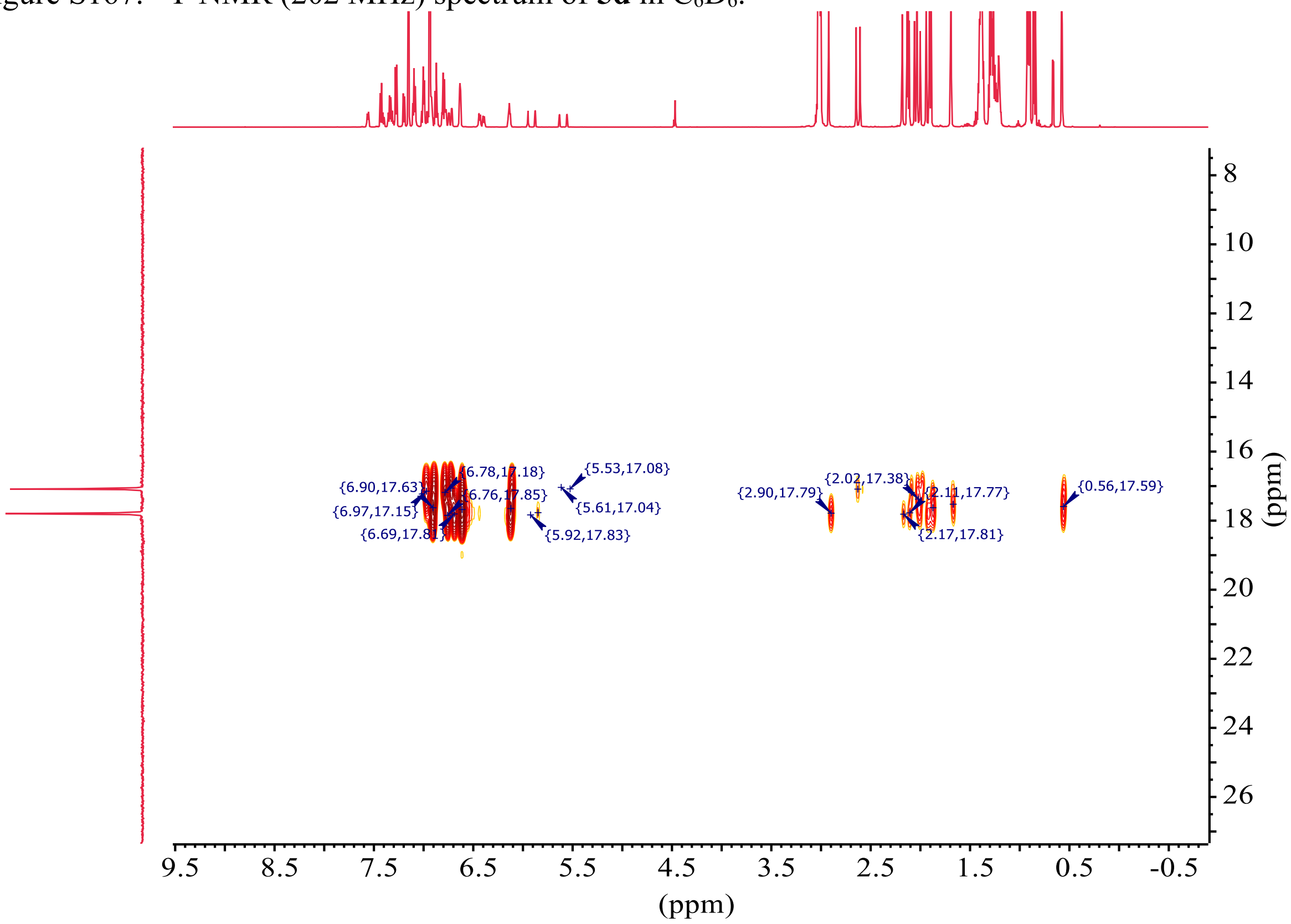

Figure S168: ^{31}P NMR (202 MHz) spectrum of **5d** in C_6D_6.

Mass Spectrometry Data

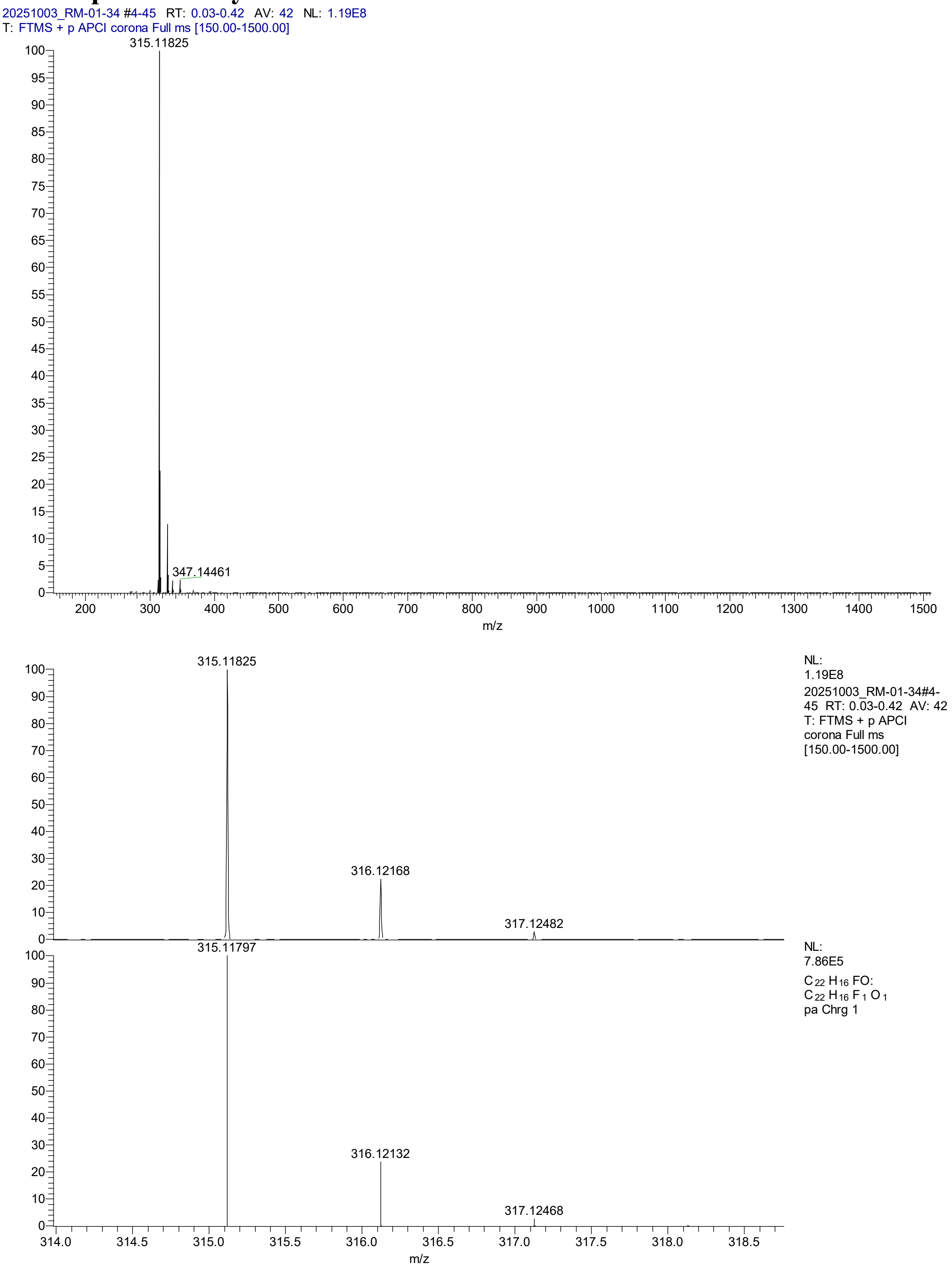

Figure S169: HRMS(APCI+) Spectra for **1d**.

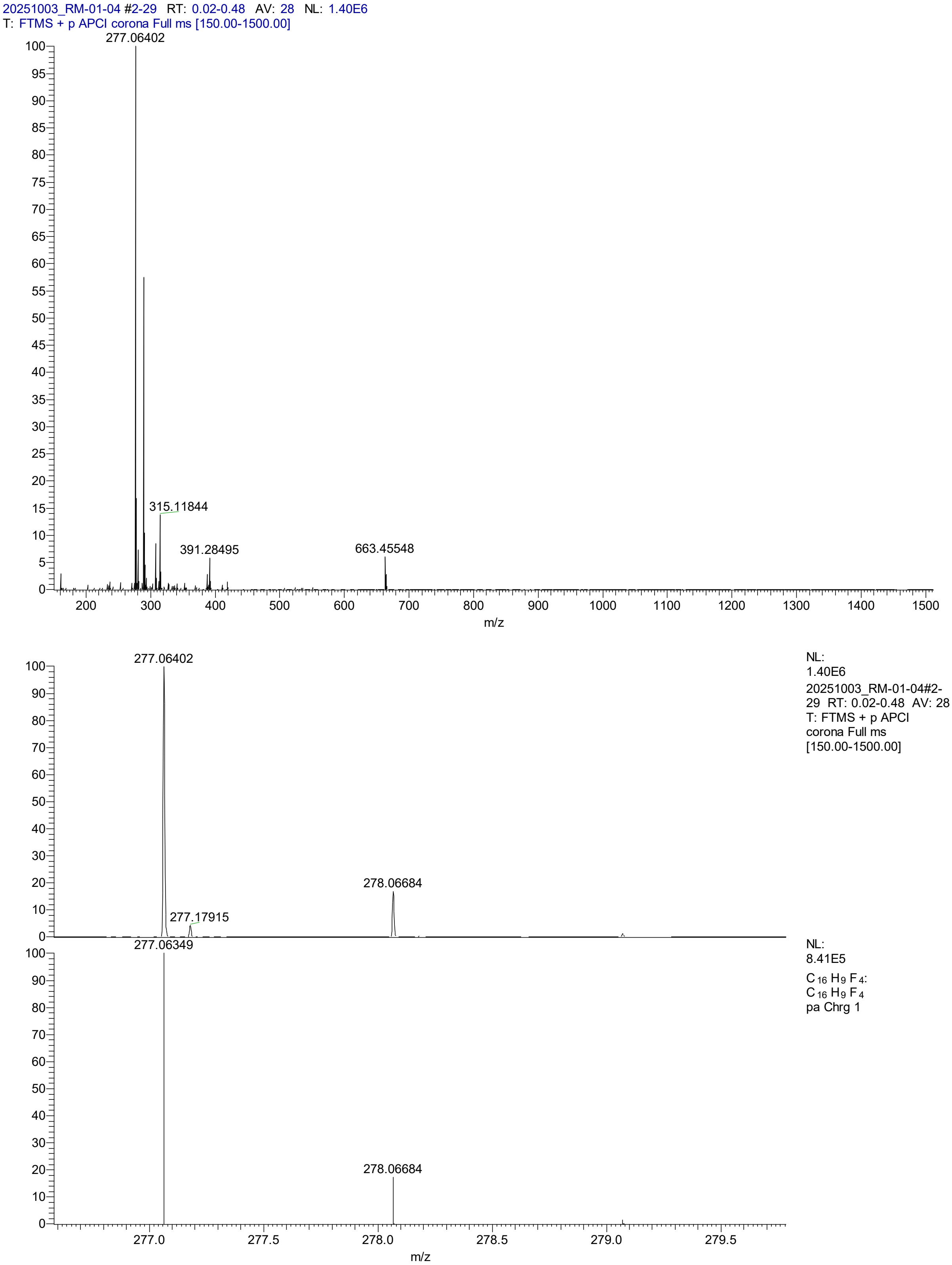

Figure S170: HRMS(APCI+) Spectra for **1f**.

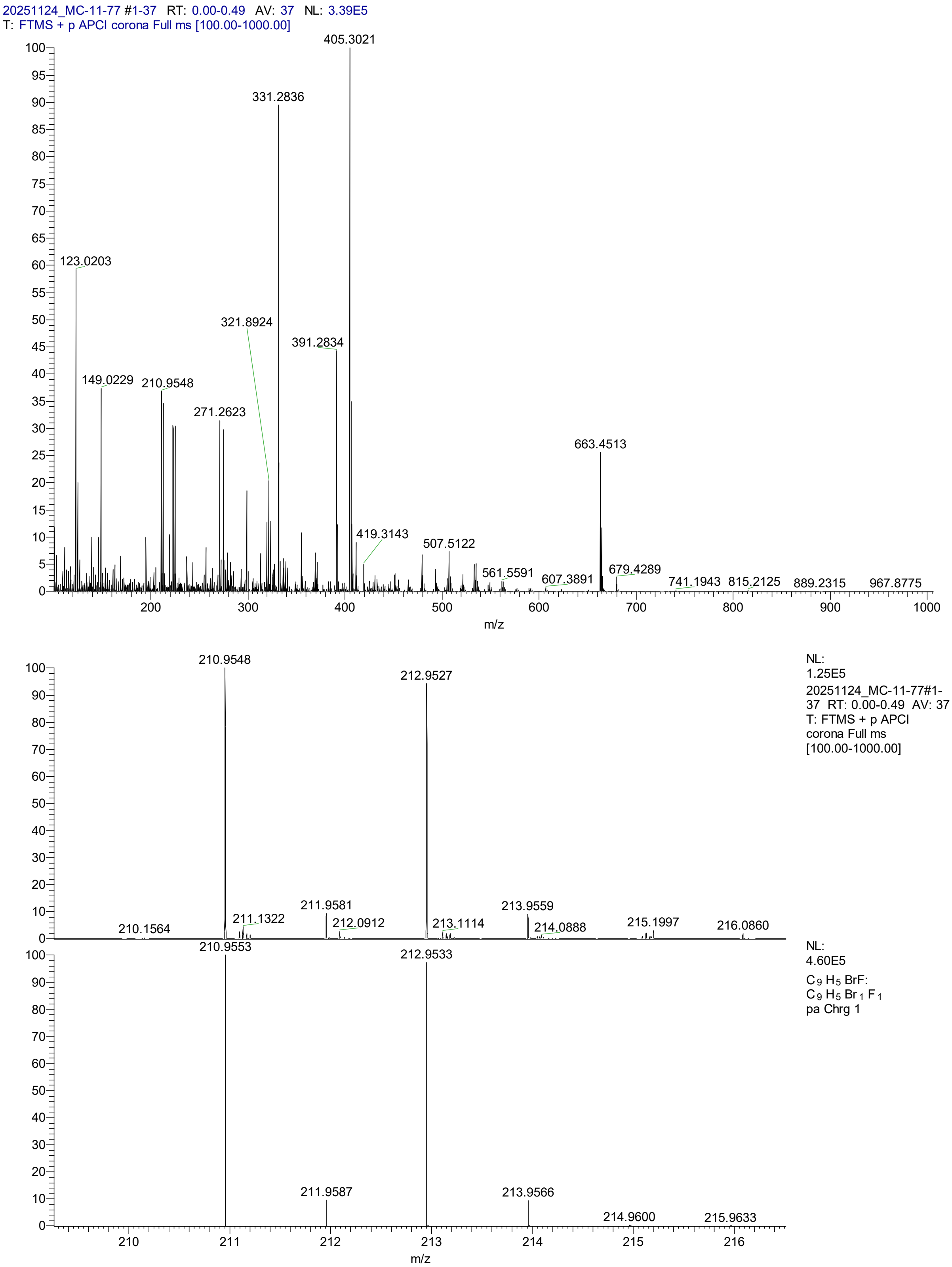

Figure S171: HRMS(APCI+) Spectra for **1h**.

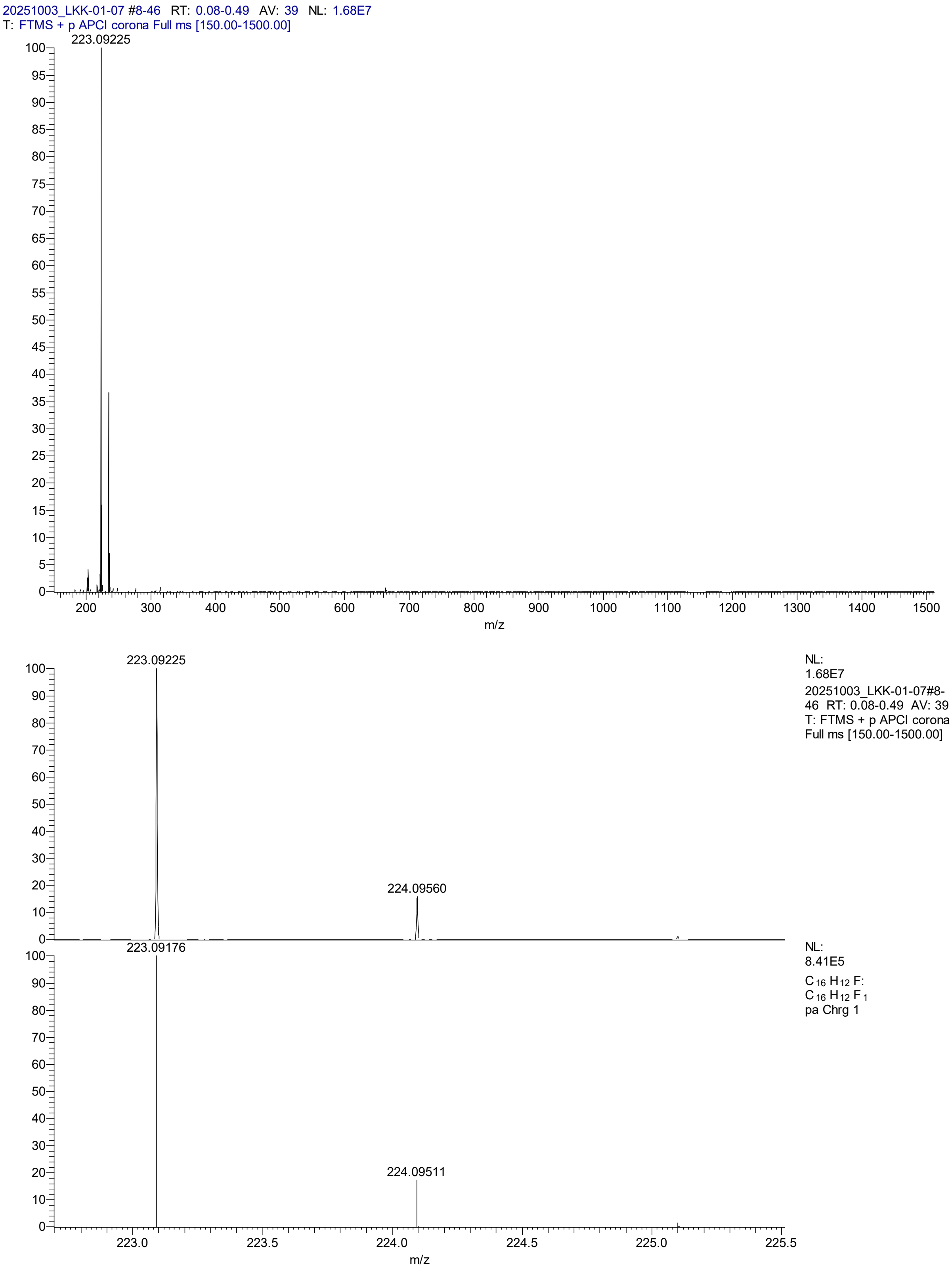

Figure S172: HRMS(APCI+) Spectra for **1i**.

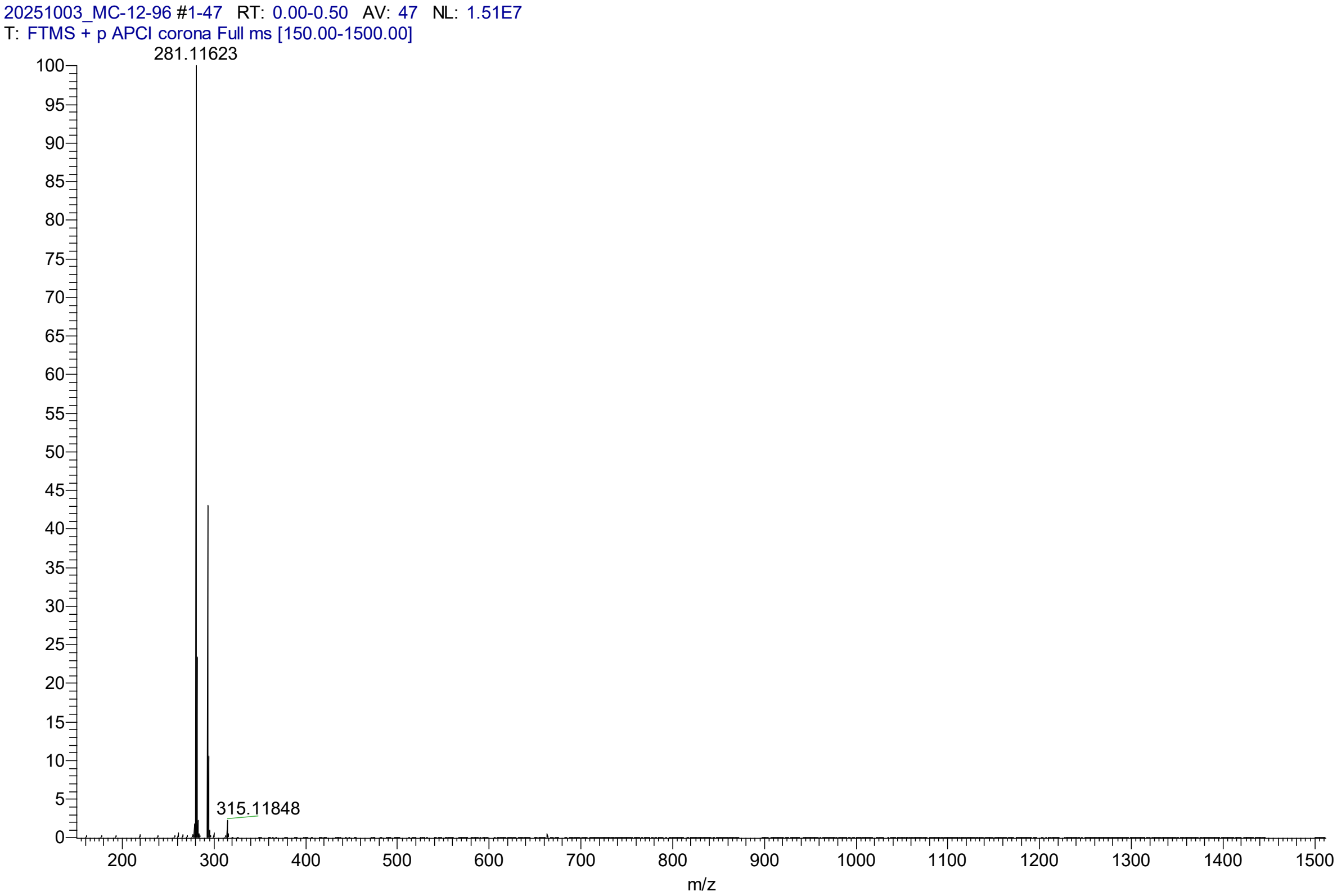

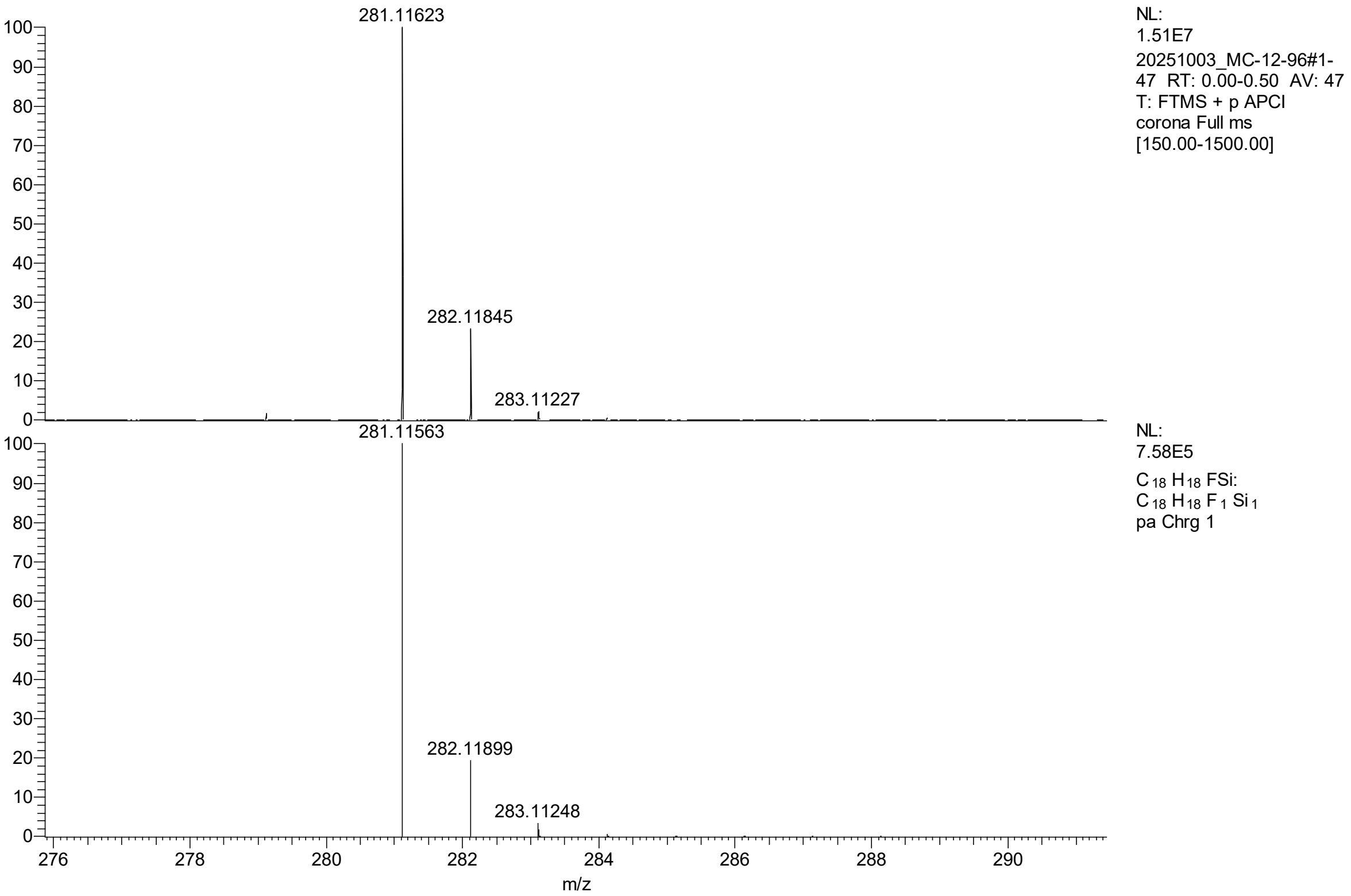

Figure S173: HRMS(APCI+) Spectra for **1j**.

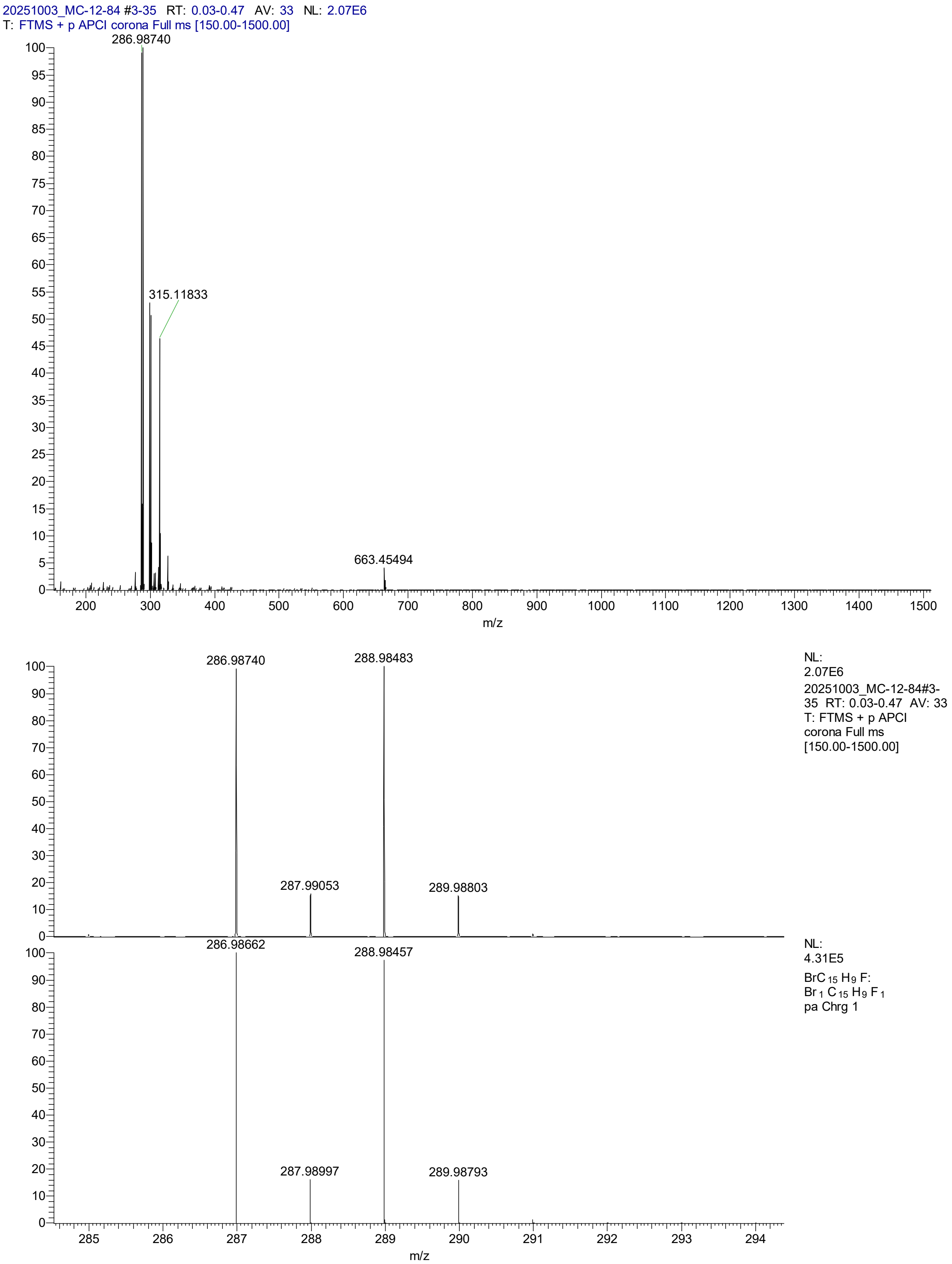

Figure S174: HRMS(APCI+) Spectra for **1k**.

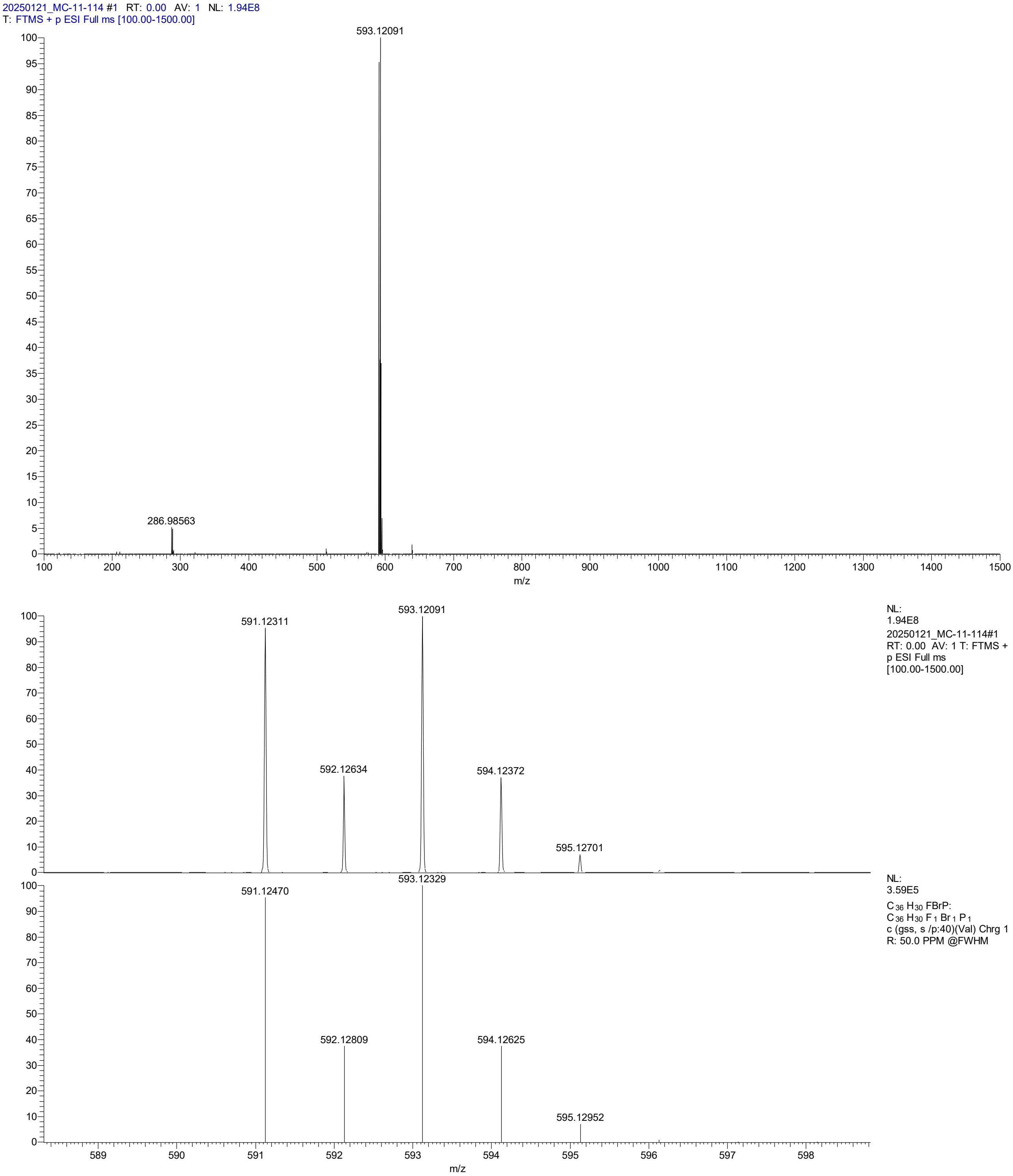

Figure S175: HRMS(ESI+) Spectra for **3a-PTol₃**.

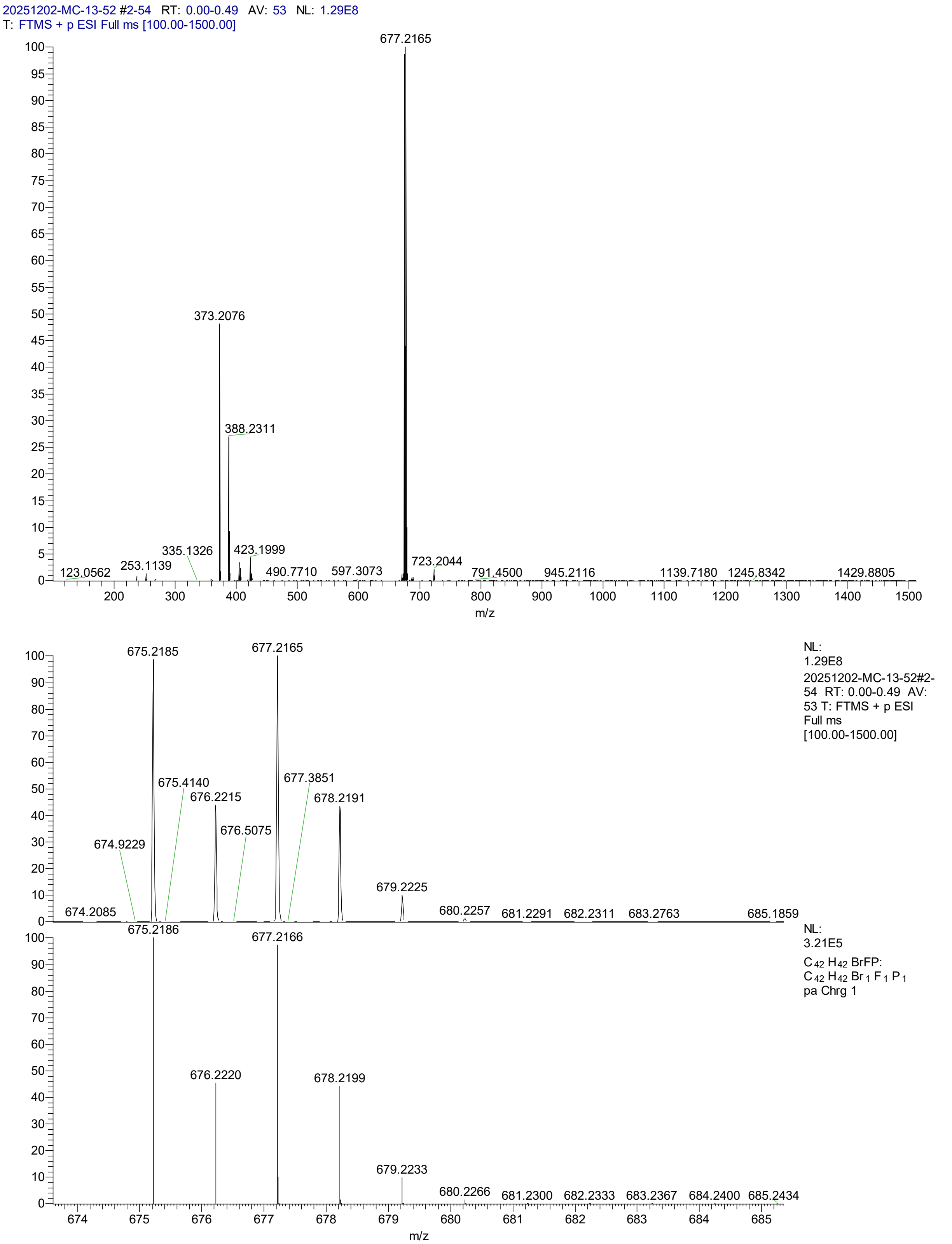

Figure S176: HRMS(ESI+) Spectra for **3a-PMes$_3$**.

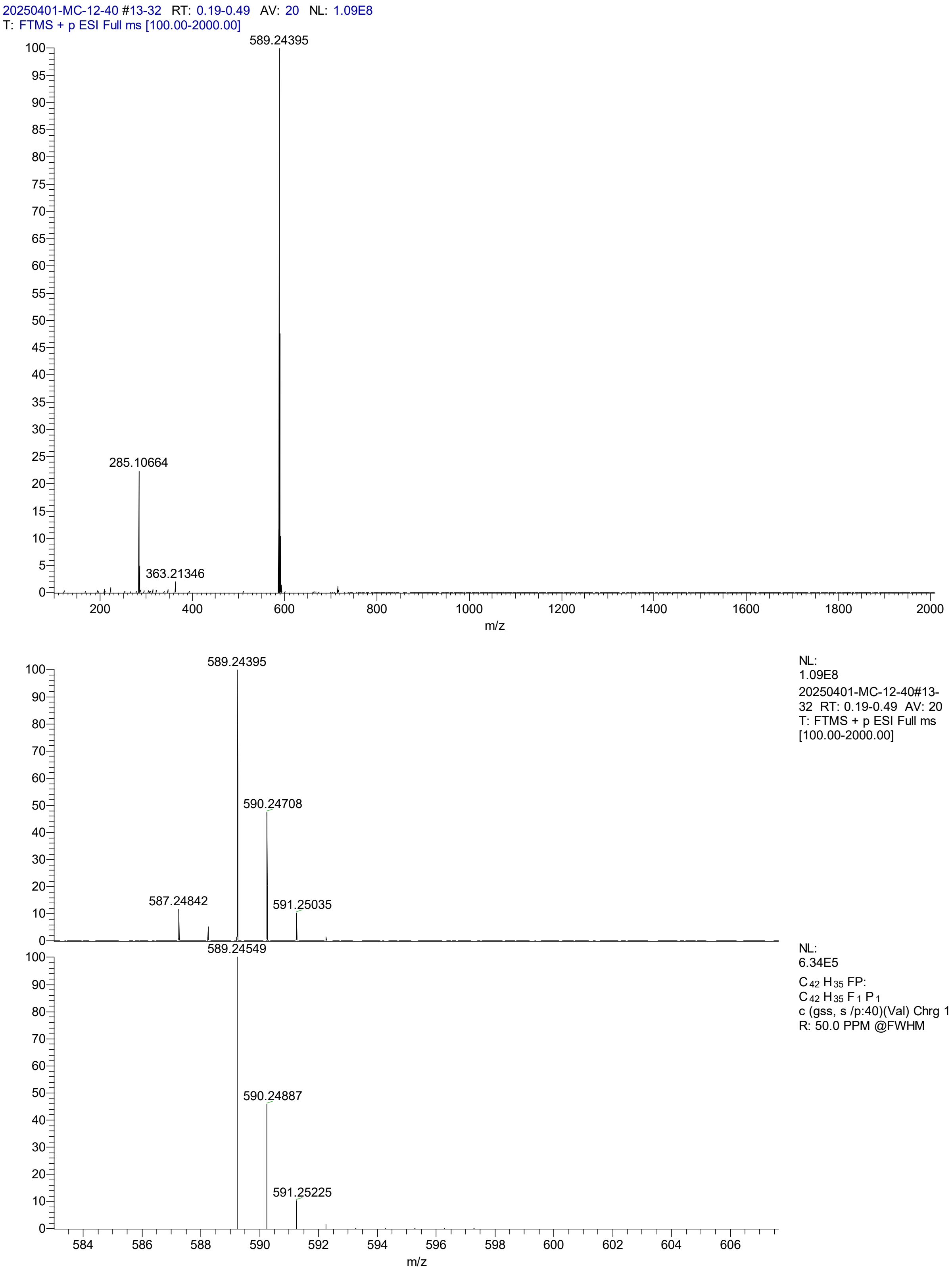

Figure S177: HRMS(ESI+) Spectra for **3b-PTol₃**.

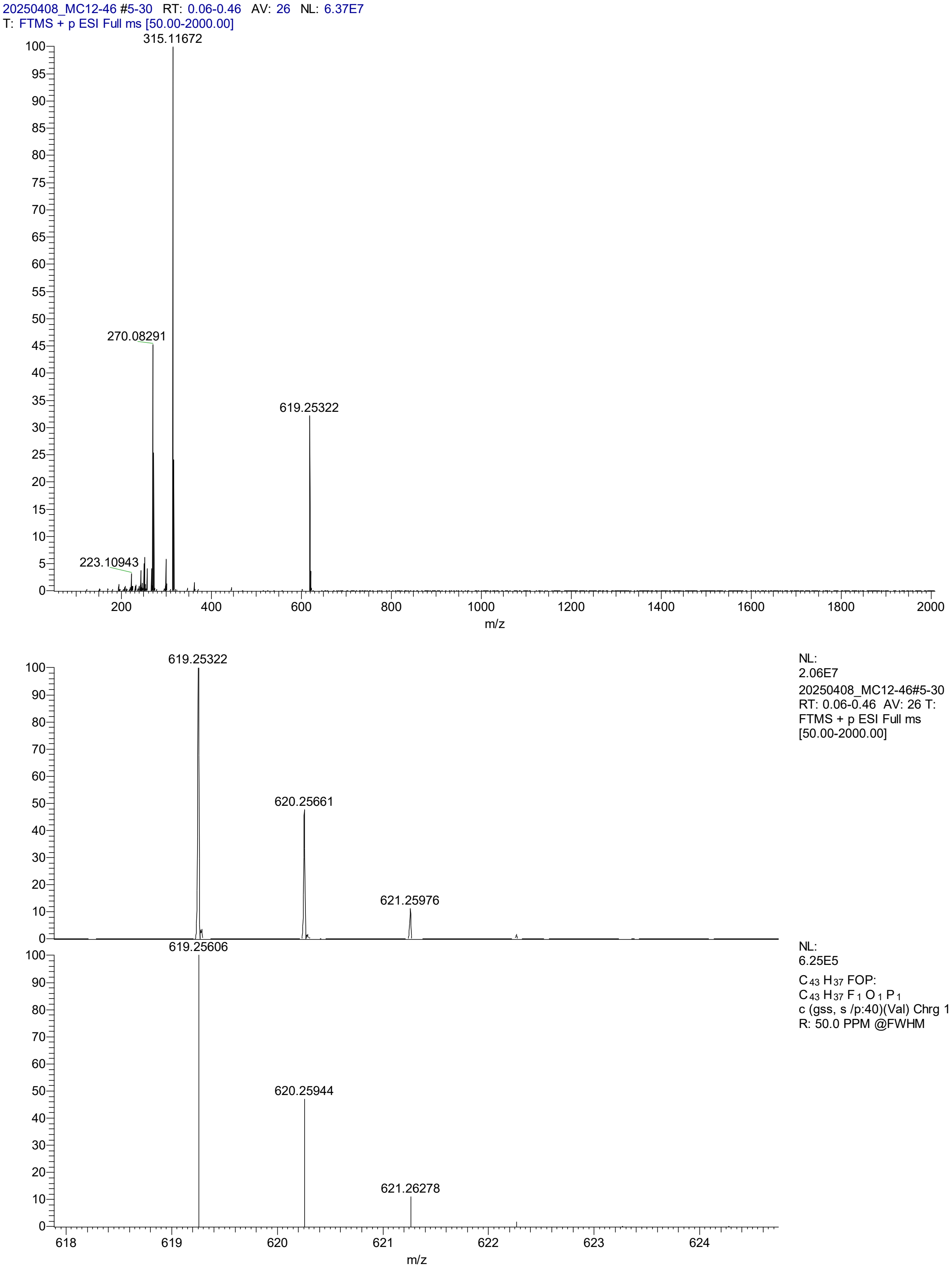

Figure S178: HRMS(ESI+) Spectra for **3d-PTol$_3$**.

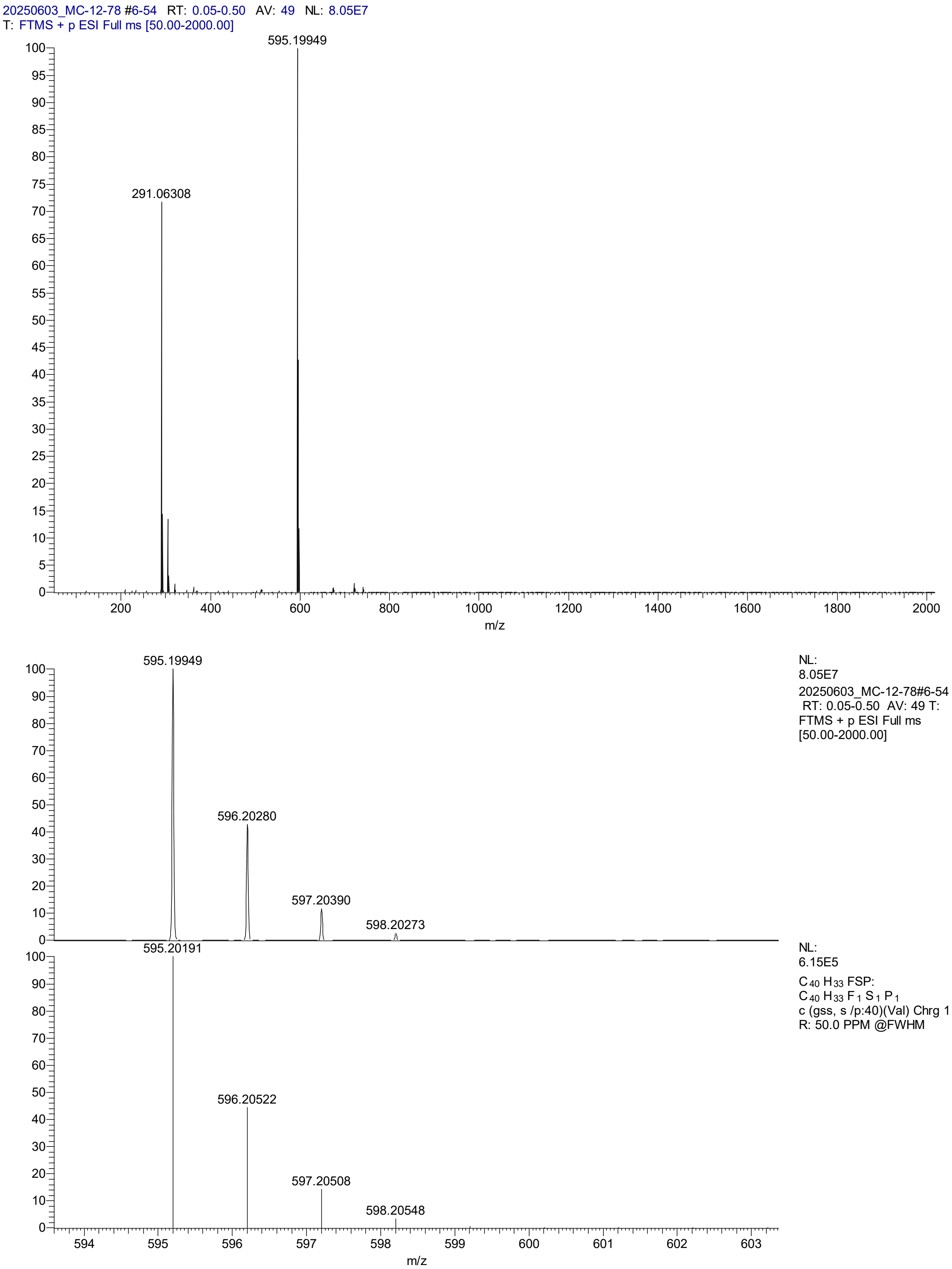

Figure S179: HRMS(ESI+) Spectra for **3e-PTol₃**.

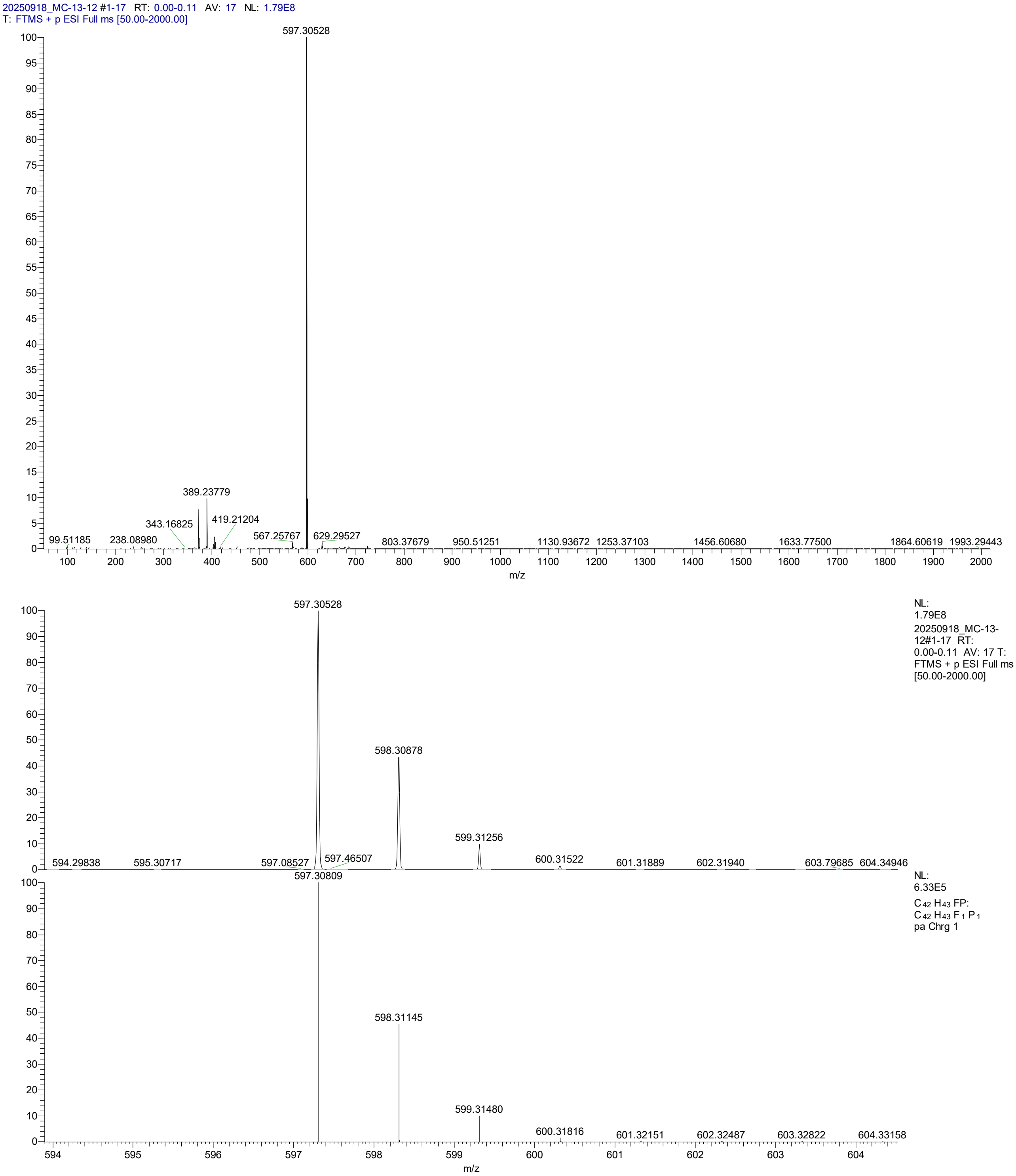

Figure S180: HRMS(ESI+) Spectra for **3f-PMes₃**.

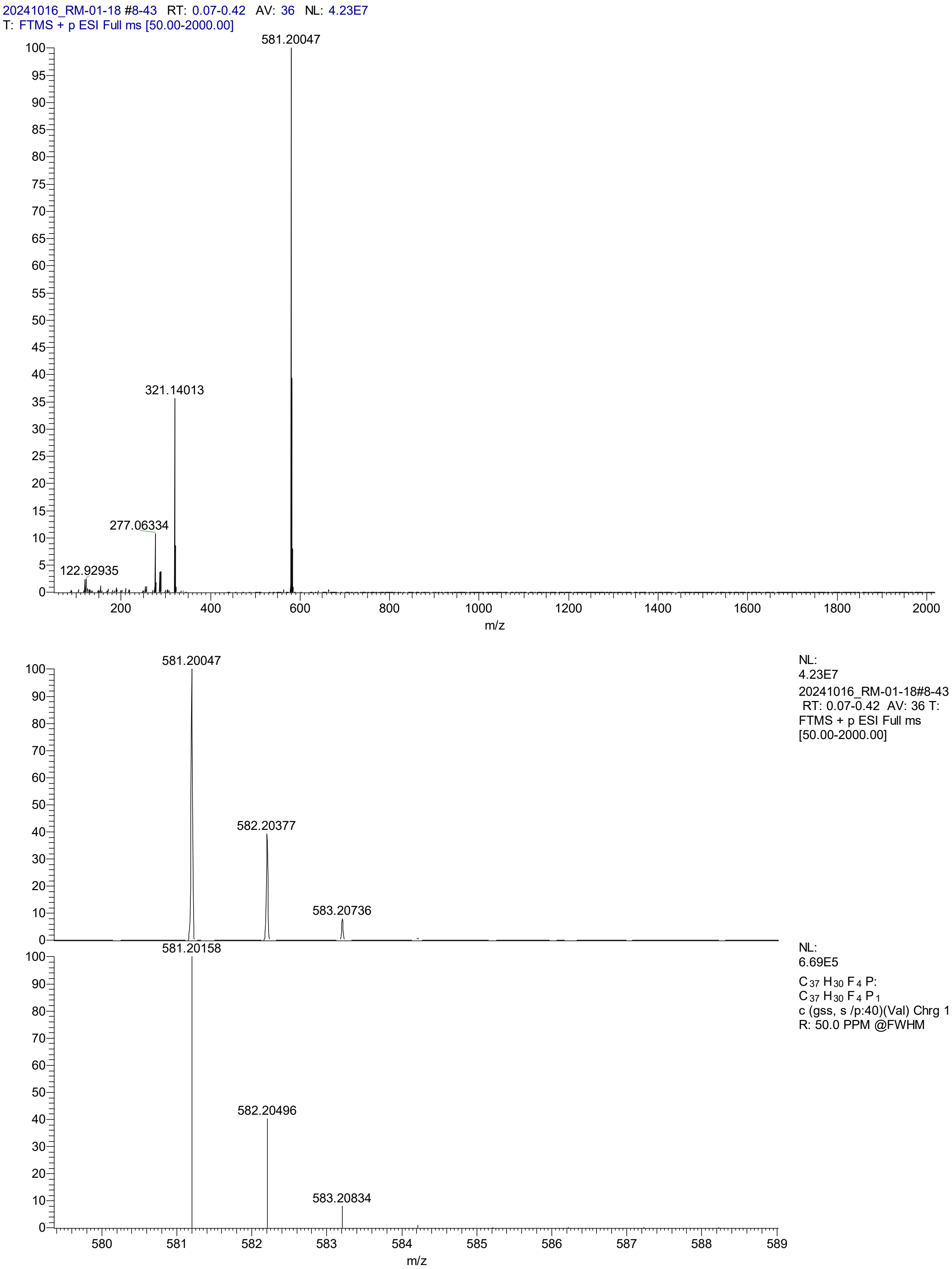

Figure S181: HRMS(ESI+) Spectra for **3f-PTol₃**.

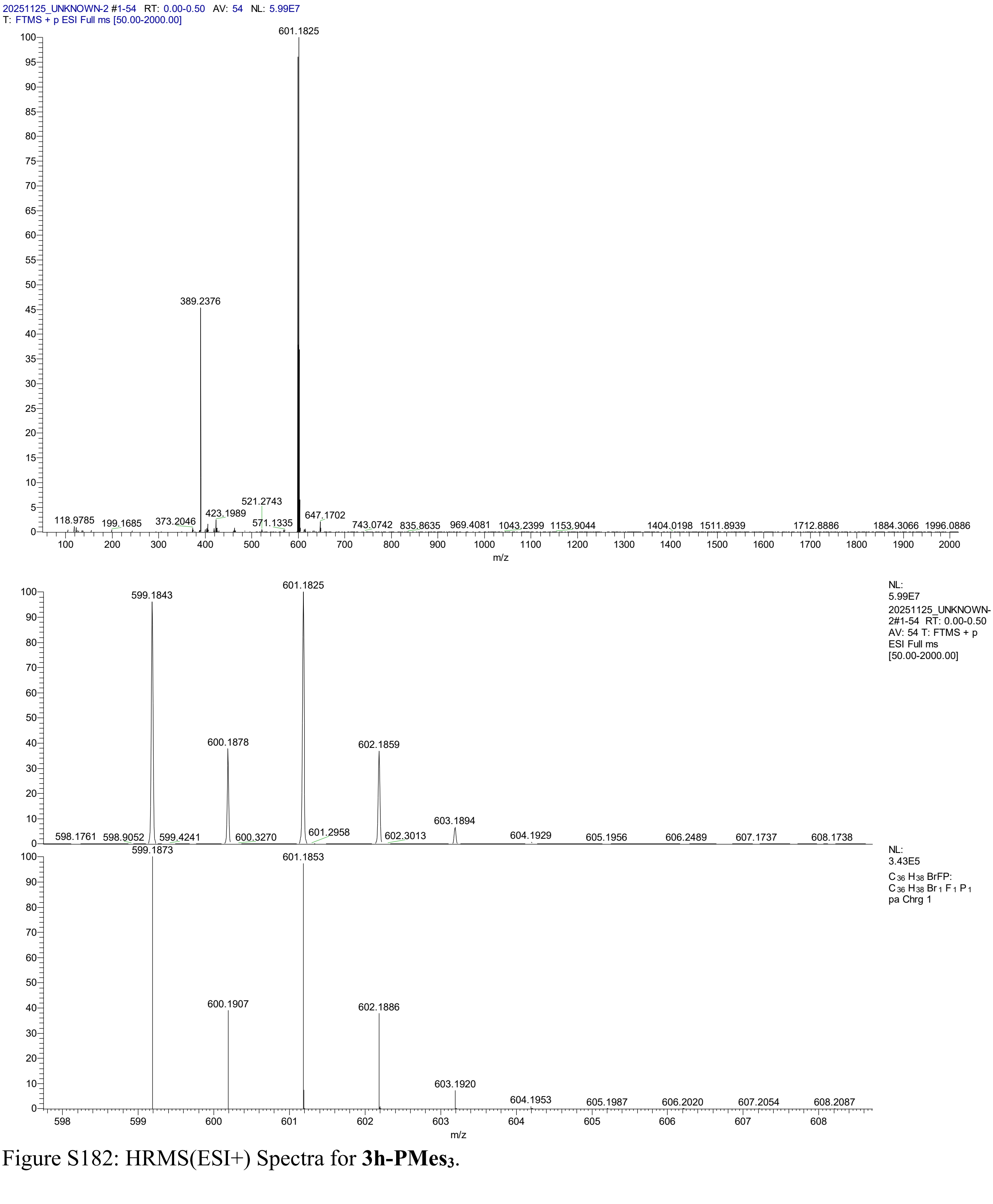

Figure S182: HRMS(ESI+) Spectra for **3h-PMes$_3$**.

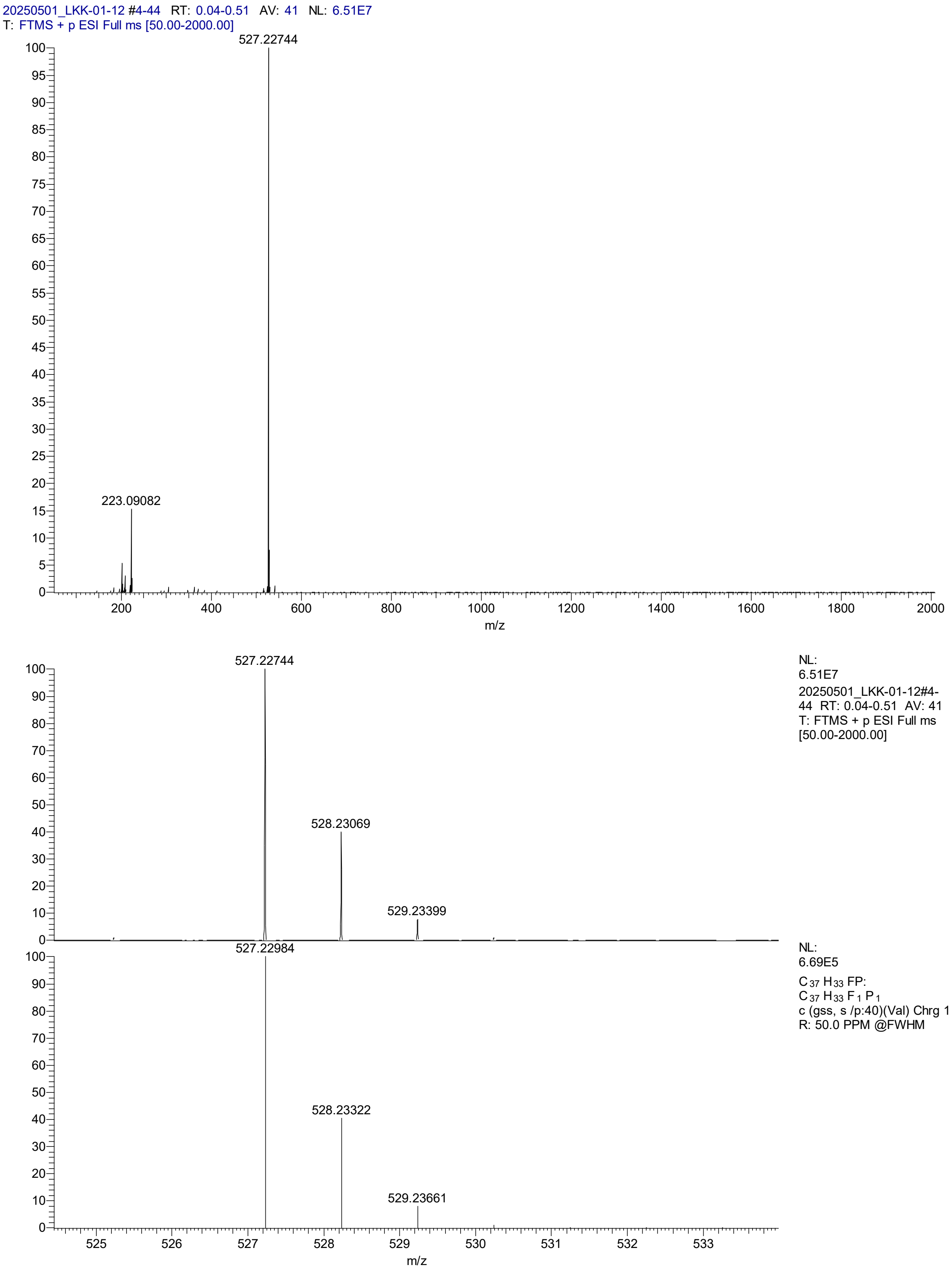

Figure S183: HRMS(ESI+) Spectra for **3i-PTol$_3$**.

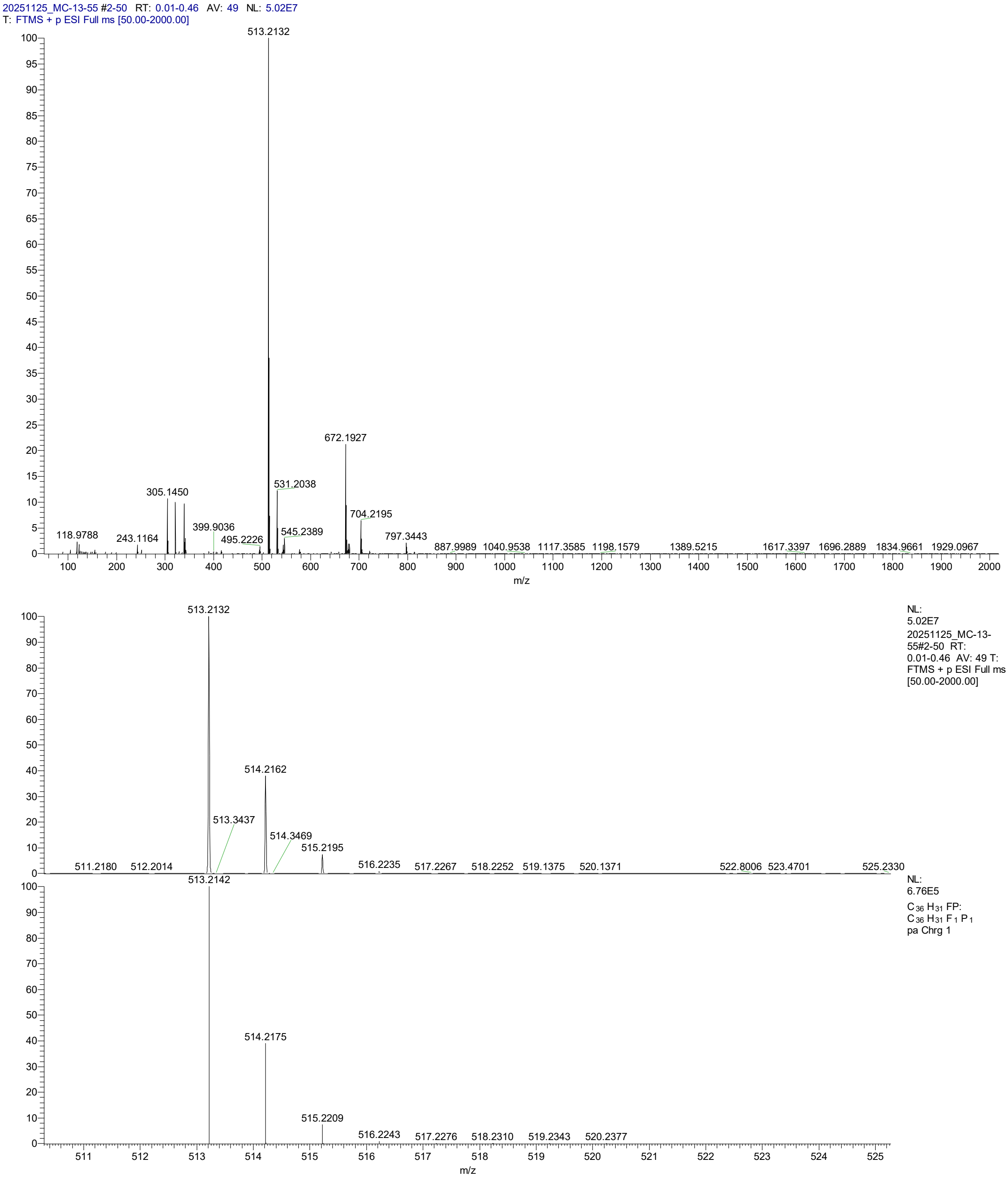

Figure S184: HRMS(ESI+) Spectra for **2m-PTol$_3$**.

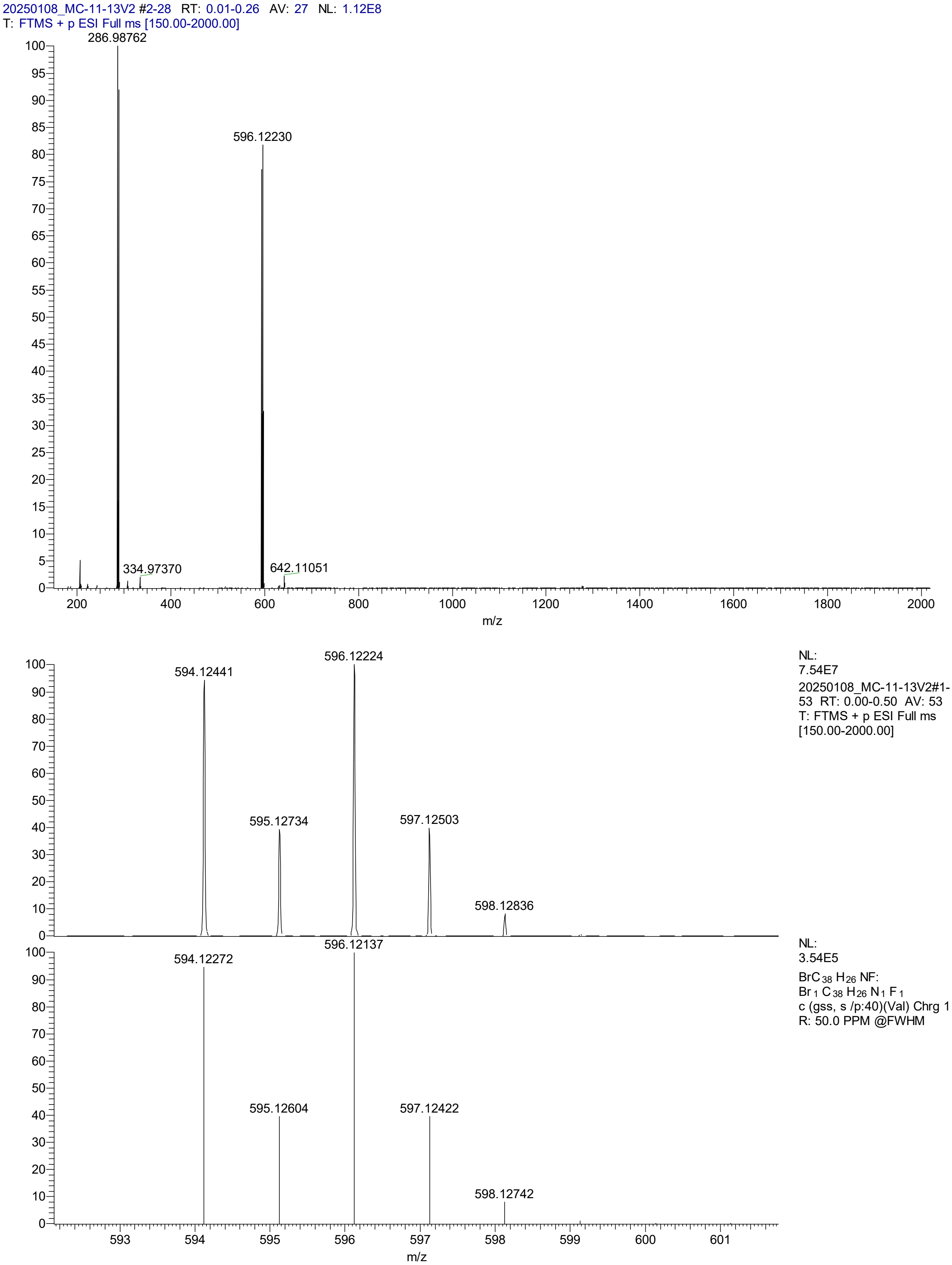

Figure S185: HRMS(ESI+) Spectra for **3a-TPPy**.

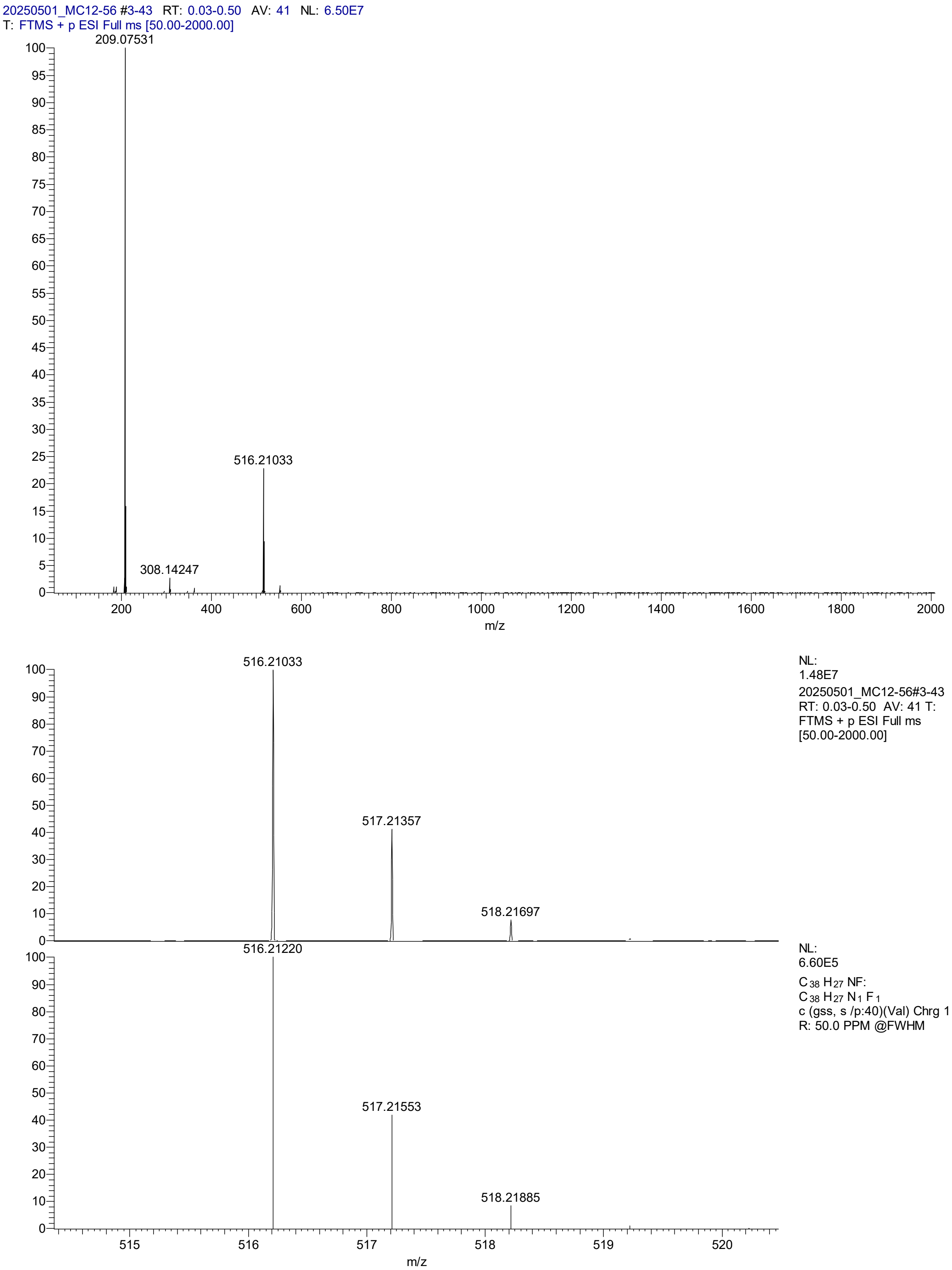

Figure S186: HRMS(ESI+) Spectra for **3c-TPPy**.

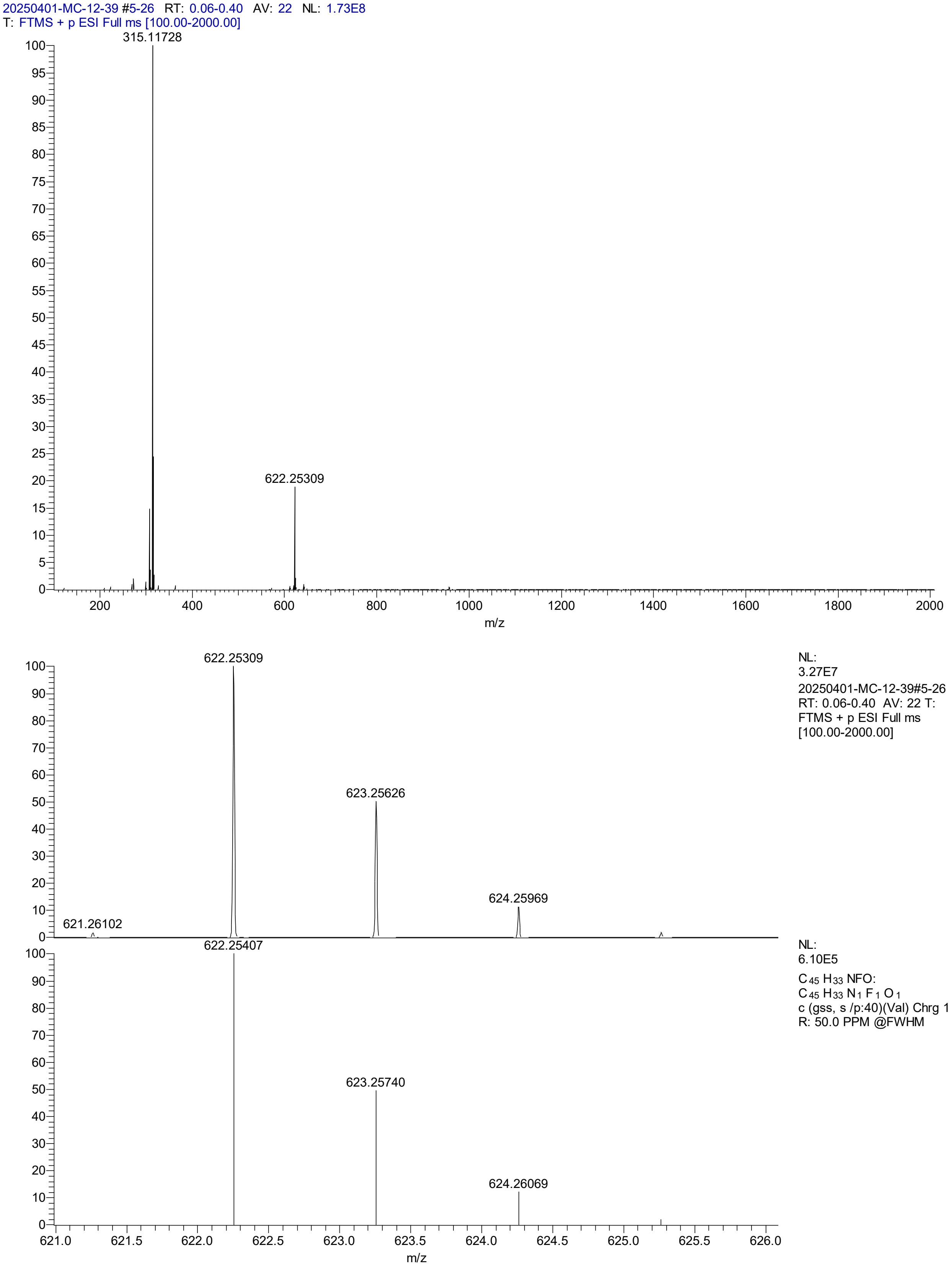

Figure S187: HRMS(ESI+) Spectra for **3d-TPPy**.

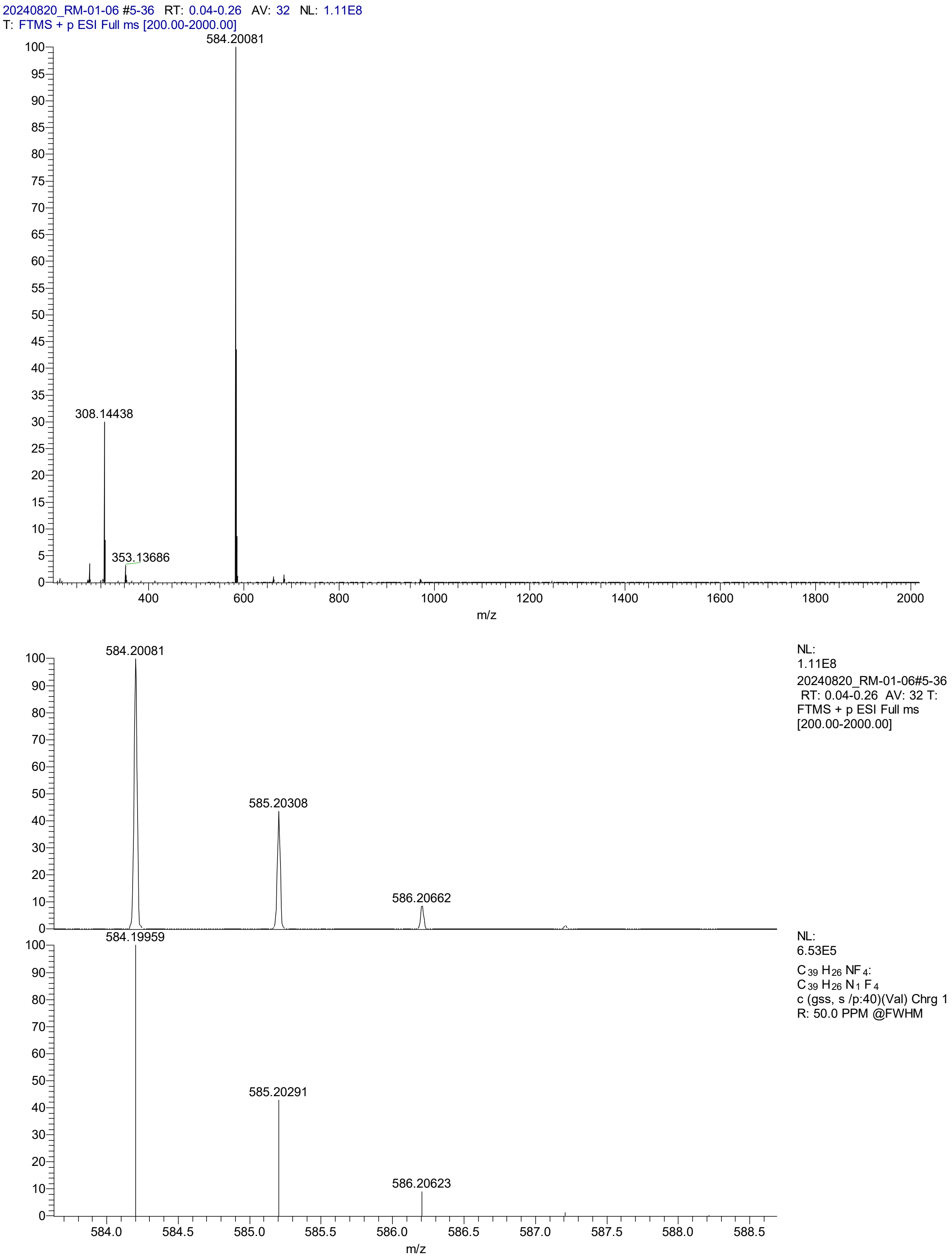

Figure S188: HRMS(ESI+) Spectra for **3f-TPPy**.

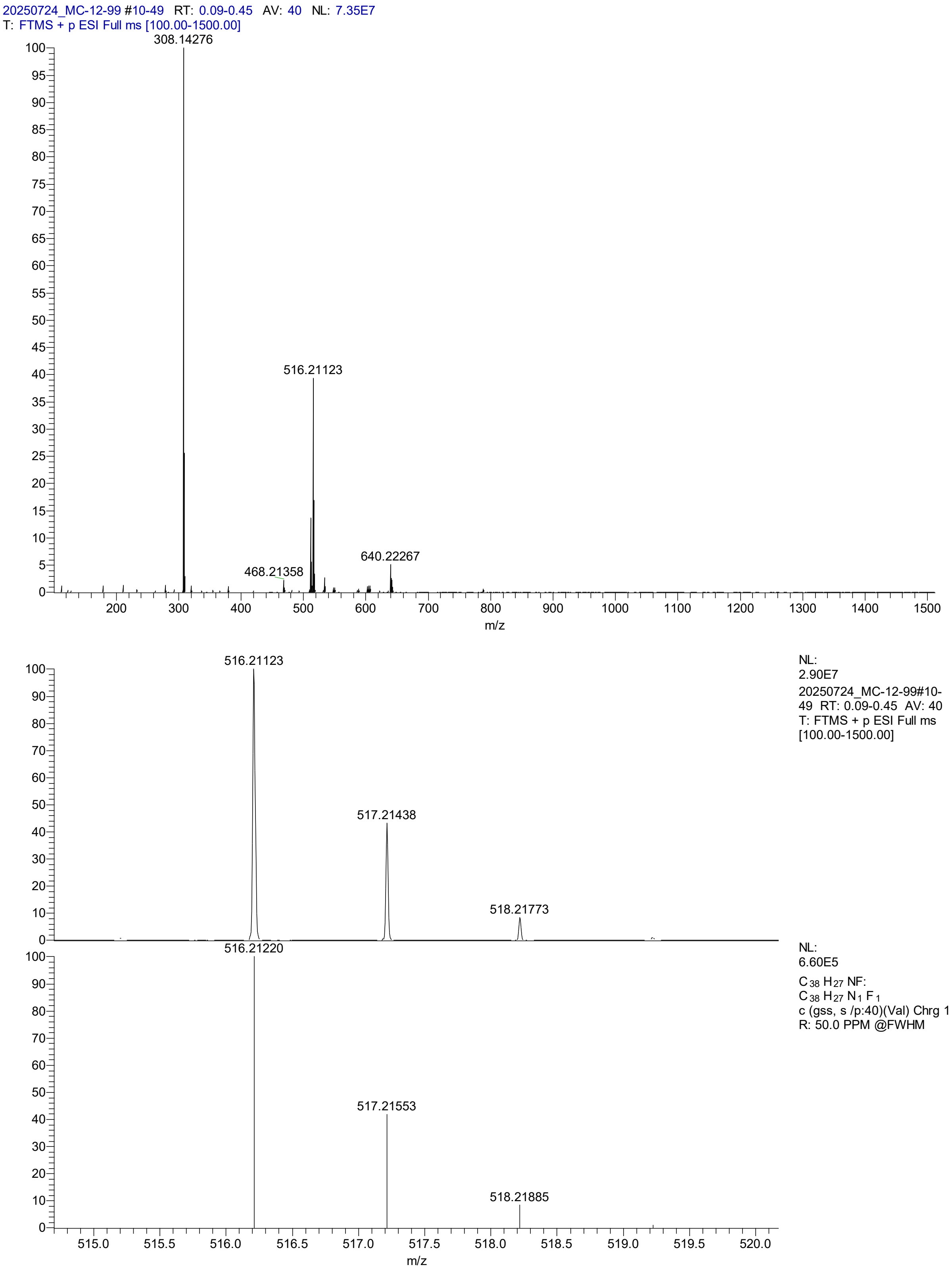

Figure S189: HRMS(ESI+) Spectra for **3j-TPPy**.

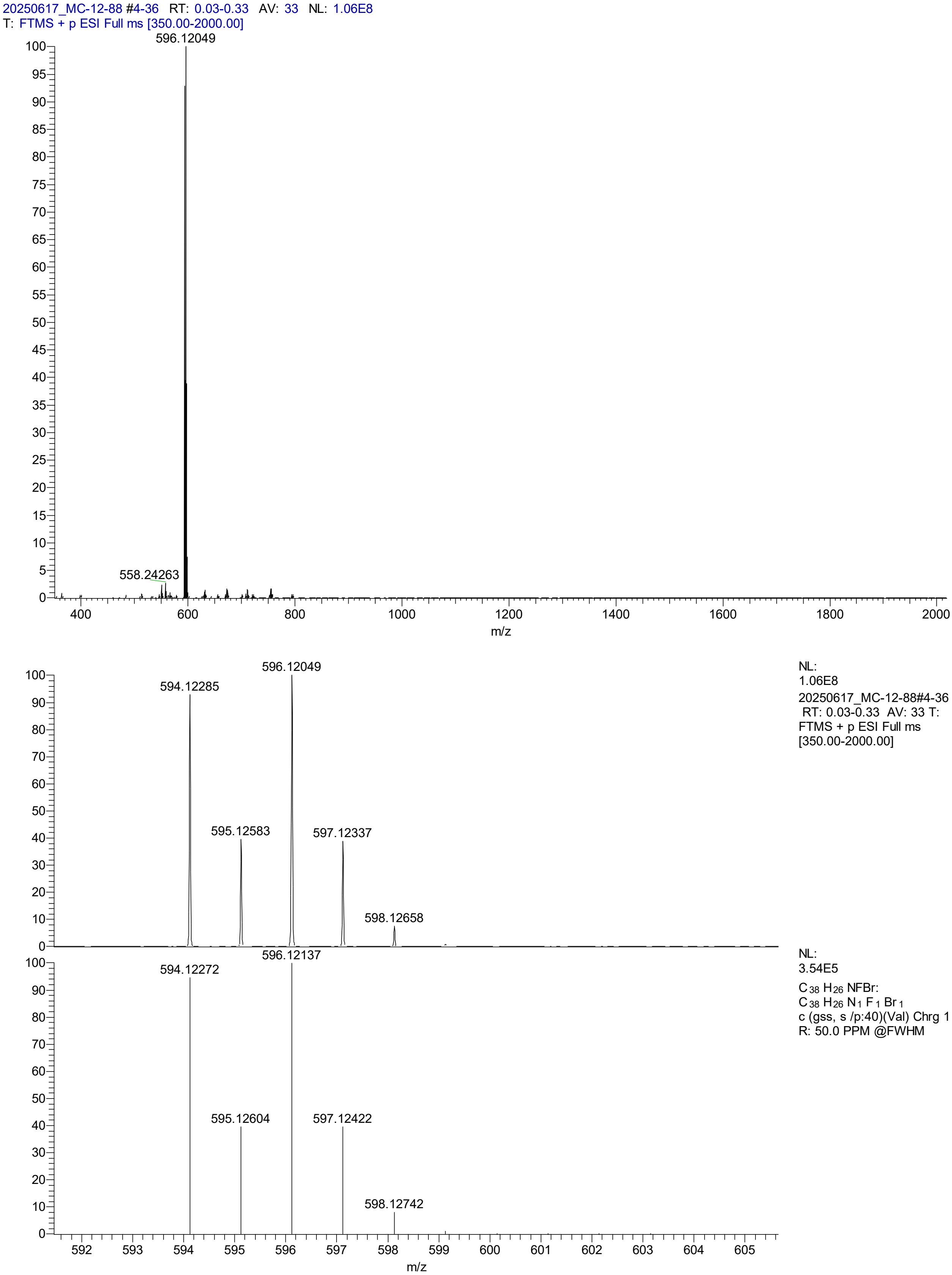

Figure S190: HRMS(ESI+) Spectra for **3k-TPPy**.

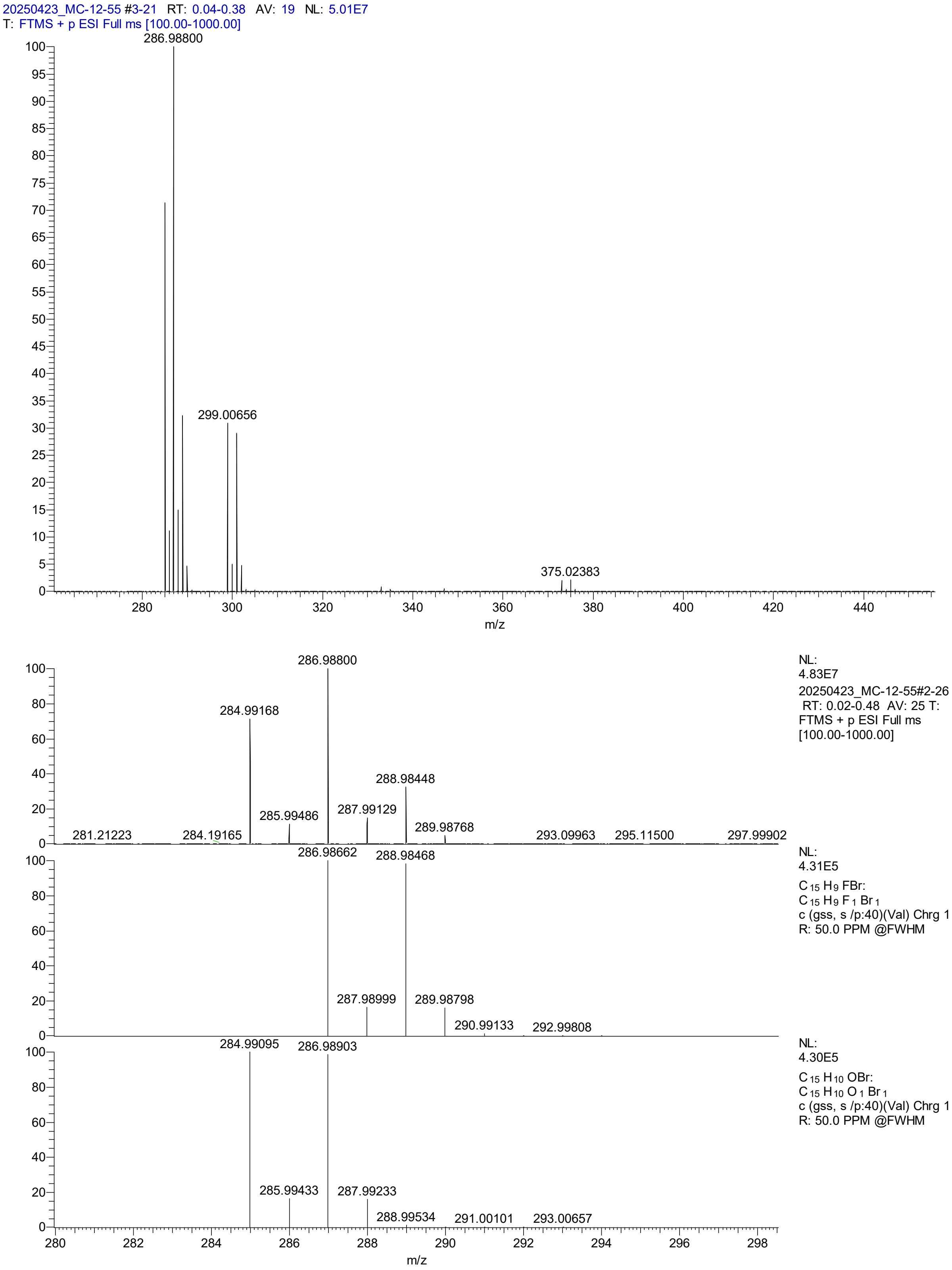

Figure S191: HRMS(ESI+) Spectra for **3a-THT**.

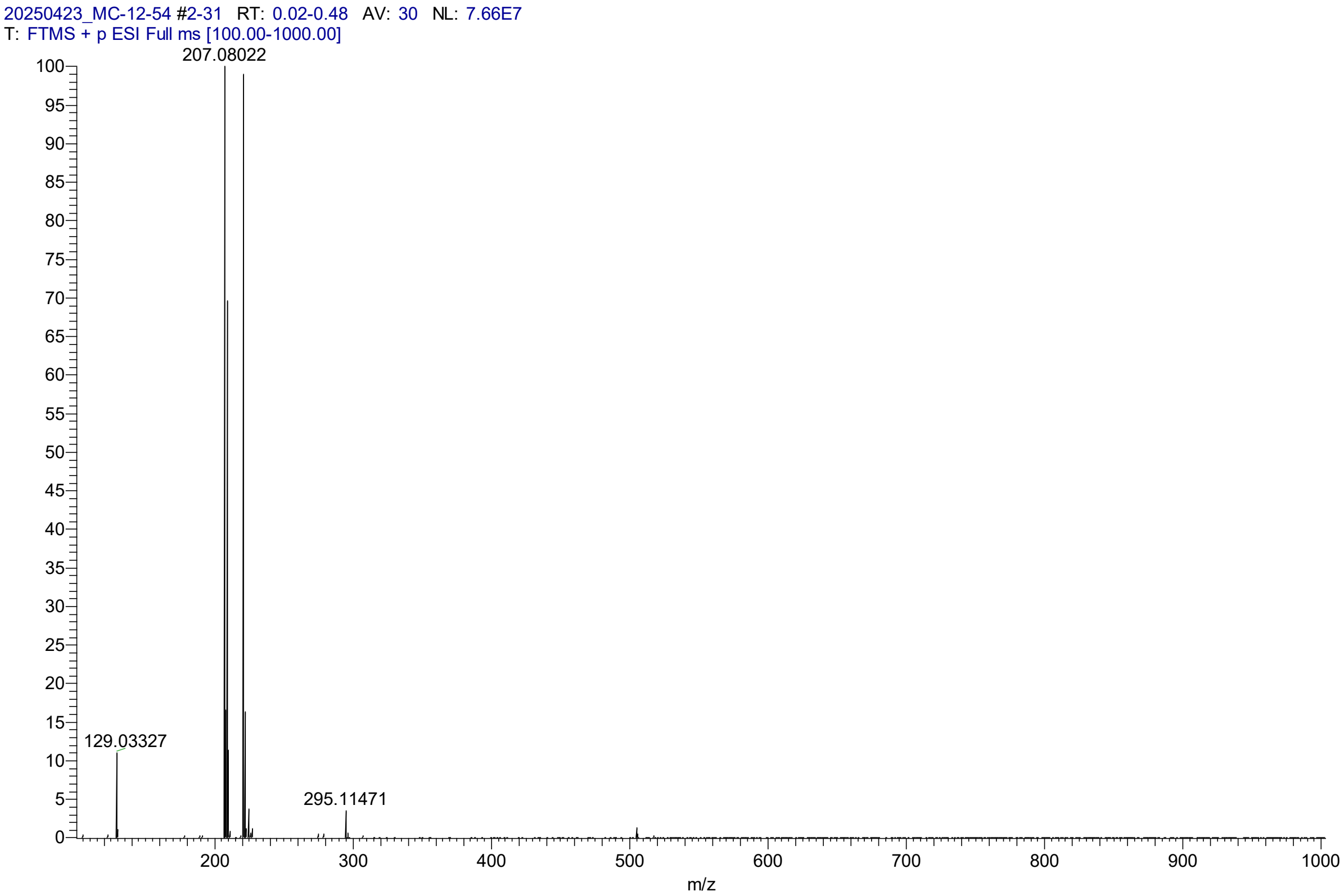

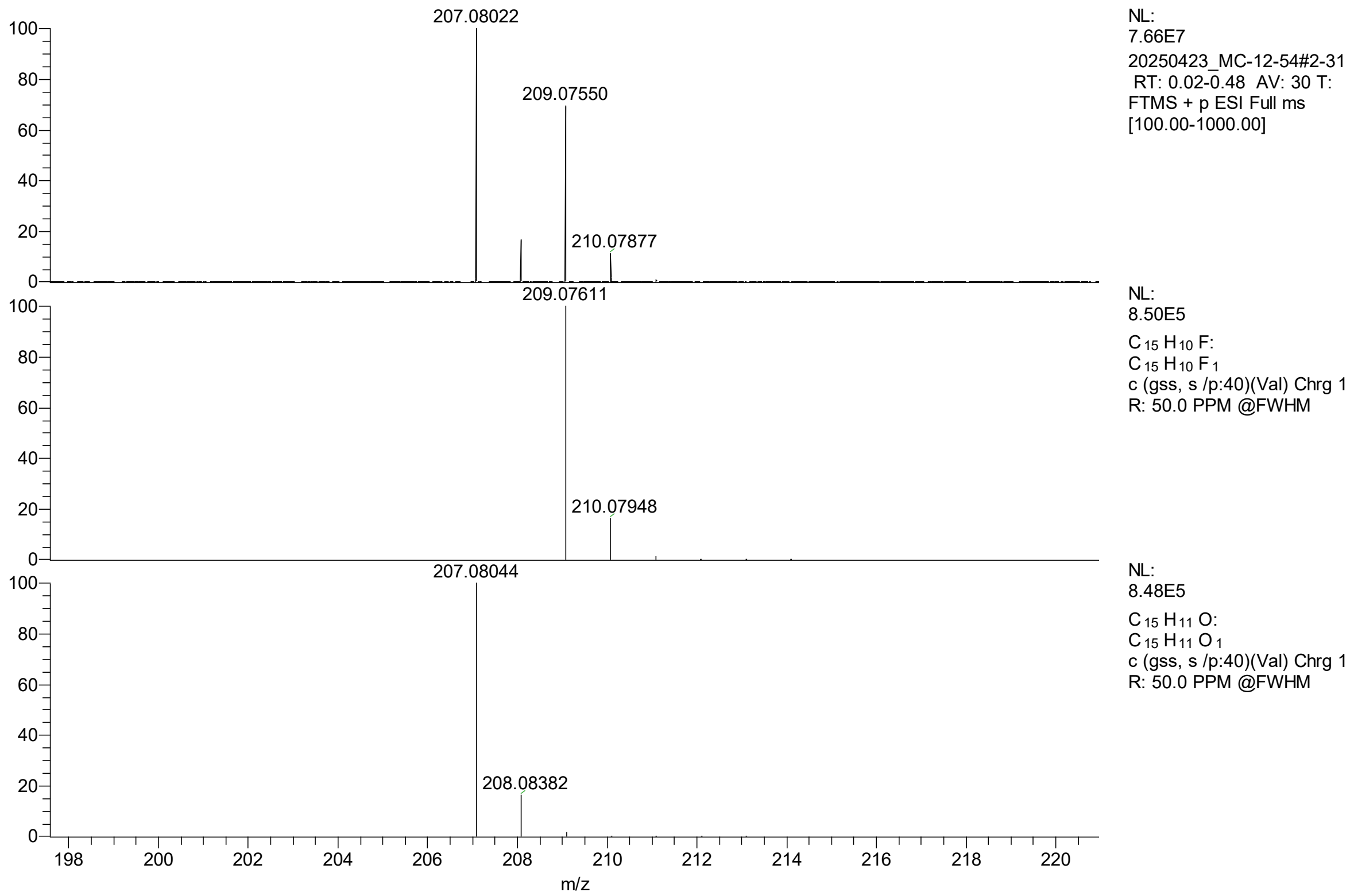

Figure S192: HRMS(ESI+) Spectra for **3c-THT**.

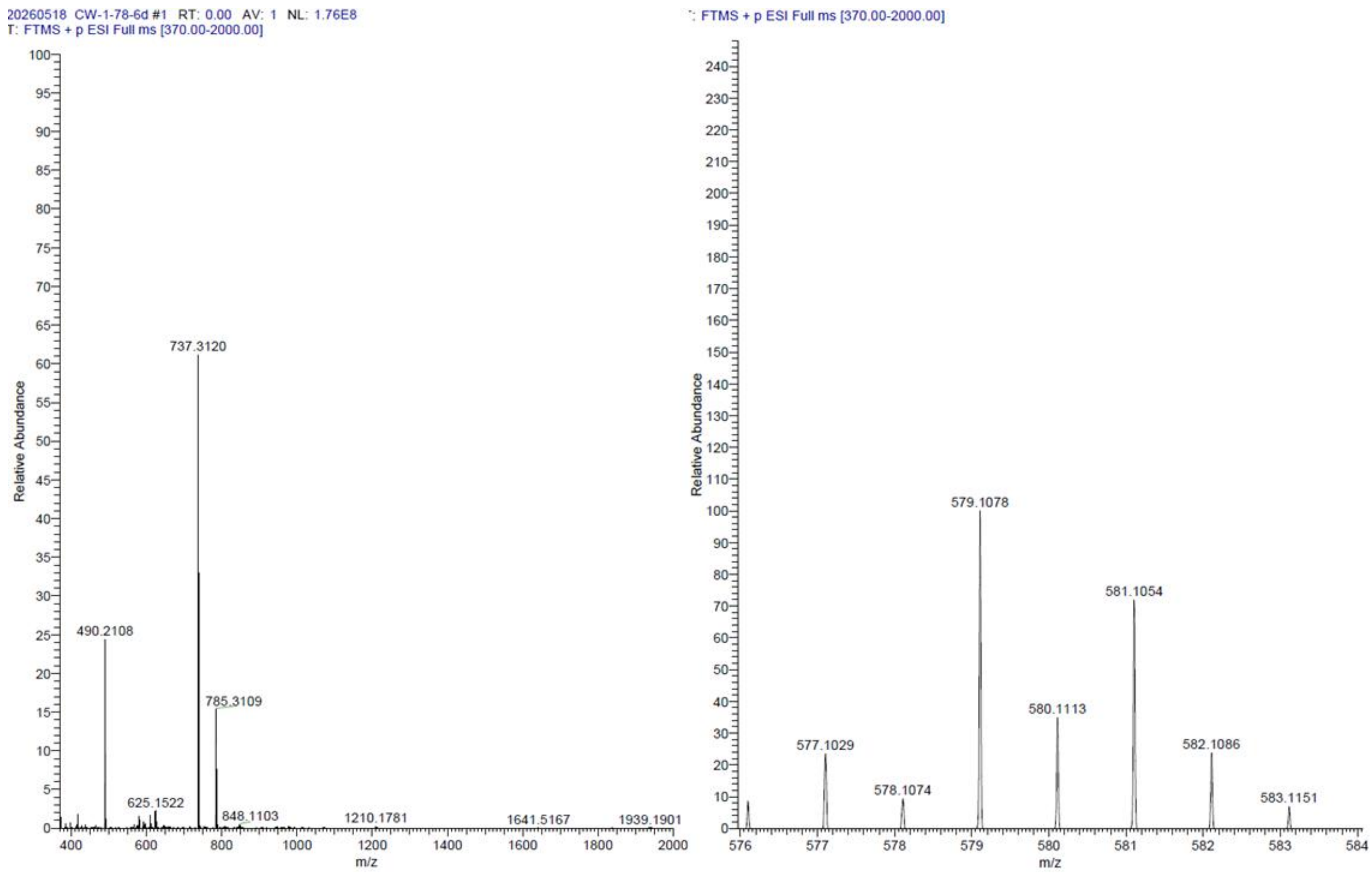

Figure S193: HRMS(ESI+) Spectra for **3i-LB$_B$***.

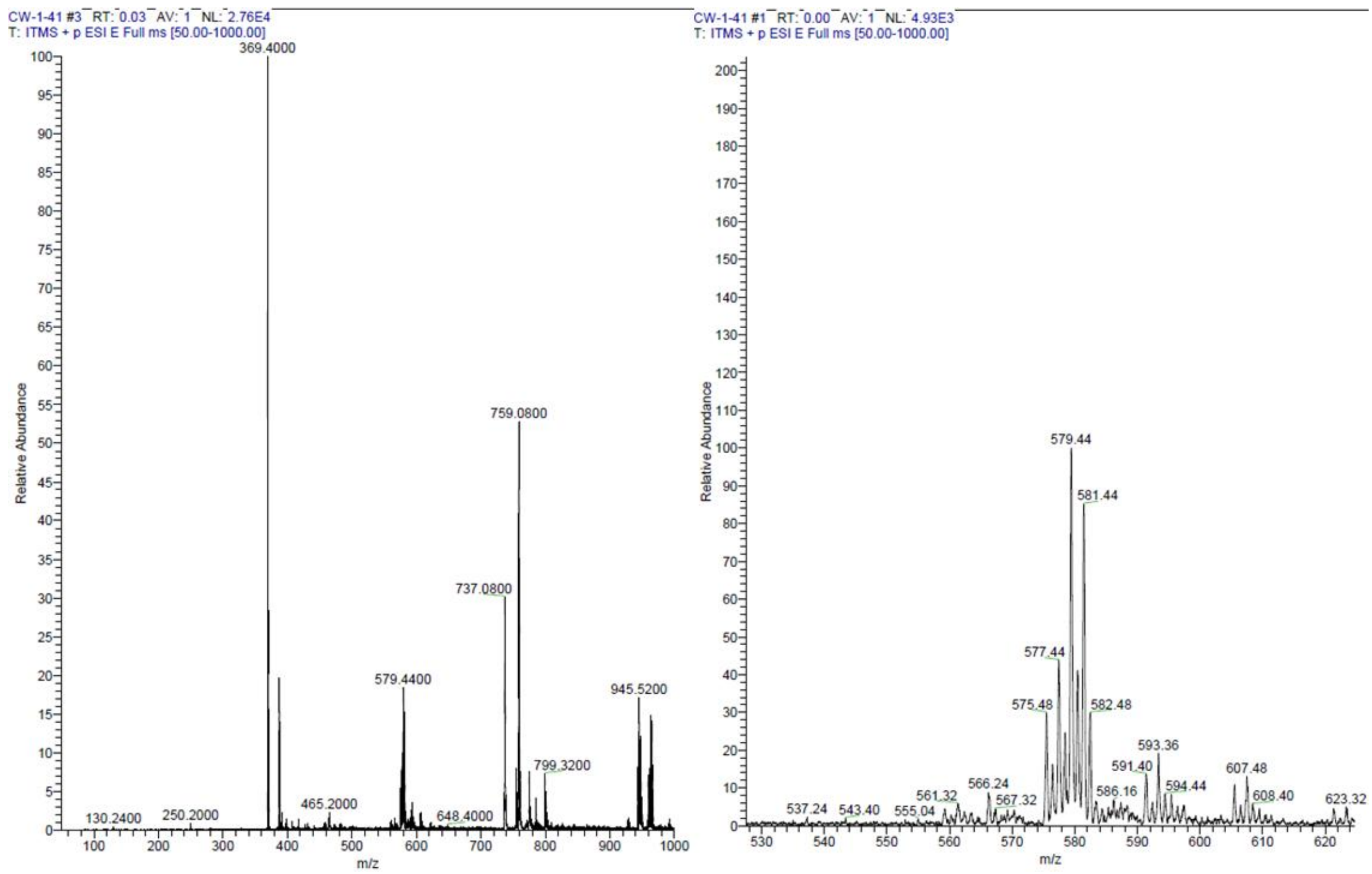

Figure S194: LRMS(ESI+) Spectra for **3i-LB$_B$***.

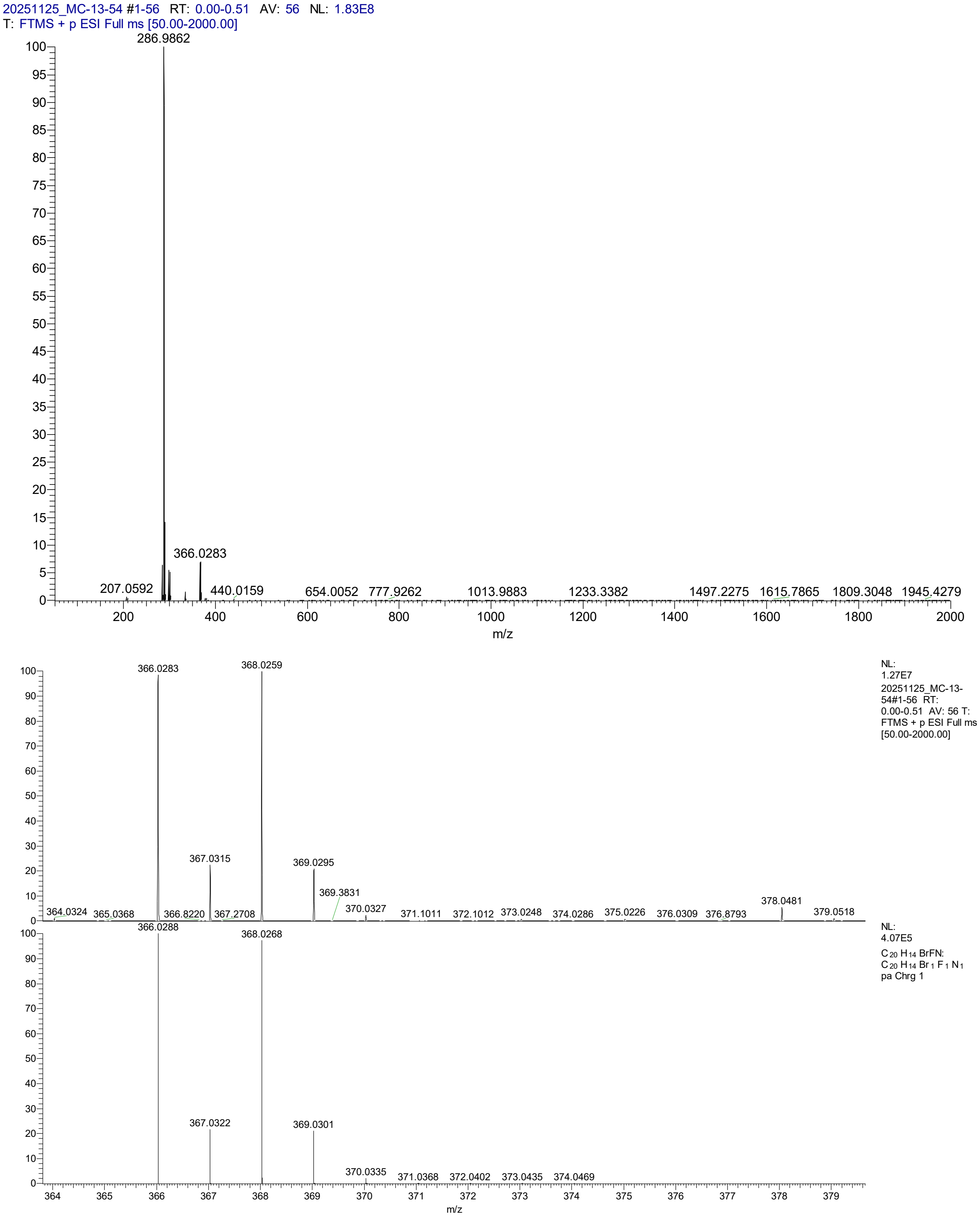

Figure S195: HRMS(ESI+) Spectra for **2a-Py**.

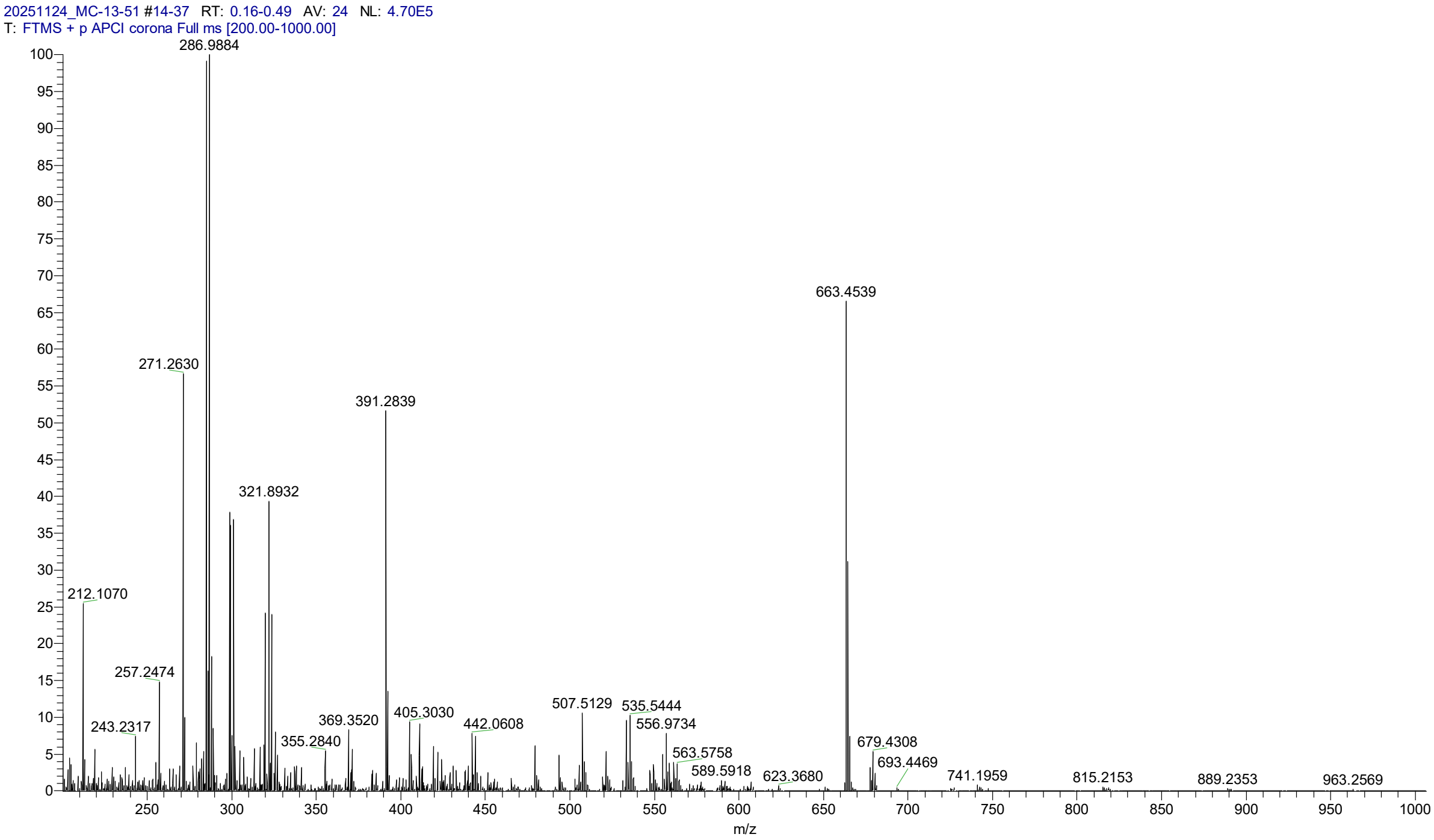

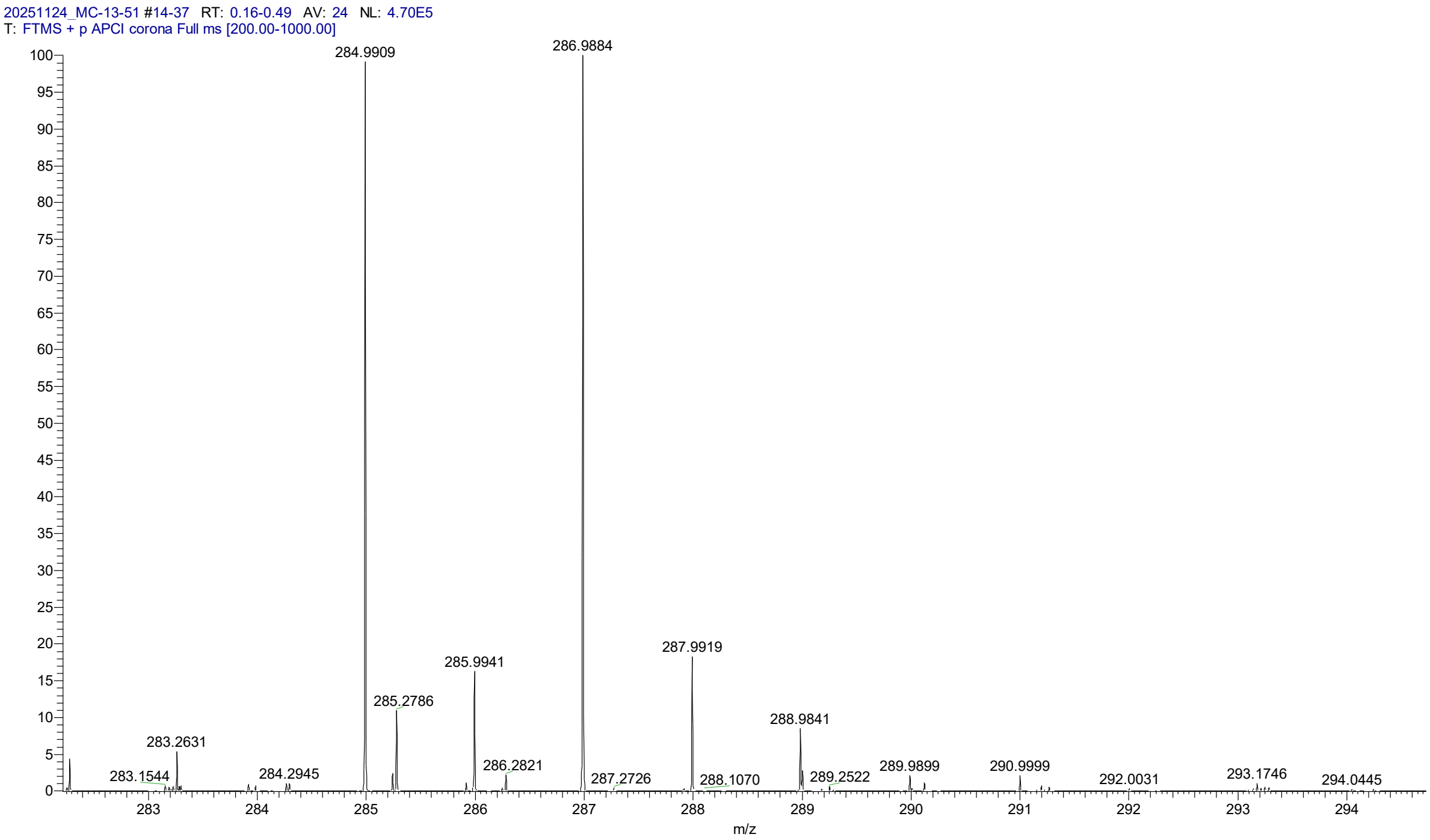

Figure S196: HRMS(ESI+) Spectra for **3a-PhPy**.

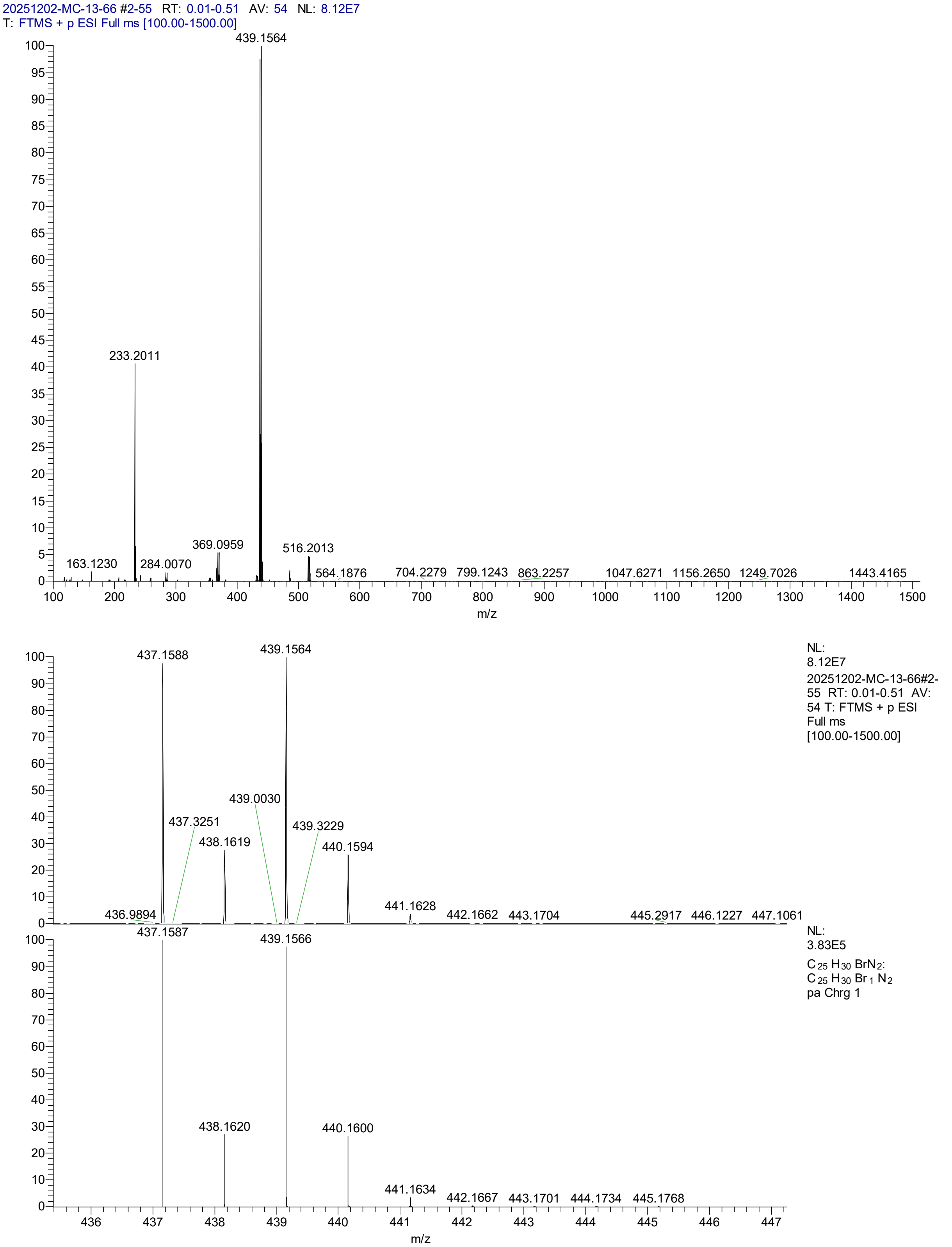

Figure S197: HRMS(ESI+) Spectra for **5a**.

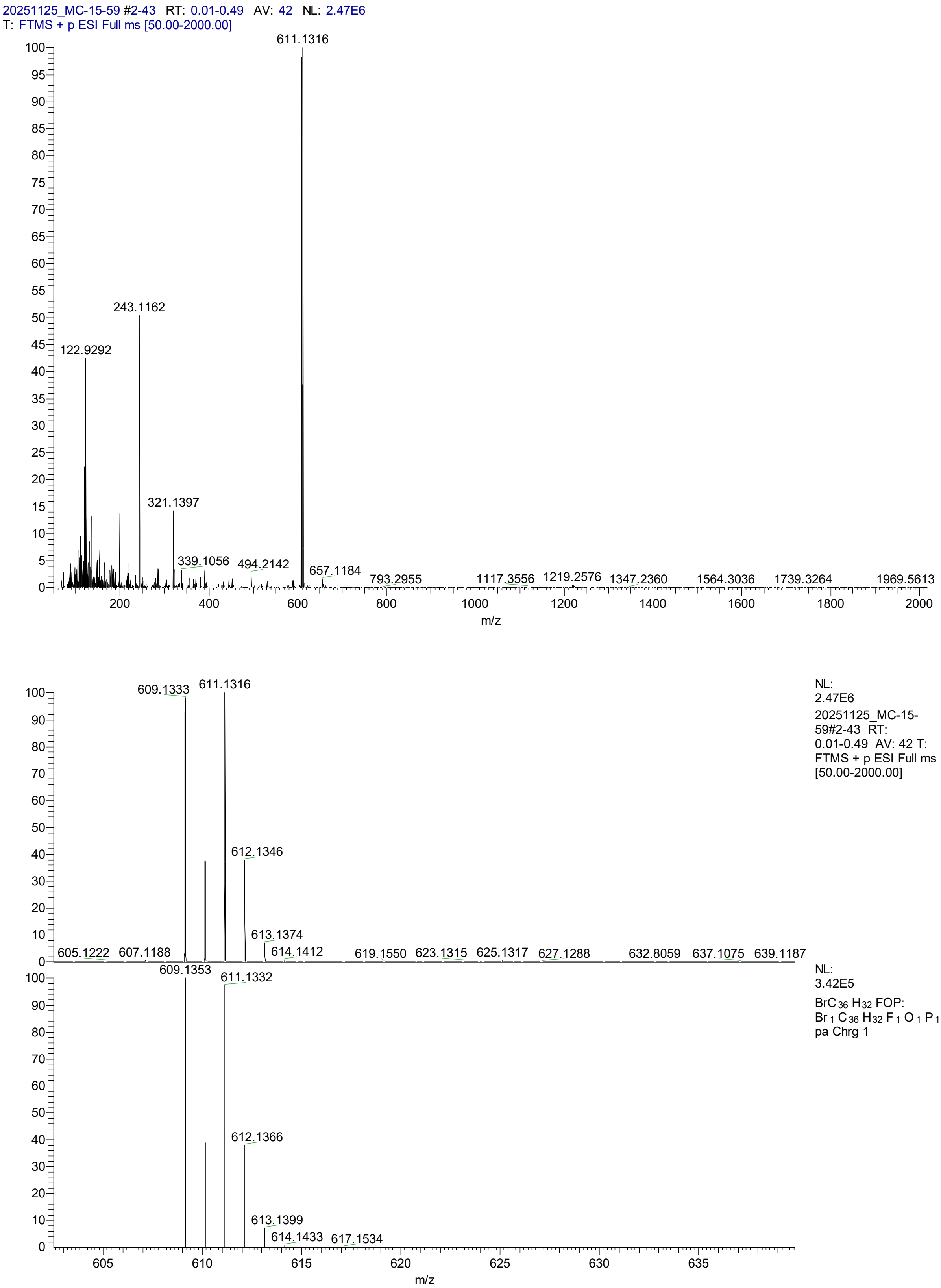

Figure S198: HRMS(ESI+) Spectra for **5b**.

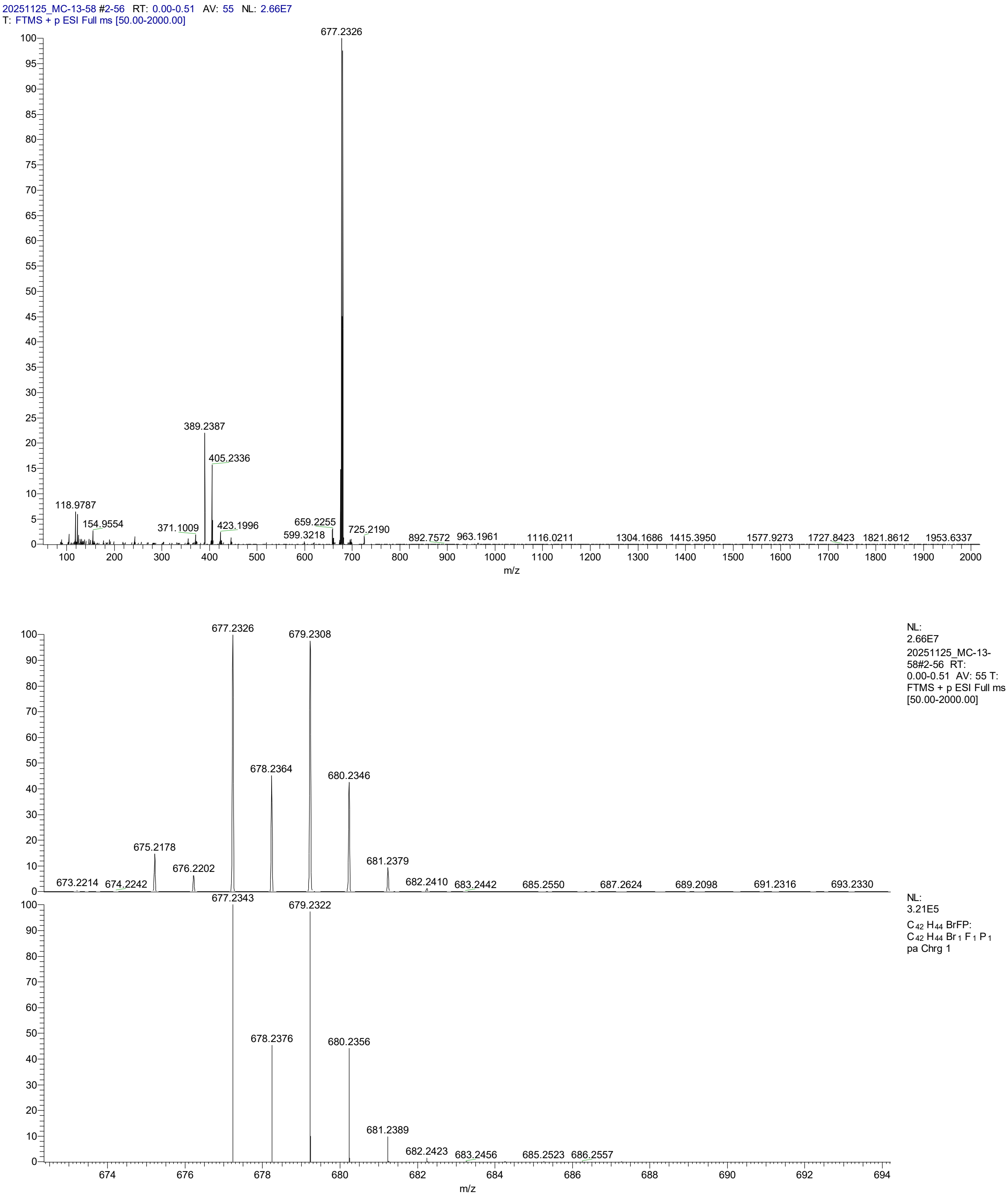

Figure S199: HRMS(ESI+) Spectra for **5c**.

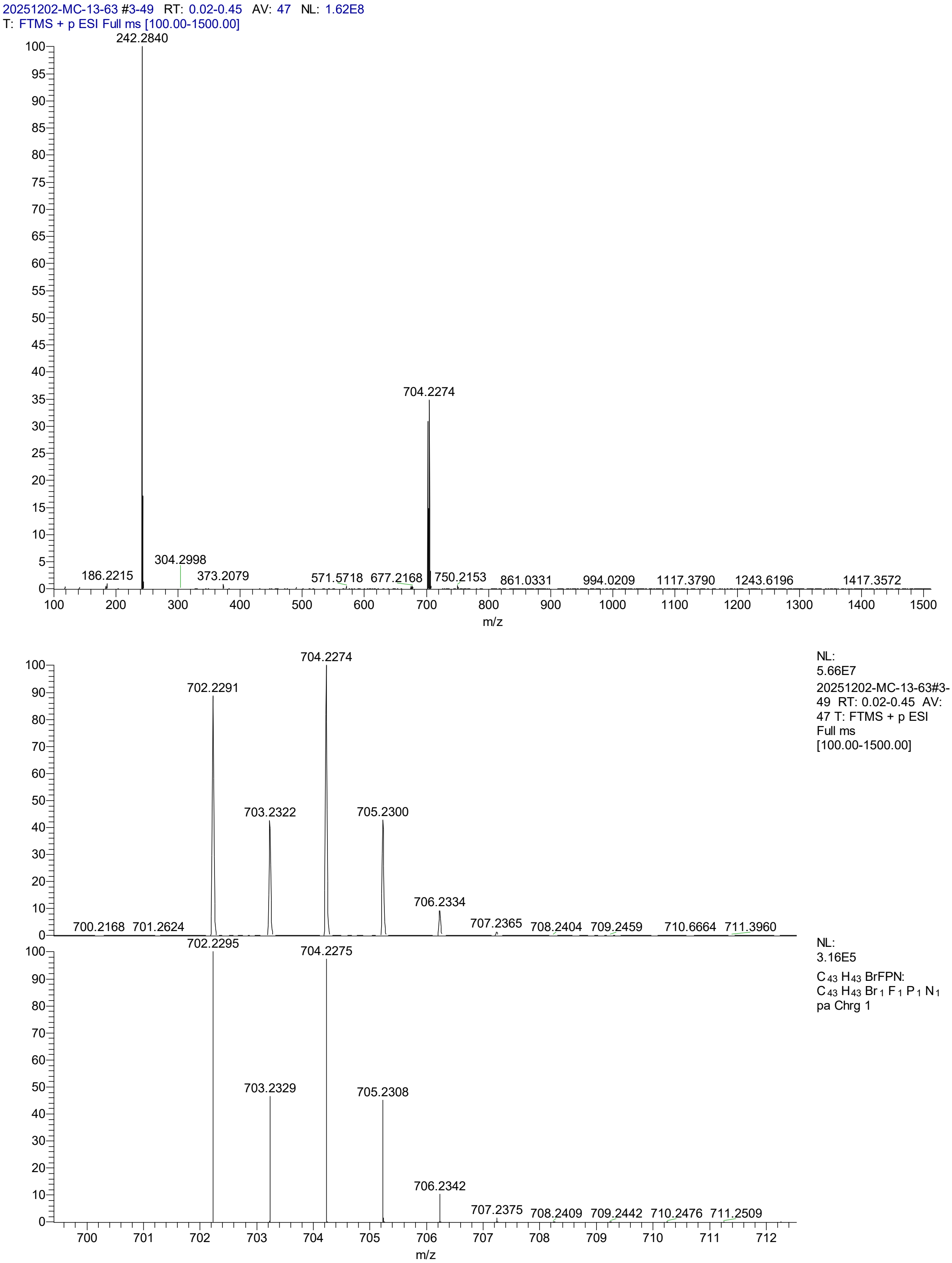

Figure S200: HRMS(ESI+) Spectra for **5d**.

Computational details

To gain insight into the mechanism of isomerisation of compounds **2** to **3**, we performed DFT calculations with the *Gaussian 16* suite of programs (Revision B.01).[11] The calculations were performed using B3LYP functional with Pople's 6-31G(d,p) basis set on all atoms (C, N, F, P, Br, and H).[12] The global solvation effects were taken into account by computing the solvation free energies employing SMD continuum solvation model (DCM; ε = 8.93).[13] Geometry optimisations and transition state locations were performed with PCM solvation method.

Reactivity descriptors were calculated by single point calculations on the optimised geometries obtained from the B3LYP functional and the 6-31G(d,p) basis sets as described above. Global and local Fukui reactivity descriptors (f^+, f^- and $f(0)$) were evaluated using NBO populations with the assistance of the UKA-FUKUI program (*version 2*).[14] Specifically, $f^+(k)$ and $f^-(k)$ are the condensed Fukui functions for nucleophilic and electrophilic attack on atom *k*, respectively and hence correspond to the electrophilicity and nucleophilicity of atom *k*. f^0 is represents the condensed Fukui function for radical attack at atom *k*.

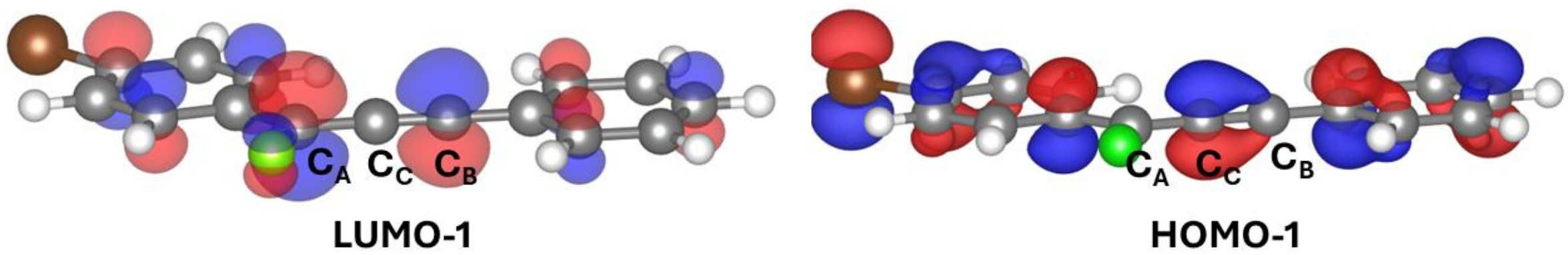

Fukui indices:

	f^+	f^-	f^0
**C_A**	**0.1839**	**-0.0144**	**0.0848**
**C_B**	**0.1747**	**-0.0064**	**0.0842**
**C_C**	**-0.0071**	**0.1296**	**0.0613**

Figure S201: Isosurfaces (cutoff = 0.05) of HOMO and LUMO for **Int_{1a}**. The table shows Fukui reactivity parameters obtained from NPA population analyses, where $f^+(k)$ = electrophilicity of atom k, $f^-(k)$ = nucleophilicty of atom k and $f^0(k)$ = radical reactivity of atom k.

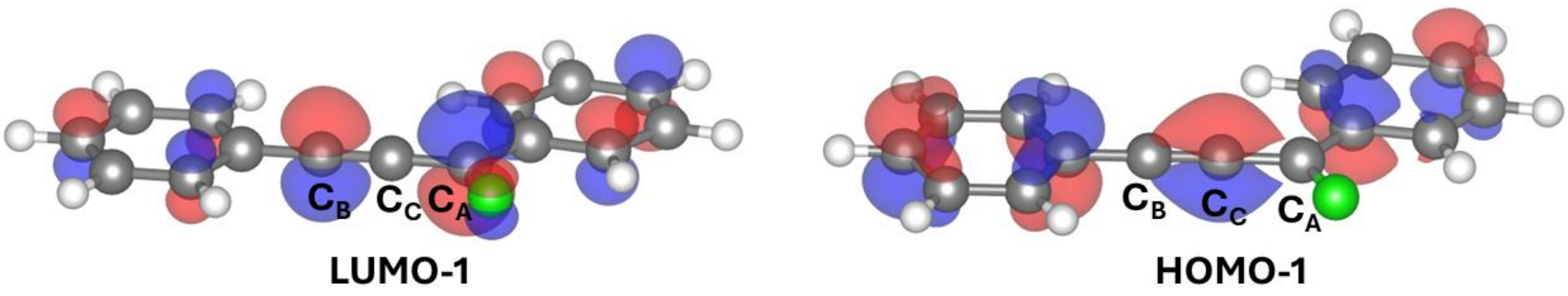

Fukui indices:

	f^+	f^-	f^0
C_A	**0.1891**	**-0.0150**	**0.0870**
C_B	**0.1781**	**-0.0131**	**0.0842**
C_C	**-0.0086**	**0.1447**	**0.0825**

Figure S202: Isosurfaces (cutoff = 0.05) of HOMO and LUMO for **Int$_{1c}$**. The table shows Fukui reactivity parameters obtained from NPA population analyses, where $f^+(k)$ = electrophilicity of atom k, $f^-(k)$ = nucleophilicty of atom k and $f^0(k)$ = radical reactivity of atom k.

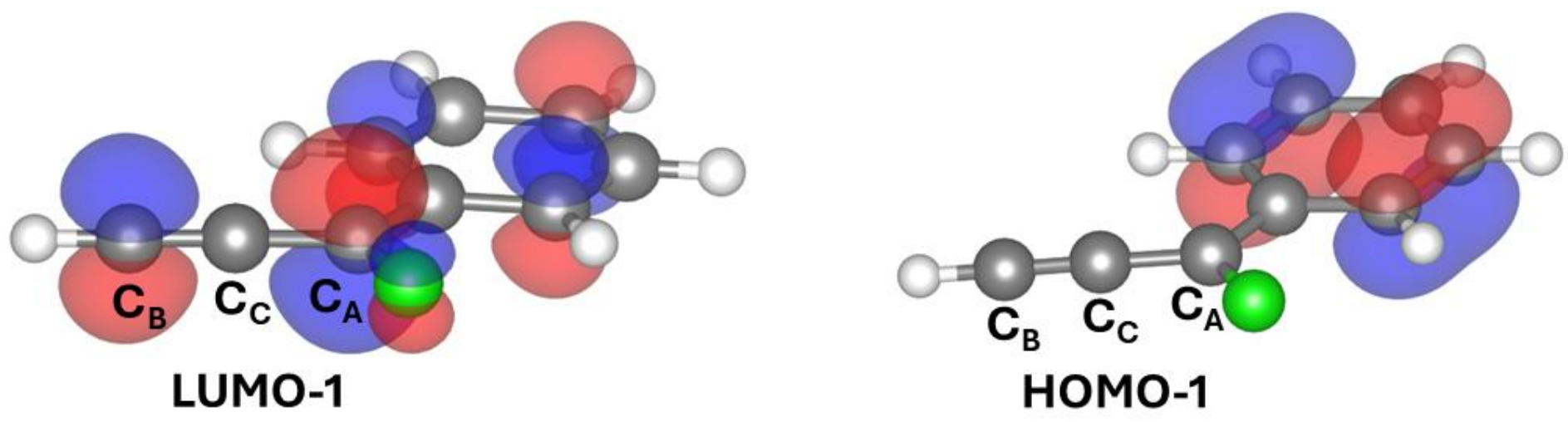

Fukui indices:

	f^+	f^-	f^0
C_A	**0.2269**	**-0.0252**	**0.1260**
C_B	**0.2295**	**0.1752**	**0.2024**
C_C	**-0.0216**	**0.0638**	**0.0211**

Figure S203: Isosurfaces (cutoff = 0.05) of HOMO and LUMO for **Int$_{1f'}$**. The table shows Fukui reactivity parameters obtained from NPA population analyses, where $f^+(k)$ = electrophilicity of atom k, $f^-(k)$ = nucleophilicty of atom k and $f^0(k)$ = radical reactivity of atom k.

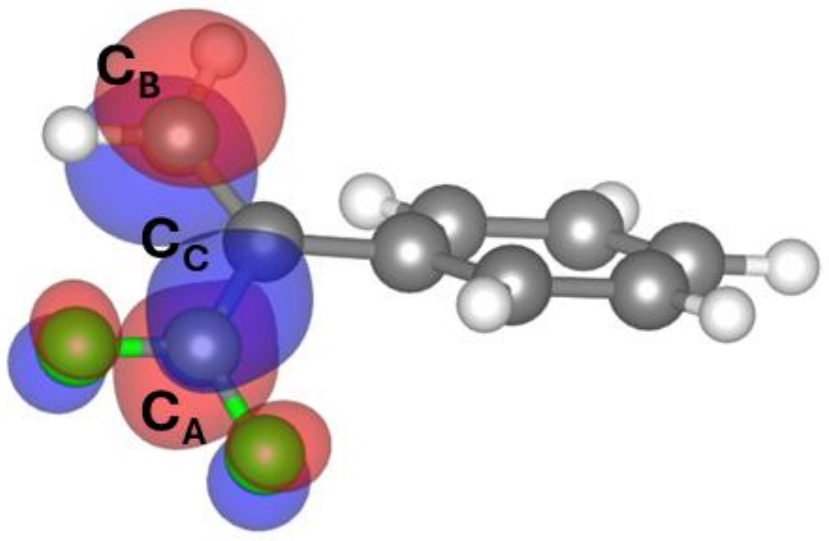

LUMO-1

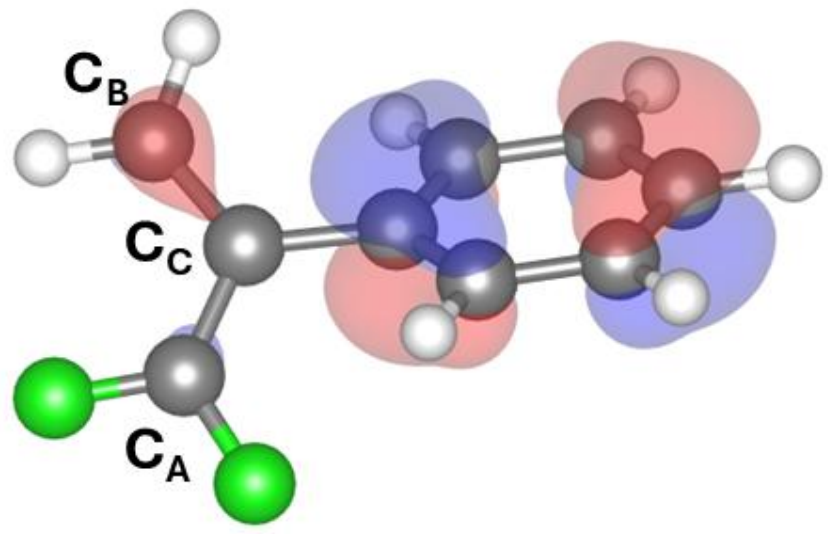

HOMO-1

Fukui indices:

	f^+	f^-	f^0
**C_A**	**0.2829**	**0.0265**	**0.1547**
**C_B**	**0.3520**	**0.0658**	**0.2089**
**C_C**	**0.0076**	**-0.0374**	**0.0149**

Figure S204: Isosurfaces (cutoff = 0.05) of HOMO and LUMO for **Int$_4$**. The table shows Fukui reactivity parameters obtained from NPA population analyses, where $f^+(k)$ = electrophilicity of atom k, $f^-(k)$ = nucleophilicty of atom k and $f^0(k)$ = radical reactivity of atom k.

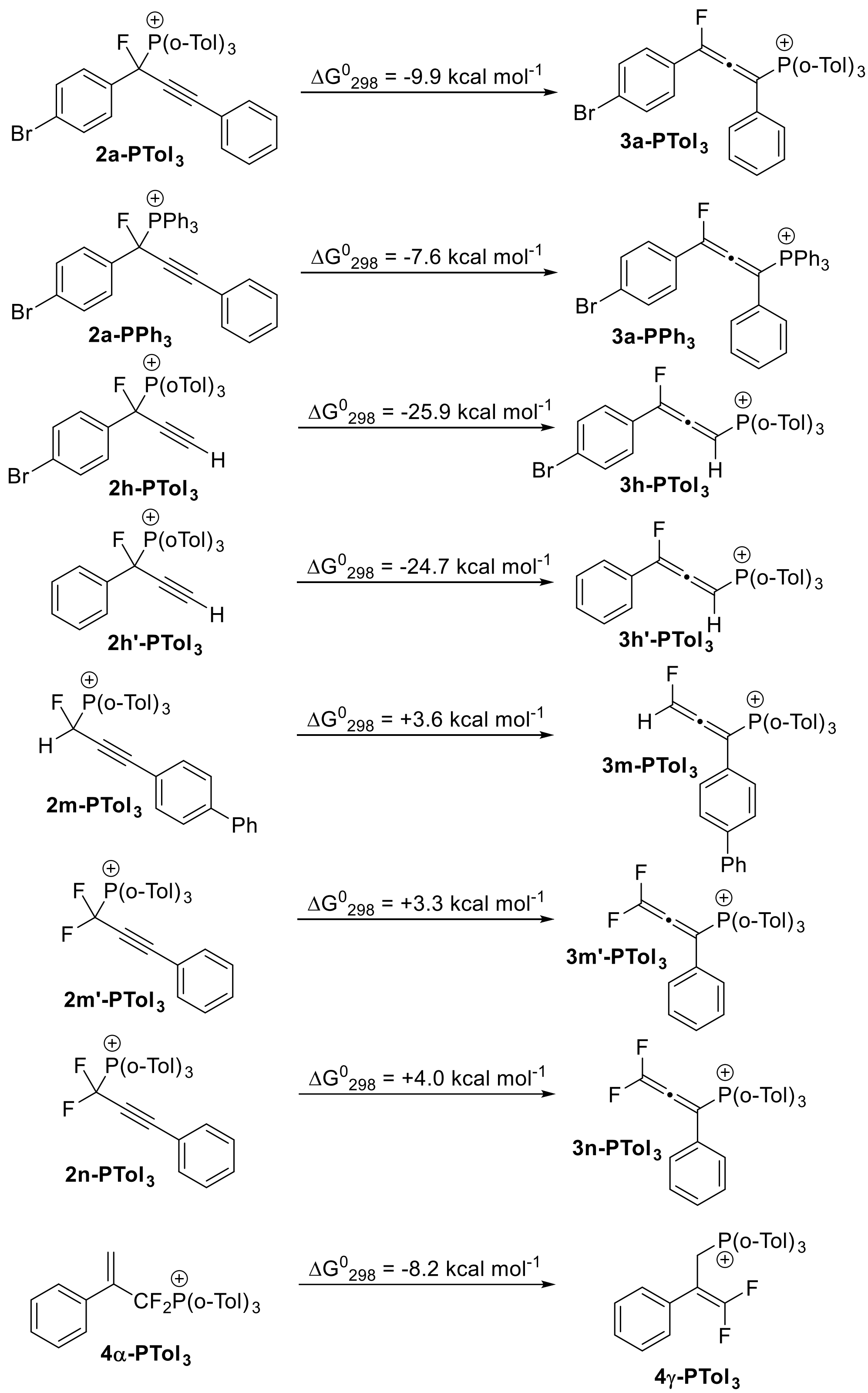

Figure S205: Calculated Gibbs free energy differences between isomeric alkyne and allene forms of selected P(*o*-Tol)$_3$ adducts.

Cartesian coordinates of the reported structures

Cartesian coordinates of the optimized geometries are given below in standard XYZ format (units are in Å).

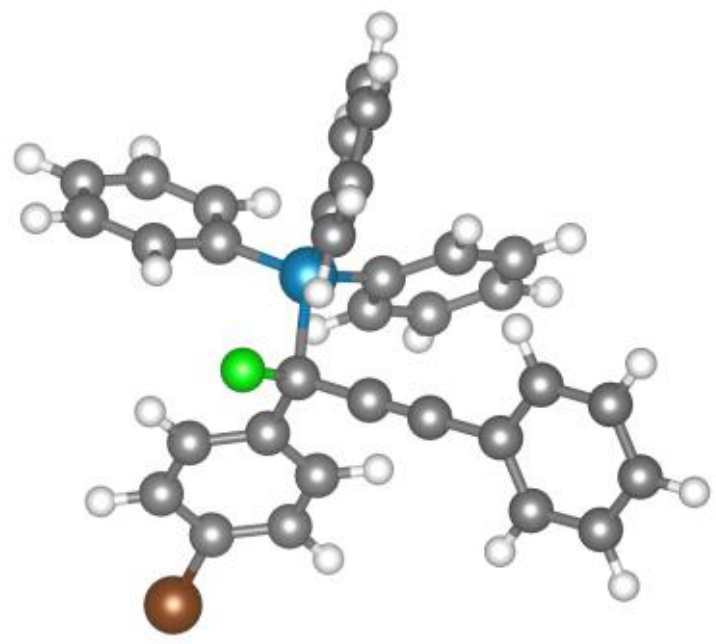

2a-PPh3

1	C	-0.09624	-0.09002	-1.05419
2	C	-1.10468	-1.11954	-0.91147
3	C	-1.96786	-1.96506	-0.78667
4	F	-0.17433	0.479859	-2.34955
5	C	1.323765	-0.58705	-0.8319
6	C	2.385723	-0.00186	-1.53501
7	C	1.582366	-1.63939	0.05634
8	C	3.689595	-0.45739	-1.35039
9	H	2.201819	0.797023	-2.24215
10	C	2.884253	-2.0968	0.251819
11	H	0.769037	-2.114	0.593467
12	C	3.925459	-1.49825	-0.45445
13	H	4.505623	-0.00683	-1.90349
14	H	3.076349	-2.91315	0.938476
15	C	-2.98292	-2.95561	-0.63728
16	C	-2.63862	-4.31956	-0.55599
17	C	-4.33748	-2.57242	-0.56855
18	C	-3.63641	-5.2787	-0.40611
19	H	-1.59515	-4.61203	-0.61178
20	C	-5.32577	-3.54138	-0.42025
21	H	-4.59946	-1.5214	-0.63393
22	C	-4.97824	-4.8929	-0.33808
23	H	-3.36773	-6.32869	-0.3434
24	H	-6.36825	-3.24274	-0.36839
25	H	-5.75278	-5.64508	-0.22192
26	C	-0.63466	0.726885	1.792105
27	C	0.556113	0.450175	2.490395
28	C	-1.87463	0.409249	2.374132
29	C	0.497779	-0.11618	3.762347
30	H	1.522126	0.672305	2.052308

31	C	-1.91857	-0.15888	3.646164
32	H	-2.80009	0.602721	1.84469
33	C	-0.73638	-0.41829	4.34161
34	H	1.419168	-0.32115	4.298055
35	H	-2.87912	-0.39741	4.091444
36	H	-0.77596	-0.85734	5.333796
37	C	0.611072	2.750062	-0.06384
38	C	1.17894	3.370689	1.060372
39	C	0.8664	3.261498	-1.35062
40	C	2.015272	4.474999	0.89269
41	H	0.969158	3.011398	2.060492
42	C	1.706749	4.361177	-1.50365
43	H	0.415503	2.807116	-2.22511
44	C	2.284299	4.96681	-0.38463
45	H	2.451643	4.949993	1.765614
46	H	1.904823	4.747431	-2.49845
47	H	2.936477	5.82582	-0.50901
48	C	-2.21476	2.020907	-0.37909
49	C	-2.77878	3.017392	0.439525
50	C	-2.88159	1.612793	-1.54465
51	C	-4.00843	3.578226	0.102945
52	H	-2.26684	3.355074	1.335224
53	C	-4.11228	2.18466	-1.87081
54	H	-2.45206	0.871151	-2.2043
55	C	-4.6776	3.160837	-1.05012
56	H	-4.44021	4.342517	0.741228
57	H	-4.62387	1.865258	-2.77324
58	H	-5.63514	3.601373	-1.31057
59	P	-0.57857	1.384552	0.106103
60	BR	5.714888	-2.12568	-0.19802

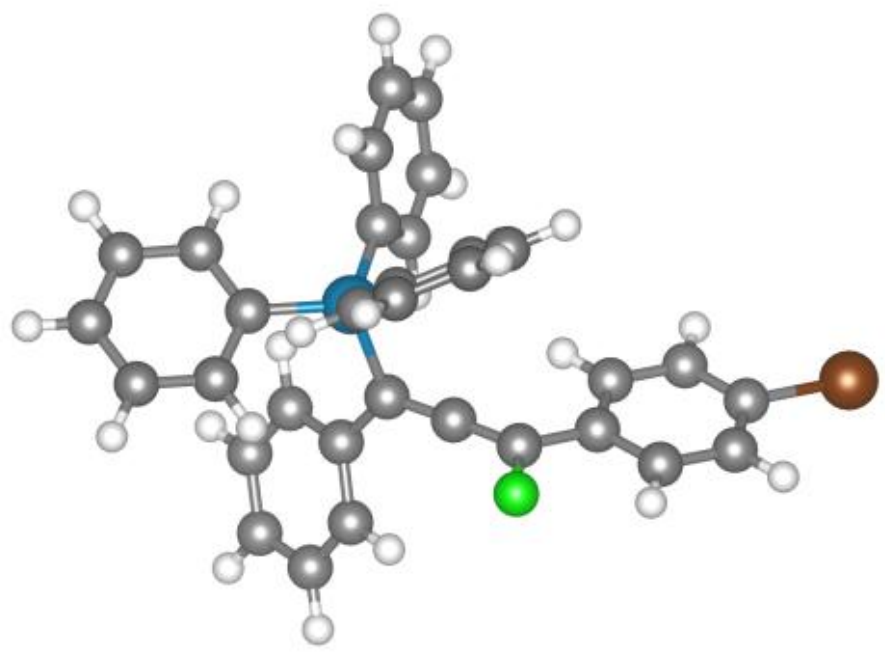

3a-PPh$_3$

1	C	0.109712	0.833917	-0.97661
2	C	-1.04563	0.995453	-0.36954
3	C	-1.64467	2.338664	-0.10614
4	C	-1.30926	3.397916	-0.97025
5	C	-2.5171	2.591128	0.965243
6	C	-1.82079	4.674688	-0.75794
7	H	-0.653	3.208985	-1.81402

8	C	-3.0268	3.872995	1.17395
9	H	-2.79706	1.800078	1.652164
10	C	-2.68143	4.917814	0.316207
11	H	-1.5533	5.478908	-1.43663
12	H	-3.69542	4.051249	2.010527
13	H	-3.08411	5.913057	0.478322
14	C	1.251589	0.706335	-1.61891
15	F	1.204946	0.68861	-2.98144
16	C	2.604708	0.579112	-1.06436
17	C	2.821716	0.563213	0.323878
18	C	3.702536	0.473418	-1.93204
19	C	4.108049	0.442763	0.836829
20	H	1.983822	0.645249	1.008725
21	C	4.994994	0.352476	-1.42524
22	H	3.55028	0.484851	-3.0046
23	C	5.184699	0.337924	-0.04566
24	H	4.269651	0.431186	1.908595
25	H	5.838705	0.271751	-2.10117
26	P	-1.92275	-0.58066	0.085615
27	C	-3.70774	-0.34202	-0.14898
28	C	-4.63099	-0.83599	0.785155
29	C	-4.16053	0.33146	-1.29743
30	C	-5.99732	-0.66562	0.562055
31	H	-4.29594	-1.34518	1.681374
32	C	-5.52807	0.492733	-1.51055
33	H	-3.45526	0.734563	-2.01677
34	C	-6.4461	-0.00542	-0.5829
35	H	-6.70872	-1.04777	1.287311
36	H	-5.87377	1.013848	-2.39764
37	H	-7.51069	0.126315	-0.75078
38	C	-1.32097	-1.91133	-0.99607
39	C	-0.10498	-2.54763	-0.69017
40	C	-2.04203	-2.28933	-2.13927
41	C	0.382796	-3.54724	-1.52948
42	H	0.455638	-2.27384	0.196949
43	C	-1.54453	-3.29224	-2.97074
44	H	-2.98716	-1.81662	-2.37992
45	C	-0.33466	-3.91948	-2.66872
46	H	1.320968	-4.03749	-1.28936
47	H	-2.1077	-3.58463	-3.85133
48	H	0.04707	-4.70186	-3.31764
49	C	-1.52821	-1.04868	1.796519
50	C	-1.87509	-2.33936	2.236437
51	C	-0.87546	-0.16458	2.669889
52	C	-1.58726	-2.72629	3.543854
53	H	-2.35694	-3.04176	1.564396

54	C	-0.58728	-0.56523	3.974792
55	H	-0.59036	0.829052	2.342885
56	C	-0.94522	-1.84084	4.413066
57	H	-1.85874	-3.7223	3.879023
58	H	-0.08134	0.122052	4.645296
59	H	-0.71908	-2.14801	5.429548
60	BR	6.956197	0.170179	0.658347

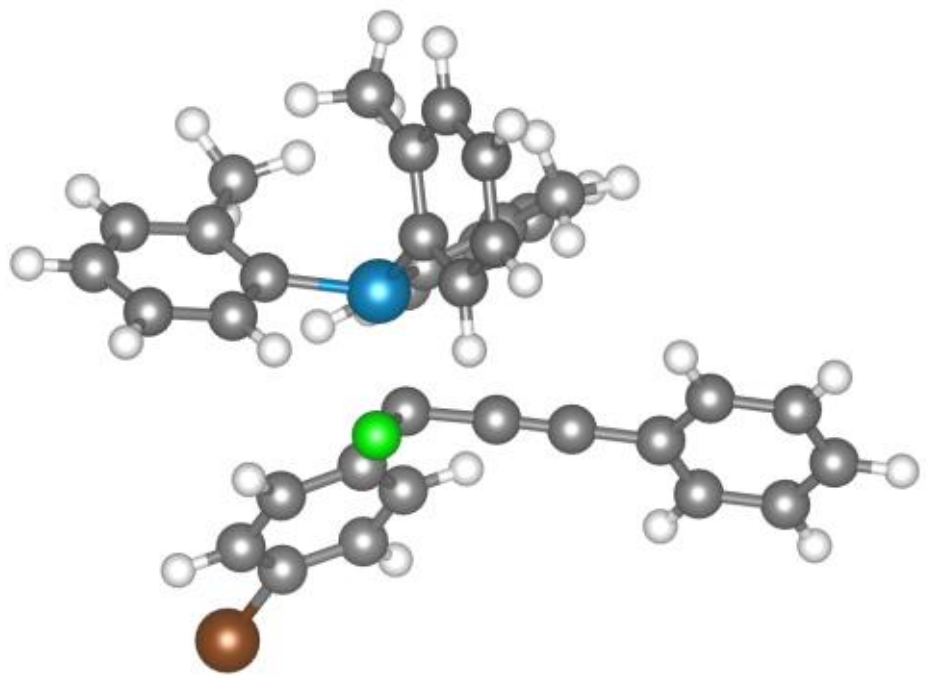

2a-PTol$_3$

1	C	0.033182	0.265768	-1.0944
2	C	0.969981	1.355912	-0.89635
3	C	1.744791	2.284348	-0.78187
4	F	0.145038	-0.19898	-2.42431
5	C	-1.41483	0.67921	-0.86828
6	C	-2.44323	0.020519	-1.55689
7	C	-1.73574	1.742168	-0.01387
8	C	-3.7713	0.405166	-1.38404
9	H	-2.21622	-0.78582	-2.24263
10	C	-3.06073	2.13344	0.167441
11	H	-0.95269	2.276271	0.511094
12	C	-4.06666	1.456188	-0.51793
13	H	-4.55935	-0.10638	-1.92468
14	H	-3.29783	2.958483	0.829071
15	C	2.656249	3.373633	-0.64461
16	C	2.188476	4.644904	-0.25616
17	C	4.029179	3.185648	-0.9017
18	C	3.08297	5.703429	-0.12409
19	H	1.130581	4.790001	-0.06299
20	C	4.912992	4.252986	-0.7689
21	H	4.388073	2.207617	-1.20525
22	C	4.443579	5.510691	-0.37961
23	H	2.718027	6.680715	0.1766
24	H	5.969547	4.103783	-0.96935
25	H	5.137249	6.339829	-0.27703
26	C	0.514697	-0.50919	1.708046
27	C	-0.79649	-0.36522	2.206491
28	C	1.613118	0.016815	2.43965
29	C	-1.04048	0.250718	3.429004

30	H	-1.64004	-0.74377	1.642011
31	C	1.328445	0.623661	3.672777
32	C	0.033023	0.739279	4.170527
33	H	-2.05874	0.344205	3.791925
34	H	2.157527	1.023231	4.249169
35	H	-0.13516	1.219672	5.129755
36	C	-0.48833	-2.68918	-0.27235
37	C	-1.12984	-3.53222	0.672369
38	C	-0.57318	-2.99746	-1.65064
39	C	-1.88333	-4.60689	0.165439
40	C	-1.32515	-4.07003	-2.11129
41	H	-0.04901	-2.39015	-2.37766
42	C	-1.99868	-4.87463	-1.19305
43	H	-2.3777	-5.25894	0.879875
44	H	-1.37374	-4.27515	-3.17577
45	H	-2.59117	-5.71982	-1.53006
46	C	2.279707	-1.77456	-0.52209
47	C	2.9468	-2.84686	0.126277
48	C	2.859062	-1.16509	-1.64967
49	C	4.192931	-3.23889	-0.38037
50	C	4.097399	-1.58501	-2.12974
51	H	2.351037	-0.36941	-2.17336
52	C	4.769282	-2.62337	-1.48948
53	H	4.717347	-4.05037	0.115521
54	H	4.525227	-1.10004	-3.00126
55	H	5.73669	-2.95816	-1.85147
56	P	0.614055	-1.2427	0.037391
57	BR	-5.88856	1.98796	-0.27407
58	C	2.394909	-3.58938	1.317322
59	H	1.612555	-4.29355	1.01448
60	H	3.185945	-4.16644	1.801948
61	H	1.965258	-2.92196	2.068981
62	C	-1.06614	-3.42756	2.179287
63	H	-0.16087	-2.94994	2.545924
64	H	-1.92159	-2.86967	2.574689
65	H	-1.11454	-4.43187	2.608989
66	C	3.056931	-0.01941	2.000916
67	H	3.487028	-1.02004	2.107583
68	H	3.183298	0.284815	0.961488
69	H	3.645221	0.658358	2.623712

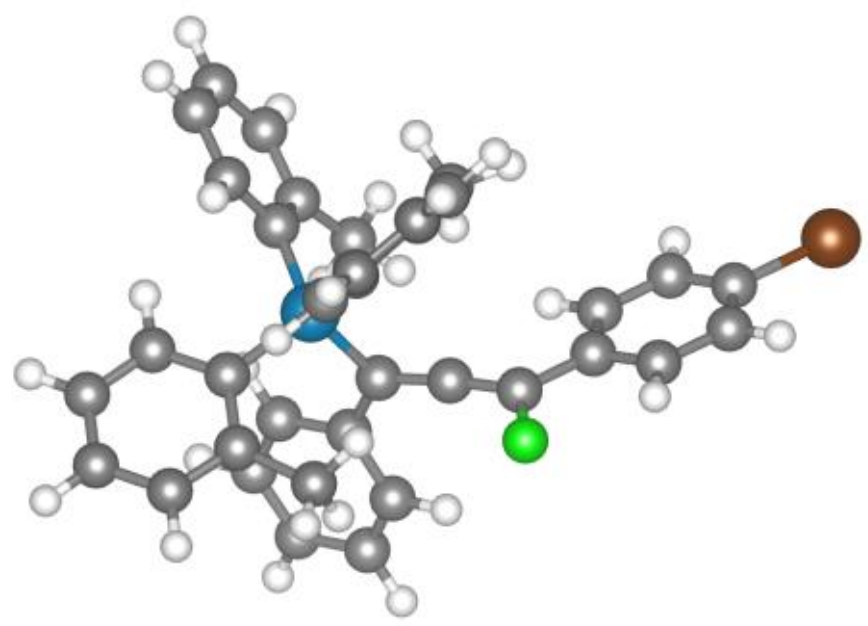

3a-PTol₃

1	C	0.357003	0.917793	-0.63252
2	C	-0.80953	1.042326	-0.03923
3	C	-1.37485	2.360484	0.385388
4	C	-0.79164	3.540053	-0.11568
5	C	-2.47026	2.473771	1.256361
6	C	-1.28348	4.788581	0.252881
7	H	0.047544	3.474299	-0.80039
8	C	-2.96163	3.727172	1.622952
9	H	-2.9472	1.591517	1.66574
10	C	-2.37176	4.888427	1.124393
11	H	-0.81939	5.685107	-0.1469
12	H	-3.8077	3.790082	2.300392
13	H	-2.75732	5.862826	1.408586
14	C	1.46944	0.850965	-1.33268
15	F	1.35014	0.964451	-2.68934
16	C	2.8487	0.667684	-0.86868
17	C	3.1675	0.744947	0.497635
18	C	3.869441	0.419957	-1.79936
19	C	4.477156	0.567474	0.929606
20	H	2.39263	0.957444	1.226993
21	C	5.184759	0.244303	-1.37461
22	H	3.638919	0.363347	-2.85656
23	C	5.474898	0.317869	-0.01416
24	H	4.717539	0.631293	1.984701
25	H	5.96904	0.052999	-2.09797
26	P	-1.85706	-0.50106	0.02079
27	C	-3.46791	0.006926	-0.70878
28	C	-4.63844	-0.2405	0.031835
29	C	-3.55111	0.665658	-1.96638
30	C	-5.88907	0.128494	-0.45397
31	H	-4.58115	-0.71813	1.001108
32	C	-4.82969	1.01272	-2.42846
33	C	-5.9846	0.753501	-1.69526
34	H	-6.77659	-0.07573	0.136171
35	H	-4.90873	1.506517	-3.39266
36	H	-6.95277	1.042377	-2.09299
37	C	-1.10134	-1.82813	-0.9947

38	C	0.091889	-2.48711	-0.60203
39	C	-1.78882	-2.24414	-2.14877
40	C	0.569106	-3.51111	-1.43175
41	C	-1.2948	-3.27557	-2.94314
42	H	-2.72239	-1.77273	-2.42892
43	C	-0.10356	-3.9041	-2.587
44	H	1.489883	-4.01398	-1.15031
45	H	-1.84024	-3.57923	-3.83078
46	H	0.29953	-4.70444	-3.20024
47	C	-2.12501	-1.26005	1.673866
48	C	-2.89651	-2.44105	1.592997
49	C	-1.60266	-0.843	2.922182
50	C	-3.20704	-3.18006	2.72742
51	H	-3.26113	-2.78938	0.632139
52	C	-1.93674	-1.61633	4.048175
53	C	-2.73053	-2.75506	3.967857
54	H	-3.80655	-4.08005	2.638141
55	H	-1.54088	-1.30961	5.012032
56	H	-2.95922	-3.31934	4.866924
57	BR	7.27998	0.079883	0.575093
58	C	-2.37621	1.063775	-2.83176
59	H	-2.70361	1.16403	-3.86958
60	H	-1.9845	2.03826	-2.51889
61	H	-1.54632	0.357293	-2.81479
62	C	0.84769	-2.20371	0.673044
63	H	0.842166	-1.15095	0.956503
64	H	0.418641	-2.77313	1.505471
65	H	1.890126	-2.5132	0.567908
66	C	-0.69141	0.337591	3.155834
67	H	-0.0291	0.539273	2.314395
68	H	-1.26142	1.251107	3.353068
69	H	-0.06848	0.148735	4.033671

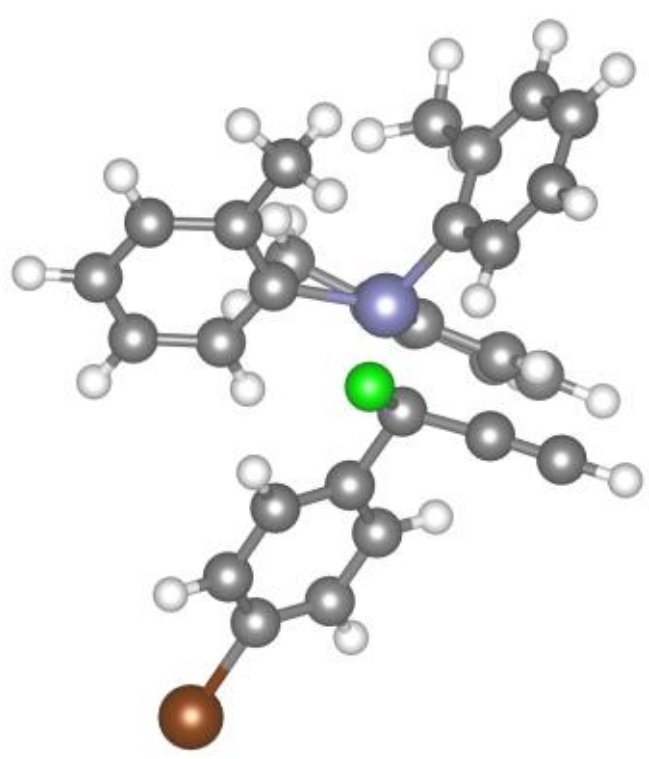

2h-PTol$_3$

1	C	0	-0.0841	-0.05358	-1.57161
2	C	0	1.39356	-0.07702	-1.20447
3	C	0	2.015727	-1.26939	-0.81184
4	C	0	2.152542	1.095193	-1.30695

5	C	0	3.373875	-1.29014	-0.5038
6	H	0	1.450831	-2.19253	-0.75056
7	C	0	3.516158	1.081743	-1.01481
8	H	0	1.689797	2.021084	-1.62306
9	C	0	4.10973	-0.11083	-0.61005
10	H	0	3.849905	-2.21462	-0.19915
11	H	0	4.099909	1.990468	-1.10384
12	C	0	-0.40251	-1.12077	-2.50809
13	C	0	-0.61011	-1.98582	-3.32519
14	H	0	-0.80164	-2.74753	-4.05199
15	F	0	-0.37024	1.181765	-2.19151
16	P	0	-1.31648	0.049461	-0.03018
17	C	0	-1.05237	-1.43427	1.019307
18	C	0	-0.82086	-1.48813	2.418964
19	C	0	-1.17401	-2.63484	0.284962
20	C	0	-0.64715	-2.7614	2.988978
21	C	0	-1.00675	-3.87601	0.886703
22	H	0	-1.41071	-2.60652	-0.77138
23	C	0	-0.72215	-3.93694	2.250067
24	H	0	-0.46706	-2.81765	4.058647
25	H	0	-1.10473	-4.78024	0.294961
26	H	0	-0.58432	-4.89482	2.742425
27	C	0	-0.82115	1.627448	0.739971
28	C	0	-1.56805	2.835458	0.7069
29	C	0	0.461404	1.607271	1.326802
30	C	0	-0.98043	3.965076	1.297127
31	C	0	1.014434	2.750028	1.893581
32	H	0	1.032376	0.68731	1.359359
33	C	0	0.284237	3.936235	1.880046
34	H	0	-1.5409	4.894938	1.290904
35	H	0	2.001895	2.706314	2.341024
36	H	0	0.695084	4.839383	2.321061
37	C	0	-3.0176	-0.01425	-0.71238
38	C	0	-4.13475	-0.31413	0.11225
39	C	0	-3.20703	0.239258	-2.08445
40	C	0	-5.39169	-0.39575	-0.50322
41	C	0	-4.47106	0.154409	-2.66015
42	H	0	-2.37732	0.52636	-2.71333
43	C	0	-5.56713	-0.17784	-1.86747
44	H	0	-6.25302	-0.6242	0.117711
45	H	0	-4.59076	0.353106	-3.72025
46	H	0	-6.55926	-0.24977	-2.30281
47	C	0	-2.93678	3.006159	0.093083
48	H	0	-2.93117	2.799752	-0.98049
49	H	0	-3.68128	2.348145	0.548208
50	H	0	-3.27361	4.03577	0.231674

51	C	0	-4.07014	-0.50974	1.607158
52	H	0	-3.73607	-1.51779	1.870991
53	H	0	-3.39685	0.201986	2.091638
54	H	0	-5.06321	-0.36837	2.040623
55	C	0	-0.79183	-0.32184	3.380686
56	H	0	0.224853	0.059032	3.521417
57	H	0	-1.41512	0.51519	3.069258
58	H	0	-1.14565	-0.65819	4.358794
59	BR	0	5.980094	-0.13823	-0.20224

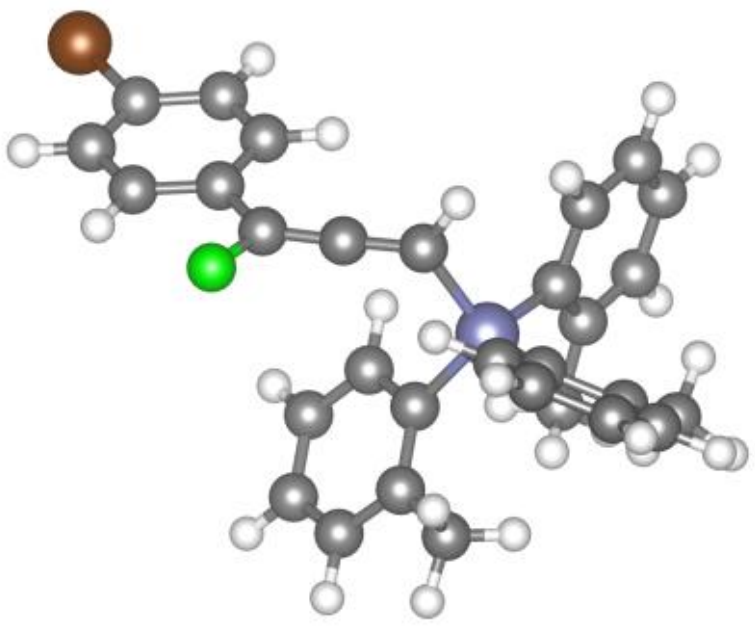

3h-PTol$_3$

1	C	0	1.432684	-1.67134	-1.17758
2	C	0	2.762719	-1.10604	-0.92523
3	C	0	3.837344	-1.96097	-0.63656
4	C	0	2.979902	0.281878	-0.96422
5	C	0	5.106336	-1.44209	-0.38451
6	H	0	3.685282	-3.03346	-0.60568
7	C	0	4.242136	0.807971	-0.71211
8	H	0	2.162604	0.955789	-1.20184
9	C	0	5.294275	-0.06264	-0.42295
10	H	0	5.932277	-2.10744	-0.16119
11	H	0	4.404585	1.878819	-0.74665
12	F	0	1.399623	-3.03005	-1.24168
13	C	0	0.296264	-1.02342	-1.33356
14	C	0	-0.81752	-0.37348	-1.52171
15	H	0	-1.12095	-0.06335	-2.52251
16	P	0	-2.02929	0.05613	-0.2012
17	C	0	-2.01477	1.889678	-0.13225
18	C	0	-2.11418	2.673516	1.04469
19	C	0	-1.90111	2.51759	-1.39005
20	C	0	-2.06355	4.068662	0.89403
21	C	0	-1.86445	3.902893	-1.50213
22	H	0	-1.84967	1.930061	-2.29936
23	C	0	-1.93665	4.682604	-0.34846
24	H	0	-2.13873	4.682528	1.787041
25	H	0	-1.77895	4.362771	-2.48123
26	H	0	-1.9057	5.76591	-0.41498
27	C	0	-1.38834	-0.70295	1.326503
28	C	0	-2.02809	-1.6983	2.105273

29	C	0	-0.10696	-0.23482	1.681559
30	C	0	-1.32641	-2.18439	3.219916
31	C	0	0.558326	-0.73937	2.793637
32	H	0	0.370998	0.542842	1.094584
33	C	0	-0.05823	-1.72347	3.564936
34	H	0	-1.80059	-2.94598	3.83176
35	H	0	1.543375	-0.3642	3.051096
36	H	0	0.443466	-2.12993	4.43796
37	C	0	-3.60087	-0.60547	-0.86205
38	C	0	-3.47153	-1.73941	-1.68847
39	C	0	-4.87973	-0.04769	-0.61376
40	C	0	-4.58625	-2.32911	-2.27618
41	H	0	-2.49503	-2.17347	-1.87238
42	C	0	-5.98015	-0.66179	-1.22958
43	C	0	-5.84731	-1.78113	-2.04846
44	H	0	-4.46458	-3.20436	-2.90595
45	H	0	-6.96727	-0.24627	-1.05019
46	H	0	-6.72829	-2.22465	-2.50253
47	C	0	-5.13804	1.145599	0.271915
48	H	0	-4.61815	1.072922	1.23025
49	H	0	-4.82101	2.077933	-0.20601
50	H	0	-6.20711	1.228213	0.48172
51	C	0	-2.28037	2.139932	2.448691
52	H	0	-2.85195	1.212798	2.49457
53	H	0	-1.30933	1.94384	2.916125
54	H	0	-2.79391	2.883476	3.063496
55	C	0	-3.4013	-2.26446	1.835922
56	H	0	-4.16635	-1.48632	1.772958
57	H	0	-3.43455	-2.83035	0.900741
58	H	0	-3.68428	-2.94237	2.64434
59	BR	0	7.034611	0.659851	-0.07562

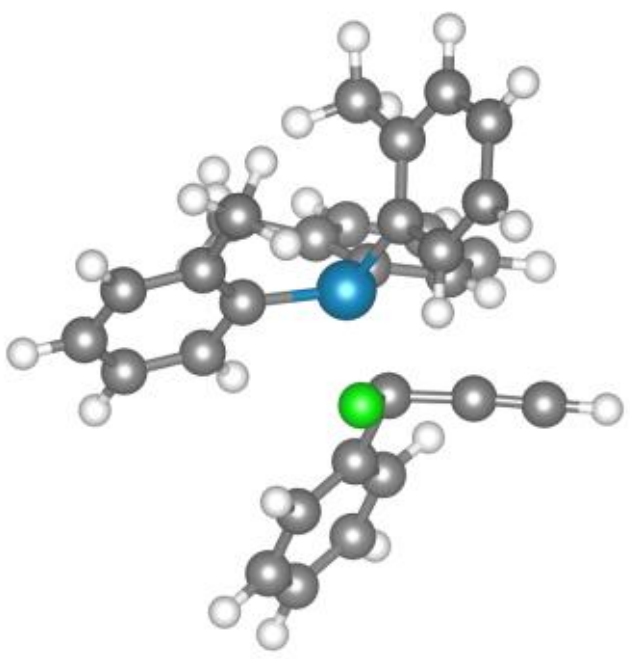

2h’-PTol$_3$

1	C	0.689717	-0.09301	-1.64939
2	C	2.183763	-0.2447	-1.38967
3	C	2.728564	-1.48906	-1.03989
4	C	3.02844	0.860588	-1.55699
5	C	4.101467	-1.61677	-0.83644
6	H	2.08543	-2.35565	-0.93443

7	C	4.40382	0.72017	-1.36624
8	H	2.616532	1.820212	-1.84185
9	C	4.94291	-0.51334	-0.99957
10	H	4.513822	-2.58306	-0.56277
11	H	5.051832	1.580029	-1.50596
12	H	6.013259	-0.6181	-0.84957
13	C	0.213978	-1.10842	-2.57764
14	C	-0.13246	-1.93543	-3.38705
15	H	-0.44741	-2.66982	-4.09873
16	F	0.463494	1.175041	-2.22689
17	P	-0.42608	0.070527	-0.02692
18	C	-0.19604	-1.4423	0.987883
19	C	0.110744	-1.52711	2.371166
20	C	-0.4341	-2.62325	0.251406
21	C	0.238982	-2.81452	2.920369
22	C	-0.30966	-3.87939	0.832365
23	H	-0.72614	-2.56583	-0.7899
24	C	0.048915	-3.97404	2.176368
25	H	0.475576	-2.89415	3.97754
26	H	-0.49777	-4.76901	0.240215
27	H	0.155708	-4.94462	2.651257
28	C	0.217137	1.602725	0.725596
29	C	-0.45856	2.851658	0.777253
30	C	1.537957	1.495511	1.208134
31	C	0.24151	3.933102	1.334102
32	C	2.201531	2.591744	1.747753
33	H	2.049435	0.541382	1.181642
34	C	1.54539	3.818795	1.810493
35	H	-0.26133	4.893775	1.388924
36	H	3.216521	2.481002	2.114821
37	H	2.043544	4.687404	2.230604
38	C	-2.17061	0.125051	-0.59619
39	C	-3.24888	-0.15305	0.285117
40	C	-2.43117	0.438143	-1.94411
41	C	-4.54473	-0.13953	-0.24973
42	C	-3.7317	0.445726	-2.43975
43	H	-1.62784	0.70242	-2.61541
44	C	-4.79348	0.144303	-1.59032
45	H	-5.37638	-0.34814	0.416914
46	H	-3.90546	0.691013	-3.48246
47	H	-5.81343	0.144112	-1.96271
48	C	-1.8632	3.112153	0.288889
49	H	-1.96274	2.93582	-0.78528
50	H	-2.59967	2.481253	0.792534
51	H	-2.13012	4.153607	0.481109
52	C	-3.10381	-0.42463	1.76206

53	H	-2.77692	-1.45064	1.956605
54	H	-2.38909	0.249141	2.240769
55	H	-4.06761	-0.28631	2.257849
56	C	0.260949	-0.37222	3.334018
57	H	1.299311	-0.03255	3.399915
58	H	-0.34931	0.490095	3.06995
59	H	-0.03418	-0.70007	4.334228

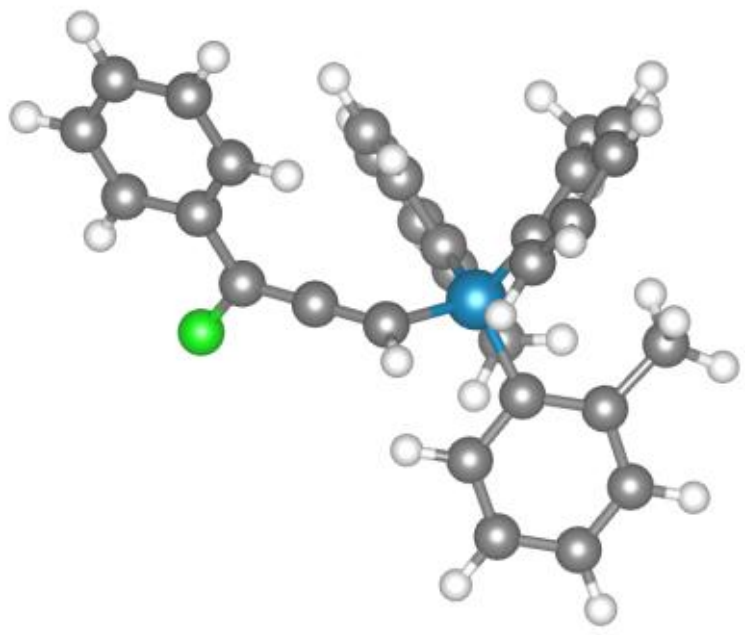

3h'-PTol$_3$

1	C	2.664108	-1.20265	-1.21068
2	C	3.89874	-0.44291	-0.9805
3	C	5.08908	-1.1266	-0.68605
4	C	3.905011	0.961678	-1.04783
5	C	6.264686	-0.41224	-0.45678
6	H	5.091177	-2.20924	-0.63609
7	C	5.082111	1.666087	-0.81435
8	H	2.991776	1.495888	-1.29227
9	C	6.265829	0.982236	-0.51781
10	H	7.180822	-0.94904	-0.22949
11	H	5.07849	2.750505	-0.86992
12	H	7.183302	1.53479	-0.33886
13	F	2.829818	-2.55261	-1.26751
14	C	1.441442	-0.73354	-1.35614
15	C	0.24251	-0.2545	-1.53499
16	H	-0.11782	-0.00345	-2.53345
17	P	-1.00238	0.002281	-0.20135
18	C	-1.28533	1.814211	-0.16505
19	C	-1.4613	2.605623	0.997908
20	C	-1.3307	2.414823	-1.44016
21	C	-1.63973	3.98637	0.813322
22	C	-1.52104	3.784295	-1.58546
23	H	-1.22997	1.813807	-2.33654
24	C	-1.66505	4.57629	-0.44699
25	H	-1.77397	4.606991	1.694549
26	H	-1.55508	4.222409	-2.5776
27	H	-1.81032	5.64847	-0.53848
28	C	-0.23029	-0.61253	1.330459
29	C	-0.70229	-1.67057	2.146042
30	C	0.977885	0.042676	1.643079

31	C	0.088578	-2.02663	3.249934
32	C	1.735342	-0.33657	2.74607
33	H	1.325336	0.868268	1.030018
34	C	1.28604	-1.38215	3.551165
35	H	-0.25702	-2.83356	3.889266
36	H	2.661856	0.18198	2.969857
37	H	1.86231	-1.69354	4.417239
38	C	-2.46013	-0.9125	-0.82065
39	C	-2.17033	-2.01886	-1.64289
40	C	-3.80506	-0.56396	-0.54284
41	C	-3.19098	-2.78408	-2.19896
42	H	-1.14114	-2.29222	-1.84731
43	C	-4.80853	-1.35089	-1.12615
44	H	-4.51759	-2.44206	-1.94259
45	H	-2.94659	-3.63505	-2.82642
46	H	-5.84484	-1.09706	-0.92249
47	H	-5.32684	-3.02472	-2.37235
48	C	-4.22242	0.580003	0.346844
49	H	-3.66014	0.600439	1.283461
50	H	-4.07905	1.546899	-0.14584
51	H	-5.2819	0.489941	0.59751
52	C	-1.48916	2.094925	2.420124
53	H	-1.91397	1.09522	2.513087
54	H	-0.48251	2.05969	2.850183
55	H	-2.08527	2.771417	3.037932
56	C	-1.99303	-2.42436	1.931944
57	H	-2.85542	-1.75402	1.882517
58	H	-1.97986	-3.00916	1.007929
59	H	-2.15647	-3.11622	2.761126

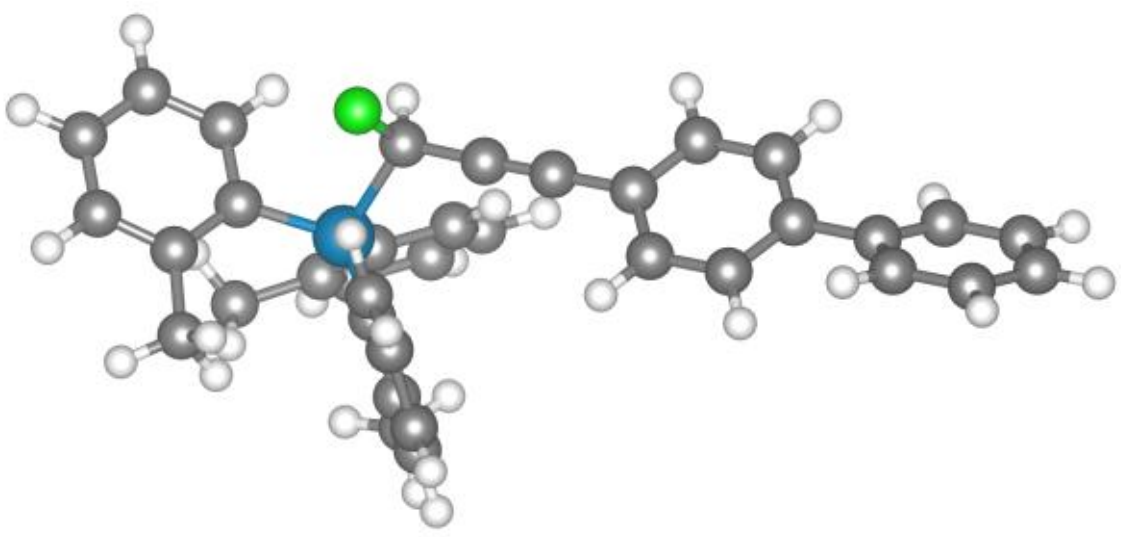

2l-PTol₃

1	C	0	1.276136	-0.05913	-1.69764
2	C	0	-0.12854	-0.04899	-1.35982
3	C	0	-1.32245	-0.07514	-1.1363
4	C	0	-2.73185	-0.04533	-0.91489
5	C	0	-3.30962	-0.86222	0.07564
6	C	0	-3.56148	0.793624	-1.68349
7	C	0	-4.68483	-0.83753	0.287705
8	H	0	-2.6744	-1.51	0.670444
9	C	0	-4.93583	0.811605	-1.4615

10	H	0	-3.1218	1.423099	-2.45026
11	C	0	-5.51672	-0.00249	-0.47541
12	H	0	-5.12104	-1.4715	1.052896
13	H	0	-5.56767	1.460443	-2.05936
14	C	0	1.713459	-1.03196	1.074302
15	C	0	1.233584	-2.25846	0.570762
16	C	0	1.590664	-0.72317	2.452389
17	C	0	0.629958	-3.18766	1.411416
18	H	0	1.338578	-2.49717	-0.48189
19	C	0	0.965368	-1.68023	3.266171
20	C	0	0.491563	-2.89125	2.767002
21	H	0	0.274323	-4.12975	1.007262
22	H	0	0.860043	-1.46331	4.324867
23	H	0	0.021083	-3.60329	3.438319
24	C	0	4.039469	-0.60244	-0.87684
25	C	0	4.877981	-1.55715	-0.25126
26	C	0	4.391071	-0.0613	-2.12965
27	C	0	6.036968	-1.94281	-0.94284
28	C	0	5.551315	-0.46068	-2.7828
29	H	0	3.769762	0.692332	-2.60147
30	C	0	6.374227	-1.41557	-2.18659
31	H	0	6.692554	-2.67335	-0.47822
32	H	0	5.805009	-0.02914	-3.74547
33	H	0	7.282373	-1.74347	-2.68332
34	C	0	2.554138	1.813286	0.215882
35	C	0	3.734995	2.478419	0.63115
36	C	0	1.344922	2.526117	0.090483
37	C	0	3.643624	3.856573	0.875039
38	C	0	1.290225	3.892444	0.346843
39	H	0	0.433205	2.017117	-0.20088
40	C	0	2.45031	4.561358	0.733883
41	H	0	4.538867	4.381834	1.19483
42	H	0	0.348918	4.422412	0.24459
43	H	0	2.427088	5.627871	0.936622
44	P	0	2.469956	0.035714	-0.19546
45	C	0	5.067304	1.807645	0.858557
46	H	0	5.57789	1.589447	-0.08451
47	H	0	5.715245	2.465491	1.442213
48	H	0	4.968948	0.866023	1.404444
49	C	0	4.635919	-2.16329	1.109718
50	H	0	4.37849	-1.41015	1.857929
51	H	0	3.82399	-2.89623	1.091777
52	H	0	5.538219	-2.67677	1.449516
53	C	0	2.091854	0.541595	3.109494
54	H	0	3.137223	0.751764	2.869421
55	H	0	1.504711	1.415583	2.812125

56	H	0	2.016498	0.446509	4.195231
57	F	0	1.602414	-1.24297	-2.37651
58	H	0	1.530721	0.782662	-2.34994
59	C	0	-6.98036	0.016859	-0.24331
60	C	0	-7.81367	-0.92505	-0.86875
61	C	0	-7.55488	0.97456	0.608314
62	C	0	-9.19391	-0.90811	-0.64574
63	H	0	-7.3823	-1.66494	-1.52472
64	C	0	-8.93554	0.988661	0.829382
65	H	0	-6.92331	1.701717	1.094415
66	C	0	-9.75762	0.047965	0.203274
67	H	0	-9.82354	-1.637	-1.13215
68	H	0	-9.36447	1.728768	1.487048
69	H	0	-10.8228	0.059424	0.375037

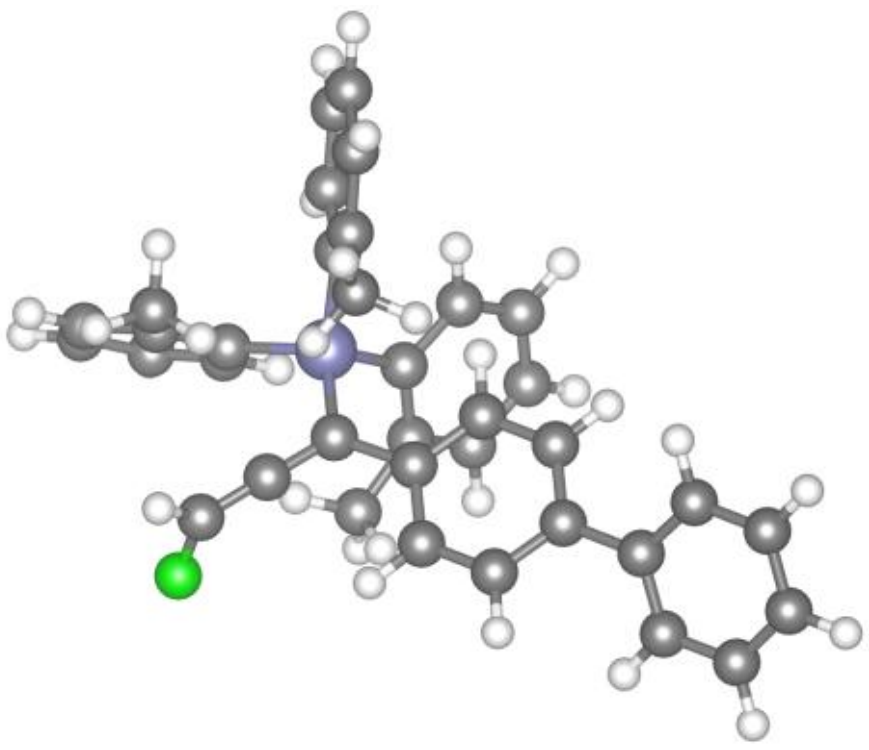

3l-PTol$_3$

1	C	0	0.830128	-1.68806	-1.73349
2	C	0	0.325879	-0.76924	-0.93647
3	C	0	-1.14026	-0.54467	-0.77506
4	C	0	-2.03435	-1.49943	-1.29424
5	C	0	-1.68324	0.566732	-0.11277
6	C	0	-3.40729	-1.34048	-1.16406
7	H	0	-1.64687	-2.37342	-1.80787
8	C	0	-3.06052	0.72208	0.017907
9	H	0	-1.04194	1.324393	0.31971
10	C	0	-3.96146	-0.22499	-0.50252
11	H	0	-4.07123	-2.08999	-1.58376
12	H	0	-3.44776	1.586893	0.547515
13	C	0	1.32064	-2.62014	-2.50456
14	F	0	1.554898	-3.87091	-2.03912
15	P	0	1.560931	0.12156	0.131673
16	C	0	0.959444	-0.13328	1.851436
17	C	0	0.782792	1.0001	2.666389
18	C	0	0.627291	-1.42296	2.349847
19	C	0	0.313948	0.882633	3.97103
20	H	0	1.003478	1.988135	2.283473
21	C	0	0.168559	-1.50327	3.673509

22	C	0	0.012515	-0.3788	4.479496
23	H	0	0.189717	1.772306	4.579779
24	H	0	-0.07818	-2.48374	4.070418
25	H	0	-0.34882	-0.48939	5.497468
26	C	0	3.23351	-0.60154	-0.07908
27	C	0	3.967686	-0.45261	-1.28281
28	C	0	3.821752	-1.23457	1.031146
29	C	0	5.25791	-1.00019	-1.32577
30	C	0	5.110508	-1.75566	0.958701
31	H	0	3.282697	-1.31243	1.96674
32	C	0	5.827858	-1.6454	-0.23086
33	H	0	5.826377	-0.90538	-2.24662
34	H	0	5.54408	-2.24086	1.827139
35	H	0	6.831381	-2.05303	-0.30696
36	C	0	1.798467	1.91942	-0.19443
37	C	0	2.789678	2.469117	0.650497
38	C	0	1.186302	2.733011	-1.18009
39	C	0	3.137361	3.812137	0.583278
40	H	0	3.298316	1.842495	1.375984
41	C	0	1.563455	4.087638	-1.21698
42	C	0	2.506549	4.631427	-0.3524
43	H	0	3.896332	4.206795	1.250704
44	H	0	1.10312	4.721791	-1.96875
45	H	0	2.763517	5.683861	-0.42417
46	C	0	0.677351	-2.7122	1.560189
47	H	0	0.794364	-3.55747	2.242951
48	H	0	-0.26133	-2.86244	1.014958
49	H	0	1.487683	-2.76006	0.833081
50	C	0	3.491056	0.301537	-2.5016
51	H	0	2.408999	0.298138	-2.6313
52	H	0	3.814731	1.347687	-2.44946
53	H	0	3.932696	-0.13033	-3.40379
54	C	0	0.197109	2.282622	-2.22823
55	H	0	0.396904	1.27653	-2.59862
56	H	0	-0.82988	2.300784	-1.85315
57	H	0	0.239583	2.964794	-3.08089
58	H	0	1.640908	-2.48053	-3.53236
59	C	0	-5.40953	-0.0627	-0.35805
60	C	0	-5.99759	1.218548	-0.32572
61	C	0	-6.25858	-1.1832	-0.24628
62	C	0	-7.37861	1.371664	-0.18876
63	H	0	-5.36797	2.089451	-0.42461
64	C	0	-7.63829	-1.02719	-0.10299
65	H	0	-5.82809	-2.17276	-0.25732
66	C	0	-8.20469	0.250355	-0.07478
67	H	0	-7.80459	2.363296	-0.17731

68	H	0	-8.26474	-1.90096	-0.0084
69	H	0	-9.27162	0.369495	0.033729

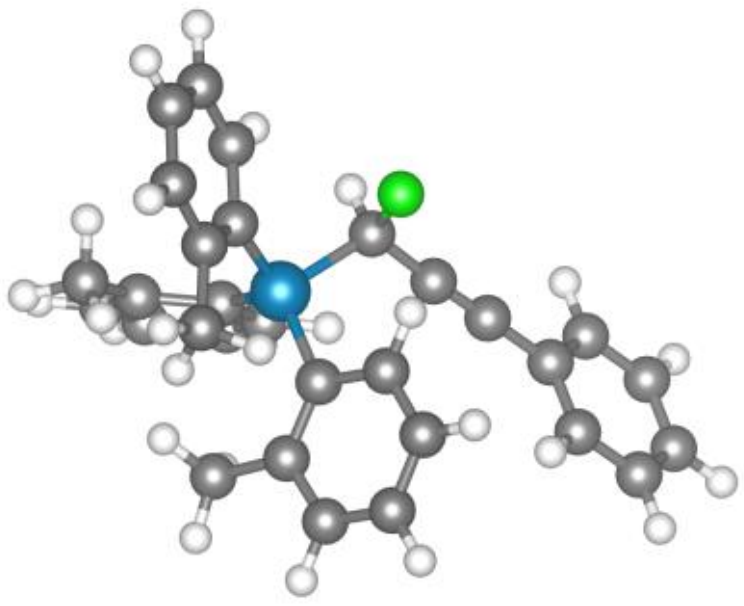

2l’-PTol$_3$

1	C	-0.17847	0.118275	-1.70604
2	C	-1.58863	0.117018	-1.39121
3	C	-2.78562	0.100209	-1.18564
4	C	-4.18975	0.094754	-0.93175
5	C	-4.74307	-0.83184	-0.02558
6	C	-5.03183	1.015986	-1.5863
7	C	-6.11388	-0.83178	0.217426
8	H	-4.09274	-1.54045	0.476544
9	C	-6.40074	1.00744	-1.33327
10	H	-4.60449	1.727374	-2.28555
11	C	-6.9436	0.085753	-0.43345
12	H	-6.53597	-1.54775	0.915982
13	H	-7.04555	1.719583	-1.83905
14	H	-8.01212	0.082371	-0.23992
15	C	0.213981	-1.12157	0.96885
16	C	-0.32599	-2.26751	0.349993
17	C	0.11563	-0.94967	2.371481
18	C	-0.96977	-3.24642	1.099786
19	H	-0.24268	-2.40225	-0.72239
20	C	-0.55007	-1.95214	3.093308
21	C	-1.08661	-3.08176	2.479686
22	H	-1.37428	-4.12429	0.606651
23	H	-0.63679	-1.83783	4.169794
24	H	-1.5881	-3.83442	3.080623
25	C	2.56304	-0.54929	-0.92383
26	C	3.398839	-1.55315	-0.37523
27	C	2.913334	0.084561	-2.13285
28	C	4.551489	-1.89272	-1.10073
29	C	4.068062	-0.27133	-2.82012
30	H	2.2923	0.872866	-2.54408
31	C	4.886666	-1.27454	-2.30251
32	H	5.205347	-2.65932	-0.69549
33	H	4.320333	0.230597	-3.74847
34	H	5.790381	-1.56944	-2.82724
35	C	1.109793	1.77452	0.388747

36	C	2.301344	2.3961	0.842215
37	C	-0.09806	2.499658	0.353766
38	C	2.217702	3.745053	1.217791
39	C	-0.14422	3.835323	0.739809
40	H	-1.01601	2.021614	0.032117
41	C	1.024671	4.462215	1.1675
42	H	3.120196	4.23642	1.56923
43	H	-1.0855	4.374083	0.706306
44	H	1.008962	5.504334	1.471875
45	P	0.99958	0.04551	-0.19112
46	C	3.640203	1.711573	0.976564
47	H	4.129856	1.582392	0.006415
48	H	4.300317	2.316872	1.602313
49	H	3.558233	0.724416	1.437023
50	C	3.164346	-2.25455	0.941368
51	H	2.92579	-1.5545	1.746461
52	H	2.343637	-2.97556	0.880169
53	H	4.064524	-2.80136	1.231439
54	C	0.692982	0.213186	3.140688
55	H	1.744035	0.388264	2.896623
56	H	0.150088	1.142372	2.943272
57	H	0.630883	0.014077	4.212946
58	F	0.143282	-0.98851	-2.50681
59	H	0.095392	1.02326	-2.25836

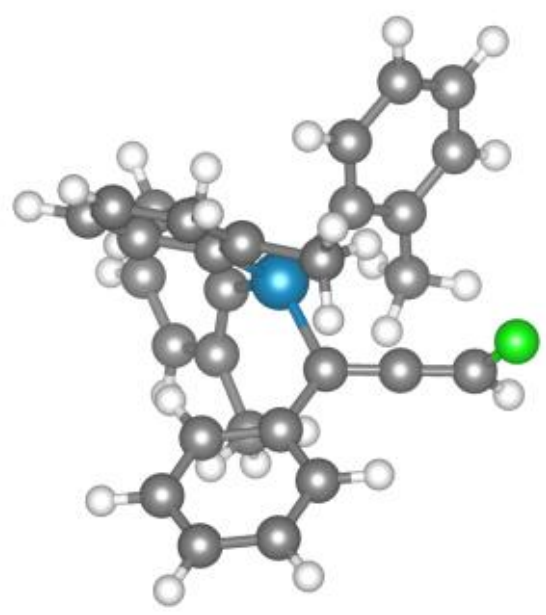

3l'-PTol$_3$

1	C	-0.24481	-1.71011	-1.73655
2	C	-0.78949	-0.72345	-1.05815
3	C	-2.24414	-0.40083	-1.10384
4	C	-3.12572	-1.35877	-1.64145
5	C	-2.77677	0.808307	-0.62537
6	C	-4.49246	-1.10842	-1.70754
7	H	-2.73218	-2.30403	-2.00104
8	C	-4.14774	1.055755	-0.6946
9	H	-2.13287	1.573904	-0.20798
10	C	-5.0098	0.100925	-1.23426
11	H	-5.15527	-1.86138	-2.12285
12	H	-4.53855	1.998574	-0.32489
13	H	-6.077	0.294651	-1.28329

14	C	0.30804	-2.70862	-2.36177
15	F	0.367199	-3.95119	-1.89663
16	P	0.355382	0.139217	0.14229
17	C	-0.54623	0.151244	1.744178
18	C	-0.71054	1.38335	2.403596
19	C	-1.10234	-1.03204	2.302757
20	C	-1.39332	1.46543	3.613347
21	H	-0.31305	2.29234	1.971693
22	C	-1.77285	-0.91234	3.529165
23	C	-1.92182	0.308653	4.181834
24	H	-1.50597	2.4276	4.102386
25	H	-2.19174	-1.81062	3.973277
26	H	-2.45134	0.353582	5.128711
27	C	1.921429	-0.79837	0.305229
28	C	2.868026	-0.87766	-0.74901
29	C	2.205825	-1.38116	1.553619
30	C	4.049479	-1.59154	-0.50312
31	C	3.396479	-2.06985	1.767994
32	H	1.503268	-1.28948	2.372454
33	C	4.318054	-2.18299	0.729209
34	H	4.777196	-1.67189	-1.30561
35	H	3.595648	-2.51176	2.738851
36	H	5.248143	-2.72352	0.876827
37	C	0.864632	1.845141	-0.31215
38	C	1.766279	2.391332	0.628055
39	C	0.520912	2.579681	-1.47312
40	C	2.286581	3.669841	0.469779
41	H	2.06625	1.81165	1.495274
42	C	1.068331	3.868579	-1.59977
43	C	1.923672	4.417619	-0.65035
44	H	2.972292	4.069356	1.209772
45	H	0.817187	4.443803	-2.48619
46	H	2.320819	5.417748	-0.79518
47	C	-1.06851	-2.40307	1.665469
48	H	-1.18765	-3.16963	2.435029
49	H	-1.90057	-2.51804	0.961486
50	H	-0.14738	-2.6219	1.123847
51	C	2.731271	-0.20173	-2.0931
52	H	1.703193	-0.10686	-2.4419
53	H	3.159808	0.806422	-2.05461
54	H	3.284532	-0.76264	-2.85065
55	C	-0.34916	2.090645	-2.60529
56	H	-0.18019	1.040397	-2.8455
57	H	-1.41281	2.211422	-2.37973
58	H	-0.14005	2.673963	-3.50502
59	H	0.782796	-2.64445	-3.31853

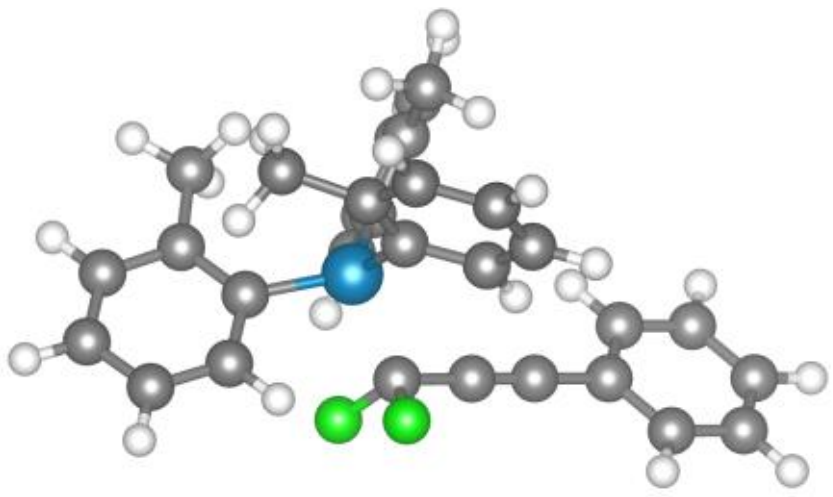

2n-PTol₃

1	C	-0.17313	0.209091	-1.65451
2	C	-1.58735	0.166186	-1.36394
3	C	-2.77778	0.132636	-1.12919
4	F	0.150749	1.387307	-2.28004
5	C	-4.17604	0.104855	-0.85506
6	C	-4.70395	-0.85341	0.032843
7	C	-5.03357	1.038254	-1.47088
8	C	-6.07053	-0.87153	0.296763
9	H	-4.03989	-1.57022	0.50398
10	C	-6.39785	1.008777	-1.1968
11	H	-4.62263	1.77298	-2.15545
12	C	-6.91772	0.056796	-0.31511
13	H	-6.4756	-1.61037	0.981274
14	H	-7.05684	1.728777	-1.67194
15	H	-7.98292	0.038239	-0.10521
16	C	0.173221	-1.20785	0.922715
17	C	-0.26487	-2.34512	0.212768
18	C	-0.04906	-1.10149	2.317507
19	C	-0.93371	-3.37668	0.862161
20	H	-0.07997	-2.43558	-0.85165
21	C	-0.73303	-2.15891	2.936892
22	C	-1.17298	-3.27728	2.232808
23	H	-1.2601	-4.24582	0.300607
24	H	-0.91286	-2.09795	4.006056
25	H	-1.69373	-4.07309	2.756693
26	C	2.578911	-0.55961	-0.84122
27	C	3.380395	-1.60353	-0.31161
28	C	2.975809	0.11921	-2.01204
29	C	4.545475	-1.93412	-1.02095
30	C	4.141465	-0.23305	-2.68226
31	H	2.376317	0.929855	-2.40815
32	C	4.925575	-1.27351	-2.18625
33	H	5.173417	-2.72994	-0.63154
34	H	4.428152	0.301949	-3.58164
35	H	5.8377	-1.56492	-2.69823
36	C	1.132298	1.717863	0.572008
37	C	2.317846	2.264695	1.128351
38	C	-0.0493	2.484349	0.525096

39	C	2.255467	3.587946	1.589795
40	C	-0.07439	3.790747	0.99993
41	H	-0.96448	2.063892	0.126276
42	C	1.089487	4.347061	1.527498
43	H	3.152127	4.024236	2.019559
44	H	-0.99528	4.362699	0.954888
45	H	1.090226	5.367102	1.899483
46	P	1.012342	0.03229	-0.11411
47	C	3.623051	1.524004	1.291867
48	H	4.131246	1.376591	0.334712
49	H	4.292372	2.098574	1.936472
50	H	3.489534	0.540172	1.751767
51	C	3.104479	-2.36277	0.965037
52	H	2.825185	-1.7062	1.793111
53	H	2.298865	-3.09235	0.839678
54	H	4.001617	-2.90954	1.2645
55	C	0.418482	0.04408	3.181308
56	H	1.484199	0.24983	3.047111
57	H	-0.12517	0.968371	2.965305
58	H	0.257185	-0.19763	4.234197
59	F	0.17213	-0.79843	-2.52369

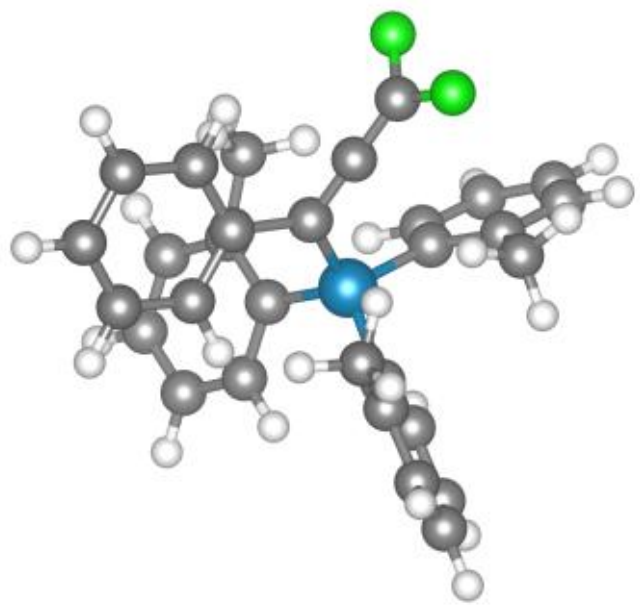

3n-PTol$_3$

1	C	-0.03591	-2.18563	-0.73689
2	C	-0.70041	-1.06068	-0.58416
3	C	-2.16809	-0.93672	-0.81381
4	C	-2.93441	-2.11551	-0.8979
5	C	-2.8229	0.300342	-0.93849
6	C	-4.30776	-2.05634	-1.11101
7	H	-2.44627	-3.07851	-0.7882
8	C	-4.20002	0.355127	-1.15325
9	H	-2.2699	1.231046	-0.88418
10	C	-4.94701	-0.81985	-1.23994
11	H	-4.88067	-2.97671	-1.16964
12	H	-4.68599	1.320664	-1.25272
13	H	-6.01943	-0.77409	-1.40325
14	C	0.633776	-3.297	-0.83648
15	F	0.752298	-4.19662	0.133301
16	P	0.287232	0.350065	0.144094

17	C	-0.72564	0.974653	1.545189
18	C	-1.02887	2.348141	1.583933
19	C	-1.23023	0.108048	2.553068
20	C	-1.80114	2.884833	2.609596
21	H	-0.67059	3.009686	0.806243
22	C	-1.99431	0.686866	3.57734
23	C	-2.28096	2.048984	3.615454
24	H	-2.02048	3.947589	2.617138
25	H	-2.37555	0.038121	4.360545
26	H	-2.87911	2.452291	4.426968
27	C	1.903366	-0.26058	0.755124
28	C	2.926628	-0.70038	-0.12423
29	C	2.140098	-0.20486	2.140711
30	C	4.137511	-1.11293	0.449778
31	C	3.360511	-0.60783	2.675604
32	H	1.375562	0.166851	2.811381
33	C	4.360313	-1.07357	1.82433
34	H	4.925045	-1.46485	-0.21049
35	H	3.521883	-0.55658	3.747446
36	H	5.315268	-1.39934	2.225528
37	C	0.698539	1.724473	-1.00389
38	C	1.486721	2.714267	-0.37591
39	C	0.385138	1.836775	-2.38037
40	C	1.919753	3.838079	-1.06864
41	H	1.766133	2.60698	0.667407
42	C	0.842086	2.986435	-3.04854
43	C	1.583337	3.978232	-2.41502
44	H	2.518567	4.58761	-0.56166
45	H	0.612952	3.08615	-4.10554
46	H	1.913782	4.847141	-2.97606
47	C	-1.04796	-1.39271	2.591694
48	H	-1.16435	-1.75127	3.617325
49	H	-1.81625	-1.88683	1.986125
50	H	-0.07644	-1.73464	2.232574
51	C	2.836639	-0.70029	-1.63223
52	H	1.832107	-0.87053	-2.01894
53	H	3.184514	0.25936	-2.03165
54	H	3.483453	-1.47893	-2.04449
55	C	-0.36212	0.816888	-3.20465
56	H	-0.09805	-0.20972	-2.94849
57	H	-1.44529	0.918395	-3.08955
58	H	-0.13314	0.963582	-4.26274
59	F	1.299213	-3.68872	-1.91676

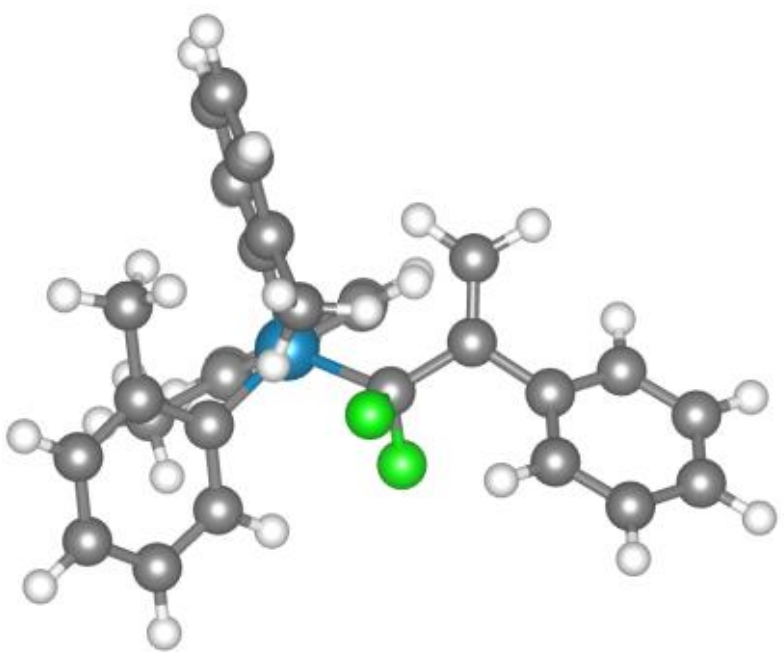

4α-PTol₃

1	C	-2.04397	-0.90836	-1.81447
2	H	-2.87455	-1.14443	-2.47118
3	H	-1.05315	-1.02057	-2.23412
4	C	-3.65888	-0.33418	-0.0238
5	C	-4.02916	-0.75291	1.266331
6	C	-4.63505	0.255174	-0.84561
7	C	-5.34223	-0.60047	1.709838
8	H	-3.29749	-1.21127	1.921468
9	C	-5.94746	0.404011	-0.39922
10	H	-4.35788	0.612795	-1.83241
11	C	-6.30642	-0.0242	0.880306
12	H	-5.61165	-0.93727	2.706566
13	H	-6.68605	0.864649	-1.04872
14	H	-7.32744	0.095381	1.230443
15	C	-2.27358	-0.51652	-0.55423
16	C	-1.15255	-0.24642	0.427743
17	F	-1.48236	0.832799	1.211771
18	F	-1.02147	-1.32034	1.288786
19	P	0.656035	0.140576	-0.10372
20	C	0.746096	1.802524	-0.81992
21	C	-0.15487	2.218633	-1.81634
22	C	1.769477	2.666276	-0.39232
23	C	-0.03266	3.491544	-2.36807
24	H	-0.94749	1.562227	-2.15618
25	C	1.882685	3.936013	-0.95705
26	C	0.983067	4.350054	-1.94014
27	H	-0.73412	3.812045	-3.13166
28	H	2.674058	4.599734	-0.62387
29	H	1.072278	5.341435	-2.37367
30	C	1.593626	0.079749	1.450603
31	C	2.698494	-0.77972	1.560032
32	C	1.232945	0.904395	2.533262
33	C	3.432269	-0.81622	2.745793
34	C	1.973317	0.855339	3.711415
35	H	0.386552	1.576466	2.46216
36	C	3.070568	-0.00335	3.820043
37	H	4.285752	-1.48197	2.824791

38	H	1.691156	1.489859	4.545538
39	H	3.643227	-0.0359	4.741871
40	C	1.269737	-1.12603	-1.24198
41	C	1.963724	-0.74198	-2.40035
42	C	1.075535	-2.4891	-0.94862
43	C	2.459405	-1.72148	-3.26101
44	H	2.11678	0.306142	-2.63191
45	C	1.575435	-3.45481	-1.81803
46	C	2.265818	-3.0729	-2.97216
47	H	2.995172	-1.4245	-4.15688
48	H	1.422704	-4.50579	-1.59446
49	H	2.651498	-3.83106	-3.64684
50	C	0.306924	-2.92147	0.313869
51	H	0.569214	-2.27929	1.128551
52	H	-0.74526	-2.85443	0.131326
53	H	0.56278	-3.93112	0.558917
54	C	3.113139	-1.68269	0.383466
55	H	2.437187	-2.50911	0.31266
56	H	3.084317	-1.11942	-0.52582
57	H	4.10601	-2.04693	0.546071
58	C	2.769237	2.225777	0.693061
59	H	2.281905	2.219888	1.645622
60	H	3.127851	1.242693	0.469811
61	H	3.592592	2.90869	0.717874

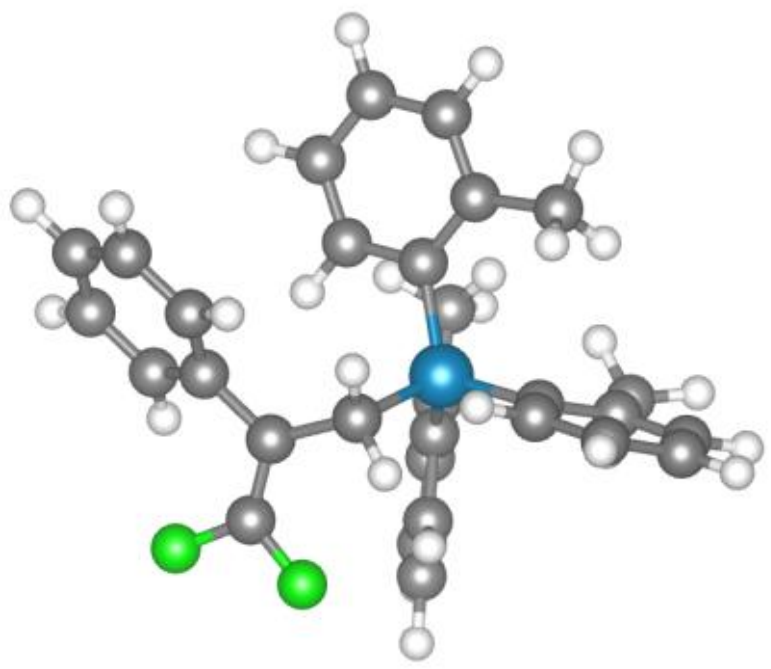

4γ-PTol$_3$

1	C	1.790347	-2.38953	-1.48352
2	C	2.920254	-0.29345	-0.95819
3	C	3.785181	-0.70106	0.07129
4	C	3.249813	0.854347	-1.698
5	C	4.946577	0.019398	0.351827
6	H	3.542821	-1.58133	0.658192
7	C	4.411928	1.572274	-1.41669
8	H	2.60607	1.182898	-2.50835
9	C	5.264351	1.158881	-0.3903
10	H	5.601341	-0.31025	1.153276
11	H	4.653298	2.453331	-2.00416
12	H	6.168514	1.71927	-0.17157
13	F	2.905662	-3.09591	-1.38226

14	F	0.793463	-3.18717	-1.85612
15	C	1.68915	-1.07141	-1.28272
16	C	0.361216	-0.4052	-1.55918
17	H	-0.25498	-1.06305	-2.17317
18	H	0.515781	0.516557	-2.12707
19	P	-0.77375	0.127301	-0.14088
20	C	-0.72679	-1.18851	1.136227
21	C	-0.60478	-1.03705	2.540152
22	C	-0.87739	-2.47587	0.580177
23	C	-0.5838	-2.21154	3.311937
24	C	-0.86501	-3.61572	1.375238
25	H	-1.02436	-2.59683	-0.48669
26	C	-0.70096	-3.4802	2.753297
27	H	-0.48938	-2.11188	4.389549
28	H	-0.98406	-4.59353	0.919943
29	H	-0.68352	-4.35672	3.394066
30	C	-2.43968	0.158371	-0.91383
31	C	-3.64293	0.004461	-0.17913
32	C	-2.491	0.333909	-2.31024
33	C	-4.847	-0.00137	-0.89847
34	C	-3.70414	0.334244	-2.99279
35	H	-1.58612	0.490729	-2.88368
36	C	-4.88871	0.153999	-2.28199
37	H	-5.7746	-0.12198	-0.34642
38	H	-3.71633	0.475934	-4.06859
39	H	-5.84374	0.145522	-2.79847
40	C	-0.16656	1.753966	0.444267
41	C	-0.83253	3.000606	0.313425
42	C	1.121765	1.702019	1.010685
43	C	-0.14945	4.140742	0.769419
44	C	1.768687	2.851331	1.450251
45	H	1.627976	0.750503	1.122426
46	C	1.125054	4.080982	1.326377
47	H	-0.64414	5.103193	0.679785
48	H	2.761728	2.779751	1.881325
49	H	1.610351	4.992302	1.662803
50	C	-0.52955	0.269572	3.296621
51	H	0.50587	0.606352	3.413184
52	H	-1.08089	1.079027	2.819106
53	H	-0.9383	0.128987	4.300728
54	C	-3.71959	-0.12592	1.322392
55	H	-3.45469	-1.13426	1.6554
56	H	-3.05797	0.57607	1.835106
57	H	-4.73919	0.075877	1.659356
58	C	-2.21189	3.208959	-0.26624
59	H	-2.26558	2.920315	-1.31922

60	H	-2.97626	2.638371	0.266709
61	H	-2.4808	4.265522	-0.19865

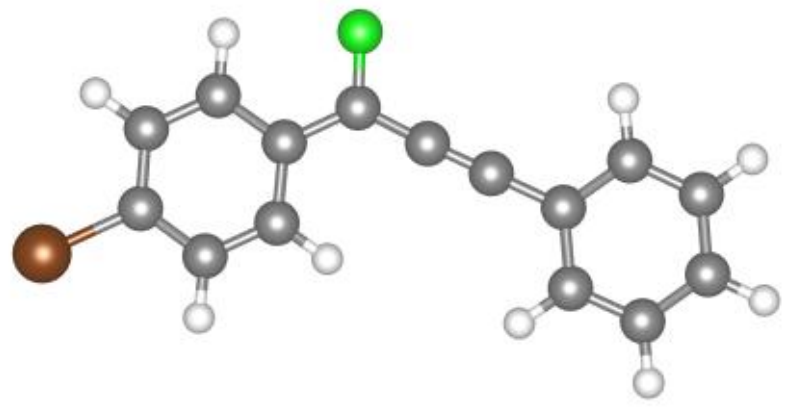

Int$_{1a}$

1	C	1.723876	0.995348	-0.00034
2	C	0.507823	1.62348	0.000186
3	C	2.838051	0.473871	-0.00027
4	F	0.544034	2.950017	0.000618
5	C	-0.78111	1.020522	0.000206
6	C	-0.91239	-0.39099	-0.00119
7	C	-1.93842	1.838281	0.001504
8	C	-2.16819	-0.9685	-0.00137
9	H	-0.02893	-1.02013	-0.00222
10	C	-3.19402	1.259409	0.001418
11	H	-1.84131	2.917076	0.002633
12	C	-3.2981	-0.13802	-5.4E-05
13	H	-2.27688	-2.04648	-0.00254
14	H	-4.08259	1.879193	0.002497
15	C	4.101516	-0.119	-0.00018
16	C	5.260829	0.700228	-0.00194
17	C	4.223792	-1.53325	0.001811
18	C	6.512909	0.106602	-0.00177
19	H	5.150606	1.778928	-0.00349
20	C	5.485405	-2.10621	0.002127
21	H	3.328863	-2.14591	0.003157
22	C	6.625102	-1.29034	0.000308
23	H	7.405169	0.723539	-0.00321
24	H	5.590361	-3.18578	0.003783
25	H	7.609833	-1.74743	0.000514
26	BR	-5.01925	-0.92948	-0.00027

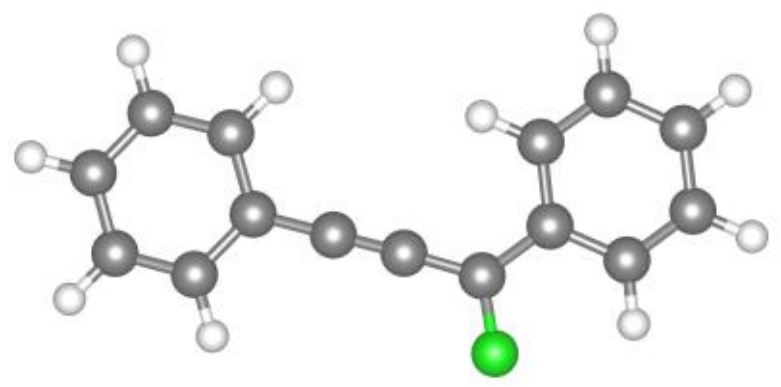

Int$_{1c}$

1	C	0.008156	0.657322	-0.00037
2	C	-1.28672	1.083939	0.000051
3	C	1.20578	0.396792	-0.00045
4	F	-1.49807	2.420519	0.00051
5	C	-2.44068	0.2621	0.000137
6	C	-2.32308	-1.15224	-0.00069

7	C	-3.72444	0.868646	0.001127
8	C	-3.46594	-1.93319	-0.00057
9	H	-1.34104	-1.60906	-0.00143
10	C	-4.85749	0.07296	0.001287
11	H	-3.8065	1.947285	0.001793
12	C	-4.73152	-1.32467	0.00043
13	H	-3.38487	-3.01238	-0.00124
14	H	-5.83936	0.52822	0.002051
15	H	-5.6216	-1.94251	0.000533
16	C	2.559642	0.066845	-0.00043
17	C	3.538264	1.097751	-0.00046
18	C	2.961314	-1.29647	-0.00028
19	C	4.883729	0.761567	-0.00033
20	H	3.220164	2.13239	-0.00058
21	C	4.31212	-1.61002	-0.00014
22	H	2.206938	-2.0732	-0.00025
23	C	5.271334	-0.58639	-0.00017
24	H	5.635107	1.541139	-0.00036
25	H	4.626247	-2.64602	0.000001
26	H	6.324704	-0.8402	-5.5E-05

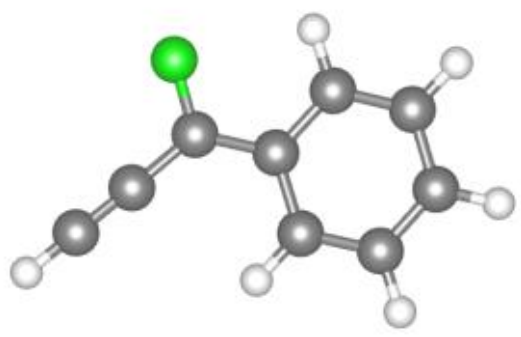

Int1f'

1	C	-2.37495	-0.44986	0.000041
2	C	-1.30446	0.436497	-0.00012
3	C	-3.33922	-1.19446	0.000333
4	H	-4.18475	-1.85384	0.000635
5	F	-1.66677	1.757636	-0.00037
6	C	0.064236	0.146832	0.000002
7	C	0.501148	-1.21786	-0.00016
8	C	1.026237	1.208251	0.000249
9	C	1.855271	-1.49941	-0.00021
10	H	-0.23118	-2.01713	-0.0003
11	C	2.375001	0.902015	0.000272
12	H	0.692296	2.238014	0.000455
13	C	2.790629	-0.44526	-2E-06
14	H	2.197745	-2.52666	-0.0004
15	H	3.112659	1.695109	0.000496
16	H	3.850783	-0.67469	-4.5E-05

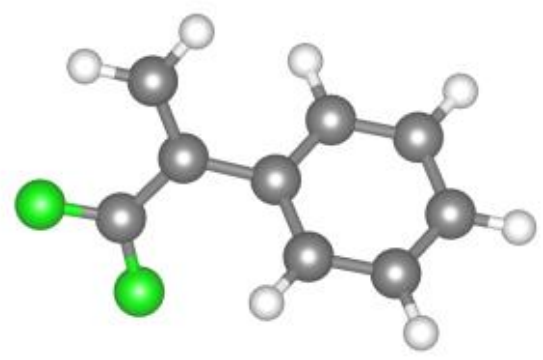

Int$_4$

1	C	-1.02966	0.577075	0.227213
2	C	-1.51214	1.78404	0.658386
3	H	-0.82287	2.555632	0.985806
4	H	-2.57322	2.011215	0.685178
5	C	-2.00923	-0.35802	-0.1701
6	C	0.415796	0.241088	0.11203
7	C	0.928208	-0.96494	0.614036
8	C	1.278882	1.174193	-0.48175
9	C	2.294156	-1.22729	0.523329
10	H	0.271737	-1.68585	1.091867
11	C	2.643101	0.903199	-0.56953
12	H	0.878292	2.098942	-0.88611
13	C	3.151813	-0.29668	-0.06781
14	H	2.688119	-2.15753	0.920196
15	H	3.305497	1.626075	-1.03519
16	H	4.214605	-0.50715	-0.13845
17	F	-1.72325	-1.50926	-0.63786
18	F	-3.26872	-0.15045	-0.10638

References

[1] G. Sheldrick, *Acta Crystallographica Section A* **2015**, *71*, 3-8.
[2] G. Sheldrick, *Acta Crystallographica Section C* **2015**, *71*, 3-8.
[3] O. V. Dolomanov, L. J. Bourhis, R. J. Gildea, J. A. K. Howard, H. Puschmann, *J. Appl. Crystallogr.* **2009**, *42*, 339-341.
[4] S. Mizuta, I. S. R. Stenhagen, M. O'Duill, J. Wolstenhulme, A. K. Kirjavainen, S. J. Forsback, M. Tredwell, G. Sandford, P. R. Moore, M. Huiban, S. K. Luthra, J. Passchier, O. Solin, V. Gouverneur, *Org. Lett.* **2013**, *15*, 2648-2651.
[5] X. Li, S. Li, S. Sun, F. Yang, W. Zhu, Y. Zhu, Y. Wu, Y. Wu, *Adv. Synth. Catal.* **2016**, *358*, 1699-1704.
[6] T. M. Milzarek, J. Waser, *Angew. Chem. Int. Ed.* **2023**, *62*, e202306128.
[7] D. P. Hari, P. Caramenti, L. Schouwey, M. Chang, S. Nicolai, D. Bachert, T. Wright, C. Orella, J. Waser, *Organic Process Research & Development* **2020**, *24*, 106-110.
[8] A. G. Massey, A. J. Park, *J. Organomet. Chem.* **1964**, *2*, 245-250.
[9] M. Cloutier, M. Mamone, J.-F. Paquin, *Chem. Commun.* **2020**, *56*, 5969-5972.
[10] Q. Xie, Z. Zhu, L. Li, C. Ni, J. Hu, *Angew. Chem. Int. Ed.* **2019**, *58*, 6405-6410.
[11] Gaussian 16, Revision A.03, M. J. Frisch, G. W. Trucks, H. B. Schlegel, G. E. Scuseria, M. A. Robb, J. R. Cheeseman, G. Scalmani, V. Barone, G. A. Petersson, H. Nakatsuji, X. Li, M. Caricato, A. V. Marenich, J. Bloino, B. G. Janesko, R. Gomperts, B. Mennucci, H. P. Hratchian, J. V. Ortiz, A. F. Izmaylov, J. L. Sonnenberg, D. Williams-Young, F. Ding, F. Lipparini, F. Egidi, J. Goings, B. Peng, A. Petrone, T. Henderson, D. Ranasinghe, V. G. Zakrzewski, J. Gao, N. Rega, G. Zheng, W. Liang, M. Hada, M. Ehara, K. Toyota, R. Fukuda, J. Hasegawa, M. Ishida, T. Nakajima, Y. Honda, O. Kitao, H. Nakai, T. Vreven, K. Throssell, J. A. Montgomery, Jr., J. E. Peralta, F. Ogliaro, M. J. Bearpark, J. J. Heyd, E. N. Brothers, K. N. Kudin, V. N. Staroverov, T. A. Keith, R. Kobayashi, J. Normand, K. Raghavachari, A. P. Rendell, J. C. Burant, S. S. Iyengar, J. Tomasi, M. Cossi, J. M. Millam, M. Klene, C. Adamo, R. Cammi, J. W. Ochterski, R. L. Martin, K. Morokuma, O. Farkas, J. B. Foresman, and D. J. Fox, Gaussian, Inc., Wallingford CT, 2016.
[12] (a) A. D. Becke, *J. Chem. Phys*., **1993**, *98*, 5648–5652. (b) C. Lee, W. Yang, R. G. Parr, *Phys. Rev. B*, **1988**, *37*, 785–789. (c) B. Miehlich, A. Savin, H. Stoll, H. Preuss, *Chem. Phys. Lett.* **1989**, *157*, 200–206. (d) P. J. Stephens, F. J. Devlin, C. F. Chabalowski, M. J. Frisch, *J. Phys. Chem.*, **1994**, *98*, 11623−11627
[13] A. V. Marenich, C. J. Cramer, D. G. Truhlar, *J. Phys. Chem. B,* **2009**, *113*, 6378.
[14] (a) P. K. Chattaraj and S. Giri, *Annu. Rep. Prog. Chem., Sect. C,* **2009**, *105*, 13–39. (b) J. Sánchez-Márquez, D. Zorrilla, A. Sánchez-Coronilla, D. M. de los Santos, J. Navas, C. Fernández-Lorenzo, R. Alcántara, J. Martín-Calleja *J. Mol. Model.* **2014**, *20*, 2492.
